# Mindfulness-Based Blood Pressure Reduction (MB-BP): Stage 1 single-arm clinical trial

**DOI:** 10.1371/journal.pone.0223095

**Published:** 2019-11-27

**Authors:** Eric B. Loucks, William R. Nardi, Roee Gutman, Ian M. Kronish, Frances B. Saadeh, Yu Li, Anna E. Wentz, Julie Webb, David R. Vago, Abigail Harrison, Willoughby B. Britton

**Affiliations:** 1 Department of Epidemiology, Brown University School of Public Health, Providence, Rhode Island, United States of America; 2 Department of Behavioral Sciences, Brown University School of Public Health, Providence, Rhode Island, United States of America; 3 Department of Medicine, The Warren Alpert Medical School of Brown University, Providence, Rhode Island, United States of America; 4 Department of Biostatistics, Brown University School of Public Health, Providence, Rhode Island, United States of America; 5 Department of Medicine, Columbia University Medical Center Vanderbilt University School of Medicine, Nashville, Tennessee, United States of America; 6 Department of Psychiatry and Behavioral Sciences, Vanderbilt University School of Medicine, Nashville, Tennessee, United States of America; 7 Department of Psychiatry and Human Behavior, The Warren Alpert Medical School of Brown University, Providence, Rhode Island, United States of America; University of California Los Angeles, UNITED STATES

## Abstract

**Background and objectives:**

Impacts of mindfulness-based programs on blood pressure remain equivocal, possibly because the programs are not adapted to engage with determinants of hypertension, or due to floor effects. Primary objectives were to create a customized Mindfulness-Based Blood Pressure Reduction (MB-BP) program, and to evaluate acceptability, feasibility, and effects on hypothesized proximal self-regulation mechanisms. Secondary outcomes included modifiable determinants of blood pressure (BP), and clinic-assessed systolic blood pressure (SBP).

**Methods:**

This was a Stage 1 single-arm trial with one year follow-up. Focus groups and in-depth interviews were performed to evaluate acceptability and feasibility. Self-regulation outcomes, and determinants of BP, were assessed using validated questionnaires or objective assessments. The MB-BP curriculum was adapted from Mindfulness-Based Stress Reduction to direct participants’ mindfulness skills towards modifiable determinants of blood pressure.

**Results:**

Acceptability and feasibility findings showed that of 53 eligible participants, 48 enrolled (91%). Of these, 43 (90%) attended at least 7 of the 10 MB-BP classes; 43 were followed to one year (90%). Focus groups (n = 19) and semi-structured interviews (n = 10) showed all participants viewed the delivery modality favorably, and identified logistic considerations concerning program access as barriers. A priori selected primary self-regulation outcomes showed improvements at one-year follow-up vs. baseline, including attention control (Sustained Attention to Response Task correct no-go score, p<0.001), emotion regulation (Difficulties in Emotion Regulation Score, p = 0.02), and self-awareness (Multidimensional Assessment of Interoceptive Awareness, p<0.001). Several determinants of hypertension were improved in participants not adhering to American Heart Association guidelines at baseline, including physical activity (p = 0.02), Dietary Approaches to Stop Hypertension-consistent diet (p<0.001), and alcohol consumption (p<0.001). Findings demonstrated mean 6.1 mmHg reduction in SBP (p = 0.008) at one year follow-up; effects were most pronounced in Stage 2 uncontrolled hypertensives (SBP≥140 mmHg), showing 15.1 mmHg reduction (p<0.001).

**Conclusion:**

MB-BP has good acceptability and feasibility, and may engage with self-regulation and behavioral determinants of hypertension.

## Introduction

The annual cost to society for high blood pressure in the United States was $53.2 billion as of 2014.[1] Elimination of hypertension was estimated to have a larger impact on cardiovascular disease (CVD) mortality than the removal of any other CVD risk factor in females, and any risk factor, except smoking, among males.[2] Elevated blood pressure is critically important to population health. Remarkably, we know much of what causes it. For example, diet, physical activity, alcohol consumption, and antihypertensive medication adherence are major determinants of blood pressure.[3] However, global estimates show that a large proportion of society world-wide (31% of adults) had Stage 2 hypertension (>140/90 mmHg) in 2010.[4] In the United States, only about half of the population with hypertension has it controlled.[1] Hypertension has become one of the greatest international noncommunicable disease priorities to prevent and treat.[5]

**Fig 1:**
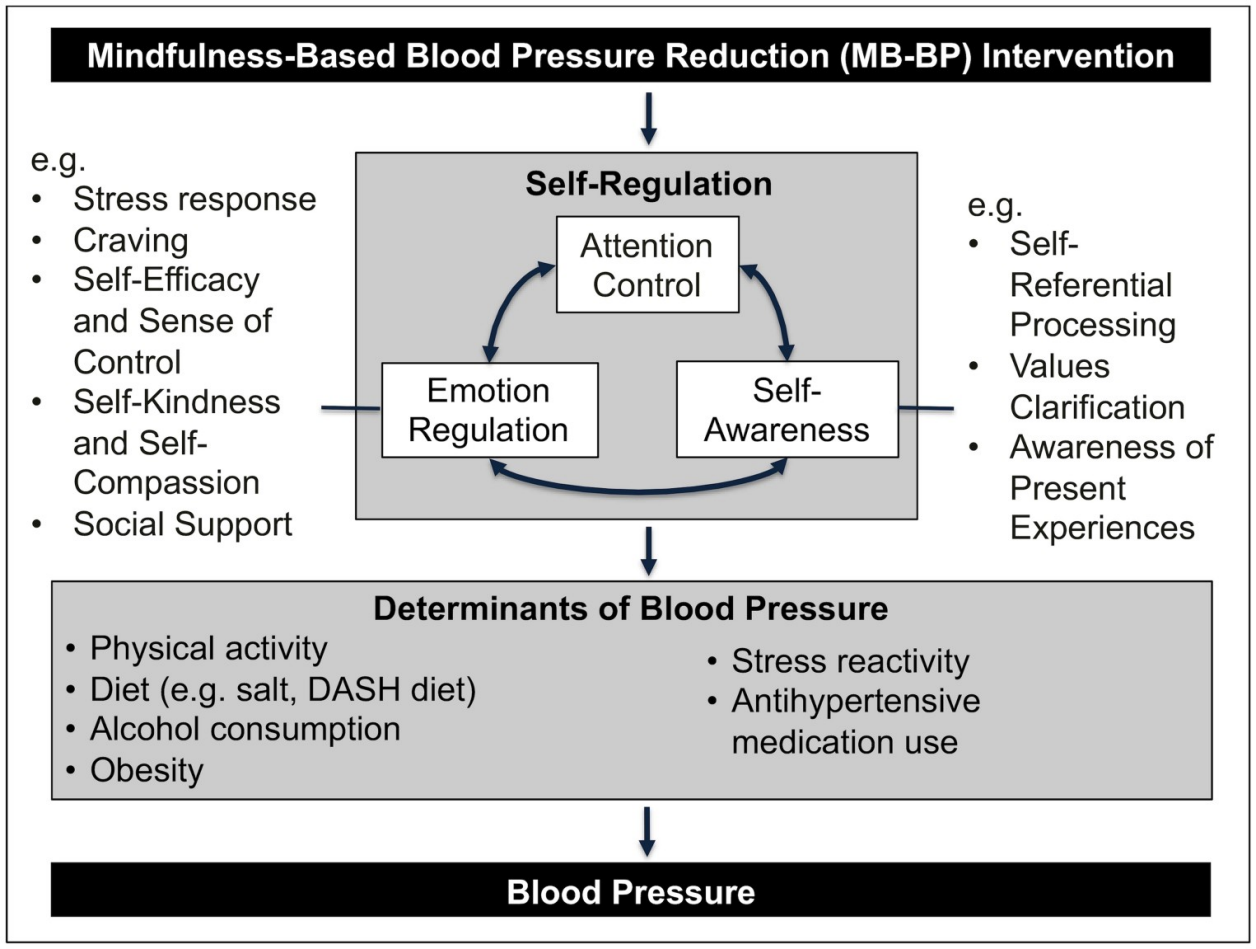
Theoretical framework of mechanisms through which Mindfulness-Based Blood Pressure Reduction (MB-BP) program may influence blood pressure.

Mindfulness, defined as “paying attention, on purpose, in the present moment, nonjudgmentally”[6] may be a framework through which to engage healthy lifestyles in societies that promote behavioral determinants of hypertension, including obesity, high salt intake, sedentary activities, and excessive alcohol consumption.[7] In 2015, a consensus theoretical framework through which mindfulness could influence cardiovascular disease was proposed, with relevance to blood pressure specifically shown in Fig 1.[7] This framework builds on existing models of mindfulness [8–10] and hypothesizes that mindfulness can influence behavioral underpinnings of hypertension through improving self-regulation, including via enhancing attention control, self-awareness and emotion regulation.[7]

Preliminary research suggests that mindfulness meditation may influence blood pressure, but evidence is currently equivocal. Specifically, a 2014 systematic review and meta-analysis of randomized controlled trials showed that in 5 studies (n = 286) there was a small but significant overall improvement in blood pressure for participants that went through mindfulness-based interventions *vs*. controls (standardized mean difference of -0.78; 95% CI: -1.46, -0.09).[11] When the study with the largest effects was removed from the meta-analysis, findings became null.[11] Furthermore, none of the mindfulness interventions were customized to participants with elevated blood pressure, but instead taught mindfulness techniques applied to either stress reduction or preventing depression relapse.[11] A 2018 systematic review suggested similar findings.[12] Customization of mindfulness interventions for specific patient populations has evidence to be effective, as a recent systematic review and meta-analyses demonstrated for Mindfulness-Based Cognitive Therapy effects on preventing depression relapse.[13] Consequently, we hypothesized that a mindfulness-based program adapted to those with elevated blood pressure, that trains participants in mindfulness skills of attention control, self-awareness and emotion regulation as well as the application of those skills in relation to modifiable determinants of blood pressure (e.g. physical activity, diet, stress reactivity, antihypertensive medication adherence; Fig 1), may boost effects.[7] This intervention is named Mindfulness-Based Blood Pressure Reduction (MB-BP).

The primary objective of this Stage 1 clinical trial was to create an adapated Mindfulness-Based Blood Pressure Reduction (MB-BP) program, and evaluate acceptability, feasibility, and effects on hypothesized primary proximal self-regulation mechanisms. Primary outcomes, as registered *a priori* on ClinicalTrials.gov (registration #NCT02702258) were attention control, self-awareness and emotion regulation. These proximal modifiable mechanisms were selected as primary outcomes in order to be consistent with the National Institutes of Health Science of Behavior Change (SOBC) approach to experimental medicine, that emphasizes early intervention research to evaluate engagement with proximal mechanisms.[14, 15] Secondary distal outcomes included more established modifiable determinants of blood pressure including Dietary Approaches to Stop Hypertension (DASH)-consistent diet, body mass index, physical activity, alcohol consumption, perceived stress, and antihypertensive medication adherence.[3] Secondary analyses also evaluated the role of MB-BP on clinic-assessed systolic blood pressure.

## Methods

### Study sample description

Participants were recruited and assessed during 2016–2017. The most common sources of participants were flyer/recruitment cards distributed throughout Rhode Island and Massachusetts (43% of participants), referral from friends, family members, coworkers or other person (19%), referral from a former or current participant (16%), and from a primary care practitioner or other health care professional (16%).

Inclusion criteria were: (1) Hypertension/prehypertension (SBP≥120 mmHg systolic or DBP ≥80 mmHg or prescribed antihypertensive medication for treatment of hypertension); (2) Able to speak, read, and write in English; (3) All adults (≥18 years of age), genders and racial/ethnic groups were eligible to be included.

Exclusion Criteria were: (1) Current regular meditation practice (>once/week); (2) Serious medical illness precluding regular class attendance; (3) Current substance abuse suicidal ideation or eating disorder; and (4) History of bipolar or psychotic disorders or self-injurious behaviors. These participants were excluded following standard guidelines [16] because of risk for disrupting group participation, requiring additional or specialized treatment beyond capacity of this study, or already participating in practices similar to the intervention. The study protocol was approved by the institutional review board at Brown University (protocol #1412001171) on September 3, 2015. Participants provided written informed consent.

In order to remove potential biases of selecting primary outcomes based on initial observed effects in this study, the trial was registered prior to the first follow-up assessment. Specifically, the protocol was first submitted to ClinicalTrials.gov on March 3, 2016, and was posted on March 8, 2016. The first baseline assessment was performed on February 9, 2016. The first three-month follow-up assessment was performed on May 2, 2016, and the last one-year follow-up assessment took place on October 27, 2017. The authors confirm that all ongoing and related trials for this intervention are registered.

### Intervention description

This study adapted Mindfulness-Based Stress Reduction (MBSR) for participants with prehypertension/hypertension, creating MB-BP. Specifically, MB-BP is based on, and time-matched to, the standardized MBSR intervention described elsewhere.[17–20] It consists of an orientation session, eight 2.5-hour weekly group sessions, and a 7.5-hour one-day session, and is led by a qualified MBSR instructor with expertise in cardiovascular disease etiology, treatment, and prevention. MB-BP and MBSR contain similar instruction and practices in mindfulness meditation, and conversations about stress and coping. Students learn a range of mindfulness skills including body scan exercises, meditation and yoga. Homework consists of practicing mindfulness skills for ≥45 min/day, 6 days/week.

The unique areas of MB-BP are education on hypertension risk factors, hypertension health effects, and specific mindfulness modules focused on awareness of diet, physical activity, medication adherence, alcohol consumption, stress, and social support for behavior change. MB-BP builds a foundation of mindfulness skills (e.g. meditation, yoga, self-awareness, attention control, emotion regulation) through the MBSR curriculum. MB-BP directs those skills towards participants’ relationship with their risk factors for hypertension (Fig 1).

MB-BP participants have their blood pressure and hypertension risk factors assessed at baseline, and are provided with this information during the orientation session. During this session, the importance of hypertension for health and mortality is described, along with hypertension risk factors. This phase aims to engage participants’ interest in hypertension risk factors, and increase motivation for behavior change. MB-BP encourages participants to explore personal readiness for change in the different hypertension risk factors, and explore utilizing mindfulness practices to engage with those risk factors that they choose to. Instructors hold twenty-minute one-on-one interviews with each participant at the beginning of the course to foster a relationship between the instructor and participant, identify reasons for participation, and pinpoint opportunities for the instructor to customize the course to individual participants.

The course focuses on different determinants of BP each week. However, four common themes exist across all BP determinants, including: (1) Awareness of thoughts, emotions and physical sensations particularly surrounding determinants of BP such as food overconsumption, sedentary activities, alcohol consumption, and antihypertensive medication adherence. (2) Craving, particularly for determinants of BP such as overconsumption of palatable foods (e.g. those high in salt or sugar), sedentary activities, and alcohol consumption. (3) The impact of bringing mindfulness to every moment, particularly in relation to BP determinants, recognizing that this present moment is influenced by prior moments, including what we ate, the physical activity we had, and the amount of alcohol consumed. Participants are trained to bring non-judgmental attention to the often short-term pleasures of overconsumption of foods, sedentary activities, heavy alcohol consumption, or not taking antihypertensive medications, as well as to bring non-judgmental attention to the longer term suffering associated with these activities. Through this process, participants are encouraged to reflect on whether behavioral choices provide more benefit or harm to their well-being, and to choose beneficial behaviors. (4) Self-compassion: as self-regulatory and self-awareness skills increase as a result of the mindfulness practices, the curriculum emphasizes that it is common for participants to start caring for themselves in kinder ways. It is a way of better knowing ourselves, and through knowing ourselves in each moment, we often want to care for ourselves in each moment. This may mean taking medication that will support health, or being more physically active, eating more healthily, or consuming alcohol in more moderate amounts. As a whole, the MB-BP program trains participants in mindfulness skills, and then supports them to apply those skills to determinants of BP most relevant in their lives. After the 8-week course is completed, the only curriculum offerings are optional bimonthly MB-BP community group meetings, one-day retreats three times per year, and website access to new bimonthly meditations and talks.

The curriculum guide and MB-BP instructor certification program can be accessed by contacting the lead author. Specific customizations of MB-BP from MBSR are shown in S1 Table. The MB-BP intervention took place in classrooms at the Brown University School of Public Health, in Providence, RI.

### Measures

All assessments were performed by trained research assistants. Research assistants were instructed in demonstrating equipoise (i.e. genuine uncertainty in the expert medical community over whether the MB-BP treatment will be beneficial) during all participant interactions. Regular quality control and assurance evaluations were performed on research assistants’ assessment accuracy, adherence to protocols, and equipoise. Accuracy of assessment equipment was evaluated at the beginning of every intervention cycle when baseline assessments were performed (approximately every 4 months), including blood pressure monitors, stadiometers and weighing scales. In-person assessments took place in a dedicated assessment room at the Brown University School of Public Health. Most self-report questionnaires were administered via Qualtrics, accessible by participants in their home environment through computer or smart phone.

#### Feasibility and acceptability

Focus group discussions (FGD) were held at the Brown University School of Public Health. The 48 participants who completed the MB-BP program were invited to participate in the FGD, of which 19 accepted. In order to minimize selection bias, 13 of the 29 participants who did not participate in the FGD were randomly selected to be invited to participate with in-depth interviews (IDI). Selected IDI participants were contacted by phone and asked if they would be interested in participating. Ten of the thirteen contacted agreed to be interviewed (three were unable to be reached despite three or more attempts). IDI’s were conducted by audio-only Zoom calls. Both FGD and IDI were implemented by qualitative researchers.

Qualitative methods explored *a priori* domains consistent with intervention acceptability (e.g. support materials, intervention delivery modality), and areas identified as specific to mindfulness interventions (e.g. instructor competency, mindfulness practice adherence). FGDs and IDIs were administered using standardized procedures (see S2 Table).[21–24] FGD participants rated the usefulness of MB-BP customizations via a *closed card sort activity*, where they were asked to categorize the customizations as “very useful”, “somewhat useful”, “not useful” (S3 Table).[25, 26] Finally, FGD and IDI participants completed a short closed- and open-ended survey assessing opinions of MB-BP class duration (e.g. “Each weekly session was 2.5 hours long. Do you think the session should be 2 hours, 2.5 hours, or 3 hours, and why? The retreat was scheduled as being 7.5 hours long. How long do you think the day should be [5, 6, 7, or 8 hours’? Why? Please write any additional feedback or comments about the MB-BP course below”).

#### Treatment fidelity methods

Treatment fidelity strategies were performed in accordance with recommendations of the NIH Behavior Change consortium, specifically ensuring treatment fidelity in the following five areas: study design, provider training, treatment delivery, receipt of treatment, and enactment of treatment skills.[27] Specifically, the study provided the same treatment dose for each participant enrolled in the MB-BP intervention, including fixed length and number of contact sessions for all MB-BP sessions. Class sessions were audio recorded. Trained research assistants reviewed 10% of classes via randomly selected audio recordings. Competency ratings on these recordings were calculated as the percent concordance to the MB-BP Curriculum Guide modules. We ensured equivalent dose amongst participants including meditation, yoga and stress reduction training, by allocating time recommendations in the MB-BP Curriculum Guide for each module, and tracking the audio recordings. MB-BP instruction was performed by a qualified MBSR instructor (with 2 years experience as an MBSR instructor; 19 years mindfulness meditation experience), having PhD-level training in cardiovascular disease prevention, treatment and etiology. MBSR teacher qualification is fairly extensive, detailed elsewhere.[28] A MB-BP Curriculum Guide was created, and followed by the instructor. MB-BP instructor certification methods were developed through this intervention, and are now available (contact lead author for more information). As this was the intervention development phase, the lead author (E.L.) provided the MB-BP instruction. In order to reduce bias of the lead author, he did not have access to the dataset and was not present at FGDs or IDIs. The data analyst (Y.L.) performed all quantitative statistical analyses, and three co-authors (W.N., A.W., J.W.) coded and analyzed qualitative data independent of the lead author. Participants’ perceptions of instructor’s warmth and credibility were assessed using brief measures based on the validated Therapist Empathy Scale at Weeks 4 and 8 of the intervention. Receipt of treatment, and enactment of treatment skills, were assessed by class attendance, booster session attendance, participants’ mindfulness practice diaries, self-report mindfulness levels (via Five Facet Mindfulness Questionnaire), and frequency by which participants engaged in evidence-based behaviors that can reduce blood pressure including AHA-recommended levels of physical activity, salt intake, DASH diet adherence, alcohol consumption, antihypertensive medication adherence, and stress management.[29–31] Analyses also evaluated the proportion of participants who set goals to improve a specific determinant of hypertension (e.g. diet, physical activity, excessive alcohol consumption, stress reactivity) during classes 4–6 when goals were set and recorded.

#### Primary outcomes: Self-regulation

The three primary self-regulation outcomes at 12 months follow-up were registered *a priori* on ClinicalTrials.gov (Identifier #NCT02702258) as follows:

Multidimensional Assessment of Interoceptive Awareness (MAIA): The MAIA is a 32 item self-report measure with response options ranging on a 6-point Likert scale (0/never-5/Always). Psychometric tests indicate the scale maintains moderate internal consistency (α = >0.70), good model (Comparative Fit Index = 0.886).[32] The MAIA is composed of eight individual scales, specifically *Noticing* (awareness of uncomfortable, comfortable, and neutral body sensations); *Not Distracting* (tendency not to ignore or distract oneself from sensations of pain or discomfort); *Not-Worrying* (tendency not to worry or experience emotional distress with sensations of pain or discomfort); *Attention Regulation* (ability to sustain and control attention to body sensations); *Emotional Awareness* (awareness of the connection between body sensations and emotional states); *Self-Regulation* (ability to regulate distress by attention to body sensations); *Body Listening* (active listening to the body for insight); and *Trusting* (experience of one’s body as safe and trustworthy).[32, 33] In order to reduce issues of multiple statistical testing, the primary outcome was an overall MAIA summary scale consisting of the mean of all items (including reverse coding when indicated). Secondary analyses evaluated impacts of MB-BP on each individual MAIA scale.

Sustained Attention to Response Task (SART): The SART is a computerized go/no-go task that evaluates sustained attention, response inhibition as well as self-regulation.[34] The test requires participants to withhold a behavioral response (i.e. pressing the spacebar) to a single infrequent target presented on a computer screen, in this case the number ‘3’, while also engaging in behavioral responses for frequent non-targets (number 0–9).[34] The SART demonstrated moderate test/re-test reliability in preliminary validation studies (α = 0.76).[34–36] In addition, performance on the test was predictive of attention deficits in a general clinical sample and demonstrated the ability to predict between brain injured patients and standard age matched controls.[34]

Difficulties in Emotion Regulation Scale (DERS): The Difficulties in Emotional Regulation Scale (DERS) is a 36-item measure evaluating an individual’s capacity to regulate their emotional state.[37] Questionnaire responses are rated on a 5-point Likert scale (1 = almost never, 5 = almost always) and higher scores indicate a decreased ability to emotionally regulate.[37] The DERS has high internal consistency (α = 0.93) and good test/re-test reliability in validity studies.[37]

#### Secondary outcomes

Health Behaviors and Perceived Stress: *Physical activity* was assessed utilizing the Rapid Assessment Physical Activity Scale because it is has validity/reliability assessments, and is responsive to behavioral changes including the types (e.g. yoga) introduced in MB-BP.[38, 39] *Dietary Approaches to Stop Hypertension-consisted diet* was assessed using the Harvard 61-food item “80out” Food Frequency Questionnaire,[40] and coding of DASH diet adherence using methods developed by Folsom et al.[41] *Body mass index* was directly assessed using a calibrated weighing scale (SECA, Model 22089, Hamburg, Germany) and stadiometer (SECA, Hamburg, Germany). *Perceived stress* was assessed utilizing the 10-item Perceived Stress Scale (PSS-10) with established validity and reliability.[42, 43] *Alcohol consumption* was assessed via a modified Centers for Disease Control and Prevention Behavioral Factor Surveillance System Questionnaire which has demonstrated concurrent validity with other nationally representative survey measures (i.e. NHIS, NHANES) in multiple studies evaluating alcohol consumption as well as binge drinking.[44, 45] *Day-to-day adherence* to a representative blood pressure medication (i.e., implementation) was assessed using eCAPS^™^ (Information Mediary Corp.; Ottawa, Canada), an electronic adherence monitoring device.[46] If there were multiple antihypertensive medications taken, the most frequently taken medication was prioritized for eCAP assessments. The eCAPs contain chips in the pill caps that record the date and time when the pill bottles are opened. Medication adherence was calculated as the percent of days that participants had the correct number of eCAP openings, as prescribed for their specific antihypertensive medication. Prescribed antihypertensive medication type, dose, and frequency were assessed directly via review of participants’ medication prescription bottles brought to each assessment, and recorded by trained research technicians. Participants were provided with an eCAP at their baseline assessment and were asked to use the eCAP for the duration of their study involvement. Baseline medication adherence was assessed for all days available leading up to the orientation class. Medication adherence for the follow up periods was assessed as follows: (a) Medication adherence at the 3 month follow-up timepoint was calculated for the first 6 weeks after the last day of the intervention. If there were missing data, it was based on 6 weeks of data after the orientation class but no later than 4.5 months follow-up time. (b) Medication adherence at the 6 month follow-up timepoint was assessed based on 3 weeks on either side of the 6 month timepoint after the orientation class; 6 weeks on either side if there were missing data. (c) Medication adherence at 1 year follow-up was assessed based on 6 weeks prior to the 1 year time point; using up to 12 weeks of data prior to the 1 year time if there were missing data, always prioritizing using the data closest to the 1 year assessment time.

Blood Pressure: Clinic blood pressure was evaluated according to American Heart Association guidelines.[47] Blood pressure was assessed using a calibrated Omron HEM-705CPN automated BP monitor (Lake Forest, IL) with established validity.[48] Participants rested, seated for 5 minutes with arm at heart level and legs uncrossed prior to blood pressure assessment. Participants were instructed to refrain from caffeine consumption and physical activity at least 30 minutes prior to blood pressure assessment. Blood pressure was assessed during the initial “screening assessment”, and then again at least one week later for the “baseline assessment.” Only the baseline assessment was used in order to remove potential upward biases in blood pressure because of stress-induced novelty resulting from attending the research clinic for the first time. At each assessment, three blood pressure readings were obtained with 60 seconds duration between assessments. The mean of the second and third blood pressure readings was used for analyses.

#### Adverse events

Adverse events (AEs) were defined as “any untoward medical occurrence in a subject during participation in the clinical study. An adverse finding could include a sign, symptom, abnormal assessment (laboratory test value, vital signs, electrocardiogram finding, etc.), or any combination of these regardless of relationship to participation in the study.”[49] Furthermore, serious adverse events (SAE) were defined as “any untoward medical occurrence that resulted in death, was life threatening, required inpatient hospitalization or prolongation of existing hospitalization, resulted in persistent or significant disability/incapacity, or was a congenital anomaly.”

In the stage 1 trial, AEs and SAEs were monitored throughout the study duration using four primary approaches. First, participants were monitored for potential psychological distress (i.e. anxiety, depression and suicidal ideation) using the Beck Anxiety Inventory (BAI) and the Center for Epidemiology Study Depression Scale Revised (CESD-R) self-report questionnaires at each in-person research assessment. Participants whose BAI and/or CESD-R scores fell outside of the predetermined ‘acceptable’ range would trigger the study safety protocol to be implemented, which would involve, at a minimum, a trained research staff following up with the participant regarding his/her current mental health and passing along the details of the case to the study clinician for review and potential follow up, and, at a maximum, would require immediate intervention in the form of calling 911 if a participant indicated he/she was at risk for harming himself/herself. Second, monthly safety monitoring emails were sent to all participants inquiring about physical injuries. Participants who had sustained any type of physical injury in the subsequent four weeks were asked to complete a brief online form providing additional detail on the injuries sustained. AE data on physical injuries were compiled and reported to the Data Safety Monitoring Board (DSMB) annually. All SAEs were reported immediately to the study PI and DSMB chair. Third, participants were monitored for out-of-range laboratory values (i.e. blood pressure readings). Specifically, safety parameters were programmed into the in-person assessments such that any blood pressure readings outside of a predetermined ‘safe range’ (i.e., systolic blood pressure > 200 mmHg or < 90 mmHg, or diastolic blood pressure >110 mmHg) triggered the study safety protocol. Data on out-of-range laboratory values were compiled and reported to the Data Safety Monitoring Board on an annual basis. The fourth approach to safety monitoring involved passive monitoring by the study interventionist and trained research staff. Under this approach, study personnel were trained to document and report all AEs and SAEs observed or reported during the course of routine interaction with study participants (e.g., during the study intervention, in-person assessments, or study communications).

The study coordinator was responsible for documentation and reporting all AEs and SAEs. Annual Data Safety Monitoring Reports were compiled and presented to the DSMB as well as to the funding agency. The study coordinator used the AE attribution scale to determine relatedness to the intervention for all reported events. All AEs were categorized according to likelihood that they were related to the study intervention using the labels: “definitely unrelated,” “definitely related,” “probably related,” or “possibly related” to the study intervention. Similarly, all events were graded by severity (mild, moderate, or severe) depending on the intensity of the event for the study participant. An AE was termed “mild” if it did not have a major impact on the participant, “moderate” if it caused the participant some minor inconvenience, and “severe” if it caused a substantial disruption to the participant’s well-being.

#### Effect modifiers

Given prior research described above suggesting potential for floor effects, *a priori* determined analyses evaluated effect modification by participants with ≥140 mmHg systolic (i.e. stage 2 systolic hypertension) *vs*. those with SBP 120–139 mmHg (i.e. elevated blood pressure or stage 1 hypertension according to the 2017 American College of Cardiology/American Heart Association [ACC/AHA] Guidelines).[3] Participants taking and not taking antihypertensive medication were included in the study. Consequently, for those taking antihypertensive medication, participants in the SBP 120–139 mmHg range can include varying levels of controlled hypertension.

A recent systematic review compiled early evidence that four of seven clinical trial studies demonstrated home mindfulness practice was associated with improvements in clinical outcomes.[50] Given this was a single arm trial, stratifying by home practice also provides some benefit for examining dose-response effects by home practice amount (recognizing that home practice amount was not randomly assigned). *Post-hoc* analyses explored if SBP effects were different by tertile of formal home practice amount. Home practice was assessed weekly during the intervention using questionnaires that probed participants’ formal mindfulness practice amount in all home practice modules assigned, including standing yoga, lying-down yoga, body scan, awareness of breath meditation, and sitting meditation (including focused attention and open monitoring). The total formal practice time during the MB-BP course was summed, and tertiles were calculated.

Further *post-hoc* analyses evaluated impacts of MB-BP intervention on participants not adhering to AHA guidelines, defined as follows: BMI≥25kg/m^2^; DASH Diet Score <5.5; sodium intake >1500 mg/d; physical activity <30 minutes per day of moderate physical activities at least 5 per week or 20 minutes per day of vigorous physical activities at least 3 days per week; and alcohol consumption ≤2 drinks per day for males and ≤1 drink per day for females. Given evidence from national surveys that mean PSS-10 score in US adults is approximately 16, analyses also evaluated stress reductions in participants with PSS-10 score>16.[51] These analyses were done as many participants had acceptable levels of blood pressure determinants at baseline, and were encouraged to focus on modifying determinants of blood pressure that were of most relevance to them personally.

#### Statistical methods

Power calculations were performed utilizing G*Power Version 3.1.9.2 (Henrick-Heine-University of Düsseldorf, Germany) as follows. As this was the first time the MB-BP intervention was evaluated, we chose a medium effect size classically defined as Cohen’s d = 0.05.[52, 53] With Cohen’s d medium effect size of 0.5, power of 0.80, alpha of 0.0167, and one-tailed paired T-test, power analyses suggested that a sample size of 38 participants would be adequate. With Cohen’s d medium effect size of 0.5, power of 0.80, alpha of 0.0167, and one-tailed paired T-test, power analyses suggested that a sample size of 38 participants would be adequate. One-tailed (rather than two-tailed) was selected due to hypothesis that MB-BP would improve primary outcomes. Alpha of 0.0167 was selected based on Bonferroni correction that account for statistical tests with three primary outcomes. The study’s sample size of 44 suggests adequate statistical power was available.

Analyses were performed as intention to treat (ITT). The analytic approach included descriptive statistics (mean, SD) of the primary and secondary outcomes at baseline, three months, six months, and one-year follow-up. Due to the wide range of home mindfulness practice amounts, median and interquartile range were reported for this variable. To account for the repeated measures design, hierarchical linear models with random intercepts were applied for all repeatedly measured primary and secondary outcomes, using maximum likelihood estimation with observed information matrix for statistical variance. These models estimated the change in outcome levels from baseline through one-year follow-up. With five participants lost to follow-up at 12 months (10% loss to follow-up), we performed sensitivity analyses to evaluate robustness of findings for primary outcomes. Sensitivity analyses imputed null effects, as well as 5% changes in outcomes in the *opposite* direction from those actually observed in analyses, for the five participants, and reported p-values for above analyses. All statistical analyses were performed using Stata statistical software, version 14.0 (StataCorp LLC, College Station, TX). The data analyst (Y.L.) performed all statistical analyses.

Qualitative data was analyzed using NVivo v.11. Structured codes were developed from study objectives (e.g. acceptability of intervention components) and the mindfulness and cardiovascular health theoretical framework.[54, 55] Both FGD and IDI transcripts were double coded by two members of the research staff (W.N. J.W.). Staff used directed content analysis, a structured, deductive process appropriate in instances of existing theoretical frameworks and predefined study hypotheses.[54, 56] Cross-checks for consistency in coding were performed by a third member (A.W.) of the research team and *a priori* and emergent themes were reviewed by the PI and qualitative expert (A.H.) for consistency.

## Results

### Feasibility and acceptability

**Fig 2:**
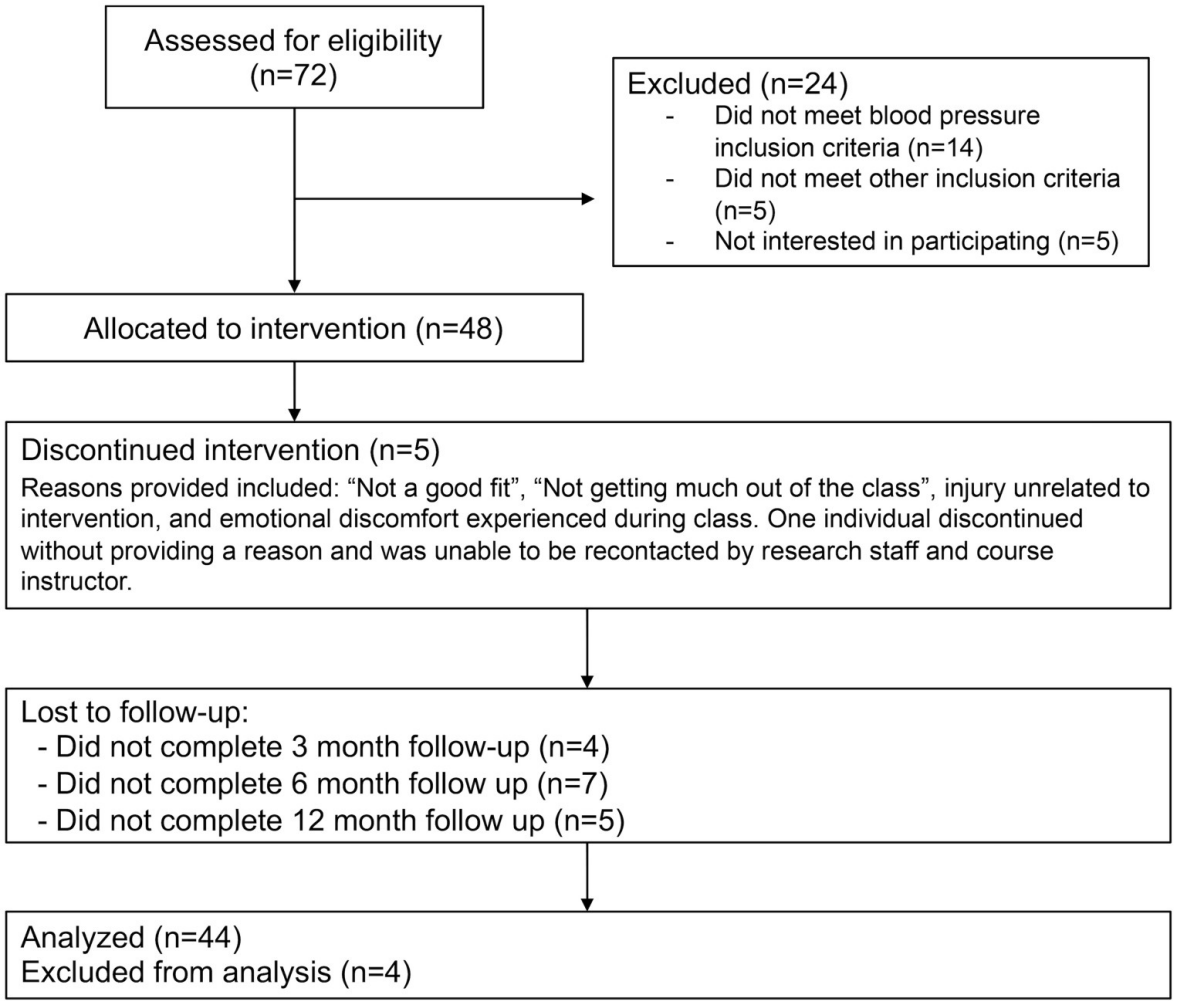
CONSORT flow diagram for the MB-BP study participation.

After assessing 72 participants for eligibility and excluding 14 for not meeting inclusion criteria and 5 not interested in participating, there were 53 eligible participants (Fig 2). Feasibility and acceptability findings showed that of the 53 eligible participants, 48 enrolled in the study (91%). Of these participants, 43 (90%) attended at least 7 of the 10 MB-BP classes (including orientation session and all-day retreat). Participation rates for the 48 participants in the orientation session, along with week 1, 2, 3, 4, 5, 6, 7, 8, and the all-day retreat were 98%, 98%, 92%, 77%, 81%, 88%, 81%, 85%, 83% and 79%, respectively. Forty-three participants were followed through to one year assessment (90% retention rate). Demographics show the study population (n = 48) included 61% females, and was predominantly white race/ethnicity (96%; Table 1). Participants were highly educated on average (92% with college degree), with a mean age of 60 (range 26–83) years. Approximately 60% (29 of 48) of participants were taking antihypertensive medication at baseline. Baseline levels of body mass index, diet, physical activity, alcohol consumption, stress and antihypertensive medication use are shown in Table 2.

**Table 1:** Demographic characteristics of sample at baseline (n = 48). Please refer to Table 2 for determinants of blood pressure levels at baseline.

Variable	Point Estimate
Age, y	60.0
Race, % White	95.9
Gender, % women	61.2
Education, % college education	91.8
Hypertension Status	
Uncontrolled stage 2 hypertension, %	47.9
Stage 1 hypertension, %	33.3
Elevated blood pressure, %	18.8
Taking antihypertensive medication, %	60.4

Stage 2 hypertension: SBP≥140 or DBP≥90 mmHg; Stage 1 hypertension: 130≤SBP<140 or 80≤DBP<90 mmHg; Elevated blood pressure: 120≤SBP<130 mmHg and DBP<80 mmHg. DBP, diastolic blood pressure; SBP, systolic blood pressure. Note that "uncontrolled" stage 2 hypertension reflects participants with SBP≥140 or DBP≥90 mmHg at baseline. Some participants in stage 1 hypertension or elevated blood pressure categories may have been in Stage 2 hypertension in the past, but may be currently controlled by antihypertensive medication or nonpharmacologic interventions.

**Table 2:** Secondary outcomes, representing evidence-based determinants of blood pressure that are intermediate MB-BP intervention targets.

		Baseline			3 months				6 months				12 months			
		n	Point Estimate	95% CI	n	Point Estimate	95%^ CI	p	n	Point Estimate	95% CI	p	n	Point Estimate	95% CI	p
**Overweight/Obesity**																
Body Mass Index	All participants, mean	48	28.5	27.2, 29.7	44	28.2	26.9, 29.5	**0.05**	41	28.3	27.0, 29.6	0.33	43	28.6	27.3, 29.9	0.377
	Participants with BMI ≥25 kg/m^2^ at baseline, mean	40	29.5	28.2, 30.8	37	29.2	27.9, 30.5	**0.04**	34	29.4	28.1, 30.7	0.43	36	29.7	28.4, 31.0	0.285
**Diet**																
DASH Diet Score	All participants, mean	48	3.27	3.02, 3.52	44	3.49	3.24, 3.75	0.15	41	3.42	3.15, 3.68	0.35	43	3.41	3.15, 3.67	0.370
	Participants with DASH diet score <5.5 at baseline, mean	32	2.78	2.51, 3.06	29	3.38	3.10, 3.67	**<0.001**	26	3.37	3.08, 3.67	**<0.001**	28	3.34	3.05, 3.63	**<0.001**
Sodium Intake, Added at Table (shakes per day)	All participants, mean	48	0.69	0.36, 1.01	43	0.38	0.06, 0.72	**0**	43	0.41	0.08, 0.74	**0.01**	42	0.46	0.13, 0.79	**0.022**
	Participants consuming >1500 mg/d at baseline, mean	10	1.63	0.61, 2.65	8	0.43	-0.63,1.50	**0**	8	0.54	-0.52,1.61	**0**	8	0.55	-0.52,1.61	**0.003**
**Physical Activity**																
Aerobic Physical Activity	All participants, % adhering to AHA physical activity guidelines*	48	69%	54%, 80%	44	66%	51%, 78%	0.77	43	65%	50%, 78%	0.71	43	70%	55%, 82%	0.916
	Participants not adhering to AHA physical activity guidelines at baseline,* % adherence	15	0%	NA	14	29%	11%, 57%	**0.03**	13	38%	17%, 66%	**0.01**	14	29%	11%, 57%	**0.026**
Flexibility Activities	All participants, % engaging in flexibility activities	48	42%	29%, 56%	44	73%	58%, 84%	**0**	43	63%	48%, 76%	**0.04**	43	60%	45%, 74%	0.073
	Participants not engaging in flexibility activities at baseline, % adherence	28	0%	NA	28	57%	38%, 74%	**<0.001**	26	42%	25%, 62%	**<0.001**	27	41%	24%, 60%	**<0.001**
Muscle Strengthening Activities	All participants, % engaging in muscle strengthening	48	38%	25%, 52%	44	41%	27%, 56%	0.74	43	44%	30%, 59%	0.52	43	37%	24%, 52%	0.980
	Participants not engaging in muscle strengthening at baseline, % adherence	31	0%	NA	29	21%	10%, 39%	**0.01**	27	26%	13%, 46%	**0**	28	14%	5%, 33%	**0.030**
**Alcohol Consumption**																
Daily Alcohol Consumption	All participants, mean number of drinks per day	47	0.64	0.41, 0.86	43	0.53	0.30, 0.76	0.06	42	0.51	0.27, 0.74	**0.02**	43	0.56	0.33, 0.80	0.200
	Mean number of drinks per day in participants not adhering to AHA guidelines at baseline**	7	2.61	1.95, 3.27	6	1.75	1.05, 2.45	**0**	5	1.41	0.68, 2.14	**<0.001**	5	1.72	1.02, 2.42	**0.001**
Binge Drinking	All participants, mean binge drinking days in past 30 days	46	0.8	0.2, 1.4	29	0.3	-0.4, 0.9	0.08	29	0.6	-0.1, 1.3	0.55	34	0.3	-0.4, 0.9	0.060
	Mean binge drinking days in past 30 days, only in participants binge drinking at baseline	8	4.5	2.0, 7.0	6	1.7	-1.1, 4.5	**0.04**	5	3.3	0.3, 6.2	0.37	6	1.5	-1.2, 4.3	**0.030**
**Stress**																
Perceived Stress Scale	All participants, mean	48	22.7	20.7, 24.8	42	20.1	18.2, 22.4	**0**	43	20.0	17.9, 22.1	**0**	42	20.7	18.6, 22.8	**0.012**
	Participants with PSS score greater than US mean of 16, mean	40	24.3	22.6, 25.9	25	23.9	22.0, 25.8	0.67	28	22.8	21.0, 24.6	0.09	28	23.3	21.5, 25.1	0.280
**Antihypertensive Medication Adherence**																
Directly assessed from participants’ medication bottles	Increased antihypertensive medication dose and/or added additional antihypertensive medication type, %	NA	NA	NA	2	7.7	1.8, 27.5	0.15	3	12.5	3.8, 33.9	0.07	3	11.5	3.6, 31.6	0.074
	Decreased antihypertensive medication dose and/or removed antihypertensive medication type, %	NA	NA	NA	2	7.7	1.8, 27.5	0.15	4	16.7	6.1, 38.3	**0.04**	3	11.5	3.6, 31.6	0.074
	Changed antihypertensive medication type, %	NA	NA	NA	3	11.5	3.6, 31.6	0.07	3	12.5	3.8, 33.9	0.07	5	19.2	7.9, 39.9	**0.019**
Directly assessed via eCAPs	Percent adherence to antihypertensive medication assessed via eCAPS, %	16	0.95	0.90, 0.99	16	0.87	0.82, 0.93	0.004	16	0.91	0.88, 0.94	0.15	16	0.91	0.88, 0.94	0.190

*AHA guidelines consistent with RAPA are engaging in 30 minutes or more per day of moderate physical activities 5 or more days per week, or 20 minutes or more per day of vigorous physical activities 3 or more days per week

**AHA guidelines for alcohol consumption are < = 2 drinks per day for males and < = 1 drink per day for females

Analyses were performed using hierarchical linear models. P values represent comparison of respective follow-up time to baseline

Qualitative results demonstrated that participants reported a high degree of acceptability for the program, and viewed the intervention delivery modality, training modules, and support systems as appropriate. Several themes emerged, as shown below. Qualitative findings were similar between FGDs and IDIs.

#### Theme one—Instructor was an effective teacher and model for the practices

The feedback regarding the instructor was singularly positive regarding both the delivery of the intervention, and modeling the practice, for participants. Participants explained that the instructor was able to convey the information effectively, “*way of conveying both information and activities in a way that’s engaging and appropriate*”(P 246), with a comprehensive knowledge of the practice *“he sort of walks and lives his talk”* (P 245), as well as an ability to engage with them appropriately “*I always felt*, *no matter what you said or how you said it*, *he listened*, *he’d process it”* (P 144) and that this supported the learning “*deepens the learning in a non-judgmental supportive fashion”* (P 245). One of the most critical attributes was the instructor’s ability to offer relatable personal examples “*When he brought up things to illustrate points*, *he would bring in personal anecdotes”* (P 331) which supported their understanding of the modules and impacted their willingness to implement intervention training “*That was a big influence on me and how I can follow this practice it will lower my blood pressure”* (P 021). Many expressed that it would have been helpful to have more one-on-one time to review and troubleshoot their continued practice either in-person “*half an hour [others interrupt to say “that’s a good idea”] where we sit down with him*” (P 830) or submit additional materials in writing for his review “*here is what I am planning to do over the next two months*, *is it reasonable*?*”*.

#### Theme two—Intervention digital materials were useful for home practice but the monitoring was burdensome

Other intervention elements that were viewed favorably, and recognized as critical to effective participation, included access to audio recordings on USB flash drives, compact discs, and online downloads/streaming through the web portal. These resources supported home practice “*One of the greatest things was learning something in class*, *and then being able to take the CD home”* (P 879) however some believed that the website, although useful, *“may need a little work*, *I don’t know*, *to be more user-friendly”* with one unaware they even had web access “*is that on the graduate website*?*”* (P 497). There was overwhelming agreement that the materials used to document the home practice were highly burdensome *“We were given homework that was serious homework*, *it was a lot of responsibility”* (P 160), *“There’s a lot of written homework”* (P 895) which made practice while balancing other aspects of their life difficult *“There*, *there’s a lot*, *a lot*. *And I have two friends in the program that are working*. *And one of them was like*, *'Pshhh*, *this is ridiculous”* (P 276).

#### Theme three: Study personnel were helpful and accessible

Study staff were viewed as readily available and helpful “*I found that having a phone number*, *or if you did email or whatever to get in touch with them*, *they always got back you”* (P 276) specifically for troubleshooting technical difficulties “*couldn’t do something on the computer*, *I couldn’t find something*, *or I couldn’t… the Fitbit wasn’t working right*, *or… I mean*, *I probably called [the project coordinator] about five different times and she always got right back”* (P 276) or questions regarding mindfulness practice and intervention components “*he always emails back and calls back*, *he’s very good”* (P 852). Participants consistently reported that they felt accommodated and the study was effectively organized and implemented by staff *“I really appreciated how organized it was*, *that it started on time*, *that people*, *the parking… I thought we were well-treated with the parking and really good food*, *and our time was respected*” (P 160). However, despite generally positive feedback regarding implementation there were still logistic challenges to the in-person delivery. Participants recommended improvements included minimizing impediments around building access, that the study location was difficult to get to driving (e.g. inner city location) and that the room temperature and lighting made engaging in mindfulness practice during class more difficult.

#### Theme four—Group delivery of the intervention allowed an opportunity for social support fostering a deeper understanding and application of practices

One of the important aspects of the program was the interpersonal/social support available in the group. Participants reported that being in a group engendered a sense of community “*the sense of community was very very helpful to me*” (P 331) and was recognized “*a huge factor”* in the successful understanding of the practices “*they came up with great ideas and different ideas and different things of interest”* (P 347) and application of intervention components. More specifically the focus group participant expressed that being able to experience “*commonality*, *and also the commitment”* regarding “*dealing with the same issue of blood pressure”* was *“very powerful to me”*. Others indicated that hearing the shared lived experiences they were exposed to enhanced their ability to learn and engage with the practices, “*So one-on-one I don’t know that learning would have been nearly as effective as doing it in a group”* (P 246) and *“I think I just seem to have more energy meditating with other people*. *I don’t know why I just feel calmer more relaxed”* (P 683). However, although participants generally agreed that the larger group was a benefit to practice there was some contention regarding the group dyads with some expressing that it provided an opportunity for *“great ideas and different ideas and different things of interest”* believing that *“The smaller groups were superb”* (P 347) while others found the breakout groups too intimate and felt they were “*forced to communicate with people that I don’t know*” (P 507) which was uncomfortable.

*Participant Ratings on Usefulness of MB-BP Customizations*: Card sort results are shown in S3 Table. Overall participants rated the “engaging mindfully in an aerobic activity other than yoga” as the most useful intervention customization, with 69% of participants rating this as “very useful” (mean score = 1.62, on a scale where not useful = 0, useful = 1, very useful = 2). The second highest rated activity was a “motivational interviewing-based goal setting worksheet on improving a determinant of blood pressure during the coming week”, followed by “group discussion” (69% “very useful”, mean score = 1.56). In addition, “engaging in breakout group discussions around experiences implementing the blood pressure determinant goal” was also rated highly (63% “very useful”, total score = 1.50) consistent with qualitative feedback (Theme 4). Also consistent with participant qualitative feedback, documenting the “unpleasant events calendar for diet and alcohol consumption” (50% “not useful, mean score = 0.69) and the “pleasant events calendar for diet and alcohol consumption” (31% “not useful”, mean score = 0.94) were viewed as the least useful intervention customizations.

*Open Ended Survey on Preferred Length of Class Session/All Day Retreat*: Participants indicated that 2.5 hours was an effective amount of time for class (S4 Table) to cover intervention materials with an average preferred time of 2.4 (n = 26). The average preferred retreat length was 7.0 hours (n = 23), reduced from 7.5. The majority of participants expressed that they would prefer 7.5–8 hours (n = 14) however, of the nine participants who preferred less time, two cited cognitive fatigue, one suggested it was too difficult to stay silent that long, one stated six hours but believed an hour break away from everyone would be best resulting in seven hours, and one seemed confused by the question stating that five hours “was good” and they “wouldn’t decrease the time”.

### Engagement of MB-BP with primary self-regulation outcomes

All three primary self-regulation outcomes were significantly improved from baseline to one-year follow-up, including attention control (SART % correct no-go: 69.5% to 79.0%, respectively; p<0.001), self-awareness (MAIA mean score: 22.6 to 26.4, respectively; p<0.001), and emotion regulation (DERS mean score: 70.4 to 65.1, respectively; p = 0.02; Table 3). Objective task performance on the SART was measured by several metrics reflecting accuracy and perceptual sensitivity in response to target no-go trials (“hits”) and errors in response to go-trial non-targets (“false alarms”). Errors of commission (failure to withhold a response to a no-go target trial) reflect a pronounced state of task disengagement, including failures in both sustained attention and inhibitory control.[57] Percent correct rejections of no-go target stimuli across trials was identified as the primary outcome based on an emphasis on self-regulatory metrics for successful response inhibition. Other performance indices were demonstrated significant (p<0.05) improvements, such as correct go, number of omission error, number of commission error, and perceptual discrimination indices (A’ and D’) between target and non-target stimuli (S5 Table). Findings for the individual MAIA scales are provided in S6 Table. Six of the eight subscales demonstrated significant improvements at one-year follow-up vs. baseline: noticing: 3.51 vs. 3.11, respectively; p = 0.009; not worrying: 3.86 vs 3.47, respectively; p = 0.006, attention regulation: 2.97 vs 2.36, respectively; p<0.001, self-regulation: 3.47 vs 2.47, respectively; p<0.001, body listening: 2.74 vs 1.98, respectively; p<0.001, and trusting: 2.96 vs 3.66, respectively; p<0.001. Two subscales did not reach statistical significance at the p<0.05 level at one-year follow-up vs. baseline: non-distracting: 3.37 vs 3.03, respectively; p = 0.09, emotional awareness: 3.45 vs 3.12, respectively; p = 0.16.

**Table 3:** Primary outcomes at baseline through 1-year follow-up, representing proximal MB-BP intervention targets.

	Baseline			3 months				6 months				1 year			
	n	Point Estimate	95 CI	n	Point Estimate	95 CI	p	n	Point Estimate	95% CI	p	n	Point Estimate	95% CI	p
Difficulties in Emotion Regulation Scale, mean score	48	70.4	65.0, 75.8	41	67.7	62.1, 73.3	0.23	43	65.7	60.1, 71.3	**0.04**	41	65.1	59.5, 70.8	**0.02**
Multidimensional Assessment of Interoceptive Awareness, mean score	35	22.6	20.8, 24.4	37	26.3	24.6, 28.1	**<0.001**	38	26.3	24.5, 28.0	**<0.001**	35	26.4	24.6, 28.1	**<0.001**
Sustained Attention to Response Task, % correct no-go	42	69.5	65.1, 73.9	43	75.9	71.6, 80.2	**0.002**	36	76.4	71.9, 81.0	**<0.001**	41	79.0	74.6, 83.4	**<0.001**

Analyses were performed using hierarchical linear models. P values represent comparison of respective follow-up time to baseline.

With five participants lost to follow-up at 12 months (10% loss to follow-up), we performed sensitivity analyses to evaluate robustness of findings for primary outcomes. Sensitivity analyses evaluated if the five participants were to have had null effects where there was no change in the MAIA, DERS, SART from baseline to 12 months follow-up, that p-values for the models (n = 53) were <0.001, 0.03, and <0.001, respectively. Further analyses showed that if there were 5% changes in the outcomes for the five missing participants in the *opposite* direction from those actually observed in analyses (i.e. increase in DERS, and reduction in MAIA and SART scores), that p-values were as follows for the MAIA (p<0.001), DERS (p = 0.04), and SART (p<0.001).

### Engagement of MB-BP with modifiable determinants of blood pressure

While physical activity, DASH diet score, alcohol consumption, and salt intake did not significantly change for the entire population, it significantly improved in those who were not adhering to AHA guidelines for physical activity, salt, and alcohol at baseline, or who had low DASH diet scores at baseline, as shown in Table 2 [3, 41, 58]. For physical activity, the data showed significant improvements in aerobic, flexibility and strengthening exercises via the RAPA questionnaire for those with low physical activity levels at baseline. An exception to this overall pattern of improvements in those with poor baseline levels of hypertension risk factors was that stress levels were significantly reduced in all participants, but not significantly improved in those with PSS scores higher than a representative sample of the general US adult population that showed mean PSS-10 level of 16 (Table 2).[51] This suggests that those with higher levels of stress may not have as great a stress reduction as those with lower levels of stress. BMI showed marginal significant improvements at 3 months (p = 0.04), but effects did not hold through one-year follow-up (Table 2). Antihypertension medication use changed in 42% of participants over one-year follow-up, with equal proportions increasing and decreasing their medication dosage by one-year follow-up (Table 2).

### Engagement of MB-BP with blood pressure

**Fig 3:**
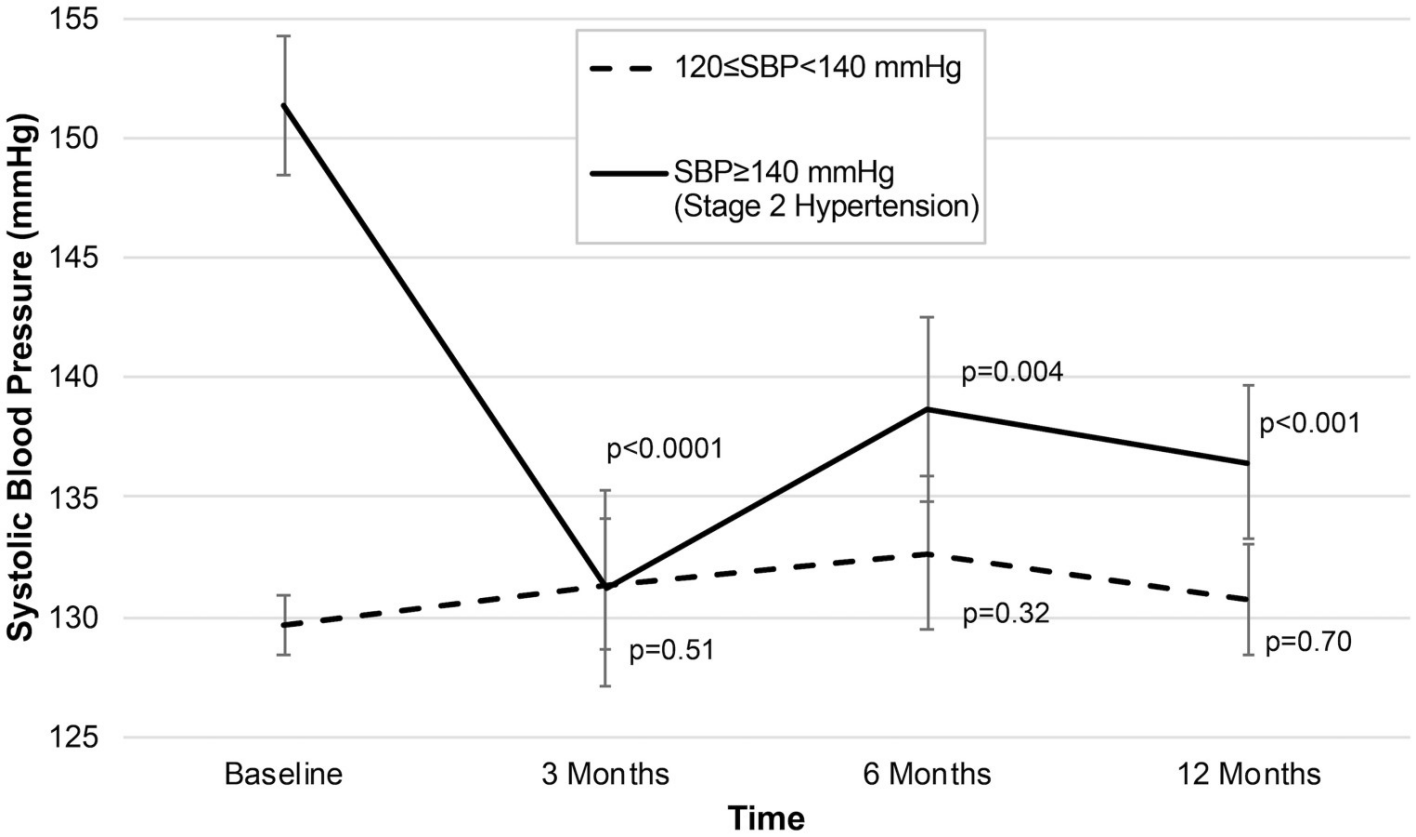
Changes in systolic blood pressure (SBP) from baseline through follow-up after MB-BP intervention. Analyses were performed using hierarchical linear models. P-values represent comparison of respective follow-up time to baseline. Error bars are standard errors of the mean. Error bars represent standard error of the mean. P-values compare follow-up times to baseline. Sample sizes for comparisons at 3, 6 and 12 months vs. baseline are 20, 18, and 19, respectively, for participants with SBP≥140, and 24, 23, and 24, respectively, for participants with SBP≥120 and <140 mmHg.

**Fig 4:**
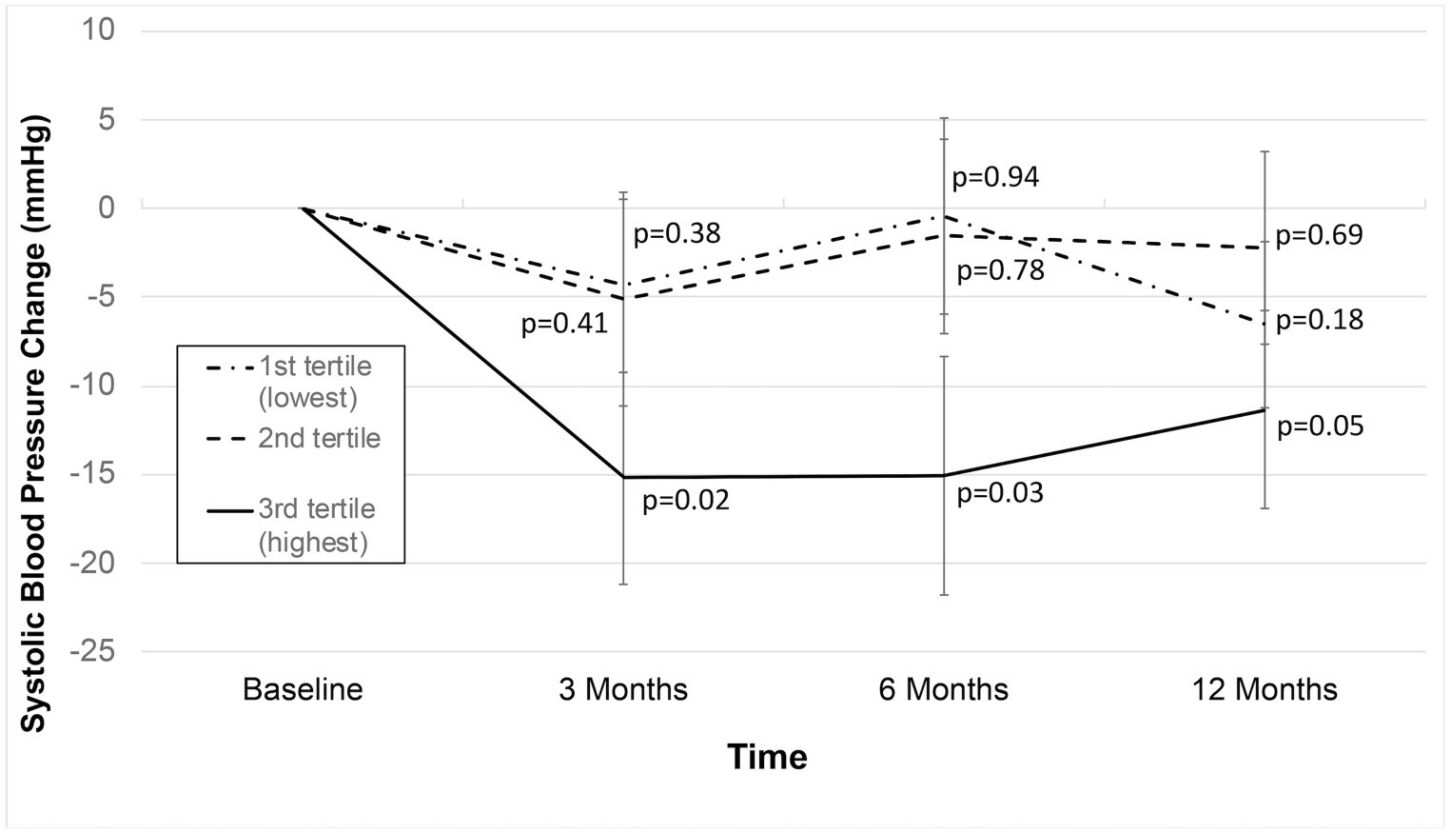
Mean systolic blood pressure (SPB) over one-year follow-up, stratified by tertile of formal home mindfulness practice amount during the eight week MB-BP course. Analyses were performed using hierarchical linear models. P-values represent comparison of respective follow-up time to baseline. Error bars are standard errors of the mean. Sample sizes for comparisons at 3, 6 and 12 months vs. baseline are 14, 13 and 11 respectively, for highest tertile of home practice, 15,14, and 14, respectively, for participants with medium tertile of home practice, and 15, 14, and 14, respectively, for participants with lowest tertile of home practice.

Mean systolic blood pressure showed significant reductions of 6.1 mmHg (p = 0.008) at one-year follow-up, lowering from 139.3 at baseline to 133.2 mmHg. Floor effects were pronounced, as *a priori* selected evaluations of effect modification by baseline blood pressure showed large reductions in mean systolic blood pressure in Stage 2 hypertensives (15.1 mmHg reduction from baseline at one year follow-up; n = 19; p = 0.0002), as shown (Fig 3), where SBP went from 151.5 at baseline to 136.4 mmHg at one year follow-up. Blood pressure maintenance was observed in participants with mean systolic blood pressure of at least 120 and below 140 mmHg, showing a 0.56 mmHg reduction from baseline at one-year follow-up (p = 0.42; n = 24), where SBP was 130.54 at baseline, and 129.98 mmHg at one year. *Post-hoc* analyses suggested evidence of effect modification by the amount of formal mindfulness practice participants engaged in outside of class, where those in the highest tertile of formal mindfulness practice (median = 18.0 h per week, interquartile range: 13.5–27.0 h) demonstrated 15.2 mmHg lower SBP at three months follow-up (p = 0.02), which held through one year at 11.4 mmHg reduction from baseline (p = 0.05) (Fig 4). Comparatively, those in the lowest tertile of formal mindfulness practice amount (median = 0.0 h per week; interquartile range = 0.0–2.3 h) demonstrated a 4.4 mmHg reduction in mean SBP at three months follow-up (p = 0.38), which remained not statistically significant through one-year follow-up (p = 0.18) (Fig 4). Diastolic blood pressure findings showed similar floor effects. In the few participants (n = 7) with diastolic blood pressure ≥90 mmHg at baseline (i.e. stage 2 hypertension using diastolic blood pressure cut-point), there was a mean reduction of 7.5 mmHg at 12-months follow-up (p = 0.08). In the larger number of participants with diastolic blood pressure ≥80 mmHg at baseline (i.e. Stage 1 or 2 hypertension using diastolic blood pressure cut-point), mean diastolic blood pressure reduced by 4.0 mmHg at one-year follow-up (n = 24; p = 0.03). For all participants in the study, there was an observed reduction of 1.1 mmHg diastolic blood pressure at 1-year follow-up for all participants (p = 0.22).

### Adverse events

Adverse events were monitored and reported in the annual DSMB reports. No serious adverse events or deaths were reported during the duration of the stage 1 trial. Of the adverse events reported, two were deemed to be related to the study intervention. Specifically, one participant dropped out of the intervention due to feeling emotional discomfort during the course. The one injury confidently attributed to the MB-BP program was reported due to the yoga practice (shoulder injury) that was resolved during follow-up (details in S7 Table).

### Treatment fidelity

Treatment fidelity analyses demonstrated 96.3% adherence to the MB-BP curriculum guide modules by the instructor. Average Therapist Empathy Scale score was 26.4 (scale range: -4 to 30) suggesting participants perceived the instructor had a high degree of empathy. Receipt of treatment analyses showed that 43 (89.6%) of the 48 participants who started the course attended at least 7 of the 9 weekly classes (including the orientation session); 79.2% attended the all-day retreat. Of the 43 participants who did not drop out, all 43 (100%) attended at least 7 of the 9 weekly classes, and 88.4% (n = 38) attended the all-day retreat. Enactment of treatment skills analyses demonstrated a median (interquartile range) duration of weekly formal home mindfulness practice of 6.8 (4.5, 11.3), 3.5 (0.9, 7.0) and 3.3 (1.2, 6.7) hours at 3 months, 6 months and one-year follow-up, respectively. Analyses showed that 39.5% of participants attended at least one of the optional all-day retreats or weekly community group sessions post-graduation from MB-BP. Furthermore, of the 86% of participants that submitted goal-setting forms, 100% set goals in weeks 4–6 related to improving determinants of hypertension such as diet, physical activity, alcohol consumption and stress-reactivity. Self-reported mindfulness, via the FFMQ total score, increased from a mean of 127.5 at baseline to 137.7 (p<0.0001), 136.1 (p<0.0001), and 138.6 (p = 0.0002), at 3 months, 6 months and one year follow-up, respectively. With regard to behavioral enactment of treatment skills, several health behaviors were improved in participants who were not adhering to American Heart Association guidelines or had low DASH diet scores at baseline,[3, 41, 58] including physical activity (p = 0.02), DASH-consistent diet (p<0.001), and alcohol consumption (p<0.001).

## Discussion

The study findings suggested good acceptability and feasibility for the MB-BP intervention, where of the 48 participants, 43 (90%) attended at least 7 of the 10 MB-BP classes, and 43 were followed-up to one year (90%). Focus groups (n = 19) and semi-structured interviews (n = 7) showed all participants viewed the delivery modality favorably, however logistic considerations concerning program access were barriers. Participants identified several intervention attributes as positive, including the intervention’s experiential, didactic approach to mindfulness and hypertension education, as well as course length and number of contact hours.

Findings in this single arm trial suggest that MB-BP may improve several proximal, intermediate and distal pathways to hypertension development, consistent with the proposed theoretical framework (Fig 1). The primary self-regulation outcomes (i.e. emotion regulation (DERS), attention control (SART) and self-awareness (MAIA)), showed significant pre-post improvements. While the DERS has not been widely evaluated in mindfulness interventions to date, preliminary clinical trials suggest that mindfulness interventions may influence the DERS.[59, 60] The SART, as well as other metrics of attention control, has been shown to be responsive in some but not all studies, and the specific SART outcomes vary by study.[61–65] While this outcome offers promise as a direct behavioral measure of attention control, systematic parametric manipulations are currently being tested to determine improved sensitivity and specificity to mindfulness interventions, such as varying stimulus duration, inter-trial intervals and using emotionally laden go and no-go stimuli to explore attentional stability during valenced-emotional events.[66] The MAIA is increasingly studied, and, although still relatively sparse, reports to date consistently suggest that mindfulness interventions engage with the measure.[67, 68]

As described in the theoretical framework (Fig 1), mindfulness training develops attention control through meditative practices on objects such as the breath or body scan. When the mind wanders, as it invariably does, participants are invited to notice where it wandered to, and without any judgment, invite attention back to the object of meditation. Participants are also trained to focus attention on real-life circumstances such as mindful eating and walking. Through this process, it is hypothesized that attention control develops, similar to any muscle we may train in the body, so that the ability to place the mind on things of import during daily life, including determinants of hypertension, such as stress reactions, overconsumption of foods, and the enjoyment of certain physical activities, social relationships, or tasty healthy foods may become more present. Related to this is the training of self-awareness (Fig 1). Specific modules in MBSR and MB-BP train participants to be acutely aware of their physical sensations, emotions and thoughts, as arguably our entire personal experience of existence is in these three domains. By disentangling the experience of physical sensations, thoughts and emotions into separate categories, and deliberately training awareness of them, such as through the body scan which places non-judgmental awareness on each and every part of the body to observe its experience in this moment, along with meditations such as sitting meditations that bring focus in turn to the breath, physical sensations, emotions, thoughts, and open awareness of whatever arises in the domains of sensations, emotions and thoughts, this trains participants to enhance their self-awareness. Applied practices come through, where participants are given palatable unhealthy foods (e.g. chocolate chip cookies, salty potato chips) during class, and invited to non-judgmentally note thoughts, sensations and emotions as they see these items, and eat them (if they choose to). During the 30–35 minutes after, participants are invited to notice with self-awareness sensations natural results such as sugar highs or crashes, or absence of sugar highs and crashes, knowing that this present moment is influenced by prior moments. Similar applied activities are done with physical activities (e.g. walking or jogging during class), social communications, and consuming healthy DASH-diet consistent foods during the all-day retreat, along with invitations to nonjudgmentally notice the short- and longer-term sensations, thoughts and emotions that take place with alcohol consumption or antihypertensive medication use. By enhancing self-awareness in non-judgmental, gentle, curious ways, information may come through on the reality of participants’ experiences, particularly in relation to how they eat, exercise, drink, and interact with stressors, which can lead to important personal insights and behavior change. Emotion regulation (Fig 1) is tied in explicitly with the MBSR curriculum, as MBSR focuses on stress reduction. MB-BP retains the stress-reduction curricula from MBSR. Through attention control and self-awareness, participants are trained to detect stressors often earlier on in their experience, before they become overwhelming. Specific practices such as the S-T-O-P (Stop, Take a breath, Observe and Open towards the experience, Proceed) are taught to enable participants to pause when stressors are present, turn towards them with their enhanced attention control and self-awareness to observe their experience, and then to proceed in skillful ways moving forward. More detailed qualitative findings on how these self-regulation mechanisms were actually experienced by participants will be published in a forthcoming manuscript. Through enhanced heathy emotion regulation, participants may find that stress reactivity declines, along with the associated emotion regulation behaviors that can trigger elevated blood pressure, whether it is stress-induced sympathetic nervous system activity, emotional eating, or adverse coping behaviors such as excessive alcohol consumption. Overall, single-arm trial quantitative evidence in the current paper supports that attention control, self-awareness and emotion regulation may be improved by the MB-BP intervention, which could lead to improved determinants of hypertension.

There is evidence that several, but not all, modifiable determinants of blood pressure were influenced by the MB-BP intervention. Upon entering the study, participants were diverse in which determinants of blood pressure they needed to change. Some arrived very physically fit, but wanted to improve their diet. Others had healthy dietary patterns, but wanted to reduce stress. Any combination of hypertension determinants, or lack thereof, was possible. MB-BP educated participants about risk factors and treatments of hypertension, showed them their personal expertly-assessed hypertension determinant levels at the beginning of the course, and encouraged them to decide which modifiable determinants of blood pressure they want to work on during the course. The curriculum supported them to go down whichever risk factor reduction path(s) they choose. The data suggest that this patterning played out during the course. Specifically, while physical activity, DASH diet score, alcohol consumption, and salt intake did not significantly change for the entire population, it significantly improved in those who were not adhering to AHA guidelines for physical activity, salt, and alcohol at baseline,[3, 58] or who had low DASH diet score.[41] Furthermore, MB-BP may improve stress levels for the overall study population. These findings are generally consistent with other studies. The minimal research at this time shows early indications for impacts of mindfulness interventions on physical activity.[69–71] While studies to date suggest possible effects of mindfulness on eating behaviors such as binge eating and emotional eating,[72] there is a dearth of information on the relation of mindfulness with evidence-based dietary risk factors for hypertension such as the DASH diet, Mediterranean diet, salt consumption and caloric restriction. Impacts of mindfulness interventions on weight loss show heterogeneity.[73] While the field is still young with relatively few findings or effective customized interventions for weight loss, the most consistent evidence on weight loss is for Acceptance and Commitment Therapy, with fewer consistent effects demonstrated for other mindfulness interventions.[73] Findings in the current study show a significant BMI reduction at 3 months follow-up in those who were overweight or obese at baseline, but effects were not statistically significant from baseline at later follow up times.[7, 74] This study shows novel findings that both DASH diet adherence and added salt may be reduced with the MB-BP intervention. Systematic reviews about impacts of mindfulness interventions on stress suggest significant reductions, which are consistent with what was found in the current study.[74, 75]

This study’s methodological approach utilized the recommended NIH Stage Model and SOBC framework.[14, 76, 77] The SOBC framework uses an experimental medicine approach that emphasizes four steps, described in more detail elsewhere: (1) Identifying an intervention target (i.e. a factor hypothesized to involved in the health behavior/outcome); (2) Developing valid and reliable assays (i.e. measures) of the target; (3) Engaging the target through experimental manipulations or interventions; and (4) Testing the degree to which the target is engaged and determining the degree to which this engagement produces the desired behavior/health change. This study identified proximal self-regulation targets, and intermediate health behavior targets (Step 1), and evaluated them utilizing validated measures (Step 2).[76] Evidence from this study suggested that MB-BP engaged most of the proximal, intermediate, and distal targets (Step 3), although as a single arm Stage 1 clinical trial, the lack of a control group is important to note so as not to overattribute causality to these findings until replicated in an RCT design by multiple research groups. Mediation analyses are required to evaluate if changes in proximal self-regulation mechanisms translate into behavior change and blood pressure change (Step 4). These analyses require statistical power beyond the current study, and will be investigated in the future.

Findings in this study suggest floor effects for impacts of MB-BP on blood pressure being greatest in participants with Stage 2 hypertension, which is consistent with a 2014 systematic review and meta-analysis where the largest blood pressure reductions were shown in the study with the highest baseline blood pressure, including only participants with BP>140/90 mmHg.[78] Given no mindfulness interventions to date have been customized to patients with elevated blood pressure, this study’s findings provide initial support that customizing MB-BP to modifiable determinants of blood may be effective. [11, 79]

Strengths of the study include reasonably long-term follow-up to one-year. Furthermore, it implemented an adapted mindfulness-based program, grounded in both the evidence-based MBSR, as well as in hypertension etiology and treatment. This may enable a more effective intervention than others that do not customize to the realities of patients with elevated blood pressure. The principal investigator (E.L.) who was also the MB-BP instructor, did not have access to the data file, and did not perform the statistical or qualitative analyses which were performed by an independent data analyst (Y.L.), or three trained co-authors (W.N., A.W., J.W.) who coded and analyzed qualitative data. Additional MB-BP instructors are now trained and certified, and are instructing an ongoing MB-BP randomized controlled trial. The study used mixed qualitative and quantitative methods, which is unique in the mindfulness field. This allows for both quantitative *a priori* hypothesized evaluated targets, as well as open ended feedback that scientists may not have hypothesized beforehand. The findings suggested good acceptability and feasibility, with low participant drop out (10%). Several outcomes were directly objectively measured, such as the SART, body mass index, antihypertensive medication adherence (via eCAPS), and blood pressure. There are several limitations. This was a single arm trial, without a control condition, consequently it is uncertain if improvements in self-regulation, health behaviors, and blood pressure were due to alternative causes such as regression to the mean, repeated exposure to measures, or the Hawthorne effect (i.e. alteration of behavior due to participants’ awareness of being observed). In another study evaluating impacts of MBSR vs. progressive muscle relaxation control, there was a clinic-assessed SBP reduction of 4.9 mmHg in the MBSR group, and 0.7 mmHg reduction in the control group, suggesting minimal reductions over time in control group conditions for participants in a mindfulness-based program study.[80] Furthermore, several mechanisms were self-reported. Increased use of objective measures will help reduce possibilities of bias from self-report. Clinic-assessed blood pressure, while still the standard for clinical decision making for hypertension, is imperfect because of issues such as white coat hypertension and masked hypertension.[3] Blood pressure readings at other times of day, such as through home blood pressure monitoring or ambulatory BP monitoring will strengthen clinical relevance of findings.[80, 81] Although ambulatory BP monitoring has superior predictive value for CVD outcomes compared to clinic BP, evidence is lacking whether a reduction in ambulatory BP from antihypertensive treatment is related to a reduction in CVD outcomes.[47, 82, 83] With the study sample being predominately white race/ethnicity and highly educated, it will be necessary to determine if effects are similar in other racial/ethnic and socioeconomic groups. Replication evaluations of the MB-BP interventions by other independent groups will be important. As a result of these limitations, we are currently conducting a Stage 2a clinical trial (ClinicalTrials.gov #NCT03256890) to address these limitations effectively.

## Conclusion

This study provides early evidence that a mindfulness-based program adapted to participants with elevated blood pressure is acceptable and feasible. It may engage with self-regulation measures hypothesized to be mechanisms between mindfulness-based programs and blood pressure, including attention control, self-awareness and emotion regulation. Several modifiable determinants of blood pressure were significantly improved at 1 year-follow-up in participants not adhering to AHA guidelines at baseline including physical activity, DASH diet, salt intake, and alcohol consumption. Perceived stress was also significantly lowered. While findings are still early, and limited by being a single-arm clinical trial, they provide insight into informing the theory that, in the current social context and food environment, when we are surrounded by low cost palatable foods, sedentary occupations and pastimes, and fairly easily accessible alcohol, that mindfulness approaches, particularly those that deliberately engage with these health behaviors, may be utilized as a skillful approach to navigate through to optimal health.

## Supporting information

S1 Table Overview of how MB-BP customized Mindfulness-Based Stress Reduction (MBSR). All MBSR modules are maintained (not shown here), but many are slightly abbreviated to make room for the novel MB-BP modules shown below.
(PDF)

S2 Table Focus group protocol. (PDF)

S3 Table Participant ratings on usefulness of MB-BP customizations. (PDF)

S4 Table Open ended survey on preferred length of class session and all-day retreat. (PDF)

S5 Table Sustained attention to response task outcomes at baseline, and following MB-BP intervention. (PDF)

S6 Table Multidimensional assessment of interceptive awareness scale scores at baseline, and following MB-BP intervention. (PDF)

S7 Table Physical adverse events. (PDF)

S1 File Mindfulness-Based Blood Pressure Reduction (MB-BP) study protocol. The file contains the full protocol, including all amendments approved by the Brown IRB that occurred during the course of the study. Brown’s IRB requires track changes to and they are included so that the protocol is as accurate and clear as possible.
(PDF)

S2 File TREND statement. TREND statement for Mindfulness-Based Blood Pressure Reduction Study Stage 1 Manuscript.
(DOCX)
