# Supplementary material for: Mindfulness-Based Blood Pressure Reduction (MB-BP): Stage 1 single-arm clinical trial
Source: PLoS One. 2019 Nov 27;14(11):e0223095. doi: 10.1371/journal.pone.0223095 (PMC6881004; doi:10.1371/journal.pone.0223095)
Supplement: S1 Table — All MBSR modules are maintained (not shown here), but many are slightly abbreviated to make room for the novel MB-BP modules shown below. (PDF) [file pone.0223095.s001.pdf]

Supporting Information Table 1: Overview of how MB-BP customized Mindfulness-Based Stress Reduction (MBSR). All MBSR modules are maintained (not shown here), but many are slightly abbreviated to make room for the novel MB-BP modules shown below.

Module	Intervention Component	Customization Description	N w (N) or modified (M)*	In Class (IC) v Home Practice (HP)	Time (min)
Orientation Session	Personalized Health Feedback on Hypertension Risk Factors	Provide participants health cards showing their baseline-assessed BP and hypertension risk factor levels, including *SBP, *DBP, *BMI, diet, physical activity, perceived stress, sleep, and antihypertensive medication use.	N	*IC*	10
	Introduce Impact of Hypertension on Cardiovascular Disease	Describe key modifiable hypertension risk factors, how the current societal environment promotes hypertension, how mindfulness meditation can impact risk factors.	N	*IC*	30
	Course Logistics Overview	Explore our behavioral, physical, emotional and cognitive patterns related to hypertension risk, more skillful responses to these patterns, as well as more mindful ways to communicate and choose nourishing behaviors and activities.	M	*IC*	10
1:1 Interviews	Individual Intake Interviews for Instructor with Each Participant	Participants are asked (1) what brought them to be interested in learning about mindfulness at this time in their lives; and (2) what is their relationship with determinants of blood pressure such as diet, overweight/obesity, physical activity, alcohol consumption, stress reactivity and antihypertensive medication use. Conversational explorations occur determining in what areas participants are ready to change, and how best the course can support them to do so.	M	*IC*	20
Class 1	Introduction Behavior Change Theory & Hypertension	Overview of the theory of behavioral medicine and the application of self-regulatory skills as related to hypertension.	M	*IC*	10
Class 2	Mindful Eating of Highly Palatable (e.g. high sugar/fat/salt) Food Item	Participants invited to engage in eating a highly palatable snack, and reflect on the effects they experience in thoughts, emotions, and physical sensations.	N	*IC*	20
	Pleasant Events Calendar	Suggest targeting pleasant events related to hypertension risk (e.g. eating or alcohol consumption).	M	*HP*	20
Class 3	Information & Review of Basic Forms of Physical Activity	(1) Strength training; (2) Aerobic training; (3) Flexibility training; according to American Heart Association guidelines. Dyad & group discussion.	N	*IC*	25
	Unpleasant Events Calendar	Suggest targeting unpleasant events related to hypertension risk (e.g. eating or alcohol consumption).	M	*HP*	20
	Physical Activity	Mindful attention to thoughts, emotions and physical sensations related to the physical activity beforehand, during and after engaging in the activity.	N	*HP*	30
Class 4	Aerobic Physical Activity	Walking/jogging, allowing modifications of physical activity as needed (e.g. body weight-based strength exercises). Bringing mindful awareness to physical activity, especially thoughts, emotions and physical sensations before, during and after physical activity. Group discussion.	N	*IC*	20
	Motivational Interviewing Module	Goal setting worksheet related to improving a determinant of blood pressure during the coming week; group discussion.	M	*IC*	20
	Blood Pressure Determinant Goal Setting	Pick a do-able goal related to diet, alcohol consumption, or physical activity for the week that participants feel may help them (e.g. eating more healthily, engaging in particular physical activity); make it specific; pick a way to measure it.	M	*HP*	
Class 5	Blood Pressure Determinant Goal Break Out Groups	Small group sharing, where group members cluster by blood pressure determinant they set goals on during prior week (e.g. physical activity, diet). Share experiences practicing with their goal over the past week.	N	*IC*	15
	Medication Adherence Group Discussion	Hand out fact sheet on antihypertensive medication classes, including potential benefits and adverse effects. Explore through group discussion participants' relationship with antihypertensive medication use. Sharing how it can be skillful to take medication if that is what body needs.	N	*IC*	15
	Motivational Interviewing Module	Goal setting worksheet related to improving a determinant of blood pressure during the coming week; group discussion.	M	*IC*	15
	Blood Pressure Determinant Goal Setting	Pick a do-able goal related to diet, alcohol consumption, physical activity or antihypertensive medication use for the week that participants feel may help them; make it specific; pick a way to measure it.	M	*HP*	
Class 6	Blood Pressure Determinant Goal Break Out Groups	Small group sharing, where group members cluster by blood pressure determinant they set goals on during prior week (e.g. physical activity, alcohol, diet). Share experiences practicing with their goal over the past week.	N	*IC*	20
	Social Support Module	Journaling exercise picking one of the areas you are exploring shifting (e.g. meditation, physical activity, diet, medication adherence) and the social support available in the participants life to support this goal. Group discussion.	N	*IC*	20
	Blood Pressure Determinant Goal Setting	Pick a do-able goal related to diet, alcohol consumption, physical activity, antihypertensive medication use, or increasing social support related to determinant of blood pressure, for the week; make it specific; pick a way to measure it.	M	*HP*	
Class 7	Optional Blood Pressure Determinant Goal Setting	Invitation, if participants would like to, pick a do-able goal related to determinant of blood pressure, for the week; make it specific; pick a way to measure it.	M	*HP*	10
Class 8	Theoretical Mechanism for Mindfulness on Cardiovascular Disease	Show the framework through which mindfulness may influence blood pressure and heart health, and how this course worked with those pathways.	N	*HP*	20
All-Day retreat	Aerobic Physical Activity	Silent aerobic or strength physical activity. Aware of physical sensations, thoughts and emotions.	N	*IC*	40
	Dietary Approaches to Stop Hypertension (DASH) diet lunch	DASH diet & 2015-2020 Dietary Guidelines for Americans inviting participants to fill their plates accordingly, noticing physical sensations, thoughts and emotions during the entire arch of lunch, including before, during, and after.	N	*IC*	60
	Self-Care Written Reflection	Invite participants to write about self-care in response to pre-set prompts.	N	*IC*	15

**BMI, body mass index; DBP, diastolic blood pressure; SBP, systolic blood pressure*.
