# Supplementary material for: Mindfulness-Based Blood Pressure Reduction (MB-BP): Stage 1 single-arm clinical trial
Source: PLoS One. 2019 Nov 27;14(11):e0223095. doi: 10.1371/journal.pone.0223095 (PMC6881004; doi:10.1371/journal.pone.0223095)
Supplement: S2 Table — (PDF) [file pone.0223095.s002.pdf]

**Supporting Information Table 2.** Qualitative protocol agenda and questions.

| Agenda | Time completed |
|---|---|
| Research Assistant downstairs directing participants to focus group room location. Set up name tents.<br>Test recorder.<br>Turn on recorder, place in the middle of the table. | **5:30pm** |
| Chatting and eating. | **5:45pm** |
| Welcome and introduction<br>• Thank you for sharing your thoughts on MB-BP<br>• Introductions<br>    ○ Facilitator<br>    ○ Note taker<br>• Why and How<br>    ○ We are trying to improve the intervention<br>    ○ Discussing your opinions on the different activities<br>    ○ Please share your point of view, even if it's different from others<br>    ○ No wrong answers<br>    ○ We equally welcome positive and negative feedback<br>• Suggestions<br>    ○ Suggestions to help us have a good discussion<br>        ▪ Speak up<br>        ▪ Audio recording, one person speaks at a time<br>        ▪ Any report that we write about what we hear today will not be associated with your identity<br>        ▪ Tendency for some people to be comfortable speaking up more than others. It's important to us to hear from everyone today. So, I may ask you to share if I haven't hear from you. Or I may ask you to let others share if you are sharing a lot.<br>• What to expect<br>    ○ My role is to listen, facilitate discussion between you all and move us along 5 questions.<br>    ○ In the interest of time, I may have to move the discussion along so that we can cover all 5 questions<br>    ○ Let's begin | **5:50pm** |
| Allow participants to look over class overview.<br>1. Looking at the list of course activities, which is most memorable for you? Why? | **5:50pm** |
| **Card Sorting Activity:**<br><br>Give them cards showing different activities.<br><br>Put cards for activities you found very useful in one pile, modules that you thought were somewhat useful in another pile, and activities you thought were not useful in a 3rd pile.<br><br>Please place the cards in the piles according to whether you found them "very useful," "somewhat useful," or "not useful." | **6:03pm**<br><br>**6:08pm** |
| Note taker writes the questions shown below on the board.<br>1.What was most helpful about this course, and why?<br><br>**<u>Suggested Probes</u>:**<br>Was there something you learned in the course that was helpful?<br>Could you give an example from your experience?<br>Was there a specific activity that you found most helpful? | **6:12pm** |
| 2. After going through this mindfulness intervention, what is your understanding of how it works to improve your blood pressure?*<br><br>**<u>Suggested Probes</u>:**<br>Could you give an example from your experience?<br>Have you seen changes in your blood pressure?<br>If yes what do you attribute to causing these changes? | **6:18pm** |
| 3. We want to make this intervention better. You have been through it once. How do you think we can make it better?*<br><br>**<u>Suggested Probes</u>:**<br>Where there any activities (e.g. breakout groups, you think could be improved?<br>Were there any improvement on the space for the class?<br>Is there anything you think we can do to improve on the communications? (e.g. email, phone, text) | **6:40pm** |
| 4. Every instructor can improve. How can this instructor improve?<br><br>**<u>Suggested Probes</u>:**<br>Was the communication clear?<br>Did you feel like you were understood? | **6:50pm** |
| Pass out survey.<br>"I'm going to hand out a quick survey, which I'd like for you to fill out." | **6:55pm** |
| Note taker summarizes the discussion.<br><br>Is there anything we should have talked about, but didn't? | **7:00pm** |
