# Supplementary material for: Mindfulness-Based Blood Pressure Reduction (MB-BP): Stage 1 single-arm clinical trial
Source: PLoS One. 2019 Nov 27;14(11):e0223095. doi: 10.1371/journal.pone.0223095 (PMC6881004; doi:10.1371/journal.pone.0223095)
Supplement: S3 Table — (PDF) [file pone.0223095.s003.pdf]

Supporting Information Table 3. Participant ratings on usefulness of MB-BP customizations, assessed using closed card sort methodology.

Description of Intervention Component	Very Useful (2 points)		Useful (1 point)		Not Useful (0 points)		
	# of votes	%	# of votes	%	# of votes	%	Mean Score
1. In Class: Sweet, palatable snack meditation (e.g. eating sweet muffins) and discussion	8	*50%*	5	*31%*	3	*19%*	1.31
2. Home Practice: Fill out Pleasant Events Calendar for the week for events related to eating and alcohol consumption.	4	*25%*	7	*44%*	5	*31%*	0.93
3. Home Practice: Unpleasant Events Calendar for the week for events related to eating or alcohol consumption.	3	*19%*	5	*31%*	8	*50%*	0.68
4. Home Practice: Engage in one 20-30 min type of physical activity other than yoga (e.g. strength training, aerobic training). Similar to the mindfulness in daily activities home, practice bringing mindful attention to thoughts, emotions and physical sensations related to the physical activity beforehand, during and after engaging in the activity.	11	*69%*	4	*25%*	1	*6%*	1.63
5. In Class: Engage in 20 minutes of aerobic physical activity, with additional 5 minutes of warm-up, and 5 minutes of yoga afterwards – specifically walking/jogging at a fairly fast pace, preferably outside. Monitor thoughts, emotions and physical sensations prior to starting the walk, during, and after.	8	*50%*	7	*44%*	1	*6%*	1.44
6. In Class: Picking a goal related to diet, alcohol consumption or physical activity for the week. (1) What is your goal related to diet, alcohol consumption, physical activity, or stress for the week? (2) On a scale of 1-10, where 10 is high, how MOTIVATED are you to achieve this goal? (3) On a scale of 1-10, how CONFIDENT are you that you will achieve the goal? (4) What could you do that would bring your motivation or confidence a little higher? (5) What might make it difficult to achieve the goal this week, and if that happens, what will you do? (6) What is a way to measure this goal that resonates with you?	11	*69%*	3	*19%*	2	*13%*	1.56
7. Home Practice: Pick a do-able goal related to diet, alcohol consumption, or physical activity for the week may be helpful (e.g. eating less, eating more healthily, engaging in particular type of physical activity).	8	*50%*	7	*44%*	1	*6%*	1.44
8. In Class: Move into groups of same hypertension risk factors that they focused on last week during Home Practice. Within groups, share experiences with practicing with this risk factor.	10	*63%*	4	*25%*	2	*13%*	1.50
9. Home Practice: Pick a NEW goal related to diet, alcohol consumption, physical activity, medication adherence, or increasing social support related to a behavior change, for the week that will be helpful.	9	*56%*	7	*44%*	0	*0%*	1.56
10. In Class: Discuss theme of what is taken in, as food or as any kind of sensory experience, and patterns that are self-destructive and self-nourishing. Work through the Nourishment/Poison worksheets. In partners, ask nourishment questions while the other responds.	8	*50%*	4	*25%*	4	*25%*	1.25
11. All-Day Class: Writing about self-care. e.g. through free writing that no one else will see, respond to queries…How do I care for myself (1) in general, and (2) in specific ways that can lower blood pressure? How would to practice more self-care (1) in general, and (2) in specific ways that could lower blood pressure? Would caring in these ways help others? If so, how?	8	*50%*	6	*38%*	2	*13%*	1.38
