# Supplementary material for: Mindfulness-Based Blood Pressure Reduction (MB-BP): Stage 1 single-arm clinical trial
Source: PLoS One. 2019 Nov 27;14(11):e0223095. doi: 10.1371/journal.pone.0223095 (PMC6881004; doi:10.1371/journal.pone.0223095)
Supplement: S4 Table — (PDF) [file pone.0223095.s004.pdf]

Supporting Information Table 4: Open Ended Survey on Preferred Length of Class Session/All Day Retreat

FG**/ IDI***	Class Length Preference (hrs)	Notes	Retreat Length Preference (hrs)	Notes
Average	2.43		6.93	
FG 1	2.5	It felt long enough to be effective and not too long where I felt restless or the need to rush back to work.	7.5	I also felt like the time allotted was perfect. I was a bit concerned about it being too long but at the end it went by quickly and was very enjoyable.
FG 1	2.5	We were able to meditate a few times had instruction time and time to review various things about the practice at home and in the sessions.	7.5	I enjoyed the retreat but I was exhausted at the end of it. Although, I think 7.5 hours worked because of the variety of things we did.
FG 1	2.5	2.5 hours was perfect for me.	N/A*	
FG 1	2.5	Length was good. 2.5 hours before work does seem to allow for practice.	6	A break in the middle away from the room would be better. 3 hours AM and 3 hours PM (and coffee please).
FG 2	3	Use the last hour for discussion.	6	6 hours would have been enough.
FG 2	2.5	Perhaps an open forum discussion to extend 3hours on a voluntary basis	7.5	We needed the long day to show us/allow us to be mindful for our self care all day.
FG 2	3	3 hours every other week and then extend.	N/A*	
FG 2	2.25	As the session is a mixture of activities and lecture the time well utilized.	6	My concentration was shot and I was exhausted from learning and new activities. Cut out 1 walk.
FG 2	2.5	About the right time. Not too tie. At most 3 hours.	7.5	7.5 is right. Maybe 7. It was a long tiring.
FG 2	2.5	it was the right amount to really immerse in the intervention.	7	7.5 seemed difficult for so much silent.
FG 2	2.5	There was so much material to cover and so much information to get across. It never felt too long.	7.5	I valued the retreat day. 7.5 hours was really good.
FG 2	2.5	It was late in the day and starting at 5:30 ending at 8:00 was just right.	N/A*	
FG 3	2	I would have preferred 2 hours and fewer activities felt mentally fatigued.	6	I would say 6 hours again …I felt a bit mentally fatigued.
FG 3	2.5	I feel that 2.5 hour was a very appropriate amount of time the way the classes were structured. I never felt like class was too long or too short. I would leave it to 2.5 hours	8	I think the retreat could have been a little longer at 8 hours. I did not want it to end.
FG 3	2.5	I found the time convenient. Not too early, not too late.	8	8 hours would be fine with me but too much for others. It seemed like we only had about half the class for the retreat. It didn't feel long. I would have no problem staying the 8 hours.
FG 3	2.5	In the beginning it seemed long. But that changed as the time went by. 2.5 was good.	7.5	7.5 was fine.
FG 3	2.5	No 2.5 was good.	7.5	7.5 seemed just right.
FG 3	2.5	2.5 hours seemed adequate to cover content.	7.5	it was perfect and allowed time to integrate existing knowledge and add new content- besides to eat and move with leisure.
FG 3	3	All more time for creativity.	8	8 hours (9-5) - allow more time to introduce a poetry or art exercise.
IDI 1	3	Maybe 3 I mean I know it's got to be a fine balance between giving a room and people having time but it did feel sometimes like we had to sort of shorten things at the end because like we would have had a discussion and it might have gone long or a little bit longer.	7.5	I do not remember my feelings about that I don't think I had any negative ones I don't think I was like " oh this is too long" again it's like …yeah probably that day felt like plenty of time because like there was no one else at the university and you know I think I really enjoyed that day and didn't feel like it was too long or too short.
IDI 2	2.5	Whether you keep it at 2 hours and maybe go a week longer, or extend it, I think, I would leave it to your devices to figure out what makes sense from a design of the intervention, but the block of time that's being able to commit to, that's being able to commit to that block of time anyway so, whether two or three wouldn't matter to me personally.	6.5	It might make it a little easier, uh. Like I said, that could just be me. If I were to do it, I would end it's at 3 o'clock, and um, yeah.
IDI 3	2.5	I don't, no, it was fine I was probably tired, (laughs) at the end of two and a half hours, but, no. I, they tended to go by quickly, I thought the sessions, yeah, were fine as they were, in terms of length.	7.5	I was pretty exhausted, but, but that was expected. Um after that, knowing what to expect, um that time was fine, I don't know that it, I, I can't, I think extending it beyond I'm not sure that would have, you know more than seven and a half I think that, you know as I said I think I was pretty exhausted. I'm always at the point where, whereas it wraps up, you know I'm feeling fabulous but I'm tired. So I think the length of it for me is fine.
IDI 4	2.5	Yeah, I think two and a half is probably good. I don't feel like if it was any shorter, then I don't think you could get to everything and you know after two and a half hours, like that might be too long.	7	Well, you have lunch so, that took up like an hour so, I'd say probably seven is good, like three hours in the morning, lunch, and then another three hours.
IDI 5	2	Yea actually I think possibly that's a possibility that they could be shortened to 2 hours.	4	Yeah that was quite long I think that could probably be half a day ? Then we would be able to accommodate what they needed to do as far as relaxation and going outdoors you know. That felt a little long. Yeah I think maybe 4 hours.
IDI 6	2.75	Um.. it could… it could be increased time wise because I think that um it would just reinforce the discussions and exercises that we shared during each meeting. [...] If… let's see. (pause) I think each meeting seemed to go by very quickly there was no drag in my experience of it. I um don't know if it would be.. certainly not reduced the time. I'm not sure how beneficial extending the time would be. I … I um… lets see I could go longer then 2 hours but … but I'm not sure how much I would think no more then an additional 1.	5	I think 5 hours was good. (confusion as to how long said the length of the day was good) I wouldn't decrease the time but I wonder in addition to wondering about myself I wonder about how long people attention could be held without drifting away on thoughts going of the thoughts going on the sessions.
IDI 7	2.5	Well it worked for me. You know I could spend that amount of time but I'm conjecturing that if you wanted to work with people that are really wound up, have the type A personalities, that have some health risk because of that um type of life they live.. you're gonna have a hard time getting those folks in to spend that kind of time.	7.5	Because everything about the course was fine for me. I wouldn't have any criticism I enjoyed that day. And um you know the one thing I would say is that if you could marry that retreat day with a change in venue. Get some place away from the city. Um you might get even more enjoyment out of it. So be back in the same classroom it was and there's so many good options especially if you are doing it in the summer like we did were you could… experience a deeper level of peace or achieve that level of peace more readily because you wouldn't have the business of the city.

participant did not attend the all-day retreat ** Focus Group (FG) number indicates the cohort associated with FG * In-depth Interview (IDI); number indicates the participant ID number*
