# Supplementary material for: Mindfulness-Based Blood Pressure Reduction (MB-BP): Stage 1 single-arm clinical trial
Source: PLoS One. 2019 Nov 27;14(11):e0223095. doi: 10.1371/journal.pone.0223095 (PMC6881004; doi:10.1371/journal.pone.0223095)
Supplement: S5 Table — (PDF) [file pone.0223095.s005.pdf]

Supporting Information Table 5. Sustained Attention to Response Task outcomes at baseline, and following MB-BP intervention.

	Baseline				3 months					6 months					12 months				
	n	Point Estimate	SD	95% CI	n	Point Estimate	SD	95% CI	p	n	Point Estimate	SD	95% CI	p	n	Point Estimate	SD	95% CI	p
CorrGo	42	868.2	72.8	846.0, 890.4	43	883.8	77.4	860.4, 907.1	0.10	36	875.4	77.1	850.1, 900.8	0.19	41	899.3	61.1	880.5, 918.2	**0.001**
TotalGo	42	939.8	39.6	927.7, 951.8	43	943.5	45.5	929.8, 957.2	0.53	36	943.1	47.3	927.6, 958.7	0.36	41	950.4	32.2	940.5, 960.4	0.07
CorrNoGo	42	49.1	12.6	45.2, 52.9	43	53.7	12.1	50.0, 57.3	**0.006**	36	53.6	10.2	50.2, 56.9	**0.004**	41	56.3	9.4	53.4, 59.2	**<0.001**
TotalNoGo	42	70.5	2.9	69.6, 71.3	43	70.5	3.4	69.4, 71.5	0.99	36	70.6	2.7	69.7, 71.5	0.70	41	71.1	2.0	70.5, 71.7	0.19
CorrGoPercent	42	92.3	5.5	90.6, 94.0	43	93.5	5.1	92.0, 95.1	0.06	36	92.7	5.2	91.0, 94.4	0.24	41	94.6	4.6	93.2, 96.0	**0.001**
CorrNoGoPercent	42	69.5	16.9	64.2, 74.5	43	75.9	15.6	71.2, 80.6	**0.002**	36	75.6	13.0	71.3, 79.9	**0.002**	41	79.1	12.3	75.3, 82.9	**<0.001**
TotalCorrPercent	42	90.7	6.0	88.9, 92.5	43	92.3	5.6	90.6, 94.0	**0.02**	36	91.5	5.5	89.7, 93.3	0.09	41	93.5	4.9	92.0, 95.0	**<0.001**
NumOmissionError	42	71.6	50.7	56.1, 87.0	43	59.7	45.5	46.0, 73.4	**0.05**	36	67.7	46.7	52.3, 83.1	0.23	41	51.1	42.5	38.0, 64.2	**0.001**
NumCommissionError	42	21.4	11.3	18.0, 24.9	43	16.8	10.7	13.6, 20.0	**0.002**	36	17.1	8.7	14.2, 19.9	**0.002**	41	14.8	8.5	12.1, 17.4	**<0.001**
PercOmissionError	42	7.7	5.5	6.0, 9.4	43	6.5	5.1	4.9, 8.0	0.06	36	7.3	5.2	5.6, 9.0	0.24	41	5.4	4.6	4.0, 6.8	**0.001**
PercCommissionError	42	30.7	16.9	25.5, 35.8	43	24.1	15.6	19.4, 28.8	**0.002**	36	24.4	13.0	20.2, 28.7	**0.002**	41	20.9	12.3	17.1, 24.7	**<0.001**
CorrGo_mRT	42	382.6	97.8	352.8, 412.4	43	377.0	75.3	354.4, 399.7	0.63	36	372.3	81.5	345.6, 399.2	0.82	41	391.8	88.1	364.6, 418.9	0.34
IncorrNoGo_mRT	42	307.8	63.1	288.5, 327.0	42	290.6	39.7	278.5, 302.7	0.17	35	286.3	43.9	271.6, 300.9	0.20	41	318.9	100.4	287.9, 349.9	0.28
CorrGoBeforeCorrNoGo_mRT	42	401.6	98.8	371.4, 431.7	43	397.7	77.9	374.3, 421.2	0.72	36	391.9	83.2	364.6, 419.3	0.87	41	411.0	88.7	383.6, 438.3	0.30
CorrGoBeforeIncorrNoGo_mRT	42	334.7	66.8	314.3, 355.0	42	326.5	47.1	312.1, 340.8	0.48	36	330.7	58.7	311.4, 350.0	0.99	41	347.2	73.2	324.6, 369.8	0.20
CorrGoAfterCorrNoGo_mRT	42	347.9	105.9	315.6, 380.1	43	341.5	88.8	314.7, 368.2	0.63	36	336.3	95.3	304.9, 367.6	0.96	41	351.7	96.8	321.9, 381.6	0.47
CorrGoAfterIncorrNoGo_mRT	42	440.2	150.7	394.3, 486.1	42	393.8	129.2	354.5, 433.2	**0.02**	35	354.6	109.9	317.9, 391.2	**0.001**	41	401.4	165.4	350.4, 452.4	0.07
Aprime	42	0.8	0.1	0.8, 0.8	42	0.8	0.1	0.8, 0.8	0.22	35	0.8	0.1	0.8, 0.8	0.53	41	0.9	0.1	0.8, 0.8	**0.003**
Dprime	42	2.2	1.0	1.9, 2.4	43	2.5	1.0	2.2, 2.8	**0.02**	36	2.4	1.0	2.1, 2.7	0.04	41	2.7	0.9	2.4, 3.0	**<0.001**

Analyses were performed using hierarchical linear models. P values represent comparison of respective follow-up time to baseline.

Aprime, non-parametric perceptual sensitivity index ranging between .5 (indicating signal cannot be distinguished from noise, to 1, corresponding to perfect performance); CorrGo, mean number correct Go trials; CorrGoAfterCorrNoGo_mRT, mean RT of correct Go trials following correct NoGo trials; CorrGoAfterIncorrNoGo_mRT, mean RT of correct Go trials following incorrect NoGo trials; CorrGoBeforeçorrNoGo_mRT, mean RT of correct Go trials preceding a correct NoGo trial; CorrGoBeforeIncorrNoGo_mRT, mean RT of correct Go trials preceding an incorrect NoGo trials; CorrGo_mRT, mean reaction time (RT) for correct Go trials; CorrGoPercent, percent correct for Go trials; CorrNoGo, mean number of correct NoGo trials; CorrNoGoPercent, percent correct of NoGo trials; Dprime., perceptual sensitivity index to target.IncorrNoGo_mRT, mean RT for incorrect NoGo trials; NumOmissionError, mean number of omission errors or failures to respond to a nontarget “1-2,4-9”; NumCommissionError, mean number of commission errors or failures to withhold a response to a target, “3”; PercOmissionError, percent of trials in which there were omission errors; PercCommissionError, percent trials with commission errors; TotalGo, mean number of Total Go Trials; TotalNoGo, mean number of total NoGo trials; TotalCorrPercent, percent correct of total trials.
