# Supplementary material for: Mindfulness-Based Blood Pressure Reduction (MB-BP): Stage 1 single-arm clinical trial
Source: PLoS One. 2019 Nov 27;14(11):e0223095. doi: 10.1371/journal.pone.0223095 (PMC6881004; doi:10.1371/journal.pone.0223095)
Supplement: S7 Table — (PDF) [file pone.0223095.s007.pdf]

Supporting Information Table 7. Physical Adverse Events

Date Reported	A/E Onset	Severity*	SAE*	Relationship to study*	Action Taken	Outcome*	Additional Comments
07/21/2016	07/2016	1	2	4	0	1	Injured left shoulder during yoga exercises in class; followed up and issues have been resolved
08/03/2016	07/2016	2	2	0	0	1	Bruising, swelling on top of right foot due to post-surgical swelling and/or bone spur
09/06/2016	09/2016	1	2	0	0	1	Bruised toe when tripped in driveway
09/06/2016	09/2016	1	2	1	0	1	Old injury to thigh, hip and back faired up after jogging or possibly from sleeping on hard bed
10/10/2016	10/2016	1	2	1	0	1	Injured back when playing with grandchildren. Treated with rest, anti-inflammatory and pain medications
11/01/2016	10/2016	1	2	1	0	1	Injured back while cleaning
11/01/2016	10/2016	1	2	1	0	1	Sciatic nerve issues after participating in yoga class and music lessons
11/03/2016	10/2016	1	2	0	0	1	Sprained finger while carrying heavy object
11/18/2016	11/2016	2	2	0	4	1	Ruptured Achilles' tendon playing basketball discontinued intervention due to pain & decreased mobility.
12/02/2016	11/2016	1	2	1	0	1	Strained muscles in back, shoulders and neck while cleaning
12/02/2016	11/2016	1	2	1	0	1	Sprained ankle while playing racquetball
12/04/2016	11/2016	1	2	0	0	1	Lost balance and hit head on edge of door; bruising and swelling
01/05/2017	01/2017	1	2	0	0	1	Injured left knee after tripping over cat and falling on hard kitchen floor
01/05/2017	12/2016	1	2	0	0	1	Injured toe and reported having a benign mass removed from upper left chest
01/06/2017	01/2017	1	2	0	0	1	Fell climbing up stairs and fractured and lacerated her nose
04/03/2017	03/2017	3	1	1	0	1	Long suffered from back pain. Underwent back surgery (laminectomy) in March 2017.
05/04/2017	08/2017	1	2	0	0	1	Hit by a car while biking. Did not experience any major injuries (e.g., concussion, lacerations)

Scored using National Center for Complementary and Integrative Health Adverse Event Reporting Form using the following ratings:* ***Severity:* *1=mild, 2=moderate, 3=severe, 4=Very Sever 5=death;* ***SAE (Serious Adverse Event)****: 1= Yes, 2= No;* ***Relationship to study:*** *0=not likely, 1=Unlikely related, 2=Possibly related, 3=Probably related, 4=Definitely related;* ***Action Taken:*** *0=none, 1= Treatment modification, 2=Medical intervention, 3=Hospitalization, 4=Treatment discontinued, 5=Other;* ***Outcome:*** *1=Resolved, 2=Recovered with minor sequalae, 3=recovered with major sequalae, 4=Ongoing, continued treatment, 5=Condition worsening, 6=Death, 7=Unknown.*
