# Supplementary material for: Mindfulness-Based Blood Pressure Reduction (MB-BP): Stage 1 single-arm clinical trial
Source: PLoS One. 2019 Nov 27;14(11):e0223095. doi: 10.1371/journal.pone.0223095 (PMC6881004; doi:10.1371/journal.pone.0223095)
Supplement: S1 File — The file contains the full protocol, including all amendments approved by the Brown IRB that occurred during the course of the study. Brown’s IRB requires track changes to and they are included so that the protocol is as accurate and clear as possible. (PDF) [file pone.0223095.s008.pdf]

FULL PROTOCOL TITLE

Mindfulness-Based Blood Pressure Reduction (MB-BP) Study

Study Chairman or Principal Investigator:

Eric B. Loucks, PhD
Assistant Professor, Department of Epidemiology
Brown University School of Public Health

Supported by:
The National Center for Complementary and Integrative Health

1UH2AT009145-01

A NOTE TO READERS:
v.2019.01.11

This document is to serve as a supporting information file to the PLOS ONE manuscript titled, *Mindfulness-Based Blood Pressure Reduction (MB-BP): Stage 1 Single-Arm Clinical Trial*. The study protocol that is found with the following pages contains the complete and detailed plan (in its original language as it was submitted) for the conduct and analysis of the trial that the ethics committee (i.e., Brown University Institutional Review Board (IRB)) approved before the trial began.

The original protocol that was submitted to the Brown IRB on December 5, 2014 was a pilot study and precursor to the MB-BP Stage 1 Single Arm Clinical Trial funded by the NCCIH. In September 2015, the study PI (Loucks) was awarded a five year NIH UH2/UH3 grant (1 UH2 AT009145-01 entitled “Mindfulness Influences on Self-Regulation: Mental and Physical Health Implications”). With this funding, the pilot study approved by the Brown IRB was amended to match the proposed single arm trial approved by NCCIH. Data from the NIH-funded study are presented in this paper. Data from the pilot study are not, as primary outcomes were different between studies.

The Brown IRB reviews and approves modifications to study protocols through the use of stand-alone modification submission forms. That being said, the IRB approved study protocol for *Mindfulness-Based Blood Pressure Reduction (MB-BP): Stage 1 Single-Arm Clinical Trial* is the conglomeration of the original submission along with all approved modification submissions included in this report.

To assist in navigating, please use the hyperlinked table found on the proceeding pages.

Additional questions can be directed to the study investigator: Eric_Loucks@brown.edu.

Mindfulness-Based Blood Pressure Reduction (MB-BP) Study: Brown University
IRB Submission History and Summary of Protocol Amendments

For additional detail on protocol revisions and/or for a copy of the full IRB amendment submissions, with Study PI Signature, contact the Senior Project Coordinator, Frances_Saadeh@brown.edu or study PI, Eric_Loucks@brown.edu.

IRB Submission #	Submitted	Approved	Stage	Summary of Submission / Protocol Revisions
Original Submission	12/5/14	3/12/15	Pilot	The project was submitted to the Brown IRB in December 2014. The IRB requested three iterations of revisions to the submission prior to granting approval. These revisions were submitted: 1/5/15, 2/10/15, and 2/19/15.
Amendment #1	4/8/15	7/1/15	Pilot	This amendment dated 6/17/15, (originally submitted 4/8/15) includes changing the survey software to Qualtrics, updating the phone screening questionnaire, clarifying the study safety plan, modifying the consent form, adding questionnaires to the baseline survey, and modifying recruitment procedures.
Amendment #2	7/15/15	8/6/15	Pilot	This amendment dated 7/15/15 (revised and re-submitted on 8/5/15) included: The addition of a new participant population (NEFS sub-set) and new recruitment procedures for that population, revisions to the In-person Screening Assessments 1 and 2, revisions to wording of the phone screening questionnaire, addition of a place for staff to note contact information on the safety plan document, adding a version footer to the consent document.
Amendment #3	9/1/15	9/3/15	Stage 1	This amendment dated 9/1/15 included the addition of a funding source (grant #1 UH2 AT009145-01, grant title: Mindfulness Influences on Self-Regulation: Mental and Physical Health Implications), a change in scope of work and related study activities secondary data analyses involving concurrent studies, additional measures and study procedures, and revised informed consent document.
Amendment #4	11/11/15	1/7/16	Stage 1	This amendment (dated: 11/11/2015) requests approval to (a) add compensation, (b) add a NIH safety monitoring plan, (c) update the names and content of the phone screen and assessments,(d) add a 1-year follow-up visit, and (e) update the consent document to incorporate these changes.
Amendment #5	2/26/16	2/26/16	Stage 1	This amendment (dated: 2/26/16) requests approval to allow participants, who are not able to come to the study site in person, the option to participate by online video conferencing.
Amendment #6	4/11/16	5/9/16	Stage 1	This amendment (dated: 4/11/2016) requests approval to (a) change the study title; (b) revise the NCCIH Safety Monitoring Protocol; (c) separate the Baseline and Follow-up Assessments into two visits; (d) change the data collection mode to Qualtrics for Daily Practice Forms and Class questionnaires; (e) revise study recruitment material and use online advertising; (f) revise the in-class worksheets and assessments; and (g) revise the MB-BP Questionnaire, In-Person Assessment, Home Assessment, and Measurement of Mindfulness Practices.

IRB Submission #	Submitted	Approved	Stage	Summary of Submission / Protocol Revisions
Amendment #7	8/9/16	9/13/16	Stage 1	Approval of the amendment (memo dated August 9, 2016) includes the addition of 2 new questions to the Home Baseline Assessment.
Amendment #8	5/15/17	6/12/17	Stage 2a	This amendment (dated 5/15/2017) requests approval to (a) begin Stage 2a (previously called Stage 1b) to conduct the Randomized Controlled Trial with enhanced usual care. Stage 2a comes with changes to the study flow, incentives, screening and eligibility, consent document, phone screener, in-person screener, baseline in-person assessment, baseline-online home assessment, safety protocol, and study recruitment material.
Amendment #9	8/26/17	10/13/17	Stage 2a	This amendment (dated: 8/28/2017) requests approval to (a) add an Infographic Card, (b) add a Recruitment Card, (c) conduct a direct mailing at a partnering provider office, (d) add NIH Administrative Supplement funding to award #004754-001 "Mindfulness Influences on Self-Regulation: Mental and Physical Health Implications," IP #17101586, and (e) update the consent document with Certificate of Confidentiality language.
Amendment #10	11/6/17	1/25/18	Stage 2a and additional follow up for Stage 1	This amendment (dated: 11/6/2017) requests approval to (a) modify the approved focus group discussion questions into a one-on-one qualitative phone interview, (b) re-contact Stage 1 participants for a 2-year in-person assessment, (c) rearrange compensation amounts, (d) add recruitment for and data sharing from the UMass Medical School fMRI study, (e) add the "UMass Medical School fMRI Study Recruitment Talking Points," (f) add a consent addendum for enrolled participants to complete the phone interview, and (g) revise the approved consent to reflect the appropriate changes.
Amendment #11	5/8/18	6/13/18	Stage 2a	This amendment (dated: 5/8/2018) requests approval to (a) drop the 12 month and two year follow-ups for Stage 2a participants only; (b) reduce the number of home blood pressure readings and drop the mandatory introduction session that is part of the course; (c) assist collaborators at UMass Medical School with the initial screening and scheduling processes for their fMRI study; (d) conduct the 9-week mindfulness intervention off site in safe, community-based locations; (e) recruit from the partnership at Lifespan Emergency Department; (f) conduct qualitative interviews with low SES population to explore perceptions of and openness to mindfulness-based interventions; and (g) update consent forms with new format. Also includes approval of revised phone screener (SCR-P, v.3.0 – 5/10/2018)
Amendment #12	8/7/18	10/15/18	Stage 2a	Requested the following: 1) permission to conduct abbreviated 1 year follow-up assessment with the intervention arm of the study; 2) request to use a general release form to video-record mindfulness classes; 3) video-recording; 4) removal of the actigraph device; 5) additional questions for the 6 month follow-up assessment for control participants; 6) updated consent document (v.3.1 – 10/11/18); and 7) consent addendum for already enrolled participants.

BROWN UNIVERSITY
INSTITUTIONAL REVIEW BOARD
HUMAN RESEARCH PROTOCOL

Protocol Title:

Principal Investigator:

Funding Source (if no external funding for the project, enter "University"):

(1) Attach to this form the information required for a complete protocol, as outlined on the IRB Form #1 Instructions and Information pages. In addition, please review the document "What Makes a Complete Protocol" (an appendix to the 'Brown University Policies and Procedures for the Protection of Human Participants in Research') at http://research.brown.edu/ about-brown-research/policies/irb-policies-procedures-protection-human-participants-research.

(2) Select the appropriate type (and category number) of review. (See the following pages for a description of the exempt and expedited categories. If no exempt or expedited categories completely describe the proposed research, select 'Full Board'.) ☐ **Exempt #** ☐ **Expedited #** ☒ **Full Board**

(3) **Principal Investigator Conflict of Interest Statement:**
(1) Have you filed the annual Assurance of Compliance form and, if necessary, a disclosure as required by the University's Conflict of Interest Policy for Officers of Instruction and Research (http://research.brown.edu/ about-brown-research/policies/conflict-of-interest-statement)? ☒**YES** ☐**NO**
(2) Do you have a significant financial interest that is related to this research protocol? ☐**YES** ☒**NO**

Principal Investigator certifies to the following: ***(1)*** The rights and welfare of the participants are adequately protected. ***(2)*** The risks to an individual are outweighed by the potential benefits to him/her or by the importance of the knowledge to be gained. ***(3)*** This protocol is accurate and complete; and if the project scope or design is later changed, the PI will resubmit for review. ***(4)*** All research personnel, including the PI, has been, or will be, adequately educated in human research protections prior to beginning work on the project. ***(5)*** Where a financial conflict exists, the PI has disclosed all relevant information regarding Conflict of Interest according to University policy.

Principal Investigator signature: ______________________ **Date:** 12/5/2014

==

(Advisor's signature is required for all graduate/medical student projects.)
Advisor certifies to the following: Advisor has read the protocol and approves of the project.
Advisor's signature: ______________________________________ **Date:** __________
Print name:

==

Undergraduate student investigator signature: ______________________________ **Date:** __________
Print name: (optional signature)

For IRB Use Only
FULL BOARD PROTOCOLS **-** Institutional Review Board Members: If approving the proposed project, please certify to the best of your knowledge to the following: ***(1)*** IRB Member is familiar with the above described proposed research. ***(2)*** The rights and welfare of the research participants will be adequately safeguarded by the procedures described. ***(3)*** The potential benefits justify the risks involved. ***(4)*** IRB Member has no vested interest in the project.
IRB Member Signature: ______________________________________ Date: __________

Signature of the Authorized Official of the IRB: ______________________________ **Date:** ________

IRB Form #1 (Rev. 10/10)

Protocol Checklist and Submission Procedures

Protocol Checklist

All protocols for IRB review (full board, expedited, or exempt) must be submitted in the following format. Protocols that do not follow this required format will be returned to the investigator without IRB review.

#1 ___X__ IRB Form # 1 (fully signed)

#2 ___X__ Required protocol components (pages must be numbered) (see "What Makes a Complete Protocol" for a more detailed description)

#3 ___X__ Consent forms, including assent forms (if applicable) (pages must be numbered)

#4 ___X_ Interview instruments/questionnaires (if applicable) (pages must be numbered)

#5 ___X_ Recruitment documents (such as advertisements, flyers, cards) (if applicable)

#6 _N/A_ Letters of commitment/approval from collaborating organizations (see "What Makes a Complete Protocol" for a more detailed description) (if applicable)

#7 _N/A__ Copies of other IRB approvals (if applicable)

#8 _N/A_ Checklist Form for Research Involving the Use of Prisoners as Study Participants (if applicable)

#9 _N/A__ Complete application for outside funding/support (pages must be numbered)

~~~~~~

Protocol Submission Procedures

Full Board Protocols: Submit the original and 11 copies of the complete protocol (as identified above) to the Research Protections Office, Box 1986 in sufficient time to meet the agenda deadline (see http://research.brown.edu/rschadmin/hrpo_meetingdates.php for upcoming IRB meeting dates and protocol deadlines).

Exempt or Expedited Protocols: There is no specified deadline for submissions of Exempt or Expedited Protocols. Review time varies depending upon the project. The average review time is approximately 4 weeks. Please submit the complete (as identified above) original protocol to the Research Protections Office (RPO), Box 1986, with sufficient time to allow for review and revisions, if necessary.

{Note that the IRB (not the investigator) makes the final determination of whether a protocol is full board, expedited, or exempt. Thus, full board review may be necessary even if you suggest expedited or exempt review in your protocol.}
~~~~~~

MINDFULNESS-BASED HYPERTENSION THERAPY PILOT STUDY

IRB APPLICATION

December 5th, 2014

Eric B. Loucks, PhD
Assistant Professor
Department of Epidemiology
Brown University School of Public Health
121 South Main St., Box G-S121
Providence, RI, 02912
Phone: 401-863-6283
Email: eric.loucks@brown.edu

Section A:
Lay-Person Summary of Proposed Project

1 Specific Aims

1. To formally develop a Mindfulness-Based Hypertension Therapy (MBHT) intervention tailored to the needs for hypertensive patients, utilizing a multi-disciplinary team of experts and patients, and incorporating a Delphi-like technique to adapt protocols of a standardized mindfulness-based intervention developed at UMass Medical School for patients with prehypertension or hypertension.
2. To determine the optimal dose of the intervention developed in aim 1. We will estimate the feasibility, acceptability and safety of two different doses of the MBHT intervention in 60 patients (30 per dose arm) randomly assigned to a "low" or "high" dose of MBHT.
3. To finalize the protocol of the MBHT intervention and design of the future RCT. We will use the results of our developmental work (aim 1) and of the dosing study (aim 2) to make the final adjustments to the protocol of the intervention that will be tested for efficacy in our future RCT, incorporating the expertise of our interdisciplinary panel to optimize the design of the future RCT.

2 Significance and Innovation

The World Health Organization reported that suboptimal BP is responsible for over half of cardiovascular disease mortality world-wide.[1,2] Furthermore, of those with hypertension, more than half have uncontrolled BP.[3,4] This is despite widespread knowledge of hypertension risk factors and treatments including diet, obesity, physical activity, medication adherence and excessive alcohol consumption.[5,6] Mindfulness interventions appear to improve self-regulation, self-care, and the ability to not respond to cravings for hypertension risk factors such as high caloric palatable foods and sedentary activities (and resulting obesity).[7-11] *In fact, a 2009 Institute of Medicine report recommended prioritizing research to "Compare the effectiveness of mindfulness-based interventions (e.g. yoga, meditation, deep breathing training) and usual care in treating… cardiovascular risk factors."*[12] Evidence-based mindfulness interventions including Mindfulness-Based Stress Reduction (MBSR) may have some effects, where a recent meta-analysis and systematic review of 4 randomized controlled trials (RCTs) demonstrated significant effects, but evidence of heterogeneity in effect sizes.[13] The methodologically highest quality studies had the smallest effect sizes. MBSR has been customized to a number of disease processes, such as recurrent depression, smoking and substance use addiction with resulting increases in effect sizes compared to general mindfulness-based interventions that do not take the disease process of interest into consideration.[10,14,15] *The same may be true for hypertension, however this has not yet been studied.*

Until methodologically rigorous studies to evaluate customized mindfulness-based interventions for hypertension are performed, we will not know if the observed preliminary effects of mindfulness-based interventions on BP could have much greater effect with a tailored approach. Consequently, we propose to conduct a stage I behavioral therapy intervention study to provide the most rigorous evaluation to date on whether MBSR customized to prehypertensive and hypertensive patients provides clinically relevant reductions in BP.

3 Approach

3.1 Initial Development of High and Low-Dose MBHT (Aim 1)

We will assemble a team of experts in cardiology (e.g. Dudley, Wu, Shimbo), family medicine (e.g. Eaton), physical activity (e.g. Williams, Davidson), diet (e.g. McCaffery), medication adherence (e.g. Kronish), mindfulness interventions (e.g. Britton, Salmoirago-Blotcher, Brewer), and patients with prehypertension or hypertension. With this team, we use an iterative, Delphi-like technique to adapt protocols of a standardized mindfulness-based intervention developed at UMass Medical School, called MBSR, for patients with prehypertension or hypertension, to the unique safety and treatment needs of this population. Adaptions may include, for example, types of specific mindfulness exercises, sequence in which they are introduced, and relative emphasis on hypertension education *vs.* mindfulness practice components.

3.2 Study Population (Aim 2)

Participants will be recruited through cardiology and family practices via established relationships with physicians at Rhode Island Hospital (e.g. Sam Dudley, MD), Memorial Hospital of Rhode Island (e.g. Charles

Eaton, MD), Providence VA Medical Center (e.g. Hank Wu, MD), amongst others. Furthermore, advertisements will be posted throughout Rhode Island inviting participants with pre-hypertension or hypertension to enroll. For Aim 2, we anticipate n=60 participants with equal proportions of males and females, and stratified across prehypertension/hypertension categories, including (1) pre-hypertension, (2) controlled hypertension, and (3) uncontrolled hypertension. This will allow for early indications of potential differential effects of MBHT by gender and prehypertension/hypertension status.

Inclusion Criterion: Hypertension/prehypertension (≥120 mmHg systolic, ≥80 mmHg diastolic pressure or taking antihypertensive medication).

Exclusion Criteria: Exclusion criteria follow standard guidelines:[16] current regular meditation practice (>once/week); current substance abuse, suicidal ideation or eating disorder; history of bipolar or psychotic disorders or self-injurious behaviors.

3.3 MBHT Intervention Groups

High Dose: MBHT is based on the standardized MBSR intervention described elsewhere,[17-20] and will likely consist of nine 2.5-hour weekly group sessions and an 8-hour one-day session, led by Dr. Loucks. Dr. Loucks is an expert in cardiovascular physiology, cardiovascular epidemiology, and social epidemiology. He completed MBSR instructor training at UMass Medical School, and has 16 years of mindfulness meditation experience, including teaching and mentoring community members, as well as a recent publication record investigating associations of mindfulness with cardiovascular health.[11,21,22] The unique areas of MBHT are expected to be education on hypertension risk factors, hypertension health effects, and specific mindfulness modules focused on awareness of diet, physical activity, medication adherence, alcohol consumption, stress, and social support for behavior change. A preliminary manual has been created based on the standardized MBSR manual developed at UMass Medical School, and will be further developed through the Delphi-like approach described above, and sequentially revised based on participant feedback and preliminary findings.[19,20] MBHT sessions contain instruction and practice in mindfulness meditation, and conversations about stress and coping. Students learn a range of mindfulness skills including body scan exercises, meditation and yoga. Homework consists of practicing skills for ≥45 min/day, 6 days/week. Booster sessions will be offered (likely 2.5-hour sessions every 2 months).

Low Dose: Low Dose MBHT will be based on the High Dose intervention above, but instead prioritizing practices to fit within nine 1-hour weekly groups sessions. Through the aforementioned Delphi-like approach, the most salient parts of the high dose intervention described above will be offered in low dose MBHT.

3.4 Measures of Compliance

Participant Adherence: Data on treatment protocol adherence will be collected weekly by research staff.

MBHT Instructor Competency and Treatment Fidelity: Treatment fidelity strategies will be performed in accordance with recommendations of the NIH Behavior Change consortium.[23]

3.5 Blinding

All study staff, including those performing health assessments and contacting participants to schedule follow-up assessments, will be blinded to treatment allocation with the exception of the MBHT instructor (Loucks) and data manager (who performs randomization).

3.6 Equipoise

This study will be designed to disrupt clinical equipoise, defined as “no consensus within the expert clinical community about the comparative merits of the alternative [trial arms] to be tested.”[24]

3.7 Assessments

We will follow study procedures similar to those performed during previous epidemiologic studies by our group. In-person screenings will take place at the Brown University Center for Population Health and Clinical Epidemiology, described in Sections 2.7.1 and 2.7.2 at baseline, completion of the 9-week MBHT course and at 3- and 6-months post-intervention. Major assessment variables are shown in **Table 1.** Qualitative interviews (likely focus groups) will evaluate participants’ experiences such as usefulness of specific modules, duration of sessions, time burden, and overall intervention effectiveness.

3.7.1 Primary Outcomes

Clinic BP Assessment: Clinic-assessed systolic and diastolic BP remains the standard of care for monitoring hypertension treatment.[3,5,6,25,26] Baseline clinic BP will be determined at two in-person screening visits ≥1 week apart, completed according to American Heart Association guidelines,[25] followed by in-person assessments at all follow-up periods. BP will be assessed using a calibrated Omron HEM907XL Intellisense automated BP monitor with established validity.[27]

Table 1. Examples of variables proposed to be assessed during MBHT study

Variables	Obtained During Study			
	Baseline	8 w	3 m	6 m
Race/ethnicity	✓			
Gender	✓			
Adverse childhood experiences	✓			
Socioeconomic status	✓	✓	✓	✓
Depressive symptomatology	✓	✓	✓	✓
Anxiety	✓	✓	✓	✓
Perceived stress	✓	✓	✓	✓
Self efficacy	✓	✓	✓	✓
Mindfulness	✓	✓	✓	✓
Blood pressure	✓	✓		✓
Smoking	✓	✓	✓	✓
Diet	✓	✓	✓	✓
Physical activity	✓	✓	✓	✓
Alcohol consumption	✓	✓	✓	✓
Body mass index	✓	✓		✓
Sleep quantity and quality	✓	✓	✓	✓
Social support	✓	✓	✓	✓
Loneliness	✓	✓	✓	✓
Medication use and adherence	✓	✓	✓	✓

m, months; w,weeks

3.7.2 Potential Mediators

Examples of mediators to be assessed include: (1) Body Mass Index: height and weight directly assessed using standard epidemiologic methods. *(2) Physical activity:* MET minutes per week, assessed using actigraphy, which enables the quantification of physical activity.[28-32] *(3) Diet*: diet assessed utilizing Dietary Approaches to Stop Hypertension (DASH) eating pattern score,[33] measured via diet history food frequency questionnaire.[34] *(4) Perceived stress:* measured using Perceived Stress Scale.[35] *(5) Antihypertensive Medication Adherence:* measured continuously using electronic medication bottle caps (GlowCaps).

3.7.3 Potential Effect Modifiers

We propose to evaluate 2 potentially important effect modifiers: (1) gender, (2) prehypertension/hypertension category.

3.8 Intervention Allocation

Stratified randomization will be used.[36,37] Variables used to create strata include gender (male *vs.* female), race/ethnicity (white *vs.* non-white), and prehypertension/ hypertension status (pre-hypertension, controlled hypertension, uncontrolled hypertension). Simple random sampling will occur within each strata.

3.9 Data Analysis

Qualitative analyses will evaluate participant experiences such as usefulness of specific modules, duration of sessions, time burden, and overall intervention effectiveness. Quantitative analyses will incorporate generalized linear models (GLM) with properly chosen link functions, performed using generalized estimating equations (GEE) with robust standard error estimators.[38,39] We will test effects of high *vs.* low dose MBHT on clinic-assessed systolic and diastolic BP. Following “intention-to-treat” principles, analyses will be conducted on all participants randomized to high *vs*. low dose, regardless of intervention completion. To test whether effects of MBHT on BP may be effect modified by variables described in Section 2.7.3 we will add product terms between MBHT and each potential effect modifier to models described above. Formal statistical significance tests of product terms will be performed. Analyses are considered statistically underpowered, but will provide effect size estimates and statistical variances for NIH grant applications to support studies with larger sample sizes.

References

1. Mendis S, Puska P, Norrving B. *Global atlas on cardiovascular disease prevention and control.* Geneva: World Health Organization;2011.
2. Hoyert DL, Xu J. Deaths: Preliminary Data for 2011. *National Vital Statistics Reports.* 2012;61(6):1-65.
3. Chobanian AV, Bakris GL, Black HR, et al. Seventh report of the Joint National Committee on Prevention, Detection, Evaluation, and Treatment of High Blood Pressure. *Hypertension.* Dec 2003;42(6):1206-1252.
4. United States Centers for Disease Control and Prevention. Getting blood pressure under control. *CDC Vitalsigns.* 2012;September.
5. James PA, Oparil S, Carter BL, et al. 2014 evidence-based guideline for the management of high blood pressure in adults: report from the panel members appointed to the Eighth Joint National Committee (JNC 8). *JAMA.* Feb 5 2014;311(5):507-520.
6. Weber MA, Schiffrin EL, White W, et al. Clinical practice guidelines for the management of hypertension in the community: A statement by the American Society of Hypertension and the International Society of Hypertension. *J Hypertens.* 2014;32(1):3-15.
7. Epstein RM. Mindful practice. *JAMA.* Sep 1 1999;282(9):833-839.
8. Sedlmeier P, Eberth J, Schwarz M, et al. The psychological effects of meditation: a meta-analysis. *Psychol Bull.* Nov 2012;138(6):1139-1171.
9. Lutz A, Slagter HA, Dunne JD, Davidson RJ. Cognitive-emotional interactions - Attention regulation and monitoring in meditation. *Trends Cogn Sci.* Apr 2008;12(4):163-169.
10. Brewer JA, Mallik S, Babuscio TA, et al. Mindfulness training for smoking cessation: results from a randomized controlled trial. *Drug Alc Depend.* 2011;119(1-2):72-80.
11. Loucks EB, Britton WB, Howe CJ, Eaton CB, Buka SL. Positive associations of dispositional mindfulness with cardiovascular health: The New England Family Study. *Int J Behav Med.* 2014: DOI 10.1007/s12529-12014-19448-12529.
12. Institute of Medicine. *Initial National Priorities for Comparative Effectiveness Research.* Washington, D.C. 2009.
13. Abbott RA, Whear R, Rodgers LR, et al. Effectiveness of mindfulness-based stress reduction and mindfulness based cognitive therapy in vascular disease: A systematic review and meta-analysis of randomised controlled trials. *J Psychosom Res.* May 2014;76(5):341-351.
14. Goyal M, Singh S, Sibinga EM, et al. Meditation programs for psychological stress and well-being: A systematic review and meta-analysis. *JAMA Intern Med.* Mar 1 2014;174(3):357-368.
15. Bowen S, Witkiewitz K, Clifasefi SL, et al. Relative Efficacy of Mindfulness-Based Relapse Prevention, Standard Relapse Prevention, and Treatment as Usual for Substance Use Disorders: A Randomized Clinical Trial. *JAMA Psychiatr.* Mar 19 2014.
16. Santorelli SF, Kabat-Zinn J. *MBSR Curriculum Guide and Supporting Materials. Mindfulness Based Stress Reduction Professional Training.* Worcester, MA: University of Massachusetts Center for Mindfulness; 2003.
17. Kabat-Zinn J. An outpatient program in behavioral medicine for chronic pain patients based on the practice of mindfulness meditation: theoretical considerations and preliminary results. *Gen Hosp Psychiatry.* Apr 1982;4(1):33-47.
18. Kabat-Zinn J, Massion AO, Kristeller J, et al. Effectiveness of a meditation-based stress reduction program in the treatment of anxiety disorders. *Am J Psychiatry.* Jul 1992;149(7):936-943.
19. Santorelli S, Bonus K, McCallum C, et al. Mindfulness-Based Stress Reduction: Guidelines and Standards of Practice. *Integrative Medicine Mindfulness Program at University of Wisconsin- Madison and the Center for Mindfulness, University of Massachusetts.* 2004.
20. MacCoon DG, Imel ZE, Rosenkranz MA, et al. The validation of an active control intervention for Mindfulness Based Stress Reduction (MBSR). *Behav Res Ther.* Jan 2012;50(1):3-12.
21. Loucks EB. Meditation intervention reviews: selecting ideal control groups for meditation interventions. *JAMA Intern Med.* Jul 2014;174(7):1194-1195.
22. Loucks EB, Britton WB, Howe CJ, et al. Associations of dispositional mindfulness with obesity and central adiposity: The New England Family Study. Under Review.
23. Bellg AJ, Borrelli B, Resnick B, et al. Enhancing treatment fidelity in health behavior change studies: best practices and recommendations from the NIH Behavior Change Consortium. *Health Psychol.* Sep 2004;23(5):443-451.

24. Freedman B. Equipoise and the ethics of clinical research. *N Engl J Med.* Jul 16 1987;317(3):141-145.
25. Pickering TG, Hall JE, Appel LJ, et al. Recommendations for blood pressure measurement in humans and experimental animals: part 1: blood pressure measurement in humans: a statement for professionals from the Subcommittee of Professional and Public Education of the American Heart Association Council on High Blood Pressure Research. *Circulation.* Feb 8 2005;111(5):697-716.
26. Williams B, Lindholm LH, Sever P. Systolic pressure is all that matters. *Lancet.* Jun 28 2008;371(9631):2219-2221.
27. El Assaad MA, Topouchian JA, Darne BM, Asmar RG. Validation of the Omron HEM-907 device for blood pressure measurement. *Blood Press Monit.* Aug 2002;7(4):237-241.
28. Chen KY, Acra SA, Majchrzak K, et al. Predicting energy expenditure of physical activity using hip- and wrist-worn accelerometers. *Diabet Technol Therapeut.* 2003;5(6):1023-1033.
29. Crouter SE, Clowers KG, Bassett DR, Jr. A novel method for using accelerometer data to predict energy expenditure. *J Appl Physiol (1985).* Apr 2006;100(4):1324-1331.
30. Troiano RP. Translating accelerometer counts into energy expenditure: advancing the quest. *J Appl Physiol (1985).* Apr 2006;100(4):1107-1108.
31. Esliger DW, Tremblay MS. Technical reliability assessment of three accelerometer models in a mechanical setup. *Med Sci Sports Exerc.* Dec 2006;38(12):2173-2181.
32. Heil DP. Predicting activity energy expenditure using the Actical activity monitor. *Res Quart Exercise Sport.* Mar 2006;77(1):64-80.
33. Roger VL, Go AS, Lloyd-Jones DM, et al. Heart disease and stroke statistics--2012 update: a report from the American Heart Association. *Circulation.* Jan 3 2012;125(1):e2-e220.
34. Subar AF, Thompson FE, Kipnis V, et al. Comparative validation of the Block, Willett, and National Cancer Institute food frequency questionnaires: The Eating at America's Table Study. *Am J Epidemiol.* Dec 15 2001;154(12):1089-1099.
35. Cohen S, Kamarck T, Mermelstein R. A global measure of perceived stress. *J Health Soc Behav.* Dec 1983;24(4):385-396.
36. Kernan WN, Viscoli CM, Makuch RW, Brass LM, Horwitz RI. Stratified randomization for clinical trials. *J Clin Epidemiol.* 1999;52(1):19-26.
37. Friedman LM, Furberg CD, DeMets DL. *Fundamentals of Clinical Trials.* New York, NY: Springer; 2010.
38. Liang K-Y, Zeger SL. Longitudinal data analysis using generalized linear models. *Biometrika.* 1986;73(1):13-22.
39. Zeger SL, Liang K-Y. Longitudinal data analysis for discrete and continuous outcomes. *Biometrics.* 1986;42:121-130.

Section B:

Application for Funding

An Application for the Brown internal Richard B. Salomon Faculty Research Award has been made.

An NIH R34 is planned to be submitted in March, 2015.

Section C:
Specific Aims and Methodology

1 Significance and Innovation

The World Health Organization reported that suboptimal BP is responsible for over half of cardiovascular disease mortality world-wide.[1,2] Furthermore, of those with hypertension, more than half have uncontrolled BP.[3,4] This is despite widespread knowledge of hypertension risk factors and treatments including diet, obesity, physical activity, medication adherence and excessive alcohol consumption.[5,6] Mindfulness interventions appear to improve self-regulation, self-care, and the ability to not respond to cravings for hypertension risk factors such as high caloric palatable foods and sedentary activities (and resulting obesity).[7-11] *In fact, a 2009 Institute of Medicine report recommended prioritizing research to "Compare the effectiveness of mindfulness-based interventions (e.g. yoga, meditation, deep breathing training) and usual care in treating… cardiovascular risk factors."*[12] Evidence-based mindfulness interventions including Mindfulness-Based Stress Reduction (MBSR) may have some effects, where a recent meta-analysis and systematic review of 4 randomized controlled trials (RCTs) demonstrated significant effects, but evidence of heterogeneity in effect sizes.[13] The methodologically highest quality studies had the smallest effect sizes. MBSR has been customized to a number of disease processes, such as recurrent depression, smoking and substance use addiction with resulting increases in effect sizes compared to general mindfulness-based interventions that do not take the disease process of interest into consideration.[10,14,15] *The same may be true for hypertension, however this has not yet been studied.*

Until methodologically rigorous studies to evaluate customized mindfulness-based interventions for hypertension are performed, we will not know if the observed preliminary effects of mindfulness-based interventions on BP could have much greater effect with a tailored approach. Consequently, we propose to conduct a stage I behavioral therapy intervention study to provide the most rigorous evaluation to date on whether MBSR customized to prehypertensive and hypertensive patients provides clinically relevant reductions in BP. These preliminary findings will set the stage for NIH applications, including R34 and R01 applications.

2 Specific Aims

1. To formally develop a Mindfulness-Based Hypertension Therapy (MBHT) intervention tailored to the needs for hypertensive patients, utilizing a multi-disciplinary team of experts and patients, and incorporating a Delphi-like technique to adapt protocols of a standardized mindfulness-based intervention developed at UMass Medical School for patients with prehypertension or hypertension.
2. To determine the optimal dose of the intervention developed in aim 1. We will estimate the feasibility, acceptability and safety of two different doses of the MBHT intervention in 60 patients (30 per dose arm) randomly assigned to a "low" or "high" dose of MBHT.
3. To finalize the protocol of the MBHT intervention and design of the future RCT. We will use the results of our developmental work (aim 1) and of the dosing study (aim 2) to make the final adjustments to the protocol of the intervention that will be tested for efficacy in our future RCT, incorporating the expertise of our interdisciplinary panel to optimize the design of the future RCT.

4 Approach

4.1 Initial Development of High and Low-Dose MBHT (Aim 1)

We will assemble a team of experts in cardiology (e.g. Dudley, Wu, Shimbo), family medicine (e.g. Eaton), physical activity (e.g. Williams, Davidson), diet (e.g. McCaffery), medication adherence (e.g. Kronish), mindfulness interventions (e.g. Britton, Salmoirago-Blotcher, Brewer), and patients with prehypertension or hypertension. With this team, we use an iterative, Delphi-like technique to adapt protocols of a standardized mindfulness-based intervention developed at UMass Medical School, called MBSR, for patients with prehypertension or hypertension, to the unique safety and treatment needs of this population. Adaptions may include, for example, types of specific mindfulness exercises, sequence in which they are introduced, and relative emphasis on hypertension education *vs.* mindfulness practice components.

4.2 Study Population (Aim 2)

Participants will be recruited through cardiology and family practices via established relationships with physicians at Rhode Island Hospital (e.g. Sam Dudley, MD), Memorial Hospital of Rhode Island (e.g. Charles Eaton, MD), Providence VA Medical Center (e.g. Hank Wu, MD), amongst others. Furthermore, advertisements will be posted throughout Rhode Island inviting participants with pre-hypertension or hypertension to enroll. For Aim 2, we anticipate n=60 participants with equal proportions of males and females, and stratified across prehypertension/hypertension categories, including (1) pre-hypertension, (2) controlled hypertension, and (3) uncontrolled hypertension. This will allow for early indications of potential differential effects of MBHT by gender and prehypertension/hypertension status.

Inclusion Criterion: Hypertension/prehypertension (≥120 mmHg systolic, ≥80 mmHg diastolic pressure or taking antihypertensive medication). All genders and racial/ethnic groups are eligible to be included.

Exclusion Criteria: Exclusion criteria follow standard guidelines and recommendations:[1] (a) current regular meditation practice (>once/week); (b) serious medical illness precluding regular class attendance; (c) current substance abuse, suicidal ideation or eating disorder, (d) history of bipolar or psychotic disorders or self-injurious behaviors. These participants are excluded because they may disrupt group participation, require additional or specialized treatment, or are already participating in practices similar to the intervention.

4.3 Confidentiality of Data

The clinical data will be de-identified but linked. Private information such as name, date of birth, and address for recontacting will be kept in a password protected, encrypted database on a different disk that the clinical data. Only the principal investigator for the purposes of patient safety or monitoring by the NIH, data safety monitoring boards or HIPPA compliance officer approved agents will be given access to identifiable personal information.

Data which contains person-identifiers is considered highly sensitive. Such data is, by policy, only stored within the VMScluster at the Brown University Center for Population Health and Clinical Epidemiology. Access is controlled by the VMS ACL mechanism, allowing read-only access to a small group of specific users and read-write access to an even smaller group. Internet access (e.g., via a Web server) is disallowed for such storage areas. Windows security controls are superceded by VMS controls, although Windows access is permitted since some data is acquired on Windows-specific media (e.g., CD-ROM). All uses of privileged necessary to grant/revoke access to any data, as well as any other security-relevant event data, is continuously logged and analyzed in the context of both the VMScluster and the Windows Domain. Network access is tightly controlled to allow anonymous access only to a small amount of read-only data; all "risky protocols" are disallowed from Internet access. For any given study with person-identifying information, such data is stripped before the data is made accessible to staff not authorized to access such identifiers. In those cases where the data is longitudinal, a non-reversible encryption of the person ID is made in order to provide a unique person ID not traceable to the underlying person. The specific algorithm used for any given set of data is stored in the same fashion and given the same security as the data itself. To make data use simpler, hierarchical "trees" of directories are given consistent access controls tied to a unique identifier, which is then granted to staff authorized to use such data. Programming staff authorized to make individual-level data available to staff not authorized to access the person-identifying components routinely copy the "encrypted" versions from the more-secured tree to a less-secured tree. Security controls are thus relatively automatic, based on the propagation of rights based on the location of a file within the file system and the identity of the user attempting to access it.

4.4 MBHT Intervention Groups

High Dose: MBHT is based on the standardized MBSR intervention described elsewhere,[17-20] and will likely consist of nine 2.5-hour weekly group sessions and an 8-hour one-day session, led by Dr. Loucks. Dr. Loucks is an expert in cardiovascular physiology, cardiovascular epidemiology, and social epidemiology. He completed MBSR instructor training at UMass Medical School, and has 16 years of

mindfulness meditation experience, including teaching and mentoring community members, as well as a recent publication record investigating associations of mindfulness with cardiovascular health.[11,21,22] The unique areas of MBHT are expected to be education on hypertension risk factors, hypertension health effects, and specific mindfulness modules focused on awareness of diet, physical activity, medication adherence, alcohol consumption, stress, and social support for behavior change. A preliminary manual has been created based on the standardized MBSR manual developed at UMass Medical School, and will be further developed through the Delphi-like approach described above, and sequentially revised based on participant feedback and preliminary findings.[19,20] MBHT sessions contain instruction and practice in mindfulness meditation, and conversations about stress and coping. Students learn a range of mindfulness skills including body scan exercises, meditation and yoga. Homework consists of practicing skills for ≥45 min/day, 6 days/week. Booster sessions will be offered (likely 2.5-hour sessions every 2 months).

Low Dose: Low Dose MBHT will be based on the High Dose intervention above, but instead prioritizing practices to fit within nine 1-hour weekly groups sessions. Through the aforementioned Delphi-like approach, the most salient parts of the high dose intervention described above will be offered in low dose MBHT.

4.5 Measures of Compliance

Participant Adherence: Data on treatment protocol adherence will be collected weekly by research staff.

MBHT Instructor Competency and Treatment Fidelity: Treatment fidelity strategies will be performed in accordance with recommendations of the NIH Behavior Change consortium.[23]

4.6 Blinding

All study staff, including those performing health assessments and contacting participants to schedule follow-up assessments, will be blinded to treatment allocation with the exception of the MBHT instructor (Loucks) and data manager (who performs randomization).

4.7 Equipoise

This study will be designed to disrupt clinical equipoise, defined as “no consensus within the expert clinical community about the comparative merits of the alternative [trial arms] to be tested.”[24]

4.8 Assessments

We will follow study procedures similar to those performed during previous epidemiologic studies by our group. In-person screenings will take place at the Brown University Center for Population Health and Clinical Epidemiology, described in Sections 2.7.1 and 2.7.2 at baseline, completion of the 9-week MBHT course and at 3- and 6-months post-intervention. Major assessment variables are shown in **Table 1.** Qualitative interviews (likely focus groups) will evaluate participants’ experiences such as usefulness of specific modules, duration of sessions, time burden, and overall intervention effectiveness.

Table 1. Examples of variables proposed to be assessed during MBHT study

	Obtained During Study			
Variables	Baseline	8 w	3 m	6 m
Race/ethnicity	✓			
Gender	✓			
Adverse childhood experiences	✓			
Socioeconomic status	✓	✓	✓	✓
Depressive symptomatology	✓	✓	✓	✓
Anxiety	✓	✓	✓	✓
Perceived stress	✓	✓	✓	✓
Self efficacy	✓	✓	✓	✓
Mindfulness	✓	✓	✓	✓
Blood pressure	✓	✓		✓
Smoking	✓	✓	✓	✓
Diet	✓	✓	✓	✓
Physical activity	✓	✓	✓	✓
Alcohol consumption	✓	✓	✓	✓
Body mass index	✓	✓		✓
Sleep quantity and quality	✓	✓	✓	✓
Social support	✓	✓	✓	✓
Loneliness	✓	✓	✓	✓
Medication use and adherence	✓	✓	✓	✓

m, months; w,weeks

4.8.1 Primary Outcomes

Clinic BP Assessment: Clinic-assessed systolic and diastolic BP remains the standard of care for monitoring hypertension treatment.[3,5,6,25,26] Baseline clinic BP will be

determined at two in-person screening visits ≥1 week apart, completed according to American Heart Association guidelines,[25] followed by in-person assessments at all follow-up periods. BP will be assessed using a calibrated Omron HEM907XL Intellisense automated BP monitor with established validity.[27]

4.8.2 Potential Mediators

Examples of mediators to be assessed include: (1) Body Mass Index: height and weight directly assessed using standard epidemiologic methods. *(2) Physical activity:* MET minutes per week, assessed using actigraphy, which enables the quantification of physical activity.[28-32] *(3) Diet*: diet assessed utilizing Dietary Approaches to Stop Hypertension (DASH) eating pattern score,[33] measured via diet history food frequency questionnaire.[34] *(4) Perceived stress:* measured using Perceived Stress Scale.[35] *(5) Antihypertensive Medication Adherence:* measured continuously using electronic medication bottle caps (GlowCaps).

4.8.3 Potential Effect Modifiers

We propose to evaluate 2 potentially important effect modifiers: (1) gender, (2) prehypertension/hypertension category.

4.9 Intervention Allocation

Stratified randomization will be used.[36,37] Variables used to create strata include gender (male *vs.* female), race/ethnicity (white *vs.* non-white), and prehypertension/ hypertension status (pre-hypertension, controlled hypertension, uncontrolled hypertension). Simple random sampling will occur within each strata.

4.10 Data Analysis

Qualitative analyses will evaluate participant experiences such as usefulness of specific modules, duration of sessions, time burden, and overall intervention effectiveness. Quantitative analyses will incorporate generalized linear models (GLM) with properly chosen link functions, performed using generalized estimating equations (GEE) with robust standard error estimators.[38,39] We will test effects of high *vs.* low dose MBHT on clinic-assessed systolic and diastolic BP. Following “intention-to-treat” principles, analyses will be conducted on all participants randomized to high *vs*. low dose, regardless of intervention completion. To test whether effects of MBHT on BP may be effect modified by variables described in Section 2.7.3 we will add product terms between MBHT and each potential effect modifier to models described above. Formal statistical significance tests of product terms will be performed. Analyses are considered statistically underpowered, but will provide effect size estimates and statistical variances for NIH grant applications to support studies with larger sample sizes.

References

1. Mendis S, Puska P, Norrving B. *Global atlas on cardiovascular disease prevention and control.* Geneva: World Health Organization;2011.
2. Hoyert DL, Xu J. Deaths: Preliminary Data for 2011. *National Vital Statistics Reports.* 2012;61(6):1-65.
3. Chobanian AV, Bakris GL, Black HR, et al. Seventh report of the Joint National Committee on Prevention, Detection, Evaluation, and Treatment of High Blood Pressure. *Hypertension.* Dec 2003;42(6):1206-1252.
4. United States Centers for Disease Control and Prevention. Getting blood pressure under control. *CDC Vitalsigns.* 2012;September.
5. James PA, Oparil S, Carter BL, et al. 2014 evidence-based guideline for the management of high blood pressure in adults: report from the panel members appointed to the Eighth Joint National Committee (JNC 8). *JAMA.* Feb 5 2014;311(5):507-520.
6. Weber MA, Schiffrin EL, White W, et al. Clinical practice guidelines for the management of hypertension in the community: A statement by the American Society of Hypertension and the International Society of Hypertension. *J Hypertens.* 2014;32(1):3-15.
7. Epstein RM. Mindful practice. *JAMA.* Sep 1 1999;282(9):833-839.
8. Sedlmeier P, Eberth J, Schwarz M, et al. The psychological effects of meditation: a meta-analysis. *Psychol Bull.* Nov 2012;138(6):1139-1171.
9. Lutz A, Slagter HA, Dunne JD, Davidson RJ. Cognitive-emotional interactions - Attention regulation and monitoring in meditation. *Trends Cogn Sci.* Apr 2008;12(4):163-169.
10. Brewer JA, Mallik S, Babuscio TA, et al. Mindfulness training for smoking cessation: results from a randomized controlled trial. *Drug Alc Depend.* 2011;119(1-2):72-80.
11. Loucks EB, Britton WB, Howe CJ, Eaton CB, Buka SL. Positive associations of dispositional mindfulness with cardiovascular health: The New England Family Study. *Int J Behav Med.* 2014: DOI 10.1007/s12529-12014-19448-12529.
12. Institute of Medicine. *Initial National Priorities for Comparative Effectiveness Research.* Washington, D.C. 2009.
13. Abbott RA, Whear R, Rodgers LR, et al. Effectiveness of mindfulness-based stress reduction and mindfulness based cognitive therapy in vascular disease: A systematic review and meta-analysis of randomised controlled trials. *J Psychosom Res.* May 2014;76(5):341-351.
14. Goyal M, Singh S, Sibinga EM, et al. Meditation programs for psychological stress and well-being: A systematic review and meta-analysis. *JAMA Intern Med.* Mar 1 2014;174(3):357-368.
15. Bowen S, Witkiewitz K, Clifasefi SL, et al. Relative Efficacy of Mindfulness-Based Relapse Prevention, Standard Relapse Prevention, and Treatment as Usual for Substance Use Disorders: A Randomized Clinical Trial. *JAMA Psychiatr.* Mar 19 2014.
16. Santorelli SF, Kabat-Zinn J. *MBSR Curriculum Guide and Supporting Materials. Mindfulness Based Stress Reduction Professional Training.* Worcester, MA: University of Massachusetts Center for Mindfulness; 2003.
17. Kabat-Zinn J. An outpatient program in behavioral medicine for chronic pain patients based on the practice of mindfulness meditation: theoretical considerations and preliminary results. *Gen Hosp Psychiatry.* Apr 1982;4(1):33-47.
18. Kabat-Zinn J, Massion AO, Kristeller J, et al. Effectiveness of a meditation-based stress reduction program in the treatment of anxiety disorders. *Am J Psychiatry.* Jul 1992;149(7):936-943.
19. Santorelli S, Bonus K, McCallum C, et al. Mindfulness-Based Stress Reduction: Guidelines and Standards of Practice. *Integrative Medicine Mindfulness Program at University of Wisconsin-Madison and the Center for Mindfulness, University of Massachusetts.* 2004.
20. MacCoon DG, Imel ZE, Rosenkranz MA, et al. The validation of an active control intervention for Mindfulness Based Stress Reduction (MBSR). *Behav Res Ther.* Jan 2012;50(1):3-12.
21. Loucks EB. Meditation intervention reviews: selecting ideal control groups for meditation interventions. *JAMA Intern Med.* Jul 2014;174(7):1194-1195.
22. Loucks EB, Britton WB, Howe CJ, et al. Associations of dispositional mindfulness with obesity and central adiposity: The New England Family Study. Under Review.

23. Bellg AJ, Borrelli B, Resnick B, et al. Enhancing treatment fidelity in health behavior change studies: best practices and recommendations from the NIH Behavior Change Consortium. *Health Psychol.* Sep 2004;23(5):443-451.
24. Freedman B. Equipoise and the ethics of clinical research. *N Engl J Med.* Jul 16 1987;317(3):141-145.
25. Pickering TG, Hall JE, Appel LJ, et al. Recommendations for blood pressure measurement in humans and experimental animals: part 1: blood pressure measurement in humans: a statement for professionals from the Subcommittee of Professional and Public Education of the American Heart Association Council on High Blood Pressure Research. *Circulation.* Feb 8 2005;111(5):697-716.
26. Williams B, Lindholm LH, Sever P. Systolic pressure is all that matters. *Lancet.* Jun 28 2008;371(9631):2219-2221.
27. El Assaad MA, Topouchian JA, Darne BM, Asmar RG. Validation of the Omron HEM-907 device for blood pressure measurement. *Blood Press Monit.* Aug 2002;7(4):237-241.
28. Chen KY, Acra SA, Majchrzak K, et al. Predicting energy expenditure of physical activity using hip- and wrist-worn accelerometers. *Diabet Technol Therapeut.* 2003;5(6):1023-1033.
29. Crouter SE, Clowers KG, Bassett DR, Jr. A novel method for using accelerometer data to predict energy expenditure. *J Appl Physiol (1985).* Apr 2006;100(4):1324-1331.
30. Troiano RP. Translating accelerometer counts into energy expenditure: advancing the quest. *J Appl Physiol (1985).* Apr 2006;100(4):1107-1108.
31. Esliger DW, Tremblay MS. Technical reliability assessment of three accelerometer models in a mechanical setup. *Med Sci Sports Exerc.* Dec 2006;38(12):2173-2181.
32. Heil DP. Predicting activity energy expenditure using the Actical activity monitor. *Res Quart Exercise Sport.* Mar 2006;77(1):64-80.
33. Roger VL, Go AS, Lloyd-Jones DM, et al. Heart disease and stroke statistics--2012 update: a report from the American Heart Association. *Circulation.* Jan 3 2012;125(1):e2-e220.
34. Subar AF, Thompson FE, Kipnis V, et al. Comparative validation of the Block, Willett, and National Cancer Institute food frequency questionnaires: The Eating at America's Table Study. *Am J Epidemiol.* Dec 15 2001;154(12):1089-1099.
35. Cohen S, Kamarck T, Mermelstein R. A global measure of perceived stress. *J Health Soc Behav.* Dec 1983;24(4):385-396.
36. Kernan WN, Viscoli CM, Makuch RW, Brass LM, Horwitz RI. Stratified randomization for clinical trials. *J Clin Epidemiol.* 1999;52(1):19-26.
37. Friedman LM, Furberg CD, DeMets DL. *Fundamentals of Clinical Trials.* New York, NY: Springer; 2010.
38. Liang K-Y, Zeger SL. Longitudinal data analysis using generalized linear models. *Biometrika.* 1986;73(1):13-22.
39. Zeger SL, Liang K-Y. Longitudinal data analysis for discrete and continuous outcomes. *Biometrics.* 1986;42:121-130.

Section D:
Possible Risks to Participants

The following is a statement from NCCAM about the potential risks of meditation practice: "Meditation is considered to be safe for healthy people. There have been rare reports that meditation could cause or worsen symptoms in people who have certain psychiatric problems, but this question has not been fully researched. People with physical limitations may not be able to participate in certain meditative practices involving physical movement. Individuals with existing mental or physical health conditions should speak with their health care providers prior to starting a meditative practice and make their meditation instructor aware of their condition."[285]

Discomfort during meditation: Meditation-based interventions may results in discomfort with attention to unpleasant thoughts, feelings or body sensations. Some individuals may experience an initial increase in undesirable emotions with increased attention to them. In order to minimize risk, instructors encourage participants to be aware of their discomfort and to set their own limitations. Participants are encouraged to alter their postures, including standing, sitting in chairs, lying down, as needed in order to be comfortable. In addition, the curriculum of the training program is specifically geared towards addressing such discomfort.

Psychological distress: Research subjects participating in this study may have feelings of loss of privacy from being contacted about participating in the study, and possible psychological distress caused by questions on the diagnostic interview that bring up painful memories or feelings. However, the resulting potential for injury to research subjects is judged to be minimal. We have already contacted and clinically evaluated thousands of participants from the New England Family using similar assessment procedures to this study, with good responses from the participants. With regard to psychological distress from taking part in the MBHT intervention, given that screening questions will exclude participants with substantial mental illness, and given the NCAAM statement above that "Meditation is considered to be safe for healthy people.",[285] we expect that risk of psychological distress will be low. The risk of increased psychological distress from meditation will be clearly outlined in the consent form and participants will be encouraged to consult with both the course instructor and study staff in the case of any increased distress. One of the co-investigators (Dr. Britton) is a clinical psychologist. She will be available to advise on any psychological events that occur, and provide referrals for treatment if needed.

Loss of confidentiality: Likelihood: rare. Minimization: Confidentiality will be maintained by numerically coding all data, by disguising identifying information, and by keeping all data in locked file drawers. All information obtained from participants will be accessible only to research staff.

Potential Benefits of the Proposed Research to Human Subjects and Others: There may be no direct benefits from participating in this study. The potential benefits of participating in the proposed study include improved knowledge on stress reduction techniques and risk for hypertension. Each participant will also be contributing to an important study which will answer questions on whether mindfulness-based stress reduction may influence health.

Section E:
Informed Consent

Each potential participant will be fully informed about the nature of the study, its risks and benefits, and about his/her rights as a research subject. The person performing the consent will be either Dr. Loucks or a trained undergraduate trained research assistant. The participant will sign and receive a copy of the document stating that he/she has given his/her informed consent to participate before interviewing is begun. In every instance, project personnel will verbally review the content of the consent form before participants sign.

Agreement to Participate in a Research Study

Investigation of the effects of mindfulness meditation on blood pressure and well-being

You are being asked to take part in a Brown University research study about the effects of mindfulness and hypertension education on blood pressure and risk factors for hypertension. This form will explain the purpose of the study, how the study will be carried out and what you will be expected to do. It will also explain the possible risks and possible benefits of being in the study. If any part of the following description is not clear to you, you are encouraged to contact the researcher to answer any questions before you decide whether to take part in the study. If you decide to participate, please fill out and sign the last page of this form.

1a. Nature and Purpose of the Study

The purpose of the study is to investigate the impact of mindfulness practices and health education on blood pressure. You have been selected for this study because you expressed interest and met entrance criteria for having prehypertension or hypertension, or another cardiovascular risk factor that could be influenced by this program. In order to assess the effects of these practices, you will be asked to complete some questionnaires, and a laboratory assessment before and after learning the mindfulness practices.

1b. Explanation of Procedures

If you agree to participate, you will be asked to consent to the following:

1) Participate in an interview in which you will be asked questions about past and present mental health, including depression and suicide.

2) Complete questionnaires about a wide range of topics, including your diet, physical activity, smoking, medication use, personality, emotions, attention and past experiences, including stressful or traumatic experiences. These questions will probe sensitive psychological areas, including physical, emotion and sexual abuse. These questionnaires may take up to 2 hours to complete. By completing the interviews and questionnaires, you are giving the researchers permission to use the information you have provided. You have the right not to answer any of the questions.

3) Directly assessed blood pressure, height, weight, waist circumference and hip circumference.

4) You will be asked to perform some cognitive tasks. One of these tasks may involve computer-based tests of attention or decision-making. Together these tests may take as long as 45 minutes.

5) You will be randomly assigned (like the flip of a coin) to enter one of two 9 week meditation programs.

6) You will participate in the 9-week meditation program, which consists of 9 weekly sessions of 1-2.5 hours each and may include one 6 hour weekend retreat. Daily homework assignments may take as long as one hour and consist of practicing mindfulness exercises with the aid of a guided audio CD and completing worksheets related to stress, thoughts, and common reactions to various types of events.

7) Class sessions will be audio taped so we can analyze the quality of the treatment you receive. The recordings will be transcribed so that we may analyze the text. The recordings will be identified by study number, will only be heard by study staff and will be destroyed after transcription.

8) You will be asked to complete a few short questionnaires each week during the 9 week condition and to notify the lab if you change your medication in any way.

9) After 9 weeks, you will be asked to complete a second packet of questionnaires and return to the laboratory to repeat the same procedures for a second day of testing.

10) Three months and six months after the end of the 9 week condition, you will be asked to return to the laboratory to repeat the same testing procedures.

Feedback:
At the end of the study, you will receive individual feedback about the changes that occurred since the first assessment. Specifically, you will receive an individualized handout listing % change (increase or decrease) on scales of attention, stress, mood, health behaviors and blood pressure across the study.

2. Discomforts and Risks
The questionnaires are routine, standardized forms for epidemiologic research. Certain questions may be upsetting as they may probe sensitive psychological areas and inquire about upsetting or traumatic events, including physical, sexual or emotional abuse and/or current psychiatric symptoms. Since your participation is voluntary, you have the right to skip any questions that make you uncomfortable.

Meditation-based interventions may results in discomfort with attention to unpleasant thoughts, feelings or body sensations. Some individuals may experience an initial increase in undesirable feelings with increased attention to them.

3. Benefits
We cannot and do not guarantee or promise that you will receive any direct benefits from this study. However, participation in the study creates the potential benefit of a) identifying effective treatments for elevated blood pressure, b) gaining knowledge of the effects of mindfulness practices, c) gaining first-hand knowledge about the most current scientific methods in scientific research d) receiving information about your psychological and physical functioning.

4. Alternative Therapies
A number of different therapies, including antihypertensive medication, diet changes, physical activity, and reducing excessive alcohol consumption may also be beneficial for reducing blood pressure. Education about these therapies are integrated into this course, but other forms of these alternative therapies are also available in the community.

5. Confidentiality
Your responses for this study will be kept confidential. All data that we collect will be linked to a study ID# instead of your name. All questionnaires in this study will be filled out through an online survey website (SurveyMonkey). All of these questionnaires will be linked solely to your study ID#, so that your identity is protected and your answers are confidential. Although these measures have been taken to protect your personal information, complete confidentiality cannot be guaranteed when transmitting information over the internet.

While your confidentiality is protected to the extent of the law, there are limitations to confidentiality. If your questionnaire responses indicate that you pose a serious danger to yourself or to another person, then the PI, or a co-investigator (Willoughby Britton) who is a clinical psychologist, may contact the you

to discuss your responses and possible referral to another treatment provider. Questionnaire items that may warrant follow-up include endorsements of statements about hurting yourself or others, any high scores in depression, anxiety, aggressive behaviors, or other clinically significant problems. You should also know that there are times when the law might require the release of your responses without your permission. For example, State law requires researchers to report abuse or neglect of children to the Department of Children, Youth and Families (DCYF). State law also requires researchers to report abuse or neglect of people age 60 and older to the Department of Elderly Affairs.

The findings of the study may be used for medical publication. Your name will not be used in any published reports about this study. Results will be reported in a summarized manner in such a way that you cannot be identified. All personally identifiable information will be "de-identified" and only a unique code number will be used. Study records will be identified with a unique code number and initials. All study records and specimens will be stored in a secure storage area.

Keeping study records: The Principal Investigator for this study will keep your research records indefinitely for research purposes.

6. Refusal/Withdrawal

Participation is voluntary, and you may decide to opt out or refuse any part of participation, including not answering certain questions. If you decide now to participate, you can change your mind later and quit the study. The decision to not participate or to withdraw from the study will not adversely affect current or future interactions with Brown University.

If you decide not to participate, or if you quit the study, we will provide you with referrals for alternative treatments, if desired.

7. Contact Information

If you have questions about study procedures at any time you may contact the researchers: Dr. Eric B. Loucks, email: eric.loucks@brown.edu, telephone (401) 863-6283. If you would like more information about the rules for research studies, or the rights of people who take part in those studies, you may contact the Brown University Research Protections Office, telephone number 1-866-309-2095 or 401-863-3050.

CONSENT FORM:
Please sign and return.

Please read the following agreement and sign to consent to participating.

I HAVE READ THE ABOVE DESCRIPTION OF THIS STUDY. ALL OF MY QUESTIONS HAVE BEEN SATISFACTORILY ANSWERED, AND, I AGREE TO PARTICIPATE IN THIS RESEARCH STUDY.

____
PRINT NAME

____ __________________
Signature of participant **Date**

CONTACT INFORMATION

Name (print):____

Permanent Address:____

Email(s):____

Telephone:_________________________**(cell)**____________________________**(other)**

Section F:
Recruiting Materials

Front of Advertisement Card:

Brown University
School of Public Health
Department of Epidemiology

RESEARCH STUDY:
Mindfulness-Based Intervention for Blood Pressure Reduction

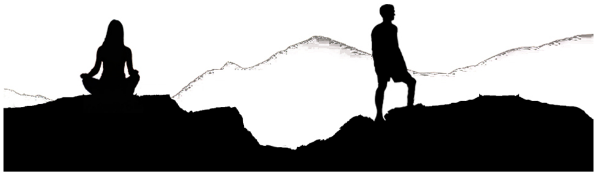

Free to qualified participants

Back of Advertisement Card:

This is a 9-week program that includes mindfulness meditation, mindful movements, and education about risk factors for blood pressure. The study is testing whether combining these interventions lowers blood pressure. The program consists of nine weekly sessions that are 1 or 2.5 hours (depending on study arm), and an all-day weekend retreat.

Research participation includes interviews, questionnaires and measurements of blood pressure, height, weight, and waist circumference, before and after the meditation program.

Contact: For more information, or to see if you qualify, call the Brown Sociobiology Laboratory at 401-863-6283, or email SBL@gmail.com. See website at [URL needs to be created].

Sample Text of Recruitment Letter (placed on Dr. Loucks' letterhead):

[Date]

Dear _____,

Your physician [name of physician] identified you as someone with prehypertension or hypertension who may be interested in participating in a new study at Brown University that is designed to evaluate effects of an intervention called Mindfulness-Based Hypertension Therapy (MBHT). MBHT combines mindfulness practices such as meditation and mindful movements with education about hypertension management and prevention. The aim of MBHT is to create a skill set and supportive environment to help to reduce blood pressure, and improve overall well-being. Your involvement in this study could help us better understand if MBHT is effective, and how it could be improved. The MBHT intervention will take place over 9 weeks, and would involve you attending a class once per week that would be between 1 and 2.5 hours, depending on which group you are assigned to. You may also be assigned to participate in a full day mindfulness retreat that would be about 7.5 hours duration. Also, health assessments would be performed before you start the intervention, and again immediately at the end of the 9-week intervention, as well as at 3 months and 6 months follow-up. The assessments would include measures such as blood pressure, height, weight, diet, physical activity, smoking, depression, stress, and medication use. These assessments will take 1-2 hours to complete.

At the end of the study, you will receive individual feedback about the changes that occurred since the first assessment. Specifically, you will receive an individualized handout listing your changes on scales of attention, stress, mood, health behaviors and blood pressure across the study.

We cannot and do not guarantee or promise that you will receive any direct benefits from this study. However, participation in the study creates the potential benefit of a) identifying effective treatments for elevated blood pressure, b) gaining knowledge of the effects of mindfulness practices, c) gaining first-hand knowledge about the most current scientific methods in scientific research d) receiving information about your psychological and physical functioning.

If you are interested in participating in this study, please call 401-863-6283, or email eric.loucks@brown.edu.

Sincerely yours,

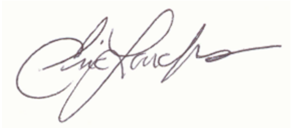

Eric B. Loucks, Ph.D.
Assistant Professor

Section G:
Questionnaires and Focus Group Topics

SCREENING QUESTIONNAIRE
(PHONE-DELIVERED)

MBHT PHONE SCREEN

Date of Screen: ___________________ Interviewer: _______________________

Screening #: _________________________________ (month.date.year.hour.minute)

Name: ____

Address: ____

Phone number(s): ____

Email address (or mailing if no email):____

Notes from Interviewer:

____
____
____

My name is ____________. I am calling from the Epidemiology Department at the Brown University School of Public Health, because you expressed interest in participating in our mindfulness study. I want to do 10-15 minutes phone interview with you to determine if you are a good match for this particular study. Is now a good time to speak? [If yes, proceed. If no, ask for other times and note in Potential Subject Log].

There are a few things that I'd like to make clear before we start the interview. First of all, some of the questions will be very personal and sensitive. Are you in a private place to talk? Because this interview is of a personal nature, it is important that you understand that everything you say will be kept strictly confidential. No one outside of our project will ever be able to see your answers, and we will not keep your name in the same place as any of your answers. If you are not eligible after the phone screen, we will destroy your information. If you like, though, we can keep your information on file for future studies.

INCLUSION CRITERIA: Need YES to all			
			Comments
AGE	Age_______ Between 18 and 65?	YES NO	
FXN	Highest level of education?		
FXN	Current occupation?		
ENG	Can you read and write in English?	YES NO	
INTEREST	What interested you about this study? What kind of symptoms are you hoping to treat?	YES NO	

EXCLUSION CRITERIA: LIFETIME: must be NO			
			Comments
Bipolar depression MANIA	Has anyone ever told you that you have bipolar disorder or manic depression?	YES NO	
BPD	Has anyone ever used the word "Borderline" to describe you?	YES NO	
Psychosis	Have you ever had a hallucination or seen things that other people can't see, or hear things other people can't hear? Have you ever been diagnosed with schizophrenia or psychosis?	YES NO	
MANIA/ Antipsychotic MEDS	Have you ever taken any of the following medications?<br>Lithium?<br>Seroquel (quetiapine)<br>Abilify (aripiprazole)<br>Zyprexa (olanzapine)<br>Clozaril (clozapine)<br>Haldol/Haloperidol<br>Geodon (ziprasidone)<br>Risperdal (risperidone)	YES NO	
Self-harm	Have you ever deliberately cut or injured yourself? Have you ever had a suicide attempt? (Details/age)	YES NO	
Antisocial	Have you ever been arrested? Any Legal problems? Have you ever been in a fight?	YES NO	

EXCLUSION: CURRENT (check timeframe)			
			Comments
PTSD	Would you say you have a trauma history? Physical sexual abuse, assault rape? Where are you with that now?<br>Any problems with dissociation (memory loss)? Flashbacks?	YES NO	Last month?
OCD	Any problems with obsessions or compulsions? Washing hands or checking the oven over and over? If yes, Has anyone diagnosed you with obsessive compulsive disorder?	YES NO	Last month?
PANIC	Have you ever had a panic attack? (Sweating, heart palpitations, nausea, trouble breathing, fear of dying/choking/going crazy…<br>Out of blue or triggered?<br>Have you had one in the last month?	YES NO	Last month?
ALCOHOL	How much alcohol do you currently drink?<br>Any drug use?<br>Ever abuse drug/alcohol?<br>Time in recovery – min 1 year	YES NO	Last year?
EATING DIS	How is your relationship with food? Ever have an eating disorder? Starving, Binge eating, or vomiting? Where are you with that now?	YES NO	Last year?
MEDITATION	What is your experience with meditation?<br>Rule out current regular practice (>1 week)<br>Lifetime daily mindfulness meditation (see "what is meditation?)	YES NO	
MISC	Any other psychological problem I haven't mentioned?<br>Social Anxiety? Ok in groups?<br>Phobias?	YES NO	
Time commitment	(After study explanation)This study is a significant time commitment, is this a good time for you?		
DECISION	PASS NO PASS QUERY		

PASS FORM: Initial appropriate boxes with dates		Date/initials
NO PASS	According to this test, it doesn't look like you are going to be a good fit for the study. There may be other studies you quality for. Would you like me to keep your information to pass on to these studies? Otherwise, our copy of this information can be destroyed.	
QUERY	There are several items I will need to check with my supervisor about. We will get back in touch.	
PASS:<br>Study description and requirements<br>Time commitment	You qualify for the study. If you want to continue,<br>the next phase would require an interview, a packet of questionnaires and evening visit to the laboratory before the 9 week meditation program. After the program, you would fill out the same questionnaires and do the lab visit again.<br>The meditation program is either 1 or 2.5 hours once a week, usually in the evening, and requires up to an hour of practice every day. There also may be an all-day retreat on a Saturday part way through the course.<br>Is this something that you would be able to make time for in your schedule?	YES NO
SCHEDULE<br>Available for current program?	It's important to attend all 9 sessions.<br>The next program is:<br>Availability:<br>Mon 9:00-11:30, 4-6:30 pm<br>Tues 9:00-11:30, 4-6:30 pm<br>Wed 9:00-11:30, 4-6:30 pm<br>Thurs 9:00-11:30, 4-6:30 pm<br><br>Other:	YES NO

QUESTIONNAIRES ANSWERED BY PARTICIPANTS AT BASELINE

Questionnaire Table of Contents

Introductory Questions 8
Background Questions 10
Physical Activity 14
Food and Drinks (FD) 16
Eating Practices (EP) 21
Alcohol Consumption (AC) 23
Smoking (SM) 24
Medications (ME) 25
Self-Control 47
Sense of Control 48
CES-D Depression Inventory 50
Perceived Stress Scale 52
Mindfulness 53
Emotion Regulation 64
Beck Anxiety Inventory 66
Parent's Education 67
Your Childhood Experiences 69
Loneliness 74
Your Sleep 75

<u>Introductory Questions</u>

IQ1. What is your main reason for participating in this study?

IQ2. What do you care about most?

IQ3. What gives you most pleasure in your life?

IQ4. What are your greatest worries?

Please list three personal goals you have for taking this mindfulness program:

1.____

2. ____

3. ____

Background Questions

B1. What is your age? _____ years old ☐ I do not know ☐ I prefer not to answer

B2. Are you Latino or Hispanic?

☐ No → ***skip to B3***
☐ Yes
☐ I do not know
☐ I prefer not to answer

B2a. Which of the following represents your family's country of origin? ***(check all that apply)***

☐ Cuba
☐ Mexico
☐ Puerto Rico
☐ Spain
☐ South America
☐ Columbia
☐ Dominican Republic
☐ Other Central American
☐ Other: ________________________
☐ I do not know
☐ I prefer not to answer

B3. If you were asked to put yourself into only one of these groups, in which one would you place yourself? ***(select <u>one</u> only):***

☐ Asian
☐ Pacific Islander
☐ African American/Black
☐ Caucasian/White
☐ Native American
☐ Other : _________________________
☐ I do not know
☐ I prefer not to answer

B4. Which of the following best describes your current work situation? ***(select <u>one</u> only)***

☐ Working full-time
☐ Working part-time
☐ Retired
☐ Unemployed: Looking for work
☐ Unemployed: Not currently looking for work
☐ Unemployed due to disability
☐ Keeping house or raising children full-time
☐ Military
☐ Full-time student
☐ Other:________________________
☐ I do not know
☐ I prefer not to answer

B9. What is the highest grade or level of regular school you have completed?

☐ Elementary School
☐ Junior High
☐ High School
☐ College
☐ Graduate School
☐ I do not know
☐ I prefer not to answer

B11. What is the highest degree you earned? ***(select <u>one</u> only)***

☐ GED
☐ High school diploma or equivalency
☐ Associate degree (Junior College)
☐ Bachelor's degree
☐ Master's degree
☐ Doctorate (Phd, EdD, etc)
☐ Professional (MD, JD, DDS, DVM, etc.)
☐ Other: ______________________________
☐ None of the above (less than GED or less than high school graduation)
☐ I do not know
☐ I prefer not to answer

B12. Did you ever attend any other school like a technical, vocational, or trade school?

☐ No
☐ Yes
☐ I do not know
☐ I prefer not to answer

B13. In total, about how many full-time years of education have you had, including 1st grade and all years of school after 1st grade?

________ years

☐ I do not know
☐ I prefer not to answer

B14. Do you currently live alone?

- □ No
- □ Yes → ***skip to B15***
- □ I do not know
- □ I prefer not to answer

B14a. How many people are currently living in your household, including yourself?

_________ people □ I do not know □ I prefer not to answer

B14b. Of these people, how many are under 18?

_________ people □ I do not know □ I prefer not to answer
(if none, write 0)

B14c. Of the adults in your household (including yourself), how many bring income into the household?

_________ people □ I do not know □ I prefer not to answer
(if none, write 0)

B16. How much did YOU earn, before taxes and other deductions, during the past 12 months? ***(Please include all income from your job(s), money from government programs, child support, interest and dividends, money from rental property, etc.)***

- □ Less than $5,000
- □ $5,000 through $11,999
- □ $12,000 through $15,999
- □ $16,000 through $24,999
- □ $25,000 through $34,999
- □ $35,000 through $49,999
- □ $50,000 through $74,999
- □ $75,000 through $99,999
- □ $100,000 and greater
- □ I do not know
- □ I prefer not to answer

B17. How much did your HOUSEHOLD earn, before taxes and other deductions, during the past 12 months? ***(Please include all income from your job(s), your family member's job(s), money from government programs, child support, interest and dividends, money from rental property, etc.)***

- □ Less than $5,000
- □ $5,000 through $11,999
- □ $12,000 through $15,999
- □ $16,000 through $24,999
- □ $25,000 through $34,999
- □ $35,000 through $49,999
- □ $50,000 through $74,999
- □ $75,000 through $99,999
- □ $100,000 and greater
- □ I do not know
- □ I prefer not to answer

Physical Activity

Physical activities are activities where you move and increase your heart rate above its resting rate, whether you do them for pleasure, work, or transportation. The following questions ask about the amount and intensity of physical activity you usually do. The intensity of the activity is related to *the amount of energy* you use doing these activities.

Examples of physical activity intensity levels:

Light activities
Your heart beats slightly faster than normal
You can talk and sing

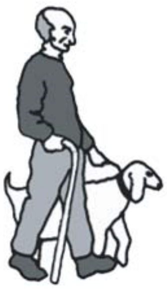
Light exercise

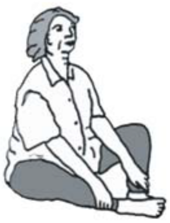
Light stretching

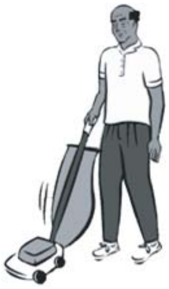
Light vacuuming or yard work

Moderate activities
Your heart beats faster than normal
You can talk but not sing

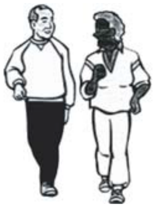
Brisk walking

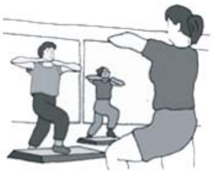
Aerobics class

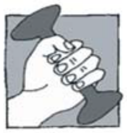
Strength training

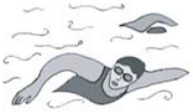
Swim gently

Vigorous activities
Your heart rate increases a lot
You can’t talk, or your talking is broken up by large breaths

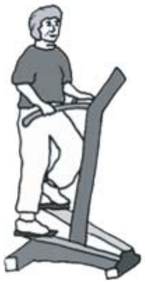
Aerobics classes

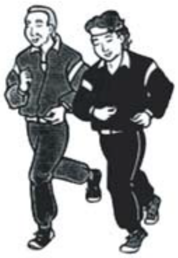
Jogging, Running, or Power Walking

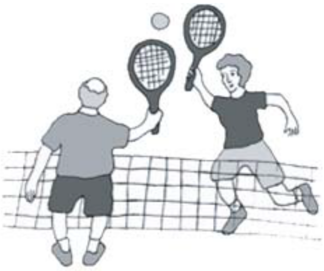
Singles tennis, Racquetball, Pickle ball

How physically active are you?

	YES	NO
PA1. I rarely or never do any physical activities.		
PA2. I do some light and/or moderate physical activities, but not every week.		
PA3. I do some light physical activity every week.		
PA4. I do moderate physical activity every week but less than 5 days per week or less than 30 minutes on those days.		
PA5. I do vigorous physical activities every week, but less than 3 days per week or less than 20 minutes on those days.		
PA6. I do 30 minutes or more per day of moderate physical activities 5 or more days per week.		
PA7. I do 20 minutes or more per day of vigorous physical activities 3 or more days per week.		
PA8. I do activities to increase muscle strength, such as lifting weights or calisthenics, once a week or more.		
PA9. I do activities to improve flexibility, such as stretching or yoga, once a week or more.		

Food and Drinks (FD)

The first set of questions ask about foods you usually eat or drink. For each food, please place an X in the box (e.g. ☒) for the answer that best matches how often you eat or drink it. Remember to count fruit or vegetables that were part of another dish, like a banana on cereal or vegetables in a stew. Also, please pay attention to the serving sizes. For example, one serving size of bread is one slice, so a sandwich would contain 2 servings of bread. Please include all foods you eat, both at home and away from home. Enclosed are pictures of common serving sizes to assist you in filling out your questionnaire.

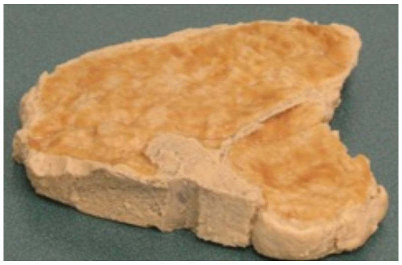

4-6 ounces

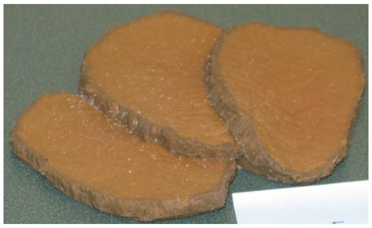

3 ounces

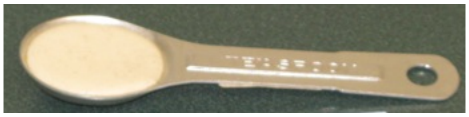

1 teaspoon

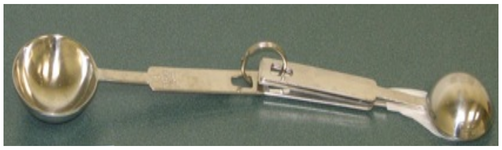

1 tablespoon

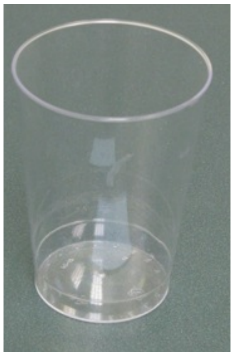

8 ounces

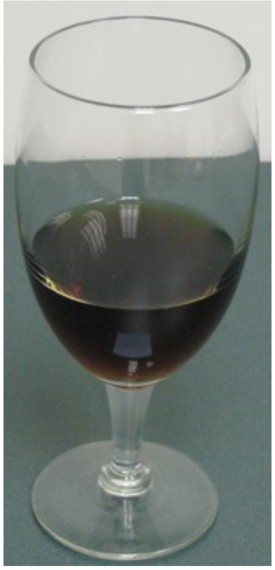

Small glass

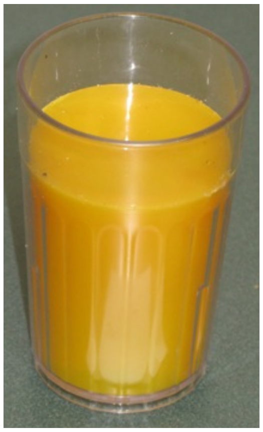

1 cup

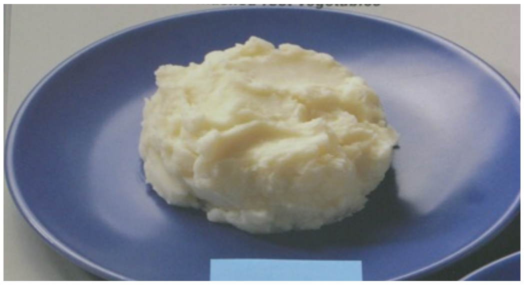

1 cup

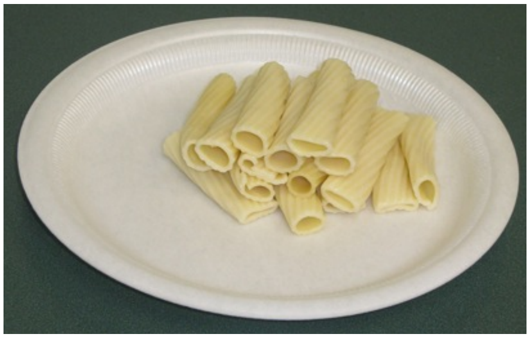

4 ounces

How Often You Eat or Drink This Item

	Never or less than 1 per month	1-3 per month	1 per week	2-4 per week	5-6 per week	1 per day	2-3 per day	4-5 per day	More than 5 per day	I don't know	I prefer not to answer
**FD1**. Skim/low fat milk (8 oz. glass)	☐	☐	☐	☐	☐	☐	☐	☐	☐	☐	☐
**FD2.** Whole milk (8 oz. glass)	☐	☐	☐	☐	☐	☐	☐	☐	☐	☐	☐
**FD3.** Ice cream (1 cup)	☐	☐	☐	☐	☐	☐	☐	☐	☐	☐	☐
**FD4.** Yogurt (1 cup)	☐	☐	☐	☐	☐	☐	☐	☐	☐	☐	☐
**FD5.** Cottage or ricotta cheese (1/2 cup)	☐	☐	☐	☐	☐	☐	☐	☐	☐	☐	☐
**FD6.** Butter, added to food or bread (1 pat), exclude use in cooking	☐	☐	☐	☐	☐	☐	☐	☐	☐	☐	☐
**FD7.** Fruit (not including fruit juice. A serving = 1 piece, or 1/2 grapefruit, or 1/2 cup berries)	☐	☐	☐	☐	☐	☐	☐	☐	☐	☐	☐
**FD8.** 100% fruit juice, not including fruit drinks (small glass)	☐	☐	☐	☐	☐	☐	☐	☐	☐	☐	☐

How Often You Eat or Drink This Item

	Never or less than 1 per month	1-3 per month	1 per week	2-4 per week	5-6 per week	1 per day	2-3 per day	4-5 per day	More than 5 per day	I don't know	I prefer not to answer
**FD9.** Vegetables - raw and cooked (1/2 cup), including mixed dishes such as soups, casseroles and lasagna. Do not include potatoes.	☐	☐	☐	☐	☐	☐	☐	☐	☐	☐	☐
**FD10.** Eggs (do not include egg beaters) (1)	☐	☐	☐	☐	☐	☐	☐	☐	☐	☐	☐
**FD11.** Chicken or turkey (4-6 oz.)	☐	☐	☐	☐	☐	☐	☐	☐	☐	☐	☐
**FD12.** Processed meat (1 piece sausage, 1 slice salami, 2 pieces bacon)	☐	☐	☐	☐	☐	☐	☐	☐	☐	☐	☐
**FD13.** Hot dog/ hamburger	☐	☐	☐	☐	☐	☐	☐	☐	☐	☐	☐

How Often You Eat or Drink This Item

	Never or less than 1 per month	1-3 per month	1 per week	2-4 per week	5-6 per week	1 per day	2-3 per day	4-5 per day	More than 5 per day	I don't know	I prefer not to answer
**FD14**. Beef, pork, or lamb as a sandwich or mixed dish, e.g. stew, casserole, lasagna, etc.	☐	☐	☐	☐	☐	☐	☐	☐	☐	☐	☐
**FD15**. Beef, pork, or lamb as a main dish, e.g. steak, roast, ham, etc. (4-6 oz.)	☐	☐	☐	☐	☐	☐	☐	☐	☐	☐	☐
**FD16**. Fish (including canned tuna) (3-5 oz.)	☐	☐	☐	☐	☐	☐	☐	☐	☐	☐	☐
**FD17**. Shellfish such as shrimp, lobster, scallops and clams as a main dish	☐	☐	☐	☐	☐	☐	☐	☐	☐	☐	☐
**FD18**. White bread (including pita bread) (slice)	☐	☐	☐	☐	☐	☐	☐	☐	☐	☐	☐
**FD19.** Dark bread (incl. whole wheat and rye) (slice)	☐	☐	☐	☐	☐	☐	☐	☐	☐	☐	☐
**FD20.** Bakery items (cookies, brownies, doughnuts, cake) (1 piece or slice)	☐	☐	☐	☐	☐	☐	☐	☐	☐	☐	☐
**FD21.** Nuts (small packet, 1 oz., 1 Tbs peanut butter)	☐	☐	☐	☐	☐	☐	☐	☐	☐	☐	☐
**FD22.** Pasta (e.g. spaghetti, and macaroni and cheese; 1 cup cooked)	☐	☐	☐	☐	☐	☐	☐	☐	☐	☐	☐
**FD23.** Potatoes, baked, boiled or mashed (do not include French fries/ chips) (1 potato or 1 cup)	☐	☐	☐	☐	☐	☐	☐	☐	☐	☐	☐

How Often You Eat or Drink This Item

	Never or less than 1 per month	1-3 per month	1 per week	2-4 per week	5-6 per week	1 per day	2-3 per day	4-5 per day	More than 5 per day	I don't know	I prefer not to answer
**FD24**. Regular soft drinks (including caffeine free, but NOT including diet colas) (consider the serving size as 1 glass, bottle or can)	☐	☐	☐	☐	☐	☐	☐	☐	☐	☐	☐
**FD25**. Coffee (regular or decaf) (1 cup)	☐	☐	☐	☐	☐	☐	☐	☐	☐	☐	☐

Eating Practices (EP)

	Definitely True	Mostly True	Mostly False	Definitely False
1. I deliberately take small helpings to control my weight.	◻	◻	◻	◻
2. I start to eat when I feel anxious.	◻	◻	◻	◻
3. Sometimes when I start eating, I just can't seem to stop.	◻	◻	◻	◻
4. When I feel sad, I often eat too much.	◻	◻	◻	◻
5. I don't eat some foods because they make me fat.	◻	◻	◻	◻
6. Being with someone who is eating, often makes me want to also eat.	◻	◻	◻	◻
7. When I feel tense or ''wound up'', I often feel I need to eat.	◻	◻	◻	◻
8. I often get so hungry that my stomach feels like a bottomless pit.	◻	◻	◻	◻
9. I'm always so hungry that it's hard for me to stop eating before finishing all of the food on my plate.	◻	◻	◻	◻
10. When I feel lonely, I console myself by eating.	◻	◻	◻	◻
11. I consciously hold back on how much I eat at meals to keep from gaining weight.	◻	◻	◻	◻
12. When I smell a sizzling steak or see a juicy piece of meat, I find it very difficult to keep from eating even if I've just finished a meal.	◻	◻	◻	◻
13. I'm always hungry enough to eat at any time.	◻	◻	◻	◻
14. If I feel nervous, I try to calm down by eating.	◻	◻	◻	◻
15. When I see something that looks very delicious, I often get so hungry that I have to eat right away.	◻	◻	◻	◻
16. When I feel depressed, I want to eat.	◻	◻	◻	◻

	Almost Never	Seldom	Usually	Almost Always
17. How often do you avoid 'stocking up'' on tempting foods?	◻	◻	◻	◻

	Unlikely	A little likely	Somewhat likely	Very likely
18. How likely are you to make an effort to eat less than you want?	◻	◻	◻	◻

	Never	Rarely	Sometimes	At least once a week
19. Do you go on eating binges even though you're not hungry?	☐	☐	☐	☐

	Only at mealtimes	Sometimes between meals	Often between meals	Almost always
20. How often do you feel hungry?	☐	☐	☐	☐

	1	2	3	4	5	6	7	8
21. On a scale from 1 to 8, where 1 means no restraint in eating and 8 means total restraint, what number would you give yourself? Mark the number that best applies to you:	☐	☐	☐	☐	☐	☐	☐	☐

Alcohol Consumption (AC)

A drink of alcohol is defined as 1 can or bottle of beer, 1 glass of wine, 1 can or bottle of wine cooler, 1 cocktail, or 1 shot of liquor.

AC1. During the past 30 days, how many days per week or per month did you have at least 1 drink of any alcoholic beverage? [if none, **STOP**]

____

AC2. On the days when you drank, about how many drinks did you drink on average?

____

AC3. ***Men:*** Considering all types of alcoholic beverages, how many times during the past 30 days did you have **5** or more drinks on an occasion?

Women: Considering all types of alcoholic beverages, how many times during the past 30 days did you have ***4*** or more drinks on an occasion?

____

Smoking (SM)

SM1. Have you smoked at least 100 cigarettes in your entire life?

- ☐ Yes
- ☐ No
- ☐ Don't know
- ☐ Prefer not to answer

SM2. Did you ever become a daily smoker (that is, smoke every day or nearly every day for two months or longer)?

- ☐ Yes
- ☐ No → ***skip to SM6***
- ☐ Don't know
- ☐ Prefer not to answer

SM3. How old were you when you last smoked daily?

Age ______ (in years)

☐ Don't know ☐ Prefer not to answer ☐ Still smoking daily

SM4. Do you smoke cigarettes now?

- ☐ Yes
- ☐ No → ***skip to next section***
- ☐ Don't know
- ☐ Prefer not to answer

SM4a. How many cigarettes per day do you smoke? (One pack equals 20 cigarettes)

Number of cigarettes ________

- ☐ Don't know
- ☐ Prefer not to answer

Medications (ME)

ME1. Do you take any prescription medications or over-the-counter drugs?

- ☐ No → ***skip to end of survey***
- ☐ Yes
- ☐ Don't know
- ☐ Prefer not to answer

What is the name of the first prescription medication or over-the-counter drug that you take?

ME2a. ☐ Label product name: ______________________________

☐ Label generic name: ______________________________

☐ Don't know ☐ Prefer not to answer

ME2b. What is the dosage form?

Oral
- ☐ Pill, tablet, or capsule
- ☐ Sublingual or orally-disintegrating tablet
- ☐ Liquid solution or suspension (drink, syrup)
- ☐ Powder

Topical
- ☐ Liquid, cream, gel, or ointment
- ☐ Ear drops (otic)
- ☐ Eye drops (ophthalmic)
- ☐ Skin patch (transdermal)

Inhaled
- ☐ Inhaler or nebulizer

Injected
- ☐ Injection

Suppository
- ☐ Rectal (e.g., enema)
- ☐ Vaginal (e.g., douche, pessary)

Other:
- ☐ Don't know
- ☐ Prefer not to answer

ME2c. How frequently do you take it?

- ☐ _________ times per day
- ☐ _________ times per week
- ☐ _________ times per month
- ☐ Don't know
- ☐ Prefer not to answer

ME2d. What is the strength? ***Record strength of how it is actually taken, not how it is prescribed.)***

- ☐ _________ %
- ☐ _________ mg
- ☐ _________ mcg
- ☐ _________ grams
- ☐ _________ I.U.
- ☐ _________ Other unit: _____________
- ☐ Don't know
- ☐ Prefer not to answer

ME2d1. Total dosage **per day.** ***(Record strength of how it is actually taken, not how it is prescribed.)***

- ☐ _________ %
- ☐ _________ mg

- ☐ _________ mcg
- ☐ _________ grams
- ☐ _________ I.U.
- ☐ _________ Other unit: _____________
- ☐ Don't know
- ☐ Prefer not to answer

ME2e. Do you take it regularly or only as needed?

- ☐ Regularly
- ☐ Only as needed
- ☐ Don't know
- ☐ Prefer not to answer

ME2f. For how long have you been taking it?

- ☐ For _________ days
- ☐ For _________ weeks
- ☐ For _________ months
- ☐ For _________ years
- ☐ Don't know
- ☐ Prefer not to answer

ME2g. ***Interviewer comments*:**

____

ME2h. Do you take any other prescription medications or over-the-counter drugs?

- ☐ No → ***skip end of survey***
- ☐ Yes
- ☐ Don't know
- ☐ Prefer not to answer

What is the name of the next prescription medication or over-the-counter drug that you take?

ME83a. ☐ Label product name:

____

☐ Label generic name: ____

☐ Don't know ☐ Prefer not to answer

ME3b. What is the dosage form?

Oral
- ☐ Pill, tablet, or capsule
- ☐ Sublingual or orally-disintegrating tablet
- ☐ Liquid solution or suspension (drink, syrup)
- ☐ Powder

Topical
- ☐ Liquid, cream, gel, or ointment
- ☐ Ear drops (otic)
- ☐ Eye drops (ophthalmic)
- ☐ Skin patch (transdermal)

Inhaled
- ☐ Inhaler or nebulizer

Injected
- ☐ Injection

Suppository
- ☐ Rectal (e.g., enema)
- ☐ Vaginal (e.g., douche, pessary)

Other:
- ☐ Don't know
- ☐ Prefer not to answer

ME3c. How frequently do you take it?

- ☐ _________ times per day
- ☐ _________ times per week
- ☐ _________ times per month
- ☐ Don't know
- ☐ Prefer not to answer

ME3d. What is the strength? ***(Record strength of how it is actually taken, not how it is prescribed.)***

- ☐ _________ %
- ☐ _________ mg
- ☐ _________ mcg
- ☐ _________ grams
- ☐ _________ I.U.
- ☐ _________ Other unit: _____________
- ☐ Don't know
- ☐ Prefer not to answer

ME3d1. Total dosage **per day.** ***(Record strength of how it is actually taken, not how it is prescribed.)***

- ☐ _________ %
- ☐ _________ mg
- ☐ _________ mcg
- ☐ _________ grams
- ☐ _________ I.U.
- ☐ _________ Other unit: _____________
- ☐ Don't know
- ☐ Prefer not to answer

ME3e. Do you take it regularly or only as needed?

- ☐ Regularly
- ☐ Only as needed
- ☐ Don't know
- ☐ Prefer not to answer

ME3f. For how long have you been taking it?

- ☐ For _________ days
- ☐ For _________ weeks
- ☐ For _________ months
- ☐ For _________ years
- ☐ Don't know
- ☐ Prefer not to answer

ME3g. ***Interviewer comments*:**

____

ME3h. Do you take any other prescription medications or over-the-counter drugs?

- ☐ No → ***skip to end of survey***
- ☐ Yes
- ☐ Don't know
- ☐ Prefer not to answer

What is the name of the next prescription medication or over-the-counter drug that you take?

ME4a. ☐ Label product name: ____

- ☐ Label generic name: ____
- ☐ Don't know
- ☐ Prefer not to answer

ME4b. What is the dosage form?

Oral
- ☐ Pill, tablet, or capsule
- ☐ Sublingual or orally-disintegrating tablet
- ☐ Liquid solution or suspension (drink, syrup)
- ☐ Powder

Topical
- ☐ Liquid, cream, gel, or ointment
- ☐ Ear drops (otic)
- ☐ Eye drops (ophthalmic)
- ☐ Skin patch (transdermal)

Inhaled
- ☐ Inhaler or nebulizer

Injected
- ☐ Injection

Suppository
- ☐ Rectal (e.g., enema)
- ☐ Vaginal (e.g., douche, pessary)

Other:
- ☐ Don't know
- ☐ Prefer not to answer

ME4c. How frequently do you take it?
- ☐ _________ times per day
- ☐ _________ times per week
- ☐ _________ times per month
- ☐ Don't know
- ☐ Prefer not to answer

ME4d. What is the strength? ***(Record strength of how it is actually taken, not how it is prescribed.)***
- ☐ _________ %
- ☐ _________ mg
- ☐ _________ mcg
- ☐ _________ grams
- ☐ _________ I.U.
- ☐ _________ Other unit: _____________
- ☐ Don't know
- ☐ Prefer not to answer

ME4d1. Total dosage **per day**. ***(Record strength of how it is actually taken, not how it is prescribed.)***
- ☐ _________ %
- ☐ _________ mg
- ☐ _________ mcg
- ☐ _________ grams
- ☐ _________ I.U.
- ☐ _________ Other unit: _____________
- ☐ Don't know
- ☐ Prefer not to answer

ME4e. Do you take it regularly or only as needed?
- ☐ Regularly
- ☐ Only as needed
- ☐ Don't know
- ☐ Prefer not to answer

ME4f. For how long have you been taking it?
- ☐ For _________ days
- ☐ For _________ weeks
- ☐ For _________ months
- ☐ For _________ years
- ☐ Don't know
- ☐ Prefer not to answer

ME4g. ***Interviewer comments*:**

____

ME4h. Do you take any other prescription medications or over-the-counter drugs?

☐ No → *skip to next section* ☐ Don't know

☐ Yes ☐ Prefer not to answer

What is the name of the next prescription medication or over-the-counter drug that you take?

ME5a. ☐ Label product name:

____

☐ Label generic name: ____

☐ Don't know ☐ Prefer not to answer

ME5b. What is the dosage form?

Oral

☐ Pill, tablet, or capsule

☐ Sublingual or orally-disintegrating tablet

☐ Liquid solution or suspension (drink, syrup)

☐ Powder

Topical

☐ Liquid, cream, gel, or ointment

☐ Ear drops (otic)

☐ Eye drops (ophthalmic)

☐ Skin patch (transdermal)

Inhaled

☐ Inhaler or nebulizer

Injected

☐ Injection

Suppository

☐ Rectal (e.g., enema)

☐ Vaginal (e.g., douche, pessary)

Other:

☐ Don't know

☐ Prefer not to answer

ME5c. How frequently do you take it?

☐ _________ times per day

☐ _________ times per week

☐ _________ times per month

☐ Don't know

☐ Prefer not to answer

ME5d. What is the strength? ***Record strength of how it is actually taken, not how it is prescribed.)***

☐ _________ %

☐ _________ mg

☐ _________ mcg

☐ _________ grams

☐ _________ I.U.

☐ _________ Other unit: _____________

☐ Don't know

☐ Prefer not to answer

ME5d1. Total dosage **per day**. ***(Record strength of how it is actually taken, not how it is prescribed.)***

☐ _________ %
☐ _________ mg
☐ _________ mcg
☐ _________ grams
☐ _________ I.U.
☐ _________ Other unit: _____________
☐ Don't know
☐ Prefer not to answer

ME5e. Do you take it regularly or only as needed?

☐ Regularly
☐ Only as needed
☐ Don't know
☐ Prefer not to answer

ME5f. For how long have you been taking it?

☐ For _________ days
☐ For _________ weeks
☐ For _________ months
☐ For _________ years
☐ Don't know
☐ Prefer not to answer

ME5g. ***Interviewer comments*:**

____

ME5h. Do you take any other prescription medications or over-the-counter drugs?

☐ No → ***skip to end of survey***
☐ Yes
☐ Don't know
☐ Prefer not to answer

What is the name of the next prescription medication or over-the-counter drug that you take?

ME6a. ☐ Label product name:

____

☐ Label generic name: ____

☐ Don't know ☐ Prefer not to answer

ME6b. What is the dosage form?

Oral

☐ Pill, tablet, or capsule
☐ Sublingual or orally-disintegrating tablet
☐ Liquid solution or suspension (drink, syrup)
☐ Powder

Topical

☐ Liquid, cream, gel, or ointment
☐ Ear drops (otic)

☐ Eye drops (ophthalmic)
☐ Skin patch (transdermal)

Suppository
☐ Rectal (e.g., enema)
☐ Vaginal (e.g., douche, pessary)

Inhaled
☐ Inhaler or nebulizer

Injected
☐ Injection

Other:
☐ Don't know
☐ Prefer not to answer

ME6c. How frequently do you take it?

☐ _________ times per day
☐ _________ times per week
☐ _________ times per month
☐ Don't know
☐ Prefer not to answer

ME6d. What is the strength? ***(Record strength of how it is actually taken, not how it is prescribed.)***

☐ _________ %
☐ _________ mg
☐ _________ mcg
☐ _________ grams
☐ _________ I.U.
☐ _________ Other unit: _____________
☐ Don't know
☐ Prefer not to answer

ME6d1. Total dosage **per day**. ***(Record strength of how it is actually taken, not how it is prescribed.)***

☐ _________ %
☐ _________ mg
☐ _________ mcg
☐ _________ grams
☐ _________ I.U.
☐ _________ Other unit: _____________
☐ Don't know
☐ Prefer not to answer

ME6e. Do you take it regularly or only as needed?

☐ Regularly
☐ Only as needed
☐ Don't know
☐ Prefer not to answer

ME6f. For how long have you been taking it?

☐ For _________ days
☐ For _________ weeks
☐ For _________ months
☐ For _________ years
☐ Don't know
☐ Prefer not to answer

ME6g. ***Interviewer comments*:**

____

ME6h. Do you take any other prescription medications or over-the-counter drugs?

☐ No → *skip to next section*
☐ Yes
☐ Don't know
☐ Prefer not to answer

What is the name of the next prescription medication or over-the-counter drug that you take?

ME7a. ☐ Label product name: ______________________________

☐ Label generic name: ______________________________

☐ Don't know ☐ Prefer not to answer

ME7b. What is the dosage form?

Oral
☐ Pill, tablet, or capsule
☐ Sublingual or orally-disintegrating tablet
☐ Liquid solution or suspension (drink, syrup)
☐ Powder

Topical
☐ Liquid, cream, gel, or ointment
☐ Ear drops (otic)
☐ Eye drops (ophthalmic)
☐ Skin patch (transdermal)

Inhaled
☐ Inhaler or nebulizer

Injected
☐ Injection

Suppository
☐ Rectal (e.g., enema)
☐ Vaginal (e.g., douche, pessary)

Other:
☐ Don't know
☐ Prefer not to answer

ME7c. How frequently do you take it?

☐ _________ times per day
☐ _________ times per week
☐ _________ times per month
☐ Don't know
☐ Prefer not to answer

ME7d. What is the strength? ***Record strength of how it is actually taken, not how it is prescribed.)***

☐ _________ %
☐ _________ mg
☐ _________ mcg
☐ _________ grams
☐ _________ I.U.
☐ _________ Other unit: _____________
☐ Don't know
☐ Prefer not to answer

ME7d1. Total dosage **per day.** ***(Record strength of how it is actually taken, not how it is prescribed.)***

☐ _________ %
☐ _________ mg

- [ ] _________ mcg
- [ ] _________ grams
- [ ] _________ I.U.
- [ ] _________ Other unit: _____________
- [ ] Don't know
- [ ] Prefer not to answer

ME7e. Do you take it regularly or only as needed?

- [ ] Regularly
- [ ] Only as needed
- [ ] Don't know
- [ ] Prefer not to answer

ME7f. For how long have you been taking it?

- [ ] For _________ days
- [ ] For _________ weeks
- [ ] For _________ months
- [ ] For _________ years
- [ ] Don't know
- [ ] Prefer not to answer

ME7g. ***Interviewer comments***:

____

ME7h. Do you take any other prescription medications or over-the-counter drugs?

- [ ] No → ***skip to end of survey***
- [ ] Yes
- [ ] Don't know
- [ ] Prefer not to answer

What is the name of the next prescription medication or over-the-counter drug that you take?

ME8a. [ ] Label product name:

____

[ ] Label generic name: ____

[ ] Don't know [ ] Prefer not to answer

ME8b. What is the dosage form?

Oral

- [ ] Pill, tablet, or capsule
- [ ] Sublingual or orally-disintegrating tablet
- [ ] Liquid solution or suspension (drink, syrup)
- [ ] Powder

Topical

- [ ] Liquid, cream, gel, or ointment
- [ ] Ear drops (otic)
- [ ] Eye drops (ophthalmic)
- [ ] Skin patch (transdermal)

Inhaled
☐ Inhaler or nebulizer

Injected
☐ Injection

Suppository
☐ Rectal (e.g., enema)
☐ Vaginal (e.g., douche, pessary)

Other:
☐ Don't know
☐ Prefer not to answer

ME8c. How frequently do you take it?
☐ _________ times per day
☐ _________ times per week
☐ _________ times per month
☐ Don't know
☐ Prefer not to answer

ME8d. What is the strength? *(Record strength of how it is actually taken, not how it is prescribed.)*
☐ _________ %
☐ _________ mg
☐ _________ mcg
☐ _________ grams
☐ _________ I.U.
☐ _________ Other unit: _____________
☐ Don't know
☐ Prefer not to answer

ME8d1. Total dosage **per day**. ***(Record strength of how it is actually taken, not how it is prescribed.)***
☐ _________ %
☐ _________ mg
☐ _________ mcg
☐ _________ grams
☐ _________ I.U.
☐ _________ Other unit: _____________
☐ Don't know
☐ Prefer not to answer

ME8e. Do you take it regularly or only as needed?
☐ Regularly
☐ Only as needed
☐ Don't know
☐ Prefer not to answer

ME8f. For how long have you been taking it?
☐ For _________ days
☐ For _________ weeks
☐ For _________ months
☐ For _________ years
☐ Don't know
☐ Prefer not to answer

ME8g. ***Interviewer comments***:
____

ME8h. Do you take any other prescription medications or over-the-counter drugs?

☐ No → ***skip to next section***
☐ Yes
☐ Don't know
☐ Prefer not to answer

What is the name of the next prescription medication or over-the-counter drug that you take?

ME9a. ☐ Label product name:
____

☐ Label generic name:
____

☐ Don't know ☐ Prefer not to answer

ME9b. What is the dosage form?

Oral
☐ Pill, tablet, or capsule
☐ Sublingual or orally-disintegrating tablet
☐ Liquid solution or suspension (drink, syrup)
☐ Powder

Topical
☐ Liquid, cream, gel, or ointment
☐ Ear drops (otic)
☐ Eye drops (ophthalmic)
☐ Skin patch (transdermal)

Inhaled
☐ Inhaler or nebulizer

Injected
☐ Injection

Suppository
☐ Rectal (e.g., enema)
☐ Vaginal (e.g., douche, pessary)

Other:
☐ Don't know
☐ Prefer not to answer

ME9c. How frequently do you take it?
☐ _________ times per day
☐ _________ times per week
☐ _________ times per month
☐ Don't know
☐ Prefer not to answer

ME9d. What is the strength? *(Record strength of how it is actually taken, not how it is prescribed.)*
☐ _________ %
☐ _________ mg
☐ _________ mcg
☐ _________ grams
☐ _________ I.U.
☐ _________ Other unit: _____________
☐ Don't know
☐ Prefer not to answer

ME9d1. Total dosage **per day.** ***(Record strength of how it is actually taken, not how it is prescribed.)***
☐ _________ %
☐ _________ mg
☐ _________ mcg
☐ _________ grams

☐ _________ I.U.
☐ _________ Other unit: _____________

☐ Don't know
☐ Prefer not to answer

ME9e. Do you take it regularly or only as needed?

☐ Regularly
☐ Only as needed

☐ Don't know
☐ Prefer not to answer

ME9f. For how long have you been taking it?

☐ For _________ days
☐ For _________ weeks
☐ For _________ months

☐ For _________ years
☐ Don't know
☐ Prefer not to answer

ME9g. ***Interviewer comments***:
____

ME9h. Do you take any other prescription medications or over-the-counter drugs?

☐ No → ***skip to next section***
☐ Yes

☐ Don't know
☐ Prefer not to answer

What is the name of the next prescription medication or over-the-counter drug that you take?

ME10a. ☐ Label product name: ____

☐ Label generic name: ____

☐ Don't know ☐ Prefer not to answer

ME10b. What is the dosage form?

Oral
☐ Pill, tablet, or capsule
☐ Sublingual or orally-disintegrating tablet
☐ Liquid solution or suspension (drink, syrup)
☐ Powder

Topical
☐ Liquid, cream, gel, or ointment
☐ Ear drops (otic)
☐ Eye drops (ophthalmic)
☐ Skin patch (transdermal)

Inhaled
☐ Inhaler or nebulizer

Injected
☐ Injection

Suppository
☐ Rectal (e.g., enema)
☐ Vaginal (e.g., douche, pessary)

Other:
☐ Don't know
☐ Prefer not to answer

ME10c. How frequently do you take it?
☐ _________ times per day
☐ _________ times per week
☐ _________ times per month
☐ Don't know
☐ Prefer not to answer

ME10d. What is the strength? ***(Record strength of how it is actually taken, not how it is prescribed.)***
☐ _________ %
☐ _________ mg
☐ _________ mcg
☐ _________ grams
☐ _________ I.U.
☐ _________ Other unit: ______________
☐ Don't know
☐ Prefer not to answer

ME10d1. Total dosage **per day**. ***(Record strength of how it is actually taken, not how it is prescribed.)***
☐ _________ %
☐ _________ mg
☐ _________ mcg
☐ _________ grams
☐ _________ I.U.
☐ _________ Other unit: ______________
☐ Don't know
☐ Prefer not to answer

ME10e. Do you take it regularly or only as needed?
☐ Regularly
☐ Only as needed
☐ Don't know
☐ Prefer not to answer

ME10f. For how long have you been taking it?
☐ For _________ days
☐ For _________ weeks
☐ For _________ months
☐ For _________ years
☐ Don't know
☐ Prefer not to answer

ME10g. ***Interviewer comments***:
____

ME10h. Do you take any other prescription medications or over-the-counter drugs?
☐ No → ***skip to end of survey***
☐ Yes
☐ Don't know
☐ Prefer not to answer

What is the name of the next prescription medication or over-the-counter drug that you take?

ME11a. ☐ Label product name: ______________________________

☐ Label generic name: ______________________________

☐ Don't know ☐ Prefer not to answer

ME11b. What is the dosage form?

Oral
☐ Pill, tablet, or capsule
☐ Sublingual or orally-disintegrating tablet
☐ Liquid solution or suspension (drink, syrup)
☐ Powder

Topical
☐ Liquid, cream, gel, or ointment
☐ Ear drops (otic)
☐ Eye drops (ophthalmic)
☐ Skin patch (transdermal)

Inhaled
☐ Inhaler or nebulizer

Injected
☐ Injection

Suppository
☐ Rectal (e.g., enema)
☐ Vaginal (e.g., douche, pessary)

Other:
☐ Don't know
☐ Prefer not to answer

ME11c. How frequently do you take it?
☐ _________ times per day
☐ _________ times per week
☐ _________ times per month
☐ Don't know
☐ Prefer not to answer

ME11d. What is the strength? ***(Record strength of how it is actually taken, not how it is prescribed.)***
☐ _________ %
☐ _________ mg
☐ _________ mcg
☐ _________ grams
☐ _________ I.U.
☐ _________ Other unit: _____________
☐ Don't know
☐ Prefer not to answer

ME11d1. Total dosage **per day**. ***(Record strength of how it is actually taken, not how it is prescribed.)***
☐ _________ %
☐ _________ mg
☐ _________ mcg
☐ _________ grams
☐ _________ I.U.
☐ _________ Other unit: _____________
☐ Don't know
☐ Prefer not to answer

ME11e. Do you take it regularly or only as needed?

- ☐ Regularly
- ☐ Only as needed
- ☐ Don't know
- ☐ Prefer not to answer

ME11f. For how long have you been taking it?

- ☐ For _________ days
- ☐ For _________ weeks
- ☐ For _________ months
- ☐ For _________ years
- ☐ Don't know
- ☐ Prefer not to answer

ME11g. ***Interviewer comments***:

____

ME11h. Do you take any other prescription medications or over-the-counter drugs?

- ☐ No → ***skip to end of survey***
- ☐ Yes
- ☐ Don't know
- ☐ Prefer not to answer

What is the name of the next prescription medication or over-the-counter drug that you take?

ME12a. ☐ Label product name: ____

☐ Label generic name: ____

☐ Don't know ☐ Prefer not to answer

ME12b. What is the dosage form?

Oral
- ☐ Pill, tablet, or capsule
- ☐ Sublingual or orally-disintegrating tablet
- ☐ Liquid solution or suspension (drink, syrup)
- ☐ Powder

Topical
- ☐ Liquid, cream, gel, or ointment
- ☐ Ear drops (otic)
- ☐ Eye drops (ophthalmic)
- ☐ Skin patch (transdermal)

Inhaled
- ☐ Inhaler or nebulizer

Injected
- ☐ Injection

Suppository
- ☐ Rectal (e.g., enema)
- ☐ Vaginal (e.g., douche, pessary)

Other:
- ☐ Don't know
- ☐ Prefer not to answer

ME12c. How frequently do you take it?

☐ _________ times per day
☐ _________ times per week
☐ _________ times per month
☐ Don't know
☐ Prefer not to answer

ME12d. What is the strength? ***(Record strength of how it is actually taken, not how it is prescribed.)***

☐ _________ %
☐ _________ mg
☐ _________ mcg
☐ _________ grams
☐ _________ I.U.
☐ _________ Other unit: _____________
☐ Don't know
☐ Prefer not to answer

ME12d1. Total dosage **per day.** ***(Record strength of how it is actually taken, not how it is prescribed.)***

☐ _________ %
☐ _________ mg
☐ _________ mcg
☐ _________ grams
☐ _________ I.U.
☐ _________ Other unit: _____________
☐ Don't know
☐ Prefer not to answer

ME12e. Do you take it regularly or only as needed?

☐ Regularly
☐ Only as needed
☐ Don't know
☐ Prefer not to answer

ME12f. For how long have you been taking it?

☐ For _________ days
☐ For _________ weeks
☐ For _________ months
☐ For _________ years
☐ Don't know
☐ Prefer not to answer

ME12g. ***Interviewer comments***:
____

ME12h. Do you take any other prescription medications or over-the-counter drugs?

☐ No → ***skip to end of survey***
☐ Yes
☐ Don't know
☐ Prefer not to answer

What is the name of the next prescription medication or over-the-counter drug that you take?

ME13a. ☐ Label product name: ____

☐ Label generic name: ____

☐ Don't know ☐ Prefer not to answer

ME13b. What is the dosage form?

Oral
- ☐ Pill, tablet, or capsule
- ☐ Sublingual or orally-disintegrating tablet
- ☐ Liquid solution or suspension (drink, syrup)
- ☐ Powder

Topical
- ☐ Liquid, cream, gel, or ointment
- ☐ Ear drops (otic)
- ☐ Eye drops (ophthalmic)
- ☐ Skin patch (transdermal)

Inhaled
- ☐ Inhaler or nebulizer

Injected
- ☐ Injection

Suppository
- ☐ Rectal (e.g., enema)
- ☐ Vaginal (e.g., douche, pessary)

Other:
- ☐ Don't know
- ☐ Prefer not to answer

ME13c. How frequently do you take it?
- ☐ _________ times per day
- ☐ _________ times per week
- ☐ _________ times per month
- ☐ Don't know
- ☐ Prefer not to answer

ME13d. What is the strength? *(Record strength of how it is actually taken, not how it is prescribed.)*
- ☐ _________ %
- ☐ _________ mg
- ☐ _________ mcg
- ☐ _________ grams
- ☐ _________ I.U.
- ☐ _________ Other unit: _____________
- ☐ Don't know
- ☐ Prefer not to answer

ME13d1. Total dosage **per day.** ***(Record strength of how it is actually taken, not how it is prescribed.)***
- ☐ _________ %
- ☐ _________ mg
- ☐ _________ mcg
- ☐ _________ grams
- ☐ _________ I.U.
- ☐ _________ Other unit: _____________
- ☐ Don't know
- ☐ Prefer not to answer

ME13e. Do you take it regularly or only as needed?
- ☐ Regularly
- ☐ Only as needed
- ☐ Don't know
- ☐ Prefer not to answer

ME13f. For how long have you been taking it?
- ☐ For _________ days
- ☐ For _________ weeks

☐ For _________ months ☐ Don't know
☐ For _________ years ☐ Prefer not to answer

ME13g. ***Interviewer comments***:
____

ME13h. Do you take any other prescription medications or over-the-counter drugs?
☐ No → ***skip to next section*** ☐ Don't know
☐ Yes ☐ Prefer not to answer

What is the name of the next prescription medication or over-the-counter drug that you take?

ME14a. ☐ Label product name: ____

☐ Label generic name: ____

☐ Don't know ☐ Prefer not to answer

ME14b. What is the dosage form?

Oral
☐ Pill, tablet, or capsule
☐ Sublingual or orally-disintegrating tablet
☐ Liquid solution or suspension (drink, syrup)
☐ Powder

Topical
☐ Liquid, cream, gel, or ointment
☐ Ear drops (otic)
☐ Eye drops (ophthalmic)
☐ Skin patch (transdermal)

Inhaled
☐ Inhaler or nebulizer

Injected
☐ Injection

Suppository
☐ Rectal (e.g., enema)
☐ Vaginal (e.g., douche, pessary)

Other:
☐ Don't know
☐ Prefer not to answer

ME14c. How frequently do you take it?
☐ _________ times per day ☐ Don't know
☐ _________ times per week ☐ Prefer not to answer
☐ _________ times per month

ME14d. What is the strength? ***Record strength of how it is actually taken, not how it is prescribed.)***
☐ _________ % ☐ _________ mcg
☐ _________ mg ☐ _________ grams

☐ _________ I.U.
☐ _________ Other unit: _____________
☐ Don't know
☐ Prefer not to answer

ME14d1. Total dosage **per day. *(Record strength of how it is actually taken, not how it is prescribed.)***

☐ _________ %
☐ _________ mg
☐ _________ mcg
☐ _________ grams
☐ _________ I.U.
☐ _________ Other unit: _____________
☐ Don't know
☐ Prefer not to answer

ME14e. Do you take it regularly or only as needed?

☐ Regularly
☐ Only as needed
☐ Don't know
☐ Prefer not to answer

ME14f. For how long have you been taking it?

☐ For _________ days
☐ For _________ weeks
☐ For _________ months
☐ For _________ years
☐ Don't know
☐ Prefer not to answer

ME14g. ***Interviewer comments*:**
____

ME14h. Do you take any other prescription medications or over-the-counter drugs?

☐ No → ***skip to end of survey***
☐ Yes
☐ Don't know
☐ Prefer not to answer

What is the name of the next prescription medication or over-the-counter drug that you take?

ME15a. ☐ Label product name: ____

☐ Label generic name: ____

☐ Don't know ☐ Prefer not to answer

ME15b. What is the dosage form?

Oral
☐ Pill, tablet, or capsule
☐ Sublingual or orally-disintegrating tablet
☐ Liquid solution or suspension (drink, syrup)
☐ Powder

☐ Liquid, cream, gel, or ointment
☐ Ear drops (otic)
☐ Eye drops (ophthalmic)
☐ Skin patch (transdermal)

Topical

Inhaled

☐ Inhaler or nebulizer

Injected

☐ Injection

Suppository

☐ Rectal (e.g., enema)

☐ Vaginal (e.g., douche, pessary)

Other:

☐ Don't know

☐ Prefer not to answer

ME15c. How frequently do you take it?

☐ _________ times per day

☐ _________ times per week

☐ _________ times per month

☐ Don't know

☐ Prefer not to answer

ME15d. What is the strength? *(Record strength of how it is actually taken, not how it is prescribed.)*

☐ _________ %

☐ _________ mg

☐ _________ mcg

☐ _________ grams

☐ _________ I.U.

☐ _________ Other unit: _____________

☐ Don't know

☐ Prefer not to answer

ME15d1. Total dosage **per day. *(Record strength of how it is actually taken, not how it is prescribed.)***

☐ _________ %

☐ _________ mg

☐ _________ mcg

☐ _________ grams

☐ _________ I.U.

☐ _________ Other unit: _____________

☐ Don't know

☐ Prefer not to answer

ME15e. Do you take it regularly or only as needed?

☐ Regularly

☐ Only as needed

☐ Don't know

☐ Prefer not to answer

ME15f. For how long have you been taking it?

☐ For _________ days

☐ For _________ weeks

☐ For _________ months

☐ For _________ years

☐ Don't know

☐ Prefer not to answer

ME15g. ***Interviewer comments*:**

____

ME15h. Do you take any other prescription medications or over-the-counter drugs?

☐ No → ***skip to next section***
☐ Yes
☐ Don't know
☐ Prefer not to answer

What is the name of the next prescription medication or over-the-counter drug that you take?

ME16a. ☐ Label product name: ______________________________

☐ Label generic name: ______________________________

☐ Don't know ☐ Prefer not to answer

ME16b. What is the dosage form?

Oral
☐ Pill, tablet, or capsule
☐ Sublingual or orally-disintegrating tablet
☐ Liquid solution or suspension (drink, syrup)
☐ Powder

Topical
☐ Liquid, cream, gel, or ointment
☐ Ear drops (otic)
☐ Eye drops (ophthalmic)
☐ Skin patch (transdermal)

Inhaled
☐ Inhaler or nebulizer

Injected
☐ Injection

Suppository
☐ Rectal (e.g., enema)
☐ Vaginal (e.g., douche, pessary)

Other:
☐ Don't know
☐ Prefer not to answer

ME16c. How frequently do you take it?
☐ _________ times per day
☐ _________ times per week
☐ _________ times per month
☐ Don't know
☐ Prefer not to answer

ME16d. What is the strength? *(Record strength of how it is actually taken, not how it is prescribed.)*
☐ _________ %
☐ _________ mg
☐ _________ mcg
☐ _________ grams
☐ _________ I.U.
☐ _________ Other unit: _____________
☐ Don't know
☐ Prefer not to answer

ME16d1. Total dosage **per day.** ***(Record strength of how it is actually taken, not how it is prescribed.)***
☐ _________ %
☐ _________ mg
☐ _________ mcg
☐ _________ grams

- ☐ _________ I.U.
- ☐ _________ Other unit: _____________
- ☐ Don’t know
- ☐ Prefer not to answer

ME16e. Do you take it regularly or only as needed?

- ☐ Regularly
- ☐ Only as needed
- ☐ Don’t know
- ☐ Prefer not to answer

ME16f. For how long have you been taking it?

- ☐ For _________ days
- ☐ For _________ weeks
- ☐ For _________ months
- ☐ For _________ years
- ☐ Don’t know
- ☐ Prefer not to answer

ME16g. ***Interviewer comments*:** _____________________________________
____
____

Self-Control

Using the scale provided, please indicate how much each of the following statements reflects how you typically are.

	Not at all	A little	Somewhat	Fair Amount	Very much
1. I am good at resisting temptation.	☐	☐	☐	☐	☐
2. I have a hard time breaking bad habits.	☐	☐	☐	☐	☐
3. I am lazy.	☐	☐	☐	☐	☐
4. I say inappropriate things.	☐	☐	☐	☐	☐
5. I do certain things that are bad for me, if they are fun.	☐	☐	☐	☐	☐
6. I refuse things that are bad for me.	☐	☐	☐	☐	☐
7. I wish I had more self-discipline.	☐	☐	☐	☐	☐
8. People would say that I have iron self-discipline.	☐	☐	☐	☐	☐
9. Pleasure and fun sometimes keep me from getting work done.	☐	☐	☐	☐	☐
10. I have trouble concentrating.	☐	☐	☐	☐	☐
11. I am able to work effectively toward long-term goals.	☐	☐	☐	☐	☐
12. Sometimes I can’t stop myself from doing something, even if I know it is wrong.	☐	☐	☐	☐	☐
13. I often act without thinking through all the alternatives.	☐	☐	☐	☐	☐

Sense of Control

The next few statements are about certain ways you may feel about your life. Please indicate how strongly you agree or disagree with each of the following statements: Strongly agree; Agree; Neutral, Disagree, or Strongly disagree

	Strongly agree	Agree	Neutral	Disagree	Strongly disagree	Don't know	Prefer not to answer
**SE1**. I have little control over the things that happen to me.	☐	☐	☐	☐	☐	☐	☐
**SE2**. There is really no way I can solve some of the problems I have.	☐	☐	☐	☐	☐	☐	☐
**SE3**. There is little I can do to change many of the important things in my life.	☐	☐	☐	☐	☐	☐	☐
**SE4**. I often feel helpless in dealing with the problems of life.	☐	☐	☐	☐	☐	☐	☐
**SE5**. Sometimes I feel that I am being pushed around in life.	☐	☐	☐	☐	☐	☐	☐
**SE6**. What happens to me in the future mostly depends on me.	☐	☐	☐	☐	☐	☐	☐
**SE7**. I can do just about anything I really set my mind to do.	☐	☐	☐	☐	☐	☐	☐

Self-Compassion

Please read each statement carefully before answering. To the left of each item, indicate how often you behave in the stated manner, using the following scale:

0	1	2	3	4
Almost never				Almost always

_____1. When I fail at something important to me I become consumed by feelings of inadequacy.

_____2. I try to be understanding and patient towards those aspects of my personality I don't like.

_____3. When something painful happens I try to take a balanced view of the situation.

_____4. When I'm feeling down, I tend to feel like most other people are probably happier than I am.

_____5. I try to see my failings as part of the human condition.

_____6. When I'm going through a very hard time, I give myself the caring and tenderness I need.

_____7. When something upsets me I try to keep my emotions in balance.

_____8. When I fail at something that's important to me, I tend to feel alone in my failure

_____9. When I'm feeling down I tend to obsess and fixate on everything that's wrong.

_____10. When I feel inadequate in some way, I try to remind myself that feelings of inadequacy are shared by most people.

_____11. I'm disapproving and judgmental about my own flaws and inadequacies.

_____12. I'm intolerant and impatient towards those aspects of my personality I don't like.

CES-D Depression Inventory

For each statement, please place a mark in the column that best describes how you have been feeling *in the past week*.

	Rarely or none of the time (less than 1 day)	Some or a little of the time (1 – 2 days)	Occasionally or a moderate amount of the time (3 – 4 days)	Most or all of the time (5 – 7 days)
C1. I was bothered by things that usually don't bother me.	☐	☐	☐	☐
C2. I did not feel like eating; my appetite was poor.	☐	☐	☐	☐
C3. I felt that I could not shake off the blues, even with the help from family or friends.	☐	☐	☐	☐
C4. I felt that I was just as good as other people.	☐	☐	☐	☐
C5. I had trouble keeping my mind on what I was doing.	☐	☐	☐	☐
C6. I felt depressed.	☐	☐	☐	☐
C7. I felt that everything I did was an effort.	☐	☐	☐	☐
C8. I felt hopeful about the future.	☐	☐	☐	☐
C9. I thought my life had been a failure.	☐	☐	☐	☐
C10. I felt fearful.	☐	☐	☐	☐
C11. My sleep was restless.	☐	☐	☐	☐
C12. I was happy.	☐	☐	☐	☐
C13. I talked less than usual.	☐	☐	☐	☐
C14. I felt lonely.	☐	☐	☐	☐

	Rarely or none of the time (less than 1 day)	Some or a little of the time (1 – 2 days)	Occasionally or a moderate amount of the time (3 – 4 days)	Most or all of the time (5 – 7 days)
C15. People were unfriendly.	☐	☐	☐	☐
C16. I enjoyed life.	☐	☐	☐	☐
C17. I had crying spells.	☐	☐	☐	☐
C18. I felt sad.	☐	☐	☐	☐
C19. I felt that people dislike me.	☐	☐	☐	☐
C20. I could not get “going”.	☐	☐	☐	☐

Perceived Stress Scale

The questions in this scale ask you about your feelings and thoughts during the last month. In each case, you will be asked to indicate by circling how often you felt or thought a certain way.

	Never	Almost Never	Sometimes	Fairly Often	Often
P1. In the last month, how often have you been upset because of something that happened unexpectedly?	☐	☐	☐	☐	☐
P2. In the last month, how often have you felt that you were unable to control the important things in your life?	☐	☐	☐	☐	☐
P3. In the last month, how often have you felt nervous and "stressed"?	☐	☐	☐	☐	☐
P4. In the last month, how often have you felt confident about your ability to handle your personal problems?	☐	☐	☐	☐	☐
P5. In the last month, how often have you felt that things were going your way?	☐	☐	☐	☐	☐
P6. In the last month, how often have you found that you could not cope with all the things that you had to do?	☐	☐	☐	☐	☐
P7. In the last month, how often have you been able to control irritations in your life?	☐	☐	☐	☐	☐
P8. In the last month, how often have you felt that you were on top of things?	☐	☐	☐	☐	☐
P9. In the last month, how often have you been angered because of things that were outside of your control?	☐	☐	☐	☐	☐
P10. In the last month, how often have you felt difficulties were piling up so high that you could not overcome them?	☐	☐	☐	☐	☐

Mindfulness

Instructions: Below is a collection of statements about your everyday experience. Using the scale below, please indicate how frequently or infrequently you currently have each experience. Please answer according to what really reflects your experience rather than what you think your experience should be. Please treat each item separately from every other item.

Please indicate the degree to which you agree with each of the following items using the scale below. Simply check your response to each item

	**Almost always**	**Very frequently**	**Somewhat frequently**	**Somewhat infrequently**	**Very infrequently**	**Almost never**	**I do not know**	**I prefer not to answer**
Y5. I could be experiencing some emotion and not be conscious of it until some time later.	☐	☐	☐	☐	☐	☐	☐	☐
Y6. I break or spill things because of carelessness, not paying attention, or thinking of something else.	☐	☐	☐	☐	☐	☐	☐	☐
Y7. I find it difficult to stay focused on what's happening in the present.	☐	☐	☐	☐	☐	☐	☐	☐
Y8. I tend to walk quickly to get where I'm going without paying attention to what I experience along the way.	☐	☐	☐	☐	☐	☐	☐	☐
Y9. I tend not to notice feelings of physical tension or discomfort until they really grab my attention.	☐	☐	☐	☐	☐	☐	☐	☐

	Almost always	Very frequently	Somewhat frequently	Somewhat infrequently	Very infrequently	Almost never	I do not know	I prefer not to answer
Y10. It seems I am "running on automatic" without much awareness of what I'm doing.	☐	☐	☐	☐	☐	☐	☐	☐
Y11. I rush through activities without being really attentive to them.	☐	☐	☐	☐	☐	☐	☐	☐
Y12. I get so focused on the goal I want to achieve that I lose touch with what I am doing right now to get there.	☐	☐	☐	☐	☐	☐	☐	☐
Y13. I do jobs or tasks automatically, without being aware of what I'm doing.	☐	☐	☐	☐	☐	☐	☐	☐
Y14. I find myself listening to someone with one ear, doing something else at the same time.	☐	☐	☐	☐	☐	☐	☐	☐
Y15. I drive places on "automatic pilot" and then wonder why I went there.	☐	☐	☐	☐	☐	☐	☐	☐
Y16. I find myself preoccupied with the future or the past.	☐	☐	☐	☐	☐	☐	☐	☐
Y17. I find myself doing things without paying attention.	☐	☐	☐	☐	☐	☐	☐	☐

	Almost always	Very frequently	Somewhat frequently	Somewhat infrequently	Very infrequently	Almost never	I do not know	I prefer not to answer
Y18. I snack without being aware that I'm eating.	☐	☐	☐	☐	☐	☐	☐	☐

Please rate each of the following statements using the scale provided. Write the number in the blank that best describes *your own opinion* of what is *generally true for you*.

	Never	Almost Never	Sometimes	Fairly Often	Often
F1. When I'm walking, I deliberately notice the sensations of my body moving.	☐	☐	☐	☐	☐
F2. I'm good at finding words to describe my feelings.	☐	☐	☐	☐	☐
F3. I criticize myself for having irrational or inappropriate emotions.	☐	☐	☐	☐	☐
F4. I perceive my feelings and emotions without having to react to them.	☐	☐	☐	☐	☐
F5. When I do things, my mind wanders off and I'm easily distracted.	☐	☐	☐	☐	☐
F6. When I take a shower or bath, I stay alert to the sensations of water on my body.	☐	☐	☐	☐	☐
F7. I can easily put my beliefs, opinions, and expectations into words.	☐	☐	☐	☐	☐
F8. I don't pay attention to what I'm doing because I'm daydreaming, worrying, or otherwise distracted.	☐	☐	☐	☐	☐
F9. I watch my feelings without getting lost in them.	☐	☐	☐	☐	☐
F10. I tell myself I shouldn't be feeling the way I'm feeling.	☐	☐	☐	☐	☐
F11. I notice how foods and drinks affect my thoughts, bodily sensations, and emotions.	☐	☐	☐	☐	☐
F12. It's hard for me to find the words to describe what I'm thinking.	☐	☐	☐	☐	☐

	Never	Almost Never	Sometimes	Fairly Often	Often
F13. I am easily distracted.	☐	☐	☐	☐	☐
F14. I believe some of my thoughts are abnormal or bad and I shouldn't think that way.	☐	☐	☐	☐	☐
F15. I pay attention to sensations, such as the wind in my hair or sun on my face.	☐	☐	☐	☐	☐
F16. I have trouble thinking of the right words to express how I feel about things.	☐	☐	☐	☐	☐
F17. I make judgments about whether my thoughts are good or bad.	☐	☐	☐	☐	☐
F18. I find it difficult to stay focused on what's happening in the present.	☐	☐	☐	☐	☐
F19. When I have distressing thoughts or images, I "step back" and am aware of the thought or image without getting taken over by it.	☐	☐	☐	☐	☐
F20. I pay attention to sounds, such as clocks ticking, birds chirping, or cars passing.	☐	☐	☐	☐	☐
F21. In difficult situations, I can pause without immediately reacting.	☐	☐	☐	☐	☐
F22. When I have a sensation in my body, it's difficult for me to describe it because I can't find the right words.	☐	☐	☐	☐	☐
F23. It seems I am "running on automatic" without much aware- ness of what I'm doing.	☐	☐	☐	☐	☐
F24. When I have distressing thoughts or images, I feel calm soon after.	☐	☐	☐	☐	☐
F25. I tell myself that I shouldn't be thinking the way I'm thinking.	☐	☐	☐	☐	☐

	Never	Almost Never	Sometimes	Fairly Often	Often
F26. I notice the smells and aromas of things.	☐	☐	☐	☐	☐
F27. Even when I'm feeling terribly upset, I can find a way to put it into words.	☐	☐	☐	☐	☐
F28. I rush through activities without being really attentive to them.	☐	☐	☐	☐	☐
F29. When I have distressing thoughts or images, I am able just to notice them without reacting.	☐	☐	☐	☐	☐
F30. I think some of my emotions are bad or inappropriate and I shouldn't feel them.	☐	☐	☐	☐	☐
F31. I notice visual elements in art or nature, such as colors, shapes, textures, or patterns of light and shadow.	☐	☐	☐	☐	☐
F32. My natural tendency is to put my experiences into words.	☐	☐	☐	☐	☐
F33. When I have distressing thoughts or images, I just notice them and let them go.	☐	☐	☐	☐	☐
F34. I do jobs or tasks automatically without being aware of what I'm doing.	☐	☐	☐	☐	☐
F35. When I have distressing thoughts or images, I judge myself as good or bad depending what the thought or image is about.	☐	☐	☐	☐	☐
F36. I pay attention to how my emotions affect my thoughts and behavior.	☐	☐	☐	☐	☐
F37. I can usually describe how I feel at the moment in consider- able detail.	☐	☐	☐	☐	☐

	Never	Almost Never	Someti mes	Fairly Often	Often
F38. I find myself doing things without paying attention.	☐	☐	☐	☐	☐
F39. I disapprove of myself when I have irrational ideas.	☐	☐	☐	☐	☐

This Week's Experiences

Instructions: Below is a collection of statements about how you find yourself reacting to unpleasant thoughts and feelings. Using the scale below, please indicate how frequently or infrequently you experienced each of the following reactions this week**. Please answer according to what *really reflects your experience this week* rather than what you think your experience should be.**

This week, when I encountered an unpleasant thought or feeling,

	Almost Never	Very Infrequently	Somewhat Infrequently	Somewhat Frequently	Very Frequently	Almost Always
1. I tried to think pleasant thoughts instead	☐	☐	☐	☐	☐	☐
2. I allowed myself to experience it	☐	☐	☐	☐	☐	☐
3. I got angry or upset at myself for having the thought/emotion	☐	☐	☐	☐	☐	☐
4. I felt the desire to make it better or make it go away	☐	☐	☐	☐	☐	☐
5. I wished I didn't feel or think that way	☐	☐	☐	☐	☐	☐
6. I tried to let my thoughts just come and go without getting too entangled with them	☐	☐	☐	☐	☐	☐
7. I thought the mood would never change	☐	☐	☐	☐	☐	☐
8. I explored the body sensations that accompanied the emotion.	☐	☐	☐	☐	☐	☐
9. I named the emotion over and over as long as it lasted	☐	☐	☐	☐	☐	☐
10. I turned to work or other activities to take my mind off things.	☐	☐	☐	☐	☐	☐
11. I named the emotion and then redirected my attention to the present moment	☐	☐	☐	☐	☐	☐
12. I thought "This mood, too, shall pass"	☐	☐	☐	☐	☐	☐

	Almost Never	Very Infrequently	Somewhat Infrequently	Somewhat Frequently	Very Frequently	Almost Always
13. I focused on my breathing	☐	☐	☐	☐	☐	☐
14. I took an active interest in how the experience changed over time	☐	☐	☐	☐	☐	☐
15. I explored my reactions to the thought or emotion	☐	☐	☐	☐	☐	☐
16. I thought "my thinking is being distorted by my mood"	☐	☐	☐	☐	☐	☐
17. Think "Why do I have problems that other people don't have?"	☐	☐	☐	☐	☐	☐
18. I forgave myself for having the thought/emotion	☐	☐	☐	☐	☐	☐
19. I challenged the thought's validity	☐	☐	☐	☐	☐	☐
20. I asked myself questions about the experience in order to explore it better	☐	☐	☐	☐	☐	☐
21. I tried not to change it because I believe it is important to experience	☐	☐	☐	☐	☐	☐
22. I labelled my thoughts "images" or verbal "talk"	☐	☐	☐	☐	☐	☐
23. I thought "Why do I always react this way?"	☐	☐	☐	☐	☐	☐
24. I adopted a welcoming stance toward the emotion/thought	☐	☐	☐	☐	☐	☐
25. I went somewhere alone to think about my feelings	☐	☐	☐	☐	☐	☐
26. I sent out compassion to all people who struggle with this emotion	☐	☐	☐	☐	☐	☐
27. I restrained myself from doing anything too quickly	☐	☐	☐	☐	☐	☐

	Almost Never	Very Infrequently	Somewhat Infrequently	Somewhat Frequently	Very Frequently	Almost Always
28. I became genuinely curious about the thought/emotion	☐	☐	☐	☐	☐	☐
29. I laughed or kidded myself about the situation	☐	☐	☐	☐	☐	☐
30. I treated the repetitive thought like a "top ten radio tune" that's playing in the background	☐	☐	☐	☐	☐	☐
31. I talked to others about how I was feeling	☐	☐	☐	☐	☐	☐
32. I tried to forget about it	☐	☐	☐	☐	☐	☐
33. I thought "Why can't I handle things better"	☐	☐	☐	☐	☐	☐
34. I tried to come up with a way to make it go away	☐	☐	☐	☐	☐	☐
35. I said to myself "thinking" or "this is just a thought"	☐	☐	☐	☐	☐	☐
36. I thought about how much I disliked feeling that way	☐	☐	☐	☐	☐	☐
37. I reminded myself thoughts are not accurate reflections of reality	☐	☐	☐	☐	☐	☐
38. I thought that that I must be headed into a downward spiral	☐	☐	☐	☐	☐	☐
39. I thought "What I am doing to deserve this?	☐	☐	☐	☐	☐	☐

Emotion Regulation

Please indicate how often the following statements apply to you by checking the box that best describes your experience.

	Almost Never (0-10%)	Sometimes (11-35%)	About Half The Time (36-65%)	Most of the Time (66-90%)	Almost Always (91-100%)
1. I am clear about my feelings.	☐	☐	☐	☐	☐
2. I pay attention to how I feel.	☐	☐	☐	☐	☐
3. I experience my emotions as overwhelming and out of control.	☐	☐	☐	☐	☐
4. I have no idea how I am feeling.	☐	☐	☐	☐	☐
5. I have difficulty making sense out of my feelings.	☐	☐	☐	☐	☐
6. I am attentive to my feelings.	☐	☐	☐	☐	☐
7. I know exactly how I am feeling.	☐	☐	☐	☐	☐
8. I care about what I am feeling.	☐	☐	☐	☐	☐
9. I am confused about how I feel.	☐	☐	☐	☐	☐
10. When I'm upset, I acknowledge my emotions.	☐	☐	☐	☐	☐
11. When I'm upset, I become angry with myself for feeling that way.	☐	☐	☐	☐	☐
12. When I'm upset, I become embarrassed for feeling that way.	☐	☐	☐	☐	☐
13. When I'm upset, I have difficulty getting work done.	☐	☐	☐	☐	☐
14. When I'm upset, I become out of control.	☐	☐	☐	☐	☐
15. When I'm upset, I believe that I will remain that way for a long time.	☐	☐	☐	☐	☐
16. When I'm upset, I believe that I will end up feeling very depressed.	☐	☐	☐	☐	☐
17. When I'm upset, I believe that my feelings are valid and important.	☐	☐	☐	☐	☐
18. When I'm upset, I have difficulty focusing on other things.	☐	☐	☐	☐	☐
19. When I'm upset, I feel out of control.	☐	☐	☐	☐	☐
20. When I'm upset, I can still get things done.	☐	☐	☐	☐	☐
21. When I'm upset, I feel ashamed at myself for feeling that way.	☐	☐	☐	☐	☐
22. When I'm upset, I know that I can find a way to eventually feel better.	☐	☐	☐	☐	☐

	Almost Never (0-10%)	Sometimes (11-35%)	About Half The Time (36-65%)	Most of the Time (66-90%)	Almost Always (91-100%)
23. When I'm upset, I feel like I am weak.	☐	☐	☐	☐	☐
24. When I'm upset, I feel like I can remain in control of my behaviours.	☐	☐	☐	☐	☐
25. When I'm upset, I feel guilty for feeling that way.	☐	☐	☐	☐	☐
26. When I'm upset, I have difficulty concentrating.	☐	☐	☐	☐	☐
27. When I'm upset, I have difficulty controlling my behaviours.	☐	☐	☐	☐	☐
28. When I'm upset, I believe there is nothing I can do to make myself feel better.	☐	☐	☐	☐	☐
29. When I'm upset, I become irritated at myself for feeling that way.	☐	☐	☐	☐	☐
30. When I'm upset, I start to feel very bad about myself.	☐	☐	☐	☐	☐
31. When I'm upset, I believe that wallowing in it is all I can do.	☐	☐	☐	☐	☐
32. When I'm upset, I lose control over my behaviour.	☐	☐	☐	☐	☐
33. When I'm upset, I have difficulty thinking about anything else.	☐	☐	☐	☐	☐
34. When I'm upset I take time to figure out what I'm really feeling.	☐	☐	☐	☐	☐
35. When I'm upset, it takes me a long time to feel better.	☐	☐	☐	☐	☐
36. When I'm upset, my emotions feel overwhelming.	☐	☐	☐	☐	☐

Beck Anxiety Inventory

Below is a list of common symptoms of anxiety. Please carefully read each item in the list. Indicate how much you have been bothered by that symptom during the past month, including today, by circling the number in the corresponding space in the column next to each symptom.

	Not At All	Mildly – it didn't bother me much	Moderately – it wasn't pleasant at all times	Severely – it bothered me a lot
Numbness or tingling	☐	☐	☐	☐
Feeling hot	☐	☐	☐	☐
Wobbliness in legs	☐	☐	☐	☐
Unable to relax	☐	☐	☐	☐
Fear of worst happening	☐	☐	☐	☐
Dizzy or lightheaded	☐	☐	☐	☐
Heart pounding/racing	☐	☐	☐	☐
Unsteady	☐	☐	☐	☐
Terrified or afraid	☐	☐	☐	☐
Nervous	☐	☐	☐	☐
Feeling of choking	☐	☐	☐	☐
Hands trembling	☐	☐	☐	☐
Shaky/unsteady	☐	☐	☐	☐
Fear of losing control	☐	☐	☐	☐
Difficulty in breathing	☐	☐	☐	☐
Fear of dying	☐	☐	☐	☐
Scared	☐	☐	☐	☐
Indigestion	☐	☐	☐	☐
Faint/lightheaded	☐	☐	☐	☐
Face flushed	☐	☐	☐	☐
Hot/cold sweats	☐	☐	☐	☐

Parent’s Education

1. Please check the box beside the highest grade or degree that your BIOLOGICAL MOTHER completed.

Never went to school	☐
Grades 1 to 3	☐
Grades 4 to 8	☐
Grades 9 to 11	☐
Grade 12	☐
GED	☐
One or more years of Vocational or Professional School after High School	☐
One or more years of College	☐
One or more years of Graduate or Professional School after College	☐
I don’t know	☐
I prefer not to answer	☐

2. Please check the box beside the highest grade or degree that your BIOLOGICAL FATHER completed.

Never went to school	☐
Grades 1 to 3	☐
Grades 4 to 8	☐
Grades 9 to 11	☐
Grade 12	☐
GED	☐
One or more years of Vocational or Professional School after High School	☐
One or more years of College	☐
One or more years of Graduate or Professional School after College	☐
I don’t know	☐
I prefer not to answer	☐

Now please think of the two most important adults in your home between the time you were born and age 18 years. Please check the category below that best described their lecel of education during this time period.

3. First adult's highest level of education:

Never went to school	☐
Grades 1 to 3	☐
Grades 4 to 8	☐
Grades 9 to 11	☐
Grade 12	☐
GED	☐
One or more years of Vocational or Professional School after High School	☐
One or more years of College	☐
One or more years of Graduate or Professional School after College	☐
I don't know	☐
I prefer not to answer	☐

4. Second adult's highest level of education

Never went to school	☐
Grades 1 to 3	☐
Grades 4 to 8	☐
Grades 9 to 11	☐
Grade 12	☐
GED	☐
One or more years of Vocational or Professional School after High School	☐
One or more years of College	☐
One or more years of Graduate or Professional School after College	☐
I don't know	☐
I prefer not to answer	☐

Your Childhood Experiences

The following questions ask about some difficult experiences that you might have had as a child. These questions may be emotionally difficult to answer. Just as a reminder, you do not need answer any questions that you would prefer not to. Your answers to these questions, as with all questions, will remain confidential.

C1. Before you were 18 years old, did a parent or other adult in the household often or very often…

Swear at you, insult you, put you down, or humiliate you?
or
Act in a way that made you afraid that you might be physically hurt?

□ No
□ Yes
□ I do not know
□ I prefer not to answer

C2. Before you were 18 years old, did a parent or other adult in the household often or very often…

Push, grab, slap, or throw something at you?
or
Ever hit you so hard that you had marks or were injured?

□ No
□ Yes
□ I do not know
□ I prefer not to answer

C3. Before you were 18 years old, did an adult or person at least 5 years older than you ever…

Touch or fondle you or have you touch their body in a sexual way?
or
Attempt or actually have oral, anal, or vaginal intercourse with you?

□ No
□ Yes
□ I do not know
□ I prefer not to answer

C4. Before you were 18 years old, did you often or very often feel that …

No one in your family loved you or thought you were important or special?
or
Your family didn't look out for each other, feel close to each other, or support each other?

□ No
□ Yes
□ I do not know
□ I prefer not to answer

C5. Before you were 18 years old, did you often or very often feel that …

You didn't have enough to eat, had to wear dirty clothes, and had no one to protect you?
or
Your parents were too drunk or high to take care of you or take you to the doctor if you needed it?

□ No
□ Yes
□ I do not know
□ I prefer not to answer

C6. Before you were 18 years old, was a biological parent ever lost to you through divorce, abandonment, or other reason?

□ No
□ Yes
□ I do not know
□ I prefer not to answer

C7. Before you were 18 years old, was your mother or stepmother:

Often or very often pushed, grabbed, slapped, or had something thrown at her?
or
Sometimes, often, or very often kicked, bitten, hit with a fist, or hit with something hard?
or
Ever repeatedly hit over at least a few minutes or threatened with a gun or knife?

□ No
□ Yes
□ I do not know
□ I prefer not to answer

C8. Before you were 18 years old, did you live with anyone who was a problem drinker or alcoholic, or who used street drugs?

- □ No
- □ Yes
- □ I do not know
- □ I prefer not to answer

C9. Before you were 18 years old, was a household member depressed or mentally ill, or did a household member attempt suicide?

- □ No
- □ Yes
- □ I do not know
- □ I prefer not to answer

C10. Before you were 18 years old, did a household member go to prison?

- □ No
- □ Yes
- □ I do not know
- □ I prefer not to answer

About You

Y1. How many close friends do you have? By close friends, we mean people that you feel at ease with, and that you can talk to about private matters.

□ None → ***skip to Y2***

____ Number of close friends

□ I do not know

□ I prefer not to answer

Y1a. How many of these close friends do you see at least once a month?

____ Number of close friends ***(if none, write zero)***

□ I do not know

□ I prefer not to answer

Y2. Thinking about your relatives, how many relatives do you feel at ease with, and feel that you can talk to about private matters?

□ None → ***skip to Y3***

____ Number of relatives

□ I do not know

□ I prefer not to answer

Y2a. How many of these relatives do you see at least once a month?

____ Number of relatives ***(if none, write zero)***

□ I do not know

□ I prefer not to answer

Y3. About how often do you participate in groups or clubs, such as religious connected groups, self-help groups, charities, or a public service or community group.

☐ Never or almost never
☐ A few times a year
☐ Once or twice a month
☐ Once a week
☐ More than once a week
☐ I do not know
☐ I prefer not to answer

Y4. About how often do you go to religious meetings or services?

☐ Never or almost never
☐ A few times a year
☐ Once or twice a month
☐ Once a week
☐ More than once a week
☐ I do not know
☐ I prefer not to answer

Loneliness

INSTRUCTIONS: Indicate how often each of the statements below is descriptive of you.

**Statement**	Never	Rarely	Sometimes	Often
1. I feel in tune with the people around me	☐	☐	☐	☐
2. I lack companionship	☐	☐	☐	☐
3. There is no one I can turn to	☐	☐	☐	☐
4. I do not feel alone	☐	☐	☐	☐
5. I feel part of a group of friends	☐	☐	☐	☐
6. I have a lot in common with the people around me	☐	☐	☐	☐
7. I am no longer close to anyone	☐	☐	☐	☐
8. My interests and ideas are not shared by those around me	☐	☐	☐	☐
9. I am an outgoing person	☐	☐	☐	☐
10. There are people I feel close to	☐	☐	☐	☐
11. I feel left out	☐	☐	☐	☐
12. My social relationships are superficial	☐	☐	☐	☐
13. No one really knows me well	☐	☐	☐	☐
14. I feel isolated from others	☐	☐	☐	☐
15. I can find companionship when I want it	☐	☐	☐	☐
16. There are people who really understand me	☐	☐	☐	☐
17. I am unhappy being so withdrawn	☐	☐	☐	☐
18. People are around me but not with me	☐	☐	☐	☐
19. There are people I can talk to	☐	☐	☐	☐
20. There are people I can turn to	☐	☐	☐	☐

Your Sleep

The following questions relate to your usual sleep habits during the past month only. Your answers should indicate the most accurate reply for the majority of days and nights in the past month. ***Please answer all questions.***

S1. **During the past month,** what time have you usually gone to bed at night?

BED TIME ___________ AM / PM

□ I do not know
□ I prefer not to answer

S2. **During the past month**, how long (in minutes) has it usually taken you to fall asleep each night?

NUMBER OF MINUTES ___________

□ I do not know
□ I prefer not to answer

S3. During the past month, at what time have you usually gotten up in the morning?

GETTING UP TIME ___________ AM / PM

□ I do not know
□ I prefer not to answer

S4. During the past month, how many hours of actual sleep did you get on average at night? (This may be different than the number of hours you spent in bed.)

AVERAGE HOURS OF SLEEP PER NIGHT ___________

□ I do not know
□ I prefer not to answer

For each of the remaining questions, check the one best response. Please answer all questions.

During the past month, how often have you had trouble sleeping because you…………..

S5. …..Could not get to sleep within 30 minutes?

□ Not during the past month
□ Less than once a week
□ Once or twice a week
□ Three or more times a week
□ I do not know
□ I prefer not to answer

S6. …..Woke up in the middle of the night or early morning?

□ Not during the past month
□ Less than once a week
□ Once or twice a week
□ Three or more times a week
□ I do not know
□ I prefer not to answer

S7. …..Had to get up to use the bathroom?

□ Not during the past month
□ Less than once a week
□ Once or twice a week
□ Three or more times a week
□ I do not know
□ I prefer not to answer

S8. …..Could not breathe comfortably?

- ☐ Not during the past month
- ☐ Less than once a week
- ☐ Once or twice a week
- ☐ Three or more times a week
- ☐ I do not know
- ☐ I prefer not to answer

S9. …..Coughed or snored loudly?

- ☐ Not during the past month
- ☐ Less than once a week
- ☐ Once or twice a week
- ☐ Three or more times a week
- ☐ I do not know
- ☐ I prefer not to answer

S10. …..Felt too cold?

- ☐ Not during the past month
- ☐ Less than once a week
- ☐ Once or twice a week
- ☐ Three or more times a week
- ☐ I do not know
- ☐ I prefer not to answer

S11. …..Felt too hot?

- ☐ Not during the past month
- ☐ Less than once a week
- ☐ Once or twice a week
- ☐ Three or more times a week
- ☐ I do not know
- ☐ I prefer not to answer

S12. …..Had bad dreams?

□ Not during the past month
□ Less than once a week
□ Once or twice a week
□ Three or more times a week
□ I do not know
□ I prefer not to answer

S13. …..Had pain?

□ Not during the past month
□ Less than once a week
□ Once or twice a week
□ Three or more times a week
□ I do not know
□ I prefer not to answer

S14. Other reason(s), **please describe:** ____

S14a. How often during the past month have you had trouble sleeping because of this?

□ Not during the past month
□ Less than once a week
□ Once or twice a week
□ Three or more times a week
□ I do not know
□ I prefer not to answer

S15. **During the past month**, how would you rate your sleep quality overall?

□ Very good
□ Fairly good
□ Fairly bad
□ Very bad
□ I do not know
□ I prefer not to answer

S17. **During the past month**, how often have you taken medicine to help you sleep (prescribed or "over the counter")?

□ Not during the past month
□ Less than once a week
□ Once or twice a week
□ Three or more times a week
□ I do not know
□ I prefer not to answer

S18. **During the past month**, how often have you had trouble staying awake while driving, eating meals, or engaging in social activity?

□ Not during the past month
□ Less than once a week
□ Once or twice a week
□ Three or more times a week
□ I do not know
□ I prefer not to answer

S19. **During the past month**, how much of a problem has it been for you to keep up enough enthusiasm to get things done?

□ No problem at all
□ Somewhat of a problem
□ A very big problem
□ I do not know
□ I prefer not to answer

S20. Do you have a bed partner or a room-mate in the same room ?

□ No bed partner or roommate in the same room → ***skip to next survey section:***
□ Partner in same bed
□ Room-mate in the same room
□ I do not know
□ I prefer not to answer

If you have a roommate or bed partner, ask him/her how often in the past month you have had . . . .

S21. …..Loud snoring

- ☐ Not during the past month
- ☐ Less than once a week
- ☐ Once or twice a week
- ☐ Three or more times a week
- ☐ Unable to ask him/her
- ☐ he/she does not know
- ☐ he/she prefers not to answer

S22. ……Long pauses between breaths while asleep

- ☐ Not during the past month
- ☐ Less than once a week
- ☐ Once or twice a week
- ☐ Three or more times a week
- ☐ Unable to ask him/her
- ☐ he/she does not know
- ☐ he/she prefers not to answer

S23. …..Legs twitching or jerking while you sleep

- ☐ Not during the past month
- ☐ Less than once a week
- ☐ Once or twice a week
- ☐ Three or more times a week
- ☐ Unable to ask him/her
- ☐ he/she does not know
- ☐ he/she prefers not to answer

S24. …..Episodes of disorientation or confusion during sleep

□ Not during the past month
□ Less than once a week
□ Once or twice a week
□ Three or more times a week
□ Unable to ask him/her
□ he/she does not know
□ he/she prefers not to answer

S25. ……Other restlessness while you sleep; ***please describe:***

____

S25a. □ Not during the past month
□ Less than once a week
□ Once or twice a week
□ Three or more times a week
□ Unable to ask him/her
□ he/she does not know
□ he/she prefers not to answer

FOCUS GROUP TOPICS

Focus Group Topics

- How is your experience of the class length? Does it seem about right, or would you prefer it longer or shorter? – e.g. length of each class (2.5 hours vs. 1 hour), 9 week duration
- Which modules did you find worked the best for you?
- Are there topics or modules you think would help improve the course?
- Are there areas we should expand?
- Are there areas we should reduce?
- Describe different modules, and ask how well they worked, specifically what they liked, didn't like, and recommendations for what to change and what to keep the same.
    - Hypertension education in the orientation session
    - Class 2: Highly palatable snack practice
    - Class 2: Home practice related to pleasant events calendar on eating and alcohol consumption
    - Class 3: Exploration of physical activity including discussion in class, and home practice of performing physical activity.
    - Class 4: 15 min physical activity module followed by discussion .
    - Class 4: Motivational interviewing module related to diet, alcohol consumption and physical activity
    - Class 4-7: Home practice related to goal for diet, alcohol consumption, physical activity or diet.
    - Class 5: Exploring relationship with medication adherence
    - Class 6: Social support module
    - All-Day: Silent exercise module
    - Booster sessions

QUESTIONNAIRES ANSWERED BY PARTICIPANTS AT 9 WEEKS, 3 MONTHS AND 6 MONTHS FOLLOW-UP

Questionnaires administered at 9 weeks, 3 months and 6 months follow-up are identical to those administered at baseline, with the exception that questionnaires for which the answers should not change or be informative (i.e. introductory questions, age, race/ethnicity, education, childhood experiences) are not given at follow-ups.

MINDFULNESS-BASED
HYPERTENSION THERAPY PILOT STUDY

IRB APPLICATION

January 5th, 2015

Eric B. Loucks, PhD
Assistant Professor
Department of Epidemiology
Brown University School of Public Health
121 South Main St., Box G-S121
Providence, RI, 02912
Phone: 401-863-6283
Email: eric.loucks@brown.edu

COVER LETTER

Thank you for the helpful feedback. Below is an itemized response to the Board's recommendations.

The Board requested the following:

1. **Please ensure the study documents are consistent throughout the entire protocol submission. The Board noted that the protocol, recruitment material, consent document, surveys, and focus group documents contained conflicting information, did not match each other, and referenced sections and procedures that were not included in other documents in the submission.**

   RESPONSE: Thank you, we apologize for the inconsistencies. Documents should now be consistent throughout.

2. **Please provide further description of the recruitment and consenting processes. The Board noted that the protocol contained too few details about these parts of the study.**

   RESPONSE: Please see increased information below.

   APPLICATION CHANGE (pg. 15):

   Participants will be recruited through cardiology and family practices via established relationships with physicians at Rhode Island Hospital (e.g. Sam Dudley, MD), Memorial Hospital of Rhode Island (e.g. Charles Eaton, MD), Providence VA Medical Center (e.g. Hank Wu, MD), amongst others. IRB approval will be obtained by hospitals requiring it, such as Rhode Island Hospital and the Providence VA Medical Center. Typically, invitation letters (example text shown in Section F) will be sent to 96 eligible patients in each collaborative hospital. Patient eligibility will be based on medical records of blood pressure and gender. Participants will be recruited in equal numbers based on hypertension status (i.e. pre-hypertensive, controlled hypertensive, and uncontrolled hypertensive), with equal proportions being male and female when possible. This approach will provide important recruitment and participation rate data for future studies. Furthermore, advertisements will be posted throughout Rhode Island inviting participants with pre-hypertension or hypertension to enroll (example advertisement shown in Section F).

3. **Please clarify how many participants you intend to enroll in the study. The Board was unsure if you would enroll the first 60 participants, or if you intend to screen a much higher number of potential participants to enroll a total of 60 people. The Board also questioned how the 60 participants would be stratified across the three prehypertension/hypertension categories described in the protocol.**

   RESPONSE: This has now been clarified. Please see change below.

   APPLICATION CHANGE (pg. 15):
   For Aims 1-3, we anticipate n=60 participants with equal proportions of males and females, and stratified across prehypertension/hypertension categories, including (1) pre-hypertension, (2) controlled hypertension, and (3) uncontrolled hypertension. This will allow for early indications of potential differential effects of MBHT by gender and prehypertension/hypertension status. Likely substantially greater than 60 participants will need to be recruited for screening, of which a proportion will be eligible based on criteria below and strata requirements above. We expect approximately 120

participants required to be screened for 60 participants to be included in the study. This study will provide more accurate information on recruitment rates, which will inform future randomized controlled trials if the study shows initial evidence of effectiveness. For Aim 4, pilot data from Aim 2 will provide needed effect sizes and statistical variance for power calculations. However, current power calculations were based on SBP and DBP changes in the Mindfulness-Based Stress Reduction RCT performed by Hughes *et al.*[1] Specifically, Hughes *et al.* showed, with mean baseline SBP of 129.5 (6.3 SD) mmHg and DBP of 77.8 (5.5 SD) mmHg, at 8 weeks follow-up a net reduction of 4.2 mmHg SBP and 3.1 mmHg DBP for MBSR vs. active control group.[1] For a sample size of 35 participants per group, power analyses show 80% and 90% power to detect differences of 4.3 and 4.9 mmHg SBP, respectively. This suggests adequate power for main effects analyses. At this time, Aim 4 anticipates 70 participants in the study (35 per treatment arm), of which we expect approximately 140 participants will need to be screened.

4. **Please further clarify when, where, and how participants will be asked to complete the screening and baseline surveys.**

RESPONSE: This information has now been clarified in Part 3.7.5 of Section C, as well as changes throughout Section G.

APPLICATION CHANGES:

Pg. 17:

Interviewer training includes high standards for selection of interviewers who are at ease with strangers, are culturally-sensitive and able to readily establish professional rapport. Interviewers will be thoroughly trained in the administration of the structured interview, anthropometry, and blood pressure assessments. Routine quality control procedures will be in place regarding calibration of scales and blood pressure instruments. Clinical exams will be supervised by Dr. Loucks to at least monthly to evaluate accuracy of technique, particularly for anthropometry and blood pressure assessment. Re-training will be implemented if needed. Data will be entered and cleaned in an ongoing matter to facilitate quality control and preliminary analyses.

Pp. 18-19:

3.7.5 Timing and Locations of Assessments

Phone-Based Screening: For people who indicate interest in the study, this screening will take place by phone using trained research assistants to assess the following exclusion criteria (see Section G for questions): (a) current regular meditation practice (>once/week); (b) serious medical illness precluding regular class attendance; (c) current substance abuse, suicidal ideation or eating disorder, (d) history of bipolar or psychotic disorders or self-injurious behaviors. These participants are excluded because they may disrupt group participation, require additional or specialized treatment, or are already participating in practices similar to the intervention.

In-Person Screening: If participants remain eligible after the phone-based screening, they will attend an in-person screening for blood pressure and medication assessment. If mean blood pressure shows hypertension/prehypertension (≥120 mmHg systolic, ≥80 mmHg diastolic pressure or taking antihypertensive medication), participants will be invited to return for a second blood pressure reading. At that time, if the mean blood pressure across both assessment times is ≥120 mmHg systolic, ≥80 mmHg diastolic pressure, or the participants is taking antihypertensive medication, they will be invited to participant in the study.

Baseline Assessments Completed at Home: Questions about demographics, health behaviors, self control, depressive symptomatology, anxiety symptoms, mindfulness, sleep, childhood experiences,

amongst others will be assessed via internet-based Survey Monkey or via paper-based forms. Please see Section G for the questionnaires.

Follow-Up Assessments at 8 Weeks and 3 Months Follow-Up: Identical assessments will take place at 8 weeks and 3 months follow-up, as done with the screening and baseline assessments described above.

Focus Groups: Focus group assessments of participants will take place following the final week of class. Descriptions of focus group topics are in Section G.

5. **Please clarify whether the data collected from the screening survey will be destroyed after eligibility is determined, since participants will not go through a consent process before completing this survey.**

   RESPONSE: Data for these participants will be destroyed.

   APPLICATION CHANGES (pg. 18):

   Phone-Based Screening: For people who indicate interest in the study, this screening will take place by phone using trained research assistants to assess the following exclusion criteria (see Section G for questions): (a) current regular meditation practice (>once/week); (b) serious medical illness precluding regular class attendance; (c) current substance abuse, suicidal ideation or eating disorder, (d) history of bipolar or psychotic disorders or self-injurious behaviors. These participants are excluded because they may disrupt group participation, require additional or specialized treatment, or are already participating in practices similar to the intervention. For participants excluded from the study at this stage, data will be destroyed, as participants will not have gone through a consent process prior to completing this survey.

   In-Person Screening: If participants remain eligible after the phone-based screening, they will attend an in-person screening for blood pressure and medication assessment. If mean blood pressure shows hypertension/prehypertension (≥120 mmHg systolic, ≥80 mmHg diastolic pressure or taking antihypertensive medication), participants will be invited to return for a second blood pressure reading. At that time, if the mean blood pressure across both assessment times is ≥120 mmHg systolic, ≥80 mmHg diastolic pressure, or the participants is taking antihypertensive medication, they will be invited to participant in the study. For participants excluded from the study at this stage, data will be destroyed, as participants will not have gone through a consent process prior to completing this survey.

6. **Please ensure the "Confidentiality of Data" section of the protocol is consistent, and specify how the digital data will be protected.**

   RESPONSE: The sections should now be consistent. Please see pg. 16 for the protection of digital data.

7. **Please describe your and your undergraduate students' training and qualifications related to conducting the psychological assessments of the phone screen survey.**

   RESPONSE:

   I am a social epidemiologist studying psychosocial determinants of health. I received training at the University of Massachusetts Medical School on baseline assessment of participants prior to undergoing Mindfulness-Based Stress Reduction. Similar questions are asked and discussed with

participants at the University of Massachusetts Mindfulness-Based Stress Reduction intervention as is proposed in the study at Brown University. Furthermore, I will work closely with co-investigator and psychologist Dr. Willoughby Britton, who has screening questions identical or similar to these assessed in her Brown University Mindfulness-Based Cognitive Therapy study entitled: "Dismantling Mindfulness: Contributions of attention vs. acceptance" (#1105000399).” She will be available for questions that arise in the context of this survey. Student interviewer training includes high standards for selection of interviewers who are at ease with strangers, are culturally-sensitive and able to readily establish professional rapport. Interviewers will be thoroughly trained in the administration of the structured interview, including the screening questionnaire. A Safety Plan has been developed, which will provide structure in cases of participants indicating mental distress, particularly related to suicidal ideation.

APPLICATION CHANGE *(pp. 31-33):*

Please see Safety Plan.

8. **Please confirm that the "MBHT Phone Screen" face page will not be attached or connected to the phone script or screening data collected.**

   RESPONSE: This face page will not be attached or connected to the phone script or screening data collected.

9. **Please identify how data collected from Survey Monkey during the baseline survey will be linked to participants, and how participant e-mail addresses will not be linked with their data.**

   RESPONSE: For online questionnaires, participants will be provided with a weblink to the survey. Upon going to the weblink, they will enter the survey. First, participants will be asked to enter their study ID #. There will be no opportunities for participants to enter their names, personal addresses or email addresses into the SurveyMonkey data collection forms. Data will then be downloaded and stored within the secure VMScluster at the Brown University Center for Population Health and Clinical Epidemiology. If participants prefer, they will be provided with a paper copy of the questionnaire that can be filled out. Study staff will then manually enter the responses, and the data will be stored on the VMScluster.

10. **The Board was unsure if the computer is the most effective data collection method for the types of questions in the baseline survey (for example, the lengthy "Medications" section). Please provide a rationale for why this survey should be completed by computer.**

    RESPONSE: Thank you for pointing this out. The medications will be assessed in-person during the first screening assessment, as blood pressure medication use is one of the screening criteria. Medications will also be assessed during the in-person assessments at 9 weeks and 3 months follow-up. Please see Part 3.7.5 in Section C, as well as Section G, for more details.

11. **To the baseline survey, please add a disclaimer informing participants that their answers will not be seen immediately, and include psychological and health resources for participant use.**

    RESPONSE: Thank you, please see the changes below.

    APPLICATION CHANGES (pg. 77 of questionnaire document in Section H):

Thank you for completing this survey!

Please note that these responses will not be seen immediately. Resources are shown below if you feel that you would like to talk with someone immediately for assistance.

National Suicide Prevention Lifeline: 1-800-273-8255
National Sexual Assault Hotline: 1-800-656-4673

Other options are to:

- Call your doctor's office
- Call 911 for emergency services
- Go to the nearest hospital emergency room.

12. **Please include a more complete safety plan and describe the role of the licensed clinician on the study, as the protocol does not provide sufficient detail of what will be required of them and exactly when they will be contacted during study procedures. Please note that the clinician must be licensed in Rhode Island and have the expertise in behavioral health necessary to address any potential risks that could arise during the course of the study.**

    RESPONSE: Thank you, a safety plan has been added. We are currently in discussions with clinicians licensed in Rhode Island. Dr. Ellen Flynn, MD, is a Brown professor and RI licensed psychiatrist who has expressed interest in being the licensed clinician for this study. She is currently performing a similar role for collaborator Dr. Willoughby Britton's Brown IRB approved protocol entitled "entitled: "Dismantling Mindfulness: Contributions of attention vs. acceptance" (#1105000399)." We will be meeting in early January 2015 to discuss further. This study will not be initiated until an IRB-approved clinician has been confirmed.

    APPLICATION CHANGE *(pp. 31-33):*

    Please see Safety Plan.

13. **The Board noted that participants may give answers to particular questions in the screening and baseline surveys that, when looked at individually or in combination with other answers, could indicate potential risk. The Board requested that you review each measure used to determine psychological state and identify the specific questions and responses that may trigger a call to the licensed clinician.**

    RESPONSE: Thank you. This has now been added to the Safety Plan.

    APPLICATION CHANGE *(pp. 31-33):*

    Please see Safety Plan.

14. **To the "Advertisement Card" and "Recruitment Letter," please add the restriction that participants cannot be regular meditators.**

    RESPONSE: This has been done.

    APPLICATION CHANGE (pg. 29):

This is a 9-week program that includes mindfulness meditation, mindful movements, and education about risk factors for blood pressure. The study is testing whether combining these interventions lowers blood pressure. The program consists of nine weekly sessions that are 2.5 hours (depending on study arm), and an all-day weekend retreat. People who have a current regular meditation practice (i.e. meditate more than once per week) are not eligible.

The Board requested the following revisions to the consent process:

1. **Please add a procedure table for both the "high dose" and "low dose" groups, detailing the different procedures for each group, the amount of time per procedure, and the total time of involvement for each group.**

   RESPONSE: The high vs. low dose aspect of the protocol has been removed for scientific reasons. A stronger study design is now being employed.

2. **In the "Explanation of Procedures" section (#8), please clarify the request for participants to notify you if they change their medication.**

   RESPONSE: We have removed reference to assessing medication use in this section, as medication use will be assessed in person at 8 weeks and 3 months follow-up.

3. **In the "Explanation of Procedures" section (#9), please clarify the "second packet of questionnaires" participants will be asked to complete, what these questionnaires will ask, and where participants will be asked to complete them.**

   RESPONSE: We have changed the wording as shown below.

   APPLICATION CHANGES:

   Pp. 18-19:

   3.7.5. Timing and Locations of Assessments

   Phone-Based Screening: For people who indicate interest in the study, this screening will take place by phone using trained research assistants to assess the following exclusion criteria (see Section G for questions): (a) current regular meditation practice (>once/week); (b) serious medical illness precluding regular class attendance; (c) current substance abuse, suicidal ideation or eating disorder, (d) history of bipolar or psychotic disorders or self-injurious behaviors. These participants are excluded because they may disrupt group participation, require additional or specialized treatment, or are already participating in practices similar to the intervention. For participants excluded from the study at this stage, data will be destroyed, as participants will not have gone through a consent process prior to completing this survey.

   In-Person Screening: If participants remain eligible after the phone-based screening, they will attend an in-person screening for blood pressure and medication assessment. If mean blood pressure shows hypertension/prehypertension (≥120 mmHg systolic, ≥80 mmHg diastolic pressure or taking antihypertensive medication), participants will be invited to return for a second blood pressure reading. At that time, if the mean blood pressure across both assessment times is ≥120 mmHg systolic, ≥80 mmHg diastolic pressure, or the participants is taking antihypertensive medication, they will be invited to participant in the study. For participants excluded from the study at this stage, data will be

destroyed, as participants will not have gone through a consent process prior to completing this survey.

Baseline Assessments Completed at Home: Questions about demographics, health behaviors, self control, depressive symptomatology, anxiety symptoms, mindfulness, sleep, childhood experiences, amongst others will be assessed via internet-based Survey Monkey or via paper-based forms. Please see Section G for the questionnaires.

Follow-Up Assessments at 8 Weeks and 3 Months Follow-Up: Identical assessments will take place at 8 weeks and 3 months follow-up, as done with the screening and baseline assessments described above, with the exception that questionnaires for which the answers should not change or be informative (i.e. introductory questions, age, race/ethnicity, education, childhood experiences) are not given at follow-ups.

Focus Groups: Focus group assessments of participants will take place following the final week of class. Descriptions of focus group topics are in Section G.

4. **Please revise the "Benefits" section by removing "c," as participants will not gain knowledge about scientific methods in research.**

   RESPONSE: This has been done.

5. **In the "Refusal/Withdrawal" section, please add that participants' refusal to enroll or decision to withdraw from the study will also not affect their relationship with their physician.**

   RESPONSE: This has been completed.

   APPLICATION CHANGE (pg. 26):

   Refusal/Withdrawal
   Participation is voluntary, and you may decide to opt out or refuse any part of participation, including not answering certain questions. If you decide now to participate, you can change your mind later and quit the study. The decision to not participate or to withdraw from the study will not adversely affect current or future interactions with Brown University. The decision to not participate or to withdraw from the study will also not adversely affect your relationship with your physician.

   If you decide not to participate, or if you quit the study, we will provide you with referrals for alternative treatments, if desired.

Section A:
Lay-Person Summary of Proposed Project

The World Health Organization reported that suboptimal blood pressure (BP) is responsible for more than half of cardiovascular disease mortality world-wide. Furthermore, greater than half of those with hypertension have uncontrolled BP. *A 2009 Institute of Medicine report recommended prioritizing research to "Compare the effectiveness of mindfulness-based interventions (e.g. yoga, meditation, deep breathing training) and usual care in treating... cardiovascular risk factors."* Evidence-based mindfulness interventions, including Mindfulness-Based Stress Reduction, may have some effects on blood pressure, where a recent meta-analysis and systematic review of 4 randomized controlled trials demonstrated significant effects, but evidence of heterogeneity in effect sizes. The methodologically highest quality studies had the smallest effect sizes (range 0-5 mmHg). Mindfulness-Based Stress Reduction (MBSR) has been customized to a number of disease processes, such as Mindfulness-Based Cognitive Therapy for patients with recurrent depression, and Mindfulness-Based Relapse Prevention for patients with substance use addictions. Effect sizes have been increased by customizing mindfulness interventions to diseases of interest. The same may be true for hypertension, *however mindfulness interventions customized for prehypertensive/hypertensive patients have never been investigated. Until methodologically rigorous studies to evaluate customized interventions for hypertension are performed, we will not know if the observed preliminary effects of general mindfulness interventions on blood pressure reduction could be much more effective with a tailored approach.* Consequently, we propose to conduct a stage I behavioral therapy intervention study to evaluate whether MBSR customized to prehypertensive and hypertensive patients has the potential to provide clinically relevant reductions in BP. Consequently the specific aims are:

Stage 1a: Therapy Development/Manual Writing

1 To outline and evaluate key novel elements of mindfulness-based hypertension therapy (MBHT), customized from the evidence-based MBSR. *We hypothesize that the most important novel element will be generation of mindfulness skills specifically applied to hypertension risk factors such as diet, physical activity, obesity, alcohol consumption and antihypertensive medication adherence.* This aim will be achieved using (1) focus groups of participants undergoing the MBHT behavioral intervention, (2) discussion with experts (including cardiologists, epidemiologists, mindfulness experts, mindfulness intervention instructors) prior to, and following pilot testing of MBHT in participants, and (3) clinical judgment of the investigators performing the intervention.

2 To determine effectiveness of MBHT on primary outcomes (systolic blood pressure, retention rates, recruitment rates, and adverse effects) and secondary outcomes (hypertension risk factors such as diet, physical activity, obesity, and antihypertensive medication adherence) in hypertension subgroups, specifically participants with (1) prehypertension, (2) controlled hypertension, and (3) uncontrolled hypertension. Initial decisions about the targeted sample based on hypertension status will be made.

3 To develop an MBHT therapist manual and training program, including procedures for training, supervising, and evaluating therapists. Furthermore, acceptable therapist characteristics will be developed. The manual and training program will include themes such as specification of unique and common elements of MBHT *vs.* other interventions, description of interventions excluded from MBHT, and specification of key treatment parameters such as frequency and duration of treatment, session length, topics addressed, sequence of sessions, as well as therapist adherence and competency measures. The MBHT training will consist of a therapist manual, a formal didactic training seminar, and at least one closely supervised training session.

Stage Ib: Pilot Trial

4 To determine whether a mindfulness-based hypertension therapy (MBHT) intervention, customized from the evidence-based MBSR, has promise to be an effective behavioral therapy for participants with hypertension and/or prehypertension. We will perform a randomized controlled pilot trial for MBHT *vs.* enhanced usual care control. *We hypothesize that MBHT will have adequate recruitment rates (≥10% of prehypertensive/hypertensive participants invited from physicians' offices), fairly low*

drop out rates (<15%), and medium effect sizes (e.g. 5-10 mmHg systolic BP) for reduction in blood pressure.

These findings will provide publishable pilot data that will inform future randomized clinical trials that evaluate effects of MBHT on long-term changes in blood pressure *vs.* usual care and active control groups. *If proven effective, MBHT could be offered as a complementary program in the prehypertensive/hypertensive patient population that contributes to over half of the cardiovascular disease mortality world-wide.*

Section B:

Application for Funding

An Application for the Brown internal Richard B. Salomon Faculty Research Award has been made.

These data will provide preliminary findings for an NIH R34 application that is planned to be submitted.

Section C:
Specific Aims and Methodology

1 Significance and Innovation

The World Health Organization reported that suboptimal BP is responsible for over half of cardiovascular disease mortality world-wide.[2,3] Furthermore, of those with hypertension, more than half have uncontrolled BP.[4,5] This is despite widespread knowledge of hypertension risk factors and treatments including diet, obesity, physical activity, medication adherence and excessive alcohol consumption.[6,7] Mindfulness interventions appear to improve self-regulation, self-care, and the ability to not respond to cravings for hypertension risk factors such as high caloric palatable foods and sedentary activities (and resulting obesity).[8-12] *In fact, a 2009 Institute of Medicine report recommended prioritizing research to "Compare the effectiveness of mindfulness-based interventions (e.g. yoga, meditation, deep breathing training) and usual care in treating… cardiovascular risk factors."*[13] Evidence-based mindfulness interventions including Mindfulness-Based Stress Reduction (MBSR) may have some effects, where a recent meta-analysis and systematic review of 4 randomized controlled trials (RCTs) demonstrated significant effects, but evidence of heterogeneity in effect sizes.[14] The methodologically highest quality studies had the smallest effect sizes. MBSR has been customized to a number of disease processes, such as recurrent depression, smoking and substance use addiction with resulting increases in effect sizes compared to general mindfulness-based interventions that do not take the disease process of interest into consideration.[11,15,16] *The same may be true for hypertension, however this has not yet been studied.*

Until methodologically rigorous studies to evaluate customized mindfulness-based interventions for hypertension are performed, we will not know if the observed preliminary effects of mindfulness-based interventions on BP could have much greater effect with a tailored approach. Consequently, we propose to conduct a stage I behavioral therapy intervention study to provide the most rigorous evaluation to date on whether MBSR customized to prehypertensive and hypertensive patients provides clinically relevant reductions in BP. These findings will provide publishable pilot data that will inform future randomized clinical trials that evaluate effects of MBHT on long-term changes in blood pressure *vs.* usual care and active control groups. *If proven effective, MBHT could be offered as a complementary program in the prehypertensive/hypertensive patient population that contributes to over half of the cardiovascular disease mortality world-wide.*

2 Specific Aims

Stage 1a: Therapy Development/Manual Writing

1 To outline and evaluate key novel elements of mindfulness-based hypertension therapy (MBHT), customized from the evidence-based MBSR. *We hypothesize that the most important novel element will be generation of mindfulness skills specifically applied to hypertension risk factors such as diet, physical activity, obesity, alcohol consumption and antihypertensive medication adherence.* This aim will be achieved using (1) focus groups of participants undergoing the MBHT behavioral intervention, (2) discussion with experts (including cardiologists, epidemiologists, mindfulness experts, mindfulness intervention instructors) prior to, and following pilot testing of MBHT in participants, and (3) clinical judgment of the investigators performing the intervention.

2 To determine effectiveness of MBHT on primary outcomes (systolic blood pressure, retention rates, recruitment rates, and adverse effects) and secondary outcomes (hypertension risk factors such as diet, physical activity, obesity, and antihypertensive medication adherence) in hypertension subgroups, specifically participants with (1) prehypertension, (2) controlled hypertension, and (3) uncontrolled hypertension. Initial decisions about the targeted sample based on hypertension status will be made.

3 To develop an MBHT therapist manual and training program, including procedures for training, supervising, and evaluating therapists. Furthermore, acceptable therapist characteristics will be developed. The manual and training program will include themes such as specification of unique and common elements of MBHT *vs.* other interventions, description of interventions excluded from MBHT, and specification of key treatment parameters such as frequency and duration of treatment, session length, topics addressed, sequence of sessions, as well as therapist adherence and competency

measures. The MBHT training will consist of a therapist manual, a formal didactic training seminar, and at least one closely supervised training session.

Stage Ib: Pilot Trial

4 To determine whether a mindfulness-based hypertension therapy (MBHT) intervention, customized from the evidence-based MBSR, has promise to be an effective behavioral therapy for participants with hypertension and/or prehypertension. We will perform a randomized controlled pilot trial for MBHT *vs.* MBSR active control. *We hypothesize that MBHT will have adequate recruitment rates (≥10% of prehypertensive/hypertensive participants invited from physicians' offices), fairly low drop out rates (<15%), and medium effect sizes (e.g. 5 mmHg systolic BP) for reduction in blood pressure compared to MBSR.*

3 Approach

3.1 Study Population (Aim 2)

Participants will be recruited through cardiology and family practices via established relationships with physicians at Rhode Island Hospital (e.g. Sam Dudley, MD), Memorial Hospital of Rhode Island (e.g. Charles Eaton, MD), Providence VA Medical Center (e.g. Hank Wu, MD), amongst others. IRB approval will be obtained by hospitals requiring it, such as Rhode Island Hospital and the Providence VA Medical Center. Typically, invitation letters (example text shown in Section F) will be sent to 96 eligible patients in each collaborative hospital. Patient eligibility will be based on medical records of blood pressure and gender. Participants will be recruited in equal numbers based on hypertension status (i.e. pre-hypertensive, controlled hypertensive, and uncontrolled hypertensive), with equal proportions being male and female when possible. This approach will provide important recruitment and participation rate data for future studies. Furthermore, advertisements will be posted throughout Rhode Island inviting participants with pre-hypertension or hypertension to enroll (example advertisement shown in Section F).

For Aims 1-3, we anticipate n=60 participants with equal proportions of males and females, and stratified across prehypertension/hypertension categories, including (1) pre-hypertension, (2) controlled hypertension, and (3) uncontrolled hypertension. This will allow for early indications of potential differential effects of MBHT by gender and prehypertension/hypertension status. Likely substantially greater than 60 participants will need to be recruited for screening, of which a proportion will be eligible based on criteria below and strata requirements above. We expect approximately 120 participants required to be screened for 60 participants to be included in the study. This study will provide more accurate information on recruitment rates, which will inform future randomized controlled trials if the study shows initial evidence of effectiveness. For Aim 4, pilot data from Aim 2 will provide needed effect sizes and statistical variance for power calculations. However, current power calculations were based on SBP and DBP changes in the Mindfulness-Based Stress Reduction RCT performed by Hughes *et al.*[1] Specifically, Hughes *et al.* showed, with mean baseline SBP of 129.5 (6.3 SD) mmHg and DBP of 77.8 (5.5 SD) mmHg, at 8 weeks follow-up a net reduction of 4.2 mmHg SBP and 3.1 mmHg DBP for MBSR vs. active control group.[1] For a sample size of 35 participants per group, power analyses show 80% and 90% power to detect differences of 4.3 and 4.9 mmHg SBP, respectively. This suggests adequate power for main effects analyses. At this time, Aim 4 anticipates 70 participants in the study (35 per treatment arm), of which we expect approximately 140 participants will need to be screened.

Inclusion Criterion: Hypertension/prehypertension (≥120 mmHg systolic, ≥80 mmHg diastolic pressure or taking antihypertensive medication). All genders and racial/ethnic groups are eligible to be included.

Exclusion Criteria: Exclusion criteria follow standard guidelines and recommendations:[17] (a) current regular meditation practice (>once/week); (b) serious medical illness precluding regular class attendance; (c) current substance abuse, suicidal ideation or eating disorder, (d) history of bipolar or psychotic

disorders or self-injurious behaviors. These participants are excluded because they may disrupt group participation, require additional or specialized treatment, or are already participating in practices similar to the intervention.

3.2 Confidentiality of Data

The clinical data will be de-identified but linked. Private information such as name, date of birth, and address for recontacting will be kept in a password protected, encrypted database on a different disk that the clinical data. Only the principal investigator for the purposes of patient safety or monitoring by the NIH, data safety monitoring boards or HIPPA compliance officer approved agents will be given access to identifiable personal information.

Data which contains person-identifiers is considered highly sensitive. Such data is, by policy, only stored within the VMScluster at the Brown University Center for Population Health and Clinical Epidemiology. Access is controlled by the VMS ACL mechanism, allowing read-only access to a small group of specific users and read-write access to an even smaller group. Internet access (e.g., via a Web server) is disallowed for such storage areas. Windows security controls are superceded by VMS controls, although Windows access is permitted since some data is acquired on Windows-specific media (e.g., CD-ROM). All uses of privileged necessary to grant/revoke access to any data, as well as any other security-relevant event data, is continuously logged and analyzed in the context of both the VMScluster and the Windows Domain. Network access is tightly controlled to allow anonymous access only to a small amount of read-only data; all "risky protocols" are disallowed from Internet access. For any given study with person-identifying information, such data is stripped before the data is made accessible to staff not authorized to access such identifiers. In those cases where the data is longitudinal, a non-reversible encryption of the person ID is made in order to provide a unique person ID not traceable to the underlying person. The specific algorithm used for any given set of data is stored in the same fashion and given the same security as the data itself. To make data use simpler, hierarchical "trees" of directories are given consistent access controls tied to a unique identifier, which is then granted to staff authorized to use such data. Programming staff authorized to make individual-level data available to staff not authorized to access the person-identifying components routinely copy the "encrypted" versions from the more-secured tree to a less-secured tree. Security controls are thus relatively automatic, based on the propagation of rights based on the location of a file within the file system and the identity of the user attempting to access it.

For online questionnaires, participants will be provided with a weblink to the survey. Upon going to the weblink, they will enter the survey. First, participants will be asked to enter their study ID #. There will be no opportunities for participants to enter their names, personal addresses or email addresses into the SurveyMonkey data collection forms. Data will then be downloaded and stored within the secure VMScluster at the Brown University Center for Population Health and Clinical Epidemiology. If participants prefer, they will be provided with a paper copy of the questionnaire that can be filled out. Study staff will then manually enter the responses, and the data will be stored on the VMScluster.

3.3 MBHT Intervention Groups

MBHT Intervention: MBHT is based on the standardized MBSR intervention described elsewhere,[18-21] and will likely consist of nine 2.5-hour weekly group sessions and an 8-hour one-day session, led by Dr. Loucks. Dr. Loucks is an expert in cardiovascular physiology, cardiovascular epidemiology, and social epidemiology. He completed MBSR instructor training at UMass Medical School, and has 16 years of mindfulness meditation experience, including teaching and mentoring community members, as well as a recent publication record investigating associations of mindfulness with cardiovascular health.[12,22,23] The unique areas of MBHT are expected to be education on hypertension risk factors, hypertension health effects, and specific mindfulness modules focused on awareness of diet, physical activity, medication adherence, alcohol consumption, stress, and social support for behavior change. A preliminary manual has

been created based on the standardized MBSR manual developed at UMass Medical School, and will be further developed through the Delphi-like approach described above, and sequentially revised based on participant feedback and preliminary findings.[20,21] MBHT sessions contain instruction and practice in mindfulness meditation, and conversations about stress and coping. Students learn a range of mindfulness skills including body scan exercises, meditation and yoga. Homework consists of practicing skills for ≥45 min/day, 6 days/week. Booster sessions will be offered (likely 2.5-hour sessions every 2 months).

MBSR Active Control Group: MBSR will consist of nine 2.5-hour weekly group sessions and an 8-hour one-day session, led by Dr. Eric Loucks, who is has completed MBSR instructor training at UMass Medical School.[18,19] MBSR sessions contain instruction and practice in mindfulness meditation, and conversations about stress and coping. Students learn a range of mindfulness skills including body scan exercises, meditation and yoga. Homework consists of practicing skills for ≥45 min/day, 6 days/week. Booster sessions will be offered (likely 2.5-hour sessions every 2 months).

3.4 Measures of Compliance

Participant Adherence: Data on treatment protocol adherence will be collected weekly by research staff.

MBHT Instructor Competency and Treatment Fidelity: Treatment fidelity strategies will be performed in accordance with recommendations of the NIH Behavior Change consortium.[24]

3.5 Blinding

All study staff, including those performing health assessments and contacting participants to schedule follow-up assessments, will be blinded to treatment allocation with the exception of the MBHT/MBSR instructor (Loucks) and data manager (who performs randomization).

3.6 Equipoise

This study will be designed to disrupt clinical equipoise, defined as “no consensus within the expert clinical community about the comparative merits of the alternative [trial arms] to be tested.”[25]

3.7 Assessments

We will follow study procedures similar to those performed during previous epidemiologic studies by our group. In-person screenings will take place at the Brown University Center for Population Health and Clinical Epidemiology, described in Sections 2.7.1 and 2.7.2 at baseline, completion of the 8-week MBHT course and at 3-months post-intervention. Major assessment variables are shown in **Table 1.** Qualitative interviews (focus groups) will evaluate participants’ experiences such as usefulness of specific modules, duration of sessions, time burden, and overall intervention effectiveness. <u>Interviewer training includes high standards for selection of interviewers who are at ease with strangers, are culturally-sensitive and able to readily establish professional rapport. Interviewers will be thoroughly trained in the administration of the structured interview, anthropometry, and blood pressure assessments.</u> Routine quality control procedures will be in

Table 1. Examples of variables proposed to be assessed during RCT.

	Obtained During Study		
Variables	Baseline	8 w	3 m
Race/ethnicity	✓		
Gender	✓		
Adverse childhood experiences	✓		
Socioeconomic status	✓	✓	✓
Depressive symptomatology	✓	✓	✓
Anxiety	✓	✓	✓
Perceived stress	✓	✓	✓
Self efficacy	✓	✓	✓
Mindfulness	✓	✓	✓
Blood pressure	✓	✓	
Smoking	✓	✓	✓
Diet	✓	✓	✓
Physical activity	✓	✓	✓
Alcohol consumption	✓	✓	✓
Body mass index	✓	✓	
Sleep quantity and quality	✓	✓	✓
Social support	✓	✓	✓
Loneliness	✓	✓	✓
Medication use and adherence	✓	✓	✓

m, months; w,weeks

place regarding calibration of scales and blood pressure instruments. Clinical exams will be supervised by Dr. Loucks to at least monthly to evaluate accuracy of technique, particularly for anthropometry and blood pressure assessment. Re-training will be implemented if needed. Data will be entered and cleaned in an ongoing matter to facilitate quality control and preliminary analyses.

3.7.1 Primary Outcomes

Clinic BP Assessment: Clinic-assessed systolic and diastolic BP remains the standard of care for monitoring hypertension treatment.[4,6,7,26,27] Baseline clinic BP will be determined at two in-person screening visits ≥1 week apart, completed according to American Heart Association guidelines,[26] followed by in-person assessments at all follow-up periods. BP will be assessed using a calibrated Omron HEM907XL Intellisense automated BP monitor with established validity.[28]

3.7.2 Potential Mediators

Examples of mediators to be assessed include: (1) Body Mass Index: height and weight directly assessed using standard epidemiologic methods. *(2) Physical activity:* MET minutes per week, assessed using actigraphy, which enables the quantification of physical activity.[29-33] *(3) Diet*: diet assessed utilizing Dietary Approaches to Stop Hypertension (DASH) eating pattern score,[34] measured via diet history food frequency questionnaire.[35] *(4) Perceived stress:* measured using Perceived Stress Scale.[36] *(5) Antihypertensive Medication Use:* recorded by trained technicians from medications brought in by participants at baseline assessment.

3.7.3 Potential Effect Modifiers

We propose to evaluate 2 potentially important effect modifiers: (1) gender, (2) prehypertension/hypertension category.

3.7.4 Qualitative Assessments

Following completion of the MBHT intervention, participants will be asked to participate in a focus group discussion outlined in **Box 1**, shown below. The goal of the focus groups will be to adapt MBHT to the unique needs of this population. Adaptions may include, for example, types of specific mindfulness exercises, sequence in which they are introduced, and relative emphasis on hypertension education *vs.* mindfulness practice components.

3.7.5 Timing and Locations of Assessments

Phone-Based Screening: For people who indicate interest in the study, this screening will take place by phone using trained research assistants to assess the following exclusion criteria (see Section G for questions): (a) current regular meditation practice (>once/week); (b) serious medical illness precluding regular class attendance; (c) current substance abuse, suicidal ideation or eating disorder, (d) history of bipolar or psychotic disorders or self-injurious behaviors. These participants are excluded because they may disrupt group participation, require additional or specialized treatment, or are already participating in practices similar to the intervention. For participants excluded from the study at this stage, data will be destroyed, as participants will not have gone through a consent process prior to completing this survey.

In-Person Screening: If participants remain eligible after the phone-based screening, they will attend an in-person screening for blood pressure and medication assessment. If mean blood pressure shows hypertension/prehypertension (≥120 mmHg systolic, ≥80 mmHg diastolic pressure or taking antihypertensive medication), participants will be invited to return for a second blood pressure reading. At that time, if the mean blood pressure across both assessment times is ≥120 mmHg systolic, ≥80 mmHg diastolic pressure, or the participants is taking antihypertensive medication, they will be invited to participant in the study. For participants excluded from the study at this stage, data will be destroyed, as participants will not have gone through a consent process prior to completing this survey.

Baseline Assessments Completed at Home: Questions about demographics, health behaviors, self control, depressive symptomatology, anxiety symptoms, mindfulness, sleep, childhood experiences,

amongst others will be assessed via internet-based Survey Monkey or via paper-based forms. Please see Section G for the questionnaires.

Follow-Up Assessments at 8 Weeks and 3 Months Follow-Up: Identical assessments will take place at 8 weeks and 3 months follow-up, as done with the screening and baseline assessments described above, with the exception that questionnaires for which the answers should not change or be informative (i.e. introductory questions, age, race/ethnicity, education, childhood experiences) are not given at follow-ups.

Focus Groups: Focus group assessments of participants will take place following the final week of class. Descriptions of focus group topics are in Section G.

Box 1. Focus Group Topics

- How is your experience of the class length? Does it seem about right, or would you prefer it longer or shorter? – e.g. length of each class, 8 week duration
- Which modules did you find worked the best for you?
- Are there topics or modules you think would help improve the course?
- Are there areas we should expand?
- Are there areas we should reduce?
- Describe different modules, and ask how well they worked, specifically what they liked, didn't like, and recommendations for what to change and what to keep the same.
    - Hypertension education in the orientation session
    - Class 2: Highly palatable snack practice
    - Class 2: Home practice related to pleasant events calendar on eating and alcohol consumption
    - Class 3: Exploration of physical activity including discussion in class, and home practice of performing physical activity.
    - Class 4: 15 min physical activity module followed by discussion .
    - Class 4: Motivational interviewing module related to diet, alcohol consumption and physical activity
    - Class 4-7: Home practice related to goal for diet, alcohol consumption, physical activity or diet.
    - Class 5: Exploring relationship with medication adherence
    - Class 6: Social support module
    - All-Day: Silent exercise module
    - Booster sessions

3.8 Intervention Allocation

Stratified randomization will be used.[37,38] Variables used to create strata include gender (male *vs.* female), race/ethnicity (white *vs.* non-white), and prehypertension/ hypertension status (pre-hypertension, controlled hypertension, uncontrolled hypertension). Simple random sampling will occur within each strata.

3.9 Data Analysis

Qualitative analyses will evaluate participant experiences such as usefulness of specific modules, duration of sessions, time burden, and overall intervention effectiveness. Quantitative analyses will incorporate generalized linear models (GLM) with properly chosen link functions, performed using generalized estimating equations (GEE) with robust standard error estimators.[39,40] We will test effects of high *vs.* low dose MBHT on clinic-assessed systolic and diastolic BP. Following "intention-to-treat" principles, analyses will be conducted on all participants randomized to high *vs*. low dose, regardless of intervention completion. To test whether effects of MBHT on BP may be effect modified by variables described in Section 2.7.3 we will add product terms between MBHT and each potential effect modifier to models described above. Formal statistical significance tests of product terms will be performed. Analyses are considered statistically underpowered, but will provide effect size estimates and statistical variances for NIH grant applications to support studies with larger sample sizes.

Section D:
Possible Risks to Participants

The following is a statement from NCCAM about the potential risks of meditation practice: "Meditation is considered to be safe for healthy people. There have been rare reports that meditation could cause or worsen symptoms in people who have certain psychiatric problems, but this question has not been fully researched. People with physical limitations may not be able to participate in certain meditative practices involving physical movement. Individuals with existing mental or physical health conditions should speak with their health care providers prior to starting a meditative practice and make their meditation instructor aware of their condition."[41]

Discomfort during meditation: Meditation-based interventions may results in discomfort with attention to unpleasant thoughts, feelings or body sensations. Some individuals may experience an initial increase in undesirable emotions with increased attention to them. In order to minimize risk, instructors encourage participants to be aware of their discomfort and to set their own limitations. Participants are encouraged to alter their postures, including standing, sitting in chairs, lying down, as needed in order to be comfortable. In addition, the curriculum of the training program is specifically geared towards addressing such discomfort.

Psychological distress: Research subjects participating in this study may have feelings of loss of privacy from being contacted about participating in the study, and possible psychological distress caused by questions on the diagnostic interview that bring up painful memories or feelings. However, the resulting potential for injury to research subjects is judged to be minimal. We have already contacted and clinically evaluated thousands of participants from the New England Family using similar assessment procedures to this study, with good responses from the participants. With regard to psychological distress from taking part in the MBHT intervention, given that screening questions will exclude participants with substantial mental illness, and given the NCAAM statement above that "Meditation is considered to be safe for healthy people.",[41] we expect that risk of psychological distress will be low. The risk of increased psychological distress from meditation will be clearly outlined in the consent form and participants will be encouraged to consult with both the course instructor and study staff in the case of any increased distress. One of the co-investigators (Dr. Britton) is a clinical psychologist. She will be available to advise on any psychological events that occur, and provide referrals for treatment if needed.

Loss of confidentiality: Likelihood: rare. Minimization: Confidentiality will be maintained by numerically coding all data, by disguising identifying information, and by keeping all hard copies data in locked file drawers. All information obtained from participants will be accessible only to research staff. The clinical data will be de-identified but linked. For electronic data, private information such as name, date of birth, and address for recontacting will be kept in a password protected, encrypted database on a different disk than the clinical data. Only the principal investigator for the purposes of patient safety or monitoring by the NIH, data safety monitoring boards or HIPPA compliance officer approved agents will be given access to identifiable personal information.

Potential Benefits of the Proposed Research to Human Subjects and Others: There may be no direct benefits from participating in this study. The potential benefits of participating in the proposed study include improved knowledge on stress reduction techniques and risk for hypertension. Each participant will also be contributing to an important study which will answer questions on whether mindfulness-based stress reduction may influence health.

Section E:
Informed Consent

Each potential participant will be fully informed about the nature of the study, its risks and benefits, and about his/her rights as a research subject. The person performing the consent will be either Dr. Loucks or a trained undergraduate trained research assistant. The participant will sign and receive a copy of the document stating that he/she has given his/her informed consent to participate before interviewing is begun. In every instance, project personnel will verbally review the content of the consent form before participants sign.

Agreement to Participate in a Research Study

Investigation of the effects of mindfulness meditation on blood pressure and well-being

You are being asked to take part in a Brown University research study about the effects of mindfulness and hypertension education on blood pressure and risk factors for hypertension. This form will explain the purpose of the study, how the study will be carried out and what you will be expected to do. It will also explain the possible risks and possible benefits of being in the study. If any part of the following description is not clear to you, you are encouraged to contact the researcher to answer any questions before you decide whether to take part in the study. If you decide to participate, please fill out and sign the last page of this form.

1a. Nature and Purpose of the Study
The purpose of the study is to investigate the impact of mindfulness practices and health education on blood pressure. You have been selected for this study because you expressed interest and met entrance criteria for having prehypertension or hypertension, or another cardiovascular risk factor that could be influenced by this program. In order to assess the effects of these practices, you will be asked to complete some questionnaires, and a laboratory assessment before and after learning the mindfulness practices.

1b. Explanation of Procedures

If you agree to participate, you will be asked to consent to the following:

1) Participate in an interview in which you will be asked questions about past and present mental health, including depression and suicide.

2) Complete questionnaires about a wide range of topics, including your diet, physical activity, smoking, medication use, personality, emotions, attention and past experiences, including stressful or traumatic experiences. These questions will probe sensitive psychological areas, including physical, emotion and sexual abuse. These questionnaires may take up to 2 hours to complete. By completing the interviews and questionnaires, you are giving the researchers permission to use the information you have provided. You have the right not to answer any of the questions.

3) Directly assessed blood pressure, height, weight, waist circumference and hip circumference.

4) You will be asked to perform some cognitive tasks. One of these tasks may involve computer-based tests of attention or decision-making. Together these tests may take as long as 45 minutes.

5) You will be randomly assigned (like the flip of a coin) to enter one of two 8-week meditation programs.

6) You will participate in the 8-week meditation program, which consists of 9 sessions of 2.5 hours each and will include one 8 hour weekend retreat. Daily homework assignments may take as long as one hour and consist of practicing mindfulness exercises with the aid of a guided audio CD and completing worksheets related to stress, thoughts, and common reactions to various types of events.

7) Class sessions will be audio taped so we can analyze the quality of the treatment you receive. The recordings will be transcribed so that we may analyze the text. The recordings will be identified by study number, will only be heard by study staff and will be destroyed after transcription.

8) You will be asked to complete a few short questionnaires each week during the 8 week condition.

9) After 8 weeks, you will be asked to complete questionnaires and return to the laboratory to repeat the same procedures for a second day of testing.

10) Three months after the end of the 8 week condition, you will be asked to return to the laboratory to repeat the same testing procedures.

Feedback:
At the end of the study, you will receive individual feedback about the changes that occurred since the first assessment. Specifically, you will receive an individualized handout listing % change (increase or decrease) on scales of attention, stress, mood, health behaviors and blood pressure across the study.

2. Discomforts and Risks
The questionnaires are routine, standardized forms for epidemiologic research. Certain questions may be upsetting as they may probe sensitive psychological areas and inquire about upsetting or traumatic events, including physical, sexual or emotional abuse and/or current psychiatric symptoms. Since your participation is voluntary, you have the right to skip any questions that make you uncomfortable.

Meditation-based interventions may results in discomfort with attention to unpleasant thoughts, feelings or body sensations. Some individuals may experience an initial increase in undesirable feelings with increased attention to them.

3. Benefits
We cannot and do not guarantee or promise that you will receive any direct benefits from this study. However, participation in the study creates the potential benefit of a) identifying effective treatments for elevated blood pressure, b) gaining knowledge of the effects of mindfulness practices, c) receiving information about your psychological and physical functioning.

4. Alternative Therapies
A number of different therapies, including antihypertensive medication, diet changes, physical activity, and reducing excessive alcohol consumption may also be beneficial for reducing blood pressure. Education about these therapies are integrated into this course, but other forms of these alternative therapies are also available in the community.

5. Confidentiality
Your responses for this study will be kept confidential. All data that we collect will be linked to a study ID# instead of your name. All questionnaires in this study will be filled out through an online survey website (SurveyMonkey) or a paper version of the survey if you prefer. All of these questionnaires will be linked solely to your study ID#, so that your identity is protected and your answers are confidential. Although these measures have been taken to protect your personal information, complete confidentiality cannot be guaranteed when transmitting information over the internet.

While your confidentiality is protected to the extent of the law, there are limitations to confidentiality. If your questionnaire responses indicate that you pose a serious danger to yourself or to another person, then

a collaborator [provide name] who is a licensed psychiatrist, may contact you to discuss your responses and possible referral to a treatment provider. Questionnaire items that may warrant follow-up include endorsements of statements about hurting yourself, any high scores in depression, anxiety, or other clinically significant problems. You should also know that there are times when the law might require the release of your responses without your permission. For example, State law requires researchers to report abuse or neglect of children to the Department of Children, Youth and Families (DCYF). State law also requires researchers to report abuse or neglect of people age 60 and older to the Department of Elderly Affairs.

The findings of the study may be used for medical publication. Your name will not be used in any published reports about this study. Results will be reported in a summarized manner in such a way that you cannot be identified. All personally identifiable information will be "de-identified" and only a unique code number will be used. Study records will be identified with a unique code number and initials. All study records and specimens will be stored in a secure storage area.

Keeping study records: The Principal Investigator for this study will keep your research records indefinitely for research purposes.

6. Refusal/Withdrawal

Participation is voluntary, and you may decide to opt out or refuse any part of participation, including not answering certain questions. If you decide now to participate, you can change your mind later and quit the study. The decision to not participate or to withdraw from the study will not adversely affect current or future interactions with Brown University. The decision to not participate or to withdraw from the study will also not adversely affect your relationship with your physician.

If you decide not to participate, or if you quit the study, we will provide you with referrals for alternative treatments, if desired.

7. Contact Information

If you have questions about study procedures at any time you may contact the researchers: Dr. Eric B. Loucks, email: eric.loucks@brown.edu, telephone (401) 863-6283. If you would like more information about the rules for research studies, or the rights of people who take part in those studies, you may contact the Brown University Research Protections Office, telephone number 1-866-309-2095 or 401-863-3050.

CONSENT FORM:
Please sign and return.

Please read the following agreement and sign to consent to participating.

I HAVE READ THE ABOVE DESCRIPTION OF THIS STUDY. ALL OF MY QUESTIONS HAVE BEEN SATISFACTORILY ANSWERED, AND, I AGREE TO PARTICIPATE IN THIS RESEARCH STUDY.

____
PRINT NAME

______________________________________ ____________________
Signature of participant **Date**

CONTACT INFORMATION

Name (print):____

Permanent Address:____

Email(s):____

Telephone:________________________(cell)__________________________(other)

Section F:
Recruiting Materials

Front of Advertisement Card:

BROWN

Brown University
School of Public Health
Department of Epidemiology

RESEARCH STUDY:
Mindfulness-Based Intervention for
Blood Pressure Reduction

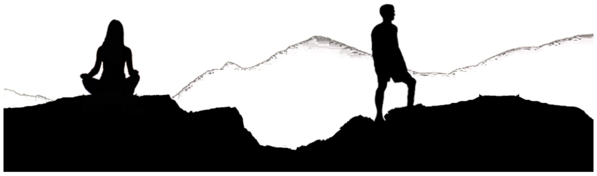

Free to qualified participants

Back of Advertisement Card:

This is a 8-week program that includes mindfulness meditation, mindful movements, and education about risk factors for blood pressure. The study is testing whether combining these interventions lowers blood pressure. The program consists of nine weekly sessions that are 2.5 hours (depending on study arm), and an all-day weekend retreat. People who have a current regular meditation practice (i.e. meditate more than once per week) are not eligible.

Research participation includes interviews, questionnaires and measurements of blood pressure, height, weight, and waist circumference, before and after the meditation program.

Contact: For more information, or to see if you qualify, call the Brown Sociobiology Laboratory at 401-863-6283, or email SBL@gmail.com. See website at [URL needs to be created].

Sample Text of Recruitment Letter (placed on Dr. Loucks' letterhead):

[Date]

Dear _____,

Your physician [name of physician] identified you as someone with prehypertension or hypertension who may be interested in participating in a new study at Brown University that is designed to evaluate effects of an intervention called Mindfulness-Based Hypertension Therapy (MBHT). MBHT combines mindfulness practices such as meditation and mindful movements with education about hypertension management and prevention. The aim of MBHT is to create a skill set and supportive environment to help to reduce blood pressure, and improve overall well-being. Your involvement in this study could help us better understand if MBHT is effective, and how it could be improved. The MBHT intervention will take place over 8 weeks, and would involve you attending a class once per week that would be between 1 and 2.5 hours, depending on which group you are assigned to. You may also be assigned to participate in a full day mindfulness retreat that would be about 7.5 hours duration. Also, health assessments would be performed before you start the intervention, and again immediately at the end of the 8-week intervention, as well as at 3 months and 6 months follow-up. The assessments would include measures such as blood pressure, height, weight, diet, physical activity, smoking, depression, stress, and medication use. These assessments will take 1-2 hours to complete.

At the end of the study, you will receive individual feedback about the changes that occurred since the first assessment. Specifically, you will receive an individualized handout listing your changes on scales of attention, stress, mood, health behaviors and blood pressure across the study.

We cannot and do not guarantee or promise that you will receive any direct benefits from this study. However, participation in the study creates the potential benefit of a) identifying effective treatments for elevated blood pressure, b) gaining knowledge of the effects of mindfulness practices, c) gaining first-hand knowledge about the most current scientific methods in scientific research d) receiving information about your psychological and physical functioning.

If you are interested in participating in this study, please call 401-863-6283, or email eric.loucks@brown.edu.

Sincerely yours,

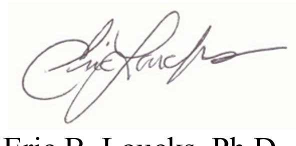

Eric B. Loucks, Ph.D.
Assistant Professor

Section G:
Safety Plan

SAFETY PLAN

If a participant's interview or questionnaire responses indicate that he/she poses a serious danger to him/herself or to another person, then the safety plan described below will be triggered into effect.

1 HOW QUESTIONNAIRES WILL BE USED TO DETERMINE RISK

1.1 Beck Anxiety Inventory (BAI)

The BAI was designed to measure clinical anxiety in a way that minimizes the overlap between depression and anxiety.[1] A BAI score of 26-63 represents severe anxiety.[1] Questionnaires will be reviewed within one week of completion. If participants score ≥26 on the BAI, study staff will notify the PI within 24 hours of seeing this information, who will in turn notify the collaborating clinical psychologist within 24 hours to determine need for a psychological consultation.

1.2 Centers for Epidemiologic Studies Depression Revised (CESD-R) Scale

The 20 items in CESD-R scale measure symptoms of depression in nine different groups as defined by the American Psychiatric Association Diagnostic and Statistical Manual, fourth edition.[2] These symptom groups are shown below, with their associated scale question numbers.

1. Sadness (dysphoria): Question numbers 2,4, 6
2. Loss of Interest (anhedonia): Question numbers 8, 10
3. Appetite: Question numbers 1, 18
4. Sleep: Question numbers 5, 11, 19
5. Thinking / concentration: Question numbers 3, 20
6. Guilt (worthlessness): Question numbers 9, 17
7. Tired (fatigue): Question numbers 7, 16
8. Movement (agitation): Question numbers 12, 13
9. Suicidal ideation: Question numbers 14, 15

Questionnaires will be reviewed within one week of completion.

Participants are considered to meet criteria for major depressive episode if they have anhedonia or dysphoria nearly every day for the past two weeks, plus symptoms in an additional 4 DSM symptom groups noted as occurring nearly every day for the past two weeks.[2] If participants meet criteria for major depressive episode, study staff will notify the PI within 24 hours of seeing this information, who will in turn notify the collaborating clinical psychologist within 24 hours to determine need for a psychological consultation.

If participants respond having any suicidal ideation (CESD-R questions 14 or 15), this will trigger the safety plan where our collaborating clinical psychologist will be notified directly by study staff to contact this participant immediately for suicidality evaluation and counseling. The PI will be informed that this has taken place.

1.3 Other Triggers for the Safety Plan

Although current suicidality is not assessed during verbal interviews, history of suicide attempts is asked verbally. If participants indicate current suicidal ideation at any time during the screening or other verbal interviews, study staff will be instructed to immediately end the interview in a sensitive way, and let participants know that a trained clinical psychologist will contact them to offer help. The staff member will provide the following information to study participants.

National Suicide Prevention Lifeline: 1-800-273-8255

Other options are to:
- Call your doctor's office
- Call 911 for emergency services
- Go to the nearest hospital emergency room.

2 References

1. Beck AT, Epstein N, Brown G, Steer RA. An inventory for measuring clinical anxiety: psychometric properties. *Journal of consulting and clinical psychology.* Dec 1988;56(6):893-897.
2. Van Dam NT, Earleywine M. Validation of the Center for Epidemiologic Studies Depression Scale--Revised (CESD-R): pragmatic depression assessment in the general population. *Psychiatry research.* Mar 30 2011;186(1):128-132.

References

1. Hughes JW, Fresco DM, Myerscough R, van Dulmen M, Carlson LE, Josephson R. Randomized controlled trial of Mindfulness-Based Stress Reduction for prehypertension. *Psychosom Med.* 2013;75:721-728.
2. Mendis S, Puska P, Norrving B. *Global atlas on cardiovascular disease prevention and control.* Geneva: World Health Organization;2011.
3. Hoyert DL, Xu J. Deaths: Preliminary Data for 2011. *National Vital Statistics Reports.* 2012;61(6):1-65.
4. Chobanian AV, Bakris GL, Black HR, et al. Seventh report of the Joint National Committee on Prevention, Detection, Evaluation, and Treatment of High Blood Pressure. *Hypertension.* Dec 2003;42(6):1206-1252.
5. United States Centers for Disease Control and Prevention. Getting blood pressure under control. *CDC Vitalsigns.* 2012;September.
6. James PA, Oparil S, Carter BL, et al. 2014 evidence-based guideline for the management of high blood pressure in adults: report from the panel members appointed to the Eighth Joint National Committee (JNC 8). *JAMA.* Feb 5 2014;311(5):507-520.
7. Weber MA, Schiffrin EL, White WB, et al. Clinical practice guidelines for the management of hypertension in the community a statement by the American Society of Hypertension and the International Society of Hypertension. *J Hypertens.* Jan 2014;32(1):3-15.
8. Epstein RM. Mindful practice. *JAMA.* Sep 1 1999;282(9):833-839.
9. Sedlmeier P, Eberth J, Schwarz M, et al. The psychological effects of meditation: a meta-analysis. *Psychol Bull.* Nov 2012;138(6):1139-1171.
10. Lutz A, Slagter HA, Dunne JD, Davidson RJ. Cognitive-emotional interactions - Attention regulation and monitoring in meditation. *Trends Cogn Sci.* Apr 2008;12(4):163-169.
11. Brewer JA, Mallik S, Babuscio TA, et al. Mindfulness training for smoking cessation: results from a randomized controlled trial. *Drug and alcohol dependence.* Dec 1 2011;119(1-2):72-80.
12. Loucks EB, Britton WB, Howe CJ, Eaton CB, Buka SL. Positive associations of dispositional mindfulness with cardiovascular health: The New England Family Study. *Int J Behav Med.* 2014:DOI 10.1007/s12529-12014-19448-12529.
13. Institute of Medicine. *Initial National Priorities for Comparative Effectiveness Research.* Washington, D.C.2009.
14. Abbott RA, Whear R, Rodgers LR, et al. Effectiveness of mindfulness-based stress reduction and mindfulness based cognitive therapy in vascular disease: A systematic review and meta-analysis of randomised controlled trials. *J Psychosom Res.* May 2014;76(5):341-351.
15. Goyal M, Singh S, Sibinga EM, et al. Meditation programs for psychological stress and well-being: A systematic review and meta-analysis. *JAMA internal medicine.* Mar 1 2014;174(3):357-368.
16. Bowen S, Witkiewitz K, Clifasefi SL, et al. Relative Efficacy of Mindfulness-Based Relapse Prevention, Standard Relapse Prevention, and Treatment as Usual for Substance Use Disorders: A Randomized Clinical Trial. *JAMA psychiatry.* Mar 19 2014.
17. Santorelli SF, Kabat-Zinn J. *MBSR Curriculum Guide and Supporting Materials. Mindfulness Based Stress Reduction Professional Training.* . Worcester, MA: University of Massachusetts Center for Mindfulness; 2003.
18. Kabat-Zinn J. An outpatient program in behavioral medicine for chronic pain patients based on the practice of mindfulness meditation: theoretical considerations and preliminary results. *Gen Hosp Psychiatry.* Apr 1982;4(1):33-47.
19. Kabat-Zinn J, Massion AO, Kristeller J, et al. Effectiveness of a meditation-based stress reduction program in the treatment of anxiety disorders. *Am J Psychiatry.* Jul 1992;149(7):936-943.

20. Santorelli S, Bonus K, McCallum C, et al. Mindfulness-Based Stress Reduction: Guidelines and Standards of Practice. *Integrative Medicine Mindfulness Program at University of Wisconsin-Madison and the Center for Mindfulness, University of Massachusetts.* 2004.
21. MacCoon DG, Imel ZE, Rosenkranz MA, et al. The validation of an active control intervention for Mindfulness Based Stress Reduction (MBSR). *Behaviour research and therapy.* Jan 2012;50(1):3-12.
22. Loucks EB. Meditation intervention reviews: selecting ideal control groups for meditation interventions. *JAMA internal medicine.* Jul 2014;174(7):1194-1195.
23. Loucks EB, Britton WB, Howe CJ, et al. Associations of dispositional mindfulness with obesity and central adiposity: The New England Family Study. Under Review.
24. Bellg AJ, Borrelli B, Resnick B, et al. Enhancing treatment fidelity in health behavior change studies: best practices and recommendations from the NIH Behavior Change Consortium. *Health Psychol.* Sep 2004;23(5):443-451.
25. Freedman B. Equipoise and the ethics of clinical research. *N Engl J Med.* Jul 16 1987;317(3):141-145.
26. Pickering TG, Hall JE, Appel LJ, et al. Recommendations for blood pressure measurement in humans and experimental animals: part 1: blood pressure measurement in humans: a statement for professionals from the Subcommittee of Professional and Public Education of the American Heart Association Council on High Blood Pressure Research. *Circulation.* Feb 8 2005;111(5):697-716.
27. Williams B, Lindholm LH, Sever P. Systolic pressure is all that matters. *Lancet.* Jun 28 2008;371(9631):2219-2221.
28. El Assaad MA, Topouchian JA, Darne BM, Asmar RG. Validation of the Omron HEM-907 device for blood pressure measurement. *Blood Press Monit.* Aug 2002;7(4):237-241.
29. Chen KY, Acra SA, Majchrzak K, et al. Predicting energy expenditure of physical activity using hip- and wrist-worn accelerometers. *Diabetes technology & therapeutics.* 2003;5(6):1023-1033.
30. Crouter SE, Clowers KG, Bassett DR, Jr. A novel method for using accelerometer data to predict energy expenditure. *J Appl Physiol (1985).* Apr 2006;100(4):1324-1331.
31. Troiano RP. Translating accelerometer counts into energy expenditure: advancing the quest. *J Appl Physiol (1985).* Apr 2006;100(4):1107-1108.
32. Esliger DW, Tremblay MS. Technical reliability assessment of three accelerometer models in a mechanical setup. *Med Sci Sports Exerc.* Dec 2006;38(12):2173-2181.
33. Heil DP. Predicting activity energy expenditure using the Actical activity monitor. *Research quarterly for exercise and sport.* Mar 2006;77(1):64-80.
34. Roger VL, Go AS, Lloyd-Jones DM, et al. Heart disease and stroke statistics--2012 update: a report from the American Heart Association. *Circulation.* Jan 3 2012;125(1):e2-e220.
35. Subar AF, Thompson FE, Kipnis V, et al. Comparative validation of the Block, Willett, and National Cancer Institute food frequency questionnaires : the Eating at America's Table Study. *American journal of epidemiology.* Dec 15 2001;154(12):1089-1099.
36. Cohen S, Kamarck T, Mermelstein R. A global measure of perceived stress. *J Health Soc Behav.* Dec 1983;24(4):385-396.
37. Kernan WN, Viscoli CM, Makuch RW, Brass LM, Horwitz RI. Stratified randomization for clinical trials. *J Clin Epidemiol.* 1999;52(1):19-26.
38. Friedman LM, Furberg CD, DeMets DL. *Fundamentals of Clinical Trials.* New York, NY: Springer; 2010.
39. Liang K-Y, Zeger SL. Longitudinal data analysis using generalized linear models. *Biometrika.* 1986;73(1):13-22.
40. Zeger SL, Liang K-Y. Longitudinal data analysis for discrete and continuous outcomes. *Biometrics.* 1986;42:121-130.
41. (NCCAM) NCfCaAM. Meditation: An Introduction. http://nccam.nih.gov/health/meditation/overview.htm - sideeffects 2013. Accessed April 26, 2013.

42. Beck AT, Epstein N, Brown G, Steer RA. An inventory for measuring clinical anxiety: psychometric properties. *Journal of consulting and clinical psychology.* Dec 1988;56(6):893-897.
43. Van Dam NT, Earleywine M. Validation of the Center for Epidemiologic Studies Depression Scale--Revised (CESD-R): pragmatic depression assessment in the general population. *Psychiatry research.* Mar 30 2011;186(1):128-132.

MINDFULNESS-BASED
HYPERTENSION THERAPY PILOT STUDY

REVISED IRB APPLICATION

February 11th, 2015

Eric B. Loucks, PhD
Assistant Professor
Department of Epidemiology
Brown University School of Public Health
121 South Main St., Box G-S121
Providence, RI, 02912
Phone: 401-863-6283
Email: eric.loucks@brown.edu

COVER LETTER

Thank you for the helpful feedback. Below is an itemized response to the Board's recommendations.

The Board requested the following:

1. **The Board noted that Dr. Ellen Flynn will act as the new clinical psychologist on the study and asked that you confer with her regarding the safety plan. The Board specifically requested that participant responses on the "Center for Epidemiologic Studies Depression Revised Scale" (CESD-R) be reviewed immediately, since the proposed plan details a potential nine-day delay from a participant's completion of the CESD-R to when they may be contacted by Dr. Flynn for evaluation.**

   RESPONSE: Thank you for this request. Dr. Flynn has reviewed the safety plan and provided comments for improvement. Those improvements are shown below in the safety plan and the initial phone-based screening questionnaire. Participants' scores on the CESD-R are now planned to be reviewed much more quickly, as described in the updated safety plan below.

   CHANGE: Please see underlined sections of Safety Plan (pp. 12-13) and Phone-Based Screening Questionnaire (pp. 16-18).

2. **To the consent document's procedure section, please add a table detailing the different procedures, the amount of time per procedure, and the total time of involvement for participants.**

   RESPONSE: The table shown below has been added to the consent document.

   CHANGE (pg. 7):

   Table Summarizing Activities and Time Commitment for this Study.

Activity	Time Commitment
First blood pressure screen	30 minutes
Second blood pressure screen	30 minutes
Baseline health assessments, such as questionnaires, height, weight and waist circumference.	2 hours
Mindfulness course.	9 sessions that are 2.5 hours each.<br>1 retreat day on a Saturday that will be 8 hours<br>Total course time: 30.5 hours
Home mindfulness practices assigned during course.	1 hour per day, 6 days per week, for 8 weeks.<br>Total time: 48 hours.
Health assessments, such as questionnaires, blood pressure, height, weight and waist circumference, that take place immediately after course completion.	2 hours
Health assessments, such as questionnaires, blood	2 hours

pressure, height, weight and waist circumference, that take place that take place 3 months after course completion.	
**TOTAL TIME COMMITMENT FOR STUDY**	**85 hours**

3. **To the consent documents' "Confidentiality" section (pg. 26, 2nd paragraph), please correct the title "Department of Elderly Affairs" to "Division of Elderly Affairs."**

   RESPONSE: This has been changed as requested, shown below.

   CHANGE (pg. 8 para. 5):

   State law also requires researchers to report abuse or neglect of people age 60 and older to the Division of Elderly Affairs.

Informed Consent

Each potential participant will be fully informed about the nature of the study, its risks and benefits, and about his/her rights as a research subject. The person performing the consent will be either Dr. Loucks or a trained undergraduate trained research assistant. The participant will sign and receive a copy of the document stating that he/she has given his/her informed consent to participate before interviewing is begun. In every instance, project personnel will verbally review the content of the consent form before participants sign.

Agreement to Participate in a Research Study

Investigation of the effects of mindfulness meditation on blood pressure and well-being

You are being asked to take part in a Brown University research study about the effects of mindfulness and hypertension education on blood pressure and risk factors for hypertension. This form will explain the purpose of the study, how the study will be carried out and what you will be expected to do. It will also explain the possible risks and possible benefits of being in the study. If any part of the following description is not clear to you, you are encouraged to contact the researcher to answer any questions before you decide whether to take part in the study. If you decide to participate, please fill out and sign the last page of this form.

1a. Nature and Purpose of the Study
The purpose of the study is to investigate the impact of mindfulness practices and health education on blood pressure. You have been selected for this study because you expressed interest and met entrance criteria for having prehypertension or hypertension, or another cardiovascular risk factor that could be influenced by this program. In order to assess the effects of these practices, you will be asked to complete some questionnaires, and a laboratory assessment before and after learning the mindfulness practices.

1b. Explanation of Procedures

If you agree to participate, you will be asked to consent to the following:

1) Participate in an interview in which you will be asked questions about past and present mental health, including depression and suicide.

2) Complete questionnaires about a wide range of topics, including your diet, physical activity, smoking, medication use, personality, emotions, attention and past experiences, including stressful or traumatic experiences. These questions will probe sensitive psychological areas, including physical, emotion and sexual abuse. These questionnaires may take up to 1.5 hours to complete. By completing the interviews and questionnaires, you are giving the researchers permission to use the information you have provided. You have the right not to answer any of the questions.

3) Directly assessed blood pressure, height, weight, waist circumference and hip circumference.

4) You will be asked to perform some cognitive tasks. One of these tasks may involve computer-based tests of attention or decision-making. Together these tests may take as long as 30 minutes.

5) You may be randomly assigned (like the flip of a coin) to enter one of two different 8-week meditation programs.

6) You will participate in an 8-week meditation program, which consists of 9 sessions of 2.5 hours each and will include one 8-hour weekend retreat. Daily homework assignments may take as long as one hour and consist of practicing mindfulness exercises with the aid of a guided audio CD and completing worksheets related to stress, thoughts, and common reactions to various types of events.

7) Class sessions will be audio taped so we can analyze the quality of the treatment you receive. The recordings may be transcribed so that we can analyze the text. The recordings will be identified by study number, will only be heard by study staff and will be destroyed after transcription.

8) You will be asked to complete a few short questionnaires each week during the 8 week condition.

9) After 8 weeks, you will be asked to complete questionnaires and return to the laboratory to repeat the same procedures for a second day of testing.

10) Three months after the end of the 8 week condition, you will be asked to return to the laboratory to repeat the same testing procedures.

Table Summarizing Activities and Time Commitment for this Study.

**Activity**	**Time Commitment**
First blood pressure screen	30 minutes
Second blood pressure screen	30 minutes
Baseline health assessments, such as questionnaires, height, weight and waist circumference.	2 hours
Mindfulness course	9 sessions that are 2.5 hours each.<br>1 retreat day on a Saturday that will be 8 hours<br>Total course time: 30.5 hours
Home mindfulness practices assigned during course.	1 hour per day, 6 days per week, for 8 weeks.<br>Total time: 48 hours.
Health assessments, such as questionnaires, blood pressure, height, weight and waist circumference, that take place immediately after course completion.	2 hours
Health assessments, such as questionnaires, blood pressure, height, weight and waist circumference, that take place that take place 3 months after course completion.	2 hours
TOTAL TIME COMMITMENT FOR STUDY	85 hours

Feedback:
At the end of the study, you will receive individual feedback about the changes that occurred since the first assessment. Specifically, you will receive an individualized handout listing % change (increase or decrease) on scales of attention, stress, mood, health behaviors, weight, and blood pressure across the study.

2. Discomforts and Risks
The questionnaires are routine, standardized forms for epidemiologic research. Certain questions may be upsetting as they may probe sensitive psychological areas and inquire about upsetting or traumatic events, including physical, sexual or emotional abuse and/or current psychiatric symptoms. Since your participation is voluntary, you have the right to skip any questions that make you uncomfortable.

Meditation-based interventions may results in discomfort with attention to unpleasant thoughts, feelings or body sensations. Some individuals may experience an initial increase in undesirable feelings with increased attention to them.

3. Benefits

We cannot and do not guarantee or promise that you will receive any direct benefits from this study. However, participation in the study creates the potential benefit of a) identifying effective treatments for elevated blood pressure, b) gaining knowledge of the effects of mindfulness practices, c) receiving information about your psychological and physical functioning.

4. Alternative Therapies

A number of different therapies, including antihypertensive medication, diet changes, physical activity, and reducing excessive alcohol consumption may also be beneficial for reducing blood pressure. Education about these therapies are integrated into this course, but other forms of these alternative therapies are also available in the community.

5. Confidentiality

Your responses for this study will be kept confidential. All data that we collect will be linked to a study ID# instead of your name. All questionnaires in this study will be filled out through an online survey website (SurveyMonkey) or a paper version of the survey if you prefer. All of these questionnaires will be linked solely to your study ID#, so that your identity is protected and your answers are confidential. Although these measures have been taken to protect your personal information, complete confidentiality cannot be guaranteed when transmitting information over the internet.

While your confidentiality is protected to the extent of the law, there are limitations to confidentiality. If your questionnaire responses indicate that you pose a serious danger to yourself or to another person, then a collaborator [provide name] who is a licensed psychiatrist, may contact you to discuss your responses and possible referral to a treatment provider. Questionnaire items that may warrant follow-up include endorsements of statements about hurting yourself, any high scores in depression, anxiety, or other clinically significant problems. You should also know that there are times when the law might require the release of your responses without your permission. For example, State law requires researchers to report abuse or neglect of children to the Department of Children, Youth and Families (DCYF). State law also requires researchers to report abuse or neglect of people age 60 and older to the Division of Elderly Affairs.

The findings of the study may be used for medical publication. Your name will not be used in any published reports about this study. Results will be reported in a summarized manner in such a way that you cannot be identified. All personally identifiable information will be "de-identified" and only a unique code number will be used. Study records will be identified with a unique code number and initials. All study records and specimens will be stored in a secure storage area.

Keeping study records: The Principal Investigator for this study will keep your research records indefinitely for research purposes.

6. Refusal/Withdrawal

Participation is voluntary, and you may decide to opt out or refuse any part of participation, including not answering certain questions. If you decide now to participate, you can change your mind later and quit the study. The decision to not participate or to withdraw from the study will not adversely affect current or future interactions with Brown University. The decision to not participate or to withdraw from the study will also not adversely affect your relationship with your physician.

If you decide not to participate, or if you quit the study, we will provide you with referrals for alternative treatments, if desired.

7. Contact Information

If you have questions about study procedures at any time you may contact the researchers: Dr. Eric B. Loucks, email: eric.loucks@brown.edu, telephone (401) 863-6283. If you would like more information about the rules for research studies, or the rights of people who take part in those studies, you may contact the Brown University Research Protections Office, telephone number 1-866-309-2095 or 401-863-3050.

CONSENT FORM:
Please sign and return.

Please read the following agreement and sign to consent to participating.

I HAVE READ THE ABOVE DESCRIPTION OF THIS STUDY. ALL OF MY QUESTIONS HAVE BEEN SATISFACTORILY ANSWERED, AND, I AGREE TO PARTICIPATE IN THIS RESEARCH STUDY.

____

PRINT NAME

______________________________________ ________________

Signature of participant **Date**

CONTACT INFORMATION

Name (print):____

Permanent Address:____

Email(s):____

Telephone:_______________________**(cell)**_________________________**(other)**

Safety Plan

SAFETY PLAN

If a participant's interview or questionnaire responses indicate that he/she poses a serious danger to him/herself or to another person, then the safety plan described below will be triggered into effect.

1 HOW QUESTIONNAIRES WILL BE USED TO DETERMINE RISK

1.1 Beck Anxiety Inventory (BAI)

The BAI was designed to measure clinical anxiety in a way that minimizes the overlap between depression and anxiety.[1] A BAI score of 26-63 represents severe anxiety.[1] Questionnaires will be reviewed within 48 hours of completion. If participants score ≥26 on the BAI, study staff will immediately notify the PI, Dr. Eric Loucks, and the collaborating psychiatrist, Dr. Ellen Flynn, to determine need for a psychiatric consultation.

1.2 Centers for Epidemiologic Studies Depression Revised (CESD-R) Scale

The 20 items in CESD-R scale measure symptoms of depression in nine different groups as defined by the American Psychiatric Association Diagnostic and Statistical Manual, fourth edition.[2] These symptom groups are shown below, with their associated scale question numbers.

1. Sadness (dysphoria): Question numbers 2,4, 6
2. Loss of Interest (anhedonia): Question numbers 8, 10
3. Appetite: Question numbers 1, 18
4. Sleep: Question numbers 5, 11, 19
5. Thinking / concentration: Question numbers 3, 20
6. Guilt (worthlessness): Question numbers 9, 17
7. Tired (fatigue): Question numbers 7, 16
8. Movement (agitation): Question numbers 12, 13
9. Suicidal ideation: Question numbers 14, 15

Questionnaires will be reviewed within 48 hours of completion.

Participants are considered to meet criteria for major depressive episode if they have anhedonia or dysphoria nearly every day for the past two weeks, plus symptoms in an additional 4 DSM symptom groups noted as occurring nearly every day for the past two weeks.[2] If participants meet criteria for major depressive episode, study staff will immediately notify the PI, Dr. Eric Loucks, and the collaborating psychiatrist, Dr. Ellen Flynn, to determine need for a psychiatric consultation.

If participants respond having any suicidal ideation (CESD-R questions 14 or 15), this will trigger the safety plan where Dr. Flynn will be immediately notified directly by study staff to contact this participant immediately for suicidality evaluation and counseling. The PI will be informed that this has taken place.

1.3 Other Triggers for the Safety Plan

During the phone-based screening, if participants respond yes to "Are you currently suicidal?", the interviewer will immediately have 911 and Dr. Ellen Flynn called by a colleague who has been informed beforehand that this is a possibility. Specifically, while keeping the participant on the phone, the text shown below in bold italics will be visually shown to the colleague.

I have a study participant on the phone who is currently suicidal. Please call 911 immediately, and tell them:

"I am calling on behalf of [my name] who is performing a research study at Brown University, and has a participant on the phone who says they are currently suicidal." Please provide the participants' contact information to the 911 operator (i.e. name, address, phone #, email address) as requested. This information is on page 1 of the screening questionnaire right here [interviewer has this information].

Finally, after calling 911, please call Dr. Ellen Flynn, who is the psychiatrist supporting this study. Her cell phone # is 401-258-9829. Please provide her with the same information as was done during the 911 call.

While keeping the participant on the phone, questions to be asked, in this order, include:
1. "Do you have a plan?"
2. If yes… "What are you planning?"
3. "Do you have the means to commit suicide?"
4. "Do you have access to a gun?"
5. "We are really concerned about you, and are calling 911."

Other questions that could be asked in order to keep them on the phone:
- "Tell me what is going on."
- "What's happening right now?"

The following information can be provided to study participants if they state they have had considered killing themselves in the past month. If they are currently suicidal, the main priority is to keep them on the phone while 911 and Dr. Flynn are being contacted.

National Suicide Prevention Lifeline: 1-800-273-8255

Other options are to:
- Call your doctor's office
- Call 911 for emergency services
- Go to the nearest hospital emergency room.

Local Non-Urgent Free or Inexpensive Mental Health Services:
Gateway Healthcare: 401-729-8701
Anchor Counseling Center: 401-475-9979
The Providence Center: 401-276-4020

SCREENING QUESTIONNAIRE
(PHONE-DELIVERED)

MBHT PHONE SCREEN

Date of Screen: ___________________ **Interviewer:** _______________________

Screening #: _________________________________ **(month.date.year.hour.minute)**

Name: ____

Address: ____

Phone number(s): ____

Email address (or mailing if no email):____

Notes from Interviewer:

____

____

____

My name is ____________. I am calling from the Brown University School of Public Health, because you expressed interest in participating in our mindfulness study. I want to do 10-15 minutes phone interview with you to determine if you are a good match for this particular study. Is now a good time to speak? [If yes, proceed. If no, ask for other times and note in Potential Subject Log].

There are a few things that I'd like to make clear before we start the interview. First of all, some of the questions will be very personal and sensitive. Are you in a private place to talk? Because this interview is of a personal nature, it is important that you understand that everything you say will be kept strictly confidential. No one outside of our project will ever be able to see your answers, and we will not keep your name in the same place as any of your answers. If you are not eligible after the phone screen, we will destroy your information. If you like, though, we can keep your information on file for future studies.

INCLUSION CRITERIA: All answers in 3rd column must be YES			
			Comments
AGE	Age_______ Between 18 and 65?	YES NO	
ENG	Can you read and write in English?	YES NO	

EXCLUSION CRITERIA: All answers in 3rd column must be NO			
			Comments
Bipolar depression MANIA	Has anyone ever told you that you have bipolar disorder or manic depression?	YES NO	
BPD	Has anyone ever used the word "Borderline" to describe you?	YES NO	
Psychosis	Have you ever had a hallucination or seen things that other people can't see, or hear things other people can't hear?<br><br>Have you ever been diagnosed with schizophrenia or psychosis?	YES NO<br><br>YES NO	
MANIA/ Antipsychotic MEDS	Have you ever taken any of the following medications?<br>Lithium<br>Seroquel (quetiapine)<br>Abilify (aripiprazole)<br>Zyprexa (olanzapine)<br>Clozaril (clozapine)<br>Haldol/Haloperidol<br>Geodon (ziprasidone)<br>Risperdal (risperidone)	 <br>YES NO<br>YES NO<br>YES NO<br>YES NO<br>YES NO<br>YES NO<br>YES NO<br>YES NO	
Self-harm	Have you ever had a suicide attempt?<br><br>[If yes, ask…] Have you considered killing yourself during the past month?<br><br>[If yes, ask…] Are you currently suicidal?<br><br>[If yes, keep participant on the phone, and follow suicide safety plan below]<br><br>[If no, ask…] Are you getting any help for that? If not, then provide list of resources from Safety Plan including Gateway, Anchor and The Providence Center] Not urgent, but inform Dr. Flynn about what was discussed.	 <br>YES NO<br><br>YES NO<br><br><br><br>YES NO	

EXCLUSION: CURRENT (check timeframe)			
PTSD	Would you say you have a trauma history?<br>[If yes…]<br>In the past month, have you had any problems with dissociation (memory loss)?<br>In the past month, have you had any flashbacks (i.e. sudden and disturbing vivid memory) about the trauma?	YES NO<br><br>YES NO	
OCD	In the past month, have you had any problems with obsessions or compulsions, such as washing your hands or checking the oven over and over again?<br>[If yes…] Has anyone diagnosed you with obsessive compulsive disorder?	YES NO<br><br>YES NO	
PANIC	In the past month, have you had a panic attack (i.e. sweating, heart palpitations, nausea, trouble breathing, fear of dying/choking/going crazy)?	YES NO	
ALCOHOL	Have you had any problems with alcoholism or drug use in the past year?	YES NO	
EATING DIS	In the past year, have you had an eating disorder, such as starving, binge eating, or vomiting?	YES NO	
MEDITATION	Do you currently practice meditation more than once per week? (yoga does not count as meditation in this context)	YES NO	
MED ILL	This class will take place at Brown University in-person. Do you have any medical or mobility issues that would affect you being able to attend class?	YES NO	
DECISION	PASS NO PASS QUERY		

PASS FORM: Initial appropriate boxes with dates		Date/initials
NO PASS	Thank you for taking the time to answer these questions. According to this test, it doesn't look like you are going to be a good fit for the study. There may be other studies you quality for. Would you like me to keep your information to pass on to these studies? Otherwise, our copy of this information can be destroyed.	
QUERY	Thank you for taking the time to answer these questions. There are a few items I need to check with my supervisor about. I will contact him right now, and we will get back in touch with you shortly about your participation in this study.	
PASS:<br>Study description and requirements<br>Time commitment	Thank you for taking the time to answer these questions. You qualify for the next stage of screening, which is to take your blood pressure at two different times at least a week apart from each other. Before we go on to book this, I would like to tell you a little more about the study. If you want to continue, the next phase would require an interview, a packet of questionnaires and visit to the laboratory before the 8-week meditation program. After the program, you would fill out the same questionnaires and do the lab visit again twice – once right after the course, and again 3 months later. This study involves an 8-week program that will provide training in mindfulness meditation, yoga and health behaviours related to blood pressure control. The mindfulness program takes place in a group setting meeting for 2 ½ hours once a week, 9 times over an 8 week period. For example if it were on a Monday, we would meet on 9 Mondays in a row. The program requires up to an hour of mindfulness practice every day. There will also be an all-day retreat on a Saturday part way through the course, that will be about 8 hours long. Is this something that you would be able to make time for in your schedule?	YES NO
SCHEDULE<br>Available for current program?	It's important to attend all 9 sessions.<br>The next program is:<br>Availability:<br>Mon 9:00-11:30, 4:00-6:30 pm<br>Tues 9:00-11:30, 4:00-6:30 pm<br>Wed 9:00-11:30, 4:00-6:30 pm<br>Thurs 9:00-11:30, 4:00-6:30 pm<br><br>Other:	YES NO

References

1. Beck AT, Epstein N, Brown G, Steer RA. An inventory for measuring clinical anxiety: psychometric properties. *Journal of consulting and clinical psychology.* Dec 1988;56(6):893-897.
2. Van Dam NT, Earleywine M. Validation of the Center for Epidemiologic Studies Depression Scale--Revised (CESD-R): pragmatic depression assessment in the general population. *Psychiatry research.* Mar 30 2011;186(1):128-132.

MINDFULNESS-BASED
HYPERTENSION THERAPY PILOT STUDY

REVISED IRB APPLICATION

February 15th, 2015

Eric B. Loucks, PhD
Assistant Professor
Department of Epidemiology
Brown University School of Public Health
121 South Main St., Box G-S121
Providence, RI, 02912
Phone: 401-863-6283
Email: eric.loucks@brown.edu

COVER LETTER

Thank you for the helpful feedback. Below is an itemized response to the Board's recommendations.

The Board requested the following:

1. **The plan says that Dr. Flynn will be notified immediately if participants meet the criteria for a major depressive episode or suicidality, but right above that, Section 1.2 says that the questionnaires will be reviewed within 48 hours of completion. Will these questionnaire not be immediately reviewed? Since these measures will not be completed in-person, if they may not be reviewed for 48 hours, Dr. Flynn may not interact with a participant in trouble for 2 days. The IRB feels that this is too long a delay to review these measures.**

   RESPONSE: The anxiety and depressive symptomatology questionnaires have now been moved to be completed in-person instead of at home. Responses will be reviewed immediately upon completion of the in-person assessments. Values of concern will be reported immediately to Dr. Flynn.

   CHANGE: Please see underlined changes to the safety plan on pg. 11.

2. **The IRB also feels that the RA should not ask the types of clinical questions, as described in Section 1.3's script for talking to suicidal participants. The exact language the RA will use when speaking with participants does not need to be reviewed by the IRB, but once a participant expresses suicidal ideation, (step 1) Dr. Flynn should be contacted and (step 2) the RA should speak calmly with the participants about what the RA is doing (calling Dr. Flynn) and why. The RA can speak with participants to keep them on the phone, but the discussion should not be clinical in nature.**

   RESPONSE: The clinical questions have been removed. Recommended steps 1 and 2 have been implemented.

   CHANGE: Please see underlined changes to the Safety Plan on pg. 12.

3. **The revised consent document's new activities/time table (pg. 7) says the total time commitment is 85 hours, but when I add up the time, it comes to 85.5 hours. Would you correct the consent document?**

   RESPONSE: This has been changed to 85.5 hours as suggested.

   CHANGE: Please see underlined change to the consent document on pg. 6.

Informed Consent

Each potential participant will be fully informed about the nature of the study, its risks and benefits, and about his/her rights as a research subject. The person performing the consent will be either Dr. Loucks or a trained undergraduate trained research assistant. The participant will sign and receive a copy of the document stating that he/she has given his/her informed consent to participate before interviewing is begun. In every instance, project personnel will verbally review the content of the consent form before participants sign.

Agreement to Participate in a Research Study

Investigation of the effects of mindfulness meditation on blood pressure and well-being

You are being asked to take part in a Brown University research study about the effects of mindfulness and hypertension education on blood pressure and risk factors for hypertension. This form will explain the purpose of the study, how the study will be carried out and what you will be expected to do. It will also explain the possible risks and possible benefits of being in the study. If any part of the following description is not clear to you, you are encouraged to contact the researcher to answer any questions before you decide whether to take part in the study. If you decide to participate, please fill out and sign the last page of this form.

1a. Nature and Purpose of the Study

The purpose of the study is to investigate the impact of mindfulness practices and health education on blood pressure. You have been selected for this study because you expressed interest and met entrance criteria for having prehypertension or hypertension, or another cardiovascular risk factor that could be influenced by this program. In order to assess the effects of these practices, you will be asked to complete some questionnaires, and a laboratory assessment before and after learning the mindfulness practices.

1b. Explanation of Procedures

If you agree to participate, you will be asked to consent to the following:

1) Participate in an interview in which you will be asked questions about past and present mental health, including depression and suicide.

2) Complete questionnaires about a wide range of topics, including your diet, physical activity, smoking, medication use, personality, emotions, attention and past experiences, including stressful or traumatic experiences. These questions will probe sensitive psychological areas, including physical, emotion and sexual abuse. These questionnaires may take up to 1.5 hours to complete. By completing the interviews and questionnaires, you are giving the researchers permission to use the information you have provided. You have the right not to answer any of the questions.

3) Directly assessed blood pressure, height, weight, waist circumference and hip circumference.

4) You will be asked to perform some cognitive tasks. One of these tasks may involve computer-based tests of attention or decision-making. Together these tests may take as long as 30 minutes.

5) You may be randomly assigned (like the flip of a coin) to enter one of two different 8-week meditation programs.

6) You will participate in an 8-week meditation program, which consists of 9 sessions of 2.5 hours each and will include one 8-hour weekend retreat. Daily homework assignments may take as long as one hour and consist of practicing mindfulness exercises with the aid of a guided audio CD and completing worksheets related to stress, thoughts, and common reactions to various types of events.

7) Class sessions will be audio taped so we can analyze the quality of the treatment you receive. The recordings may be transcribed so that we can analyze the text. The recordings will be identified by study number, will only be heard by study staff and will be destroyed after transcription.

8) You will be asked to complete a few short questionnaires each week during the 8 week condition.

9) After 8 weeks, you will be asked to complete questionnaires and return to the laboratory to repeat the same procedures for a second day of testing.

10) Three months after the end of the 8 week condition, you will be asked to return to the laboratory to repeat the same testing procedures.

Table Summarizing Activities and Time Commitment for this Study.

**Activity**	**Time Commitment**
First blood pressure screen	30 minutes
Second blood pressure screen	30 minutes
Baseline health assessments, such as questionnaires, height, weight and waist circumference.	2 hours
Mindfulness course	9 sessions that are 2.5 hours each.<br>1 retreat day on a Saturday that will be 8 hours<br>Total course time: 30.5 hours
Home mindfulness practices assigned during course.	1 hour per day, 6 days per week, for 8 weeks.<br>Total time: 48 hours.
Health assessments, such as questionnaires, blood pressure, height, weight and waist circumference, that take place immediately after course completion.	2 hours
Health assessments, such as questionnaires, blood pressure, height, weight and waist circumference, that take place that take place 3 months after course completion.	2 hours
TOTAL TIME COMMITMENT FOR STUDY	85.5 hours

Feedback:
At the end of the study, you will receive individual feedback about the changes that occurred since the first assessment. Specifically, you will receive an individualized handout listing % change (increase or decrease) on scales of attention, stress, mood, health behaviors, weight, and blood pressure across the study.

2. Discomforts and Risks
The questionnaires are routine, standardized forms for epidemiologic research. Certain questions may be upsetting as they may probe sensitive psychological areas and inquire about upsetting or traumatic events, including physical, sexual or emotional abuse and/or current psychiatric symptoms. Since your participation is voluntary, you have the right to skip any questions that make you uncomfortable.

Meditation-based interventions may results in discomfort with attention to unpleasant thoughts, feelings or body sensations. Some individuals may experience an initial increase in undesirable feelings with increased attention to them.

3. Benefits

We cannot and do not guarantee or promise that you will receive any direct benefits from this study. However, participation in the study creates the potential benefit of a) identifying effective treatments for elevated blood pressure, b) gaining knowledge of the effects of mindfulness practices, c) receiving information about your psychological and physical functioning.

4. Alternative Therapies

A number of different therapies, including antihypertensive medication, diet changes, physical activity, and reducing excessive alcohol consumption may also be beneficial for reducing blood pressure. Education about these therapies are integrated into this course, but other forms of these alternative therapies are also available in the community.

5. Confidentiality

Your responses for this study will be kept confidential. All data that we collect will be linked to a study ID# instead of your name. All questionnaires in this study will be filled out through an online survey website (SurveyMonkey) or a paper version of the survey if you prefer. All of these questionnaires will be linked solely to your study ID#, so that your identity is protected and your answers are confidential. Although these measures have been taken to protect your personal information, complete confidentiality cannot be guaranteed when transmitting information over the internet.

While your confidentiality is protected to the extent of the law, there are limitations to confidentiality. If your questionnaire responses indicate that you pose a serious danger to yourself or to another person, then a collaborator [provide name] who is a licensed psychiatrist, may contact you to discuss your responses and possible referral to a treatment provider. Questionnaire items that may warrant follow-up include endorsements of statements about hurting yourself, any high scores in depression, anxiety, or other clinically significant problems. You should also know that there are times when the law might require the release of your responses without your permission. For example, State law requires researchers to report abuse or neglect of children to the Department of Children, Youth and Families (DCYF). State law also requires researchers to report abuse or neglect of people age 60 and older to the Division of Elderly Affairs.

The findings of the study may be used for medical publication. Your name will not be used in any published reports about this study. Results will be reported in a summarized manner in such a way that you cannot be identified. All personally identifiable information will be "de-identified" and only a unique code number will be used. Study records will be identified with a unique code number and initials. All study records and specimens will be stored in a secure storage area.

Keeping study records: The Principal Investigator for this study will keep your research records indefinitely for research purposes.

6. Refusal/Withdrawal

Participation is voluntary, and you may decide to opt out or refuse any part of participation, including not answering certain questions. If you decide now to participate, you can change your mind later and quit the study. The decision to not participate or to withdraw from the study will not adversely affect current or future interactions with Brown University. The decision to not participate or to withdraw from the study will also not adversely affect your relationship with your physician.

If you decide not to participate, or if you quit the study, we will provide you with referrals for alternative treatments, if desired.

7. Contact Information

If you have questions about study procedures at any time you may contact the researchers: Dr. Eric B. Loucks, email: eric.loucks@brown.edu, telephone (401) 863-6283. If you would like more information about the rules for research studies, or the rights of people who take part in those studies, you may contact the Brown University Research Protections Office, telephone number 1-866-309-2095 or 401-863-3050.

CONSENT FORM:
Please sign and return.

Please read the following agreement and sign to consent to participating.

I HAVE READ THE ABOVE DESCRIPTION OF THIS STUDY. ALL OF MY QUESTIONS HAVE BEEN SATISFACTORILY ANSWERED, AND, I AGREE TO PARTICIPATE IN THIS RESEARCH STUDY.

____
PRINT NAME

______________________________________ ____________________
Signature of participant **Date**

CONTACT INFORMATION

Name (print):____

Permanent Address:____

Email(s):____

Telephone:_________________________**(cell)**_____________________________**(other)**

Safety Plan

SAFETY PLAN

If a participant's interview or questionnaire responses indicate that he/she poses a serious danger to him/herself or to another person, then the safety plan described below will be triggered into effect.

1 HOW QUESTIONNAIRES WILL BE USED TO DETERMINE RISK

1.1 Beck Anxiety Inventory (BAI)

The BAI was designed to measure clinical anxiety in a way that minimizes the overlap between depression and anxiety.[1] A BAI score of 26-63 represents severe anxiety.[1] The BAI will be administered during the in-person assessment visits, and scores will be reviewed immediately upon completion of the in-person assessments. If participants score ≥26 on the BAI, study staff will immediately notify the PI, Dr. Eric Loucks, and the collaborating psychiatrist, Dr. Ellen Flynn, to determine need for a psychiatric consultation.

1.2 Centers for Epidemiologic Studies Depression Revised (CESD-R) Scale

The 20 items in CESD-R scale measure symptoms of depression in nine different groups as defined by the American Psychiatric Association Diagnostic and Statistical Manual, fourth edition.[2] These symptom groups are shown below, with their associated scale question numbers. The CESD-R will be administered during the in-person assessment visits, and scores will be reviewed immediately upon completion of the in-person assessments.

1. Sadness (dysphoria): Question numbers 2,4, 6
2. Loss of Interest (anhedonia): Question numbers 8, 10
3. Appetite: Question numbers 1, 18
4. Sleep: Question numbers 5, 11, 19
5. Thinking / concentration: Question numbers 3, 20
6. Guilt (worthlessness): Question numbers 9, 17
7. Tired (fatigue): Question numbers 7, 16
8. Movement (agitation): Question numbers 12, 13
9. Suicidal ideation: Question numbers 14, 15

Participants are considered to meet criteria for major depressive episode if they have anhedonia or dysphoria nearly every day for the past two weeks, plus symptoms in an additional 4 DSM symptom groups noted as occurring nearly every day for the past two weeks.[2] If participants meet criteria for major depressive episode, study staff will immediately notify the PI, Dr. Eric Loucks, and the collaborating psychiatrist, Dr. Ellen Flynn, to determine need for a psychiatric consultation.

If participants respond having any suicidal ideation (CESD-R questions 14 or 15), this will trigger the safety plan where Dr. Flynn will be immediately notified directly by study staff to contact this participant immediately for suicidality evaluation and counselling. The PI will be informed that this has taken place.

1.3 Other Triggers for the Safety Plan

During the phone-based screening, if participants respond yes to "Are you currently suicidal?", the interviewer should perform the following 2 steps:

1. Immediately have 911 and Dr. Ellen Flynn called by a colleague who has been informed beforehand that this is a possibility.

Specifically, while keeping the participant on the phone, the text shown below in bold italics will be visually shown to the colleague.

I have a study participant on the phone who is currently suicidal. Please call 911 immediately, and tell them:

"I am calling on behalf of [my name] who is performing a research study at Brown University. She has a participant on the phone who says they are currently suicidal." Please provide the participants' contact information to the 911 operator (i.e. name, address, phone #, email address) as requested. This information is on page 1 of the screening questionnaire right here [interviewer has this information].

Finally, after calling 911, please call Dr. Ellen Flynn, who is the psychiatrist supporting this study. Her cell phone # is 401-258-9829. Please provide her with the same information as was done during the 911 call.

2. While speaking calmly with the participant, let them know what you are doing. Specifically, let them know we are calling our study's psychiatrist Dr. Flynn and 911, and why you are doing that (i.e. because we are concerned about you). You can speak with participant to keep him/her on the phone, but the discussion should not be clinical in nature.

Examples of questions that could be asked in order to keep them on the phone:

- "Tell me what is going on."
- "What's happening right now?"
- Tell me more about why you are interesting in being part of this study.
- What are you hoping to get out of this study?

The following information can be provided to study participants if they state they have had considered killing themselves in the past month. If they are currently suicidal, the main priority is to keep them on the phone while 911 and Dr. Flynn are being contacted.

National Suicide Prevention Lifeline: 1-800-273-8255

Other options are to:

- Call your doctor's office
- Call 911 for emergency services
- Go to the nearest hospital emergency room.

Local Non-Urgent Free or Inexpensive Mental Health Services:
Gateway Healthcare: 401-729-8701
Anchor Counseling Center: 401-475-9979
The Providence Center: 401-276-4020

References

1. Beck AT, Epstein N, Brown G, Steer RA. An inventory for measuring clinical anxiety: psychometric properties. *Journal of consulting and clinical psychology.* Dec 1988;56(6):893-897.
2. Van Dam NT, Earleywine M. Validation of the Center for Epidemiologic Studies Depression Scale--Revised (CESD-R): pragmatic depression assessment in the general population. *Psychiatry research.* Mar 30 2011;186(1):128-132.

Brown University
Research Protections Office
Institutional Review Board
Amendment Request

Date of Request: 6/17/15 **Investigator's Name and Title:** Eric Loucks, Assistant Professor

Study Title: Mindfulness-Based Hypertension Therapy Pilot Study (#1412001171)

Original Type of Review: ☐ Exempt ☐ Expedited ☒ Full Board

1) **Provide a brief lay summary of the overall project. Include enough detail to allow the IRB to evaluate the requested change(s) within the context of the overall project.** (Attach summary to this form) Please see attached.

2) **Provide a detailed description of the changes being requested** (Use additional pages, if necessary):
Please see attached.

3.) **State the reason (justification) for the requested amendment.** (Use additional pages, if necessary):
Please see attached.

4.) **What is your assessment of how the changes will affect the overall risk/benefit ratio of the study and the willingness of individuals to participate?**
Please see attached.

5.) **Does the requested amendment require new documents or changes to the approved consent form or other documents?**

☒ Consent/assent documents (attach revised version with changes highlighted)

☒ New/revised instruments (attach - if revised, highlight changes)

☐ New/revised advertising materials (attach - if revised, highlight changes)

Do you have a conflict of interest on this project according to Brown's policy? ☐**YES** ☒**NO**

If YES, has this conflict been previously disclosed to the IRB? ☐**YES** ☐**NO**

PI signature: ________________________ Date: 6/17/15

1) Provide a brief lay summary of the overall project. Include enough detail to allow the IRB to evaluate the requested change(s) within the context of the overall project.

The World Health Organization reported that suboptimal blood pressure (BP) is responsible for more than half of cardiovascular disease mortality world-wide. Furthermore, greater than half of those with hypertension have uncontrolled BP. *A 2009 Institute of Medicine report recommended prioritizing research to "Compare the effectiveness of mindfulness-based interventions (e.g. yoga, meditation, deep breathing training) and usual care in treating… cardiovascular risk factors."* Evidence-based mindfulness interventions, including Mindfulness-Based Stress Reduction, may have some effects on blood pressure, where a recent meta-analysis and systematic review of 4 randomized controlled trials demonstrated significant effects, but evidence of heterogeneity in effect sizes. The methodologically highest quality studies had the smallest effect sizes (range 0-5 mmHg). Mindfulness-Based Stress Reduction (MBSR) has been customized to a number of disease processes, such as Mindfulness-Based Cognitive Therapy for patients with recurrent depression, and Mindfulness-Based Relapse Prevention for patients with substance use addictions. Effect sizes have been increased by customizing mindfulness interventions to diseases of interest. The same may be true for hypertension, *however mindfulness interventions customized for prehypertensive/hypertensive patients have never been investigated. Until methodologically rigorous studies to evaluate customized interventions for hypertension are performed, we will not know if the observed preliminary effects of general mindfulness interventions on blood pressure reduction could be much more effective with a tailored approach.* Consequently, we propose to conduct a stage I behavioral therapy intervention study to evaluate whether MBSR customized to prehypertensive and hypertensive patients has the potential to provide clinically relevant reductions in BP. Consequently the specific aims are:

Stage 1a: Therapy Development/Manual Writing

1 To outline and evaluate key novel elements of mindfulness-based hypertension therapy (MBHT), customized from the evidence-based MBSR. *We hypothesize that the most important novel element will be generation of mindfulness skills specifically applied to hypertension risk factors such as diet, physical activity, obesity, alcohol consumption and antihypertensive medication adherence.* This aim will be achieved using (1) focus groups of participants undergoing the MBHT behavioral intervention, (2) discussion with experts (including cardiologists, epidemiologists, mindfulness experts, mindfulness intervention instructors) prior to, and following pilot testing of MBHT in participants, and (3) clinical judgment of the investigators performing the intervention.

2 To determine effectiveness of MBHT on primary outcomes (systolic blood pressure, retention rates, recruitment rates, and adverse effects) and secondary outcomes (hypertension risk factors such as diet, physical activity, obesity, and antihypertensive medication adherence) in hypertension subgroups, specifically participants with (1) prehypertension, (2) controlled hypertension, and (3) uncontrolled hypertension. Initial decisions about the targeted sample based on hypertension status will be made.

3 To develop an MBHT therapist manual and training program, including procedures for training, supervising, and evaluating therapists. Furthermore, acceptable therapist characteristics will be developed. The manual and training program will include themes such as specification of unique and common elements of MBHT *vs.* other interventions, description of interventions excluded from MBHT, and specification of key treatment parameters such as frequency and duration of treatment, session length, topics addressed,

sequence of sessions, as well as therapist adherence and competency measures. The MBHT training will consist of a therapist manual, a formal didactic training seminar, and at least one closely supervised training session.

Stage Ib: Pilot Trial

4 To determine whether a mindfulness-based hypertension therapy (MBHT) intervention, customized from the evidence-based MBSR, has promise to be an effective behavioral therapy for participants with hypertension and/or prehypertension. We will perform a randomized controlled pilot trial for MBHT *vs.* enhanced usual care control. *We hypothesize that MBHT will have adequate recruitment rates (≥10% of prehypertensive/hypertensive participants invited from physicians' offices), fairly low drop out rates (<15%), and medium effect sizes (e.g. 5-10 mmHg systolic BP) for reduction in blood pressure.*

These findings will provide publishable pilot data that will inform future randomized clinical trials that evaluate effects of MBHT on long-term changes in blood pressure *vs.* usual care and active control groups. *If proven effective, MBHT could be offered as a complementary program in the prehypertensive/hypertensive patient population that contributes to over half of the cardiovascular disease mortality world-wide.*

2) Provide a detailed description of the changes being requested (Use additional pages, if necessary):

1. Survey software:
    a. We changed the survey software from SurveyMonkey to Qualtrics. We will ensure that all software options that allow for personally identifiable information to be automatically obtained from Qualtrics (e.g. IP addresses) have been turned off. This is done through the "Anonymize Response" Survey Option that ensures IP addresses and panel information are removed from each survey.

2. Phone screening questionnaire:
    a. We updated the script for Research Assistants to use, to improve clarity. The changes to the script are shown in yellow highlighted text on pp. 3, 4, 5, 7, 8 of the Phone Screening Questionnaire in Appendix 1.

3. First in-person screening assessment:
    a. We clarified the Safety Plan for the Research Assistants, shown in highlighted yellow on pg. 19 in Appendix 2. The protocol has not been changed; only the language so that the exact steps have been clarified to the Research Assistants.

4. Consent form:
    a. Reference to the SurveyMonkey software was removed
    b. Reference to the control group was removed, as we are not using a control during the initial pilot testing of the novel intervention.
    c. The time commitment for the questionnaires has been updated to more accurately reflect the expected time commitment.
    d. The text now states that the class sessions "will be" audio recorded instead of "may be" audio recorded, as this better reflects the needs of the study to evaluate intervention fidelity.

5. Baseline Survey:
    a. Three questionnaires have been added to the baseline survey, shown below. The specific questions are shown in yellow highlighted text in the attached Baseline Questionnaire on pp. 56-59 in Appendix 4.
        i. Multidimensional Assessment of Interoceptive Awareness (IA).[1]
        ii. Experiences Questionnaire (measure of reperceiving/decentering) (EQ).[2]
        iii. Cognitive Emotion Regulation Scale (CR).[3]

6. Protocol for Physicians to Refer Patients to Mindfulness-Based Hypertension Therapy Study
    a. We were contacted by a physician named Cathleen Hood who would like to refer some of her patients to the study. A protocol that we will provide to her and other interested physicians for patient referral is attached (Appendix 5).

References:

1. Mehling WE, Price C, Daubenmier JJ, Acree M, Bartmess E, Stewart A. The Multidimensional Assessment of Interoceptive Awareness (MAIA). *PLoS One* 2012; **7**(11)**:** e48230.

2. Fresco DM, Moore MT, van Dulmen MH, Segal ZV, Ma SH, Teasdale JD *et al.* Initial psychometric properties of the experiences questionnaire: validation of a self-report measure of decentering. *Behavior therapy* 2007; **38**(3)**:** 234-46.

3. Garnefski N, Kraaij V, Spinhoven P. Negative life events, cognitive emotion regulation and emotional problems. *Personality Indiv Differences* 2001; **30:** 1311-1327.

3.) State the reason (justification) for the requested amendment:

Amendment 1 above was done due to a respected colleague (Prof. Melissa Clark) recommending Qualtrics as a higher quality survey software compared to SurveyMonkey. Amendments 2-4 above were done to improve clarity. Amendment 5 above was done to improve identification of the mechanisms by which mindfulness intervention may improve blood pressure.

4.) What is your assessment of how the changes will affect the overall risk/benefit ratio of the study and the willingness of individuals to participate?

I do not expect any changes to the risk/benefit ratio or willingness of individuals to participate.

5.) Does the requested amendment require new documents or changes to the approved consent form or other documents?

Yes. Please see attached appendices 1-3. Changes are shown in yellow highlight or track changes.

APPENDIX 1

PHONE SCREENING QUESTIONNAIRE

PHONE-DELIVERED SCREENING QUESTIONNAIRE

PHONE SCREENING QUESTIONNAIRE PART 1

*The script is shown below in **bold italics.***

PID. Participant ID: ___________

SQ01. Staff ID: ______________

SQ02. Today's Date (MMDDYY): ________________

SQ03. Current Time (24 hour time, e.g. 14:45): ________

Please now call the participant.

SQ04. Was the participant reached? YES NO

Hello, my name is ____________. I am calling from the Brown University School of Public Health because (name of participant) expressed interest in participating in our mindfulness blood pressure study. Is he/she available to talk at this time?

I would like to do a 10-15 minute phone interview with you to determine if you are a good match for this particular study.

*SQ05. **Is now a good time to speak?***

[If yes, proceed to SQ05 script below]

If no… ***When would be a good time to talk?***

SQ06. Day to call back (DDMMYY) ____________

SQ07. Time to call back ______ AM/PM

OK, great. How about I give a quick overview of the study, and can then answer any questions you may have to see if it is a good fit for you. We will then go through a screening questionnaire to see if you qualify for the study. Does this sound OK?

A basic overview of the study is that it is looking to see if mindfulness practices improve blood pressure, and if education about hypertension risk factors may also improve blood pressure. Specifically, we will provide training in meditation, mindful movements, and the roles of things like diet, physical activity and medication in reducing blood pressure. You will be taught by a very experienced teacher who in an expert in these fields. The course takes place weekly for 9 weeks, where you come to a class once each week for 2.5 hours each time. There is also a one-day retreat on a Saturday that will be 8 hours long. Also, we will ask you to participate in health assessments before and after the study. Health assessments include measures such as blood pressure, height and weight, and questionnaires about your health and experiences.

Can I answer any questions about the study that you may have? [answer any they may have.]

SQ08. ***Does this study sound like something you would be interested in doing? Yes No***

[If yes, proceed to next statement below. If no, politely thank the participant for considering being in this study, and end the call].

Great. There are a few things that I would like to make clear before we start the interview. First of all, some of the questions that I will ask now to figure out if you are eligible to be in this study will be very personal and sensitive.

SQ09. ***Are you in a private place to talk?***

[If yes, proceed to text below. If no, reschedule meeting using variables SQ04 and SQ05 above].

Because this interview is of a personal nature, it is important that you understand that everything you say will be kept strictly confidential. No one outside of our project will ever be able to see your answers, and we will not keep your name in the same place as any of your answers. If you are not eligible after the phone screen, we will destroy your information. If you like, though, we can keep your information on file for future studies.

SQ10. Participant's First Name: ____________________________________

SQ11. Participant's Last Name:______________________________________

Participant's Address:
SQ12a. Street address: _____________
SQ12b. City: ____________________
SQ12c: State: ________
SQ12d: Zip Code: ________________

SQ13a. Participant's Phone number #1____________________________________

SQ13b. Participant's Phone number #2____________________________________

SQ14. Participant's email address (or mailing address if no email):______________________

SQ15. Notes from interviewer related to participants' contact information (if any):
____
____
____

PHONE SCREENING QUESTIONNAIRE PART 2

PID. Participant ID #: _______________

INCLUSION CRITERIA: All answers in 3rd column must be YES. If an answer is NO, immediately proceed to question SQ40.			
			Comments
SQ26.	***What is your age?*** (At least 18 years old)	YES NO	
SQ27.	***Can you read and write in English?***	YES NO	

EXCLUSION CRITERIA: All answers in 3rd column must be NO with the exception of SQ32a and SQ33a. If an answer (other than SQ32a and SQ33a) is YES, then immediately proceed to question SQ40.			
			Comments
SQ28.	***Has anyone ever told you that you have bipolar disorder or manic depression?***	**YES NO**	
SQ29.	***Has anyone ever used the word "Borderline" to describe you?***	**YES NO**	
SQ30a.<br><br>SQ30b.	***Have you ever had a hallucination or seen things that other people can't see, or hear things other people can't hear?***<br><br>***Have you ever been diagnosed with schizophrenia or psychosis?***	**YES NO**<br><br>**YES NO**	
 SQ31_1<br>SQ31_2<br>SQ31_3<br>SQ31_4<br>SQ31_5<br>SQ31_6<br>SQ31_7<br>SQ31_8	***Have you ever taken any of the following medications that I am about to read to you?***<br>***Lithium***<br>***Seroquel (quetiapine)***<br>***Abilify (aripiprazole)***<br>***Zyprexa (olanzapine)***<br>***Clozaril (clozapine)***<br>***Haldol/Haloperidol***<br>***Geodon (ziprasidone)***<br>***Risperdal (risperidone)***	 **YES NO**<br>**YES NO**<br>**YES NO**<br>**YES NO**<br>**YES NO**<br>**YES NO**<br>**YES NO**<br>**YES NO**	
SQ32a<br><br>SQ32b<br><br>SQ32c<br><br><br>SQ32d	***Have you ever had a suicide attempt?***<br><br>***[If yes, ask…] Have you considered killing yourself during the past month?***<br><br>***[If yes, ask…] Are you currently suicidal?***<br><br>***[If yes, keep participant on the phone, and follow suicide safety plan below]***<br><br>***[If no, ask…] Are you getting any help for that? If not, then provide list of resources from Safety Plan including Gateway, Anchor and The Providence Center] Not urgent, but inform Dr. Flynn about what was discussed.***	**YES NO**<br><br>**YES NO**<br><br><br>**YES NO**<br><br><br>**YES NO**	

EXCLUSION CRITERIA: All answers in 3rd column must be NO with the exception of SQ32a and SQ33a. If an answer (other than SQ32a and SQ33a) is YES, then immediately proceed to question SQ40.			
SQ33a<br><br><br>SQ33b<br><br><br>SQ33c	***Would you say you have a trauma history?***<br>***[If yes…]***<br>***In the past month, have you had any problems with dissociation (memory loss)?***<br>***In the past month, have you had any flashbacks (i.e. sudden and disturbing vivid memory) about the trauma?***	**YES NO**<br><br><br>**YES NO**<br><br><br>**YES NO**	
SQ34a<br><br><br><br>SQ34b	***In the past month, have you had any problems with obsessions or compulsions, such as washing your hands or checking the oven over and over again?***<br>***[If yes…] Has anyone diagnosed you with obsessive compulsive disorder?***	**YES NO**<br><br><br><br><br>**YES NO**	
SQ35	***In the past month, have you had a panic attack (i.e. sweating, heart palpitations, nausea, trouble breathing, fear of dying/choking/going crazy)?***	**YES NO**	
SQ36	***Have you had any problems with alcoholism or drug use in the past year?***	**YES NO**	
SQ37	***In the past year, have you had an eating disorder, such as starving, binge eating, or vomiting?***	**YES NO**	
SQ38a<br><br><br>SQ38b<br><br><br><br><br>SQ38c	***Do you currently have a mindfulness practice, such as meditation or yoga?***<br><br>***Please tell me more about your mindfulness practice, including how often you practice per week.***<br><br><br><br>***Do you currently practice meditation more than once per week? (yoga does not count as meditation in this context)***	**YES NO**<br><br>**Fill in response in comments section to the right**<br><br>**YES NO**	
SQ39	***This class will take place at Brown University in-person. Do you have any medical or mobility issues that would affect you being able to attend class?***	**YES NO**	
SQ40	Participant qualifies for next step of study (next step is 1st blood pressure screening)	**YES NO**<br><br>**If YES go to question SQ41.**<br><br>**If NO, go to question SQ42.**	

SQ41. ***Thank you for taking the time to answer these questions. You qualify for the next stage of screening, which is to take your blood pressure at two different times at least a week apart from each other. Let's book the in-person screening sessions*** [Book both sessions. We can cancel the second session if they do not qualify].

Thank you for taking the time to answer these questions, and for your interest in this study. We look forward to meeting you in person. Do you have any questions before we end this call?

SQ42a***. Thank you for taking the time to answer these questions. According to the survey, you do not qualify for the study at this time. There may be other studies you quality for.***

SQ42b. ***Would you like me to keep your information to pass on to these studies?*** YES / NO

SQ42c. [If yes…] ***OK, thank you. We will keep this information for future studies you may qualify for. Thank you for taking the time to talk with me today. Can I answer any questions before hanging up?***

SQ42d. [If no…] ***OK, our copy of this information will be destroyed. Thank you for taking the time to talk with me today. Can I answer any questions before hanging up?***

SAFETY PLAN

During the phone-based screening, if participants respond yes to “Are you currently suicidal?”, the interviewer should perform the following 2 steps:

1. **Immediately have 911 and Dr. Ellen Flynn called by a colleague who has been informed beforehand that this is a possibility.**

<u>Specifically, while keeping the participant on the phone, show the text below in bold italics to a colleague.</u>

I have a study participant on the phone who is currently suicidal. Please call 911 immediately, and tell them:

“I am calling on behalf of [my name] who is performing a research study at Brown University. She has a participant on the phone who says they are currently suicidal.” Please provide the participants’ contact information to the 911 operator (i.e. name, address, phone #, email address) as requested. This information is on page 1 of the screening questionnaire right here [interviewer has this information].

Finally, after calling 911, please call Dr. Ellen Flynn, who is the psychiatrist supporting this study. Her cell phone # is 401-258-9829. Please provide her with the same information as was done during the 911 call.

2. **While speaking calmly with the participant, let them know what you are doing. Specifically, let them know we are calling our study’s psychiatrist Dr. Flynn and 911, and why you are doing that (i.e. because we are concerned about you). You can speak with participant to keep him/her on the phone, but the discussion should not be clinical in nature.**

Examples of questions that could be asked in order to keep them on the phone:

- “Tell me what is going on.”
- “What’s happening right now?”
- Tell me more about why you are interesting in being part of this study.
- What are you hoping to get out of this study?

The following information can be provided to study participants if they state they have had considered killing themselves in the past month. If they are currently suicidal, the main priority is to keep them on the phone while 911 and Dr. Flynn are being contacted.

National Suicide Prevention Lifeline: 1-800-273-8255

Other options are to:

- Call your doctor’s office
- Call 911 for emergency services
- Go to the nearest hospital emergency room.

Local Non-Urgent Free or Inexpensive Mental Health Services:
Gateway Healthcare: 401-729-8701; Anchor Counseling Center: 401-475-9979;
The Providence Center: 401-276-4020

APPENDIX 2

FIRST IN-PERSON SCREENING ASSESSMENT

FIRST IN-PERSON VISIT SCREENING QUESTIONNAIRE AND ASSESSMENT FORMS

PID. Participant ID # __________
BA01. Staff ID # __________
BA02. Today's date (MMDDYY): ___________

Blood Pressure:
BA03a. Blood pressure 1st reading, systolic blood pressure: _________ mmHg
BA03b. Blood pressure 1st reading, diastolic blood pressure: __________ mmHg
BA03c. Blood pressure 2nd reading, systolic blood pressure: _________ mmHg
BA03d. Blood pressure 2nd reading, diastolic blood pressure: __________ mmHg
BA03e. Blood pressure 3rd reading, systolic blood pressure: _________ mmHg
BA03f. Blood pressure 3rd reading, diastolic blood pressure: __________ mmHg
BA03g. Blood pressure 4th reading, systolic blood pressure: _________ mmHg
BA03h. Blood pressure 4th reading, diastolic blood pressure: __________ mmHg
BA03i. Blood pressure 5th reading, systolic blood pressure: _________ mmHg
BA03j. Blood pressure 5th reading, diastolic blood pressure: __________ mmHg

BA04. Were the 4th and 5th systolic blood pressure readings within 20 mmHg of each other?
□ Yes
□ No ***(repeat measurements***

BA05. Were the 4th and 5th diastolic blood pressure readings within 10 mmHg of each other?
□ Yes
□ No ***(repeat measurements)***

BA06a. Repeated blood pressure 1st reading, systolic blood pressure: _________ mmHg
BA06b. Repeated blood pressure1st reading, diastolic blood pressure: __________ mmHg
BA06c. Repeated blood pressure2nd reading, systolic blood pressure: _________ mmHg
BA06d. Repeated blood pressure2nd reading, diastolic blood pressure: __________ mmHg
BA06e. Repeated blood pressure3rd reading, systolic blood pressure: _________ mmHg
BA06f. Repeated blood pressure3rd reading, diastolic blood pressure: __________ mmHg
BA06g. Repeated blood pressure4th reading, systolic blood pressure: _________ mmHg
BA06h. Repeated blood pressure4th reading, diastolic blood pressure: __________ mmHg
BA06i. Repeated blood pressure5th reading, systolic blood pressure: _________ mmHg
BA06j. Repeated blood pressure5th reading, diastolic blood pressure: __________ mmHg

BA07. Blood pressure cuff size used: □ S □ Reg □ L □ XL

BA08. Arm that cuff was placed on: □ L □ R

BA09. Height: ______ . __ cm (one decimal place)

BA10. Weight: ______ . __ kg (one decimal place)

Waist Circumference:
BA11a. Measurement 1 ______ . __ cm (one decimal place)
BA11b. Measurement 2 ______ . __ cm (one decimal place)

BA12. Is the difference between Measurement 1 and Measurement 2 greater than 1.0cm?

□ Yes ***(repeat measurements 3&4)***

□ No

BA13a. Measurement 3 ______ . __ cm

BA13b. Measurement 4 ______ . __ cm

Medications (ME)

ME01. Do you take any prescription medications or over-the-counter drugs?

- ☐ No → ***skip to end of medications questions***
- ☐ Yes
- ☐ Don't know
- ☐ Prefer not to answer

ME02a. What is the name of the first prescription medication or over-the-counter drug that you take?

☐ Label product name:

____

☐ Label generic name:

____

☐ Don't know ☐ Prefer not to answer

ME02b. What is the dosage form?

Oral

- ☐ Pill, tablet, or capsule
- ☐ Sublingual or orally-disintegrating tablet
- ☐ Liquid solution or suspension (drink, syrup)
- ☐ Powder

Topical

- ☐ Liquid, cream, gel, or ointment
- ☐ Ear drops (otic)
- ☐ Eye drops (ophthalmic)
- ☐ Skin patch (transdermal)

Inhaled

- ☐ Inhaler or nebulizer

Injected

- ☐ Injection

Suppository

- ☐ Rectal (e.g., enema)
- ☐ Vaginal (e.g., douche, pessary)

Other:

- ☐ Don't know
- ☐ Prefer not to answer

ME02c. How frequently do you take it?

- ☐ _________ times per day
- ☐ _________ times per week
- ☐ _________ times per month
- ☐ Don't know
- ☐ Prefer not to answer

ME02d. What is the strength? ***(Record strength of how it is actually taken, not how it is prescribed.)***

- ☐ _________ %
- ☐ _________ mg
- ☐ _________ mcg
- ☐ _________ grams
- ☐ _________ I.U.
- ☐ _________ Other unit: _____________
- ☐ Don't know
- ☐ Prefer not to answer

ME02e. Total dosage **per day.** ***(Record strength of how it is actually taken, not how it is prescribed.)***

- ☐ _________ %
- ☐ _________ mg
- ☐ _________ mcg
- ☐ _________ grams
- ☐ _________ I.U.
- ☐ _________ Other unit: _____________
- ☐ Don't know
- ☐ Prefer not to answer

ME02f. Do you take it regularly or only as needed?

- ☐ Regularly
- ☐ Only as needed
- ☐ Don't know
- ☐ Prefer not to answer

ME02g. For how long have you been taking it?

- ☐ For _________ days
- ☐ For _________ weeks
- ☐ For _________ months
- ☐ For _________ years
- ☐ Don't know
- ☐ Prefer not to answer

ME02h. ***Interviewer comments***:

____

ME02i. Do you take any other prescription medications or over-the-counter drugs?

- ☐ No → ***skip to end of medications questions***
- ☐ Yes
- ☐ Don't know
- ☐ Prefer not to answer

ME03a. What is the name of the next prescription medication or over-the-counter drug that you take?

☐ Label product name:

____

☐ Label generic name:

____

☐ Don't know ☐ Prefer not to answer

ME03b. What is the dosage form?

Oral

- ☐ Pill, tablet, or capsule
- ☐ Sublingual or orally-disintegrating tablet
- ☐ Liquid solution or suspension (drink, syrup)
- ☐ Powder

Topical

- ☐ Liquid, cream, gel, or ointment
- ☐ Ear drops (otic)
- ☐ Eye drops (ophthalmic)
- ☐ Skin patch (transdermal)

Inhaled

- ☐ Inhaler or nebulizer

Injected

- ☐ Injection

Suppository

- ☐ Rectal (e.g., enema)
- ☐ Vaginal (e.g., douche, pessary)

Other:

- ☐ Don't know
- ☐ Prefer not to answer

ME03c. How frequently do you take it?

- ☐ _________ times per day
- ☐ _________ times per week
- ☐ _________ times per month
- ☐ Don't know
- ☐ Prefer not to answer

ME03d. What is the strength? ***(Record strength of how it is actually taken, not how it is prescribed.)***

- ☐ _________ %
- ☐ _________ mg
- ☐ _________ mcg
- ☐ _________ grams
- ☐ _________ I.U.
- ☐ _________ Other unit: _____________
- ☐ Don't know
- ☐ Prefer not to answer

ME03e. Total dosage **per day.** ***(Record strength of how it is actually taken, not how it is prescribed.)***

- ☐ _________ %
- ☐ _________ mg
- ☐ _________ mcg
- ☐ _________ grams
- ☐ _________ I.U.
- ☐ _________ Other unit: _____________
- ☐ Don't know
- ☐ Prefer not to answer

ME3f. Do you take it regularly or only as needed?

- ☐ Regularly
- ☐ Only as needed
- ☐ Don't know
- ☐ Prefer not to answer

ME03g. For how long have you been taking it?

- ☐ For _________ days
- ☐ For _________ weeks
- ☐ For _________ months
- ☐ For _________ years

☐ Don't know ☐ Prefer not to answer

ME03h. ***Interviewer comments***:

____

ME03i. Do you take any other prescription medications or over-the-counter drugs?

☐ No → ***skip to end of medications questions***
☐ Yes
☐ Don't know
☐ Prefer not to answer

ME04a. What is the name of the next prescription medication or over-the-counter drug that you take?

☐ Label product name:

____

☐ Label generic name:

____

☐ Don't know ☐ Prefer not to answer

ME04b. What is the dosage form?

Oral
☐ Pill, tablet, or capsule
☐ Sublingual or orally-disintegrating tablet
☐ Liquid solution or suspension (drink, syrup)
☐ Powder

Topical
☐ Liquid, cream, gel, or ointment
☐ Ear drops (otic)
☐ Eye drops (ophthalmic)
☐ Skin patch (transdermal)

Inhaled
☐ Inhaler or nebulizer

Injected
☐ Injection

Suppository
☐ Rectal (e.g., enema)
☐ Vaginal (e.g., douche, pessary)

Other:
☐ Don't know
☐ Prefer not to answer

ME04c. How frequently do you take it?

☐ _________ times per day
☐ _________ times per week
☐ _________ times per month
☐ Don't know
☐ Prefer not to answer

ME04d. What is the strength? ***(Record strength of how it is actually taken, not how it is prescribed.)***

☐ _________ %
☐ _________ mg
☐ _________ mcg
☐ _________ grams
☐ _________ I.U.

☐ _________ Other unit: _____________
☐ Don't know
☐ Prefer not to answer

ME04e. Total dosage **per day.** ***(Record strength of how it is actually taken, not how it is prescribed.)***

☐ _________ %
☐ _________ mg
☐ _________ mcg
☐ _________ grams
☐ _________ I.U.
☐ _________ Other unit: _____________
☐ Don't know
☐ Prefer not to answer

ME04f. Do you take it regularly or only as needed?

☐ Regularly
☐ Only as needed
☐ Don't know
☐ Prefer not to answer

ME04g. For how long have you been taking it?

☐ For _________ days
☐ For _________ weeks
☐ For _________ months
☐ For _________ years
☐ Don't know
☐ Prefer not to answer

ME04h. ***Interviewer comments*****:**

____

ME04i. Do you take any other prescription medications or over-the-counter drugs?

☐ No → ***skip to end of medications section***
☐ Yes
☐ Don't know
☐ Prefer not to answer

ME05a. What is the name of the next prescription medication or over-the-counter drug that you take?

☐ Label product name:
____

☐ Label generic name:
____

☐ Don't know ☐ Prefer not to answer

ME05b. What is the dosage form?

Oral
☐ Pill, tablet, or capsule
☐ Sublingual or orally-disintegrating tablet
☐ Liquid solution or suspension (drink, syrup)
☐ Powder

Topical
☐ Liquid, cream, gel, or ointment
☐ Ear drops (otic)
☐ Eye drops (ophthalmic)
☐ Skin patch (transdermal)

Inhaled
☐ Inhaler or nebulizer

Injected
☐ Injection

Suppository
☐ Rectal (e.g., enema)
☐ Vaginal (e.g., douche, pessary)

Other:
☐ Don't know
☐ Prefer not to answer

ME05c. How frequently do you take it?
☐ _________ times per day
☐ _________ times per week
☐ _________ times per month
☐ Don't know
☐ Prefer not to answer

ME05d. What is the strength? ***(Record strength of how it is actually taken, not how it is prescribed.)***
☐ _________ %
☐ _________ mg
☐ _________ mcg
☐ _________ grams
☐ _________ I.U.
☐ _________ Other unit: _____________
☐ Don't know
☐ Prefer not to answer

ME05e. Total dosage **per day.** ***(Record strength of how it is actually taken, not how it is prescribed.)***
☐ _________ %
☐ _________ mg
☐ _________ mcg
☐ _________ grams
☐ _________ I.U.
☐ _________ Other unit: _____________
☐ Don't know
☐ Prefer not to answer

ME05f. Do you take it regularly or only as needed?
☐ Regularly
☐ Only as needed
☐ Don't know
☐ Prefer not to answer

ME05g. For how long have you been taking it?
☐ For _________ days
☐ For _________ weeks
☐ For _________ months
☐ For _________ years
☐ Don't know
☐ Prefer not to answer

ME05h. ***Interviewer comments***:
____

ME05i. Do you take any other prescription medications or over-the-counter drugs?

☐ No → ***skip to end of end of medications questions***

☐ Yes

☐ Don't know

☐ Prefer not to answer

ME06a. What is the name of the next prescription medication or over-the-counter drug that you take?

☐ Label product name: ______________________________

☐ Label generic name: ______________________________

☐ Don't know

☐ Prefer not to answer

ME06b. What is the dosage form?

<u>Oral</u>

☐ Pill, tablet, or capsule

☐ Sublingual or orally-disintegrating tablet

☐ Liquid solution or suspension (drink, syrup)

☐ Powder

<u>Topical</u>

☐ Liquid, cream, gel, or ointment

☐ Ear drops (otic)

☐ Eye drops (ophthalmic)

☐ Skin patch (transdermal)

<u>Inhaled</u>

☐ Inhaler or nebulizer

<u>Injected</u>

☐ Injection

<u>Suppository</u>

☐ Rectal (e.g., enema)

☐ Vaginal (e.g., douche, pessary)

<u>Other:</u>

☐ Don't know

☐ Prefer not to answer

ME06c. How frequently do you take it?

☐ _________ times per day

☐ _________ times per week

☐ _________ times per month

☐ Don't know

☐ Prefer not to answer

ME06d. What is the strength? ***(Record strength of how it is actually taken, not how it is prescribed.)***

☐ _________ %

☐ _________ mg

☐ _________ mcg

☐ _________ grams

☐ _________ I.U.

☐ _________ Other unit: _____________

☐ Don't know

☐ Prefer not to answer

ME06e. Total dosage **<u>per day.</u>** ***(Record strength of how it is actually taken, not how it is prescribed.)***

- ☐ _________ %
- ☐ _________ mg
- ☐ _________ mcg
- ☐ _________ grams
- ☐ _________ I.U.
- ☐ _________ Other unit: _____________
- ☐ Don't know
- ☐ Prefer not to answer

ME06e. Do you take it regularly or only as needed?

- ☐ Regularly
- ☐ Only as needed
- ☐ Don't know
- ☐ Prefer not to answer

ME06f. For how long have you been taking it?

- ☐ For _________ days
- ☐ For _________ weeks
- ☐ For _________ months
- ☐ For _________ years
- ☐ Don't know
- ☐ Prefer not to answer

ME06g. ***Interviewer comments***:

____

ME06h. Do you take any other prescription medications or over-the-counter drugs?

- ☐ No → *skip to next section*
- ☐ Yes
- ☐ Don't know
- ☐ Prefer not to answer

ME06i. Do you take any other prescription medications or over-the-counter drugs?

- ☐ No → ***skip to end of medications questions***
- ☐ Yes
- ☐ Don't know
- ☐ Prefer not to answer

ME07a. What is the name of the next prescription medication or over-the-counter drug that you take?

☐ Label product name:

____

☐ Label generic name:

____

- ☐ Don't know
- ☐ Prefer not to answer

ME07b. What is the dosage form?

Oral

- ☐ Pill, tablet, or capsule
- ☐ Sublingual or orally-disintegrating tablet
- ☐ Liquid solution or suspension (drink, syrup)
- ☐ Powder

Topical
☐ Liquid, cream, gel, or ointment
☐ Ear drops (otic)
☐ Eye drops (ophthalmic)
☐ Skin patch (transdermal)

Inhaled
☐ Inhaler or nebulizer

Injected
☐ Injection

Suppository
☐ Rectal (e.g., enema)
☐ Vaginal (e.g., douche, pessary)

Other:
☐ Don't know
☐ Prefer not to answer

ME07c. How frequently do you take it?
☐ _________ times per day
☐ _________ times per week
☐ _________ times per month
☐ Don't know
☐ Prefer not to answer

ME07d. What is the strength? ***(Record strength of how it is actually taken, not how it is prescribed.)***
☐ _________ %
☐ _________ mg
☐ _________ mcg
☐ _________ grams
☐ _________ I.U.
☐ _________ Other unit: _____________
☐ Don't know
☐ Prefer not to answer

ME07e. Total dosage **per day.** ***(Record strength of how it is actually taken, not how it is prescribed.)***
☐ _________ %
☐ _________ mg
☐ _________ mcg
☐ _________ grams
☐ _________ I.U.
☐ _________ Other unit: _____________
☐ Don't know
☐ Prefer not to answer

ME07f. Do you take it regularly or only as needed?
☐ Regularly
☐ Only as needed
☐ Don't know
☐ Prefer not to answer

ME07g For how long have you been taking it?
☐ For _________ days
☐ For _________ weeks
☐ For _________ months
☐ For _________ years
☐ Don't know
☐ Prefer not to answer

ME07h. ***Interviewer comments***:
____

ME07i. Do you take any other prescription medications or over-the-counter drugs?

- ☐ No → ***skip to end of medications questions***
- ☐ Yes
- ☐ Don't know
- ☐ Prefer not to answer

ME08a. What is the name of the next prescription medication or over-the-counter drug that you take?

- ☐ Label product name: ______________________________
- ☐ Label generic name: ______________________________
- ☐ Don't know
- ☐ Prefer not to answer

ME08b. What is the dosage form?

Oral
- ☐ Pill, tablet, or capsule
- ☐ Sublingual or orally-disintegrating tablet
- ☐ Liquid solution or suspension (drink, syrup)
- ☐ Powder

Topical
- ☐ Liquid, cream, gel, or ointment
- ☐ Ear drops (otic)
- ☐ Eye drops (ophthalmic)
- ☐ Skin patch (transdermal)

Inhaled
- ☐ Inhaler or nebulizer

Injected
- ☐ Injection

Suppository
- ☐ Rectal (e.g., enema)
- ☐ Vaginal (e.g., douche, pessary)

Other:
- ☐ Don't know
- ☐ Prefer not to answer

ME08c. How frequently do you take it?

- ☐ _________ times per day
- ☐ _________ times per week
- ☐ _________ times per month
- ☐ Don't know
- ☐ Prefer not to answer

ME08d. What is the strength? *(Record strength of how it is actually taken, not how it is prescribed.)*

- ☐ _________ %
- ☐ _________ mg
- ☐ _________ mcg
- ☐ _________ grams
- ☐ _________ I.U.
- ☐ _________ Other unit: _____________
- ☐ Don't know
- ☐ Prefer not to answer

ME08e. Total dosage **per day.** ***(Record strength of how it is actually taken, not how it is prescribed.)***

- ☐ _________ %
- ☐ _________ mg
- ☐ _________ mcg
- ☐ _________ grams
- ☐ _________ I.U.
- ☐ _________ Other unit: _____________
- ☐ Don’t know
- ☐ Prefer not to answer

ME08f. Do you take it regularly or only as needed?

- ☐ Regularly
- ☐ Only as needed
- ☐ Don’t know
- ☐ Prefer not to answer

ME08g. For how long have you been taking it?

- ☐ For _________ days
- ☐ For _________ weeks
- ☐ For _________ months
- ☐ For _________ years
- ☐ Don’t know
- ☐ Prefer not to answer

ME08h. ***Interviewer comments***:

____

ME08i. Do you take any other prescription medications or over-the-counter drugs?

- ☐ No → ***skip to end of medications questions***
- ☐ Yes
- ☐ Don’t know
- ☐ Prefer not to answer

ME09a. What is the name of the next prescription medication or over-the-counter drug that you take?

☐ Label product name:

____

☐ Label generic name:

____

☐ Don’t know ☐ Prefer not to answer

ME09b. What is the dosage form?

Oral

- ☐ Pill, tablet, or capsule
- ☐ Sublingual or orally-disintegrating tablet
- ☐ Liquid solution or suspension (drink, syrup)
- ☐ Powder

Topical

- ☐ Liquid, cream, gel, or ointment
- ☐ Ear drops (otic)
- ☐ Eye drops (ophthalmic)

☐ Skin patch (transdermal)

Inhaled

- ☐ Inhaler or nebulizer

Injected

- ☐ Injection

Suppository

- ☐ Rectal (e.g., enema)
- ☐ Vaginal (e.g., douche, pessary)

Other:

☐ Don't know

☐ Prefer not to answer

ME09c. How frequently do you take it?

☐ _________ times per day
☐ _________ times per week
☐ _________ times per month
☐ Don't know
☐ Prefer not to answer

ME09d. What is the strength? *(Record strength of how it is actually taken, not how it is prescribed.)*

☐ _________ %
☐ _________ mg
☐ _________ mcg
☐ _________ grams
☐ _________ I.U.
☐ _________ Other unit: _____________
☐ Don't know
☐ Prefer not to answer

ME09e. Total dosage **per day.** ***(Record strength of how it is actually taken, not how it is prescribed.)***

☐ _________ %
☐ _________ mg
☐ _________ mcg
☐ _________ grams
☐ _________ I.U.
☐ _________ Other unit: _____________
☐ Don't know
☐ Prefer not to answer

ME09f. Do you take it regularly or only as needed?

☐ Regularly
☐ Only as needed
☐ Don't know
☐ Prefer not to answer

ME09g. For how long have you been taking it?

☐ For _________ days
☐ For _________ weeks
☐ For _________ months
☐ For _________ years
☐ Don't know
☐ Prefer not to answer

ME09h. ***Interviewer comments***:

____

ME09i. Do you take any other prescription medications or over-the-counter drugs?

☐ No → ***skip to end of medications questions***
☐ Yes
☐ Don't know
☐ Prefer not to answer

ME10a. What is the name of the next prescription medication or over-the-counter drug that you take?

☐ Label product name:

____

☐ Label generic name:

____

☐ Don't know ☐ Prefer not to answer

ME10b. What is the dosage form?

Oral
☐ Pill, tablet, or capsule
☐ Sublingual or orally-disintegrating tablet
☐ Liquid solution or suspension (drink, syrup)
☐ Powder

Topical
☐ Liquid, cream, gel, or ointment
☐ Ear drops (otic)
☐ Eye drops (ophthalmic)
☐ Skin patch (transdermal)

Inhaled
☐ Inhaler or nebulizer

Injected
☐ Injection

Suppository
☐ Rectal (e.g., enema)
☐ Vaginal (e.g., douche, pessary)

Other:
☐ Don't know
☐ Prefer not to answer

ME10c. How frequently do you take it?

☐ _________ times per day
☐ _________ times per week
☐ _________ times per month

☐ Don't know
☐ Prefer not to answer

ME10d. What is the strength? ***(Record strength of how it is actually taken, not how it is prescribed.)***

☐ _________ %
☐ _________ mg
☐ _________ mcg
☐ _________ grams
☐ _________ I.U.

☐ _________ Other unit: _____________
☐ Don't know
☐ Prefer not to answer

ME10e. Total dosage **per day.** ***(Record strength of how it is actually taken, not how it is prescribed.)***

☐ _________ %
☐ _________ mg
☐ _________ mcg
☐ _________ grams
☐ _________ I.U.

☐ _________ Other unit: _____________
☐ Don't know
☐ Prefer not to answer

ME10f. Do you take it regularly or only as needed?

☐ Regularly
☐ Only as needed
☐ Don't know
☐ Prefer not to answer

ME10g. For how long have you been taking it?

☐ For _________ days
☐ For _________ weeks
☐ For _________ months
☐ For _________ years
☐ Don't know
☐ Prefer not to answer

ME10h. ***Interviewer comments***: ____

Questions for Participants to Answer on Their Own In-Person on Computer:
For each statement, please place a mark in the column that best describes how you have been feeling.

	Not at all or less than 1 day last week	1 or 2 days last week	3 to 4 days last week	5 to 7 days last week	Nearly every day for two weeks	I do not know	I prefer not to answer
DS_1. My appetite was poor.	☐	☐	☐	☐	☐	☐	☐
DS_2. I could not shake off the blues.	☐	☐	☐	☐	☐	☐	☐
DS_3. I had trouble keeping my mind on what I was doing.	☐	☐	☐	☐	☐	☐	☐
DS_4. I felt depressed.	☐	☐	☐	☐	☐	☐	☐
DS_5. My sleep was restless.	☐	☐	☐	☐	☐	☐	☐
DS_6. I felt sad.	☐	☐	☐	☐	☐	☐	☐
DS_7. I could not get going.	☐	☐	☐	☐	☐	☐	☐
DS_8. Nothing made me happy.	☐	☐	☐	☐	☐	☐	☐
C_9. I felt like a bad person.	☐	☐	☐	☐	☐	☐	☐
DS_10. I lost interest in my usual activities.	☐	☐	☐	☐	☐	☐	☐
DS_11. I slept much more than usual.	☐	☐	☐	☐	☐	☐	☐
DS_12. I felt like I was moving too slowly.	☐	☐	☐	☐	☐	☐	☐
DS_13. I felt fidgety.	☐	☐	☐	☐	☐	☐	☐
DS_14. I wished I were dead.	☐	☐	☐	☐	☐	☐	☐
DS_15. I wanted to hurt myself.	☐	☐	☐	☐	☐	☐	☐
DS_16. I was tired all the time.	☐	☐	☐	☐	☐	☐	☐
DS_17. I did not like myself.	☐	☐	☐	☐	☐	☐	☐
DS_18. I lost a lot of weight without trying to.	☐	☐	☐	☐	☐	☐	☐
DS_19. I had a lot of trouble getting to sleep.	☐	☐	☐	☐	☐	☐	☐
DS_20. I could not focus on the important things.	☐	☐	☐	☐	☐	☐	☐

Below is a list of common symptoms of anxiety. Please carefully read each item in the list. Indicate how much you have been bothered by that symptom during the past month, including today, by marking the box in the corresponding space in the column next to each symptom.

	Not At All	Mildly – it didn't bother me much	Moderately – it wasn't pleasant at all times	Severely – it bothered me a lot	I do not know	I prefer not to answer
BA_1. Numbness or tingling	☐	☐	☐	☐	☐	☐
BA_2. Feeling hot	☐	☐	☐	☐	☐	☐
BA_3. Wobbliness in legs	☐	☐	☐	☐	☐	☐
BA_4. Unable to relax	☐	☐	☐	☐	☐	☐
BA_5. Fear of worst happening	☐	☐	☐	☐	☐	☐
BA_6. Dizzy or lightheaded	☐	☐	☐	☐	☐	☐
BA_7. Heart pounding/racing	☐	☐	☐	☐	☐	☐
BA_8. Unsteady	☐	☐	☐	☐	☐	☐
BA_9. Terrified or afraid	☐	☐	☐	☐	☐	☐
BA_10. Nervous	☐	☐	☐	☐	☐	☐
BA_11. Feeling of choking	☐	☐	☐	☐	☐	☐
BA_12. Hands trembling	☐	☐	☐	☐	☐	☐
BA_13. Shaky/unsteady	☐	☐	☐	☐	☐	☐
BA_14. Fear of losing control	☐	☐	☐	☐	☐	☐
BA_15. Difficulty in breathing	☐	☐	☐	☐	☐	☐
BA_16. Fear of dying	☐	☐	☐	☐	☐	☐
BA_17. Scared	☐	☐	☐	☐	☐	☐
BA_18. Indigestion	☐	☐	☐	☐	☐	☐
BA_19. Faint/lightheaded	☐	☐	☐	☐	☐	☐
BA_20. Face flushed	☐	☐	☐	☐	☐	☐
BA_21. Hot/cold sweats	☐	☐	☐	☐	☐	☐

SAFETY PLAN

After the participant has completed the Beck Anxiety Inventory questionnaire and the Centers for Epidemiologic Studies Depressive Symptomatology questionnaire, ask the participant to wait for a few minutes while you check that all forms and assessments are completed. While the participant is waiting in a different room, check the scores according to the criteria below. If any scores trigger the safety plan, move forward with steps below as written.

Beck Anxiety Inventory (BA)

If participant scores ≥26 on the Beck Anxiety Inventory, while having the participant wait, immediately notify the collaborating psychiatrist, Dr. Ellen Flynn (cell phone # 401-258-9829), to determine need for a psychiatric consultation. She has provided her cell phone #, and your phone # will be entered into her phone recognition software to indicate it is a call from the MBHT study. It is likely she will answer the phone. If Dr. Flynn cannot be reached, please leave a phone message for her with your contact information and provide her with the Beck Anxiety Inventory results on the message. If Dr. Flynn is not immediately reachable, or following participant release after consulting with Dr. Flynn, immediately contact the PI, Dr. Eric Loucks (cell phone #401-369-0443).

Depressive Symptomatology (DS)

The CESD-R will be administered during the in-person assessment visits, and scores will be reviewed immediately upon completion of the in-person assessments.

1. Sadness (dysphoria): Question numbers 2,4, 6
2. Loss of Interest (anhedonia): Question numbers 8, 10
3. Appetite: Question numbers 1, 18
4. Sleep: Question numbers 5, 11, 19
5. Thinking / concentration: Question numbers 3, 20
6. Guilt (worthlessness): Question numbers 9, 17
7. Tired (fatigue): Question numbers 7, 16
8. Movement (agitation): Question numbers 12, 13
9. Suicidal ideation: Question numbers 14, 15

Participants are considered to meet criteria for major depressive episode if they have anhedonia or dysphoria nearly every day for the past two weeks, plus symptoms in an additional 4 DSM symptom groups noted as occurring nearly every day for the past two weeks. If participants meet criteria for major depressive episode, while having the participant wait, immediately notify the collaborating psychiatrist, Dr. Ellen Flynn (cell phone # 401-258-9829), to determine need for a psychiatric consultation. She has provided her cell phone #, and your phone # will be entered into her phone recognition software to indicate it is a call from the MBHT study. It is likely she will answer the phone. If Dr. Flynn cannot be reached, please leave a phone message for her with your contact information and provide her with the Depressive Symptomatology results on the message. If Dr. Flynn is not immediately reachable, or following participant release after consulting with Dr. Flynn, immediately contact the PI, Dr. Eric Loucks (cell phone #401-369-0443).

If participants respond having any suicidal ideation (DS questions 14 or 15), perform the following 2 steps:

1. **Immediately call 911 and Dr. Ellen Flynn.**

Specifically, while the participant is in the waiting room, ***call 911 immediately, and tell them:***

"My name is _____. I am working on a research study at Brown University. I have a study participant in the waiting room who has shared that he/she is currently suicidal." Please provide the participants' name to the 911 operator, as requested.

Finally, after calling 911, please call Dr. Ellen Flynn, who is the psychiatrist supporting this study. Her cell phone # is 401-258-9829. Please provide her with the same information as was done during the 911 call.

2. **While speaking calmly with the participant, let them know that you have called 911 and Dr. Flynn, and why (i.e. because we are concerned about you). You can speak with participant to keep him/her in the waiting room if 911 has sent assistance, but the discussion should not be clinical in nature.**

Examples of questions that could be asked in order to keep them in the waiting room:

- "Tell me what is going on."
- "What's happening right now?"
- Tell me more about why you are interesting in being part of this study.
- What are you hoping to get out of this study?

The following information can be provided to study participants.

National Suicide Prevention Lifeline: 1-800-273-8255

Other options are to:

- Call your doctor's office
- Call 911 for emergency services
- Go to the nearest hospital emergency room.

Local Non-Urgent Free or Inexpensive Mental Health Services:
Gateway Healthcare: 401-729-8701
Anchor Counseling Center: 401-475-9979
The Providence Center: 401-276-4020

APPENDIX 3

BASELINE QUESTIONNAIRE

QUESTIONNAIRES ANSWERED BY PARTICIPANTS AT BASELINE (VIA ONLINE OR PAPER FORM)

Questionnaire Table of Contents

Introductory Questions 3
Background Questions 5
Physical Activity 8
Diet 10
Eating Practices 16
Alcohol Consumption 18
Smoking 19
About You 20
Parent's Education 41
Your Childhood Experiences 43
Relationships 46
Your Sleep 49

Introduction
We appreciate you taking the time to participate in this research study. These questionnaires will ask a series of questions on various aspects of your health, health behaviours, family, and other life circumstances. It should take approximately one hour. Please keep in mind that you can refuse to answer any questions that you are not comfortable with.

Introductory Questions

IQ1_01. Please enter the 3-digit ID number you were given. ___ ___ ___.

IQ1_02. What is your main reason for participating in this study?

IQ1_03. What do you care about most?

IQ1_04. What gives you the most pleasure in your life?

IQ1_05. What are your greatest worries?

Please list three personal goals you have for taking this mindfulness program:

PG1_01.____
____
____
____
___

PG1_02. ____
____
____
____

PG1_03. ____
____
____
____
___

Background Questions

BQ1_01. How many years old are you?

BQ1_02. Are you Latino or Hispanic?

- □ No → ***skip to B3***
- □ Yes
- □ I do not know
- □ I prefer not to answer

B1_02a. Which of the following represents your family's country of origin? ***(check all that apply)***

- □ Cuba
- □ Mexico
- □ Puerto Rico
- □ Spain
- □ South America
- □ Columbia
- □ Dominican Republic
- □ Other Central American
- □ Other: ________________________
- □ I do not know
- □ I prefer not to answer

BQ1_03. If you were asked to put yourself into only one of these groups, in which one would you place yourself? ***(select one only):***

- □ Asian
- □ Pacific Islander
- □ African American/Black
- □ Caucasian/White
- □ Native American
- □ Other : ________________________
- □ I do not know
- □ I prefer not to answer

BQ1_04. Which of the following best describes your current work situation? ***(select one only)***

- □ Working full-time
- □ Working part-time
- □ Retired
- □ Unemployed: Looking for work
- □ Unemployed: Not currently looking for work
- □ Unemployed due to disability
- □ Keeping house or raising children full-time
- □ Military
- □ Full-time student
- □ Other:________________________
- □ I do not know
- □ I prefer not to answer

BQ1_05. What is the highest grade or level of regular school you have completed?

- □ Elementary School
- □ Junior High
- □ High School
- □ College
- □ Graduate School
- □ I do not know
- □ I prefer not to answer

BQ1_06. What is the highest degree you earned? ***(select <u>one</u> only)***

- □ Elementary school
- □ Some high school, but no GED
- □ GED
- □ High school
- □ Associate degree (Junior College)
- □ Bachelor's degree
- □ Master's degree
- □ Doctorate (PhD, EdD, etc)
- □ Professional (MD, JD, DDS, DVM, etc.)
- □ Other: ______________________________
- □ I do not know
- □ I prefer not to answer

BQ1_07. Did you ever attend any other school like a technical, vocational, or trade school?

- □ No
- □ Yes
- □ I do not know
- □ I prefer not to answer

BQ1_08. In total, about how many full-time years of education have you had, including 1st grade and all years of school after 1st grade?

________ years

BQ1_09a. Do you currently live alone?

□ No
□ Yes → ***skip to BQ_10***
□ I do not know
□ I prefer not to answer

BQ1_09b. How many people currently live in your household, including yourself?

____

BQ1_09c. Of these people, how many are under 18?

____

BQ1_09d. Of the adults in your household (including yourself), how many bring income into the household?

____

Physical Activity

Physical activities are activities where you move and increase your heart rate above its resting rate, whether you do them for pleasure, work, or transportation. The following questions ask about the amount and intensity of physical activity you usually do. The intensity of the activity is related to *the amount of energy* you use doing these activities.

Examples of physical activity intensity levels:

Light activities
Your heart beats slightly faster than normal
You can talk and sing

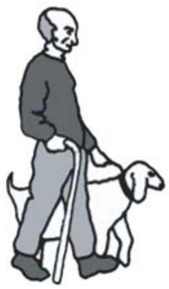
Light exercise

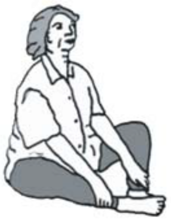
Light stretching

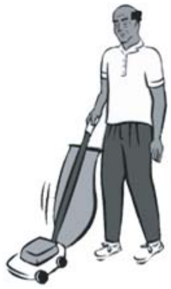
Light vacuuming or yard work

Moderate activities
Your heart beats faster than normal
You can talk but not sing

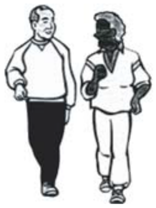
Brisk walking

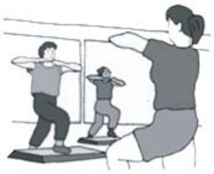
Aerobics class

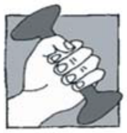
Strength training

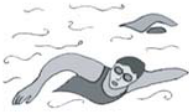
Swim gently

Vigorous activities
Your heart rate increases a lot
You can't talk, or your talking is broken up by large breaths

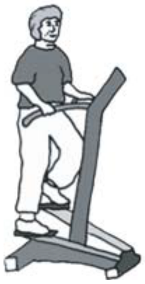
Aerobics classes

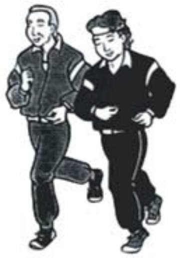
Jogging, Running, or Power Walking

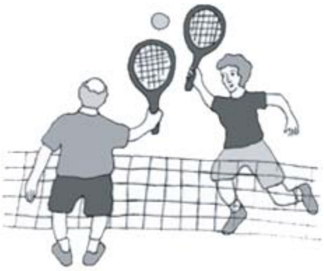
Singles tennis, Racquetball, Pickle ball

How physically active are you?

	YES	NO	I do not know	I prefer not to answer
PA1_01. I rarely or never do any physical activities.	☐	☐	☐	☐
PA1_02. I do some light and/or moderate physical activities, but not every week.	☐	☐	☐	☐
PA1_03. I do some light physical activity every week.	☐	☐	☐	☐
PA1_04. I do moderate physical activity every week but less than 5 days per week or less than 30 minutes on those days.	☐	☐	☐	☐
PA1_05. I do vigorous physical activities every week, but less than 3 days per week or less than 20 minutes on those days.	☐	☐	☐	☐
PA1_06. I do 30 minutes or more per day of moderate physical activities 5 or more days per week.	☐	☐	☐	☐
PA1_07. I do 20 minutes or more per day of vigorous physical activities 3 or more days per week.	☐	☐	☐	☐
PA1_08. I do activities to increase muscle strength, such as lifting weights or calisthenics, once a week or more.	☐	☐	☐	☐
PA1_09. I do activities to improve flexibility, such as stretching or yoga, once a week or more.	☐	☐	☐	☐

Diet

The first set of questions ask about foods you usually eat or drink. For each food, please place an X in the box (e.g. ☒) for the answer that best matches how often you eat or drink it. Remember to count fruit or vegetables that were part of another dish, like a banana on cereal or vegetables in a stew. Also, please pay attention to the serving sizes. For example, one serving size of bread is one slice, so a sandwich would contain 2 servings of bread. Please include all foods you eat, both at home and away from home. Enclosed are pictures of common serving sizes to assist you in filling out your questionnaire.

The secret to serving size is in your hand.

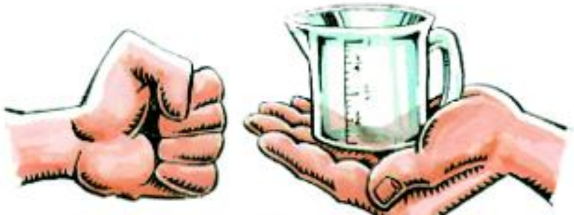

A fist or cupped hand = 1 cup

1 cup = 1½-2 servings of fruit juice
1 oz. of cold cereal
2 oz. of cooked cereal, rice or pasta
8 oz. of milk or yogurt

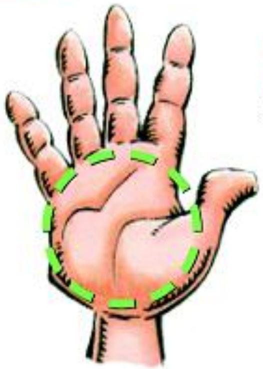

Palm = 3 oz. of meat

Choose lean poultry, fish, shellfish and beef. One palm size portion equals 3 oz. for an adult and 1½-2 oz. for a child under 5.

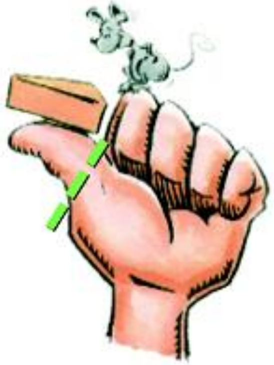

A thumb = 1 oz. of cheese

Consuming low-fat cheese helps you meet the required servings from the milk, yogurt and cheese group. 1½ oz. of low-fat cheese counts as 8 oz. of milk or yogurt.

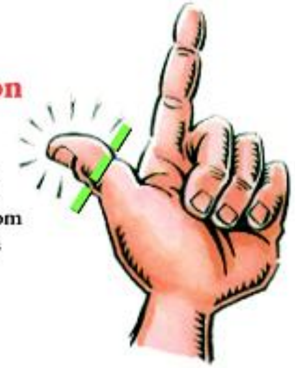

Thumb tip = 1 teaspoon

Keep high-fat foods, such as peanut butter and mayonnaise, at a minimum. One teaspoon is equal to the end of your thumb, from the knuckle up. Three teaspoons equals 1 tablespoon.

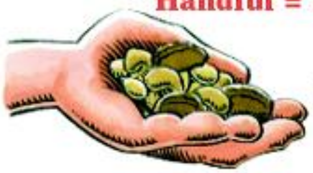

Handful = 1-2 oz. of snack food

Snacking can add up. Remember, 1 handful equals 1 oz. of nuts and small candies. For chips and pretzels, 2 handfuls equal 1 oz.

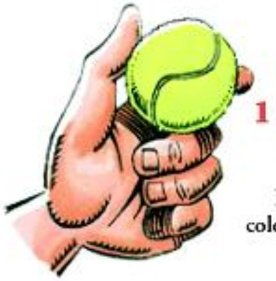

1 tennis ball = ½ cup of fruit and vegetables

Healthy diets include a variety of colorful fruits and vegetables every day.

Because hand sizes vary, compare your fist size to an actual measuring cup.

Iowa WIC Program - Iowa Department of Public Health - 2007
Adapted from North Carolina Nutrition Network

How Often You Eat or Drink This Item

	Never or less than 1 per month	1-3 per month	1 per week	2-4 per week	5-6 per week	1 per day	2-3 per day	4-5 per day	More than 5 per day	I do not know
**DT1_01**. Skim/low fat milk (8 oz. glass)	☐	☐	☐	☐	☐	☐	☐	☐	☐	☐
**DT1_02.** Whole milk (8 oz. glass)	☐	☐	☐	☐	☐	☐	☐	☐	☐	☐
**DT1_03.** Ice cream (1 cup)	☐	☐	☐	☐	☐	☐	☐	☐	☐	☐
**DT1_04.** Yogurt (1 cup)	☐	☐	☐	☐	☐	☐	☐	☐	☐	☐
**DT1_05.** Cottage or ricotta cheese (1/2 cup)	☐	☐	☐	☐	☐	☐	☐	☐	☐	☐
**DT1_06.** Butter, added to food or bread (1 pat), exclude use in cooking	☐	☐	☐	☐	☐	☐	☐	☐	☐	☐
**DT1_07.** Fruit (not including fruit juice. A serving = 1 piece, or 1/2 grapefruit, or 1/2 cup berries)	☐	☐	☐	☐	☐	☐	☐	☐	☐	☐
**DT1_08.** 100% fruit juice, not including fruit drinks (small glass)	☐	☐	☐	☐	☐	☐	☐	☐	☐	☐

How Often You Eat or Drink This Item

	Never or less than 1 per month	1-3 per month	1 per week	2-4 per week	5-6 per week	1 per day	2-3 per day	4-5 per day	More than 5 per day	I do not know	I prefer not to answer
**DT1_09.** Vegetables - raw and cooked (1/2 cup), including mixed dishes such as soups, casseroles and lasagna. Do not include potatoes.	☐	☐	☐	☐	☐	☐	☐	☐	☐	☐	☐
**DT1_10.** Eggs (do not include egg beaters) (1)	☐	☐	☐	☐	☐	☐	☐	☐	☐	☐	☐
**DT1_11.** Chicken or turkey (4-6 oz.)	☐	☐	☐	☐	☐	☐	☐	☐	☐	☐	☐
**DT1_12.** Processed meat (1 piece sausage, 1 slice salami, 2 pieces bacon)	☐	☐	☐	☐	☐	☐	☐	☐	☐	☐	☐
**DT1_13.** Hot dog/ hamburger	☐	☐	☐	☐	☐	☐	☐	☐	☐	☐	☐

How Often You Eat or Drink This Item

	Never or less than 1 per month	1-3 per month	1 per week	2-4 per week	5-6 per week	1 per day	2-3 per day	4-5 per day	More than 5 per day	I do not know	I prefer not to answer
**DT1_14**. Beef, pork, or lamb as a sandwich or mixed dish, e.g. stew, casserole, lasagna, etc.	☐	☐	☐	☐	☐	☐	☐	☐	☐	☐	☐
**DT1_15**. Beef, pork, or lamb as a main dish, e.g. steak, roast, ham, etc. (4-6 oz.)	☐	☐	☐	☐	☐	☐	☐	☐	☐	☐	☐
**DT1_16**. Fish (including canned tuna) (3-5 oz.)	☐	☐	☐	☐	☐	☐	☐	☐	☐	☐	☐
**DT1_17**. Shellfish such as shrimp, lobster, scallops and clams as a main dish	☐	☐	☐	☐	☐	☐	☐	☐	☐	☐	☐
**DT1_18**. White bread (including pita bread) (slice)	☐	☐	☐	☐	☐	☐	☐	☐	☐	☐	☐
**DT1_19.** Dark bread (including whole wheat and rye) (slice)	☐	☐	☐	☐	☐	☐	☐	☐	☐	☐	☐
**DT1_20.** Bakery items (cookies, brownies, doughnuts, cake) (1 piece or slice)	☐	☐	☐	☐	☐	☐	☐	☐	☐	☐	☐
**DT1_21.** Nuts (small packet, 1 oz., 1 Tbs peanut butter)	☐	☐	☐	☐	☐	☐	☐	☐	☐	☐	☐
**DT1_22.** Pasta (e.g. spaghetti, and macaroni and cheese; 1 cup cooked)	☐	☐	☐	☐	☐	☐	☐	☐	☐	☐	☐
**DT1_23.** Potatoes, baked, boiled or mashed (do not include French fries/ chips) (1 potato or 1 cup)	☐	☐	☐	☐	☐	☐	☐	☐	☐	☐	☐

How Often You Eat or Drink This Item

	Never or less than 1 per month	1-3 per month	1 per week	2-4 per week	5-6 per week	1 per day	2-3 per day	4-5 per day	More than 5 per day	I do not know	I prefer not to answer
**DT**1_**24**. Regular soft drinks (including caffeine free, but NOT including diet colas) (consider the serving size as 1 glass, bottle or can)	☐	☐	☐	☐	☐	☐	☐	☐	☐	☐	☐
**DT**1_**25**. Coffee (regular or decaf) (1 cup)	☐	☐	☐	☐	☐	☐	☐	☐	☐	☐	☐

Eating Practices

	Definitely True	Mostly True	Mostly False	Definitely False	I Do Not Know	I Prefer Not to Answer
EE1_01. I deliberately take small helpings to control my weight.	☐	☐	☐	☐	☐	☐
EE1_02. I start to eat when I feel anxious.	☐	☐	☐	☐	☐	☐
EE1_03. Sometimes when I start eating, I just can't seem to stop.	☐	☐	☐	☐	☐	☐
EE1_04. When I feel sad, I often eat too much.	☐	☐	☐	☐	☐	☐
EE1_05. I don't eat some foods because they make me fat.	☐	☐	☐	☐	☐	☐
EE1_06. Being with someone who is eating, often makes me want to also eat.	☐	☐	☐	☐	☐	☐
EE1_07. When I feel tense or ''wound up'', I often feel I need to eat.	☐	☐	☐	☐	☐	☐
EE1_08. I often get so hungry that my stomach feels like a bottomless pit.	☐	☐	☐	☐	☐	☐
EE1_09. I'm always so hungry that it's hard for me to stop eating before finishing all of the food on my plate.	☐	☐	☐	☐	☐	☐
EE1_10. When I feel lonely, I console myself by eating.	☐	☐	☐	☐	☐	☐
EE1_11. I consciously hold back on how much I eat at meals to keep from gaining weight.	☐	☐	☐	☐	☐	☐
EE1_12. When I smell a sizzling steak or see a juicy piece of meat, I find it very difficult to keep from eating even if I've just finished a meal.	☐	☐	☐	☐	☐	☐
EE1_13. I'm always hungry enough to eat at any time.	☐	☐	☐	☐	☐	☐
EE1_14. If I feel nervous, I try to calm down by eating.	☐	☐	☐	☐	☐	☐
EE1_15. When I see something that looks very delicious, I often get so hungry that I have to eat right away.	☐	☐	☐	☐	☐	☐
EE1_16. When I feel depressed, I want to eat.	☐	☐	☐	☐	☐	☐

	Almost Never	Seldom	Usually	Almost Always	I Do Not Know	I Prefer Not to Answer
EE1_17. How often do you avoid ‘stocking up’’ on tempting foods?	☐	☐	☐	☐	☐	☐

	Unlikely	A little likely	Somewhat likely	Very likely	I Do Not Know	I Prefer Not to Answer
EE1_18. How likely are you to make an effort to eat less than you want?	☐	☐	☐	☐	☐	☐

	Never	Rarely	Sometimes	At least once a week	I Do Not Know	I Prefer Not to Answer
EE1_19. Do you go on eating binges even though you’re not hungry?	☐	☐	☐	☐	☐	☐

	Only at mealtimes	Sometimes between meals	Often between meals	Almost always	I Do Not Know	I Prefer Not to Answer
EE1_20. How often do you feel hungry?	☐	☐	☐	☐	☐	☐

	1	2	3	4	5	6	7	8	I Do Not Know	I Prefer Not to Answer
EE1_21. On a scale from 1 to 8, where 1 means no restraint in eating and 8 means total restraint, what number would you give yourself? Mark the number that best applies to you:	☐	☐	☐	☐	☐	☐	☐	☐	☐	☐

Alcohol Consumption

A drink of alcohol is defined as 1 can or bottle of beer, 1 glass of wine, 1 can or bottle of wine cooler, 1 cocktail, or 1 shot of liquor.

AC1_01. During the past 30 days, how many days per week or per month did you have at least 1 drink of any alcoholic beverage? [if none, **STOP**]

____

AC1_02. On the days when you drank, about how many drinks did you drink on average?

____

AC1_03. ***Men:*** Considering all types of alcoholic beverages, how many times during the past 30 days did you have **5** or more drinks on an occasion?

Women: Considering all types of alcoholic beverages, how many times during the past 30 days did you have ***4*** or more drinks on an occasion?

____

Smoking

SM1_01. Have you smoked at least 100 cigarettes in your entire life?

- ☐ Yes
- ☐ No
- ☐ I Do Not Know
- ☐ Prefer not to answer

SM1_02. Did you ever become a daily smoker (that is, smoke every day or nearly every day for two months or longer)?

- ☐ Yes
- ☐ No → ***skip to SC1_01***
- ☐ I Do Not Know
- ☐ Prefer not to answer

SM1_03. How old were you when you last smoked daily?

Age ______ (in years)

☐ I Do Not Know ☐ Prefer not to answer ☐ Still smoking daily

SM1_04. Do you smoke cigarettes now?

- ☐ Yes
- ☐ No → ***skip to next section***
- ☐ I Do Not Know
- ☐ Prefer not to answer

SM1_04a. How many cigarettes per day do you smoke? (One pack equals 20 cigarettes)

Number of cigarettes ________

- ☐ I Do Not Know
- ☐ Prefer not to answer

About You

Using the scale provided, please indicate how much each of the following statements reflects how you typically are.

	Not at all	A little	Somewhat	Fair Amount	Very much	I do not know	I prefer not to answer
SC1_01. I am good at resisting temptation.	☐	☐	☐	☐	☐	☐	☐
SC1_02. I have a hard time breaking bad habits.	☐	☐	☐	☐	☐	☐	☐
SC1_03. I am lazy.	☐	☐	☐	☐	☐	☐	☐
SC1_04. I say inappropriate things.	☐	☐	☐	☐	☐	☐	☐
SC1_05. I do certain things that are bad for me, if they are fun.	☐	☐	☐	☐	☐	☐	☐
SC1_06. I refuse things that are bad for me.	☐	☐	☐	☐	☐	☐	☐
SC1_07. I wish I had more self-discipline.	☐	☐	☐	☐	☐	☐	☐
SC1_08. People would say that I have iron self-discipline.	☐	☐	☐	☐	☐	☐	☐
SC1_09. Pleasure and fun sometimes keep me from getting work done.	☐	☐	☐	☐	☐	☐	☐
SC1_10. I have trouble concentrating.	☐	☐	☐	☐	☐	☐	☐
SC1_11. I am able to work effectively toward long-term goals.	☐	☐	☐	☐	☐	☐	☐
SC1_12. Sometimes I can’t stop myself from doing something, even if I know it is wrong.	☐	☐	☐	☐	☐	☐	☐
SC1_13. I often act without thinking through all the alternatives.	☐	☐	☐	☐	☐	☐	☐

The next few statements are about certain ways you may feel about your life. Please indicate how strongly you agree or disagree with each of the following statements: Strongly agree; Agree; Neutral, Disagree, or Strongly disagree

	Strongly agree	Agree	Neutral	Disagree	Strongly disagree	I Do Not Know	Prefer not to answer
**SE**1_**01**. I have little control over the things that happen to me.	☐	☐	☐	☐	☐	☐	☐
**SE**1_**02**. There is really no way I can solve some of the problems I have.	☐	☐	☐	☐	☐	☐	☐
**SE**1_**03**. There is little I can do to change many of the important things in my life.	☐	☐	☐	☐	☐	☐	☐
**SE**1_**04**. I often feel helpless in dealing with the problems of life.	☐	☐	☐	☐	☐	☐	☐
**SE**1_**05**. Sometimes I feel that I am being pushed around in life.	☐	☐	☐	☐	☐	☐	☐
**SE**1_**06**. What happens to me in the future mostly depends on me.	☐	☐	☐	☐	☐	☐	☐
**SE**1_**07**. I can do just about anything I really set my mind to do.	☐	☐	☐	☐	☐	☐	☐

Please read each statement carefully before answering. To the left of each item, indicate how often you behave in the stated manner, using the following scale:

	Almost never	Not very often	Sometimes	Frequently	Almost always	I Do Not Know	Prefer not to answer
C1_O1. When I fail at something important to me, I become consumed by feelings of inadequacy.							
C1_O2. I try to be understanding and patient towards those aspects of my personality I don't like.							
C1_O3. When something painful happens I try to take a balanced view of the situation.							
C1_O4. When I'm feeling down, I tend to feel like most other people are probably happier than I am.							
C1_O5. I try to see my failings as part of the human condition.							
C1_O6. When I'm going through a very hard time, I give myself the caring and tenderness I need.							
C1_O7. When something upsets me I try to keep my emotions in balance.							
C1_O8. When I fail at something that's important to me, I tend to feel alone in my failure							
C1_O9. When I'm feeling down I tend to obsess and fixate on everything that's wrong.							

	Almost never	Not very often	Sometimes	Frequently	Almost always	I Do Not Know	Prefer not to answer
CO1_10. When I feel inadequate in some way, I try to remind myself that feelings of inadequacy are shared by most people.							
CO1_11. I'm disapproving and judgmental about my own flaws and inadequacies.							
CO1_12. I'm intolerant and impatient towards those aspects of my personality I don't like.							

The questions in this scale ask you about your feelings and thoughts during the last month. In each case, you will be asked to indicate by circling how often you felt or thought a certain way.

	Never	Almost Never	Sometimes	Fairly Often	Often	I do not know	I prefer not to answer
PS1_01. In the last month, how often have you been upset because of something that happened unexpectedly?	☐	☐	☐	☐	☐	☐	☐
PS1_02. In the last month, how often have you felt that you were unable to control the important things in your life?	☐	☐	☐	☐	☐	☐	☐
PS1_03. In the last month, how often have you felt nervous and "stressed"?	☐	☐	☐	☐	☐	☐	☐
PS1_04. In the last month, how often have you felt confident about your ability to handle your personal problems?	☐	☐	☐	☐	☐	☐	☐
PS1_05. In the last month, how often have you felt that things were going your way?	☐	☐	☐	☐	☐	☐	☐
PS1_06. In the last month, how often have you found that you could not cope with all the things that you had to do?	☐	☐	☐	☐	☐	☐	☐
PS1_07. In the last month, how often have you been able to control irritations in your life?	☐	☐	☐	☐	☐	☐	☐
PS1_08. In the last month, how often have you felt that you were on top of things?	☐	☐	☐	☐	☐	☐	☐
PS1_09. In the last month, how often have you been angered because of things that were outside of your control?	☐	☐	☐	☐	☐	☐	☐
PS1_10. In the last month, how often have you felt difficulties were piling up so high that you could not overcome them?	☐	☐	☐	☐	☐	☐	☐

Instructions: Below is a collection of statements about your everyday experience. Using the scale below, please indicate how frequently or infrequently you currently have each experience. Please answer according to what really reflects your experience rather than what you think your experience should be. Please treat each item separately from every other item.

Please indicate the degree to which you agree with each of the following items using the scale below. Simply check your response to each item

	**Almost always**	**Very frequently**	**Somewhat frequently**	**Somewhat infrequently**	**Very infrequently**	**Almost never**	**I do not know**	**I prefer not to answer**
MA1_01. I could be experiencing some emotion and not be conscious of it until some time later.	☐	☐	☐	☐	☐	☐	☐	☐
MA1_02. I break or spill things because of carelessness, not paying attention, or thinking of something else.	☐	☐	☐	☐	☐	☐	☐	☐
MA1_03. I find it difficult to stay focused on what's happening in the present.	☐	☐	☐	☐	☐	☐	☐	☐
MA1_04. I tend to walk quickly to get where I'm going without paying attention to what I experience along the way.	☐	☐	☐	☐	☐	☐	☐	☐
MA1_05. I tend not to notice feelings of physical tension or discomfort until they really grab my attention.	☐	☐	☐	☐	☐	☐	☐	☐
MA1_06. I forget a person's name almost as soon as I've been told it for the first time.	☐	☐	☐	☐	☐	☐	☐	☐

	Almost always	Very frequently	Somewhat frequently	Somewhat infrequently	Very infrequently	Almost never	I do not know	I prefer not to answer
MA1_07. It seems I am "running on automatic" without much awareness of what I'm doing.	☐	☐	☐	☐	☐	☐	☐	☐
MA1_08. I rush through activities without being really attentive to them.	☐	☐	☐	☐	☐	☐	☐	☐
MA1_09. I get so focused on the goal I want to achieve that I lose touch with what I am doing right now to get there.	☐	☐	☐	☐	☐	☐	☐	☐
MA1_10. I do jobs or tasks automatically, without being aware of what I'm doing.	☐	☐	☐	☐	☐	☐	☐	☐
MA1_11. I find myself listening to someone with one ear, doing something else at the same time.	☐	☐	☐	☐	☐	☐	☐	☐
MA1_12. I drive places on "automatic pilot" and then wonder why I went there.	☐	☐	☐	☐	☐	☐	☐	☐
MA1_13. I find myself preoccupied with the future or the past.	☐	☐	☐	☐	☐	☐	☐	☐
MA1_14. I find myself doing things without paying attention.	☐	☐	☐	☐	☐	☐	☐	☐
MA1_15. I snack without being aware that I'm eating.	☐	☐	☐	☐	☐	☐	☐	☐

Please rate each of the following statements using the scale provided. Write the number in the blank that best describes *your own opinion* of what is *generally true for you*.

	Never	Almost Never	Sometimes	Fairly Often	Often	I do not know	I prefer not to answer
FF1_01. When I'm walking, I deliberately notice the sensations of my body moving.	☐	☐	☐	☐	☐	☐	☐
FF1_02. I'm good at finding words to describe my feelings.	☐	☐	☐	☐	☐	☐	☐
FF1_03. I criticize myself for having irrational or inappropriate emotions.	☐	☐	☐	☐	☐	☐	☐
FF1_04. I perceive my feelings and emotions without having to react to them.	☐	☐	☐	☐	☐	☐	☐
FF1_05. When I do things, my mind wanders off and I'm easily distracted.	☐	☐	☐	☐	☐	☐	☐
FF1_06. When I take a shower or bath, I stay alert to the sensations of water on my body.	☐	☐	☐	☐	☐	☐	☐
FF1_07. I can easily put my beliefs, opinions, and expectations into words.	☐	☐	☐	☐	☐	☐	☐
FF1_08. I don't pay attention to what I'm doing because I'm daydreaming, worrying, or otherwise distracted.	☐	☐	☐	☐	☐	☐	☐
FF1_09. I watch my feelings without getting lost in them.	☐	☐	☐	☐	☐	☐	☐
FF1_10. I tell myself I shouldn't be feeling the way I'm feeling.	☐	☐	☐	☐	☐	☐	☐
FF1_11. I notice how foods and drinks affect my thoughts, bodily sensations, and emotions.	☐	☐	☐	☐	☐	☐	☐
FF1_12. It's hard for me to find the words to describe what I'm thinking.	☐	☐	☐	☐	☐	☐	☐

	Never	Almost Never	Sometimes	Fairly Often	Often	I do not know	I prefer not to answer
FF1_13. I am easily distracted.	☐	☐	☐	☐	☐	☐	☐
FF1_14. I believe some of my thoughts are abnormal or bad and I shouldn't think that way.	☐	☐	☐	☐	☐	☐	☐
FF1_15. I pay attention to sensations, such as the wind in my hair or sun on my face.	☐	☐	☐	☐	☐	☐	☐
FF1_16. I have trouble thinking of the right words to express how I feel about things.	☐	☐	☐	☐	☐	☐	☐
FF1_17. I make judgments about whether my thoughts are good or bad.	☐	☐	☐	☐	☐	☐	☐
FF1_18. I find it difficult to stay focused on what's happening in the present.	☐	☐	☐	☐	☐	☐	☐
FF1_19. When I have distressing thoughts or images, I "step back" and am aware of the thought or image without getting taken over by it.	☐	☐	☐	☐	☐	☐	☐
FF1_20. I pay attention to sounds, such as clocks ticking, birds chirping, or cars passing.	☐	☐	☐	☐	☐	☐	☐
FF1_21. In difficult situations, I can pause without immediately reacting.	☐	☐	☐	☐	☐	☐	☐
FF1_22. When I have a sensation in my body, it's difficult for me to describe it because I can't find the right words.	☐	☐	☐	☐	☐	☐	☐
FF1_23. It seems I am "running on automatic" without much aware- ness of what I'm doing.	☐	☐	☐	☐	☐	☐	☐
FF1_24. When I have distressing thoughts or images, I feel calm soon after.	☐	☐	☐	☐	☐	☐	☐
FF1_25. I tell myself that I shouldn't be thinking the way I'm thinking.	☐	☐	☐	☐	☐	☐	☐

	Never	Almost Never	Sometimes	Fairly Often	Often	I do not know	I prefer not to answer
FF1_26. I notice the smells and aromas of things.	☐	☐	☐	☐	☐	☐	☐
FF1_27. Even when I'm feeling terribly upset, I can find a way to put it into words.	☐	☐	☐	☐	☐	☐	☐
FF1_28. I rush through activities without being really attentive to them.	☐	☐	☐	☐	☐	☐	☐
FF1_29. When I have distressing thoughts or images, I am able just to notice them without reacting.	☐	☐	☐	☐	☐	☐	☐
FF1_30. I think some of my emotions are bad or inappropriate and I shouldn't feel them.	☐	☐	☐	☐	☐	☐	☐
FF1_31. I notice visual elements in art or nature, such as colors, shapes, textures, or patterns of light and shadow.	☐	☐	☐	☐	☐	☐	☐
FF1_32. My natural tendency is to put my experiences into words.	☐	☐	☐	☐	☐	☐	☐
FF1_33. When I have distressing thoughts or images, I just notice them and let them go.	☐	☐	☐	☐	☐	☐	☐
FF1_34. I do jobs or tasks automatically without being aware of what I'm doing.	☐	☐	☐	☐	☐	☐	☐
FF1_35. When I have distressing thoughts or images, I judge myself as good or bad depending what the thought or image is about.	☐	☐	☐	☐	☐	☐	☐
FF1_36. I pay attention to how my emotions affect my thoughts and behavior.	☐	☐	☐	☐	☐	☐	☐
FF1_37. I can usually describe how I feel at the moment in consider- able detail.	☐	☐	☐	☐	☐	☐	☐

	Never	Almost Never	Someti mes	Fairly Often	Often	I do not know	I prefer not to answer
FF1_38. I find myself doing things without paying attention.	☐	☐	☐	☐	☐	☐	☐
FF1_39. I disapprove of myself when I have irrational ideas.	☐	☐	☐	☐	☐	☐	☐

This Week's Experiences

Instructions: Below is a collection of statements about how you find yourself reacting to unpleasant thoughts and feelings. Using the scale below, please indicate how frequently or infrequently you experienced each of the following reactions this week**. Please answer according to what *really reflects your experience this week* rather than what you think your experience should be.**

This week, when I encountered an unpleasant thought or feeling,

	Almost Never	Very Infrequently	Somewhat Infrequently	Somewhat Frequently	Very Frequently	Almost Always	I do not know	I prefer not to answer
MS1_01. I tried to think pleasant thoughts instead	☐	☐	☐	☐	☐	☐	☐	☐
MS1_02. I allowed myself to experience it	☐	☐	☐	☐	☐	☐	☐	☐
MS1_03. I got angry or upset at myself for having the thought/emotion	☐	☐	☐	☐	☐	☐	☐	☐
MS1_04. I felt the desire to make it better or make it go away	☐	☐	☐	☐	☐	☐	☐	☐
MS1_05. I wished I didn't feel or think that way	☐	☐	☐	☐	☐	☐	☐	☐
MS1_06. I tried to let my thoughts just come and go without getting too entangled with them	☐	☐	☐	☐	☐	☐	☐	☐

	Almost Never	Very Infrequently	Somewhat Infrequently	Somewhat Frequently	Very Frequently	Almost Always	I do not know	I prefer not to answer
MS1_07. I thought the mood would never change	☐	☐	☐	☐	☐	☐	☐	☐
MS1_08. I explored the body sensations that accompanied the emotion.	☐	☐	☐	☐	☐	☐	☐	☐
MS1_09. I named the emotion over and over as long as it lasted	☐	☐	☐	☐	☐	☐	☐	☐
MS1_10. I turned to work or other activities to take my mind off things.	☐	☐	☐	☐	☐	☐	☐	☐
MS1_11. I named the emotion and then redirected my attention to the present moment	☐	☐	☐	☐	☐	☐	☐	☐
MS1_12. I thought "This mood, too, shall pass"	☐	☐	☐	☐	☐	☐	☐	☐

	Almost Never	Very Infrequently	Somewhat Infrequently	Somewhat Frequently	Very Frequently	Almost Always	I do not know	I prefer not to answer
MS1_13. I focused on my breathi	☐	☐	☐	☐	☐	☐	☐	☐
MS1_14. I took an active interest in how the experience changed over time	☐	☐	☐	☐	☐	☐	☐	☐
MS1_15. I explored my reactions to the thought or emotion	☐	☐	☐	☐	☐	☐	☐	☐
MS1_16. I thought "my thinking is being distorted by my mood"	☐	☐	☐	☐	☐	☐	☐	☐
MS1_17. Think "Why do I have problems that other people don't have?"	☐	☐	☐	☐	☐	☐	☐	☐
MS1_18. I forgave myself for having the thought/emotion	☐	☐	☐	☐	☐	☐	☐	☐
MS1_19. I challenged the thought's validity	☐	☐	☐	☐	☐	☐	☐	☐
MS1_20. I asked myself questions about the experience in order to explore it better	☐	☐	☐	☐	☐	☐	☐	☐

	Almost Never	Very Infrequently	Somewhat Infrequently	Somewhat Frequently	Very Frequently	Almost Always	I do not know	I prefer not to answer
MS1_21. I tried not to change it because I believe it is important to experience	☐	☐	☐	☐	☐	☐	☐	☐
MS1_22. I labelled my thoughts "images" or verbal "talk"	☐	☐	☐	☐	☐	☐	☐	☐
MS1_23. I thought "Why do I always react this way?"	☐	☐	☐	☐	☐	☐	☐	☐
MS1_24. I adopted a welcoming stance toward the emotion/thought	☐	☐	☐	☐	☐	☐	☐	☐
MS1_25. I went somewhere alone to think about my feelings	☐	☐	☐	☐	☐	☐	☐	☐
MS1_26. I sent out compassion to all people who struggle with this emotion	☐	☐	☐	☐	☐	☐	☐	☐
MS1_27. I restrained myself from doing anything too quickly	☐	☐	☐	☐	☐	☐	☐	☐

	Almost Never	Very Infrequently	Somewhat Infrequently	Somewhat Frequently	Very Frequently	Almost Always	I do not know	I prefer not to answer
MS1_28. I became genuinely curious about the thought/emotion	☐	☐	☐	☐	☐	☐	☐	☐
MS1_29. I laughed or kidded myself about the situation	☐	☐	☐	☐	☐	☐	☐	☐
MS1_30. I treated the repetitive thought like a “top ten radio tune” that's playing in the background	☐	☐	☐	☐	☐	☐	☐	☐
MS1_31. I talked to others about how I was feeling	☐	☐	☐	☐	☐	☐	☐	☐
MS1_32. I tried to forget about it	☐	☐	☐	☐	☐	☐	☐	☐
MS1_33. I thought "Why can't I handle things better"	☐	☐	☐	☐	☐	☐	☐	☐
MS1_34. I tried to come up with a way to make it go away	☐	☐	☐	☐	☐	☐	☐	☐
MS1_35. I said to myself "thinking" or "this is just a thought"	☐	☐	☐	☐	☐	☐	☐	☐
MS1_36. I thought about how much I disliked feeling that way	☐	☐	☐	☐	☐	☐	☐	☐
MS1_37. I reminded myself thoughts are not accurate reflections of reality	☐	☐	☐	☐	☐	☐	☐	☐

	Almost Never	Very Infrequently	Somewhat Infrequently	Somewhat Frequently	Very Frequently	Almost Always	I do not know	I prefer not to answer
MS1_38. I thought that that I must be headed into a downward spiral	☐	☐	☐	☐	☐	☐	☐	☐
MS1_39. I thought "What I am doing to deserve this?	☐	☐	☐	☐	☐	☐	☐	☐

Please indicate how often the following statements apply to you by checking the box that best describes your experience.

	Almost Never (0-10%)	Sometimes (11-35%)	About Half The Time (36-65%)	Most of the Time (66-90%)	Almost Always (91-100%)	I Do Not Know	I Prefer Not to Answer
ER1_01. I am clear about my feelings.	☐	☐	☐	☐	☐	☐	☐
ER1_02. I pay attention to how I feel.	☐	☐	☐	☐	☐	☐	☐
ER1_03. I experience my emotions as overwhelming and out of control.	☐	☐	☐	☐	☐	☐	☐
ER1_04. I have no idea how I am feeling.	☐	☐	☐	☐	☐	☐	☐
ER1_05. I have difficulty making sense out of my feelings.	☐	☐	☐	☐	☐	☐	☐
ER1_06. I am attentive to my feelings.	☐	☐	☐	☐	☐	☐	☐
ER1_07. I know exactly how I am feeling.	☐	☐	☐	☐	☐	☐	☐
ER1_08. I care about what I am feeling.	☐	☐	☐	☐	☐	☐	☐
ER1_09. I am confused about how I feel.	☐	☐	☐	☐	☐	☐	☐
ER1_10. When I'm upset, I acknowledge my emotions.	☐	☐	☐	☐	☐	☐	☐
ER1_11. When I'm upset, I become angry with myself for feeling that way.	☐	☐	☐	☐	☐	☐	☐
ER1_12. When I'm upset, I become embarrassed for feeling that way.	☐	☐	☐	☐	☐	☐	☐

	Almost Never (0-10%)	Sometimes (11-35%)	About Half The Time (36-65%)	Most of the Time (66-90%)	Almost Always (91-100%)	I Do Not Know	I Prefer Not to Answer
ER1_13. When I'm upset, I have difficulty getting work done.	☐	☐	☐	☐	☐	☐	☐
ER1_14. When I'm upset, I become out of control.	☐	☐	☐	☐	☐	☐	☐
ER1_15. When I'm upset, I believe that I will remain that way for a long time.	☐	☐	☐	☐	☐	☐	☐
ER1_16. When I'm upset, I believe that I will end up feeling very depressed.	☐	☐	☐	☐	☐	☐	☐
ER1_17. When I'm upset, I believe that my feelings are valid and important.	☐	☐	☐	☐	☐	☐	☐
ER1_18. When I'm upset, I have difficulty focusing on other things.	☐	☐	☐	☐	☐	☐	☐
ER1_19. When I'm upset, I feel out of control.	☐	☐	☐	☐	☐	☐	☐
ER1_20. When I'm upset, I can still get things done.	☐	☐	☐	☐	☐	☐	☐
ER1_21. When I'm upset, I feel ashamed at myself for feeling that way.	☐	☐	☐	☐	☐	☐	☐
ER1_22. When I'm upset, I know that I can find a way to eventually feel better.	☐	☐	☐	☐	☐	☐	☐

	Almost Never (0-10%)	Sometimes (11-35%)	About Half The Time (36-65%)	Most of the Time (66-90%)	Almost Always (91-100%)	I Do Not Know	I Prefer Not to Answer
ER1_23. When I'm upset, I feel like I am weak.	☐	☐	☐	☐	☐	☐	☐
ER1_24. When I'm upset, I feel like I can remain in control of my behaviours.	☐	☐	☐	☐	☐	☐	☐
ER1_25. When I'm upset, I feel guilty for feeling that way.	☐	☐	☐	☐	☐	☐	☐
ER1_26. When I'm upset, I have difficulty concentrating.	☐	☐	☐	☐	☐	☐	☐
ER1_27. When I'm upset, I have difficulty controlling my behaviours.	☐	☐	☐	☐	☐	☐	☐
ER1_28. When I'm upset, I believe there is nothing I can do to make myself feel better.	☐	☐	☐	☐	☐	☐	☐
ER1_29. When I'm upset, I become irritated at myself for feeling that way.	☐	☐	☐	☐	☐	☐	☐
ER1_30. When I'm upset, I start to feel very bad about myself.	☐	☐	☐	☐	☐	☐	☐
ER1_31. When I'm upset, I believe that wallowing in it is all I can do.	☐	☐	☐	☐	☐	☐	☐
ER1_32. When I'm upset, I lose control over my behaviour.	☐	☐	☐	☐	☐	☐	☐

	Almost Never (0-10%)	Sometimes (11-35%)	About Half The Time (36-65%)	Most of the Time (66-90%)	Almost Always (91-100%)	I Do Not Know	I Prefer Not to Answer
ER1_33. When I'm upset, I have difficulty thinking about anything else.	☐	☐	☐	☐	☐	☐	☐
ER1_34. When I'm upset I take time to figure out what I'm really feeling.	☐	☐	☐	☐	☐	☐	☐
ER1_35. When I'm upset, it takes me a long time to feel better.	☐	☐	☐	☐	☐	☐	☐
ER1_36. When I'm upset, my emotions feel overwhelming.	☐	☐	☐	☐	☐	☐	☐

Parent's Education

CS1_01. Please check the box beside the highest grade or degree that your BIOLOGICAL MOTHER completed.

Never went to school	☐
Grades 1 to 3	☐
Grades 4 to 8	☐
Grades 9 to 11	☐
Grade 12	☐
GED	☐
One or more years of Vocational or Professional School after High School	☐
One or more years of College	☐
One or more years of Graduate or Professional School after College	☐
I Do Not Know	☐
I prefer not to answer	☐

CS1_02. Please check the box beside the highest grade or degree that your BIOLOGICAL FATHER completed.

Never went to school	☐
Grades 1 to 3	☐
Grades 4 to 8	☐
Grades 9 to 11	☐
Grade 12	☐
GED	☐
One or more years of Vocational or Professional School after High School	☐
One or more years of College	☐
One or more years of Graduate or Professional School after College	☐
I Do Not Know	☐
I prefer not to answer	☐

Now please think of the two most important adults in your home between the time you were born and age 18 years. Please check the category below that best described their lecel of education during this time period.

CS1_03. First adult's highest level of education:

Never went to school	☐
Grades 1 to 3	☐
Grades 4 to 8	☐
Grades 9 to 11	☐
Grade 12	☐
GED	☐
One or more years of Vocational or Professional School after High School	☐
One or more years of College	☐
One or more years of Graduate or Professional School after College	☐
I Do Not Know	☐
I prefer not to answer	☐

CS1_04. Second adult's highest level of education

Never went to school	☐
Grades 1 to 3	☐
Grades 4 to 8	☐
Grades 9 to 11	☐
Grade 12	☐
GED	☐
One or more years of Vocational or Professional School after High School	☐
One or more years of College	☐
One or more years of Graduate or Professional School after College	☐
I Do Not Know	☐
I prefer not to answer	☐

Your Childhood Experiences

The following questions ask about some difficult experiences that you might have had as a child. These questions may be emotionally difficult to answer. Just as a reminder, you do not need answer any questions that you would prefer not to. Your answers to these questions, as with all questions, will remain confidential.

CE1_01. Before you were 18 years old, did a parent or other adult in the household often or very often…

Swear at you, insult you, put you down, or humiliate you?
or
Act in a way that made you afraid that you might be physically hurt?

□ No
□ Yes
□ I do not know
□ I prefer not to answer

CE1_02. Before you were 18 years old, did a parent or other adult in the household often or very often…

Push, grab, slap, or throw something at you?
or
Ever hit you so hard that you had marks or were injured?

□ No
□ Yes
□ I do not know
□ I prefer not to answer

CE1_03. Before you were 18 years old, did an adult or person at least 5 years older than you ever…

Touch or fondle you or have you touch their body in a sexual way?
or
Attempt or actually have oral, anal, or vaginal intercourse with you?

□ No
□ Yes
□ I do not know
□ I prefer not to answer

CE1_04. Before you were 18 years old, did you often or very often feel that …

No one in your family loved you or thought you were important or special?
or
Your family didn't look out for each other, feel close to each other, or support each other?

□ No
□ Yes
□ I do not know
□ I prefer not to answer

CE1_05. Before you were 18 years old, did you often or very often feel that …

You didn't have enough to eat, had to wear dirty clothes, and had no one to protect you?
or
Your parents were too drunk or high to take care of you or take you to the doctor if you needed it?

□ No
□ Yes
□ I do not know
□ I prefer not to answer

CE1_06. Before you were 18 years old, was a biological parent ever lost to you through divorce, abandonment, or other reason?

□ No
□ Yes
□ I do not know
□ I prefer not to answer

CE1_07. Before you were 18 years old, was your mother or stepmother:

Often or very often pushed, grabbed, slapped, or had something thrown at her?
or
Sometimes, often, or very often kicked, bitten, hit with a fist, or hit with something hard?
or
Ever repeatedly hit over at least a few minutes or threatened with a gun or knife?

□ No
□ Yes
□ I do not know
□ I prefer not to answer

CE1_08. Before you were 18 years old, did you live with anyone who was a problem drinker or alcoholic, or who used street drugs?

- □ No
- □ Yes
- □ I do not know
- □ I prefer not to answer

CE1_09. Before you were 18 years old, was a household member depressed or mentally ill, or did a household member attempt suicide?

- □ No
- □ Yes
- □ I do not know
- □ I prefer not to answer

CE1_10. Before you were 18 years old, did a household member go to prison?

- □ No
- □ Yes
- □ I do not know
- □ I prefer not to answer

Relationships

SN1_01. How many close friends do you have? By close friends, we mean people that you feel at ease with, and that you can talk to about private matters.

□ None → ***skip to Y2***

____ Number of close friends

□ I do not know
□ I prefer not to answer

Y1a. How many of these close friends do you see at least once a month?

____ Number of close friends ***(if none, write zero)***

□ I do not know
□ I prefer not to answer

SN1_02. Thinking about your relatives, how many relatives do you feel at ease with, and feel that you can talk to about private matters?

□ None → ***skip to Y3***

____ Number of relatives

□ I do not know
□ I prefer not to answer

Y2a. How many of these relatives do you see at least once a month?

____ Number of relatives ***(if none, write zero)***

□ I do not know
□ I prefer not to answer

SN1_03. About how often do you participate in groups or clubs, such as religious connected groups, self-help groups, charities, or a public service or community group.

- ☐ Never or almost never
- ☐ A few times a year
- ☐ Once or twice a month
- ☐ Once a week
- ☐ More than once a week
- ☐ I do not know
- ☐ I prefer not to answer

SN1_04. About how often do you go to religious meetings or services?

- ☐ Never or almost never
- ☐ A few times a year
- ☐ Once or twice a month
- ☐ Once a week
- ☐ More than once a week
- ☐ I do not know
- ☐ I prefer not to answer

INSTRUCTIONS: Indicate how often each of the statements below is descriptive of you.

**Statement**	Never	Rarely	Sometimes	Often	I Do Not Know	Prefer not to answer
LS1_01. I feel in tune with the people around me	☐	☐	☐	☐	☐	s
LS1_02. I lack companionship	☐	☐	☐	☐	☐	☐
LS1_03. There is no one I can turn to	☐	☐	☐	☐	☐	☐
LS1_04. I do not feel alone	☐	☐	☐	☐	☐	☐
LS1_05. I feel part of a group of friends	☐	☐	☐	☐	☐	☐
LS1_06. I have a lot in common with the people around me	☐	☐	☐	☐	☐	☐
LS1_07. I am no longer close to anyone	☐	☐	☐	☐	☐	☐
LS1_08. My interests and ideas are not shared by those around me	☐	☐	☐	☐	☐	☐
LS1_09. I am an outgoing person	☐	☐	☐	☐	☐	☐
LS1_10. There are people I feel close to	☐	☐	☐	☐	☐	☐
LS1_11. I feel left out	☐	☐	☐	☐	☐	☐
LS1_12. My social relationships are superficial	☐	☐	☐	☐	☐	☐
LS1_13. No one really knows me well	☐	☐	☐	☐	☐	☐
LS1_14. I feel isolated from others	☐	☐	☐	☐	☐	☐
LS1_15. I can find companionship when I want it	☐	☐	☐	☐	☐	☐
LS1_16. There are people who really understand me	☐	☐	☐	☐	☐	☐
LS1_17. I am unhappy being so withdrawn	☐	☐	☐	☐	☐	☐
LS1_18. People are around me but not with me	☐	☐	☐	☐	☐	☐
LS1_19. There are people I can talk to	☐	☐	☐	☐	☐	☐
LS1_20. There are people I can turn to	☐	☐	☐	☐	☐	☐

Your Sleep

The following questions relate to your usual sleep habits during the past month only. Your answers should indicate the most accurate reply for the majority of days and nights in the past month. ***Please answer all questions.***

SL1_01. **During the past month,** what time have you usually gone to bed at night?

BED TIME ___________ AM / PM

□ I do not know
□ I prefer not to answer

SL1_02. **During the past month**, how long (in minutes) has it usually taken you to fall asleep each night?

NUMBER OF MINUTES ___________

□ I do not know
□ I prefer not to answer

SL1_03. During the past month, at what time have you usually gotten up in the morning?

GETTING UP TIME ___________ AM / PM

□ I do not know
□ I prefer not to answer

SL1_04. During the past month, how many hours of actual sleep did you get on average at night? (This may be different than the number of hours you spent in bed.)

AVERAGE HOURS OF SL1_EEP PER NIGHT ___________

□ I do not know
□ I prefer not to answer

For each of the remaining questions, check the one best response. Please answer all questions.

During the past month, how often have you had trouble sleeping because you…………..

SL1_05. …..Could not get to sleep within 30 minutes?

□ Not during the past month
□ Less than once a week
□ Once or twice a week
□ Three or more times a week
□ I do not know
□ I prefer not to answer

SL1_06. …..Woke up in the middle of the night or early morning?

□ Not during the past month
□ Less than once a week
□ Once or twice a week
□ Three or more times a week
□ I do not know
□ I prefer not to answer

SL1_07. …..Had to get up to use the bathroom?

□ Not during the past month
□ Less than once a week
□ Once or twice a week
□ Three or more times a week
□ I do not know
□ I prefer not to answer

SL1_08. …..Could not breathe comfortably?

☐ Not during the past month
☐ Less than once a week
☐ Once or twice a week
☐ Three or more times a week
☐ I do not know
☐ I prefer not to answer

SL1_09. …..Coughed or snored loudly?

☐ Not during the past month
☐ Less than once a week
☐ Once or twice a week
☐ Three or more times a week
☐ I do not know
☐ I prefer not to answer

SL1_10. …..Felt too cold?

☐ Not during the past month
☐ Less than once a week
☐ Once or twice a week
☐ Three or more times a week
☐ I do not know
☐ I prefer not to answer

SL1_11. …..Felt too hot?

☐ Not during the past month
☐ Less than once a week
☐ Once or twice a week
☐ Three or more times a week
☐ I do not know
☐ I prefer not to answer

SL1_12. …..Had bad dreams?

□ Not during the past month
□ Less than once a week
□ Once or twice a week
□ Three or more times a week
□ I do not know
□ I prefer not to answer

SL1_13. …..Had pain?

□ Not during the past month
□ Less than once a week
□ Once or twice a week
□ Three or more times a week
□ I do not know
□ I prefer not to answer

SL1_14a. Other reason(s), **please describe:** ______________________________________

SL1_14b. How often during the past month have you had trouble sleeping because of this?

□ Not during the past month
□ Less than once a week
□ Once or twice a week
□ Three or more times a week
□ I do not know
□ I prefer not to answer

SL1_15. **During the past month**, how would you rate your sleep quality overall?

□ Very good
□ Fairly good
□ Fairly bad
□ Very bad
□ I do not know
□ I prefer not to answer

SL1_16. **During the past month**, how often have you taken medicine to help you sleep (prescribed or "over the counter")?

- □ Not during the past month
- □ Less than once a week
- □ Once or twice a week
- □ Three or more times a week
- □ I do not know
- □ I prefer not to answer

SL1_17. **During the past month**, how often have you had trouble staying awake while driving, eating meals, or engaging in social activity?

- □ Not during the past month
- □ Less than once a week
- □ Once or twice a week
- □ Three or more times a week
- □ I do not know
- □ I prefer not to answer

SL1_18. **During the past month**, how much of a problem has it been for you to keep up enough enthusiasm to get things done?

- □ No problem at all
- □ Somewhat of a problem
- □ A very big problem
- □ I do not know
- □ I prefer not to answer

SL1_19. Do you have a bed partner or a room-mate in the same room ?

- □ No bed partner or roommate in the same room → ***skip to next survey section:***
- □ Partner in same bed
- □ Room-mate in the same room
- □ I do not know
- □ I prefer not to answer

If you have a roommate or bed partner, ask him/her how often in the past month you have had . . . .

SL1_20. …..Loud snoring

☐ Not during the past month
☐ Less than once a week
☐ Once or twice a week
☐ Three or more times a week
☐ Unable to ask him/her
☐ he/she does not know
☐ he/she prefers not to answer

SL1_21. ……Long pauses between breaths while asleep

☐ Not during the past month
☐ Less than once a week
☐ Once or twice a week
☐ Three or more times a week
☐ Unable to ask him/her
☐ he/she does not know
☐ he/she prefers not to answer

SL1_22. …..Legs twitching or jerking while you sleep

☐ Not during the past month
☐ Less than once a week
☐ Once or twice a week
☐ Three or more times a week
☐ Unable to ask him/her
☐ he/she does not know
☐ he/she prefers not to answer

SL1_23. …..Episodes of disorientation or confusion during sleep

□ Not during the past month
□ Less than once a week
□ Once or twice a week
□ Three or more times a week
□ Unable to ask him/her
□ he/she does not know
□ he/she prefers not to answer

SL1_24a. ……Other restlessness while you sleep; ***please describe:***

____

SL1_24b. □ Not during the past month
□ Less than once a week
□ Once or twice a week
□ Three or more times a week
□ Unable to ask him/her
□ he/she does not know
□ he/she prefers not to answer

Below you will find a list of statements. Please indicate how often each statement applies to you generally in daily life.

	0- Never	1	2	3	4	5 - Always
IA1_01. When I am tense I notice where the tension is located in my body.	☐	☐	☐	☐	☐	☐
IA1_02. I notice when I am uncomfortable in my body.	☐	☐	☐	☐	☐	☐
IA1_03. I notice where in my body I am comfortable.	☐	☐	☐	☐	☐	☐
IA1_04. I notice changes in my breathing, such as whether it slows down or speeds up.	☐	☐	☐	☐	☐	☐
IA1_05. I do not notice (I ignore) physical tension or discomfort<br>until they become more severe.	☐	☐	☐	☐	☐	☐
IA1_06. I distract myself from sensations of discomfort.	☐	☐	☐	☐	☐	☐
IA1_07. When I feel pain or discomfort, I try to power through it.	☐	☐	☐	☐	☐	☐
IA1_08. When I feel physical pain, I become upset.	☐	☐	☐	☐	☐	☐
IA1_09. I start to worry that something is wrong if I feel any discomfort.	☐	☐	☐	☐	☐	☐
IA1_10. I can notice an unpleasant body sensation without worrying about it.	☐	☐	☐	☐	☐	☐
IA1_11. I can pay attention to my breath without being distracted by things happening around me.	☐	☐	☐	☐	☐	☐
IA1_12. I can maintain awareness of my inner bodily sensations even when there is a lot going on around me.	☐	☐	☐	☐	☐	☐
IA1_13. When I am in conversation with someone, I can pay attention to my posture.	☐	☐	☐	☐	☐	☐
IA1_14. I can return awareness to my body if I am distracted.	☐	☐	☐	☐	☐	☐
IA1_15. I can refocus my attention from thinking to sensing my body.	☐	☐	☐	☐	☐	☐
IA1_16. I can maintain awareness of my whole body even when a part of me is in pain or discomfort.	☐	☐	☐	☐	☐	☐
IA1_17. I am able to consciously focus on my body as a whole.	☐	☐	☐	☐	☐	☐
IA1_18. I notice how my body changes when I am angry.	☐	☐	☐	☐	☐	☐
IA1_19. When something is wrong in my life I can feel it in my body.	☐	☐	☐	☐	☐	☐
IA1_20. I notice that my body feels different after a peaceful experience.	☐	☐	☐	☐	☐	☐
IA1_21. I notice that my breathing becomes free and easy when I feel comfortable.	☐	☐	☐	☐	☐	☐
IA1_22. I notice how my body changes when I feel happy / joyful.	☐	☐	☐	☐	☐	☐

	0- Never	1	2	3	4	5 - Always
IA1_23. When I feel overwhelmed I can find a calm place inside.	☐	☐	☐	☐	☐	☐
IA1_24. When I bring awareness to my body I feel a sense of calm.	☐	☐	☐	☐	☐	☐
IA1_25. I can use my breath to reduce tension.	☐	☐	☐	☐	☐	☐
IA1_26. When I am caught up in thoughts, I can calm my mind by focusing on my body/breathing.	☐	☐	☐	☐	☐	☐
IA1_27. I listen for information from my body about my emotional state.	☐	☐	☐	☐	☐	☐
IA1_28. When I am upset, I take time to explore how my body feels.	☐	☐	☐	☐	☐	☐
IA1_29. I listen to my body to inform me about what to do.	☐	☐	☐	☐	☐	☐
IA1_30. I am at home in my body.	☐	☐	☐	☐	☐	☐
IA1_31. I feel my body is a safe place.	☐	☐	☐	☐	☐	☐
IA1_32. I trust my body sensations.	☐	☐	☐	☐	☐	☐

We are interested in your recent experiences. Below is a list of things that people sometimes experience. Next to each item are five choices: “never”, “rarely”, “sometimes”, “often”, and “all the time”. Please darken one of these to indicate how much you currently have experiences similar to those described.

Please do not spend too long on each item–it is your first response that we are interested in. Please be sure to answer every item.

	Never	Rarely	Sometimes	Often	All the time
RD1_01. I think about what will happen in the future.	☐	☐	☐	☐	☐
RD1_02. I remind myself that thoughts aren’t facts.	☐	☐	☐	☐	☐
RD1_03. I am better able to accept myself as I am.	☐	☐	☐	☐	☐
RD1_04. I notice all sorts of little things and details in the world around me.	☐	☐	☐	☐	☐
RD1_05. I am kinder to myself when things go wrong.	☐	☐	☐	☐	☐
RD1_06. I can slow my thinking at times of stress.	☐	☐	☐	☐	☐
RD1_07. I wonder what kind of person I really am.	☐	☐	☐	☐	☐
RD1_08. I am not so easily carried away by my thoughts and feelings.	☐	☐	☐	☐	☐
RD1_09. I notice that I don’t take difficulties so personally.	☐	☐	☐	☐	☐
RD1_10. I can separate myself from my thoughts and feelings.	☐	☐	☐	☐	☐
RD1_11. I analyze why things turn out the way they do.	☐	☐	☐	☐	☐
RD1_12. I can take time to respond to difficulties.	☐	☐	☐	☐	☐
RD1_13. I think over and over again about what others have said to me.	☐	☐	☐	☐	☐
RD1_14. I can treat myself kindly.	☐	☐	☐	☐	☐
RD1_15. I can observe unpleasant feelings without being drawn into them.	☐	☐	☐	☐	☐
RD1_16. I have the sense that I am fully aware of what is going on around me and inside me.	☐	☐	☐	☐	☐
RD1_17. I can actually see that I am not my thoughts.	☐	☐	☐	☐	☐
RD1_18. I am consciously aware of a sense of my body as a whole.	☐	☐	☐	☐	☐
RD1_19. I think about the ways in which I am different from other people.	☐	☐	☐	☐	☐
RD1_20. I view things from a wider perspective.	☐	☐	☐	☐	☐

How do you cope with events?

Everyone gets confronted with negative or unpleasant events now and then and everyone responds to them in his or her own way. By the following questions you are asked to indicate what you generally think, when you experience negative or unpleasant events.

	(Almost) Never	Some-times	Regularly	Often	(Almost) Always
CR1_01. I think that I have to accept that this has happened.	☐	☐	☐	☐	☐
CR1_02. I often think about how I feel about what I have experienced.	☐	☐	☐	☐	☐
CR1_03. I think I can learn something from the situation.	☐	☐	☐	☐	☐
CR1_04. I feel that I am the one who is responsible for what has happened.	☐	☐	☐	☐	☐
CR1_05. I think that I have to accept the situation.	☐	☐	☐	☐	☐
CR1_06. I am preoccupied with what I think and feel about what I have experienced.	☐	☐	☐	☐	☐
CR1_07. I think of pleasant things that have nothing to do with it.	☐	☐	☐	☐	☐
CR1_08. I think that I can become a stronger person as a result of what has happened.	☐	☐	☐	☐	☐
CR1_09. I keep thinking about how terrible it is what I have experienced.	☐	☐	☐	☐	☐
CR1_10. I feel that others are responsible for what has happened.	☐	☐	☐	☐	☐
CR1_11. I think of something nice instead of what has happened.	☐	☐	☐	☐	☐
CR1_12. I think about how to change the situation.	☐	☐	☐	☐	☐
CR1_13. I think that it hasn't been too bad compared to other things.	☐	☐	☐	☐	☐
CR1_14. I think that basically the cause must lie within myself.	☐	☐	☐	☐	☐
CR1_15. I think about a plan of what I can do best.	☐	☐	☐	☐	☐
CR1_16. I tell myself that there are worse things in life.	☐	☐	☐	☐	☐
CR1_17. I continually think how horrible the situation has been.	☐	☐	☐	☐	☐
CR1_18. I feel that basically the cause lies with others.	☐	☐	☐	☐	☐

Thank you for completing this survey!

Please note that these responses will not be seen immediately. Resources are shown below if you feel that you would like to talk with someone immediately for assistance.

National Suicide Prevention Lifeline: 1-800-273-8255
National Sexual Assault Hotline: 1-800-656-4673

Other options are to:
- Call your doctor's office
- Call 911 for emergency services
- Go to the nearest hospital emergency room.

APPENDIX 4

INFORMED CONSENT FORM

Agreement to Participate in a Research Study

Investigation of the Effects of Mindfulness on Blood Pressure and Well-Being

You are being asked to take part in a Brown University research study about the effects of mindfulness and hypertension education on blood pressure and risk factors for hypertension. This form will explain the purpose of the study, how the study will be carried out and what you will be expected to do. It will also explain the possible risks and possible benefits of being in the study. If any part of the following description is not clear to you, you are encouraged to contact the researcher to answer any questions before you decide whether to take part in the study. If you decide to participate, please fill out and sign the last page of this form.

1a. Nature and Purpose of the Study

The purpose of the study is to investigate the impact of mindfulness practices and health education on blood pressure. You have been selected for this study because you expressed interest and met entrance criteria for having prehypertension or hypertension, or another cardiovascular risk factor that could be influenced by this program. In order to assess the effects of these practices, you will be asked to complete some questionnaires, and a laboratory assessment before and after learning the mindfulness practices.

1b. Explanation of Procedures

If you agree to participate, you will be asked to consent to the following:

1) Participate in an interview in which you will be asked questions about past and present mental health, including depression and suicide.

2) Complete questionnaires about a wide range of topics, including your diet, physical activity, smoking, medication use, personality, emotions, attention and past experiences, including stressful or traumatic experiences. These questions will probe sensitive psychological areas, including physical, emotion and sexual abuse. These questionnaires may take up to 2 hours to complete. By completing the interviews and questionnaires, you are giving the researchers permission to use the information you have provided. You have the right not to answer any of the questions.

3) Directly assessed blood pressure, height, weight, waist circumference and hip circumference.

4) You will be asked to perform some cognitive tasks. One of these tasks may involve computer-based tests of attention or decision-making. Together these tests may take as long as 45 minutes.

5) You will participate in the mindfulness program, which consists of 9 weekly sessions of 2.5 hours each and will include one 8 hour weekend retreat. Daily homework assignments may take as long as one hour and consist of practicing mindfulness exercises with the aid of a guided audio CD and completing worksheets related to stress, thoughts, and common reactions to various types of events.

Eric Loucks 6/2/2015 5:51 PM
Comment [1]: This random assignment text was deleted, as we are not currently using a control group. The control group will be added in future research after the initial pilot testing phase.

Eric Loucks 6/17/2015 11:58 AM
Formatted: Underline, Highlight

Eric Loucks 6/17/2015 11:58 AM
Formatted: Underline

Eric Loucks 6/2/2015 5:48 PM
Deleted: ... [1]

Eric Loucks 6/17/2015 11:08 AM
Deleted: the 9-week meditation

6) Class sessions will be audio taped so we can analyze the quality of the treatment you receive. The recordings will be transcribed so that we may analyze the text. The recordings will be identified by study number, will only be heard by study staff and will be destroyed after transcription.

Eric Loucks 6/17/2015 11:56 AM
Formatted: Underline, Highlight

7) You will be asked to complete a few short questionnaires each week during the 9 week condition.

8) After 9 weeks, you will be asked to complete questionnaires and return to the laboratory to repeat the same procedures for a second day of testing.

9) Three months after the end of the 9 week condition, you will be asked to return to the laboratory to repeat the same testing procedures.

Table Summarizing Activities and Time Commitment for this Study.

**Activity**	**Time Commitment**
First blood pressure screen	30 minutes
Second blood pressure screen	30 minutes
Baseline health assessments, such as questionnaires, height, weight and waist circumference.	2 hours
Mindfulness course	9 sessions that are 2.5 hours each.<br>1 retreat day on a Saturday that will be 8 hours<br>Total course time: 30.5 hours
Home mindfulness practices assigned during course.	1 hour per day, 6 days per week, for 8 weeks.<br>Total time: 48 hours.
Health assessments, such as questionnaires, blood pressure, height, weight and waist circumference, that take place immediately after course completion.	2 hours
Health assessments, such as questionnaires, blood pressure, height, weight and waist circumference, that take place that take place 3 months after course completion.	2 hours
TOTAL TIME COMMITMENT FOR STUDY	85.5 hours

Eric Loucks 6/17/2015 11:57 AM
Formatted: Highlight

Feedback:
At the end of the study, you will receive individual feedback about the changes that occurred since the first assessment. Specifically, you will receive an individualized handout listing % change (increase or decrease) on scales of attention, stress, mood, health behaviors, weight, and blood pressure across the study.

2. <u>Discomforts and Risks</u>
The questionnaires are routine, standardized forms for epidemiologic research. Certain questions may be upsetting as they may probe sensitive psychological areas and inquire about upsetting or traumatic events, including physical, sexual or emotional abuse and/or current psychiatric symptoms. Since your participation is voluntary, you have the right to skip any questions that make you uncomfortable.

Meditation-based interventions may results in discomfort with attention to unpleasant thoughts, feelings or body sensations. Some individuals may experience an initial increase in undesirable feelings with increased attention to them.

3. <u>Benefits</u>
We cannot and do not guarantee or promise that you will receive any direct benefits from this study. However, participation in the study creates the potential benefit of a) identifying effective treatments for elevated blood pressure, b) gaining knowledge of the effects of mindfulness practices, c) receiving information about your psychological and physical functioning.

4. <u>Alternative Therapies</u>
A number of different therapies, including antihypertensive medication, diet changes, physical activity, and reducing excessive alcohol consumption may also be beneficial for reducing blood pressure. Education about these therapies are integrated into this course, but other forms of these alternative therapies are also available in the community.

5. <u>Confidentiality</u>
Your responses for this study will be kept confidential. All data that we collect will be linked to a study ID# instead of your name. All questionnaires in this study will be filled out through an online survey or a paper version of the survey if you prefer. All of these questionnaires will be linked solely to your study ID#, so that your identity is protected and your answers are confidential. Although these measures have been taken to protect your personal information, complete confidentiality cannot be guaranteed when transmitting information over the internet.

Eric Loucks 6/2/2015 5:47 PM
Deleted: website (SurveyMonkey)

While your confidentiality is protected to the extent of the law, there are limitations to confidentiality. If your questionnaire responses indicate that you pose a serious danger to yourself or to another person, then a collaborator (Dr. Ellen Flynn) who is a licensed psychiatrist, may contact you to discuss your responses and possible referral to a treatment provider. Questionnaire items that may warrant follow-up include endorsements of statements about hurting yourself, any high scores in depression, anxiety, or other clinically significant problems. You should also know that there are times when the law might require the release of your responses without your permission. For example, State law requires researchers to report abuse or neglect of children to the Department of Children, Youth and Families (DCYF). State law also requires researchers to report abuse or neglect of people age 60 and older to the Division of Elderly Affairs.

The findings of the study may be used for medical publication. Your name will not be used in any published reports about this study. Results will be reported in a summarized manner in such a way that you cannot be identified. All personally identifiable information will be "de-identified" and only a unique code number will be used. Study records will be identified with a

unique code number and initials. All study records and specimens will be stored in a secure storage area.

Keeping study records: The Principal Investigator for this study will keep your research records indefinitely for research purposes.

6. Refusal/Withdrawal
Participation is voluntary, and you may decide to opt out or refuse any part of participation, including not answering certain questions. If you decide now to participate, you can change your mind later and quit the study. The decision to not participate or to withdraw from the study will not adversely affect current or future interactions with Brown University. The decision to not participate or to withdraw from the study will also not adversely affect your relationship with your physician.

If you decide not to participate, or if you quit the study, we will provide you with referrals for alternative treatments, if desired.

7. Contact Information
If you have questions about study procedures at any time you may contact the researchers: Dr. Eric B. Loucks, email: eric.loucks@brown.edu, telephone (401) 863-6283. If you would like more information about the rules for research studies, or the rights of people who take part in those studies, you may contact the Brown University Research Protections Office, telephone number 1-866-309-2095 or 401-863-3050.

CONSENT FORM:
Please sign and return.

Please read the following agreement and sign to consent to participating.

I HAVE READ THE ABOVE DESCRIPTION OF THIS STUDY. ALL OF MY QUESTIONS HAVE BEEN SATISFACTORILY ANSWERED, AND, I AGREE TO PARTICIPATE IN THIS RESEARCH STUDY.

____
PRINT NAME

____________________________________ __________________
Signature of participant **Date**

CONTACT INFORMATION

Name (print):____

Permanent Address:____

Email(s):____

Telephone:_________________________**(cell)** _________________________**(other)**

Page 1: [1] Deleted	Eric Loucks	6/2/15 5:48 PM

You may be randomly assigned (like the flip of a coin) to enter one of two different 8-week meditation programs.

APPENDIX 5

PROTOCOL FOR PHYSICIANS TO REFER PATIENTS TO MINDFULNESS-BASED HYPERTENSION THERAPY STUDY

Protocol for Physicians to Refer Patients to Mindfulness-Based Hypertension Therapy Study

Protocol

Option 1:

1. Physicians will inform their patients about the study, and then ask if they would be interested in being contacted by the research study staff to explore if they would like to be in the study.
2. If the patient is interested, the physician will ask if the patient would be willing to have their contact information shared with the researchers, including name, phone number and email address.
3. If the patient agrees, the physician will forward this information to Dr. Loucks.

Option 2:

A second option is for physicians to provide an advertisement card (supplied by the research study staff) to their patients and invite them to contact the research study staff if they are interested in it. This may result in lower participant recruitment rates, but is a very acceptable method if the physician prefers this approach.

Ways that physicians can inform study participants about the study

1. Providing information on the study, such as the script below.

   "I wonder if you may be interested in a research study taking place at Brown University, called Mindfulness-Based Hypertension Therapy. The intervention includes teachings on mindfulness meditation, mindful movements, education about hypertension, and support for reducing hypertension risk factors. The study is testing whether this intervention lowers blood pressure. The program consists of nine weekly sessions that are 2.5 hours long, and a one-day retreat on a weekend that will be 8 hours long. People who have a current regular meditation practice (i.e. meditate more than once per week) are not eligible. Research participation includes interviews, questionnaires and measurements of blood pressure, height, weight, and waist circumference, before and after the meditation program."

2. Providing a handout, such as the advertisement card previously approved by the IRB and distributed to physicians interested in the study for their patients.

Brown University

Research Protections Office
Institutional Review Board
Amendment Request

Date of Request: 7/15/15 **Investigator's Name and Title:** Eric Loucks, Assistant Professor

Study Title: Mindfulness-Based Hypertension Therapy Pilot Study (#1412001171)

Original Type of Review: ☐ Exempt ☐ Expedited ☒ Full Board

1) **Provide a brief lay summary of the overall project. Include enough detail to allow the IRB to evaluate the requested change(s) within the context of the overall project.** (Attach summary to this form) Please see attached.

2) **Provide a detailed description of the changes being requested** (Use additional pages, if necessary):
Please see attached.

3.) **State the reason (justification) for the requested amendment.** (Use additional pages, if necessary):
Please see attached.

4.) **What is your assessment of how the changes will affect the overall risk/benefit ratio of the study and the willingness of individuals to participate?**
Please see attached.

5.) **Does the requested amendment require new documents or changes to the approved consent form or other documents?**

- ☒ Consent/assent documents (attach revised version with changes highlighted)
- ☒ New/revised instruments (attach - if revised, highlight changes)
- ☐ New/revised advertising materials (attach - if revised, highlight changes)

Do you have a conflict of interest on this project according to Brown's policy? ☐ **YES** ☒ **NO**

If YES, has this conflict been previously disclosed to the IRB? ☐ **YES** ☐ **NO**

PI signature: ______________________ Date: 7/15/15

1) **Provide a brief lay summary of the overall project. Include enough detail to allow the IRB to evaluate the requested change(s) within the context of the overall project.**

The World Health Organization reported that suboptimal blood pressure (BP) is responsible for more than half of cardiovascular disease mortality world-wide. Furthermore, greater than half of those with hypertension have uncontrolled BP. *A 2009 Institute of Medicine report recommended prioritizing research to "Compare the effectiveness of mindfulness-based interventions (e.g. yoga, meditation, deep breathing training) and usual care in treating… cardiovascular risk factors."* Evidence-based mindfulness interventions, including Mindfulness-Based Stress Reduction, may have some effects on blood pressure, where a recent meta-analysis and systematic review of 4 randomized controlled trials demonstrated significant effects, but evidence of heterogeneity in effect sizes. The methodologically highest quality studies had the smallest effect sizes (range 0-5 mmHg). Mindfulness-Based Stress Reduction (MBSR) has been customized to a number of disease processes, such as Mindfulness-Based Cognitive Therapy for patients with recurrent depression, and Mindfulness-Based Relapse Prevention for patients with substance use addictions. Effect sizes have been increased by customizing mindfulness interventions to diseases of interest. The same may be true for hypertension, *however mindfulness interventions customized for prehypertensive/hypertensive patients have never been investigated. Until methodologically rigorous studies to evaluate customized interventions for hypertension are performed, we will not know if the observed preliminary effects of general mindfulness interventions on blood pressure reduction could be much more effective with a tailored approach.* Consequently, we propose to conduct a stage I behavioral therapy intervention study to evaluate whether MBSR customized to prehypertensive and hypertensive patients has the potential to provide clinically relevant reductions in BP. Consequently the specific aims are:

Stage 1a: Therapy Development/Manual Writing

1 To outline and evaluate key novel elements of mindfulness-based hypertension therapy (MBHT), customized from the evidence-based MBSR. *We hypothesize that the most important novel element will be generation of mindfulness skills specifically applied to hypertension risk factors such as diet, physical activity, obesity, alcohol consumption and antihypertensive medication adherence.* This aim will be achieved using (1) focus groups of participants undergoing the MBHT behavioral intervention, (2) discussion with experts (including cardiologists, epidemiologists, mindfulness experts, mindfulness intervention instructors) prior to, and following pilot testing of MBHT in participants, and (3) clinical judgment of the investigators performing the intervention.

2 To determine effectiveness of MBHT on primary outcomes (systolic blood pressure, retention rates, recruitment rates, and adverse effects) and secondary outcomes (hypertension risk factors such as diet, physical activity, obesity, and antihypertensive medication adherence) in hypertension subgroups, specifically participants with (1) prehypertension, (2) controlled hypertension, and (3) uncontrolled hypertension. Initial decisions about the targeted sample based on hypertension status will be made.

3 To develop an MBHT therapist manual and training program, including procedures for training, supervising, and evaluating therapists. Furthermore, acceptable therapist characteristics will be developed. The manual and training program will include themes such as specification of unique and common elements of MBHT *vs.* other interventions, description of interventions excluded from MBHT, and specification of key treatment parameters such as frequency and duration of treatment, session length, topics addressed, sequence of sessions, as well as therapist adherence and competency measures. The MBHT training will consist of a therapist manual, a formal didactic training seminar, and at least one closely supervised training session.

Stage Ib: Pilot Trial

4 To determine whether a mindfulness-based hypertension therapy (MBHT) intervention, customized from the evidence-based MBSR, has promise to be an effective behavioral therapy for participants with hypertension and/or prehypertension. We will perform a randomized controlled pilot trial for MBHT *vs.*

enhanced usual care control. *We hypothesize that MBHT will have adequate recruitment rates (≥10% of prehypertensive/hypertensive participants invited from physicians' offices), fairly low drop out rates (<15%), and medium effect sizes (e.g. 5-10 mmHg systolic BP) for reduction in blood pressure.*

These findings will provide publishable pilot data that will inform future randomized clinical trials that evaluate effects of MBHT on long-term changes in blood pressure *vs.* usual care and active control groups. *If proven effective, MBHT could be offered as a complementary program in the prehypertensive/hypertensive patient population that contributes to over half of the cardiovascular disease mortality world-wide.*

2) Provide a detailed description of the changes being requested (Use additional pages, if necessary):

1. NEFS Recruitment Letter
    a. We plan on increasing the participant pool by recruiting participants from the Harvard/Brown University follow-up studies for the National Collaborative Perinatal Project, specifically the New England Family Study (NEFS). We will distribute letters to eligible participants of the NEFS providing a brief overview of the MBHT study and inquire about their interest in participating in the MBHT. Interested participants will then be introduced into the screening process and included in the study, if eligible.
2. In-Person Screening Assessments
    a. Prior IRB applications did not provide clear documentation that there are 2 (not 1) in-person screening assessments. There are 2 screening assessment to allow for blood pressure to be assessed on two different days, at least one week apart from each other. This allows for daily changes in blood pressure (e.g. due to changes in stress levels) to be accounted for. Both in-person screening assessments are now included in Appendix 2.
    b. For the medication questions (pp. 3-17), a question was added asking participants what the medication is used for. This question was added to help identify participants' intended use of medications.
3. Phone Screening Questionnaire
    a. Minor wording changes were made to improve clarity to participants, shown in Appendix 3 using track changes.
    b. We added a question to ask participants time availability for the intervention, shown on pg. 6 of the Phone Screening Questionnaire.
    c. To the Safety Plan, we added an area to write down participant contact info (contact info is already provided by participants earlier in the phone screening questionnaire), so that it can be presented to a staff member if the safety plan is triggered (pg. 7 of the Phone Screening Questionnaire). This information is important to be provided to the 911 operator.
4. Consent Form
    a. We added a footer to the consent form, showing the version number, date, and page numbering (Appendix 4).

3.) State the reason (justification) for the requested amendment:

Please see justification in Section 2 above.

4.) What is your assessment of how the changes will affect the overall risk/benefit ratio of the study and the willingness of individuals to participate?

I do not expect any changes to the risk/benefit ratio or willingness of individuals to participate.

5.) Does the requested amendment require new documents or changes to the approved consent form or other documents?

Yes. Please see Appendices.

APPENDIX 1

New England Family Study Recruitment Letter

[Date]

Dear _____,

You recently participated in one of our Harvard/Brown University follow-up studies of the National Collaborative Perinatal Project. Our group, known as the New England Family Study, is very grateful for your participation and this ongoing work has been important in improving our understanding of long-term health and development

Based on your blood pressure readings from the last study we evaluated your blood pressure in, you may qualify for another New England Family Study research project at Brown University. The study is designed to evaluate effects of an intervention called Mindfulness-Based Hypertension Therapy (MBHT). MBHT combines mindfulness practices such as meditation and mindful movements with education about hypertension management and prevention. The aim of MBHT is to create a skill set and supportive environment to help to reduce blood pressure, and improve overall well-being. Your involvement in this study could help us better understand if MBHT is effective, and how it could be improved. The MBHT intervention will take place over 9 weeks, and would involve you attending a class once per week that would be 2.5 hours long. You would also be asked to participate in a full day mindfulness retreat that would be about 8 hours duration. Also, health assessments would be performed before you start the intervention, and again afterwards. The assessments would include measures such as blood pressure, height, weight, diet, physical activity, smoking, life experiences, stress, and medication use.

At the end of the study, you will receive individual feedback about the changes in your health that occurred since the first assessment.

If you think you may be interested in participating in this study, please give us a call at 401-369-0443 or reply to this letter using the enclosed form and stamped envelope. We can then set up a time to give you a call to discuss the study more, and do a screening assessment to see if you quality for the study. Thank you for participating in the New England Family Study. We are incredibly grateful for your continuing contributions. We look forward to speaking with you.

Sincerely yours,

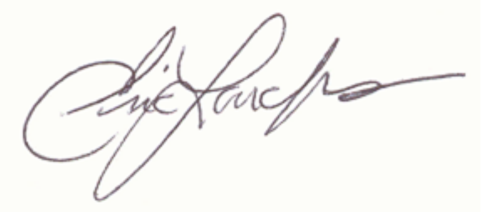

Eric B. Loucks, Ph.D.
Assistant Professor
Department of Epidemiology
Brown University School of Public Health

RESPONSE FORM

We would appreciate knowing when a member of our staff could reach you. It would be helpful if you could complete this form and return it in the enclosed self-addressed, stamped envelope.

1. If you no longer go by the name   , please make any corrections on the line below:
   ____

2. What is the best time of day for someone to contact you?
   ______ mornings
   _____ afternoons
   ______ evenings
3. What is the best phone number for us to reach you during the:
   daytime____________________________________
   evening____________________________________

4. Do you prefer to be contacted in person or by the telephone?
   ____ In person
   ____ Over the phone
   ____ Either
5. Are there certain days of the week that are more convenient for you?
   ____

6. Please mark your level of interest below (optional):
   _____ I might be interested; please contact me
   _____ I might be interested, please send me more information before contacting me
   _____ Do not contact me again at this time; you may contact me in the future to see if I am interested then
   _____ Do not contact me at this time or in the future; I do not want any further contact with this project

You may also reach us at 401-369-0443 to further discuss the study, or your intent to participate. We look forward to speaking with you. Please feel free to call us with any questions. Thank you very much for your assistance.

About the NCPP……………….

The National Collaborative Perinatal Project was conducted from 1959 through 1965 at more than ten sites in the United States. Follow-up continued through the early 1970s. More than 60,000 women and their children participated in this study nationwide, while in the Boston area more than 12,000 families participated. In the Boston area the study was known as the Maternal and Infant Health Study.

The National Collaborative Perinatal Project began when it became clear that events that occur before birth and for the first years of a person's life can have an impact on their later life. Information was taken on the health of the women participating in the study, their family's health, and their living conditions (such as whether or not the household had pets, and who else lived with the mother and child). Once the children were born, they came for regular check-ups to follow their development. The children were followed until they were seven years old.

The National Collaborative Perinatal Project was a great success. Data were collected that helped prove that pre-birth conditions had an influence on the life and development of children. It helped to establish that conditions like low-birth weight and premature birth may increase a child's chances of having health problems, although this is not always the case. It also helped to establish that children who were in play groups or pre-school at an early age often performed very well in school. Support and funding for pre-school programs were increased in part because of the information provided by this study. As a result of this study, programs were funded that helped all women get medical care while they were pregnant and medical care for their infants.

There have been many follow-up studies that have used the data collected by the National Collaborative Perinatal Project. One of these studies looked at whether women whose mothers had a difficult time during labor were more likely to have a difficult time when they themselves gave birth. With information from this study, doctors are now better able to help women who may have difficult pregnancies to take precautions and get extra medical assistance.

Thanks to the women who participated in the National Collaborative Perinatal Project in the 1960s and early 1970s, we now know that what happens to children before they are born and during their first years of life has a great effect on their development. As a result of this study, hospitals have developed better ways to care for all women before they have their babies. In addition, children around the country have greater access to medical care.

In the last few years, many children who were a part of the National Collaborative Perinatal Project participated in an important follow-up study. This study will also help to understand how early life events can affect not only early development, but also development into adulthood. The adults who participated in the follow-up study were asked questions about their education, family and emotional health to determine how people’s lives have changed since childhood. In addition, they were asked to complete numerous tasks in order to understand things such as memory and attention and how these factors relate to development. This current phase of the study is still continuing and the results are proving to be useful, in large part because of the tremendous amount of time so many study children have contributed to the project.

APPENDIX 2A

First In-Person Screening Assessment Form

PID. Participant ID # __________
BA2_01. Staff ID # __________
BA2_02. Today's date (MMDDYY): ___________

Blood Pressure:
BA2_03a. Blood pressure 1st reading, systolic blood pressure: _________ mmHg
BA2_03b. Blood pressure 1st reading, diastolic blood pressure: __________ mmHg
BA2_03c. Blood pressure 2nd reading, systolic blood pressure: _________ mmHg
BA2_03d. Blood pressure 2nd reading, diastolic blood pressure: __________ mmHg
BA2_03e. Blood pressure 3rd reading, systolic blood pressure: _________ mmHg
BA2_03f. Blood pressure 3rd reading, diastolic blood pressure: __________ mmHg
BA2_03g. Blood pressure 4th reading, systolic blood pressure: _________ mmHg
BA2_03h. Blood pressure 4th reading, diastolic blood pressure: __________ mmHg
BA2_03i. Blood pressure 5th reading, systolic blood pressure: _________ mmHg
BA2_03j. Blood pressure 5th reading, diastolic blood pressure: __________ mmHg

BA2_04. Were the 4th and 5th systolic blood pressure readings within 20 mmHg of each other?
□ Yes
□ No ***(repeat measurements***

BA2_05. Were the 4th and 5th diastolic blood pressure readings within 10 mmHg of each other?
□ Yes
□ No ***(repeat measurements)***

BA2_06a. Repeated blood pressure 1st reading, systolic blood pressure: _________ mmHg
BA2_06b. Repeated blood pressure 1st reading, diastolic blood pressure: __________ mmHg
BA2_06c. Repeated blood pressure 2nd reading, systolic blood pressure: _________ mmHg
BA2_06d. Repeated blood pressure 2nd reading, diastolic blood pressure: __________ mmHg
BA2_06e. Repeated blood pressure 3rd reading, systolic blood pressure: _________ mmHg
BA2_06f. Repeated blood pressure 3rd reading, diastolic blood pressure: __________ mmHg
BA2_06g. Repeated blood pressure 4th reading, systolic blood pressure: _________ mmHg
BA2_06h. Repeated blood pressure 4th reading, diastolic blood pressure: __________ mmHg
BA2_06i. Repeated blood pressure 5th reading, systolic blood pressure: _________ mmHg
BA2_06j. Repeated blood pressure 5th reading, diastolic blood pressure: __________ mmHg

BA2_07. Blood pressure cuff size used: □ S □ Reg □ L □ XL

BA2_08. Arm that cuff was placed on: □ L □ R

Commented [EL1]: Note that height, weight, and waist circumference assessments previously here have been moved to the second in-person baseline assessment in order to reduce participant burden for those who do not meet blood pressure eligibility criteria at the first in-person assessment. As height and weight are not part of the eligibility criteria, we felt it was best to assess it after we determine if participants are eligible to be in study (i.e. have mean systolic/diastolic blood pressure ≥120/80 after first and second baseline assessments).

Medications (ME)

ME01. Do you take any prescription medications or over-the-counter drugs?

- ☐ No → ***skip to end of medications questions***
- ☐ Yes
- ☐ Don't know
- ☐ Prefer not to answer

ME02a. What is the name of the first prescription medication or over-the-counter drug that you take?

☐ Label product name: ____

☐ Label generic name: ____

☐ Don't know ☐ Prefer not to answer

ME02b. What is the dosage form?

Oral
- ☐ Pill, tablet, or capsule
- ☐ Sublingual or orally-disintegrating tablet
- ☐ Liquid solution or suspension (drink, syrup)
- ☐ Powder

Topical
- ☐ Liquid, cream, gel, or ointment
- ☐ Ear drops (otic)
- ☐ Eye drops (ophthalmic)
- ☐ Skin patch (transdermal)

Inhaled
- ☐ Inhaler or nebulizer

Injected
- ☐ Injection

Suppository
- ☐ Rectal (e.g., enema)
- ☐ Vaginal (e.g., douche, pessary)

Other:
- ☐ Don't know
- ☐ Prefer not to answer

ME02c. How frequently do you take it?

- ☐ _________ times per day
- ☐ _________ times per week
- ☐ _________ times per month
- ☐ Don't know
- ☐ Prefer not to answer

ME02d. What is the strength? ***(Record strength of how it is actually taken, not how it is prescribed.)***

- ☐ _________ %
- ☐ _________ mg
- ☐ _________ mcg
- ☐ _________ grams
- ☐ _________ I.U.
- ☐ _________ Other unit: _____________
- ☐ Don't know
- ☐ Prefer not to answer

ME02e. Total dosage **per day**. ***(Record strength of how it is actually taken, not how it is prescribed.)***

- ☐ _________ %
- ☐ _________ mg
- ☐ _________ mcg
- ☐ _________ grams
- ☐ _________ I.U.
- ☐ _________ Other unit: _____________
- ☐ Don't know
- ☐ Prefer not to answer

ME02f. Do you take it regularly or only as needed?

- ☐ Regularly
- ☐ Only as needed
- ☐ Don't know
- ☐ Prefer not to answer

ME02g. For how long have you been taking it?

- ☐ For _________ days
- ☐ For _________ weeks
- ☐ For _________ months
- ☐ For _________ years
- ☐ Don't know
- ☐ Prefer not to answer

ME02h. What is the medication used for?

____

Commented [EL2]: This was added to help identify participants' intended use of medications.

ME02i. ***Interviewer comments***:

____

ME02j. Do you take any other prescription medications or over-the-counter drugs?

- ☐ No → ***skip to end of medications questions***
- ☐ Yes
- ☐ Don't know
- ☐ Prefer not to answer

ME03a. What is the name of the next prescription medication or over-the-counter drug that you take?

☐ Label product name:

____

☐ Label generic name:

____

☐ Don't know ☐ Prefer not to answer

ME03b. What is the dosage form?

Oral

- ☐ Pill, tablet, or capsule
- ☐ Sublingual or orally-disintegrating tablet
- ☐ Liquid solution or suspension (drink, syrup)
- ☐ Powder

Topical
☐ Liquid, cream, gel, or ointment
☐ Ear drops (otic)
☐ Eye drops (ophthalmic)
☐ Skin patch (transdermal)

Inhaled
☐ Inhaler or nebulizer

Injected
☐ Injection

Suppository
☐ Rectal (e.g., enema)
☐ Vaginal (e.g., douche, pessary)

Other:
☐ Don't know
☐ Prefer not to answer

ME03c. How frequently do you take it?
☐ _________ times per day
☐ _________ times per week
☐ _________ times per month
☐ Don't know
☐ Prefer not to answer

ME03d. What is the strength? ***(Record strength of how it is actually taken, not how it is prescribed.)***
☐ _________ %
☐ _________ mg
☐ _________ mcg
☐ _________ grams
☐ _________ I.U.
☐ _________ Other unit: _____________
☐ Don't know
☐ Prefer not to answer

ME03e. Total dosage **per day.** ***(Record strength of how it is actually taken, not how it is prescribed.)***
☐ _________ %
☐ _________ mg
☐ _________ mcg
☐ _________ grams
☐ _________ I.U.
☐ _________ Other unit: _____________
☐ Don't know
☐ Prefer not to answer

ME3f. Do you take it regularly or only as needed?
☐ Regularly
☐ Only as needed
☐ Don't know
☐ Prefer not to answer

ME03g. For how long have you been taking it?
☐ For _________ days
☐ For _________ weeks
☐ For _________ months
☐ For _________ years
☐ Don't know
☐ Prefer not to answer

ME03h. What is the medication used for?

____

ME03i. ***Interviewer comments*:**

____

ME03j. Do you take any other prescription medications or over-the-counter drugs?

- ☐ No → ***skip to end of medications questions***
- ☐ Yes
- ☐ Don't know
- ☐ Prefer not to answer

ME04a. What is the name of the next prescription medication or over-the-counter drug that you take?

☐ Label product name:

____

☐ Label generic name:

____

☐ Don't know ☐ Prefer not to answer

ME04b. What is the dosage form?

Oral
- ☐ Pill, tablet, or capsule
- ☐ Sublingual or orally-disintegrating tablet
- ☐ Liquid solution or suspension (drink, syrup)
- ☐ Powder

Topical
- ☐ Liquid, cream, gel, or ointment
- ☐ Ear drops (otic)
- ☐ Eye drops (ophthalmic)
- ☐ Skin patch (transdermal)

Inhaled
- ☐ Inhaler or nebulizer

Injected
- ☐ Injection

Suppository
- ☐ Rectal (e.g., enema)
- ☐ Vaginal (e.g., douche, pessary)

Other:
- ☐ Don't know
- ☐ Prefer not to answer

ME04c. How frequently do you take it?

- ☐ _________ times per day
- ☐ _________ times per week
- ☐ _________ times per month
- ☐ Don't know
- ☐ Prefer not to answer

ME04d. What is the strength? ***(Record strength of how it is actually taken, not how it is prescribed.)***

- ☐ _________ %
- ☐ _________ mg
- ☐ _________ mcg
- ☐ _________ grams

☐ _________ I.U.
☐ _________ Other unit: _____________
☐ Don't know
☐ Prefer not to answer

ME04e. Total dosage **per day.** ***(Record strength of how it is actually taken, not how it is prescribed.)***

☐ _________ %
☐ _________ mg
☐ _________ mcg
☐ _________ grams
☐ _________ I.U.
☐ _________ Other unit: _____________
☐ Don't know
☐ Prefer not to answer

ME04f. Do you take it regularly or only as needed?

☐ Regularly
☐ Only as needed
☐ Don't know
☐ Prefer not to answer

ME04g. For how long have you been taking it?

☐ For _________ days
☐ For _________ weeks
☐ For _________ months
☐ For _________ years
☐ Don't know
☐ Prefer not to answer

ME04h. What is the medication used for?

____

ME04i. ***Interviewer comments***:

____

ME04j. Do you take any other prescription medications or over-the-counter drugs?

☐ No → ***skip to end of medications section***
☐ Yes
☐ Don't know
☐ Prefer not to answer

ME05a. What is the name of the next prescription medication or over-the-counter drug that you take?

☐ Label product name:

____

☐ Label generic name:

____

☐ Don't know ☐ Prefer not to answer

ME05b. What is the dosage form?

Oral
☐ Pill, tablet, or capsule
☐ Sublingual or orally-disintegrating tablet
☐ Liquid solution or suspension (drink, syrup)
☐ Powder

Topical
☐ Liquid, cream, gel, or ointment
☐ Ear drops (otic)
☐ Eye drops (ophthalmic)
☐ Skin patch (transdermal)

Inhaled
☐ Inhaler or nebulizer

Injected
☐ Injection

Suppository
☐ Rectal (e.g., enema)
☐ Vaginal (e.g., douche, pessary)

Other:
☐ Don't know
☐ Prefer not to answer

ME05c. How frequently do you take it?
☐ _________ times per day
☐ _________ times per week
☐ _________ times per month
☐ Don't know
☐ Prefer not to answer

ME05d. What is the strength? ***(Record strength of how it is actually taken, not how it is prescribed.)***
☐ _________ %
☐ _________ mg
☐ _________ mcg
☐ _________ grams
☐ _________ I.U.
☐ _________ Other unit: _____________
☐ Don't know
☐ Prefer not to answer

ME05e. Total dosage **per day.** ***(Record strength of how it is actually taken, not how it is prescribed.)***
☐ _________ %
☐ _________ mg
☐ _________ mcg
☐ _________ grams
☐ _________ I.U.
☐ _________ Other unit: _____________
☐ Don't know
☐ Prefer not to answer

ME05f. Do you take it regularly or only as needed?
☐ Regularly
☐ Only as needed
☐ Don't know
☐ Prefer not to answer

ME05g. For how long have you been taking it?
☐ For _________ days
☐ For _________ weeks
☐ For _________ months
☐ For _________ years
☐ Don't know
☐ Prefer not to answer

ME05h. What is the medication used for?

____

ME05i. ***Interviewer comments*:**

____

ME05j. Do you take any other prescription medications or over-the-counter drugs?

- ☐ No → ***skip to end of end of medications questions***
- ☐ Yes
- ☐ Don't know
- ☐ Prefer not to answer

ME06a. What is the name of the next prescription medication or over-the-counter drug that you take?

☐ Label product name:

____

☐ Label generic name:

____

- ☐ Don't know
- ☐ Prefer not to answer

ME06b. What is the dosage form?

Oral
- ☐ Pill, tablet, or capsule
- ☐ Sublingual or orally-disintegrating tablet
- ☐ Liquid solution or suspension (drink, syrup)
- ☐ Powder

Topical
- ☐ Liquid, cream, gel, or ointment
- ☐ Ear drops (otic)
- ☐ Eye drops (ophthalmic)
- ☐ Skin patch (transdermal)

Inhaled
- ☐ Inhaler or nebulizer

Injected
- ☐ Injection

Suppository
- ☐ Rectal (e.g., enema)
- ☐ Vaginal (e.g., douche, pessary)

Other:
- ☐ Don't know
- ☐ Prefer not to answer

ME06c. How frequently do you take it?

- ☐ _________ times per day
- ☐ _________ times per week
- ☐ _________ times per month
- ☐ Don't know
- ☐ Prefer not to answer

ME06d. What is the strength? ***(Record strength of how it is actually taken, not how it is prescribed.)***

☐ _________ %
☐ _________ mg
☐ _________ mcg
☐ _________ grams
☐ _________ I.U.
☐ _________ Other unit: _____________
☐ Don't know
☐ Prefer not to answer

ME06e. Total dosage **per day.** ***(Record strength of how it is actually taken, not how it is prescribed.)***
☐ _________ %
☐ _________ mg
☐ _________ mcg
☐ _________ grams
☐ _________ I.U.
☐ _________ Other unit: _____________
☐ Don't know
☐ Prefer not to answer

ME06f. Do you take it regularly or only as needed?
☐ Regularly
☐ Only as needed
☐ Don't know
☐ Prefer not to answer

ME06g. For how long have you been taking it?
☐ For _________ days
☐ For _________ weeks
☐ For _________ months
☐ For _________ years
☐ Don't know
☐ Prefer not to answer

ME06h. What is the medication used for?

____

ME06i. ***Interviewer comments*:**

____

ME06i. Do you take any other prescription medications or over-the-counter drugs?
☐ No → ***skip to end of medications questions***
☐ Yes
☐ Don't know
☐ Prefer not to answer

ME07a. What is the name of the next prescription medication or over-the-counter drug that you take?

☐ Label product name:
____
☐ Label generic name:
____

☐ Don’t know
☐ Prefer not to answer

ME07b. What is the dosage form?

Oral
☐ Pill, tablet, or capsule
☐ Sublingual or orally-disintegrating tablet
☐ Liquid solution or suspension (drink, syrup)
☐ Powder

Topical
☐ Liquid, cream, gel, or ointment
☐ Ear drops (otic)
☐ Eye drops (ophthalmic)
☐ Skin patch (transdermal)

Inhaled
☐ Inhaler or nebulizer

Injected
☐ Injection

Suppository
☐ Rectal (e.g., enema)
☐ Vaginal (e.g., douche, pessary)

Other:
☐ Don’t know
☐ Prefer not to answer

ME07c. How frequently do you take it?
☐ _________ times per day
☐ _________ times per week
☐ _________ times per month
☐ Don’t know
☐ Prefer not to answer

ME07d. What is the strength? ***(Record strength of how it is actually taken, not how it is prescribed.)***
☐ _________ %
☐ _________ mg
☐ _________ mcg
☐ _________ grams
☐ _________ I.U.
☐ _________ Other unit: _____________
☐ Don’t know
☐ Prefer not to answer

ME07e. Total dosage **per day.** ***(Record strength of how it is actually taken, not how it is prescribed.)***
☐ _________ %
☐ _________ mg
☐ _________ mcg
☐ _________ grams
☐ _________ I.U.
☐ _________ Other unit: _____________
☐ Don’t know
☐ Prefer not to answer

ME07f. Do you take it regularly or only as needed?
☐ Regularly
☐ Only as needed
☐ Don’t know
☐ Prefer not to answer

ME07g For how long have you been taking it?

- ☐ For _________ days
- ☐ For _________ weeks
- ☐ For _________ months
- ☐ For _________ years
- ☐ Don't know
- ☐ Prefer not to answer

ME07h. What is the medication used for?

____

ME07i. ***Interviewer comments***:

____

ME07j. Do you take any other prescription medications or over-the-counter drugs?

- ☐ No → ***skip to end of medications questions***
- ☐ Yes
- ☐ Don't know
- ☐ Prefer not to answer

ME08a. What is the name of the next prescription medication or over-the-counter drug that you take?

- ☐ Label product name:
____
- ☐ Label generic name:
____
- ☐ Don't know
- ☐ Prefer not to answer

ME08b. What is the dosage form?

<u>Oral</u>
- ☐ Pill, tablet, or capsule
- ☐ Sublingual or orally-disintegrating tablet
- ☐ Liquid solution or suspension (drink, syrup)
- ☐ Powder

<u>Topical</u>
- ☐ Liquid, cream, gel, or ointment
- ☐ Ear drops (otic)
- ☐ Eye drops (ophthalmic)
- ☐ Skin patch (transdermal)

<u>Inhaled</u>
- ☐ Inhaler or nebulizer

<u>Injected</u>
- ☐ Injection

<u>Suppository</u>
- ☐ Rectal (e.g., enema)
- ☐ Vaginal (e.g., douche, pessary)

<u>Other:</u>
- ☐ Don't know
- ☐ Prefer not to answer

ME08c. How frequently do you take it?

- ☐ _________ times per day
- ☐ _________ times per week
- ☐ _________ times per month
- ☐ Don't know

☐ Prefer not to answer

ME08d. What is the strength? *(Record strength of how it is actually taken, not how it is prescribed.)*

☐ _________ %
☐ _________ mg
☐ _________ mcg
☐ _________ grams
☐ _________ I.U.
☐ _________ Other unit: _____________
☐ Don't know
☐ Prefer not to answer

ME08e. Total dosage **per day.** ***(Record strength of how it is actually taken, not how it is prescribed.)***

☐ _________ %
☐ _________ mg
☐ _________ mcg
☐ _________ grams
☐ _________ I.U.
☐ _________ Other unit: _____________
☐ Don't know
☐ Prefer not to answer

ME08f. Do you take it regularly or only as needed?

☐ Regularly
☐ Only as needed
☐ Don't know
☐ Prefer not to answer

ME08g. For how long have you been taking it?

☐ For _________ days
☐ For _________ weeks
☐ For _________ months
☐ For _________ years
☐ Don't know
☐ Prefer not to answer

ME08h. What is the medication used for?

____

ME08i. ***Interviewer comments***:
____

ME08j. Do you take any other prescription medications or over-the-counter drugs?

☐ No → ***skip to end of medications questions***
☐ Yes
☐ Don't know
☐ Prefer not to answer

ME09a. What is the name of the next prescription medication or over-the-counter drug that you take?

☐ Label product name:
____
☐ Label generic name:
____

☐ Don't know ☐ Prefer not to answer

ME09b. What is the dosage form?

Oral
- ☐ Pill, tablet, or capsule
- ☐ Sublingual or orally-disintegrating tablet
- ☐ Liquid solution or suspension (drink, syrup)
- ☐ Powder

Topical
- ☐ Liquid, cream, gel, or ointment
- ☐ Ear drops (otic)
- ☐ Eye drops (ophthalmic)
- ☐ Skin patch (transdermal)

Inhaled
- ☐ Inhaler or nebulizer

Injected
- ☐ Injection

Suppository
- ☐ Rectal (e.g., enema)
- ☐ Vaginal (e.g., douche, pessary)

Other:
- ☐ Don't know
- ☐ Prefer not to answer

ME09c. How frequently do you take it?
- ☐ _________ times per day
- ☐ _________ times per week
- ☐ _________ times per month
- ☐ Don't know
- ☐ Prefer not to answer

ME09d. What is the strength? *(Record strength of how it is actually taken, not how it is prescribed.)*
- ☐ _________ %
- ☐ _________ mg
- ☐ _________ mcg
- ☐ _________ grams
- ☐ _________ I.U.
- ☐ _________ Other unit: _____________
- ☐ Don't know
- ☐ Prefer not to answer

ME09e. Total dosage **per day.** ***(Record strength of how it is actually taken, not how it is prescribed.)***
- ☐ _________ %
- ☐ _________ mg
- ☐ _________ mcg
- ☐ _________ grams
- ☐ _________ I.U.
- ☐ _________ Other unit: _____________
- ☐ Don't know
- ☐ Prefer not to answer

ME09f. Do you take it regularly or only as needed?
- ☐ Regularly
- ☐ Only as needed

☐ Don't know ☐ Prefer not to answer

ME09g. For how long have you been taking it?

☐ For _________ days
☐ For _________ weeks
☐ For _________ months
☐ For _________ years
☐ Don't know
☐ Prefer not to answer

ME09h. What is the medication used for?

____

ME09i. ***Interviewer comments*:**

____

ME09j. Do you take any other prescription medications or over-the-counter drugs?

☐ No → ***skip to end of medications questions***
☐ Yes
☐ Don't know
☐ Prefer not to answer

ME10a. What is the name of the next prescription medication or over-the-counter drug that you take?

☐ Label product name:

____

☐ Label generic name:

____

☐ Don't know ☐ Prefer not to answer

ME10b. What is the dosage form?

Oral
☐ Pill, tablet, or capsule
☐ Sublingual or orally-disintegrating tablet
☐ Liquid solution or suspension (drink, syrup)
☐ Powder

Topical
☐ Liquid, cream, gel, or ointment
☐ Ear drops (otic)
☐ Eye drops (ophthalmic)
☐ Skin patch (transdermal)

Inhaled
☐ Inhaler or nebulizer

Injected
☐ Injection

Suppository
☐ Rectal (e.g., enema)
☐ Vaginal (e.g., douche, pessary)

Other:
☐ Don't know
☐ Prefer not to answer

ME10c. How frequently do you take it?

☐ _________ times per day
☐ _________ times per week

☐ _________ times per month
☐ Don't know
☐ Prefer not to answer

ME10d. What is the strength? ***(Record strength of how it is actually taken, not how it is prescribed.)***

☐ _________ %
☐ _________ mg
☐ _________ mcg
☐ _________ grams
☐ _________ I.U.
☐ _________ Other unit: _____________
☐ Don't know
☐ Prefer not to answer

ME10e. Total dosage **per day.** ***(Record strength of how it is actually taken, not how it is prescribed.)***

☐ _________ %
☐ _________ mg
☐ _________ mcg
☐ _________ grams
☐ _________ I.U.
☐ _________ Other unit: _____________
☐ Don't know
☐ Prefer not to answer

ME10f. Do you take it regularly or only as needed?

☐ Regularly
☐ Only as needed
☐ Don't know
☐ Prefer not to answer

ME10g. For how long have you been taking it?

- ☐ For _________ days
- ☐ For _________ weeks
- ☐ For _________ months
- ☐ For _________ years
- ☐ Don't know
- ☐ Prefer not to answer

ME10h. What is the medication used for?

____

ME10i. ***Interviewer comments***: ____

Commented [EL3]: Note that questionnaires on anxiety and depression previously here have been moved to the second in-person baseline assessment in order to reduce participant burden for those who do not meet blood pressure eligibility criteria at the first in-person assessment. As these depression and anxiety questionnaires are not part of the eligibility criteria, we felt it was best to assess it after we determine if participants are eligible to be in study (i.e. have mean systolic/diastolic blood pressure ≥120/80 after first and second baseline assessments).

APPENDIX 2b

Second In-Person Visit Screening Questionnaire and Assessment Forms

SECOND IN-PERSON VISIT SCREENING QUESTIONNAIRE AND ASSESSMENT FORMS

Commented [EL1]: Note that this is the second in-person screening. The first in-person screening is just for blood pressure, as it is the only inclusion/exclusion criteria for the in-person screening. Blood pressure will be assessed on two different days at least a week apart from each other, to account for daily variations in blood pressure due to conditions such as stress. The second in-person screening measures blood pressure, in addition to other variables previously approved by the IRB including height, weight, waist circumference, depression and anxiety.

PID. Participant ID # __________
BA01. Staff ID # __________
BA02. Today's date (MMDDYY): ___________

Blood Pressure:
BA03a. Blood pressure 1st reading, systolic blood pressure: _________ mmHg
BA03b. Blood pressure 1st reading, diastolic blood pressure: __________ mmHg
BA03c. Blood pressure 2nd reading, systolic blood pressure: _________ mmHg
BA03d. Blood pressure 2nd reading, diastolic blood pressure: __________ mmHg
BA03e. Blood pressure 3rd reading, systolic blood pressure: _________ mmHg
BA03f. Blood pressure 3rd reading, diastolic blood pressure: __________ mmHg
BA03g. Blood pressure 4th reading, systolic blood pressure: _________ mmHg
BA03h. Blood pressure 4th reading, diastolic blood pressure: __________ mmHg
BA03i. Blood pressure 5th reading, systolic blood pressure: _________ mmHg
BA03j. Blood pressure 5th reading, diastolic blood pressure: __________ mmHg

BA04. Were the 4th and 5th systolic blood pressure readings within 20 mmHg of each other?
- □ Yes
- □ No ***(repeat measurements***

BA05. Were the 4th and 5th diastolic blood pressure readings within 10 mmHg of each other?
- □ Yes
- □ No ***(repeat measurements)***

BA06a. Repeated blood pressure 1st reading, systolic blood pressure: _________ mmHg
BA06b. Repeated blood pressure 1st reading, diastolic blood pressure: __________ mmHg
BA06c. Repeated blood pressure 2nd reading, systolic blood pressure: _________ mmHg
BA06d. Repeated blood pressure 2nd reading, diastolic blood pressure: __________ mmHg
BA06e. Repeated blood pressure 3rd reading, systolic blood pressure: _________ mmHg
BA06f. Repeated blood pressure 3rd reading, diastolic blood pressure: __________ mmHg
BA06g. Repeated blood pressure 4th reading, systolic blood pressure: _________ mmHg
BA06h. Repeated blood pressure 4th reading, diastolic blood pressure: __________ mmHg
BA06i. Repeated blood pressure 5th reading, systolic blood pressure: _________ mmHg
BA06j. Repeated blood pressure 5th reading, diastolic blood pressure: __________ mmHg

BA07. Blood pressure cuff size used: □ S □ Reg □ L □ XL

BA08. Arm that cuff was placed on: □ L □ R

BA09. Height: ______ . __ cm (one decimal place)

BA10. Weight: ______ . __ kg (one decimal place)

Waist Circumference:
BA11a. Measurement 1 ______ . __ cm (one decimal place)
BA11b. Measurement 2 ______ . __ cm (one decimal place)

BA12. Is the difference between Measurement 1 and Measurement 2 greater than 1.0cm?
□ Yes ***(repeat measurements 3&4)***
□ No

BA13a. Measurement 3 ______ . __ cm
BA13b. Measurement 4 ______ . __ cm

Questions for Participants to Answer on Their Own In-Person on Paper:
For each statement, please place a mark in the column that best describes how you have been feeling.

	Not at all or less than 1 day last week	1 or 2 days last week	3 to 4 days last week	5 to 7 days last week	Nearly every day for two weeks	I do not know	I prefer not to answer
DS_1. My appetite was poor.	☐	☐	☐	☐	☐	☐	☐
DS_2. I could not shake off the blues.	☐	☐	☐	☐	☐	☐	☐
DS_3. I had trouble keeping my mind on what I was doing.	☐	☐	☐	☐	☐	☐	☐
DS_4. I felt depressed.	☐	☐	☐	☐	☐	☐	☐
DS_5. My sleep was restless.	☐	☐	☐	☐	☐	☐	☐
DS_6. I felt sad.	☐	☐	☐	☐	☐	☐	☐
DS_7. I could not get going.	☐	☐	☐	☐	☐	☐	☐
DS_8. Nothing made me happy.	☐	☐	☐	☐	☐	☐	☐
C_9. I felt like a bad person.	☐	☐	☐	☐	☐	☐	☐
DS_10. I lost interest in my usual activities.	☐	☐	☐	☐	☐	☐	☐
DS_11. I slept much more than usual.	☐	☐	☐	☐	☐	☐	☐
DS_12. I felt like I was moving too slowly.	☐	☐	☐	☐	☐	☐	☐
DS_13. I felt fidgety.	☐	☐	☐	☐	☐	☐	☐
DS_14. I wished I were dead.	☐	☐	☐	☐	☐	☐	☐
DS_15. I wanted to hurt myself.	☐	☐	☐	☐	☐	☐	☐
DS_16. I was tired all the time.	☐	☐	☐	☐	☐	☐	☐
DS_17. I did not like myself.	☐	☐	☐	☐	☐	☐	☐
DS_18. I lost a lot of weight without trying to.	☐	☐	☐	☐	☐	☐	☐
DS_19. I had a lot of trouble getting to sleep.	☐	☐	☐	☐	☐	☐	☐
DS_20. I could not focus on the important things.	☐	☐	☐	☐	☐	☐	☐

Below is a list of common symptoms of anxiety. Please carefully read each item in the list. Indicate how much you have been bothered by that symptom during the past month, including today, by marking the box in the corresponding space in the column next to each symptom.

	Not At All	Mildly – it didn't bother me much	Moderately – it wasn't pleasant at all times	Severely – it bothered me a lot	I do not know	I prefer not to answer
BE_1. Numbness or tingling	☐	☐	☐	☐	☐	☐
BE_2. Feeling hot	☐	☐	☐	☐	☐	☐
BE _3. Wobbliness in legs	☐	☐	☐	☐	☐	☐
BE _4. Unable to relax	☐	☐	☐	☐	☐	☐
BE _5. Fear of worst happening	☐	☐	☐	☐	☐	☐
BE _6. Dizzy or lightheaded	☐	☐	☐	☐	☐	☐
BE _7. Heart pounding/racing	☐	☐	☐	☐	☐	☐
BE _8. Unsteady	☐	☐	☐	☐	☐	☐
BE _9. Terrified or afraid	☐	☐	☐	☐	☐	☐
BE _10. Nervous	☐	☐	☐	☐	☐	☐
BE _11. Feeling of choking	☐	☐	☐	☐	☐	☐
BE _12. Hands trembling	☐	☐	☐	☐	☐	☐
BE _13. Shaky/unsteady	☐	☐	☐	☐	☐	☐
BE _14. Fear of losing control	☐	☐	☐	☐	☐	☐
BE _15. Difficulty in breathing	☐	☐	☐	☐	☐	☐
BE _16. Fear of dying	☐	☐	☐	☐	☐	☐
BE _17. Scared	☐	☐	☐	☐	☐	☐
BE _18. Indigestion	☐	☐	☐	☐	☐	☐
BE _19. Faint/lightheaded	☐	☐	☐	☐	☐	☐
BE _20. Face flushed	☐	☐	☐	☐	☐	☐
BE _21. Hot/cold sweats	☐	☐	☐	☐	☐	☐

SAFETY PLAN

After the participant has completed the Beck Anxiety Inventory questionnaire and the Centers for Epidemiologic Studies Depressive Symptomatology questionnaire, ask the participant to wait for a few minutes while you check that all forms and assessments are completed. While the participant is waiting in a different room, check the scores according to the criteria below. If any scores trigger the safety plan, move forward with steps below as written.

Beck Anxiety Inventory (BA)

If participant scores ≥26 on the Beck Anxiety Inventory, while having the participant wait, immediately notify the collaborating psychiatrist, Dr. Ellen Flynn (cell phone # 401-258-9829), to determine need for a psychiatric consultation. She has provided her cell phone #, and your phone # will be entered into her phone recognition software to indicate it is a call from the MBHT study. It is likely she will answer the phone. If Dr. Flynn cannot be reached, please leave a phone message for her with your contact information and provide her with the Beck Anxiety Inventory results on the message. If Dr. Flynn is not immediately reachable, or following participant release after consulting with Dr. Flynn, immediately contact the PI, Dr. Eric Loucks (cell phone #401-369-0443).

Depressive Symptomatology (DS)

The CESD-R will be administered during the in-person assessment visits, and scores will be reviewed immediately upon completion of the in-person assessments.

1. Sadness (dysphoria): Question numbers 2,4, 6
2. Loss of Interest (anhedonia): Question numbers 8, 10
3. Appetite: Question numbers 1, 18
4. Sleep: Question numbers 5, 11, 19
5. Thinking / concentration: Question numbers 3, 20
6. Guilt (worthlessness): Question numbers 9, 17
7. Tired (fatigue): Question numbers 7, 16
8. Movement (agitation): Question numbers 12, 13
9. Suicidal ideation: Question numbers 14, 15

Participants are considered to meet criteria for major depressive episode if they have anhedonia or dysphoria nearly every day for the past two weeks, plus symptoms in an additional 4 DSM symptom groups noted as occurring nearly every day for the past two weeks. If participants meet criteria for major depressive episode, while having the participant wait, immediately notify the collaborating psychiatrist, Dr. Ellen Flynn (cell phone # 401-258-9829), to determine need for a psychiatric consultation. She has provided her cell phone #, and your phone # will be entered into her phone recognition software to indicate it is a call from the MBHT study. It is likely she will answer the phone. If Dr. Flynn cannot be reached, please leave a phone message for her with your contact information and provide her with the Depressive Symptomatology results on the message. If Dr. Flynn is not immediately reachable, or following participant release after consulting with Dr. Flynn, immediately contact the PI, Dr. Eric Loucks (cell phone #401-369-0443).

If participants respond having any suicidal ideation (DS questions 14 or 15), perform the following 2 steps:

1. **Immediately call 911 and Dr. Ellen Flynn.**

Specifically, while the participant is in the waiting room, ***call 911 immediately, and tell them:***

"My name is _____. I am working on a research study at Brown University. I have a study participant in the waiting room who has shared that he/she is currently suicidal." Please provide the participants' name to the 911 operator, as requested.

Finally, after calling 911, please call Dr. Ellen Flynn, who is the psychiatrist supporting this study. Her cell phone # is 401-258-9829. Please provide her with the same information as was done during the 911 call.

2. **While speaking calmly with the participant, let them know that you have called 911 and Dr. Flynn, and why (i.e. because we are concerned about you). You can speak with participant to keep him/her in the waiting room if 911 has sent assistance, but the discussion should not be clinical in nature.**

Examples of questions that could be asked in order to keep them in the waiting room:
- "Tell me what is going on."
- "What's happening right now?"
- Tell me more about why you are interesting in being part of this study.
- What are you hoping to get out of this study?

The following information can be provided to study participants.

National Suicide Prevention Lifeline: 1-800-273-8255

Other options are to:
- Call your doctor's office
- Call 911 for emergency services
- Go to the nearest hospital emergency room.

Local Non-Urgent Free or Inexpensive Mental Health Services:
Gateway Healthcare: 401-729-8701
Anchor Counseling Center: 401-475-9979
The Providence Center: 401-276-4020

APPENDIX 3

Phone-Delivered Screening Questionnaire

PHONE SCREENING QUESTIONNAIRE PART 1

*The script is shown below in **bold italics.***

PID. Participant ID: ___________

SQ01. Staff ID: ______________

SQ02. Today's Date (MMDDYY): ________________

SQ03. Current Time (24 hour time, e.g. 14:45): ________

Please now call the participant.

SQ04. Was the participant reached? YES NO

Hello, my name is ____________. I am calling from the Brown University School of Public Health because (name of participant) expressed interest in participating in our mindfulness blood pressure study. Is he/she available to talk at this time?

I would like to do a 10-15 minute phone interview with you to determine if you are a good match for this particular study.

SQ05. ***Is now a good time to speak?***

[If yes, proceed to SQ05 script below]

If no… ***When would be a good time to talk?***

SQ06. Day to call back (DDMMYY) ____________

SQ07. Time to call back ______ AM/PM

OK, great. How about I give a quick overview of the study, and can then answer any questions you may have to see if it is a good fit for you. We will then go through a screening questionnaire to see if you qualify for the study. Does this sound OK?

A basic overview of the study is that it is looking to see if mindfulness practices improve blood pressure, and if education about hypertension risk factors may also improve blood pressure. Specifically, we will provide training in meditation, mindful movements, and the roles of things like diet, physical activity and medication in reducing blood pressure. You will be taught by a very experienced teacher who in an expert in these fields. The course takes place over a 9-week period, where you come to a class once each week for 2.5 hours each time. There is also a one-day retreat on a Saturday that will be 8 hours long. Also, we will ask you to participate in health assessments before and after the study. Health assessments include measures such as blood pressure, height and weight, and questionnaires about your health and experiences.

Can I answer any questions about the study that you may have? [answer any they may have.]

SQ08. ***Does this study sound like something you would be interested in doing? Yes No***

[If yes, proceed to next statement below. If no, politely thank the participant for considering being in this study, and end the call].

Great. There are a few things that I would like to make clear before we start the interview. First of all, some of the questions that I will ask now to figure out if you are eligible to be in this study will be of personal nature, including asking about your mental health and life's experiences.

SQ09. ***Are you in a private place to talk?***

[If yes, proceed to text below. If no, reschedule meeting using variables SQ04 and SQ05 above].

Because this interview is of a personal nature, it is important that you understand that everything you say will be kept strictly confidential. No one outside of our project will ever be able to see your answers, and we will not keep your name in the same place as any of your answers. If you are not eligible after the phone screen, we will destroy your information. If you like, though, we can keep your information on file for future studies.

SQ10. Participant's First Name: ____________________________________

SQ11. Participant's Last Name:______________________________________

Participant's Address (in case we need to send any study materials to you):
SQ12a. Street address: _____________
SQ12b. City: ____________________
SQ12c: State: ________
SQ12d: Zip Code: ________________

SQ13a. Participant's Phone number #1 (in case we need to contact you by phone)
____

SQ13b. Participant's Phone number #2____________________________________

SQ14. Participant's email address (or mailing address if no email):______________________

SQ15. Notes from interviewer related to participants' contact information (if any):
____
____
____

Commented [EL1]: We added this to help participants understand why we are asking for their personal information.

Commented [EL2]: We added this to help participants understand why we are asking for their personal information.

PHONE SCREENING QUESTIONNAIRE PART 2

PID. Participant ID #: ______________

INCLUSION CRITERIA: All answers in 3rd column must be YES. If an answer is NO, immediately proceed to question SQ40.			
			Comments
SQ26.	***What is your age?*** [Is age at least 18 years?]	YES NO	
SQ27.	***Can you read and write in English?***	YES NO	

EXCLUSION CRITERIA: All answers in 3rd column must be NO with the exception of SQ32a and SQ33a. If an answer (other than SQ32a and SQ33a) is YES, then immediately proceed to question SQ40.			
			Comments
I will now start to ask some questions about your mental health.			
SQ28.	***Has anyone ever told you that you have bipolar disorder or manic depression?***	**YES NO**	
SQ29.	***Has anyone ever used the word "Borderline" to describe you?***	**YES NO**	
SQ30a.<br><br>SQ30b.	***Have you ever had a hallucination or seen things that other people can't see, or hear things other people can't hear?***<br><br>***Have you ever been diagnosed with schizophrenia or psychosis?***	**YES NO**<br><br>**YES NO**	
 <br>SQ31_1<br>SQ31_2<br>SQ31_3<br>SQ31_4<br>SQ31_5<br>SQ31_6<br>SQ31_7<br>SQ31_8	***Have you ever taken any of the following medications that I am about to read to you?***<br>***Lithium***<br>***Seroquel (quetiapine)***<br>***Abilify (aripiprazole)***<br>***Zyprexa (olanzapine)***<br>***Clozaril (clozapine)***<br>***Haldol/Haloperidol***<br>***Geodon (ziprasidone)***<br>***Risperdal (risperidone)***	 <br>**YES NO DK**<br>**YES NO DK**<br>**YES NO DK**<br>**YES NO DK**<br>**YES NO DK**<br>**YES NO DK**<br>**YES NO DK**<br>**YES NO DK**	
SQ32a<br><br>SQ32b<br><br>SQ32c<br><br>SQ32d	***Have you ever had a suicide attempt?***<br><br>***[If yes, ask…] Have you considered killing yourself during the past month?***<br><br>***[If yes, ask…] Are you currently suicidal?***<br><br>***[If yes, keep participant on the phone, and follow suicide safety plan below]***<br><br>***[If no, ask…] Are you getting any help for that? If not, then provide list of resources from Safety Plan including Gateway, Anchor and The Providence Center] Not urgent, but inform Dr. Flynn about what was discussed.***	**YES NO**<br><br>**YES NO**<br><br>**YES NO**<br><br>**YES NO**	

EXCLUSION CRITERIA: All answers in 3rd column must be NO with the exception of SQ32a and SQ33a. If an answer (other than SQ32a and SQ33a) is YES, then immediately proceed to question SQ40.			
SQ33a<br><br>SQ33b<br><br>SQ33c	***Would you say you have a trauma history?***<br>***[If yes…]***<br>***In the past month, have you had any problems with dissociation (memory loss)?***<br>***In the past month, have you had any flashbacks (i.e. sudden and disturbing vivid memory) about the trauma?***	**YES NO**<br><br>**YES NO**<br><br>**YES NO**	
SQ34a<br><br>SQ34b	***In the past month, have you had any problems with obsessions or compulsions, such as washing your hands or checking the oven over and over again?***<br>***[If yes…] Has anyone diagnosed you with obsessive compulsive disorder?***	**YES NO**<br><br>**YES NO**	
SQ35	***In the past month, have you had a panic attack (i.e. sweating, heart palpitations, nausea, trouble breathing, fear of dying/choking/going crazy)?***	**YES NO**	
SQ36	***Have you had any problems with alcoholism or drug use in the past year?***	**YES NO**	
SQ37	***In the past year, have you had an eating disorder, such as starving, binge eating, or vomiting?***	**YES NO**	
SQ38a<br><br>SQ38b<br><br>SQ38c	***Do you currently have a mindfulness practice, such as meditation or yoga?***<br><br>***Please tell me more about your mindfulness practice, including how often you practice per week.***<br><br>***Do you currently practice meditation more than once per week? (yoga does not count as meditation in this context)***	**YES NO**<br><br>**Fill in response in comments section to the right**<br><br>**YES NO**	
SQ39	***This class will take place at Brown University in-person. Do you have any medical or mobility issues that would affect you being able to attend class?***	**YES NO**	
SQ40	Participant qualifies for next step of study (next step is 1st blood pressure screening)	**YES NO**<br><br>**If YES go to SQ41.**<br><br>**If NO, go to SQ42.**	

SQ41a. ***Thank you for taking the time to answer these questions. You qualify for the next stage of screening, which is to take your blood pressure at two different times at least a week apart from each other. Let's book the in-person screening sessions*** [Book both sessions. We can cancel the second session if they do not qualify].

SQ41b. ***We will be scheduling the mindfulness intervention at a time that works best with most of the participants. What days and times of the week typically work best with your schedule? Please keep in mind that the sessions are 2.5 hours long, and take place once per week for 9 weeks.***

Commented [EL3]: This was added to help schedule the intervention at a time best for most participants.

Thank you for your time and interest in this study. We look forward to meeting you in person. Do you have any questions before we end this call?

SQ42a***. Thank you for taking the time to answer these questions. According to the survey, you do not qualify for the study at this time. There may be other studies you quality for.***

SQ42b. ***Would you like me to keep your information to pass on to these studies?*** YES / NO

SQ42c. [If yes…] ***OK, thank you. We will keep this information for future studies you may qualify for. Thank you for taking the time to talk with me today. Can I answer any questions before hanging up?***

SQ42d. [If no…] ***OK, our copy of this information will be destroyed. Thank you for taking the time to talk with me today. Can I answer any questions before hanging up?***

SAFETY PLAN

Enter details below on paper during screening (These variables should are also be entered in the survey via questions SQ10-SQ13). Destroy this paper after screening is complete.

Participant's First Name: ____________________________________

Participant's Last Name:______________________________________

Participant's Address:

Street address: _____________

City: ____________________

State: ________

Zip Code: ________________

Participant's Phone number #1: ____________________________________

Participant's Phone number #2____________________________________

Commented [EL4]: This is data entered by staff on paper as participants provide this information above. If participants state they are currently suicidal, we can then bring this paper to another staff member to call 911, and it will have the participants' contact info immediately available for the 911 operator.

During the phone-based screening, if participants respond yes to "Are you currently suicidal?", the interviewer should perform the following 2 steps:

1. **Immediately have 911 and Dr. Ellen Flynn called by a colleague who has been informed beforehand that this is a possibility.**

Specifically, while keeping the participant on the phone, show the text below in the box to a colleague.

I have a study participant on the phone who is currently suicidal. Please call 911 immediately, and tell them:

"I am calling on behalf of [my name] who is performing a research study at Brown University. He has a participant on the phone who says they are currently suicidal." Please provide the participants' contact information to the 911 operator (i.e. name, address, phone #, email address) as requested. This information is shown above.

Finally, after calling 911, please call Dr. Ellen Flynn, who is the psychiatrist supporting this study. Her cell phone # is 401-258-9829. Please provide her with the same information as was done during the 911 call.

Commented [EL5]: This box was added to increase clarity to person who would make 911 call.

2. **While speaking calmly with the participant, let them know what you are doing. Specifically, let them know we are calling our study's psychiatrist Dr. Flynn and 911, and why you are doing that (i.e. because we are concerned about you). You can speak with participant to keep him/her on the phone, but the discussion should not be clinical in nature.**

Examples of questions that could be asked in order to keep them on the phone:

- "Tell me what is going on."
- "What's happening right now?"
- Tell me more about why you are interesting in being part of this study.
- What are you hoping to get out of this study?

The following information can be provided to study participants if they state they have had considered killing themselves in the past month. If they are currently suicidal, the main priority is to keep them on the phone while 911 and Dr. Flynn are being contacted.

National Suicide Prevention Lifeline: 1-800-273-8255

Other options are to:

- Call your doctor's office
- Call 911 for emergency services
- Go to the nearest hospital emergency room.

Local Non-Urgent Free or Inexpensive Mental Health Services:
Gateway Healthcare: 401-729-8701
Anchor Counseling Center: 401-475-9979
The Providence Center: 401-276-4020

APPENDIX 4

Informed Consent Form

Agreement to Participate in a Research Study

Investigation of the Effects of Mindfulness on Blood Pressure and Well-Being

You are being asked to take part in a Brown University research study about the effects of mindfulness and hypertension education on blood pressure and risk factors for hypertension. This form will explain the purpose of the study, how the study will be carried out and what you will be expected to do. It will also explain the possible risks and possible benefits of being in the study. If any part of the following description is not clear to you, you are encouraged to contact the researcher to answer any questions before you decide whether to take part in the study. If you decide to participate, please fill out and sign the last page of this form.

1a. Nature and Purpose of the Study

The purpose of the study is to investigate the impact of mindfulness practices and health education on blood pressure. You have been selected for this study because you expressed interest and met entrance criteria for having prehypertension or hypertension, or another cardiovascular risk factor that could be influenced by this program. In order to assess the effects of these practices, you will be asked to complete some questionnaires, and a laboratory assessment before and after learning the mindfulness practices.

1b. Explanation of Procedures

If you agree to participate, you will be asked to consent to the following:

1) Participate in an interview in which you will be asked questions about past and present mental health, including depression and suicide.

2) Complete questionnaires about a wide range of topics, including your diet, physical activity, smoking, medication use, personality, emotions, attention and past experiences, including stressful or traumatic experiences. These questions will probe sensitive psychological areas, including physical, emotion and sexual abuse. These questionnaires may take up to 2 hours to complete. By completing the interviews and questionnaires, you are giving the researchers permission to use the information you have provided. You have the right not to answer any of the questions.

3) Directly assessed blood pressure, height, weight, waist circumference and hip circumference.

4) You will be asked to perform some cognitive tasks. One of these tasks may involve computer-based tests of attention or decision-making. Together these tests may take as long as 45 minutes.

5) You will participate in the mindfulness program, which consists of 9 weekly sessions of 2.5 hours each and will include one 8 hour weekend retreat. Daily homework assignments may take as long as one hour and consist of practicing mindfulness exercises with the aid of a guided audio CD and completing worksheets related to stress, thoughts, and common reactions to various types of events.

6) Class sessions will be audio taped so we can analyze the quality of the treatment you receive. The recordings will be transcribed so that we may analyze the text. The recordings will be identified by study number, will only be heard by study staff and will be destroyed after transcription.

7) You will be asked to complete a few short questionnaires each week during the 9 week condition.

8) After 9 weeks, you will be asked to complete questionnaires and return to the laboratory to repeat the same procedures for a second day of testing.

9) Three months after the end of the 9 week condition, you will be asked to return to the laboratory to repeat the same testing procedures.

Table Summarizing Activities and Time Commitment for this Study.

**Activity**	**Time Commitment**
First blood pressure screen	30 minutes
Second blood pressure screen	30 minutes
Baseline health assessments, such as questionnaires, height, weight and waist circumference.	2 hours
Mindfulness course	9 sessions that are 2.5 hours each.<br>1 retreat day on a Saturday that will be 8 hours<br>Total course time: 30.5 hours
Home mindfulness practices assigned during course.	1 hour per day, 6 days per week, for 8 weeks.<br>Total time: 48 hours.
Health assessments, such as questionnaires, blood pressure, height, weight and waist circumference, that take place immediately after course completion.	2 hours
Health assessments, such as questionnaires, blood pressure, height, weight and waist circumference, that take place that take place 3 months after course completion.	2 hours
TOTAL TIME COMMITMENT FOR STUDY	85.5 hours

Feedback:

At the end of the study, you will receive individual feedback about the changes that occurred since the first assessment. Specifically, you will receive an individualized handout listing % change (increase or decrease) on scales of attention, stress, mood, health behaviors, weight, and blood pressure across the study.

2. Discomforts and Risks

The questionnaires are routine, standardized forms for epidemiologic research. Certain questions may be upsetting as they may probe sensitive psychological areas and inquire about upsetting or traumatic events, including physical, sexual or emotional abuse and/or current psychiatric symptoms. Since your participation is voluntary, you have the right to skip any questions that make you uncomfortable.

Meditation-based interventions may results in discomfort with attention to unpleasant thoughts, feelings or body sensations. Some individuals may experience an initial increase in undesirable feelings with increased attention to them.

3. Benefits

We cannot and do not guarantee or promise that you will receive any direct benefits from this study. However, participation in the study creates the potential benefit of a) identifying effective treatments for elevated blood pressure, b) gaining knowledge of the effects of mindfulness practices, c) receiving information about your psychological and physical functioning.

4. Alternative Therapies

A number of different therapies, including antihypertensive medication, diet changes, physical activity, and reducing excessive alcohol consumption may also be beneficial for reducing blood pressure. Education about these therapies are integrated into this course, but other forms of these alternative therapies are also available in the community.

5. Confidentiality

Your responses for this study will be kept confidential. All data that we collect will be linked to a study ID# instead of your name. All questionnaires in this study will be filled out through an online survey or a paper version of the survey if you prefer. All of these questionnaires will be linked solely to your study ID#, so that your identity is protected and your answers are confidential. Although these measures have been taken to protect your personal information, complete confidentiality cannot be guaranteed when transmitting information over the internet.

While your confidentiality is protected to the extent of the law, there are limitations to confidentiality. If your questionnaire responses indicate that you pose a serious danger to yourself or to another person, then a collaborator (Dr. Ellen Flynn) who is a licensed psychiatrist, may contact you to discuss your responses and possible referral to a treatment provider. Questionnaire items that may warrant follow-up include endorsements of statements about hurting yourself, any high scores in depression, anxiety, or other clinically significant problems. You should also know that there are times when the law might require the release of your responses without your permission. For example, State law requires researchers to report abuse or neglect of children to the Department of Children, Youth and Families (DCYF). State law also requires researchers to report abuse or neglect of people age 60 and older to the Division of Elderly Affairs.

The findings of the study may be used for medical publication. Your name will not be used in any published reports about this study. Results will be reported in a summarized manner in such a way that you cannot be identified. All personally identifiable information will be "de-identified" and only a unique code number will be used. Study records will be identified with a

unique code number and initials. All study records and specimens will be stored in a secure storage area.

Keeping study records: The Principal Investigator for this study will keep your research records indefinitely for research purposes.

6. Refusal/Withdrawal

Participation is voluntary, and you may decide to opt out or refuse any part of participation, including not answering certain questions. If you decide now to participate, you can change your mind later and quit the study. The decision to not participate or to withdraw from the study will not adversely affect current or future interactions with Brown University. The decision to not participate or to withdraw from the study will also not adversely affect your relationship with your physician.

If you decide not to participate, or if you quit the study, we will provide you with referrals for alternative treatments, if desired.

7. Contact Information

If you have questions about study procedures at any time you may contact the researchers: Dr. Eric B. Loucks, email: eric.loucks@brown.edu, telephone (401) 863-6283. If you would like more information about the rules for research studies, or the rights of people who take part in those studies, you may contact the Brown University Research Protections Office, telephone number 1-866-309-2095 or 401-863-3050.

CONSENT FORM:
Please sign and return.

Please read the following agreement and sign to consent to participating.

I HAVE READ THE ABOVE DESCRIPTION OF THIS STUDY. ALL OF MY QUESTIONS HAVE BEEN SATISFACTORILY ANSWERED, AND, I AGREE TO PARTICIPATE IN THIS RESEARCH STUDY.

____
PRINT NAME

__________________________________ ________________
Signature of participant **Date**

CONTACT INFORMATION

Name (print):____

Permanent Address:____

Email(s):____

Telephone:_______________________**(cell)** _______________________**(other)**

Brown University
Research Protections Office
Institutional Review Board
Amendment Request

Date of Request: 9/1/15 **Investigator's Name and Title:** Eric Loucks, Assistant Professor

Study Title: Mindfulness-Based Hypertension Therapy Pilot Study (#1412001171)

Original Type of Review: ☐ Exempt ☐ Expedited ☒ Full Board

1) **Provide a brief lay summary of the overall project. Include enough detail to allow the IRB to evaluate the requested change(s) within the context of the overall project.** (Attach summary to this form) Please see attached.

2) **Provide a detailed description of the changes being requested** (Use additional pages, if necessary):
Please see attached.

3.) **State the reason (justification) for the requested amendment.** (Use additional pages, if necessary):
Please see attached.

4.) **What is your assessment of how the changes will affect the overall risk/benefit ratio of the study and the willingness of individuals to participate?**
Please see attached.

5.) **Does the requested amendment require new documents or changes to the approved consent form or other documents?**

- ☒ Consent/assent documents (attach revised version with changes highlighted)
- ☒ New/revised instruments (attach - if revised, highlight changes)
- ☐ New/revised advertising materials (attach - if revised, highlight changes)

Do you have a conflict of interest on this project according to Brown's policy? ☐YES ☒NO
If YES, has this conflict been previously disclosed to the IRB? ☐YES ☐NO

PI signature: [signature] Date: 9/1/15

1) Provide a brief lay summary of the overall project. Include enough detail to allow the IRB to evaluate the requested change(s) within the context of the overall project.

The World Health Organization reported that suboptimal blood pressure (BP) is responsible for more than half of cardiovascular disease mortality world-wide. Furthermore, greater than half of those with hypertension have uncontrolled BP. *A 2009 Institute of Medicine report recommended prioritizing research to "Compare the effectiveness of mindfulness-based interventions (e.g. yoga, meditation, deep breathing training) and usual care in treating… cardiovascular risk factors."* Evidence-based mindfulness interventions, including Mindfulness-Based Stress Reduction, may have some effects on blood pressure, where a recent meta-analysis and systematic review of 4 randomized controlled trials demonstrated significant effects, but evidence of heterogeneity in effect sizes. The methodologically highest quality studies had the smallest effect sizes (range 0-5 mmHg). Mindfulness-Based Stress Reduction (MBSR) has been customized to a number of disease processes, such as Mindfulness-Based Cognitive Therapy for patients with recurrent depression, and Mindfulness-Based Relapse Prevention for patients with substance use addictions. Effect sizes have been increased by customizing mindfulness interventions to diseases of interest. The same may be true for hypertension, *however mindfulness interventions customized for prehypertensive/hypertensive patients have never been investigated. Until methodologically rigorous studies to evaluate customized interventions for hypertension are performed, we will not know if the observed preliminary effects of general mindfulness interventions on blood pressure reduction could be much more effective with a tailored approach.* Consequently, we propose to conduct a stage I behavioral therapy intervention study to evaluate whether MBSR customized to prehypertensive and hypertensive patients has the potential to provide clinically relevant reductions in BP. Consequently the specific aims are:

Stage 1a: Therapy Development/Manual Writing

1 To outline and evaluate key novel elements of mindfulness-based hypertension therapy (MBHT), customized from the evidence-based MBSR. *We hypothesize that the most important novel element will be generation of mindfulness skills specifically applied to hypertension risk factors such as diet, physical activity, obesity, alcohol consumption and antihypertensive medication adherence.* This aim will be achieved using (1) focus groups of participants undergoing the MBHT behavioral intervention, (2) discussion with experts (including cardiologists, epidemiologists, mindfulness experts, mindfulness intervention instructors) prior to, and following pilot testing of MBHT in participants, and (3) clinical judgment of the investigators performing the intervention.

2 To determine effectiveness of MBHT on primary outcomes (systolic blood pressure, retention rates, recruitment rates, and adverse effects) and secondary outcomes (hypertension risk factors such as diet, physical activity, obesity, and antihypertensive medication adherence) in hypertension subgroups, specifically participants with (1) prehypertension, (2) controlled hypertension, and (3) uncontrolled hypertension. Initial decisions about the targeted sample based on hypertension status will be made.

3 To develop an MBHT therapist manual and training program, including procedures for training, supervising, and evaluating therapists. Furthermore, acceptable therapist characteristics will be developed. The manual and training program will include themes such as specification of unique and common elements of MBHT *vs.* other interventions, description of interventions excluded from MBHT, and specification of key treatment parameters such as frequency and duration of treatment, session length, topics addressed, sequence of sessions, as well as therapist adherence and competency measures. The MBHT training will consist of a therapist manual, a formal didactic training seminar, and at least one closely supervised training session.

Stage Ib: Pilot Trial

4 To determine whether a mindfulness-based hypertension therapy (MBHT) intervention, customized from the evidence-based MBSR, has promise to be an effective behavioral therapy for participants with hypertension and/or prehypertension. We will perform a randomized controlled pilot trial for MBHT *vs.*

enhanced usual care control. *We hypothesize that MBHT will have adequate recruitment rates (≥10% of prehypertensive/hypertensive participants invited from physicians' offices), fairly low drop out rates (<15%), and medium effect sizes (e.g. 5-10 mmHg systolic BP) for reduction in blood pressure.*

These findings will provide publishable pilot data that will inform future randomized clinical trials that evaluate effects of MBHT on long-term changes in blood pressure *vs.* usual care and active control groups. *If proven effective, MBHT could be offered as a complementary program in the prehypertensive/hypertensive patient population that contributes to over half of the cardiovascular disease mortality world-wide.*

2) Provide a detailed description of the changes being requested (Use additional pages, if necessary):

1. Change in funding source
    a. We have an NIH grant application (1 UH2 AT009145-01 entitled "Mindfulness Influences on Self-Regulation: Mental and Physical Health Implications") pending that would provide funding to this project.
2. Change in scope
    a. The NIH grant would provide approximately $5 million over 5 years to support secondary data analyses on data for which personally identifiable information is removed, and hence not human subjects-related. Furthermore, the grant would provide additional measures to the MBHT study above, as well as potentially providing additional measures to three other studies named in the grant application as Concurrent Study B1, B2 and C (more details below). Note that Concurrent Study A in the grant (currently approved by Brown IRB) is nearing completion, and no additional measures will be added. All of these studies are currently funded and underway. IRB approval has been obtained for all studies by the host institutions (IRB approval certificates shown in **Appendix 1**). The current grant will add some fairly low participant burden measures to each study. Each of the study's host institutions proposes to perform the following additional measures, with associated timeline. IRB approval will be obtained by the host institutions prior to any additional measures being assessed.
        i. Concurrent Study B1: Yoga Program and Dietary Health Behaviors (PI: Sara Lazar, Massachusetts General Hospital, Harvard Medical School)
            1. The grant proposes to add neuroimaging to study participants. This imaging is scheduled to take place no earlier than January 2016.
        ii. Concurrent Study B2: Mindfulness-Based Stress Reduction and Dietary Health Behaviors (PI: Carl Fulwiler, UMass Medical School, Worcester).
            1. Additional measures may not be needed for this study, as the major variables are being assessed. If it appears that additional variables are needed, IRB approval will be obtained.
        iii. Concurrent Study C: MINDFUL-PC Study (PI: Zev Schuman-Olivier, Cambridge Health Alliance, Harvard Medical School). All of the following additional measures proposed in the grant will go through IRB approval at the host institution, and should represent a fairly low increase in participant burden.
            1. During Phase 1 (years 2016-2017), we will adapt and pilot mechanistic targets that we found to be associated with successful behavioral self-regulation during MBI trials for addiction (smoking cessation).[250,251] We will pilot an fMRI-compatible stop-signal task measuring inhibitory control,[252] self-report measures of self-management self-efficacy, as well as self-report[106,151] and neuroimaging tasks assessing self-compassion[253] and trait non-judgment of self.[254] We will adapt and test these targets in this primary care population in order to assess

their applicability to medical regimen adherence, and assess them for relevance and feasibility in the context of the MTPC study.

2. During Phase 2 (2016-2018), we will test the best performing targets from the three domain working groups by recruiting patients with poor medical regimen adherence and difficulty with daily medication adherence with the most promising self-report, behavioral, physiologic, and neuroimaging targets. These targets will be identified in Phase 1, and appropriate IRB amendments will be submitted as the targets are identified.
3. Measures to be added to Concurrent Study C beginning in January 2016 are as follows:
    a. *The Edinburgh Handedness Inventory* (EHI)[1] is used to document the degree of right handedness. A score of >40 is a reliable indicator of right handedness. Participants must be right handed for admission into fMRI study. This will be completed at screening visit in subjects otherwise eligible for fMRI experiment. (3 min)
    b. *Urine or Saliva toxicology:* We will conduct urine or saliva toxicology screens for alcohol and illicit drugs at screening visit and before each MRI session. (5 min)
    c. *Urine pregnancy screen:* We will conduct a urine pregnancy screen at screening visit and before each MRI session. (2 min)
    d. *Body Mass Index:* Weight and height will be measured during screening visit and BMI will be calculated. (2 min)
    e. *Stop-Signal Task (SST)*[2] The SST is a two-choice reaction time task in which participants are required to respond to one or more go stimuli. At irregular intervals and unpredictably for the participants, a stop signal is presented.[3] Following this stop signal, participants are required to attempt to inhibit their response to the go signal. We will use two versions of the SST. The first (original SST) is similar to previous versions of the task used in neuroimaging studies. A second version (controlled SST) was designed to control for the attentional confound inherent in the original version. In both versions, subjects are presented with an initial fixation cross for 350 ms. This is followed by a go signal lasting 1,400 ms in the form of a left- or right-pointing arrow in the direction of the required response. Finger presses are made with the index finger of each hand. Unpredictably, on 20% of the trials, a red circle (the stop signal) appears above the location of the go stimulus. This stop signal indicates the need to attempt to inhibit the button press. The delay between the presentation of the go and stop signals is termed the stop signal delay (SSD). The ability to stop a response is a function of the length of the SSD. The longer the SSD, the more difficult it is to stop. The SSD will be varied from trial to trial using a staircase procedure that converges subjects toward an overall performance of 50% for each run. In the controlled version of the SST, a continue signal is introduced in the form of a green circle presented below the go signal. Subjects are instructed that, on some trials, this continue signal will appear unpredictably, but this should not alter their response, i.e., the initiated response to the go signal should be completed. The continue signal occurs on the same number of trials (20%) as the stop signal and with the same timing as the previous stop signal. The duration of both stop and continue cues is 1,400 msec minus the current SSD. Continue trials thus provide a control condition for the attentional processing associated with the presentation of an unexpected

perceptual event in a context in which response inhibition is not required. (10 min)

f. *Barratt Impulsiveness Scale* (BIS-11)[4] is a 30-item scale of impulsiveness (rated on 4-point scales from 1-rarely/never to 4-almost always/always). The BIS-11 provides three sub-scores: attentional impulsivity (inability to focus on the task at hand and having intrusive, racing thoughts), motor impulsivity (acting without thinking and lacking perseverance), and non-planning impulsivity (inability to plan or think carefully about or do challenging tasks). It also provides a total impulsivity score that is reported to be an internally consistent measure of impulsiveness with clinical utility for differentiating normal individuals from psychiatric patients, substance abusers, and prison inmates. (4 min)

g. *Behavioral Inhibition System/Behavioral Activation System* (BIS/BAS)[5] is a 24-item questionnaire rated on a 4-point scale (strong agreement – strong disagreement) measuring several domains of impulsivity based on Gray's model of personality. In Gray's view, there are two basic brain systems that control behavior and emotions: the aversive system or behavioral inhibition system (BIS) and the appetitive system or behavioral approach system (BAS). BIS is activated by conditioned stimuli associated with punishment or the termination of reward. BAS is activated by stimuli associated with reward or termination of punishment in order to guide the organism to appetitive stimuli. Gray's personality theory is based on the principle that individual differences in personality reflect the variation in sensitivity toward stimuli associated with negative and positive reinforcement, respectively BIS and BAS. The BIS/BAS scales consist of 20 items that can be allocated to two primary scales: the Behavioral Inhibition System scale (BIS; 7 items) and the Behavioral Approach System scale (BAS; 13 items). The BAS can be divided into 3 subscales: fun seeking (BAS fun), reward responsiveness (BAS reward), and drive (BAS drive). Drug addicts have higher scores on BAS drive, BAS fun, and BAS total score than controls[6]. (4 min)

h. *MRI Neuroimaging Experiments* will be conducted at week 0 and/or weeks 8-9 sessions. Participants enrolled into the neuroimaging experiment may undergo fMRI brain imaging sessions. These sessions may include resting state functional connectivity,[7] two fMRI-compatible Stop-Signal Tasks[2,8] for response inhibition, as well as fMRI tasks measuring self-compassion[9] and trait non-judgment of self.[10] Total duration is approximately 60 minutes.

i. *Electronic Medication Adherence Monitoring* will be measured continuously after screening visit for the add-on pilot study using electronic medication bottle caps. Participants taking daily medication for chronic illness will be included. Medication adherence is calculated as the percent of days during which medication was taken as prescribed. These devices simply replace the pill bottle caps of study participants' normal medication bottles. Participants will be trained in the use of the devices, which should take no longer than 5-10 minutes. The rationale for this assessment is that medication use for chronic illness has large impacts on outcome, and directly assessed measures are more accurate than self-report.[11] By embedding a CPU within a cap designed to fit a standard medication vial, the cap records each time the vial is opened to take a tablet or capsule. The cap is reusable at the time of refilling. Patients will be asked to bring in all current medication bottles to each study visit. At

the time of each study visit, the adherence data are downloaded via a RFID desktop Reader. The Software can display the information immediately on a PC via an intuitive interface. No patient information is stored on the devices, ensuring confidentiality. Download session may take 15 minutes, but can be completed while participants are completing self-report measures.

b. The NIH grant would somewhat expand the scope of the MBHT intervention, as follows:
   i. Addition of an active control group. This control group will be Mindfulness-Based Stress Reduction (MBSR), which is time- and attention-matched with MBHT. MBHT is based on MBSR, but MBHT customizes MBSR to hypertension and hypertension risk factors. This control group is not expected to be added until approximately 2018. MBSR will be performed by certified instructors. MBSR teacher certification is fairly extensive, and accreditation occurs through the University of Massachusetts Medical School Center for Mindfulness in Medicine, Health Care and Society. MBSR will consist of nine 2.5-hour weekly group sessions and an 8-hour one-day session. MBSR sessions contain instruction and practice in mindfulness meditation, and conversations about stress and coping. Students learn a range of mindfulness skills including body scan exercises, meditation and yoga. Homework consists of practicing skills for ≥45 min/day, 6 days/week. The MBSR classes will be composed only of study participants. The rationale for adding this control group is that MBSR already exists in hundreds of medical centers around the USA and worldwide. If MBHT is not more effective than an existing manualized, standardized, general mindfulness-based intervention such as MBSR, it is not needed.
   ii. Addition of electronically-measured medication adherence monitoring: measured continuously after randomization using electronic medication bottle caps. Participants taking medication to treat hypertension will qualify. Medication adherence is calculated as the percent of days during which medication was taken as prescribed. These devices simply replace the pill bottle caps of study participants' normal medication bottles. Participants will be trained in the use of the devices, which should take no longer than 5-10 minutes. The rationale for this assessment is that antihypertensive medication use has large impacts on blood pressure, and directly assessed measures are more accurate than self-report.
   iii. Addition of actigraphy monitoring devices. Participants will be asked to wear actigraphy monitoring devices at baseline and follow-up assessments for one week duration each time. These devices provide direct assessments of physical activity. The rationale for this assessment is that physical activity influences blood pressure, and directly assessed physical activity is more accurate than self-report.
   iv. Fidelity of MBSR and MBHT treatment: We will assess participants' perceptions of provider warmth and credibility using brief measures based on the validated Working Alliance Inventory,[12] and Empathy Scale.[13] These have been added to the assessments at Weeks 4 and 8 of the intervention. Please see **Appendix 2** for the questionnaires. The rationale for these assessments is that it is important to evaluate fidelity of the intervention, and these are standard measures of instructor effectiveness.
   v. Participants' mindfulness practice diaries will assess regularity of meditation and mindfulness practices. Please see **Appendix 3** for the diaries. The rationale for these assessments is that it has been shown that those who perform greater amounts of mindfulness practices outside of the classroom tend to have greater effects on mental health outcomes. The same may be true of blood pressure.
   vi. Focus group assessments of experts (including cardiologists, epidemiologists, mindfulness experts, mindfulness intervention instructors) will occur prior to, and following pilot testing of MBHT in participants. The rationale for doing these focus groups is to maximize MBHT efficacy, drawing on the knowledge of experts in relevant fields.

Adaptions may include, for example, types of specific mindfulness exercises, sequence in which modules are introduced, and relative emphasis on different types of modules (e.g. mindfulness practices customized to prehypertension/hypertension risk factors vs. general mindfulness practices). Inclusion criteria for the focus groups will be the experts being one of the professions listed above. Questions will include topics such as those shown below.

1. What about this intervention do think is most valuable, and has the greatest likelihood of influencing blood pressure, and determinants of blood pressure such as physical activity, diet and antihypertensive medication use?
2. Are there any elements of the intervention you feel could be pruned out, as they may be unlikely to have an effect on blood pressure and determinants of blood pressure? If so, what are they? Why do you think they would not have an effect?
3. All interventions can be improved. How do you think this intervention could be improved?

vii. Focus groups of participants undergoing the MBHT behavioral intervention will take place following the final week of class. The rationale of doing these focus groups is to better adapt MBHT to the unique needs of this population, and maximize intervention efficacy. Adaptions may include, for example, types of specific mindfulness exercises, sequence in which modules are introduced, relative emphasis on different types of modules (e.g. mindfulness practices customized to prehypertension/hypertension risk factors vs. general mindfulness practices), and class duration. Inclusion criteria for the focus groups will be being study participants. Questions will include topics such as those shown below.

1. Think back to the beginning of the course. Have you noticed some changes in yourself or your life since then? If yes, what have you noticed?
2. Can use card sort that will be anonymous – e.g. cards showing different modules. “Put cards for modules you really liked in one pile, modules you didn’t like in another pile, and modules you thought were OK but not great in a 3rd pile. Then have buckets for people to put their cards in for “liked module” “didn’t like module” or “module was OK”.
3. What did you most like about this course? What was most helpful?
4. We want to make this intervention better. You have been through it once. How do you think we can make it better?”
5. Take a vote – raise your hand if you think the session should be 2 h, 2.5 h; same for 3 hours. Same for retreat day – 5 h, 6 h, 7 h, 8 h.
6. Every instructor can improve. How can this instructor improve?

3.) State the reason (justification) for the requested amendment:

Please see justifications in Section 2 above.

4.) What is your assessment of how the changes will affect the overall risk/benefit ratio of the study and the willingness of individuals to participate?

These assessments should add some participant burden, primarily time associated with questionnaire completion, training and use of medication adherence devices and actigraphy, neuroimaging, and partaking in the focus group.

Potential benefits to participants include that completing the meditation diaries in the MBHT study may increase intervention effectiveness, as participants may feel more committed to complete the mindfulness exercises because their “homework” is being checked and followed up on. A participant in the most recent cycle of the MBHT intervention shared with me that she felt she would have done more of the assigned home

mindfulness practices if she was asked to fill out such a diary. In addition, MBHT participants receive feedback on their health pre- *vs*. post intervention. The direct assessments of physical activity and medication adherence will provide more accurate and higher quality feedback to participants.

I do not expect these changes to influence participation or risk/benefit ratio in meaningful ways.

5.) Does the requested amendment require new documents or changes to the approved consent form or other documents?

Yes. Please see **Appendices 2-4.** We have not added the MBHT control group to the consent form at this time, as it will not be offered until approximately 2017. The consent form will be modified accordingly, prior to including this control group.

References

1. Oldfield RC. The assessment and analysis of handedness: the Edinburgh inventory. *Neuropsychologia.* 1971;9(1):97-113.
2. Sharp DJ, Bonnelle V, De Boissezon X, et al. Distinct frontal systems for response inhibition, attentional capture, and error processing. *Proc Natl Acad Sci U S A.*107(13):6106-6111.
3. Aron AR, Fletcher PC, Bullmore ET, Sahakian BJ, Robbins TW. Stop-signal inhibition disrupted by damage to right inferior frontal gyrus in humans. *Nat Neurosci.* 2003;6(2):115-116.
4. Patton JH, Stanford MS, Barratt ES. Factor structure of the Barratt impulsiveness scale. *J Clin Psychol.* 1995;51(6):768-774.
5. Carver CS. Negative affects deriving from the behavioral approach system. *Emotion.* 2004;4(1):3-22.
6. Franken IH, Muris P, Georgieva I. Gray's model of personality and addiction. *Addict Behav.* 2006;31(3):399-403.
7. Cordes D, Haughton VM, Arfanakis K, et al. Mapping functionally related regions of brain with functional connectivity MR imaging. *AJNR Am J Neuroradiol.* 2000;21(9):1636-1644.
8. Aron AR, Poldrack RA. Cortical and subcortical contributions to Stop signal response inhibition: role of the subthalamic nucleus. *J Neurosci.* 2006;26(9):2424-2433.
9. Simon-Thomas ER, Godzik J, Castle E, et al. An fMRI study of caring vs self-focus during induced compassion and pride. *Soc Cogn Affect Neurosci.* 2012;7(6):635-648.
10. Longe O, Maratos FA, Gilbert P, et al. Having a word with yourself: neural correlates of self-criticism and self-reassurance. *Neuroimage.* 2010;49(2):1849-1856.
11. Wiens MO, MacLeod S, Musiime V, et al. Adherence to antiretroviral therapy in HIV-positive adolescents in Uganda assessed by multiple methods: a prospective cohort study. *Paediatric drugs.* 2012;14(5):331-335.
12. Horvath AO, Greenberg LS. Development and Validation of the Working Alliance Inventory. *J Couns Psychol.* 1989;36(2):223-233.
13. Burns DD, Auerbach A. Therapeutic empathy in cognitive-behavioural therapy: does it really make a difference? In: Salkovskis P, ed. *Frontiers of Cognitive Therapy*. New York: Guilford; 1996.

APPENDIX 1

IRB Approval Documents for Concurrent Studies

BROWN

Research Protections Office
Box 1986
Providence, RI 02912
Tel: (401) 863-3050
Fax: (401) 863-7292

Memorandum

To: Willoughby Britton, Box G-BH
From: Research Protections Office
Date: April 17, 2015
RE: Protocol Entitled: Dismantling Mindfulness: Contributions of Attention vs. Acceptance (#1105000399).
Continuing Review #4

The above referenced progress report received a full board review and approval by the Brown University IRB on April 16, 2015. Continuation of this study is approved through May 16, 2016.

Should the research continue, the next progress report must be submitted two months prior to the expiration date for review and approval.

Any amendments or changes to the research protocol including changes to the consent documents and questionnaires/surveys must be submitted to the IRB for review and approval prior to implementation. Also, any change in the status of participants to any of the vulnerable populations (as identified in 45 CFR 46) including, pregnant women, prisoners, or those with diminished capacity, requires IRB review and approval prior to their continuing in the study.

The IRB anticipates that investigators will employ recruitment procedures that allow for the equitable recruitment of women and minorities into research studies.

Note: All research staff must successfully complete the Brown University Education Program in the Protection of Human Research Participants (CITI at http://www.citiprogram.org) prior to beginning work on the project.

*** For Your Information ***

The next deadline for submission of new protocols, amendments to protocols, and annual progress reports requiring full board review is April 30, 2015.

All pertinent federal and University policies and guidelines related to the involvement of participants in research can be obtained through the Research Protections Office (RPO) at http://www.brown.edu/research/institutional-review-board-irb. This includes the *Belmont Report*, 45 CFR 46, and the Brown University Federal Wide Assurance #00004460.

Partners Human Research Committee
116 Huntington Avenue, Suite 1002
Boston, MA 02116
Tel: (617) 424-4100
Fax: (617) 424-4199

Continuing Review: Notification of IRB Approval/Activation
Protocol #: 2013P001153/MGH

Date: June 17, 2015

To: Sara W Lazar, Ph.D
MGH
Psychiatry

From: Partners Human Research Committee
116 Huntington Avenue, Suite 1002
Boston, MA 02116

Title of Protocol:	Psychological mechanisms of change in a mind-body training program
Version/Number:	v2
Version Date:	6/12/2015
Sponsor/Funding Support:	NIH-NCCAM National Center for Complementary and Alternative Medicine
Study Population:	Adults
Consent/Authorization:	Required (Mild deception with appropriate debriefing)
Documentation of Consent:	Written
Informed Consent From:	Adult Subject
Informed Consent By:	Other Study Staff (PS page 8 (study coordinator or study staff))
IRB Continuing Review #:	3
IRB Review Type:	Expedited
Expedited Category/ies:	(7)
IRB Approval Date:	6/17/2015
Approval Activation Date:	6/17/2015
IRB Expiration Date:	6/24/2016

This project has been reviewed by MGH IRB . During the review of this project, the IRB specifically considered (i) the risks and anticipated benefits, if any, to subjects; (ii) the selection of subjects; (iii) the procedures for obtaining and documenting informed consent; (iv) the safety of subjects; and (v) the privacy of subjects and confidentiality of the data.

Please note that if an IRB member had a conflict of interest with regard to the review of this project, consistent with IRB policies and procedures, the member was required to leave the room during the discussion and vote on this project except to provide information requested by the IRB.

The following protocol documents have been approved and supporting documents noted by

PARTNERS HEALTHCARE
FOUNDED BY BRIGHAM AND WOMEN'S HOSPITAL AND MASSACHUSETTS GENERAL HOSPITAL

Partners Human Research Committee
116 Huntington Avenue, Suite 1002
Boston, MA 02116
Tel: (617) 424-4100
Fax: (617) 424-4199

the IRB:
Protocol summary (v. 06/12/2015)
Detailed protocol (v. 06/12/2015)
Consent form
Advertisements (2)
Flyer
Recruitment letter
Instruments/ questionnaires (25)
Diaries
Manual of operations
Debriefing script
Data collection form

As Principal Investigator, you are responsible for ensuring that this project is conducted in compliance with all applicable federal, state and local laws and regulations, institutional policies, and requirements of the IRB, which include, but are not limited to, the following:

1. Submission of any and all proposed changes to this project (e.g., protocol, recruitment materials, consent form, status of the study, etc.) to the IRB for review and approval prior to initiation of the change(s), except where necessary to eliminate apparent immediate hazards to the subject(s). Changes made to eliminate apparent immediate hazards to subjects must be reported to the IRB as an unanticipated problem.
2. Submission of continuing review submissions for re-approval of the project prior to expiration of IRB approval and a final continuing review submission when the project has been completed.
3. Submission of any and all unanticipated problems, including adverse event(s) in accordance with the IRB's policy on reporting unanticipated problems including adverse events.
4. Obtaining informed consent from subjects or their legally authorized representative prior to initiation of research procedures when and as required by the IRB and, when applicable, documenting informed consent using the current IRB approved consent form(s)with the IRB-approval stamp in the document footer.
5. Informing all investigators and study staff listed on the project of changes and unanticipated problems, including adverse events, involving risks to subjects or others.
6. When investigator financial disclosure forms are required, updating your financial interests in Insight and for informing all site responsible investigators, co-investigators and any other members of the study staff identified by you as being responsible for the design, conduct, or reporting of this research study of their obligation to update their financial interest disclosures in Insight if (a) they have acquired new financial interests related to the study and/or (b) any of their previously reported financial interests related to the study have changed.

The IRB has the authority to terminate projects that are not in compliance with these requirements.

Questions related to this project may be directed to Ednice Depina-Monteiro, EEMONTEIRO@PARTNERS.ORG, 617-424-4144.

CC: Thomas Dylan Calahan, MGH - Psychiatry, Non-Study Staff

FOUNDED BY BRIGHAM AND WOMEN'S HOSPITAL
AND MASSACHUSETTS GENERAL HOSPITAL

Partners Human Research Committee
116 Huntington Avenue, Suite 1002
Boston, MA 02116
Tel: (617) 424-4100
Fax: (617) 424-4199

Thomas Dylan Calahan, MGH - Psychiatry, Non-Study Staff

Cambridge Health Alliance

NOTICE OF IRB APPROVAL – EXPEDITED NEW STUDY

October 15, 2014

Zev Schuman-Olivier, MD
Deptment of Psychiatry
Cambridge Health Alliance
26 Central Street
Somerville, MA 02143

Dear Dr. Schuman-Olivier,

The protocol titled, "**Integrating Mindfulness into the Patient-Centered Medical home (MINDFUL-PC)**" meets federal guidelines for a minimal risk study. Therefore, it was approved under the expedited review process (45 CFR 46.110). This is notification that I have approved the study and wish to communicate the following:

**Type of Review**	:	**Expedited Review**
**Action Taken**	:	**Approved**
**Date Approved**	:	**October 9, 2014**
**Next Review Date**	:	**October 8, 2015**
**IRB #**	:	**CHA-IRB-1002/08/14**
**Protocol Version**	:	**v19 – September 29, 2014**
**Patient ICF version**	:	**v12 – September 19, 2014**
**PCP ICF Version**	:	**v11 – September 19, 2014**

The convened IRB will be notified of the approval; the research may begin immediately. Please note the following:

- In accordance with 45 CFR 46.117[c][2] the IRB granted a waiver of documentation of consent as it relates to the portion of the study that is conducted electronically : the research presents no more than minimal risk and involves no procedures for which written consent is normally required.

- Previously identified stipulations have been addressed and resolved. Requested documentation was received from the study team 10/06/14; it was reviewed and the study was approved 10/09/14.

- Any and all changes to this project (*e.g.*, protocol, recruitment materials, consent form, study completion) **must be** submitted to the IRB for review and approval prior to initiation of the change(s). **Only** in circumstances where changes are necessary to eliminate apparent immediate hazards to the subject(s), changes can be implemented prior to IRB approval and submitted to the IRB subsequently for review and approval (45 CFR 46.103).

- Any and all unanticipated problems involving risks to human subjects or others that occur during the course of this project must be reported in accordance with the IRB's policy on adverse event reporting.

- Any non-compliance with IRB policies or regulations governing human subject protections must be reported to the IRB immediately.

- Use only IRB approved copies of the consent form(s), questionnaire(s), letter(s), advertisement(s) etc., in your research. Do not use expired consent forms.

- For funded studies: Research must not begin without authorization from the Office for Sponsored Research.

- As a reminder, the HIPAA Privacy and Security Rule apply to this study. If you create, receive, store, or transmit electronic PHI you must meet institutional Security Rule standards.

Federal regulations (45 CFR 46.109(e)) require IRBs to conduct continuing review of research at intervals appropriate to the degree of risk of the study, but not less than once a year. Non-compliance with continuing review procedures could result in a suspension of research activities.

Please direct questions, correspondence, and forms (*e.g.,* continuing reviews, amendments, adverse events, and safety reports) to Ida Rego, irego@challiance.org, 617-499-8302.

Sincerely,

L. Givon MD

Lior Givon, MD, PhD
Chair, Institutional Review Board

THIS LETTER MUST BE RETAINED WITH YOUR FILES.

University of Massachusetts
UMASS. Medical School

August 12, 2014

Carl Fulwiler, MD, PhD
Psychiatry

Dear Dr. Fulwiler:

The IRB reviewed the following:

Type of Submission:	Modification
Review Type:	Non-Committee
Project Title:	Keeping weight off: Brain Changes Associated with Healthy Behaviors
Investigator:	Carl Fulwiler, MD, PhD
IRB ID:	H00001808_4
Funding Agency:	NATIONAL INSTITUTES OF HEALTH
Grant Title:	Mind your health: Developing a neural marker for mindfulness as a pathway to wellness
Grant ID:	1 R34 AT006963-01A1
IND or IDE:	None
IRB Review Date:	8/8/2014
Documents Reviewed:	UMMS HRP-503 - Fulwiler R34 V.4 6_29_14.docx<br>Consent _Fulwiler R34_V.2_3_31_14

The IRB approved the modification effective on 8/8/2014.

In conducting this research, you are required to follow the requirements listed in the INVESTIGATOR MANUAL (HRP-103).

Sincerely,

Allison Blodgett, PhD
IRB Assistant Manager

cc: Lazaridou, Asimina

APPENDIX 2

Working Alliance Inventory (WA)

Empathy Scale (EA)

Administered to participants at intervention weeks 4 and 8.

Please answer the questions below about your experiences with the group leader.

	0<br>strongly disagree	1<br>moderately disagree	2<br>somewhat disagree	3<br>no opinion	4<br>slightly agree	5<br>moderately agree	6<br>strongly agree
ES1_01. I felt that I could trust the group leader during today's session.	☐	☐	☐	☐	☐	☐	☐
ES1_02. The group leader felt I was worthwhile.	☐	☐	☐	☐	☐	☐	☐
ES1_03. The group leader was friendly and warm towards me.	☐	☐	☐	☐	☐	☐	☐
ES1_04. The group leader understood what I said during today's session.	☐	☐	☐	☐	☐	☐	☐
ES1_05. The group leader was sympathetic and concerned about me.	☐	☐	☐	☐	☐	☐	☐
ES1_06. Sometimes the group leader did not seem to be completely genuine.	☐	☐	☐	☐	☐	☐	☐
ES1_07. The group leader pretended to like me more than he/she really does.	☐	☐	☐	☐	☐	☐	☐
ES1_08. The group leader did not always seem to care about me.	☐	☐	☐	☐	☐	☐	☐
ES1_09. The group leader did not always understand the way I felt inside.	☐	☐	☐	☐	☐	☐	☐
ES1_10. The group leader acted condescending and talked down to me.	☐	☐	☐	☐	☐	☐	☐

	Not At All True						Very True
	1	2	3	4	5	6	7
WA1_01. I feel uncomfortable with the group leader.	□	□	□	□	□	□	□
WA1_02. What I am doing in the mindfulness intervention gives me new ways of looking at my problem.	□	□	□	□	□	□	□
WA1_03. The group leader and I understand each other.	□	□	□	□	□	□	□
WA1_04. I believe the group leader likes me.	□	□	□	□	□	□	□
WA1_05. I believe the group leader is genuinely concerned for my welfare.	□	□	□	□	□	□	□
WA1_06. The group leader and I respect each other.	□	□	□	□	□	□	□
WA1_07. I feel that the group leader is not totally honest about his/her feelings towards me.	□	□	□	□	□	□	□
WA1_08. I am confident in the group leader's ability to help me.	□	□	□	□	□	□	□
WA1_09. I feel that the group leader appreciates me.	□	□	□	□	□	□	□
WA1_10. The group leader and I trust one another.	□	□	□	□	□	□	□
WA1_11. My relationship with the group leader is very important to me.	□	□	□	□	□	□	□
WA1_12. I have the feeling that if I say or do the wrong things, the group leader will stop working with me.	□	□	□	□	□	□	□
WA1_13. I feel the group leader cares about me even when I do things that he/she does not approve of.	□	□	□	□	□	□	□

APPENDIX 3

Participants’ Mindfulness Practice Diaries

Daily Practice Record Form – MBHT Week 1 ID #:___________

Please record each time you practice. Please make a note of anything that comes up in the homework so that we can talk about it at the next meeting.

Day/ Date	1.) Body Scan	2.) Mindful Eating/Meals		3.) Comments
	# Minutes	Meal	# Minutes	
Mon Date:	W1_D1_1. # Minutes	W1_D1_2a. Meal	W1_D1_2b. # Minutes	W1_D1_3.
Tues Date:	W1_D2_1. # Minutes	W1_D2_2a. Meal	W1_D2_2b. # Minutes	W1_D2_3.
Wed Date:	W1_D3_1. # Minutes	W1_D3_2a. Meal	W1_D3_2b. # Minutes	W1_D3_3.
Thurs Date:	W1_D4_1. # Minutes	W1_D4_2a. Meal	W1_D4_2b. # Minutes	W1_D4_3.
Fri Date:	W1_D5_1. # Minutes	W1_D5_2a. Meal	W1_D5_2b. # Minutes	W1_D5_3.
Sat Date:	W1_D6_1. # Minutes	W1_D6_2a. Meal	W1_D6_2b. # Minutes	W1_D6_3.
Sun Date:	W1_D7_1. # Minutes	W1_D7_2a. Meal	W1_D7_2b. # Minutes	W1_D7_3.

Daily Practice Record Form – MBHT Week 2 ID #:___________

Please record each time you practice. Please make a note of anything that comes up in the homework so that we can talk about it at the next meeting.

Day/ Date	1.) Body Scan Meditation	2.) Breath Awareness Practice	3.) Mindful Activity		4.) Comments
	# Minutes	# Minutes	Activity	# Minutes	
**Mon Date:**	W2_D1_1. **# Minutes**	W2_D1_2. **# Minutes**	W2_D1_3a. **Activity**	W2_D1_3b. **# Minutes**	W2_D1_4.
**Tues Date:**	W2_D2_1. **# Minutes**	W2_D2_2. **# Minutes**	W2_D2_3a. **Activity**	W2_D2_3b. **# Minutes**	W2_D2_4.
**Wed Date:**	W2_D3_1. **# Minutes**	W2_D3_2. **# Minutes**	W2_D3_3a. **Activity**	W2_D3_3b. **# Minutes**	W2_D3_4.
**Thurs Date:**	W2_D4_1. **# Minutes**	W2_D4_2. **# Minutes**	W2_D4_3a. **Activity**	W2_D4_3b. **# Minutes**	W2_D4_4.
**Fri Date:**	W2_D5_1. **# Minutes**	W2_D5_2. **# Minutes**	W2_D5_3a. **Activity**	W2_D5_3b. **# Minutes**	W2_D5_4.
**Sat Date:**	W2_D6_1. **# Minutes**	W2_D6_2. **# Minutes**	W2_D6_3a. **Activity**	W2_D6_3b. **# Minutes**	W2_D6_4.
**Sun Date:**	W2_D7_1. **# Minutes**	W2_D7_2. **# Minutes**	W2_D7_3a. **Activity**	W2_D7_3b. **# Minutes**	W2_D7_4.

Daily Practice Record Form – MBHT Week 3 ID #:___________

Please record each time you practice. Please make a note of anything that comes up in the homework so that we can talk about it at the next meeting.

Day/ Date	1.) Body Scan	2.) Yoga	3.) Breath Awareness	4.) Physical Activity		5.) Comments
	# Minutes	# Minutes	# Minutes	Activity	# Minutes	
**Mon Date:**	W3_D1_1. **# Minutes**	W3_D1_2. **# Minutes**	W3_D1_2. **# Minutes**	W3_D1_3a. **Activity**	W3_D1_3b. **# Minutes**	W3_D1_4.
**Tues Date:**	W3_D2_1. **# Minutes**	W3_D2_2. **# Minutes**	W3_D2_2. **# Minutes**	W3_D2_3a. **Activity**	W3_D2_3b. **# Minutes**	W3_D2_4.
**Wed Date:**	W3_D3_1. **# Minutes**	W3_D3_2. **# Minutes**	W3_D3_2. **# Minutes**	W3_D3_3a. **Activity**	W3_D3_3b. **# Minutes**	W3_D3_4.
**Thurs Date:**	W3_D4_1. **# Minutes**	W3_D4_2. **# Minutes**	W3_D4_2. **# Minutes**	W3_D4_3a. **Activity**	W3_D4_3b. **# Minutes**	W3_D4_4.
**Fri Date:**	W3_D5_1. **# Minutes**	W3_D5_2. **# Minutes**	W3_D5_2. **# Minutes**	W3_D5_3a. **Activity**	W3_D5_3b. **# Minutes**	W3_D5_4.
**Sat Date:**	W3_D6_1. **# Minutes**	W3_D6_2. **# Minutes**	W3_D6_2. **# Minutes**	W3_D6_3a. **Activity**	W3_D6_3b. **# Minutes**	W3_D6_4.
**Sun Date:**	W3_D7_1. **# Minutes**	W3_D7_2. **# Minutes**	W3_D7_2. **# Minutes**	W3_D7_3a. **Activity**	W3_D7_3b. **# Minutes**	W3_D7_4.

Daily Practice Record Form – MBHT Week 4 ID #:___________

Please record each time you practice. Please make a note of anything that comes up in the homework so that we can talk about it at the next meeting.

Day/ Date	1.) Body Scan	2.) Yoga	3.) Sitting Meditation	4.) Physical Activity		5.) Comments
	# Minutes	# Minutes	# Minutes	Activity	# Minutes	
Mon Date:	W4_D1_1. # Minutes	W4_D1_2. # Minutes	W4_D1_3. # Minutes	W4_D1_4a. Activity	W4_D1_4b. # Minutes	W4_D1_5.
Tues Date:	W4_D2_1. # Minutes	W4_D2_2. # Minutes	W4_D2_3. # Minutes	W4_D2_4a. Activity	W4_D2_4b. # Minutes	W4_D2_5.
Wed Date:	W4_D3_1. # Minutes	W4_D3_2. # Minutes	W4_D3_3. # Minutes	W4_D3_4a. Activity	W4_D3_4b. # Minutes	W4_D3_5.
Thurs Date:	W4_D4_1. # Minutes	W4_D4_2. # Minutes	W4_D4_3. # Minutes	W4_D4_4a. Activity	W4_D4_4b. # Minutes	W4_D4_5.
Fri Date:	W4_D5_1. # Minutes	W4_D5_2. # Minutes	W4_D5_3. # Minutes	W4_D5_4a. Activity	W4_D5_4b. # Minutes	W4_D5_5.
Sat Date:	W4_D6_1. # Minutes	W4_D6_2. # Minutes	W4_D6_3. # Minutes	W4_D6_4a. Activity	W4_D6_4b. # Minutes	W4_D6_5.
Sun Date:	W4_D7_1. # Minutes	W4_D7_2. # Minutes	W4_D7_3. # Minutes	W4_D7_4a. Activity	W4_D7_4b. # Minutes	W4_D7_5.

Daily Practice Record Form – MBHT Week 5

ID #:___________

Please record each time you practice. Please make a note of anything that comes up in the homework so that we can talk about it at the next meeting.

Day/ Date	1.) Body Scan	2.) Yoga	3.) Sitting Meditation	4.) S-T-O-P Practice	5.) Comments
	# Minutes	# Minutes	# Minutes	# Minutes	
**Mon Date:**	W5_D1_1. **# Minutes**	W5_D1_2. **# Minutes**	W5_D1_3. **# Minutes**	W5_D1_4. **# Minutes**	W5_D1_5.
**Tues Date:**	W5_D2_1. **# Minutes**	W5_D2_2. **# Minutes**	W5_D2_3. **# Minutes**	W5_D2_4. **# Minutes**	W5_D2_5.
**Wed Date:**	W5_D3_1. **# Minutes**	W5_D3_2. **# Minutes**	W5_D3_3. **# Minutes**	W5_D3_4. **# Minutes**	W5_D3_5.
**Thurs Date:**	W5_D4_1. **# Minutes**	W5_D4_2. **# Minutes**	W5_D4_3. **# Minutes**	W5_D4_4. **# Minutes**	W5_D4_5.
**Fri Date:**	W5_D5_1. **# Minutes**	W5_D5_2. **# Minutes**	W5_D5_3. **# Minutes**	W5_D5_4. **# Minutes**	W5_D5_5.
**Sat Date:**	W5_D6_1. **# Minutes**	W5_D6_2. **# Minutes**	W5_D6_3. **# Minutes**	W5_D6_4. **# Minutes**	W5_D6_5.
**Sun Date:**	W5_D7_1. **# Minutes**	W5_D7_2. **# Minutes**	W5_D7_3. **# Minutes**	W5_D7_4. **# Minutes**	W5_D7_5.

Daily Practice Record Form – MBHT Week 6 ID #:___________

Please record each time you practice. Please make a note of anything that comes up in the homework so that we can talk about it at the next meeting.

Day/ Date	1.) Body Scan	2.) Yoga	3.) Sitting Meditation	4.) Comments
	# Minutes	# Minutes	# Minutes	
**Mon Date:**	W6_D1_1. **# Minutes**	W6_D1_2. **# Minutes**	W6_D1_3. **# Minutes**	W6_D1_4.
**Tues Date:**	W6_D2_1. **# Minutes**	W6_D2_2. **# Minutes**	W6_D2_3. **# Minutes**	W6_D2_4.
**Wed Date:**	W6_D3_1. **# Minutes**	W6_D3_2. **# Minutes**	W6_D3_3. **# Minutes**	W6_D3_4.
**Thurs Date:**	W6_D4_1. **# Minutes**	W6_D4_2. **# Minutes**	W6_D4_3. **# Minutes**	W6_D4_4.
**Fri Date:**	W6_D5_1. **# Minutes**	W6_D5_2. **# Minutes**	W6_D5_3. **# Minutes**	W6_D5_4.
**Sat Date:**	W6_D6_1. **# Minutes**	W6_D6_2. **# Minutes**	W6_D6_3. **# Minutes**	W6_D6_4.
**Sun Date:**	W6_D7_1. **# Minutes**	W6_D7_2. **# Minutes**	W6_D7_3. **# Minutes**	W6_D7_4.

Daily Practice Record Form – MBHT Week 7

ID #:___________

Please record each time you practice. Please make a note of anything that comes up in the homework so that we can talk about it at the next meeting.

Day/ Date	1.) Mindfulness Practice		2.) Nourishing Activity		3.) Refraining-From-Poisoning Activity		4.) Accepting-What-I-Can't-Control Activity		5.) Comments
	Practice Type(s)	# Minutes	Activity	# Minutes	Activity	# Minutes	Activity	# Minutes	
Mon Date:	W7_D1_1a. Type(s)	W7_D1_1b. # Min	W7_D1_2a. Activity	W7_D1_2b. # Min	W7_D1_3a. Activity	W7_D1_3b. # Min	W7_D1_4a. Activity	W7_D1_4b. # Min	W7_D1_5.
Tues Date:	W7_D2_1a. Type(s)	W7_D2_1b. # Min	W7_D2_2a. Activity	W7_D2_2b. # Min	W7_D2_3a. Activity	W7_D2_3b. # Min	W7_D2_4a. Activity	W7_D2_4b. # Min	W7_D2_5.
Wed Date:	W7_D3_1a. Type(s)	W7_D3_1b. # Min	W7_D3_2a. Activity	W7_D3_2b. # Min	W7_D3_3a. Activity	W7_D3_3b. # Min	W7_D3_4a. Activity	W7_D3_4b. # Min	W7_D3_5.

Day/ Date	1.) Mindfulness Practice		2.) Nourishing Activity		3.) Refraining-From-Poisoning Activity		4.) Accepting-What-I-Can't-Control Activity		5.) Comments
	**Practice Type(s)**	**# Minutes**	**Activity**	**# Minutes**	**Activity**	**# Minutes**	**Activity**	**# Minutes**	
**Thurs Date:**	W7_D4_1a. **Type(s)**	W7_D4_1b. **# Min**	W7_D4_2a. **Activity**	W7_D4_2b. **# Min**	W7_D4_3a. **Activity**	W7_D4_3b. **# Min**	W7_D4_4a. **Activity**	W7_D4_4b. **# Min**	W7_D4_5.
**Fri Date:**	W7_D5_1a. **Type(s)**	W7_D5_1b. **# Min**	W7_D5_2a. **Activity**	W7_D5_2b. **# Min**	W7_D5_3a. **Activity**	W7_D5_3b. **# Min**	W7_D5_4a. **Activity**	W7_D5_4b. **# Min**	W7_D5_5.
**Sat Date:**	W7_D6_1a. **Type(s)**	W7_D6_1b. **# Min**	W7_D6_2a. **Activity**	W7_D6_2b. **# Min**	W7_D6_3a. **Activity**	W7_D6_3b. **# Min**	W7_D6_4a. **Activity**	W7_D6_4b. **# Min**	W7_D6_5.
**Sun Date:**	W7_D7_1a. **Type(s)**	W7_D7_1b. **# Min**	W7_D7_2a. **Activity**	W7_D7_2b. **# Min**	W7_D7_3a. **Activity**	W7_D7_3b. **# Min**	W7_D7_4a. **Activity**	W7_D7_4b. **# Min**	W7_D7_5.

W4_GO_7. Goal for This Week Related to Diet, Alcohol Consumption, Physical Activity or Stress Reaction/Response:

____

____

____

W4_GO_8. How Goal Will Be Measured:

____

____

____

**Day/Date**	**Event related to goal**	**How did your body feel, in detail, before, during and after this experience?**	**What moods, feelings, and thoughts were there before, during and after this event?**	**How did it contribute, or not, to achieving the goal?**	**What thoughts are in your mind now as you write about this event?**
**Mon**<br>**Date:**	W4_GO_D1_1.	W4_GO_D1_2a.<br>**Before:**<br><br>W4_GO_D1_2b.<br>**During:**<br><br>W4_GO_D1_2c.<br>**After:**	W4_GO_D1_3a.<br>**Before:**<br><br>W4_GO_D1_3b.<br>**During:**<br><br>W4_GO_D1_3c.<br>**After:**	W4_GO_D1_4.	W4_GO_D1_5.

Day/Date	Event related to goal	How did your body feel, in detail, before, during and after this experience?	What moods, feelings, and thoughts were there before, during and after this event?	How did it contribute, or not, to achieving the goal?	What thoughts are in your mind now as you write about this event?
**Tues**<br>**Date:**	W4_GO_D2_1.	W4_GO_D2_1.	W4_GO_D2_2a.<br>**Before:**<br><br>W4_GO_D2_2b.<br>**During:**<br><br>W4_GO_D2_2c.<br>**After:**	W4_GO_D2_3a.<br>**Before:**<br><br>W4_GO_D2_3b.<br>**During:**<br><br>W4_GO_D2_3c.<br>**After:**	W4_GO_D2_4.
**Wed**<br>**Date:**	W4_GO_D3_1.	W4_GO_D3_1.	W4_GO_D3_2a.<br>**Before:**<br><br>W4_GO_D3_2b.<br>**During:**<br><br>W4_GO_D3_2c.<br>**After:**	W4_GO_D3_3a.<br>**Before:**<br><br>W4_GO_D3_3b.<br>**During:**<br><br>W4_GO_D3_3c.<br>**After:**	W4_GO_D3_4.

Day/Date	Event related to goal	How did your body feel, in detail, before, during and after this experience?	What moods, feelings, and thoughts were there before, during and after this event?	How did it contribute, or not, to achieving the goal?	What thoughts are in your mind now as you write about this event?
**Thurs**<br>**Date:**	W4_GO_D4_1.	W4_GO_D4_1.	W4_GO_D4_2a.<br>**Before:**<br><br>W4_GO_D4_2b.<br>**During:**<br><br>W4_GO_D4_2c.<br>**After:**	W4_GO_D4_3a.<br>**Before:**<br><br>W4_GO_D4_3b.<br>**During:**<br><br>W4_GO_D4_3c.<br>**After:**	W4_GO_D4_4.
**Fri**<br>**Date:**	W4_GO_D5_1.	W4_GO_D5_1.	W4_GO_D5_2a.<br>**Before:**<br><br>W4_GO_D5_2b.<br>**During:**<br><br>W4_GO_D5_2c.<br>**After:**	W4_GO_D5_3a.<br>**Before:**<br><br>W4_GO_D5_3b.<br>**During:**<br><br>W4_GO_D5_3c.<br>**After:**	W4_GO_D5_4.

Day/Date	Event related to goal	How did your body feel, in detail, before, during and after this experience?	What moods, feelings, and thoughts were there before, during and after this event?	How did it contribute, or not, to achieving the goal?	What thoughts are in your mind now as you write about this event?
**Sat**<br>**Date:**	W4_GO_D6_1.	W4_GO_D6_1.	W4_GO_D6_2a.<br>**Before:**<br><br>W4_GO_D6_2b.<br>**During:**<br><br>W4_GO_D6_2c.<br>**After:**	W4_GO_D6_3a.<br>**Before:**<br><br>W4_GO_D6_3b.<br>**During:**<br><br>W4_GO_D6_3c.<br>**After:**	W4_GO_D6_4.
**Sun**<br>**Date:**	W4_GO_D7_1.	W4_GO_D7_1.	W4_GO_D7_2a.<br>**Before:**<br><br>W4_GO_D7_2b.<br>**During:**<br><br>W4_GO_D7_2c.<br>**After:**	W4_GO_D7_3a.<br>**Before:**<br><br>W4_GO_D7_3b.<br>**During:**<br><br>W4_GO_D7_3c.<br>**After:**	W4_GO_D7_4.

ID#________

Week 4: Goal Related to Diet, Alcohol Consumption, Physical Activity, or Stress Reaction/Response

W4_GO_1. What is your goal related to diet, alcohol consumption, physical activity, or stress reaction/response for the week?

W4_GO_2. On a scale of 1-10, where 10 is high, how MOTIVATED are you to achieve this goal?

W4_GO_3. On a scale of 1-10, how CONFIDENT are you that you will achieve the goal?

W4_GO_4. What could you do that would bring your motivation or confidence a little higher?

W4_GO_5. What might make it difficult to achieve the goal this week, and if that happens, what will you do?

W4_GO_6. What is a way to measure this goal that resonates with you?

W5_GO_7. Goal for This Week Related to Diet, Alcohol Consumption, Physical Activity, Stress Reaction/Response, or Antihypertensive Medication Use:

____

____

____

W5_GO_8. How Goal Will Be Measured:

____

____

____

Day/Date	Event related to goal	How did your body feel, in detail, before, during and after this experience?	What moods, feelings, and thoughts were there before, during and after this event?	How did it contribute, or not, to achieving the goal?	What thoughts are in your mind now as you write about this event?
**Mon**<br>**Date:**	W5_GO_D1_1.	W5_GO_D1_2a.<br>**Before:**<br><br>W5_GO_D1_2b.<br>**During:**<br><br>W5_GO_D1_2c.<br>**After:**	W5_GO_D1_3a.<br>**Before:**<br><br>W5_GO_D1_3b.<br>**During:**<br><br>W5_GO_D1_3c.<br>**After:**	W5_GO_D1_4.	W5_GO_D1_5.

Day/Date	Event related to goal	How did your body feel, in detail, before, during and after this experience?	What moods, feelings, and thoughts were there before, during and after this event?	How did it contribute, or not, to achieving the goal?	What thoughts are in your mind now as you write about this event?
**Tues**<br>**Date:**	W5_GO_D2_1.	W5_GO_D2_1.	W5_GO_D2_2a.<br>**Before:**<br><br>W5_GO_D2_2b.<br>**During:**<br><br>W5_GO_D2_2c.<br>**After:**	W5_GO_D2_3a.<br>**Before:**<br><br>W5_GO_D2_3b.<br>**During:**<br><br>W5_GO_D2_3c.<br>**After:**	W5_GO_D2_4.
**Wed**<br>**Date:**	W5_GO_D3_1.	W5_GO_D3_1.	W5_GO_D3_2a.<br>**Before:**<br><br>W5_GO_D3_2b.<br>**During:**<br><br>W5_GO_D3_2c.<br>**After:**	W5_GO_D3_3a.<br>**Before:**<br><br>W5_GO_D3_3b.<br>**During:**<br><br>W5_GO_D3_3c.<br>**After:**	W5_GO_D3_4.

Day/Date	Event related to goal	How did your body feel, in detail, before, during and after this experience?	What moods, feelings, and thoughts were there before, during and after this event?	How did it contribute, or not, to achieving the goal?	What thoughts are in your mind now as you write about this event?
**Thurs**<br>**Date:**	W5_GO_D4_1.	W5_GO_D4_1.	W5_GO_D4_2a.<br>**Before:**<br><br>W5_GO_D4_2b.<br>**During:**<br><br>W5_GO_D4_2c.<br>**After:**	W5_GO_D4_3a.<br>**Before:**<br><br>W5_GO_D4_3b.<br>**During:**<br><br>W5_GO_D4_3c.<br>**After:**	W5_GO_D4_4.
**Fri**<br>**Date:**	W5_GO_D5_1.	W5_GO_D5_1.	W5_GO_D5_2a.<br>**Before:**<br><br>W5_GO_D5_2b.<br>**During:**<br><br>W5_GO_D5_2c.<br>**After:**	W5_GO_D5_3a.<br>**Before:**<br><br>W5_GO_D5_3b.<br>**During:**<br><br>W5_GO_D5_3c.<br>**After:**	W5_GO_D5_4.

Day/Date	Event related to goal	How did your body feel, in detail, before, during and after this experience?	What moods, feelings, and thoughts were there before, during and after this event?	How did it contribute, or not, to achieving the goal?	What thoughts are in your mind now as you write about this event?
**Sat**<br>**Date:**	W5_GO_D6_1.	W5_GO_D6_1.	W5_GO_D6_2a.<br>**Before:**<br><br>W5_GO_D6_2b.<br>**During:**<br><br>W5_GO_D6_2c.<br>**After:**	W5_GO_D6_3a.<br>**Before:**<br><br>W5_GO_D6_3b.<br>**During:**<br><br>W5_GO_D6_3c.<br>**After:**	W5_GO_D6_4.
**Sun**<br>**Date:**	W5_GO_D7_1.	W5_GO_D7_1.	W5_GO_D7_2a.<br>**Before:**<br><br>W5_GO_D7_2b.<br>**During:**<br><br>W5_GO_D7_2c.<br>**After:**	W5_GO_D7_3a.<br>**Before:**<br><br>W5_GO_D7_3b.<br>**During:**<br><br>W5_GO_D7_3c.<br>**After:**	W5_GO_D7_4.

ID#________

Week 5: Goal Related to Diet, Alcohol Consumption, Physical Activity, Stress Reaction/Response, or Antihypertensive Medication Use

W5_GO_1. What is your goal related to diet, alcohol consumption, physical activity, or stress reaction/response, or antihypertensive medication use for the week?

W5_GO_2. On a scale of 1-10, where 10 is high, how MOTIVATED are you to achieve this goal?

W5_GO_3. On a scale of 1-10, how CONFIDENT are you that you will achieve the goal?

W5_GO_4. What could you do that would bring your motivation or confidence a little higher?

W5_GO_5. What might make it difficult to achieve the goal this week, and if that happens, what will you do?

W5_GO_6. What is a way to measure this goal that resonates with you?

W6_GO_7. Goal for This Week Related to Diet, Alcohol Consumption, Physical Activity, Stress Reaction/Response, Antihypertensive Medication Use, or Social Support:

____

____

____

W6_GO_8. How Goal Will Be Measured:

____

____

____

**Day/Date**	**Event related to goal**	**How did your body feel, in detail, before, during and after this experience?**	**What moods, feelings, and thoughts were there before, during and after this event?**	**How did it contribute, or not, to achieving the goal?**	**What thoughts are in your mind now as you write about this event?**
**Mon**<br>**Date:**	W6_GO_D1_1.	W6_GO_D1_2a.<br>**Before:**<br><br>W6_GO_D1_2b.<br>**During:**<br><br>W6_GO_D1_2c.<br>**After:**	W6_GO_D1_3a.<br>**Before:**<br><br>W6_GO_D1_3b.<br>**During:**<br><br>W6_GO_D1_3c.<br>**After:**	W6_GO_D1_4.	W6_GO_D1_5.

Day/Date	Event related to goal	How did your body feel, in detail, before, during and after this experience?	What moods, feelings, and thoughts were there before, during and after this event?	How did it contribute, or not, to achieving the goal?	What thoughts are in your mind now as you write about this event?
**Tues**<br>**Date:**	W6_GO_D2_1.	W6_GO_D2_1.	W6_GO_D2_2a.<br>**Before:**<br><br>W6_GO_D2_2b.<br>**During:**<br><br>W6_GO_D2_2c.<br>**After:**	W6_GO_D2_3a.<br>**Before:**<br><br>W6_GO_D2_3b.<br>**During:**<br><br>W6_GO_D2_3c.<br>**After:**	W6_GO_D2_4.
**Wed**<br>**Date:**	W6_GO_D3_1.	W6_GO_D3_1.	W6_GO_D3_2a.<br>**Before:**<br><br>W6_GO_D3_2b.<br>**During:**<br><br>W6_GO_D3_2c.<br>**After:**	W6_GO_D3_3a.<br>**Before:**<br><br>W6_GO_D3_3b.<br>**During:**<br><br>W6_GO_D3_3c.<br>**After:**	W6_GO_D3_4.

Day/Date	Event related to goal	How did your body feel, in detail, before, during and after this experience?	What moods, feelings, and thoughts were there before, during and after this event?	How did it contribute, or not, to achieving the goal?	What thoughts are in your mind now as you write about this event?
**Thurs**<br>**Date:**	W6_GO_D4_1.	W6_GO_D4_1.	W6_GO_D4_2a.<br>**Before:**<br><br>W6_GO_D4_2b.<br>**During:**<br><br>W6_GO_D4_2c.<br>**After:**	W6_GO_D4_3a.<br>**Before:**<br><br>W6_GO_D4_3b.<br>**During:**<br><br>W6_GO_D4_3c.<br>**After:**	W6_GO_D4_4.
**Fri**<br>**Date:**	W6_GO_D5_1.	W6_GO_D5_1.	W6_GO_D5_2a.<br>**Before:**<br><br>W6_GO_D5_2b.<br>**During:**<br><br>W6_GO_D5_2c.<br>**After:**	W6_GO_D5_3a.<br>**Before:**<br><br>W6_GO_D5_3b.<br>**During:**<br><br>W6_GO_D5_3c.<br>**After:**	W6_GO_D5_4.

Day/Date	Event related to goal	How did your body feel, in detail, before, during and after this experience?	What moods, feelings, and thoughts were there before, during and after this event?	How did it contribute, or not, to achieving the goal?	What thoughts are in your mind now as you write about this event?
**Sat**<br>**Date:**	W6_GO_D6_1.	W6_GO_D6_1.	W6_GO_D6_2a.<br>**Before:**<br><br>W6_GO_D6_2b.<br>**During:**<br><br>W6_GO_D6_2c.<br>**After:**	W6_GO_D6_3a.<br>**Before:**<br><br>W6_GO_D6_3b.<br>**During:**<br><br>W6_GO_D6_3c.<br>**After:**	W6_GO_D6_4.
**Sun**<br>**Date:**	W6_GO_D7_1.	W6_GO_D7_1.	W6_GO_D7_2a.<br>**Before:**<br><br>W6_GO_D7_2b.<br>**During:**<br><br>W6_GO_D7_2c.<br>**After:**	W6_GO_D7_3a.<br>**Before:**<br><br>W6_GO_D7_3b.<br>**During:**<br><br>W6_GO_D7_3c.<br>**After:**	W6_GO_D7_4.

ID#________

Week 6: Goal Related to Diet, Alcohol Consumption, Physical Activity, Stress Reaction/Response, Antihypertensive Medication Use, or Social Support

W6_GO_1. What is your goal related to diet, alcohol consumption, physical activity, stress reaction/response, antihypertensive medication use, or social support for the week?

W6_GO_2. On a scale of 1-10, where 10 is high, how MOTIVATED are you to achieve this goal?

W6_GO_3. On a scale of 1-10, how CONFIDENT are you that you will achieve the goal?

W6_GO_4. What could you do that would bring your motivation or confidence a little higher?

W6_GO_5. What might make it difficult to achieve the goal this week, and if that happens, what will you do?

W6_GO_6. What is a way to measure this goal that resonates with you?

W7_GO_7. Goal for This Week Related to Diet, Alcohol Consumption, Physical Activity, Stress Reaction/Response, Antihypertensive Medication Use, or Social Support:

____

____

____

W7_GO_8. How Goal Will Be Measured:

____

____

____

**Day/Date**	**Event related to goal**	**How did your body feel, in detail, before, during and after this experience?**	**What moods, feelings, and thoughts were there before, during and after this event?**	**How did it contribute, or not, to achieving the goal?**	**What thoughts are in your mind now as you write about this event?**
**Mon**<br>**Date:**	W7_GO_D1_1.	W7_GO_D1_2a.<br>**Before:**<br><br>W7_GO_D1_2b.<br>**During:**<br><br>W7_GO_D1_2c.<br>**After:**	W7_GO_D1_3a.<br>**Before:**<br><br>W7_GO_D1_3b.<br>**During:**<br><br>W7_GO_D1_3c.<br>**After:**	W7_GO_D1_4.	W7_GO_D1_5.

Day/Date	Event related to goal	How did your body feel, in detail, before, during and after this experience?	What moods, feelings, and thoughts were there before, during and after this event?	How did it contribute, or not, to achieving the goal?	What thoughts are in your mind now as you write about this event?
**Tues**<br>**Date:**	W7_GO_D2_1.	W7_GO_D2_1.	W7_GO_D2_2a.<br>**Before:**<br><br>W7_GO_D2_2b.<br>**During:**<br><br>W7_GO_D2_2c.<br>**After:**	W7_GO_D2_3a.<br>**Before:**<br><br>W7_GO_D2_3b.<br>**During:**<br><br>W7_GO_D2_3c.<br>**After:**	W7_GO_D2_4.
**Wed**<br>**Date:**	W7_GO_D3_1.	W7_GO_D3_1.	W7_GO_D3_2a.<br>**Before:**<br><br>W7_GO_D3_2b.<br>**During:**<br><br>W7_GO_D3_2c.<br>**After:**	W7_GO_D3_3a.<br>**Before:**<br><br>W7_GO_D3_3b.<br>**During:**<br><br>W7_GO_D3_3c.<br>**After:**	W7_GO_D3_4.

Day/Date	Event related to goal	How did your body feel, in detail, before, during and after this experience?	What moods, feelings, and thoughts were there before, during and after this event?	How did it contribute, or not, to achieving the goal?	What thoughts are in your mind now as you write about this event?
**Thurs**<br>**Date:**	W7_GO_D4_1.	W7_GO_D4_1.	W7_GO_D4_2a.<br>**Before:**<br><br>W7_GO_D4_2b.<br>**During:**<br><br>W7_GO_D4_2c.<br>**After:**	W7_GO_D4_3a.<br>**Before:**<br><br>W7_GO_D4_3b.<br>**During:**<br><br>W7_GO_D4_3c.<br>**After:**	W7_GO_D4_4.
**Fri**<br>**Date:**	W7_GO_D5_1.	W7_GO_D5_1.	W7_GO_D5_2a.<br>**Before:**<br><br>W7_GO_D5_2b.<br>**During:**<br><br>W7_GO_D5_2c.<br>**After:**	W7_GO_D5_3a.<br>**Before:**<br><br>W7_GO_D5_3b.<br>**During:**<br><br>W7_GO_D5_3c.<br>**After:**	W7_GO_D5_4.

Day/Date	Event related to goal	How did your body feel, in detail, before, during and after this experience?	What moods, feelings, and thoughts were there before, during and after this event?	How did it contribute, or not, to achieving the goal?	What thoughts are in your mind now as you write about this event?
**Sat**<br>**Date:**	W7_GO_D6_1.	W7_GO_D6_1.	W7_GO_D6_2a.<br>**Before:**<br><br>W7_GO_D6_2b.<br>**During:**<br><br>W7_GO_D6_2c.<br>**After:**	W7_GO_D6_3a.<br>**Before:**<br><br>W7_GO_D6_3b.<br>**During:**<br><br>W7_GO_D6_3c.<br>**After:**	W7_GO_D6_4.
**Sun**<br>**Date:**	W7_GO_D7_1.	W7_GO_D7_1.	W7_GO_D7_2a.<br>**Before:**<br><br>W7_GO_D7_2b.<br>**During:**<br><br>W7_GO_D7_2c.<br>**After:**	W7_GO_D7_3a.<br>**Before:**<br><br>W7_GO_D7_3b.<br>**During:**<br><br>W7_GO_D7_3c.<br>**After:**	W7_GO_D7_4.

ID#________

Week 7: Goal Related to Diet, Alcohol Consumption, Physical Activity, Stress Reaction/Response, Antihypertensive Medication Use, or Social Support

W7_GO_1. What is your goal related to diet, alcohol consumption, physical activity, stress reaction/response, antihypertensive medication use, or social support for the week?

W7_GO_2. On a scale of 1-10, where 10 is high, how MOTIVATED are you to achieve this goal?

W7_GO_3. On a scale of 1-10, how CONFIDENT are you that you will achieve the goal?

W7_GO_4. What could you do that would bring your motivation or confidence a little higher?

W7_GO_5. What might make it difficult to achieve the goal this week, and if that happens, what will you do?

W7_GO_6. What is a way to measure this goal that resonates with you?

APPENDIX 4

Informed Consent Form

Agreement to Participate in a Research Study

Investigation of the Effects of Mindfulness on Blood Pressure and Well-Being

You are being asked to take part in a Brown University research study about the effects of mindfulness and hypertension education on blood pressure and risk factors for hypertension. This form will explain the purpose of the study, how the study will be carried out and what you will be expected to do. It will also explain the possible risks and possible benefits of being in the study. If any part of the following description is not clear to you, you are encouraged to contact the researcher to answer any questions before you decide whether to take part in the study. If you decide to participate, please fill out and sign the last page of this form.

1a. Nature and Purpose of the Study

The purpose of the study is to investigate the impact of mindfulness practices and health education on blood pressure. You have been selected for this study because you expressed interest and met entrance criteria for having prehypertension or hypertension, or another cardiovascular risk factor that could be influenced by this program. In order to assess the effects of these practices, you will be asked to complete some questionnaires, and a laboratory assessment before and after learning the mindfulness practices.

1b. Explanation of Procedures

If you agree to participate, you will be asked to consent to the following:

1) Participate in an interview in which you will be asked questions about past and present mental health, including depression and suicide.

2) Complete questionnaires about a wide range of topics, including your diet, physical activity, smoking, medication use, personality, emotions, attention and past experiences, including stressful or traumatic experiences. These questions will probe sensitive psychological areas, including physical, emotion and sexual abuse. These questionnaires may take up to 2 hours to complete. By completing the interviews and questionnaires, you are giving the researchers permission to use the information you have provided. You have the right not to answer any of the questions.

3) Directly assessed blood pressure, height, weight, waist circumference, hip circumference, physical activity, and antihypertensive medication use.

4) You will be asked to perform some cognitive tasks. One of these tasks may involve computer-based tests of attention or decision-making. Together these tests may take as long as 45 minutes.

5) You will participate in the mindfulness program, which consists of 9 weekly sessions of 2.5 hours each and will include one 8 hour weekend retreat. Daily homework assignments may take as long as one hour and consist of practicing mindfulness exercises with the aid of a

Eric Loucks 9/1/2015 9:23 PM
Deleted: and

Eric Loucks 9/1/2015 9:21 PM
Deleted: *2*

Eric Loucks 9/1/2015 9:21 PM
Deleted: *July 15*

guided audio CD and completing worksheets related to stress, thoughts, and common reactions to various types of events.

6) Class sessions will be audio taped so we can analyze the quality of the treatment you receive. The recordings will be transcribed so that we may analyze the text. The recordings will be identified by study number, will only be heard by study staff and will be destroyed after transcription.

7) You will be asked to complete a few short questionnaires each week during the 9 week condition.

8) After 9 weeks, you will be asked to complete questionnaires and return to the laboratory to repeat the same procedures for a second day of testing. You will also be invited to participate in a focus group to share any advice you may have on how to improve the intervention.

9) Three months after the end of the 9 week condition, you will be asked to return to the laboratory to repeat the same testing procedures.

Table Summarizing Activities and Time Commitment for this Study.

**Activity**	**Time Commitment**
First blood pressure screen	30 minutes
Second blood pressure screen	30 minutes
Baseline health assessments, such as questionnaires, height, weight and waist circumference.	2 hours
Mindfulness course	9 sessions that are 2.5 hours each.<br>1 retreat day on a Saturday that will be 8 hours<br>Total course time: 30.5 hours
Home mindfulness practices assigned during course.	1 hour per day, 6 days per week, for 8 weeks.<br>Total time: 48 hours.
Health assessments, such as questionnaires, blood pressure, height, weight and waist circumference, that take place immediately after course completion.	2 hours
Health assessments, such as questionnaires, blood pressure, height, weight and waist circumference, that take place that take place 3 months after course completion.	2 hours
Participate in focus group after intervention completion to share any advice you may have on how to improve the intervention.	1.5 hours
TOTAL TIME COMMITMENT FOR STUDY	87.0 hours

Eric Loucks 9/1/2015 9:23 PM
Deleted: 5.5

Eric Loucks 9/1/2015 9:21 PM
Deleted: 2

Eric Loucks 9/1/2015 9:21 PM
Deleted: *July 15*

Feedback:

At the end of the study, you will receive individual feedback about the changes that occurred since the first assessment. Specifically, you will receive an individualized handout listing % change (increase or decrease) on scales of attention, stress, mood, health behaviors, weight, and blood pressure across the study.

2. Discomforts and Risks

The questionnaires are routine, standardized forms for epidemiologic research. Certain questions may be upsetting as they may probe sensitive psychological areas and inquire about upsetting or traumatic events, including physical, sexual or emotional abuse and/or current psychiatric symptoms. Since your participation is voluntary, you have the right to skip any questions that make you uncomfortable.

Meditation-based interventions may results in discomfort with attention to unpleasant thoughts, feelings or body sensations. Some individuals may experience an initial increase in undesirable feelings with increased attention to them.

3. Benefits

We cannot and do not guarantee or promise that you will receive any direct benefits from this study. However, participation in the study creates the potential benefit of a) identifying effective treatments for elevated blood pressure, b) gaining knowledge of the effects of mindfulness practices, c) receiving information about your psychological and physical functioning.

4. Alternative Therapies

A number of different therapies, including antihypertensive medication, diet changes, physical activity, and reducing excessive alcohol consumption may also be beneficial for reducing blood pressure. Education about these therapies are integrated into this course, but other forms of these alternative therapies are also available in the community.

5. Confidentiality

Your responses for this study will be kept confidential. All data that we collect will be linked to a study ID# instead of your name. All questionnaires in this study will be filled out through an online survey or a paper version of the survey if you prefer. All of these questionnaires will be linked solely to your study ID#, so that your identity is protected and your answers are confidential. Although these measures have been taken to protect your personal information, complete confidentiality cannot be guaranteed when transmitting information over the internet.

While your confidentiality is protected to the extent of the law, there are limitations to confidentiality. If your questionnaire responses indicate that you pose a serious danger to yourself or to another person, then a collaborator (Dr. Ellen Flynn) who is a licensed psychiatrist, may contact you to discuss your responses and possible referral to a treatment provider. Questionnaire items that may warrant follow-up include endorsements of statements about hurting yourself, any high scores in depression, anxiety, or other clinically significant problems. You should also know that there are times when the law might require the release of your responses without your permission. For example, State law requires researchers to report abuse or neglect of children to the Department of Children, Youth and Families (DCYF). State law

Eric Loucks 9/1/2015 9:21 PM
Deleted: *2*

Eric Loucks 9/1/2015 9:21 PM
Deleted: *July 15*

also requires researchers to report abuse or neglect of people age 60 and older to the Division of Elderly Affairs.

The findings of the study may be used for medical publication. Your name will not be used in any published reports about this study. Results will be reported in a summarized manner in such a way that you cannot be identified. All personally identifiable information will be "de-identified" and only a unique code number will be used. Study records will be identified with a unique code number and initials. All study records and specimens will be stored in a secure storage area.

Keeping study records: The Principal Investigator for this study will keep your research records indefinitely for research purposes.

6. Refusal/Withdrawal
Participation is voluntary, and you may decide to opt out or refuse any part of participation, including not answering certain questions. If you decide now to participate, you can change your mind later and quit the study. The decision to not participate or to withdraw from the study will not adversely affect current or future interactions with Brown University. The decision to not participate or to withdraw from the study will also not adversely affect your relationship with your physician.

If you decide not to participate, or if you quit the study, we will provide you with referrals for alternative treatments, if desired.

7. Contact Information
If you have questions about study procedures at any time you may contact the researchers: Dr. Eric B. Loucks, email: eric.loucks@brown.edu, telephone (401) 863-6283. If you would like more information about the rules for research studies, or the rights of people who take part in those studies, you may contact the Brown University Research Protections Office, telephone number 1-866-309-2095 or 401-863-3050.

Eric Loucks 9/1/2015 9:21 PM
Deleted: *2*
Eric Loucks 9/1/2015 9:21 PM
Deleted: *July 15*

CONSENT FORM:
Please sign and return.

Please read the following agreement and sign to consent to participating.

I HAVE READ THE ABOVE DESCRIPTION OF THIS STUDY. ALL OF MY QUESTIONS HAVE BEEN SATISFACTORILY ANSWERED, AND, I AGREE TO PARTICIPATE IN THIS RESEARCH STUDY.

____
PRINT NAME

__________________________________ ________________
Signature of participant **Date**

CONTACT INFORMATION

Name (print):____

Permanent Address:____

Email(s):____

Telephone:______________________**(cell)** ______________________**(other)**

Eric Loucks 9/1/2015 9:21 PM
Deleted: *2*
Eric Loucks 9/1/2015 9:21 PM
Deleted: *July 15*

Brown University
Research Protections Office
Institutional Review Board
Amendment Request

Date of Request: 11/11/15 **Investigator's Name and Title:** Eric Loucks, Assistant Professor

Study Title: Mindfulness-Based Hypertension Therapy Pilot Study (#1412001171)

Original Type of Review: ☐ Exempt ☐ Expedited ☒ Full Board

1) **Provide a brief lay summary of the overall project. Include enough detail to allow the IRB to evaluate the requested change(s) within the context of the overall project.** (Attach summary to this form) Please see attached.

2) **Provide a detailed description of the changes being requested** (Use additional pages, if necessary):
Please see attached.

3.) **State the reason (justification) for the requested amendment.** (Use additional pages, if necessary):
Please see attached.

4.) **What is your assessment of how the changes will affect the overall risk/benefit ratio of the study and the willingness of individuals to participate?**
Please see attached.

5.) **Does the requested amendment require new documents or changes to the approved consent form or other documents?**

☒ Consent/assent documents (attach revised version with changes highlighted)

☒ New/revised instruments (attach - if revised, highlight changes)

☐ New/revised advertising materials (attach - if revised, highlight changes)

Do you have a conflict of interest on this project according to Brown's policy? ☐**YES** ☒**NO**

If YES, has this conflict been previously disclosed to the IRB? ☐**YES** ☐**NO**

PI signature: [signature] Date: 11/11/15

1. **Provide a brief lay summary of the overall project. Include enough detail to allow the IRB to evaluate the requested change(s) within the context of the overall project.**

The World Health Organization reported that suboptimal blood pressure (BP) is responsible for more than half of cardiovascular disease mortality world-wide. Furthermore, greater than half of those with hypertension have uncontrolled BP. *A 2009 Institute of Medicine report recommended prioritizing research to "Compare the effectiveness of mindfulness-based interventions (e.g. yoga, meditation, deep breathing training) and usual care in treating… cardiovascular risk factors."* Evidence-based mindfulness interventions, including Mindfulness-Based Stress Reduction, may have some effects on blood pressure, where a recent meta-analysis and systematic review of 4 randomized controlled trials demonstrated significant effects, but evidence of heterogeneity in effect sizes. The methodologically highest quality studies had the smallest effect sizes (range 0-5 mmHg). Mindfulness-Based Stress Reduction (MBSR) has been customized to a number of disease processes, such as Mindfulness-Based Cognitive Therapy for patients with recurrent depression, and Mindfulness-Based Relapse Prevention for patients with substance use addictions. Effect sizes have been increased by customizing mindfulness interventions to diseases of interest. The same may be true for hypertension, *however mindfulness interventions customized for prehypertensive/hypertensive patients have never been investigated. Until methodologically rigorous studies to evaluate customized interventions for hypertension are performed, we will not know if the observed preliminary effects of general mindfulness interventions on blood pressure reduction could be much more effective with a tailored approach.* Consequently, we propose to conduct a stage I behavioral therapy intervention study to evaluate whether MBSR customized to prehypertensive and hypertensive patients has the potential to provide clinically relevant reductions in BP. Consequently the specific aims are:

Stage 1a: Therapy Development/Manual Writing

1. To outline and evaluate key novel elements of mindfulness-based hypertension therapy (MBHT), customized from the evidence-based MBSR. *We hypothesize that the most important novel element will be generation of mindfulness skills specifically applied to hypertension risk factors such as diet, physical activity, obesity, alcohol consumption and antihypertensive medication adherence.* This aim will be achieved using (1) focus groups of participants undergoing the MBHT behavioral intervention, (2) discussion with experts (including cardiologists, epidemiologists, mindfulness experts, mindfulness intervention instructors) prior to, and following pilot testing of MBHT in participants, and (3) clinical judgment of the investigators performing the intervention.
2. To determine effectiveness of MBHT on primary outcomes (systolic blood pressure, retention rates, recruitment rates, and adverse effects) and secondary outcomes (hypertension risk factors such as diet, physical activity, obesity, and antihypertensive medication adherence) in hypertension subgroups, specifically participants with (1) prehypertension, (2) controlled hypertension, and (3) uncontrolled hypertension. Initial decisions about the targeted sample based on hypertension status will be made.
3. To develop an MBHT therapist manual and training program, including procedures for training, supervising, and evaluating therapists. Furthermore, acceptable therapist characteristics will be developed. The manual and training program will include themes such as specification of unique and common elements of MBHT *vs.* other interventions, description of interventions excluded from MBHT, and specification of key treatment parameters such as frequency and duration of treatment, session length, topics addressed, sequence of sessions, as well as therapist adherence and competency

measures. The MBHT training will consist of a therapist manual, a formal didactic training seminar, and at least one closely supervised training session.

Stage Ib: Pilot Trial

4. To determine whether a mindfulness-based hypertension therapy (MBHT) intervention, customized from the evidence-based MBSR, has promise to be an effective behavioral therapy for participants with hypertension and/or prehypertension. We will perform a randomized controlled pilot trial for MBHT *vs.* enhanced usual care control. *We hypothesize that MBHT will have adequate recruitment rates (≥10% of prehypertensive/hypertensive participants invited from physicians' offices), fairly low drop out rates (<15%), and medium effect sizes (e.g. 5-10 mmHg systolic BP) for reduction in blood pressure.*

These findings will provide publishable pilot data that will inform future randomized clinical trials that evaluate effects of MBHT on long-term changes in blood pressure *vs.* usual care and active control groups. *If proven effective, MBHT could be offered as a complementary program in the prehypertensive/hypertensive patient population that contributes to over half of the cardiovascular disease mortality world-wide.*

2. Provide a detailed description of the changes being requested (Use additional pages, if necessary):

Study Protocol

We would like to update you on the following changes made to the overall study protocol…

- **NIH funding** – On September 14, 2015, we received notification from the NIH that our grant [Award # 1UH2AT009145-01] has been approved and will be funded in full.

- **Control group –** we had initially thought we would introduce a control group further down the road in 2017 or 2018, but our funding agency (NCCIH) has asked that we add a control group at this time. For this reason, we will be randomly selecting eligible participants to take part in the intervention. Those not selected, will receive enhanced usual care and will be invited to participate in the mindfulness training program around six months after the intervention group. Enhanced usual care in the setting of this study refers to participants obtaining hypertensive treatment through the services typically available to them. However, we will help participants access these services if needed.

- **Participant incentive** – we now have sufficient funds to offer participants a small participant incentive for their involvement in the study. Study participants will be given a participant incentive of $100 USD at the time of their last follow up. Only participants who have completed the study will be given the incentive.

- **Safety Monitoring** – as part of the NCCIH submission, a formal Safety Monitoring Protocol was created. We have included that here as part of the study protocol (see Appendix 1).

- **Assessment Name Changes** - Several of the assessment document names have been changed. We did this both for internal consistency and in order to clarify the timing and purpose of each assessment. Specific name changes are noted below.

- **Consent Form Modifications** – we have modified the consent form (see below for details as well as Appendix 3 for the revised consent form, v.1.4)

- **Additional Follow-up Added** – we have decided to add an additional follow up to the study, which will take place at 1 year post-baseline. The assessments conducted at this follow up will be identical to those administered at the 10 week and 6 month follow up visits.

- **Questionnaire Revisions** - we have made changes to several of the individual Assessments (see below for details as well as Appendices 4-7 for revised assessments)

<u>Consent Form</u> (v.1.4, See Appendix 3)

We have made the following changes to the study consent form:

- Added clarifying language on study procedures.
- Added in language about a control group. We had initially thought we would hold off on including a control group until a later date, but our funding agency (NCCIH) has asked that we add a control group at this time. For this reason, we will be randomly selecting eligible participants to take part in the intervention. Those not selected, will receive enhanced usual care and will be invited to participate in the mindfulness training program around six months after the intervention group.
- Added more detail about physical activity monitoring (i.e., use of actigraphy monitors) as well medication use monitoring (i.e., the use of electronic bottle caps).
- Added information about focus group involvement post-intervention.
- Added language on risk of physical injury.
- We have decided to offer a participant incentive in the amount of $100 USD, paid at the time of final follow up visit. This has been added to the consent form.
- Added information about the 1 year follow up assessment.
- Updated the Table Summarizing Activities and Time Commitment for the study

<u>Phone Screener</u>: (v.1.4, See Appendix 4)

- Modified language to make more concise and clear
- Added information about the randomization process / control group
- Added in information about the participant incentive
- Corrected typos
- Removed the script about scheduling the second In-Person visit. This will be done at the time of the In-Person screener, if the person is found to be eligible for the study and is still interested in participating.

In-Person Screening: (v.1.4, See Appendix 5)

- **Changed the name.** It was formally called the "First In-Person Visit Screening Assessment Form"
- **Moved "Medication Use" to the In-Person Baseline Assessment** – the primary purpose of the In-Person Screening Assessment is to determine whether or not an individual is eligible for the study (i.e., have mean systolic/diastolic blood pressure ≥120/80 after first and second baseline assessments). For this reason, we decided to move medication use, which was originally found in the "First In-Person Visit Assessment Form" to the In-Person Baseline Assessment since it is not part of the eligibility criteria. This will help to reduce participant burden for those individuals who are not eligible.
- *NOTE that the only assessment conducted during the In-Person Screening is Blood Pressure measurements.*

Home Baseline Assessment: (v.1.4, See Appendix 6)

- Changed the name. It was formally called the "Baseline Questionnaire"
- Corrected typos, including skip errors
- Updated the Table of Contents and Sub-headings in questionnaire
- Removed Diet questions (DT1_01 – DT1_25); we will assess diet during the In-Person Baseline Assessment using the Harvard University 80-out Food Frequency Questionnaire
- Added a question (TS01_01) on table salt use
- Added the 11 item validated Craving Experience Questionnaire (CEQ)
- Moved around some of the sub-scales for ease of administration (noted in text).
- Removed Social Network questions (SN1_01 – SN1_04) and replaced with the validated 12-item Interpersonal Support Evaluation List (ISEL-12)
- Removed the Social Network questions (SN1_01 – SN1_04)
- Added Blood Pressure Medication Use questions (BM1_01 – BM1_09), which includes the validated Morisky 8-item questionnaire[1].
- Added questions that measure "Readiness to change for hypertension risk factors" (RC1_01 – RC1_14)
- Added Family History of Hypertension questions (FH1_01 – FH1_04), which assess biological parents' history of having hypertension, based on questions from New England Family Study LEAP Project.
- Dropped several of the sleep items (SL1_05 – SL1_14a; SL1_16 – SL1_21b) to reduce ppt burden.

In-Person Baseline Assessment (v.1.4, See Appendix 7)

- Changed the name. It was formally called the "Second In-Person Visit Screening Assessment"
- Added Medication use questions (ME01 – ME10i)
- Added the validated Harvard University three page Food Frequency Questionnaire, known as "80-out". It is designed to take around 20 minutes to complete and is self-administered with paper and pencil. A proof of the questionnaire is included in this Amendment Request.
- Added two computer-based tests that measure Attention Control. They are:

  The Attention Network Test (ANT) is a brief computerized battery measuring three independent behavioral components of attention: Conflict resolution (ability to overcome distracting stimuli), spatial Orienting (the benefit of valid spatial pre-cues), and Alerting (the benefit of temporal pre-cues). Efficiency of orienting is examined by changes in RT that accompany cues indicating where the target will occur. The efficiency of the executive conflict resolution network is examined by requiring the subject to respond by pressing two keys indicating the direction (left or right) of a central arrow surrounded by congruent, incongruent or neutral flankers. Moderate to high reliabilities are found for all networks. The ANT takes around 20min to complete.

  The Sustained Attention to Response Task (SART) is a computerized test of sustained attention, response inhibition (executive function) and self-regulation. Subjects are instructed to press a key in response to rapidly displayed integers (1-9) and withhold response to a designated "no-go" integer. SART errors consist of summed commission errors (button press on no-go trial) and omission errors (button not pressed on "go" integers). SART performance is associated with prefrontal cortex functioning, has been found to increase with mindfulness training and is correlated with scores on mindfulness questionnaires (specifically, the Mindful Attention Awareness Scale). The SART takes around 15min to complete.

3. State the reason (justification) for the requested amendment:

Please see justifications in Section 2 above.

4. What is your assessment of how the changes will affect the overall risk/benefit ratio of the study and the willingness of individuals to participate?

Overall the changes we have made in this amendment affect the risk/benefit ratio in small ways. We have added some question items to the assessments, but have also removed some items in order to minimize participant burden. Assessment time has increased by about 1 hour per assessment.

The addition of a participant incentive adds a tangible benefit to participants and serves as a potential motivation to complete the study.

The inclusion of a formalized Safety Monitoring protocol helps reduce participant risk by predicting potential areas of concern and by outlining clear procedures with how to manage incidents that may come up.

5. Does the requested amendment require new documents or changes to the approved consent form or other documents?

Yes. Please see **Appendices 1-7.**

Appendix 1	Safety Monitoring Protocol
Appendix 2	Summary of Assessments
Appendix 3	Consent Form (v.1.4)
Appendix 4	Phone Screener (v.1.4)
Appendix 5	In-person Screener (v.1.4)
Appendix 6	Home Baseline Assessment (v.1.4)
Appendix 7	In-Person Baseline Assessment (v.1.4)

References

[1] Morisky DE, Green LW, Levine DM. Concurrent and predictive validity of a self-reported measure of medication adherence. Med Care. 1986;24(1):67-74.

Appendix 1:
Safety Monitoring Protocol

(Language Taken from the NCCIH Protocol Submission)

Safety Monitoring - ***as part of the NCCIH submission, a formal Safety Monitoring Protocol was created. It is outlined below.***

Oversight of internal monitoring of the participants' safety will be conducted by the PIs, Dr. Eric Loucks and Dr. Willoughby Britton. Oversight of the external Data and Safety Monitoring Committee will be conducted by the chair (Dr. Edmondson). The Data and Safety Monitoring Committee will include experts in cardiology, psychology/psychiatry, epidemiology, and biostatistics.

Entities Conducting Monitoring

The Institutional Review Board (IRBs) at Brown University will review all research procedures, and will provide oversight. Internal monitoring will be done by the Brown University principal investigators (Dr. Loucks and Dr. Britton) and the Brown University IRB. The Data Safety Monitoring Committee will provide external monitoring, and will meet every six months by phone or in-person. They will be provided data every six months to evaluate potential effects of the RCT on major outcomes (e.g. medical regimen adherence). Any serious adverse effects will be immediately reported to the principal investigators (Loucks, Britton) and the committee chair.

What is Monitored

Monitoring is done of all procedures to ensure that they conform to the approved protocol; of unforeseen circumstances that might arise and affect safety; of all reports of serious adverse events as defined in US Department of Health and Human Services regulations for the protection of human research subjects 45 CFR Part 46, and the FDA 312.32 (death, life-threatening experience, new or prolonged hospitalization, persistent or significant disability/incapacity); of other significant adverse events (adverse events that lead to drop out by participant or termination by the investigator); of unexpected adverse events resulting from the study; and of expected adverse events.

Monitoring is done of all study inclusion and exclusion criteria. During this clinical trial, we will notify officials, as mandated by law, if a participant reports intentions to harm him/herself or others, or reports child abuse or abuse of an elder. Dr. Ellen Flynn, a licensed psychiatrist, will be available to advise on any psychological events that occur, and provide referrals for treatment if needed.

Frequency of Monitoring

All adverse events will be continuously monitored by the PI. Participants will be given contact information so that they can inform us of events that occur in between study visits. Dr. Loucks will conduct daily oversight of participant safety. He will meet weekly with staff to review participant progress and their experiences with the experimental procedures, including adverse events. Any adverse events that are observed and/or reported will be immediately reported to Dr. Loucks, Dr. Britton, and the Data Safety Monitoring Committee chair. The Investigators and Data Safety Monitoring Committee will be available to meet outside of the regularly scheduled meetings (scheduled semiannually), if necessary, due to concerns regarding a particular participant or any problems that may arise for participants. If necessary, they will make appropriate recommendations for changes in protocol, or terminate the study. The Brown University IRB conducts the monitoring at the continuing reviews as scheduled, whenever modification requests are considered, and upon receiving reports of serious adverse events from the PI or anyone else.

Reporting Plan

Any serious adverse events that are observed and/or reported will be immediately reported to Dr. Loucks and the Data Safety Monitoring Committee Chair. Serious adverse events are

then reported to the Brown University IRB and to NIH. Brown's IRB requires fatalities related to the study be reported within 24 hours. All serious adverse events related to this study will be reported to the Brown University IRB immediately by telephone and by written report within 48 hours of our receipt of information regarding the event. All other adverse events related to the study will be reported at the continuing review. Serious adverse events will also be reported in writing to the NIH Project Officer within 48 hours. All serious adverse events related to the study will be reported annually in the Progress Report sent to the NIH Project Officer.

Any actions taken by the IRB, other than acceptance of the adverse event report, will be reported to the NIH along with any changes or amendments to the protocol requested by the IRB in response to these reports. Proposed changes or amendments to the protocol in general must be requested first in writing to the Brown and IRB, which will then grant or deny permission to make the requested change or amendment in protocol. NIH will subsequently be informed of any substantive changes or amendments in approved protocol.

Injury due to physical activities: It is possible that injuries could be sustained from (1) the gentle mindful movements (yoga), or (2) physical activities that participants engage in as a result of the intervention encouraging exploration of physical activity as a way to reduce blood pressure.

(1) Mindful movements: Participants receive a handout during the orientation showing the yoga poses that will be offered during the course. They are encouraged to explore limits in their body related to movement, but not to go beyond those limits Participants are asked to listen to what their body is telling them more closely than what the mindful movement instructor is telling them. Modifications of poses are available, including for those limited to chairs or wheelchairs. Participants are encouraged to bring the handout of poses to their health care providers if they have any physical limitations, so that the providers can advise on which poses to do, and which to avoid.
(2) Physical activities: Participants are encouraged to explore physical activities that promote strength and conditioning as a way to reduce blood pressure. As with the mindful movements, they are encouraged to explore limits in their body related to movement, but not to go beyond those limits. Participants are asked to listen to what their body is telling them more closely than what the mindful movement instructor is telling them. Furthermore, they are encouraged to ask their healthcare provider about advised physical activities if they have any physical limitations.

All patients will be monitored by study staff at weekly intervals in person or by telephone and assessed every two weeks with questionnaires about physical injuries, specifically the following questions:

1. Have you sustained any physical injuries during the past 2 weeks?

If yes,
(a) please describe the injury
(b) please describe what happened to cause the injury.
(c) did this injury include
  i. Broken bone(s) yes/no
  ii. Lacerations requiring stiches yes/no
  iii. Concussion yes/no

If injuries include any of the following, it will be reported to Data Safety Monitoring Committee member Dr. Hank Wu, MD, who is a practicing physician and researcher. He will advise on any follow-up needed. It will also be reported to the chair of the Data Safety Monitoring Board.

a. Broken bone(s)
b. Lacerations requiring stiches
c. Concussion

Non-Response to Treatment: The possibility that the treatment will not yield benefit is another possible risk and will be explained during informed consent procedures. Non-responders (identified as minimal change in medical regimen adherence from baseline assessment) will be provided with referrals to other treatment, if desired.

INTERVENTION DISCONTINUATION

If serious adverse events are significantly higher in the treatment vs. control groups, the study will be terminated.

COMMITTEES

Data Safety Monitoring Board:

Oversight of internal monitoring of the participants' safety will be conducted by the PIs, Dr. Eric Loucks and Dr. Willoughby Britton. Investigators on this application have extensive experience with clinical trials for mindfulness-based interventions and cardiovascular outcomes. Oversight of the external Data and Safety Monitoring Committee will be conducted by the chair, Dr. Donald Edmondson, PhD, is Assistant Professor of Behavioral Medicine at Columbia University Medical Center. He is a Psychologist, and has extensive research experience in evaluating effects of stress and psychsocial factors on cardiovascular disease outcomes.[246-264] The Data and Safety Monitoring committee will also include Dr. Wen-Chih (Hank) Wu and Dr. Tao Liu. Dr. Wu, MD, is Associate Professor of Medicine and Associate Professor of Epidemiology at Brown University. He is a practicing clinical cardiologist with research in preventive cardiology,[265-272] and will be able to advise on clinical outcomes and any cardiovascular complications arising from the study, in addition to methodological concerns. Dr. Liu, PhD, is an Assistant Professor of Biostatistics at Brown University, experienced in clinical trials.[273,274] He will receive all preliminary analyses from the primary statistician, and will have access to all data from the study, to evaluate any evidence of serious adverse effects or other concerns.

Entities Conducting Monitoring

The Institutional Review Board (IRBs) at Brown University will review all research procedures, and will provide oversight. Internal monitoring will be done by the principal investigators (Dr. Loucks and Dr. Britton) and the Brown University IRB. The Data Safety Monitoring Committee will provide external monitoring, and will meet every six months by phone or in-person. During the randomized-controlled trial phase (phase 4), they will be provided data every six months to evaluate potential effects of the RCT on the primary outcome (i.e. medical regimen adherence). Any serious adverse effects will be immediately reported to the principal investigator (Loucks) and the committee chair (Edmondson).

Appendix 2:
Summary of Assessments

Summary of Assessments (e.g., timing and intent of assessment):

(1) Phone Screener

- Previously called the "Phone Screening Questionnaire"
- Completed by phone any time prior to the intervention
- Used to screen individuals for the following eligibility criteria: age, language, mental health, and mindfulness practices (see protocol for details on eligibility requirements). Blood pressure, the primary eligibility criteria of interest, will be measured in person at the in-person screening.
- Phone screener provides talking guidelines for the RA to use in order to set up appointments for the in-person screening and to find out about general availability. Also provides the interested individual with information about the study.

(2) In-Person Screening

- Previously called the "First In-Person Visit Screening Assessment Form"
- Completed up to three months prior to intervention
- 1st BP reading taken in order to determine eligibility
- Takes place at 121 South Main Street (office/laboratory)
- If eligible, Consent Form is completed at this visit
- Estimated time to complete is 20-30 minutes

(3) Home Baseline Assessment

- Previously called the "Baseline Questionnaire"
- Completed up to three weeks prior to commencement of intervention
- Done online by participant at home or other location of choice. Participant will be sent a link to the online survey or will be provided with a paper version (if preferred).
- Questionnaire will take around 2-2.5 hrs to complete

(4) In-Person Baseline Assessment

- Previously called the "Second In-Person Visit Screening Assessment"
- Completed up to three weeks prior to intervention
- 2nd BP reading will be taken as well as the following assessments:
    - Height, weight, and hip and waist circumference
    - Medication use
    - Diet
    - Anxiety and depressive symptomology
    - Attention control activities (ANT and SART)
- Takes place at 121 South Main Street (office/laboratory)
- Estimated time to complete is around 1.5 to 2 hrs

(5) Mindfulness Course

- 9 sessions that are 2.5 hrs each as well as 1 retreat day on a Saturday that will be 8hrs long
- Participants will be given mindfulness 'homework' to practice daily (around 1hr per day, 6 days per week for 8 weeks)
- Participants will be asked to complete mindfulness assignments from home during the course. These assessments have already been approved by the IRB.
- Also approved are Teacher Fidelity assessments that will be administered during weeks 4 and 8.

(6) 10 Week Post-Intervention Assessments (HOME and IN-PERSON)

- To take place roughly 10 weeks from Baseline, after the Mindfulness Training is complete.
- Similar to assessments administered at Baseline, there will be both an online component that can be completed by the participant at home, and an in-person component that will take place at 121 South Main Street (office/laboratory)
- Questionnaires and assessments to be completed at this visit are identical to those administered at the In-Person Baseline Assessment, with the exception of question items for which the answers should not change or be informative (e.g., age, race/ethnicity, education, adverse childhood experiences, family history of hypertension).
- Additionally, adverse events are monitored at the 10 week, 6 month and 1 year follow-ups.
- Estimated time to complete is up to 3 hrs

(7) Focus Group

- Participants will be invited to take part in a focus group after the intervention is complete in order to share any advice they may have on how to improve the intervention
- Takes place at 121 South Main Street
- Estimated time is 1.5 hrs

(8) 6 Month Post-Intervention Assessments (HOME and IN-PERSON)

- To take place roughly 6 months from Baseline, after the Mindfulness Training is complete.
- Similar to assessments administered at Baseline, there will be both an online component that can be completed by the participant at home, and an in-person component that will take place at 121 South Main Street (office/laboratory)
- Questionnaires and assessments to be completed at this visit are identical to those administered at the In-Person Baseline Assessment, with the exception of question items for which the answers should not change or be informative (e.g., age, race/ethnicity, education, adverse childhood experiences, family history of hypertension).
- Additionally, adverse events are monitored at this follow up.
- Estimated time to complete is up to 3 hrs

(9) 1 Year Post-Intervention Assessments (HOME and IN-PERSON) ***<u>NEW</u>***

- To take place roughly 1 year from Baseline, after the Mindfulness Training is complete.
- Similar to assessments administered at Baseline, there will be both an online component that can be completed by the participant at home, and an in-person component that will take place at 121 South Main Street (office/laboratory)
- Questionnaires and assessments to be completed at this visit are identical to those administered at the In-Person Baseline Assessment, with the exception of question items for which the answers should not change or be informative (e.g., age, race/ethnicity, education, adverse childhood experiences, family history of hypertension).
- Additionally, adverse events are monitored at this follow up.
- Estimated time to complete is up to 3 hrs

Appendix 3:
Revised Consent Form
with Track Changes

(v.1.4 – November 6, 2015)

Agreement to Participate in a Research Study

Investigation of the Effects of Mindfulness on Blood Pressure and Well-Being

You are being asked to take part in a Brown University research study about the effects of mindfulness and hypertension education on blood pressure and risk factors for hypertension. This form will explain the purpose of the study, how the study will be carried out and what you will be expected to do. It will also explain the possible risks and possible benefits of being in the study. If any part of the following description is not clear to you, you are encouraged to contact the researcher to answer any questions before you decide whether to take part in the study. If you decide to participate, please fill out and sign the last page of this form.

1a. Nature and Purpose of the Study

The purpose of the study is to investigate the impact of mindfulness practices and health education on blood pressure. You have been selected for this study because you expressed interest in the project and because you met entrance criteria for having prehypertension or hypertension, or another cardiovascular risk factor that could be influenced by this program.

In order to assess the effects of these practices, you will be asked to complete some questionnaires and laboratory assessments before and after learning the mindfulness practices. Assessments will be completed at: baseline, 10 weeks, 6 months and 1 year. If you complete the study, you will be given $100 USD at the time of completion for your participation, to express our gratitude.

Deleted: a

Participation in this study involves receiving training in mindfulness practices as well as health education on blood pressure. If eligible, you will be randomly assigned to either receive the training now or in about six months from now (if still available and interested).

1b. Explanation of Procedures

If you agree to participate, you will be asked to consent to the following:

1) Participate in an interview in which you will be asked questions about past and present mental health, including depression and suicide.

2) Complete questionnaires about a wide range of topics, including your diet, physical activity, smoking, medication use, personality, emotions, attention and past experiences, including stressful or traumatic experiences. These questions will probe sensitive psychological areas, including physical, emotion and sexual abuse. These questionnaires may take up to 3 hours to complete. By completing the interviews and questionnaires, you are giving the researchers permission to use the information you have provided. You have the right not to answer any of the questions.

Deleted: 2

3) Directly assessed blood pressure, height, weight, waist circumference, hip circumference, physical activity, and antihypertensive (blood pressure) medication use at baseline and after the mindfulness course. Physical activity will be assessed for a week at a time using small

Deleted: and

Deleted: .

actigraphy monitors that attach to your wrist and hip. If you take antihypertensive medication, we will provide you with an electronic bottle cap that will automatically record when the pill bottle is opened during the study. This will help us measure how often the medication is used.

4) You will be asked to perform some cognitive tasks. Some of these tasks may involve computer-based tests of attention or decision-making. Together these tests may take as long as 45 minutes.

Deleted: One

5) You will participate in the mindfulness program, which consists of 9 weekly sessions of 2.5 hours each and will include one 8 hour weekend retreat. Daily homework assignments may take as long as one hour and consist of practicing mindfulness exercises with the aid of a guided audio CD and completing worksheets related to stress, thoughts, and common reactions to various types of events.

6) Class sessions will be audio taped so we can analyze the quality of the treatment you receive. The recordings will be transcribed so that we may analyze the text. The recordings will be identified by study number, will only be heard by study staff and will be destroyed after transcription.

7) You will be asked to complete a few short questionnaires each week during the 9 week condition.

8) After 10 weeks, you will be asked to complete questionnaires and return to the laboratory to repeat the same procedures for a second day of testing. You will also be invited to participate in a focus group to share any advice you may have on how to improve the intervention.

Deleted: 9

9) Six months and one year after the beginning of the study, you will be asked to return to the laboratory to repeat the same testing procedures.

Deleted: Three

Deleted: the end of the 9 week condition

Table Summarizing Activities and Time Commitment for this Study.

Activity	Time Commitment
First blood pressure screen	30 minutes
Second blood pressure screen	30 minutes
Baseline health assessments, such as questionnaires, height, weight and waist circumference.	3 hours
Mindfulness course *In-person*	9 sessions that are 2.5 hours each. 1 retreat day on a Saturday that will be 8 hours *Total course time: 30.5 hours*
Home practices assigned during mindfulness course.	1 hour per day, 6 days per week, for 8 weeks. *Total practice time: 48 hours*
Health assessments, such as questionnaires, blood pressure, height, weight and waist circumference, that take place *immediately after course completion*.	3 hours
Health assessments, such as questionnaires, blood pressure, height, weight and waist circumference, that begin *6 months after baseline*.	3 hours
Health assessments, such as questionnaires, blood pressure, height, weight and waist circumference, that begin *1 year after baseline.*	3 hours
Participate in focus group after intervention completion to share any advice you may have on how to improve the intervention.	1.5 hours
**TOTAL TIME COMMITMENT FOR STUDY**	**93.0 hours**

Deleted: 2 hours

Deleted: mindfulness

Deleted: 2 hours

Deleted: 2 hours

Deleted: *take place that take place 3*

Deleted: course completion

Deleted: **87.0 hours**

Feedback:

At the end of the study, you will receive individual feedback about the changes that occurred since the first assessment. Specifically, you will receive an individualized handout listing % change (increase or decrease) on scales of attention, stress, mood, health behaviours, weight, and blood pressure across the study.

2. Discomforts and Risks

The risks to you in this study are small. The questionnaires used in the study are routine, standardized forms for epidemiologic research. Certain questions may be upsetting as they may probe sensitive psychological areas and inquire about upsetting or traumatic events, including physical, sexual or emotional abuse and/or current psychiatric symptoms. Since your participation is voluntary, you have the right to skip any questions that make you uncomfortable.

Meditation-based interventions may results in discomfort with attention to unpleasant thoughts, feelings or body sensations. Some individuals may experience an initial increase in undesirable feelings with increased attention to them.

It is possible that injuries could be sustained during the study either from the gentle mindful movements (i.e., yoga), or from physical activities that participants engage in as a way to reduce blood pressure. To help limit this, you will receive a handout showing the yoga poses that will be offered during the course that you can show your health care provider so that they can advise on which poses to do, and which to avoid. Modifications of poses will be available as needed. None of the poses (or the yoga as a whole) are mandatory to be done. You will also be encouraged to explore physical activities that promote strength and conditioning as a way to reduce blood pressure. You will be encouraged to not go beyond any physical limits of your body, and will be encouraged to ask your healthcare provider about advised physical activities and mindful movements if you have any physical limitations.

3. Benefits

We cannot and do not guarantee or promise that you will receive any direct benefits from this study. However, participation in the study creates the potential benefit of a) identifying effective treatments for elevated blood pressure, b) gaining knowledge of the effects of mindfulness practices, c) receiving information about your psychological and physical functioning.

To express our gratitude, at the end of your last follow-up visit, you will receive $100 USD for your participation in the study.

4. Alternative Therapies

A number of different therapies, including antihypertensive medication, diet changes, physical activity, and reducing excessive alcohol consumption may also be beneficial for reducing blood pressure. Education about these therapies are integrated into this course, but other forms of these alternative therapies are also available in the community.

5. Confidentiality

Your responses for this study will be kept confidential. All data that we collect will be linked to a study ID# instead of your name. All questionnaires in this study will be filled out through an online survey or a paper version of the survey if you prefer. All of these questionnaires will be linked solely to your study ID#, so that your identity is protected and your answers are confidential. Although these measures have been taken to protect your personal information, complete confidentiality cannot be guaranteed when transmitting information over the internet.

While your confidentiality is protected to the extent of the law, there are limitations to confidentiality. If your questionnaire responses indicate that you pose a serious danger to yourself or to another person, then a collaborator (Dr. Ellen Flynn) who is a licensed psychiatrist, may contact you to discuss your responses and possible referral to a treatment provider. Questionnaire items that may warrant follow-up include endorsements of statements about hurting yourself, any high scores in depression, anxiety, or other clinically significant problems. You should also know that there are times when the law might require the release of your responses without your permission. For example, State law requires researchers to report abuse or neglect of children to the Department of Children, Youth and Families (DCYF).   State law

also requires researchers to report abuse or neglect of people age 60 and older to the Division of Elderly Affairs.

The findings of the study may be used for medical publication. Your name will not be used in any published reports about this study. Results will be reported in a summarized manner in such a way that you cannot be identified. All personally identifiable information will be "de-identified" and only a unique code number will be used. Study records will be identified with a unique code number and initials. All study records and specimens will be stored in a secure storage area.

Keeping study records: The Principal Investigator for this study will keep your research records indefinitely for research purposes.

6. Refusal/Withdrawal

Participation is voluntary, and you may decide to opt out or refuse any part of participation, including not answering certain questions. If you decide now to participate, you can change your mind later and quit the study. The decision to not participate or to withdraw from the study will not adversely affect current or future interactions with Brown University. The decision to not participate or to withdraw from the study will also not adversely affect your relationship with your physician.

If you decide not to participate, or if you quit the study, we will provide you with referrals for alternative treatments, if desired.

7. Contact Information

If you have questions about study procedures at any time you may contact the researchers: Dr. Eric B. Loucks, email: eric.loucks@brown.edu, telephone (401) 863-6283. If you would like more information about the rules for research studies, or the rights of people who take part in those studies, you may contact the Brown University Research Protections Office, telephone number 1-866-309-2095 or 401-863-3050.

CONSENT FORM:
Please sign and return.

Please read the following agreement and sign to consent to participating.

I HAVE READ THE ABOVE DESCRIPTION OF THIS STUDY. ALL OF MY QUESTIONS HAVE BEEN SATISFACTORILY ANSWERED, AND, I AGREE TO PARTICIPATE IN THIS RESEARCH STUDY.

____
PRINT NAME

___________________________________ _________________
Signature of participant **Date**

CONTACT INFORMATION

Name (print):____

Permanent Address:____

Email(s):____

Telephone:________________________**(cell)** ________________________**(other)**

Appendix 4:
Revised Phone Screener
with Track Changes

(v.1.4 – November 6, 2015)

PHONE-DELIVERED SCREENING QUESTIONNAIRE

PHONE SCREENING QUESTIONNAIRE PART 1

*The script is shown below in **bold italics.***

PID. Participant ID: ___________

SQ01. Staff ID: ______________

SQ02. Today's Date (MMDDYY): ________________

SQ03. Current Time (24 hour time, e.g. 14:45): ________

Please now call the participant.

SQ04. Was the participant reached? YES NO

Hello, my name is ____________. I am calling from the Brown University School of Public Health because (name of participant) expressed interest in participating in our mindfulness blood pressure study. Is he/she available to talk at this time?

I would like to do a 10-15 minute phone interview with you to determine if you are a good match for this particular study.

*SQ05. **Is now a good time to speak?***

[If yes, proceed to SQ05 script below]

If no… ***When would be a good time to talk?***

SQ06. Day to call back (DDMMYY) ____________

SQ07. Time to call back ______ AM/PM

OK, great. How about I give a quick overview of the study, and can then answer any questions you may have to see if it is a good fit for you. We will then go through a screening questionnaire to see if you qualify for the study. Does this sound OK?

In this study, we are looking to see if mindfulness practices improve blood pressure, and if education about hypertension risk factors may also improve blood pressure. If eligible and if selected to participate, we will provide you with training in meditation, mindful movements, and the roles of things like diet, physical activity and medication in reducing blood pressure. You will be taught by a very experienced teacher who is an expert in these fields. The course is free and will take place over a 9-week period, where you come to a class once each week for 2.5 hours each time. There is also a one-day retreat on a Saturday that will be 8 hours long. As part of the project, we will also ask you to participate in health assessments before and after the study. Health assessments include measures such as blood pressure, height and weight, and questionnaires about your health and experiences. At the end of the study, you will be given $100 USD to express our gratitude.

Deleted: ***A basic overview of the study is that it is***

Deleted: ***Specifically***

Deleted: ***Y***

Deleted: ***s***

Deleted: ***Also, w***

Do you have any questions about the study? *[answer questions]*

Deleted: *Can I answer any questions about the study that you may have*

SQ08. ***Does this study sound like something you would be interested in doing? Yes No***

[If yes, proceed to next statement below. If no, politely thank the participant for considering being in this study, and end the call].

Great. There are a few things that I would like to make clear before we start the interview. First of all, some of the questions that I will ask now to figure out if you are eligible to be in this study will be of personal nature, including asking about your mental health and life's experiences.

SQ09. ***Are you in a private place to talk?***

[If yes, proceed to text below. If no, reschedule meeting using variables SQ06 and SQ07 above].

Deleted: SQ04

Deleted: SQ05

Because this interview is of a personal nature, it is important that you understand that everything you say will be kept strictly confidential. No one outside of our project will ever be able to see your answers, and we will not keep your name in the same place as any of your answers. If you are not eligible after the phone screen, we will destroy your information. If you like, though, we can keep your information on file for future studies.

SQ10. Participant's First Name: ______________________________________

SQ11. Participant's Last Name:____

Participant's Address (in case we need to send any study materials to you):
SQ12a. Street address: _____________
SQ12b. City: ____________________
SQ12c: State: ________
SQ12d: Zip Code: ________________

SQ13a. Participant's Phone number #1 (in case we need to contact you by phone)
____

SQ13b. Participant's Phone number #2______________________________________

SQ14. Participant's email address (or mailing address if no email):_______________________

SQ15. Notes from interviewer related to participants' contact information (if any):
____
____
____

PHONE SCREENING QUESTIONNAIRE PART 2

PID. Participant ID #: ______________

INCLUSION CRITERIA: All answers in 3rd column must be YES. If an answer is NO, immediately proceed to question SQ40.			
			Comments
SQ26.	***What is your age?*** [Is age at least 18 years?]	YES NO	
SQ27.	***Can you read and write in English?***	YES NO	

EXCLUSION CRITERIA: All answers in 3rd column must be NO with the exception of SQ32a and SQ33a. If an answer (other than SQ32a and SQ33a) is YES, then immediately proceed to question SQ40.			
			Comments
I will now start to ask some questions about your mental health.			
SQ28.	***Has anyone ever told you that you have bipolar disorder or manic depression?***	**YES NO**	
SQ29.	***Has anyone ever used the word "Borderline" to describe you?***	**YES NO**	
SQ30a.<br><br>SQ30b.	***Have you ever had a hallucination or seen things that other people can't see, or hear things other people can't hear?***<br><br>***Have you ever been diagnosed with schizophrenia or psychosis?***	**YES NO**<br><br>**YES NO**	
 <br>SQ31_1<br>SQ31_2<br>SQ31_3<br>SQ31_4<br>SQ31_5<br>SQ31_6<br>SQ31_7<br>SQ31_8	***Have you ever taken any of the following medications that I am about to read to you?***<br>***Lithium***<br>***Seroquel (quetiapine)***<br>***Abilify (aripiprazole)***<br>***Zyprexa (olanzapine)***<br>***Clozaril (clozapine)***<br>***Haldol/Haloperidol***<br>***Geodon (ziprasidone)***<br>***Risperdal (risperidone)***	 <br>**YES NO DK**<br>**YES NO DK**<br>**YES NO DK**<br>**YES NO DK**<br>**YES NO DK**<br>**YES NO DK**<br>**YES NO DK**<br>**YES NO DK**	
SQ32a<br><br>SQ32b<br><br>SQ32c<br><br><br>SQ32d	***Have you ever had a suicide attempt?***<br><br>***[If yes, ask…] Have you considered killing yourself during the past month?***<br><br>***[If yes, ask…] Are you currently suicidal?***<br><br>***[If yes, keep participant on the phone, and follow suicide safety plan below]***<br><br>***[If no, ask…] Are you getting any help for that? If not, then provide list of resources from Safety Plan including Gateway, Anchor and The Providence Center] Not urgent, but inform Dr. Flynn about what was discussed.***	**YES NO**<br><br>**YES NO**<br><br><br>**YES NO**<br><br><br>**YES NO**	
EXCLUSION CRITERIA: All answers in 3rd column must be NO with the exception of SQ32a and			

SQ33a. If an answer (other than SQ32a and SQ33a) is YES, then immediately proceed to question SQ40.			
SQ33a<br><br>SQ33b<br><br>SQ33c	***Would you say you have a trauma history? [If yes…]***<br>***In the past month, have you had any problems with dissociation (memory loss)?***<br>***In the past month, have you had any flashbacks (i.e. sudden and disturbing vivid memory) about the trauma?***	**YES NO**<br><br>**YES NO**<br><br>**YES NO**	
SQ34a<br><br><br><br><br>SQ34b	***In the past month, have you had any problems with obsessions or compulsions, such as washing your hands or checking the oven over and over again?***<br>***[If yes…] Has anyone diagnosed you with obsessive compulsive disorder?***	**YES NO**<br><br><br><br><br>**YES NO**	
SQ35	***In the past month, have you had a panic attack (i.e. sweating, heart palpitations, nausea, trouble breathing, fear of dying/choking/going crazy)?***	**YES NO**	
SQ36	***Have you had any problems with alcoholism or drug use in the past year?***	**YES NO**	
SQ37	***In the past year, have you had an eating disorder, such as starving, binge eating, or vomiting?***	**YES NO**	
SQ38a<br><br><br>SQ38b<br><br><br><br><br><br><br>SQ38c	***Do you currently have a mindfulness practice, such as meditation or yoga?***<br><br>***Please tell me more about your mindfulness practice, including how often you practice per week.***<br><br><br><br><br>***Do you currently practice meditation more than once per week? (yoga does not count as meditation in this context)***	**YES NO**<br><br><br>**Fill in response in comments section to the right**<br><br>**YES NO**	
SQ39	***This class will take place at Brown University in-person. Do you have any medical or mobility issues that would affect you being able to attend class?***	**YES NO**	
SQ40	Participant qualifies for next step of study (next step is 1st blood pressure screening)	**YES NO**<br><br>**If YES go to SQ41.**<br><br>**If NO, go to SQ42.**	

SQ41a. ***Thank you for taking the time to answer these questions. You qualify for the next stage of screening, which is to take your blood pressure at our office. If you're still interested, we'd like to schedule a time to have you come in to complete the in-person screener. It will only take 30 minutes or less and will involve taking your blood pressure 3-5 times. Is there a day or time that works best for you?*** [Schedule the In-Person Screening visit now]

SQ41b. ***As I mentioned earlier, there is a mindfulness intervention that is part of this study. If you are eligible based on the In-Person screening AND are one of the individuals randomly selected to receive the intervention, you will then be invited to take part in a 9 week Mindfulness Course. We will be scheduling the mindfulness intervention at a time that works best with most of the participants. What days and times of the week typically work best with your schedule? Please keep in mind that the sessions are 2.5 hours long, and take place once per week for 9 weeks.***

[Record participant's availability for Intervention and then proceed with the script]

Thank you for your time and interest in this study. We look forward to meeting you in person on [DATE / TIME]. Do you have any questions before we end this call?

IF INELIGIBLE:

SQ42a. ***Thank you for taking the time to answer these questions. According to the survey, you do not qualify for the study at this time. There may be other studies you quality for.***

SQ42b. ***Would you like me to keep your information to pass on to these studies?*** YES / NO

SQ42c. [If yes…] ***OK, thank you. We will keep this information for future studies you may qualify for. Thank you for taking the time to talk with me today. Can I answer any questions before hanging up?***

SQ42d. [If no…] ***OK, our copy of this information will be destroyed. Thank you for taking the time to talk with me today. Can I answer any questions before hanging up?***

Deleted: ***at two different times at least a week apart from each other. Let's book the in-person screening sessions*** [Book both sessions. We can cancel the second session if they do not qualify].

Deleted:

SAFETY PLAN

Enter details below on paper during screening (These variables should are also be entered in the survey via questions SQ10-SQ13). Destroy this paper after screening is complete.

Participant's First Name: ______________________________________

Participant's Last Name:____

Participant's Address:

Street address: ______________

City: _____________________

State: ________

Zip Code: _________________

Participant's Phone number #1: ______________________________________

Participant's Phone number #2______________________________________

During the phone-based screening, if participants respond yes to "Are you currently suicidal?", the interviewer should perform the following 2 steps:

1. **Immediately have 911 and Dr. Ellen Flynn called by a colleague who has been informed beforehand that this is a possibility.**

Specifically, while keeping the participant on the phone, show the text below in the box to a colleague.

I have a study participant on the phone who is currently suicidal. Please call 911 immediately, and tell them:

"I am calling on behalf of [my name] who is performing a research study at Brown University. He has a participant on the phone who says they are currently suicidal." Please provide the participants' contact information to the 911 operator (i.e. name, address, phone #, email address) as requested. This information is shown above.

Finally, after calling 911, please call Dr. Ellen Flynn, who is the psychiatrist supporting this study. Her cell phone # is 401-258-9829. Please provide her with the same information as was done during the 911 call.

2. **While speaking calmly with the participant, let them know what you are doing. Specifically, let them know we are calling our study's psychiatrist Dr. Flynn and 911, and why you are doing that (i.e. because we are concerned about you). You can speak with participant to keep him/her on the phone, but the discussion should not be clinical in nature.**

Examples of questions that could be asked in order to keep them on the phone:

- "Tell me what is going on."
- "What's happening right now?"
- Tell me more about why you are interesting in being part of this study.
- What are you hoping to get out of this study?

The following information can be provided to study participants if they state they have had considered killing themselves in the past month. If they are currently suicidal, the main priority is to keep them on the phone while 911 and Dr. Flynn are being contacted.

National Suicide Prevention Lifeline: 1-800-273-8255

Other options are to:

- Call your doctor's office
- Call 911 for emergency services
- Go to the nearest hospital emergency room.

Local Non-Urgent Free or Inexpensive Mental Health Services:
Gateway Healthcare: 401-729-8701
Anchor Counseling Center: 401-475-9979
The Providence Center: 401-276-4020

Appendix 5:
Revised In-Person Screener
with Track Changes

(v.1.4 – November 6, 2015)

~~FIRST~~ IN-PERSON ~~VISIT~~ SCREENING ASSESSMENT FORM

PID. Participant ID # ___________
BA2_01. Staff ID # ___________
BA2_02. Today's date (MMDDYY): ___________

Blood Pressure:
BA2_03a. Blood pressure 1st reading, systolic blood pressure: __________ mmHg
BA2_03b. Blood pressure 1st reading, diastolic blood pressure: ___________ mmHg
BA2_03c. Blood pressure 2nd reading, systolic blood pressure: __________ mmHg
BA2_03d. Blood pressure 2nd reading, diastolic blood pressure: ___________ mmHg
BA2_03e. Blood pressure 3rd reading, systolic blood pressure: __________ mmHg
BA2_03f. Blood pressure 3rd reading, diastolic blood pressure: ___________ mmHg
BA2_03g. Blood pressure 4th reading, systolic blood pressure: __________ mmHg
BA2_03h. Blood pressure 4th reading, diastolic blood pressure: ___________ mmHg
BA2_03i. Blood pressure 5th reading, systolic blood pressure: __________ mmHg
BA2_03j. Blood pressure 5th reading, diastolic blood pressure: ___________ mmHg

BA2_04. Were the 4th and 5th systolic blood pressure readings within 20 mmHg of each other?
- □ Yes
- □ No ***(repeat measurements***

BA2_05. Were the 4th and 5th diastolic blood pressure readings within 10 mmHg of each other?
- □ Yes
- □ No ***(repeat measurements)***

BA2_06a. Repeated blood pressure 1st reading, systolic blood pressure: __________ mmHg
BA2_06b. Repeated blood pressure 1st reading, diastolic blood pressure: ___________ mmHg
BA2_06c. Repeated blood pressure 2nd reading, systolic blood pressure: __________ mmHg
BA2_06d. Repeated blood pressure 2nd reading, diastolic blood pressure: ___________ mmHg
BA2_06e. Repeated blood pressure 3rd reading, systolic blood pressure: __________ mmHg
BA2_06f. Repeated blood pressure 3rd reading, diastolic blood pressure: ___________ mmHg
BA2_06g. Repeated blood pressure 4th reading, systolic blood pressure: __________ mmHg
BA2_06h. Repeated blood pressure 4th reading, diastolic blood pressure: ___________ mmHg
BA2_06i. Repeated blood pressure 5th reading, systolic blood pressure: __________ mmHg
BA2_06j. Repeated blood pressure 5th reading, diastolic blood pressure: ___________ mmHg

BA2_07. Blood pressure cuff size used: □ S □ Reg □ L □ XL

BA2_08. Arm that cuff was placed on: □ L □ R

Medications (ME)

ME01. Do you take any prescription medications or over-the-counter drugs?

- ☐ No → ***skip to end of medications questions***
- ☐ Yes
- ☐ Don't know
- ☐ Prefer not to answer

ME02a. What is the name of the first prescription medication or over-the-counter drug that you take?

☐ Label product name: ______________________

☐ Label generic name: ______________________

☐ Don't know ☐ Prefer not to answer

ME02b. What is the dosage form?

Oral
- ☐ Pill, tablet, or capsule
- ☐ Sublingual or orally disintegrating tablet
- ☐ Liquid solution or suspension (drink, syrup)
- ☐ Powder

Topical
- ☐ Liquid, cream, gel, or ointment
- ☐ Ear drops (otic)
- ☐ Eye drops (ophthalmic)
- ☐ Skin patch (transdermal)

Inhaled
- ☐ Inhaler or nebulizer

Injected
- ☐ Injection

Suppository
- ☐ Rectal (e.g., enema)
- ☐ Vaginal (e.g., douche, pessary)

Other:
- ☐ Don't know
- ☐ Prefer not to answer

ME02c. How frequently do you take it?

- ☐ __________ times per day
- ☐ __________ times per week
- ☐ __________ times per month
- ☐ Don't know
- ☐ Prefer not to answer

ME02d. What is the strength? ***(Record strength of how it is actually taken, not how it is prescribed.)***

- ☐ __________ %
- ☐ __________ mg
- ☐ __________ mcg
- ☐ __________ grams
- ☐ __________ I.U.
- ☐ __________ Other unit: __________
- ☐ Don't know
- ☐ Prefer not to answer

Comment [SF1]: In order to reduce participant burden, Medication Use has been moved to the In-Person baseline assessment since it is not part of the eligibility criteria.

ME02e. Total dosage per day. ***(Record strength of how it is actually taken, not how it is prescribed.)***

- ☐ __________ %
- ☐ __________ mg
- ☐ __________ mcg
- ☐ __________ grams
- ☐ __________ I.U.
- ☐ __________ Other unit: ______________
- ☐ Don't know
- ☐ Prefer not to answer

ME02f. Do you take it regularly or only as needed?

- ☐ Regularly
- ☐ Only as needed
- ☐ Don't know
- ☐ Prefer not to answer

ME02g. For how long have you been taking it?

- ☐ For __________ days
- ☐ For __________ weeks
- ☐ For __________ months
- ☐ For __________ years
- ☐ Don't know
- ☐ Prefer not to answer

ME02h. What is the medication used for?

____

ME02i. ***Interviewer comments:***

____

ME02j. Do you take any other prescription medications or over-the-counter drugs?

- ☐ No → ***skip to end of medications questions***
- ☐ Yes
- ☐ Don't know
- ☐ Prefer not to answer

ME03a. What is the name of the next prescription medication or over-the-counter drug that you take?

☐ Label product name: ____

☐ Label generic name: ____

☐ Don't know ☐ Prefer not to answer

ME03b. What is the dosage form?

Oral

- ☐ Pill, tablet, or capsule
- ☐ Sublingual or orally disintegrating tablet
- ☐ Liquid solution or suspension (drink, syrup)
- ☐ Powder

Topical
☐ Liquid, cream, gel, or ointment
☐ Ear drops (otic)
☐ Eye drops (ophthalmic)
☐ Skin patch (transdermal)

Inhaled
☐ Inhaler or nebulizer

Injected
☐ Injection

Suppository
☐ Rectal (e.g., enema)
☐ Vaginal (e.g., douche, pessary)

Other:
☐ Don't know
☐ Prefer not to answer

ME03c. How frequently do you take it?
☐ __________ times per day
☐ __________ times per week
☐ __________ times per month
☐ Don't know
☐ Prefer not to answer

ME03d. What is the strength? ***(Record strength of how it is actually taken, not how it is prescribed.)***

☐ __________ %
☐ __________ mg
☐ __________ mcg
☐ __________ grams
☐ __________ I.U.
☐ __________ Other unit: ______________
☐ Don't know
☐ Prefer not to answer

ME03e. Total dosage per day. ***(Record strength of how it is actually taken, not how it is prescribed.)***
☐ __________ %
☐ __________ mg
☐ __________ mcg
☐ __________ grams
☐ __________ I.U.
☐ __________ Other unit: ______________
☐ Don't know
☐ Prefer not to answer

ME3f. Do you take it regularly or only as needed?
☐ Regularly
☐ Only as needed
☐ Don't know
☐ Prefer not to answer

ME03g. For how long have you been taking it?
☐ For __________ days
☐ For __________ weeks
☐ For __________ months
☐ For __________ years
☐ Don't know
☐ Prefer not to answer

ME03h. What is the medication used for?

____

ME03i. ***Interviewer comments:***

____

ME03j. Do you take any other prescription medications or over the counter drugs?

- ☐ No → ***skip to end of medications questions***
- ☐ Yes
- ☐ Don't know
- ☐ Prefer not to answer

ME04a. What is the name of the next prescription medication or over-the-counter drug that you take?

☐ Label product name: ____

☐ Label generic name: ____

☐ Don't know ☐ Prefer not to answer

ME04b. What is the dosage form?

Oral
- ☐ Pill, tablet, or capsule
- ☐ Sublingual or orally disintegrating tablet
- ☐ Liquid solution or suspension (drink, syrup)
- ☐ Powder

Topical
- ☐ Liquid, cream, gel, or ointment
- ☐ Ear drops (otic)
- ☐ Eye drops (ophthalmic)
- ☐ Skin patch (transdermal)

Inhaled
- ☐ Inhaler or nebulizer

Injected
- ☐ Injection

Suppository
- ☐ Rectal (e.g., enema)
- ☐ Vaginal (e.g., douche, pessary)

Other:
- ☐ Don't know
- ☐ Prefer not to answer

ME04c. How frequently do you take it?

- ☐ __________ times per day
- ☐ __________ times per week
- ☐ __________ times per month
- ☐ Don't know
- ☐ Prefer not to answer

ME04d. What is the strength? ***(Record strength of how it is actually taken, not how it is prescribed.)***

- ☐ __________ %
- ☐ __________ mg
- ☐ __________ mcg
- ☐ __________ grams

☐ __________ I.U. ☐ Don't know
☐ __________ Other unit: ______________ ☐ Prefer not to answer

ME04e. Total dosage **per day.** ***(Record strength of how it is actually taken, not how it is prescribed.)***

☐ __________ %
☐ __________ mg
☐ __________ mcg
☐ __________ grams
☐ __________ I.U.
☐ __________ Other unit: ______________
☐ Don't know
☐ Prefer not to answer

ME04f. Do you take it regularly or only as needed?

☐ Regularly
☐ Only as needed
☐ Don't know
☐ Prefer not to answer

ME04g. For how long have you been taking it?

☐ For __________ days
☐ For __________ weeks
☐ For __________ months
☐ For __________ years
☐ Don't know
☐ Prefer not to answer

ME04h. What is the medication used for?

____

ME04i. ***Interviewer comments:***
____

ME04j. Do you take any other prescription medications or over-the-counter drugs?

☐ No → ***skip to end of medications section***
☐ Yes
☐ Don't know
☐ Prefer not to answer

ME05a. What is the name of the next prescription medication or over-the-counter drug that you take?

☐ Label product name: ____

☐ Label generic name: ____

☐ Don't know ☐ Prefer not to answer

ME05b. What is the dosage form?

Oral

- ☐ Pill, tablet, or capsule
- ☐ Sublingual or orally disintegrating tablet
- ☐ Liquid solution or suspension (drink, syrup)
- ☐ Powder

Topical

- ☐ Liquid, cream, gel, or ointment
- ☐ Ear drops (otic)
- ☐ Eye drops (ophthalmic)
- ☐ Skin patch (transdermal)

Inhaled

- ☐ Inhaler or nebulizer

Injected

- ☐ Injection

Suppository

- ☐ Rectal (e.g., enema)
- ☐ Vaginal (e.g., douche, pessary)

Other:

- ☐ Don't know
- ☐ Prefer not to answer

ME05c. How frequently do you take it?

- ☐ __________ times per day
- ☐ __________ times per week
- ☐ __________ times per month
- ☐ Don't know
- ☐ Prefer not to answer

ME05d. What is the strength? ***(Record strength of how it is actually taken, not how it is prescribed.)***

- ☐ __________ %
- ☐ __________ mg
- ☐ __________ mcg
- ☐ __________ grams
- ☐ __________ I.U.
- ☐ __________ Other unit: ______________
- ☐ Don't know
- ☐ Prefer not to answer

ME05e. Total dosage **per day.** ***(Record strength of how it is actually taken, not how it is prescribed.)***

- ☐ __________ %
- ☐ __________ mg
- ☐ __________ mcg
- ☐ __________ grams
- ☐ __________ I.U.
- ☐ __________ Other unit: ______________
- ☐ Don't know
- ☐ Prefer not to answer

ME05f. Do you take it regularly or only as needed?

- ☐ Regularly
- ☐ Only as needed
- ☐ Don't know
- ☐ Prefer not to answer

ME05g. For how long have you been taking it?

- ☐ For __________ days
- ☐ For __________ weeks
- ☐ For __________ months
- ☐ For __________ years
- ☐ Don't know
- ☐ Prefer not to answer

ME05h. What is the medication used for?

____

ME05i. ***Interviewer comments:***

____

ME05j. Do you take any other prescription medications or over the counter drugs?

- ☐ No → ***skip to end of end of medications questions***
- ☐ Yes
- ☐ Don't know
- ☐ Prefer not to answer

ME06a. What is the name of the next prescription medication or over-the-counter drug that you take?

☐ Label product name: ______________________________

☐ Label generic name: ______________________________

- ☐ Don't know
- ☐ Prefer not to answer

ME06b. What is the dosage form?

Oral
- ☐ Pill, tablet, or capsule
- ☐ Sublingual or orally-disintegrating tablet
- ☐ Liquid solution or suspension (drink, syrup)
- ☐ Powder

Topical
- ☐ Liquid, cream, gel, or ointment
- ☐ Ear drops (otic)
- ☐ Eye drops (ophthalmic)
- ☐ Skin patch (transdermal)

Inhaled
- ☐ Inhaler or nebulizer

Injected
- ☐ Injection

Suppository
- ☐ Rectal (e.g., enema)
- ☐ Vaginal (e.g., douche, pessary)

Other:
- ☐ Don't know
- ☐ Prefer not to answer

ME06c. How frequently do you take it?

- ☐ __________ times per day
- ☐ __________ times per week
- ☐ __________ times per month
- ☐ Don't know
- ☐ Prefer not to answer

ME06d. What is the strength? ***(Record strength of how it is actually taken, not how it is prescribed.)***

☐ ________ %
☐ ________ mg
☐ ________ mcg
☐ ________ grams
☐ ________ I.U.
☐ ________ Other unit: ____________
☐ Don't know
☐ Prefer not to answer

ME06e. Total dosage **per day.** ***(Record strength of how it is actually taken, not how it is prescribed.)***

☐ ________ %
☐ ________ mg
☐ ________ mcg
☐ ________ grams
☐ ________ I.U.
☐ ________ Other unit: ____________
☐ Don't know
☐ Prefer not to answer

ME06f. Do you take it regularly or only as needed?

☐ Regularly
☐ Only as needed
☐ Don't know
☐ Prefer not to answer

ME06g. For how long have you been taking it?

☐ For ________ days
☐ For ________ weeks
☐ For ________ months
☐ For ________ years
☐ Don't know
☐ Prefer not to answer

ME06h. What is the medication used for?

____

ME06i. ***Interviewer comments:***
____

ME06i. Do you take any other prescription medications or over-the-counter drugs?

☐ No → ***skip to end of medications questions***
☐ Yes
☐ Don't know
☐ Prefer not to answer

ME07a. What is the name of the next prescription medication or over-the-counter drug that you take?

____________ ☐ Label product name: ____

____________ ☐ Label generic name: ____

- Don't know
- Prefer not to answer

ME07b. What is the dosage form?

Oral
- Pill, tablet, or capsule
- Sublingual or orally disintegrating tablet
- Liquid solution or suspension (drink, syrup)
- Powder

Topical
- Liquid, cream, gel, or ointment
- Ear drops (otic)
- Eye drops (ophthalmic)
- Skin patch (transdermal)

Inhaled
- Inhaler or nebulizer

Injected
- Injection

Suppository
- Rectal (e.g., enema)
- Vaginal (e.g., douche, pessary)

Other:
- Don't know
- Prefer not to answer

ME07c. How frequently do you take it?
- ________ times per day
- ________ times per week
- ________ times per month
- Don't know
- Prefer not to answer

ME07d. What is the strength? ***(Record strength of how it is actually taken, not how it is prescribed.)***
- ________ %
- ________ mg
- ________ mcg
- ________ grams
- ________ I.U.
- ________ Other unit: ____________
- Don't know
- Prefer not to answer

ME07e. Total dosage **per day. *(Record strength of how it is actually taken, not how it is prescribed.)***
- ________ %
- ________ mg
- ________ mcg
- ________ grams
- ________ I.U.
- ________ Other unit: ____________
- Don't know
- Prefer not to answer

ME07f. Do you take it regularly or only as needed?
- Regularly
- Only as needed
- Don't know
- Prefer not to answer

ME07g. For how long have you been taking it?

- ☐ For __________ days
- ☐ For __________ weeks
- ☐ For __________ months
- ☐ For __________ years
- ☐ Don't know
- ☐ Prefer not to answer

ME07h. What is the medication used for?

____

ME07i. ***Interviewer comments***:

____

ME07j. Do you take any other prescription medications or over-the-counter drugs?

- ☐ No → ***skip to end of medications questions***
- ☐ Yes
- ☐ Don't know
- ☐ Prefer not to answer

ME08a. What is the name of the next prescription medication or over-the-counter drug that you take?

☐ Label product name:

____

☐ Label generic name:

____

☐ Don't know

☐ Prefer not to answer

ME08b. What is the dosage form?

Oral
- ☐ Pill, tablet, or capsule
- ☐ Sublingual or orally disintegrating tablet
- ☐ Liquid solution or suspension (drink, syrup)
- ☐ Powder

Topical
- ☐ Liquid, cream, gel, or ointment
- ☐ Ear drops (otic)
- ☐ Eye drops (ophthalmic)
- ☐ Skin patch (transdermal)

Inhaled
- ☐ Inhaler or nebulizer

Injected
- ☐ Injection

Suppository
- ☐ Rectal (e.g., enema)
- ☐ Vaginal (e.g., douche, pessary)

Other:
- ☐ Don't know
- ☐ Prefer not to answer

ME08c. How frequently do you take it?

- ☐ __________ times per day
- ☐ __________ times per week
- ☐ __________ times per month
- ☐ Don't know

☐ Prefer not to answer

ME08d. What is the strength? *(Record strength of how it is actually taken, not how it is prescribed.)*

☐ __________ %
☐ __________ mg
☐ __________ mcg
☐ __________ grams
☐ __________ I.U.
☐ __________ Other unit: ______________
☐ Don't know
☐ Prefer not to answer

ME08e. Total dosage **per day.** ***(Record strength of how it is actually taken, not how it is prescribed.)***

☐ __________ %
☐ __________ mg
☐ __________ mcg
☐ __________ grams
☐ __________ I.U.
☐ __________ Other unit: ______________
☐ Don't know
☐ Prefer not to answer

ME08f. Do you take it regularly or only as needed?

☐ Regularly
☐ Only as needed
☐ Don't know
☐ Prefer not to answer

ME08g. For how long have you been taking it?

☐ For __________ days
☐ For __________ weeks
☐ For __________ months
☐ For __________ years
☐ Don't know
☐ Prefer not to answer

ME08h. What is the medication used for?

____

ME08i. ***Interviewer comments***:

____

ME08j. Do you take any other prescription medications or over-the-counter drugs?

☐ No → ***skip to end of medications questions***
☐ Yes
☐ Don't know
☐ Prefer not to answer

ME09a. What is the name of the next prescription medication or over-the-counter drug that you take?

☐ Label product name: ____

☐ Label generic name: ____

☐ Don't know ☐ Prefer not to answer

ME09b. What is the dosage form?

Oral
☐ Pill, tablet, or capsule
☐ Sublingual or orally disintegrating tablet
☐ Liquid solution or suspension (drink, syrup)
☐ Powder

Topical
☐ Liquid, cream, gel, or ointment
☐ Ear drops (otic)
☐ Eye drops (ophthalmic)
☐ Skin patch (transdermal)

Inhaled
☐ Inhaler or nebulizer

Injected
☐ Injection

Suppository
☐ Rectal (e.g., enema)
☐ Vaginal (e.g., douche, pessary)

Other:
☐ Don't know
☐ Prefer not to answer

ME09c. How frequently do you take it?

☐ ________ times per day
☐ ________ times per week
☐ ________ times per month

☐ Don't know
☐ Prefer not to answer

ME09d. What is the strength? *(Record strength of how it is actually taken, not how it is prescribed.)*

☐ ________ %
☐ ________ mg
☐ ________ mcg
☐ ________ grams
☐ ________ I.U.

☐ ________ Other unit: ____________
☐ Don't know
☐ Prefer not to answer

ME09e. Total dosage **per day. *(Record strength of how it is actually taken, not how it is prescribed.)***

☐ ________ %
☐ ________ mg
☐ ________ mcg
☐ ________ grams
☐ ________ I.U.

☐ ________ Other unit: ____________
☐ Don't know
☐ Prefer not to answer

ME09f. Do you take it regularly or only as needed?

☐ Regularly ☐ Only as needed

☐ Don't know ☐ Prefer not to answer

ME09g. For how long have you been taking it?

☐ For __________ days
☐ For __________ weeks
☐ For __________ months
☐ For __________ years
☐ Don't know
☐ Prefer not to answer

ME09h. What is the medication used for?

____

ME09i. ***Interviewer comments:***

____

ME09j. Do you take any other prescription medications or over-the-counter drugs?

☐ No → ***skip to end of medications questions***
☐ Yes
☐ Don't know
☐ Prefer not to answer

ME10a. What is the name of the next prescription medication or over-the-counter drug that you take?

☐ Label product name:
____
☐ Label generic name:
____
☐ Don't know ☐ Prefer not to answer

ME10b. What is the dosage form?

Oral
☐ Pill, tablet, or capsule
☐ Sublingual or orally disintegrating tablet
☐ Liquid solution or suspension (drink, syrup)
☐ Powder

Topical
☐ Liquid, cream, gel, or ointment
☐ Ear drops (otic)
☐ Eye drops (ophthalmic)
☐ Skin patch (transdermal)

Inhaled
☐ Inhaler or nebulizer

Injected
☐ Injection

Suppository
☐ Rectal (e.g., enema)
☐ Vaginal (e.g., douche, pessary)

Other:
☐ Don't know
☐ Prefer not to answer

ME10c. How frequently do you take it?

☐ __________ times per day ☐ __________ times per week

☐ ________ times per month ☐ Prefer not to answer
☐ Don't know

ME10d. What is the strength? ***(Record strength of how it is actually taken, not how it is prescribed.)***

☐ ________ %
☐ ________ mg
☐ ________ mcg
☐ ________ grams
☐ ________ I.U.
☐ ________ Other unit: ________
☐ Don't know
☐ Prefer not to answer

ME10e. Total dosage **per day.** ***(Record strength of how it is actually taken, not how it is prescribed.)***

☐ ________ %
☐ ________ mg
☐ ________ mcg
☐ ________ grams
☐ ________ I.U.
☐ ________ Other unit: ________
☐ Don't know
☐ Prefer not to answer

ME10f. Do you take it regularly or only as needed?

☐ Regularly
☐ Only as needed
☐ Don't know
☐ Prefer not to answer

ME10g. For how long have you been taking it?

- ☐ For __________ days
- ☐ For __________ weeks
- ☐ For __________ months
- ☐ For __________ years
- ☐ Don't know
- ☐ Prefer not to answer

ME10h. What is the medication used for?

____

ME10i. ***Interviewer comments***: ____

Appendix 6:
Revised Home Baseline Assessment with Track Changes

(v.1.4 – November 11, 2015)

HOME BASELINE ASSESSMENT

QUESTIONNAIRES ANSWERED BY PARTICIPANTS AT BASELINE (VIA ONLINE OR PAPER FORM)

Questionnaire Table of Contents

Introductory Questions....3
Background Questions....5
Physical Activity....8
Eating Practices....10
Alcohol Consumption....13
Smoking....14
About You....15
Parent's Education....43
Your Childhood Experiences....45
Blood Pressure Medication Use....48
More About You....49
Family History of Hypertension....53
Your Sleep....54

Introduction

We appreciate you taking the time to participate in this research study. These questionnaires will ask a series of questions on various aspects of your health, health behaviours, family, and other life circumstances. It should take approximately one hour. Please keep in mind that you can refuse to answer any questions that you are not comfortable with.

Introductory Questions

IQ1_01. Please enter the 4-digit ID number you were given. ___ ___ ___ ___.

IQ1_02. What is your main reason for participating in this study?

IQ1_03. What do you care about most?

IQ1_04. What gives you the most pleasure in your life?

IQ1_05. What are your greatest worries?

Please list three personal goals you have for taking this mindfulness program:

PG1_01.____
____
____
____
___

PG1_02. ____
____
____
____

PG1_03. ____
____
____
____
___

Background Questions

BQ1_01. How many years old are you?

BQ1_02. Are you Latino or Hispanic?

☐ No → ***skip to B3***
☐ Yes
☐ I do not know
☐ I prefer not to answer

B1_02a. Which of the following represents your family's country of origin? ***(check all that apply)***

☐ Cuba
☐ Mexico
☐ Puerto Rico
☐ Spain
☐ South America
☐ Columbia
☐ Dominican Republic
☐ Other Central American
☐ Other: ______________________
☐ I do not know
☐ I prefer not to answer

BQ1_03. If you were asked to put yourself into only one of these groups, in which one would you place yourself? ***(select one only):***

☐ Asian
☐ Pacific Islander
☐ African American/Black
☐ Caucasian/White
☐ Native American
☐ Other : ______________________
☐ I do not know
☐ I prefer not to answer

BQ1_04. Which of the following best describes your current work situation? ***(select one only)***

☐ Working full-time
☐ Working part-time
☐ Retired
☐ Unemployed: Looking for work
☐ Unemployed: Not currently looking for work
☐ Unemployed due to disability
☐ Keeping house or raising children full-time
☐ Military
☐ Full-time student
☐ Other: ______________________
☐ I do not know
☐ I prefer not to answer

BQ1_05. What is the highest grade or level of regular school you have completed?

☐ Elementary School
☐ Junior High
☐ High School
☐ College
☐ Graduate School
☐ I do not know
☐ I prefer not to answer

BQ1_06. What is the highest degree you earned? ***(select <u>one</u> only)***

☐ Elementary school
☐ Some high school, but no GED
☐ GED
☐ High school
☐ Associate degree (Junior College)
☐ Bachelor's degree
☐ Master's degree
☐ Doctorate (PhD, EdD, etc)
☐ Professional (MD, JD, DDS, DVM, etc.)
☐ Other: ______________________________
☐ I do not know
☐ I prefer not to answer

BQ1_07. Did you ever attend any other school like a technical, vocational, or trade school?

☐ No
☐ Yes
☐ I do not know
☐ I prefer not to answer

BQ1_08. In total, about how many full-time years of education have you had, including 1st grade and all years of school after 1st grade?

________ years

BQ1_09a. Do you currently live alone?

☐ No
☐ Yes → ***skip to next section***
☐ I do not know
☐ I prefer not to answer

Deleted: *BQ_10*

BQ1_09b. How many people currently live in your household, including yourself?

____

BQ1_09c. Of these people, how many are under 18?

____

BQ1_09d. Of the adults in your household (including yourself), how many bring income into the household?

____

Physical Activity

Physical activities are activities where you move and increase your heart rate above its resting rate, whether you do them for pleasure, work, or transportation. The following questions ask about the amount and intensity of physical activity you usually do. The intensity of the activity is related to *the amount of energy* you use doing these activities.

Examples of physical activity intensity levels:

Light activities
Your heart beats slightly faster than normal
You can talk and sing

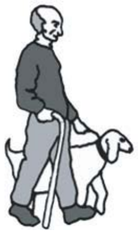
Light exercise

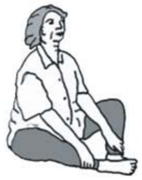
Light stretching

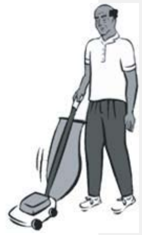
Light vacuuming or yard work

Moderate activities
Your heart beats faster than normal
You can talk but not sing

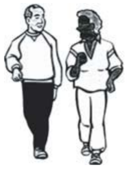
Brisk walking

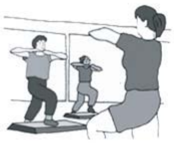
Aerobics class

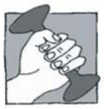
Strength training

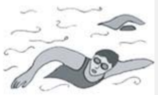
Swim gently

Vigorous activities
Your heart rate increases a lot
You can’t talk, or your talking is broken up by large breaths

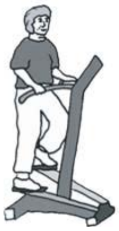
Aerobics classes

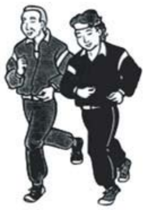
Jogging, Running, or Power Walking

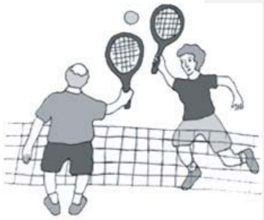
Singles tennis, Racquetball, Pickle ball

How physically active are you?

	YES	NO	I do not know	I prefer not to answer
PA1_01. I rarely or never do any physical activities.	☐	☐	☐	☐
PA1_02. I do some light and/or moderate physical activities, but not every week.	☐	☐	☐	☐
PA1_03. I do some light physical activity every week.	☐	☐	☐	☐
PA1_04. I do moderate physical activity every week but less than 5 days per week or less than 30 minutes on those days.	☐	☐	☐	☐
PA1_05. I do vigorous physical activities every week, but less than 3 days per week or less than 20 minutes on those days.	☐	☐	☐	☐
PA1_06. I do 30 minutes or more per day of moderate physical activities 5 or more days per week.	☐	☐	☐	☐
PA1_07. I do 20 minutes or more per day of vigorous physical activities 3 or more days per week.	☐	☐	☐	☐
PA1_08. I do activities to increase muscle strength, such as lifting weights or calisthenics, once a week or more.	☐	☐	☐	☐
PA1_09. I do activities to improve flexibility, such as stretching or yoga, once a week or more.	☐	☐	☐	☐

Deleted: Diet ¶

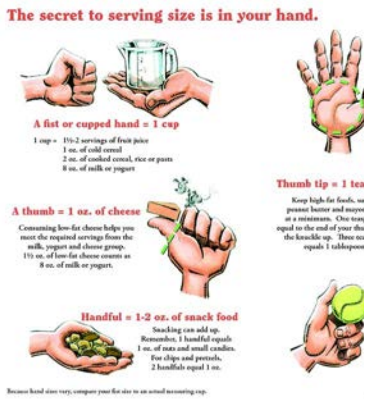

The first set of questions ask about foods you usually eat or drink. For each food, please place an X in the box (e.g. ☒) for the answer that best matches how often you eat or drink it. Remember to count fruit or vegetables that were part of another dish, like a banana on cereal or vegetables in a stew. Also, please pay attention to the serving sizes. For example, one serving size of bread is one slice, so a sandwich would contain 2 servings of bread. Please include all foods you eat, both at home and away from home. Enclosed are pictures of common serving sizes to assist you in filling out your questionnaire. ¶

Deleted: How Often You Eat or Drink This Item ...

Eating Practices

	Definitely True	Mostly True	Mostly False	Definitely False	I Do Not Know	I Prefer Not to Answer
EE1_01. I deliberately take small helpings to control my weight.	☐	☐	☐	☐	☐	☐
EE1_02. I start to eat when I feel anxious.	☐	☐	☐	☐	☐	☐
EE1_03. Sometimes when I start eating, I just can't seem to stop.	☐	☐	☐	☐	☐	☐
EE1_04. When I feel sad, I often eat too much.	☐	☐	☐	☐	☐	☐
EE1_05. I don't eat some foods because they make me fat.	☐	☐	☐	☐	☐	☐
EE1_06. Being with someone who is eating, often makes me want to also eat.	☐	☐	☐	☐	☐	☐
EE1_07. When I feel tense or ‘‘wound up'', I often feel I need to eat.	☐	☐	☐	☐	☐	☐
EE1_08. I often get so hungry that my stomach feels like a bottomless pit.	☐	☐	☐	☐	☐	☐
EE1_09. I'm always so hungry that it's hard for me to stop eating before finishing all of the food on my plate.	☐	☐	☐	☐	☐	☐
EE1_10. When I feel lonely, I console myself by eating.	☐	☐	☐	☐	☐	☐
EE1_11. I consciously hold back on how much I eat at meals to keep from gaining weight.	☐	☐	☐	☐	☐	☐
EE1_12. When I smell a sizzling steak or see a juicy piece of meat, I find it very difficult to keep from eating even if I've just finished a meal.	☐	☐	☐	☐	☐	☐
EE1_13. I'm always hungry enough to eat at any time.	☐	☐	☐	☐	☐	☐
EE1_14. If I feel nervous, I try to calm down by eating.	☐	☐	☐	☐	☐	☐
EE1_15. When I see something that looks very delicious, I often get so hungry that I have to eat right away.	☐	☐	☐	☐	☐	☐
EE1_16. When I feel depressed, I want to eat.	☐	☐	☐	☐	☐	☐

	Almost Never	Seldom	Usually	Almost Always	I Do Not Know	I Prefer Not to Answer
EE1_17. How often do you avoid 'stocking up'' on tempting foods?	☐	☐	☐	☐	☐	☐

	Unlikely	A little likely	Somewhat likely	Very likely	I Do Not Know	I Prefer Not to Answer
EE1_18. How likely are you to make an effort to eat less than you want?	☐	☐	☐	☐	☐	☐

	Never	Rarely	Sometimes	At least once a week	I Do Not Know	I Prefer Not to Answer
EE1_19. Do you go on eating binges even though you're not hungry?	☐	☐	☐	☐	☐	☐

	Only at mealtimes	Sometimes between meals	Often between meals	Almost always	I Do Not Know	I Prefer Not to Answer
EE1_20. How often do you feel hungry?	☐	☐	☐	☐	☐	☐

	1	2	3	4	5	6	7	8	I Do Not Know	I Prefer Not to Answer
EE1_21. On a scale from 1 to 8, where 1 means no restraint in eating and 8 means total restraint, what number would you give yourself? Mark the number that best applies to you:	☐	☐	☐	☐	☐	☐	☐	☐	☐	☐

Table Salt Use:

TS1_01 Please report your average total use, during the past year, of "salt added at the table". Would you say…

- Never .... ☐
- Less than once per month .... ☐
- 1-3 shakes per month .... ☐
- 1 shake per week .... ☐
- 2-4 shakes per week .... ☐
- 5-6 shakes per week .... ☐
- 1 shake per day .... ☐
- 2-3 shakes per day .... ☐
- 4-5 shakes per day .... ☐
- 6+ shakes per day .... ☐

Alcohol Consumption

A drink of alcohol is defined as 1 can or bottle of beer, 1 glass of wine, 1 can or bottle of wine cooler, 1 cocktail, or 1 shot of liquor.

AC1_01. During the past 30 days, how many days per week or per month did you have at least 1 drink of any alcoholic beverage? [if none, ***skip to next section***]

Deleted: *STOP*

____

AC1_02. On the days when you drank, about how many drinks did you drink on average?

____

AC1_03. ***Men:*** Considering all types of alcoholic beverages, how many times during the past 30 days did you have **5** or more drinks on an occasion?

Women: Considering all types of alcoholic beverages, how many times during the past 30 days did you have ***4*** or more drinks on an occasion?

____

Smoking

SM1_01. Have you smoked at least 100 cigarettes in your entire life?

- ☐ Yes
- ☐ No
- ☐ I Do Not Know
- ☐ Prefer not to answer

SM1_02. Did you ever become a daily smoker (that is, smoke every day or nearly every day for two months or longer)?

- ☐ Yes
- ☐ No → ***skip to next section***
- ☐ I Do Not Know
- ☐ Prefer not to answer

Deleted: *SC1_01*

SM1_03. How old were you when you last smoked daily?

Age ______ (in years)

☐ I Do Not Know ☐ Prefer not to answer ☐ Still smoking daily

SM1_04. Do you smoke cigarettes now?

- ☐ Yes
- ☐ No → ***skip to next section***
- ☐ I Do Not Know
- ☐ Prefer not to answer

SM1_04a. How many cigarettes per day do you smoke? (One pack equals 20 cigarettes)

Number of cigarettes ________

- ☐ I Do Not Know
- ☐ Prefer not to answer

About You

Please bring to mind a type of very tasty food that may contribute to hypertension through high salt intake or through eating too many calories (e.g., sweet sugary dessert, salty snack foods, etc.).

Think about the LAST WEEK you MOST WANTED this type of food. For each item, select a number (0 to 10) to indicate your rating.

At that time…	Not at All 0	1	2	3	4	5	6	7	8	9	Extremely 10
1. …how much did you want it?	☐	☐	☐	☐	☐	☐	☐	☐	☐	☐	☐
2. …how much did you need it?	☐	☐	☐	☐	☐	☐	☐	☐	☐	☐	☐
3. …how strong was the urge to have it?	☐	☐	☐	☐	☐	☐	☐	☐	☐	☐	☐
At that time, how vividly did you…											
4. …picture it?	☐	☐	☐	☐	☐	☐	☐	☐	☐	☐	☐
5. …imagine its taste?	☐	☐	☐	☐	☐	☐	☐	☐	☐	☐	☐
6. …imagine its smell?	☐	☐	☐	☐	☐	☐	☐	☐	☐	☐	☐
7. …imagine what it would feel like in your mouth or throat?	☐	☐	☐	☐	☐	☐	☐	☐	☐	☐	☐
8. …imagine how your body would feel?	☐	☐	☐	☐	☐	☐	☐	☐	☐	☐	☐
At that time…											
9. …how hard were you trying not to think about it?	☐	☐	☐	☐	☐	☐	☐	☐	☐	☐	☐
10. …how intrusive were the thoughts?	☐	☐	☐	☐	☐	☐	☐	☐	☐	☐	☐
11. …how hard was it to think about anything else?	☐	☐	☐	☐	☐	☐	☐	☐	☐	☐	☐

Please bring to mind any times in the LAST WEEK when you had a desire to do sedentary activities (e.g., read a book, watch a movie, be on the computer, etc.) instead of physical activities (e.g., walking, gardening, exercise).

Think about the LAST WEEK you MOST WANTED to do a sedentary activity. For each item, select a number (0 to 10) to indicate your rating.

At that time…	Not at All 0	1	2	3	4	5	6	7	8	9	Extremely 10
1. …how much did you want it?	□	□	□	□	□	□	□	□	□	□	□
2. …how much did you need it?	□	□	□	□	□	□	□	□	□	□	□
3. …how strong was the urge to have it?	□	□	□	□	□	□	□	□	□	□	□
At that time, how vividly did you…											
4. …picture it?	□	□	□	□	□	□	□	□	□	□	□
5. …imagine how your body would feel?	□	□	□	□	□	□	□	□	□	□	□
At that time…											
6. …how hard were you trying not to think about it?	□	□	□	□	□	□	□	□	□	□	□
7. …how intrusive were the thoughts?	□	□	□	□	□	□	□	□	□	□	□
8. …how hard was it to think about anything else?	□	□	□	□	□	□	□	□	□	□	□

Please bring to mind any times in the LAST WEEK when you had a desire to drink alcohol, such as wine, beer or spirits.

Think about the LAST WEEK you MOST WANTED alcohol. For each item, select a number (0 to 10) to indicate your rating.

At that time…	Not at All 0	1	2	3	4	5	6	7	8	9	Extremely 10
1. …how much did you want it?	□	□	□	□	□	□	□	□	□	□	□
2. …how much did you need it?	□	□	□	□	□	□	□	□	□	□	□
3. …how strong was the urge to have it?	□	□	□	□	□	□	□	□	□	□	□
At that time, how vividly did you…											
4. …picture it?	□	□	□	□	□	□	□	□	□	□	□
5. …imagine its taste?	□	□	□	□	□	□	□	□	□	□	□
6. …imagine its smell?	□	□	□	□	□	□	□	□	□	□	□
7. …imagine what it would feel like in your mouth or throat?	□	□	□	□	□	□	□	□	□	□	□
8. …imagine how your body would feel?	□	□	□	□	□	□	□	□	□	□	□
At that time…											
9. …how hard were you trying not to think about it?	□	□	□	□	□	□	□	□	□	□	□
10. …how intrusive were the thoughts?	□	□	□	□	□	□	□	□	□	□	□
11. …how hard was it to think about anything else?	□	□	□	□	□	□	□	□	□	□	□

Using the scale provided, please indicate how much each of the following statements reflects how you typically are.

	Not at all	A little	Somewhat	Fair Amount	Very much	I do not know	I prefer not to answer
SC1_01. I am good at resisting temptation.	☐	☐	☐	☐	☐	☐	☐
SC1_02. I have a hard time breaking bad habits.	☐	☐	☐	☐	☐	☐	☐
SC1_03. I am lazy.	☐	☐	☐	☐	☐	☐	☐
SC1_04. I say inappropriate things.	☐	☐	☐	☐	☐	☐	☐
SC1_05. I do certain things that are bad for me, if they are fun.	☐	☐	☐	☐	☐	☐	☐
SC1_06. I refuse things that are bad for me.	☐	☐	☐	☐	☐	☐	☐
SC1_07. I wish I had more self-discipline.	☐	☐	☐	☐	☐	☐	☐
SC1_08. People would say that I have iron self-discipline.	☐	☐	☐	☐	☐	☐	☐
SC1_09. Pleasure and fun sometimes keep me from getting work done.	☐	☐	☐	☐	☐	☐	☐
SC1_10. I have trouble concentrating.	☐	☐	☐	☐	☐	☐	☐
SC1_11. I am able to work effectively toward long-term goals.	☐	☐	☐	☐	☐	☐	☐
SC1_12. Sometimes I can't stop myself from doing something, even if I know it is wrong.	☐	☐	☐	☐	☐	☐	☐
SC1_13. I often act without thinking through all the alternatives.	☐	☐	☐	☐	☐	☐	☐

The next few statements are about certain ways you may feel about your life. Please indicate how strongly you agree or disagree with each of the following statements: Strongly agree; Agree; Neutral, Disagree, or Strongly disagree

	**Strongly agree**	**Agree**	**Neutral**	**Disagree**	**Strongly disagree**	**I Do Not Know**	**Prefer not to answer**
SE1_01. I have little control over the things that happen to me.	☐	☐	☐	☐	☐	☐	☐
SE1_02. There is really no way I can solve some of the problems I have.	☐	☐	☐	☐	☐	☐	☐
SE1_03. There is little I can do to change many of the important things in my life.	☐	☐	☐	☐	☐	☐	☐
SE1_04. I often feel helpless in dealing with the problems of life.	☐	☐	☐	☐	☐	☐	☐
SE1_05. Sometimes I feel that I am being pushed around in life.	☐	☐	☐	☐	☐	☐	☐
SE1_06. What happens to me in the future mostly depends on me.	☐	☐	☐	☐	☐	☐	☐
SE1_07. I can do just about anything I really set my mind to do.	☐	☐	☐	☐	☐	☐	☐

Please read each statement carefully before answering. To the left of each item, indicate how often you behave in the stated manner, using the following scale:

	Almost never	Not very often	Sometimes	Frequently	Almost always	I Do Not Know	Prefer not to answer
CO1_O1. When I fail at something important to me, I become consumed by feelings of inadequacy.							
CO1_02. I try to be understanding and patient towards those aspects of my personality I don't like.							
CO1_O3. When something painful happens I try to take a balanced view of the situation.							
CO1_O4. When I'm feeling down, I tend to feel like most other people are probably happier than I am.							
CO1_O5. I try to see my failings as part of the human condition.							
CO1_O6. When I'm going through a very hard time, I give myself the caring and tenderness I need.							
CO1_O7. When something upsets me I try to keep my emotions in balance.							
CO1_O8. When I fail at something that's important to me, I tend to feel alone in my failure							
CO1_O9. When I'm feeling down I tend to obsess and fixate on everything that's wrong.							

	Almost never	Not very often	Sometimes	Frequently	Almost always	I Do Not Know	Prefer not to answer
CO1_10. When I feel inadequate in some way, I try to remind myself that feelings of inadequacy are shared by most people.							
CO1_11. I'm disapproving and judgmental about my own flaws and inadequacies.							
CO1_12. I'm intolerant and impatient towards those aspects of my personality I don't like.							

The questions in this scale ask you about your feelings and thoughts during the last month. In each case, you will be asked to indicate by circling how often you felt or thought a certain way.

	Never	Almost Never	Sometimes	Fairly Often	Often	I do not know	I prefer not to answer
PS1_01. In the last month, how often have you been upset because of something that happened unexpectedly?	☐	☐	☐	☐	☐	☐	☐
PS1_02. In the last month, how often have you felt that you were unable to control the important things in your life?	☐	☐	☐	☐	☐	☐	☐
PS1_03. In the last month, how often have you felt nervous and "stressed"?	☐	☐	☐	☐	☐	☐	☐
PS1_04. In the last month, how often have you felt confident about your ability to handle your personal problems?	☐	☐	☐	☐	☐	☐	☐
PS1_05. In the last month, how often have you felt that things were going your way?	☐	☐	☐	☐	☐	☐	☐
PS1_06. In the last month, how often have you found that you could not cope with all the things that you had to do?	☐	☐	☐	☐	☐	☐	☐
PS1_07. In the last month, how often have you been able to control irritations in your life?	☐	☐	☐	☐	☐	☐	☐
PS1_08. In the last month, how often have you felt that you were on top of things?	☐	☐	☐	☐	☐	☐	☐
PS1_09. In the last month, how often have you been angered because of things that were outside of your control?	☐	☐	☐	☐	☐	☐	☐
PS1_10. In the last month, how often have you felt difficulties were piling up so high that you could not overcome them?	☐	☐	☐	☐	☐	☐	☐

Please indicate how often the following statements apply to you by checking the box that best describes your experience.

Comment [D1]: This section (ER1) was moved up in the questionnaire. None of the individual items have changed.

	Almost Never (0-10%)	Sometimes (11-35%)	About Half The Time (36-65%)	Most of the Time (66-90%)	Almost Always (91-100%)	I Do Not Know	I Prefer Not to Answer
ER1_01. I am clear about my feelings.	☐	☐	☐	☐	☐	☐	☐
ER1_02. I pay attention to how I feel.	☐	☐	☐	☐	☐	☐	☐
ER1_03. I experience my emotions as overwhelming and out of control.	☐	☐	☐	☐	☐	☐	☐
ER1_04. I have no idea how I am feeling.	☐	☐	☐	☐	☐	☐	☐
ER1_05. I have difficulty making sense out of my feelings.	☐	☐	☐	☐	☐	☐	☐
ER1_06. I am attentive to my feelings.	☐	☐	☐	☐	☐	☐	☐
ER1_07. I know exactly how I am feeling.	☐	☐	☐	☐	☐	☐	☐
ER1_08. I care about what I am feeling.	☐	☐	☐	☐	☐	☐	☐
ER1_09. I am confused about how I feel.	☐	☐	☐	☐	☐	☐	☐
ER1_10. When I'm upset, I acknowledge my emotions.	☐	☐	☐	☐	☐	☐	☐
ER1_11. When I'm upset, I become angry with myself for feeling that way.	☐	☐	☐	☐	☐	☐	☐
ER1_12. When I'm upset, I become embarrassed for feeling that way.	☐	☐	☐	☐	☐	☐	☐

	Almost Never (0-10%)	Sometimes (11-35%)	About Half The Time (36-65%)	Most of the Time (66-90%)	Almost Always (91-100%)	I Do Not Know	I Prefer Not to Answer
ER1_13. When I'm upset, I have difficulty getting work done.	☐	☐	☐	☐	☐	☐	☐
ER1_14. When I'm upset, I become out of control.	☐	☐	☐	☐	☐	☐	☐
ER1_15. When I'm upset, I believe that I will remain that way for a long time.	☐	☐	☐	☐	☐	☐	☐
ER1_16. When I'm upset, I believe that I will end up feeling very depressed.	☐	☐	☐	☐	☐	☐	☐
ER1_17. When I'm upset, I believe that my feelings are valid and important.	☐	☐	☐	☐	☐	☐	☐
ER1_18. When I'm upset, I have difficulty focusing on other things.	☐	☐	☐	☐	☐	☐	☐
ER1_19. When I'm upset, I feel out of control.	☐	☐	☐	☐	☐	☐	☐
ER1_20. When I'm upset, I can still get things done.	☐	☐	☐	☐	☐	☐	☐
ER1_21. When I'm upset, I feel ashamed at myself for feeling that way.	☐	☐	☐	☐	☐	☐	☐
ER1_22. When I'm upset, I know that I can find a way to eventually feel better.	☐	☐	☐	☐	☐	☐	☐

	Almost Never (0-10%)	Sometimes (11-35%)	About Half The Time (36-65%)	Most of the Time (66-90%)	Almost Always (91-100%)	I Do Not Know	I Prefer Not to Answer
ER1_23. When I'm upset, I feel like I am weak.	☐	☐	☐	☐	☐	☐	☐
ER1_24. When I'm upset, I feel like I can remain in control of my behaviours.	☐	☐	☐	☐	☐	☐	☐
ER1_25. When I'm upset, I feel guilty for feeling that way.	☐	☐	☐	☐	☐	☐	☐
ER1_26. When I'm upset, I have difficulty concentrating.	☐	☐	☐	☐	☐	☐	☐
ER1_27. When I'm upset, I have difficulty controlling my behaviours.	☐	☐	☐	☐	☐	☐	☐
ER1_28. When I'm upset, I believe there is nothing I can do to make myself feel better.	☐	☐	☐	☐	☐	☐	☐
ER1_29. When I'm upset, I become irritated at myself for feeling that way.	☐	☐	☐	☐	☐	☐	☐
ER1_30. When I'm upset, I start to feel very bad about myself.	☐	☐	☐	☐	☐	☐	☐
ER1_31. When I'm upset, I believe that wallowing in it is all I can do.	☐	☐	☐	☐	☐	☐	☐
ER1_32. When I'm upset, I lose control over my behaviour.	☐	☐	☐	☐	☐	☐	☐

	Almost Never (0-10%)	Sometimes (11-35%)	About Half The Time (36-65%)	Most of the Time (66-90%)	Almost Always (91-100%)	I Do Not Know	I Prefer Not to Answer
ER1_33. When I'm upset, I have difficulty thinking about anything else.	☐	☐	☐	☐	☐	☐	☐
ER1_34. When I'm upset I take time to figure out what I'm really feeling.	☐	☐	☐	☐	☐	☐	☐
ER1_35. When I'm upset, it takes me a long time to feel better.	☐	☐	☐	☐	☐	☐	☐
ER1_36. When I'm upset, my emotions feel overwhelming.	☐	☐	☐	☐	☐	☐	☐

This scale is made up of a list of statements each of which may or may not be true about you. For each statement, select "definitely true" if you are sure it is true about you and "probably true" if you think it is true but are not absolutely certain. Similarly, you should select "definitely false" if you are sure the statement is false and "probably false" if you think it is false but are not absolutely certain.

Comment [SF2]: NEW section

	Definitely False	Probably False	Probably True	Definitely True	I do not know	I prefer not to answer
**IS1_01** If I wanted to go on a trip for a day (for example, to the country or mountains), I would have a hard time finding someone to go with me.	☐	☐	☐	☐	☐	☐
**IS1_02** I feel that there is no one I can share my most private worries and fears with.	☐	☐	☐	☐	☐	☐
**IS1_03** If I were sick, I could easily find someone to help me with my daily chores.	☐	☐	☐	☐	☐	☐
**IS1_04** There is someone I can turn to for advice about handling problems with my family.	☐	☐	☐	☐	☐	☐
**IS1_05** If I decide one afternoon that I would like to go to a movie that evening, I could easily find someone to go with me.	☐	☐	☐	☐	☐	☐
**IS1_06** When I need suggestions on how to deal with a personal problem, I know someone I can turn to.	☐	☐	☐	☐	☐	☐
**IS1_07** I don't often get invited to do things with others.	☐	☐	☐	☐	☐	☐
**IS1_08** If I had to go out of town for a few weeks, it would be difficult to find someone who would look after my house or apartment (the plants, pets, garden, etc.).	☐	☐	☐	☐	☐	☐
**IS1_09** If I wanted to have lunch with someone, I could easily find someone to join me.	☐	☐	☐	☐	☐	☐

	Definitely False	Probably False	Probably True	Definitely True	I do not know	I prefer not to answer
**IS1_10** If I was stranded 10 miles from home, there is someone I could call who could come and get me.	□	□	□	□	□	□
**IS1_11** If a family crisis arose, it would be difficult to find someone who could give me good advice about how to handle it.	□	□	□	□	□	□
**IS1_12** If I needed some help in moving to a new house or apartment, I would have a hard time finding someone to help me.	□	□	□	□	□	□

Please indicate how often each of the statements below is descriptive of you.

Statement	Never	Rarely	Sometimes	Often	I Do Not Know	Prefer not to answer
LS1_01. I feel in tune with the people around me	☐	☐	☐	☐	☐	s
LS1_02. I lack companionship	☐	☐	☐	☐	☐	☐
LS1_03. There is no one I can turn to	☐	☐	☐	☐	☐	☐
LS1_04. I do not feel alone	☐	☐	☐	☐	☐	☐
LS1_05. I feel part of a group of friends	☐	☐	☐	☐	☐	☐
LS1_06. I have a lot in common with the people around me	☐	☐	☐	☐	☐	☐
LS1_07. I am no longer close to anyone	☐	☐	☐	☐	☐	☐
LS1_08. My interests and ideas are not shared by those around me	☐	☐	☐	☐	☐	☐
LS1_09. I am an outgoing person	☐	☐	☐	☐	☐	☐
LS1_10. There are people I feel close to	☐	☐	☐	☐	☐	☐
LS1_11. I feel left out	☐	☐	☐	☐	☐	☐
LS1_12. My social relationships are superficial	☐	☐	☐	☐	☐	☐
LS1_13. No one really knows me well	☐	☐	☐	☐	☐	☐
LS1_14. I feel isolated from others	☐	☐	☐	☐	☐	☐
LS1_15. I can find companionship when I want it	☐	☐	☐	☐	☐	☐
LS1_16. There are people who really understand me	☐	☐	☐	☐	☐	☐
LS1_17. I am unhappy being so withdrawn	☐	☐	☐	☐	☐	☐
LS1_18. People are around me but not with me	☐	☐	☐	☐	☐	☐
LS1_19. There are people I can talk to	☐	☐	☐	☐	☐	☐
LS1_20. There are people I can turn to	☐	☐	☐	☐	☐	☐

Comment [D3]: This section (LS1) was moved here. None of the individual items have changed.

Deleted: INSTRUCTIONS: I

Below you will find a list of statements. Please indicate how often each statement applies to you generally in daily life.

Comment [D4]: The following three sections (IA1, RD1, and CR1) were moved here from later in the survey. None of the individual items have changed.

	0- Never	1	2	3	4	5 - Alway
IA1_01. When I am tense I notice where the tension is located in my body.	☐	☐	☐	☐	☐	☐
IA1_02. I notice when I am uncomfortable in my body.	☐	☐	☐	☐	☐	☐
IA1_03. I notice where in my body I am comfortable.	☐	☐	☐	☐	☐	☐
IA1_04. I notice changes in my breathing, such as whether it slows down or speeds up.	☐	☐	☐	☐	☐	☐
IA1_05. I do not notice (I ignore) physical tension or discomfort<br>until they become more severe.	☐	☐	☐	☐	☐	☐
IA1_06. I distract myself from sensations of discomfort.	☐	☐	☐	☐	☐	☐
IA1_07. When I feel pain or discomfort, I try to power through it.	☐	☐	☐	☐	☐	☐
IA1_08. When I feel physical pain, I become upset.	☐	☐	☐	☐	☐	☐
IA1_09. I start to worry that something is wrong if I feel any discomfort.	☐	☐	☐	☐	☐	☐
IA1_10. I can notice an unpleasant body sensation without worrying about it.	☐	☐	☐	☐	☐	☐
IA1_11. I can pay attention to my breath without being distracted by things happening around me.	☐	☐	☐	☐	☐	☐
IA1_12. I can maintain awareness of my inner bodily sensations even when there is a lot going on around me.	☐	☐	☐	☐	☐	☐
IA1_13. When I am in conversation with someone, I can pay attention to my posture.	☐	☐	☐	☐	☐	☐
IA1_14. I can return awareness to my body if I am distracted.	☐	☐	☐	☐	☐	☐
IA1_15. I can refocus my attention from thinking to sensing my body.	☐	☐	☐	☐	☐	☐
IA1_16. I can maintain awareness of my whole body even when a part of me is in pain or discomfort.	☐	☐	☐	☐	☐	☐
IA1_17. I am able to consciously focus on my body as a whole.	☐	☐	☐	☐	☐	☐
IA1_18. I notice how my body changes when I am angry.	☐	☐	☐	☐	☐	☐
IA1_19. When something is wrong in my life I can feel it in my body.	☐	☐	☐	☐	☐	☐
IA1_20. I notice that my body feels different after a peaceful experience.	☐	☐	☐	☐	☐	☐
IA1_21. I notice that my breathing becomes free and easy when I feel comfortable.	☐	☐	☐	☐	☐	☐
IA1_22. I notice how my body changes when I feel happy / joyful.	☐	☐	☐	☐	☐	☐

	0- Never	1	2	3	4	5 - Always
IA1_23. When I feel overwhelmed I can find a calm place inside.	☐	☐	☐	☐	☐	☐
IA1_24. When I bring awareness to my body I feel a sense of calm.	☐	☐	☐	☐	☐	☐
IA1_25. I can use my breath to reduce tension.	☐	☐	☐	☐	☐	☐
IA1_26. When I am caught up in thoughts, I can calm my mind by focusing on my body/breathing.	☐	☐	☐	☐	☐	☐
IA1_27. I listen for information from my body about my emotional state.	☐	☐	☐	☐	☐	☐
IA1_28. When I am upset, I take time to explore how my body feels.	☐	☐	☐	☐	☐	☐
IA1_29. I listen to my body to inform me about what to do.	☐	☐	☐	☐	☐	☐
IA1_30. I am at home in my body.	☐	☐	☐	☐	☐	☐
IA1_31. I feel my body is a safe place.	☐	☐	☐	☐	☐	☐
IA1_32. I trust my body sensations.	☐	☐	☐	☐	☐	☐

We are interested in your recent experiences. Below is a list of things that people sometimes experience. Next to each item are five choices: “never”, “rarely”, “sometimes”, “often”, and “all the time”. Please choose one of these to indicate how much you currently have experiences similar to those described.

Deleted: darken

Please do not spend too long on each item–it is your first response that we are interested in. Please be sure to answer every item.

	Never	Rarely	Sometimes	Often	All the time
RD1_01. I think about what will happen in the future.	☐	☐	☐	☐	☐
RD1_02. I remind myself that thoughts aren’t facts.	☐	☐	☐	☐	☐
RD1_03. I am better able to accept myself as I am.	☐	☐	☐	☐	☐
RD1_04. I notice all sorts of little things and details in the world around me.	☐	☐	☐	☐	☐
RD1_05. I am kinder to myself when things go wrong.	☐	☐	☐	☐	☐
RD1_06. I can slow my thinking at times of stress.	☐	☐	☐	☐	☐
RD1_07. I wonder what kind of person I really am.	☐	☐	☐	☐	☐
RD1_08. I am not so easily carried away by my thoughts and feelings.	☐	☐	☐	☐	☐
RD1_09. I notice that I don’t take difficulties so personally.	☐	☐	☐	☐	☐
RD1_10. I can separate myself from my thoughts and feelings.	☐	☐	☐	☐	☐
RD1_11. I analyze why things turn out the way they do.	☐	☐	☐	☐	☐
RD1_12. I can take time to respond to difficulties.	☐	☐	☐	☐	☐
RD1_13. I think over and over again about what others have said to me.	☐	☐	☐	☐	☐
RD1_14. I can treat myself kindly.	☐	☐	☐	☐	☐
RD1_15. I can observe unpleasant feelings without being drawn into them.	☐	☐	☐	☐	☐
RD1_16. I have the sense that I am fully aware of what is going on around me and inside me.	☐	☐	☐	☐	☐
RD1_17. I can actually see that I am not my thoughts.	☐	☐	☐	☐	☐
RD1_18. I am consciously aware of a sense of my body as a whole.	☐	☐	☐	☐	☐
RD1_19. I think about the ways in which I am different from other people.	☐	☐	☐	☐	☐
RD1_20. I view things from a wider perspective.	☐	☐	☐	☐	☐

How do you cope with events? Everyone gets confronted with negative or unpleasant events now and then and everyone responds to them in his or her own way. By the following questions you are asked to indicate what you generally think, when you experience negative or unpleasant events.

	(Almost) Never	Some-times	Regularly	Often	(Almost) Always
CR1_01. I think that I have to accept that this has happened.	□	□	□	□	□
CR1_02. I often think about how I feel about what I have experienced.	□	□	□	□	□
CR1_03. I think I can learn something from the situation.	□	□	□	□	□
CR1_04. I feel that I am the one who is responsible for what has happened.	□	□	□	□	□
CR1_05. I think that I have to accept the situation.	□	□	□	□	□
CR1_06. I am preoccupied with what I think and feel about what I have experienced.	□	□	□	□	□
CR1_07. I think of pleasant things that have nothing to do with it.	□	□	□	□	□
CR1_08. I think that I can become a stronger person as a result of what has happened.	□	□	□	□	□
CR1_09. I keep thinking about how terrible it is what I have experienced.	□	□	□	□	□
CR1_10. I feel that others are responsible for what has happened.	□	□	□	□	□
CR1_11. I think of something nice instead of what has happened.	□	□	□	□	□
CR1_12. I think about how to change the situation.	□	□	□	□	□
CR1_13. I think that it hasn't been too bad compared to other things.	□	□	□	□	□
CR1_14. I think that basically the cause must lie within myself.	□	□	□	□	□
CR1_15. I think about a plan of what I can do best.	□	□	□	□	□
CR1_16. I tell myself that there are worse things in life.	□	□	□	□	□
CR1_17. I continually think how horrible the situation has been.	□	□	□	□	□
CR1_18. I feel that basically the cause lies with others.	□	□	□	□	□

Instructions: Below is a collection of statements about your everyday experience. Using the scale below, please indicate how frequently or infrequently you currently have each experience. Please answer according to what really reflects your experience rather than what you think your experience should be. Please treat each item separately from every other item.

Please indicate the degree to which you agree with each of the following items using the scale below. Simply check your response to each item

	Almost always	Very frequently	Somewhat frequently	Somewhat infrequently	Very infrequently	Almost never	I do not know	I prefer not to answer
MA1_01. I could be experiencing some emotion and not be conscious of it until some time later.	☐	☐	☐	☐	☐	☐	☐	☐
MA1_02. I break or spill things because of carelessness, not paying attention, or thinking of something else.	☐	☐	☐	☐	☐	☐	☐	☐
MA1_03. I find it difficult to stay focused on what's happening in the present.	☐	☐	☐	☐	☐	☐	☐	☐
MA1_04. I tend to walk quickly to get where I'm going without paying attention to what I experience along the way.	☐	☐	☐	☐	☐	☐	☐	☐
MA1_05. I tend not to notice feelings of physical tension or discomfort until they really grab my attention.	☐	☐	☐	☐	☐	☐	☐	☐
MA1_06. I forget a person's name almost as soon as I've been told it for the first time.	☐	☐	☐	☐	☐	☐	☐	☐

	Almost always	Very frequently	Somewhat frequently	Somewhat infrequently	Very infrequently	Almost never	I do not know	I prefer not to answer
MA1_07. It seems I am “running on automatic” without much awareness of what I’m doing.	☐	☐	☐	☐	☐	☐	☐	☐
MA1_08. I rush through activities without being really attentive to them.	☐	☐	☐	☐	☐	☐	☐	☐
MA1_09. I get so focused on the goal I want to achieve that I lose touch with what I am doing right now to get there.	☐	☐	☐	☐	☐	☐	☐	☐
MA1_10. I do jobs or tasks automatically, without being aware of what I’m doing.	☐	☐	☐	☐	☐	☐	☐	☐
MA1_11. I find myself listening to someone with one ear, doing something else at the same time.	☐	☐	☐	☐	☐	☐	☐	☐
MA1_12. I drive places on “automatic pilot” and then wonder why I went there.	☐	☐	☐	☐	☐	☐	☐	☐
MA1_13. I find myself preoccupied with the future or the past.	☐	☐	☐	☐	☐	☐	☐	☐
MA1_14. I find myself doing things without paying attention.	☐	☐	☐	☐	☐	☐	☐	☐
MA1_15. I snack without being aware that I’m eating.	☐	☐	☐	☐	☐	☐	☐	☐

Please rate each of the following statements using the scale provided. Write the number in the blank that best describes *your own opinion* of what is *generally true for you*.

	Never	Almost Never	Sometimes	Fairly Often	Often	I do not know	I prefer not to answer
FF1_01. When I'm walking, I deliberately notice the sensations of my body moving.	☐	☐	☐	☐	☐	☐	☐
FF1_02. I'm good at finding words to describe my feelings.	☐	☐	☐	☐	☐	☐	☐
FF1_03. I criticize myself for having irrational or inappropriate emotions.	☐	☐	☐	☐	☐	☐	☐
FF1_04. I perceive my feelings and emotions without having to react to them.	☐	☐	☐	☐	☐	☐	☐
FF1_05. When I do things, my mind wanders off and I'm easily distracted.	☐	☐	☐	☐	☐	☐	☐
FF1_06. When I take a shower or bath, I stay alert to the sensations of water on my body.	☐	☐	☐	☐	☐	☐	☐
FF1_07. I can easily put my beliefs, opinions, and expectations into words.	☐	☐	☐	☐	☐	☐	☐
FF1_08. I don't pay attention to what I'm doing because I'm daydreaming, worrying, or otherwise distracted.	☐	☐	☐	☐	☐	☐	☐
FF1_09. I watch my feelings without getting lost in them.	☐	☐	☐	☐	☐	☐	☐
FF1_10. I tell myself I shouldn't be feeling the way I'm feeling.	☐	☐	☐	☐	☐	☐	☐
FF1_11. I notice how foods and drinks affect my thoughts, bodily sensations, and emotions.	☐	☐	☐	☐	☐	☐	☐
FF1_12. It's hard for me to find the words to describe what I'm thinking.	☐	☐	☐	☐	☐	☐	☐
FF1_13. I am easily distracted.	☐	☐	☐	☐	☐	☐	☐
FF1_14. I believe some of my thoughts are abnormal or bad and I shouldn't think that way.	☐	☐	☐	☐	☐	☐	☐
FF1_15. I pay attention to sensations, such as the wind in my hair or sun on my face.	☐	☐	☐	☐	☐	☐	☐

	Never	Almost Never	Sometimes	Fairly Often	Often	I do not know	I prefer not to answer
FF1_16. I have trouble thinking of the right words to express how I feel about things.	☐	☐	☐	☐	☐	☐	☐
FF1_17. I make judgments about whether my thoughts are good or bad.	☐	☐	☐	☐	☐	☐	☐
FF1_18. I find it difficult to stay focused on what's happening in the present.	☐	☐	☐	☐	☐	☐	☐
FF1_19. When I have distressing thoughts or images, I "step back" and am aware of the thought or image without getting taken over by it.	☐	☐	☐	☐	☐	☐	☐
FF1_20. I pay attention to sounds, such as clocks ticking, birds chirping, or cars passing.	☐	☐	☐	☐	☐	☐	☐
FF1_21. In difficult situations, I can pause without immediately reacting.	☐	☐	☐	☐	☐	☐	☐
FF1_22. When I have a sensation in my body, it's difficult for me to describe it because I can't find the right words.	☐	☐	☐	☐	☐	☐	☐
FF1_23. It seems I am "running on automatic" without much aware- ness of what I'm doing.	☐	☐	☐	☐	☐	☐	☐
FF1_24. When I have distressing thoughts or images, I feel calm soon after.	☐	☐	☐	☐	☐	☐	☐
FF1_25. I tell myself that I shouldn't be thinking the way I'm thinking.	☐	☐	☐	☐	☐	☐	☐
FF1_26. I notice the smells and aromas of things.	☐	☐	☐	☐	☐	☐	☐
FF1_27. Even when I'm feeling terribly upset, I can find a way to put it into words.	☐	☐	☐	☐	☐	☐	☐
FF1_28. I rush through activities without being really attentive to them.	☐	☐	☐	☐	☐	☐	☐

	Never	Almost Never	Sometimes	Fairly Often	Often	I do not know	I prefer not to answer
FF1_29. When I have distressing thoughts or images, I am able just to notice them without reacting.	☐	☐	☐	☐	☐	☐	☐
FF1_30. I think some of my emotions are bad or inappropriate and I shouldn't feel them.	☐	☐	☐	☐	☐	☐	☐
FF1_31. I notice visual elements in art or nature, such as colors, shapes, textures, or patterns of light and shadow.	☐	☐	☐	☐	☐	☐	☐
FF1_32. My natural tendency is to put my experiences into words.	☐	☐	☐	☐	☐	☐	☐
FF1_33. When I have distressing thoughts or images, I just notice them and let them go.	☐	☐	☐	☐	☐	☐	☐
FF1_34. I do jobs or tasks automatically without being aware of what I'm doing.	☐	☐	☐	☐	☐	☐	☐
FF1_35. When I have distressing thoughts or images, I judge myself as good or bad depending what the thought or image is about.	☐	☐	☐	☐	☐	☐	☐
FF1_36. I pay attention to how my emotions affect my thoughts and behavior.	☐	☐	☐	☐	☐	☐	☐
FF1_37. I can usually describe how I feel at the moment in consider- able detail.	☐	☐	☐	☐	☐	☐	☐
FF1_38. I find myself doing things without paying attention.	☐	☐	☐	☐	☐	☐	☐
FF1_39. I disapprove of myself when I have irrational ideas.	☐	☐	☐	☐	☐	☐	☐

This Week's Experiences

Instructions: Below is a collection of statements about how you find yourself reacting to unpleasant thoughts and feelings. Using the scale below, please indicate how frequently or infrequently you experienced each of the following reactions this week. **Please answer according to what *really reflects your experience this week* rather than what you think your experience should be.**

This week, when I encountered an unpleasant thought or feeling,

	Almost Never	Very Infrequently	Somewhat Infrequently	Somewhat Frequently	Very Frequently	Almost Always	I do not know	I prefer not to answer
MS1_01. I tried to think pleasant thoughts instead	☐	☐	☐	☐	☐	☐	☐	☐
MS1_02. I allowed myself to experience it	☐	☐	☐	☐	☐	☐	☐	☐
MS1_03. I got angry or upset at myself for having the thought/emotion	☐	☐	☐	☐	☐	☐	☐	☐
MS1_04. I felt the desire to make it better or make it go away	☐	☐	☐	☐	☐	☐	☐	☐
MS1_05. I wished I didn't feel or think that way	☐	☐	☐	☐	☐	☐	☐	☐
MS1_06. I tried to let my thoughts just come and go without getting too entangled with them	☐	☐	☐	☐	☐	☐	☐	☐

	Almost Never	Very Infrequently	Somewhat Infrequently	Somewhat Frequently	Very Frequently	Almost Always	I do not know	I prefer not to answer
MS1_07. I thought the mood would never change	☐	☐	☐	☐	☐	☐	☐	☐
MS1_08. I explored the body sensations that accompanied the emotion.	☐	☐	☐	☐	☐	☐	☐	☐
MS1_09. I named the emotion over and over as long as it lasted	☐	☐	☐	☐	☐	☐	☐	☐
MS1_10. I turned to work or other activities to take my mind off things.	☐	☐	☐	☐	☐	☐	☐	☐
MS1_11. I named the emotion and then redirected my attention to the present moment	☐	☐	☐	☐	☐	☐	☐	☐
MS1_12. I thought "This mood, too, shall pass"	☐	☐	☐	☐	☐	☐	☐	☐
MS1_13. I focused on my breathing	☐	☐	☐	☐	☐	☐	☐	☐
MS1_14. I took an active interest in how the experience changed over time	☐	☐	☐	☐	☐	☐	☐	☐

	Almost Never	Very Infrequently	Somewhat Infrequently	Somewhat Frequently	Very Frequently	Almost Always	I do not know	I prefer not to answer
MS1_15. I explored my reactions to the thought or emotion	☐	☐	☐	☐	☐	☐	☐	☐
MS1_16. I thought "my thinking is being distorted by my mood"	☐	☐	☐	☐	☐	☐	☐	☐
MS1_17. Think "Why do I have problems that other people don't have?"	☐	☐	☐	☐	☐	☐	☐	☐
MS1_18. I forgave myself for having the thought/emotion	☐	☐	☐	☐	☐	☐	☐	☐
MS1_19. I challenged the thought's validity	☐	☐	☐	☐	☐	☐	☐	☐
MS1_20. I asked myself questions about the experience in order to explore it better	☐	☐	☐	☐	☐	☐	☐	☐
MS1_21. I tried not to change it because I believe it is important to experience	☐	☐	☐	☐	☐	☐	☐	☐
MS1_22. I labelled my thoughts "images" or verbal "talk"	☐	☐	☐	☐	☐	☐	☐	☐

	Almost Never	Very Infrequently	Somewhat Infrequently	Somewhat Frequently	Very Frequently	Almost Always	I do not know	I prefer not to answer
MS1_23. I thought "Why do I always react this way?"	☐	☐	☐	☐	☐	☐	☐	☐
MS1_24. I adopted a welcoming stance toward the emotion/thought	☐	☐	☐	☐	☐	☐	☐	☐
MS1_25. I went somewhere alone to think about my feelings	☐	☐	☐	☐	☐	☐	☐	☐
MS1_26. I sent out compassion to all people who struggle with this emotion	☐	☐	☐	☐	☐	☐	☐	☐
MS1_27. I restrained myself from doing anything too quickly	☐	☐	☐	☐	☐	☐	☐	☐
MS1_28. I became genuinely curious about the thought/emotion	☐	☐	☐	☐	☐	☐	☐	☐
MS1_29. I laughed or kidded myself about the situation	☐	☐	☐	☐	☐	☐	☐	☐

	Almost Never	Very Infrequently	Somewhat Infrequently	Somewhat Frequently	Very Frequently	Almost Always	I do not know	I prefer not to answer
MS1_30. I treated the repetitive thought like a “top ten radio tune” that's playing in the background	☐	☐	☐	☐	☐	☐	☐	☐
MS1_31. I talked to others about how I was feeling	☐	☐	☐	☐	☐	☐	☐	☐
MS1_32. I tried to forget about it	☐	☐	☐	☐	☐	☐	☐	☐
MS1_33. I thought "Why can't I handle things better"	☐	☐	☐	☐	☐	☐	☐	☐
MS1_34. I tried to come up with a way to make it go away	☐	☐	☐	☐	☐	☐	☐	☐
MS1_35. I said to myself "thinking" or "this is just a thought"	☐	☐	☐	☐	☐	☐	☐	☐
MS1_36. I thought about how much I disliked feeling that way	☐	☐	☐	☐	☐	☐	☐	☐
MS1_37. I reminded myself thoughts are not accurate reflections of reality	☐	☐	☐	☐	☐	☐	☐	☐
MS1_38. I thought that that I must be headed into a downward spiral	☐	☐	☐	☐	☐	☐	☐	☐
MS1_39. I thought "What I am doing to deserve this?	☐	☐	☐	☐	☐	☐	☐	☐

Parent’s Education

CS1_01. Please check the box beside the highest grade or degree that your BIOLOGICAL MOTHER completed.

Never went to school	☐
Grades 1 to 3	☐
Grades 4 to 8	☐
Grades 9 to 11	☐
Grade 12	☐
GED	☐
One or more years of Vocational or Professional School after High School	☐
One or more years of College	☐
One or more years of Graduate or Professional School after College	☐
I Do Not Know	☐
I prefer not to answer	☐

CS1_02. Please check the box beside the highest grade or degree that your BIOLOGICAL FATHER completed.

Never went to school	☐
Grades 1 to 3	☐
Grades 4 to 8	☐
Grades 9 to 11	☐
Grade 12	☐
GED	☐
One or more years of Vocational or Professional School after High School	☐
One or more years of College	☐
One or more years of Graduate or Professional School after College	☐
I Do Not Know	☐
I prefer not to answer	☐

Now please think of the two most important adults in your home between the time you were born and age 18 years. Please check the category below that best described their level of education during this time period.

Deleted: lecel

CS1_03. First adult's highest level of education:

Never went to school	☐
Grades 1 to 3	☐
Grades 4 to 8	☐
Grades 9 to 11	☐
Grade 12	☐
GED	☐
One or more years of Vocational or Professional School after High School	☐
One or more years of College	☐
One or more years of Graduate or Professional School after College	☐
I Do Not Know	☐
I prefer not to answer	☐

CS1_04. Second adult's highest level of education

Never went to school	☐
Grades 1 to 3	☐
Grades 4 to 8	☐
Grades 9 to 11	☐
Grade 12	☐
GED	☐
One or more years of Vocational or Professional School after High School	☐
One or more years of College	☐
One or more years of Graduate or Professional School after College	☐
I Do Not Know	☐
I prefer not to answer	☐

Your Childhood Experiences

The following questions ask about some difficult experiences that you might have had as a child. These questions may be emotionally difficult to answer. Just as a reminder, you do not need answer any questions that you would prefer not to. Your answers to these questions, as with all questions, will remain confidential.

CE1_01. Before you were 18 years old, did a parent or other adult in the household often or very often…

Swear at you, insult you, put you down, or humiliate you?
or
Act in a way that made you afraid that you might be physically hurt?

- □ No
- □ Yes
- □ I do not know
- □ I prefer not to answer

CE1_02. Before you were 18 years old, did a parent or other adult in the household often or very often…

Push, grab, slap, or throw something at you?
or
Ever hit you so hard that you had marks or were injured?

- □ No
- □ Yes
- □ I do not know
- □ I prefer not to answer

CE1_03. Before you were 18 years old, did an adult or person at least 5 years older than you ever…

Touch or fondle you or have you touch their body in a sexual way?
or
Attempt or actually have oral, anal, or vaginal intercourse with you?

- □ No
- □ Yes
- □ I do not know
- □ I prefer not to answer

CE1_04. Before you were 18 years old, did you often or very often feel that …

No one in your family loved you or thought you were important or special?
or
Your family didn't look out for each other, feel close to each other, or support each other?

- □ No
- □ Yes
- □ I do not know
- □ I prefer not to answer

CE1_05. Before you were 18 years old, did you often or very often feel that …

You didn't have enough to eat, had to wear dirty clothes, and had no one to protect you?
or
Your parents were too drunk or high to take care of you or take you to the doctor if you needed it?

- □ No
- □ Yes
- □ I do not know
- □ I prefer not to answer

CE1_06. Before you were 18 years old, was a biological parent ever lost to you through divorce, abandonment, or other reason?

- □ No
- □ Yes
- □ I do not know
- □ I prefer not to answer

CE1_07. Before you were 18 years old, was your mother or stepmother:

Often or very often pushed, grabbed, slapped, or had something thrown at her?
or
Sometimes, often, or very often kicked, bitten, hit with a fist, or hit with something hard?
or
Ever repeatedly hit over at least a few minutes or threatened with a gun or knife?

- □ No
- □ Yes
- □ I do not know
- □ I prefer not to answer

CE1_08. Before you were 18 years old, did you live with anyone who was a problem drinker or alcoholic, or who used street drugs?

- □ No
- □ Yes
- □ I do not know
- □ I prefer not to answer

CE1_09. Before you were 18 years old, was a household member depressed or mentally ill, or did a household member attempt suicide?

- □ No
- □ Yes
- □ I do not know
- □ I prefer not to answer

CE1_10. Before you were 18 years old, did a household member go to prison?

- □ No
- □ Yes
- □ I do not know
- □ I prefer not to answer

Deleted: SN1_01. How many close friends do you have? By close friends, we mean people that you feel at ease with, and that you can talk to about private matters.¶
¶
□ None → ***skip to Y2*** ¶
¶
____ Number of close friends ¶
¶
□ I do not know ¶
□ I prefer not to answer ¶
¶
¶
SN1_01a. How many of these close friends do you see at least once a month? ¶
¶
____ Number of close friends ***(if none, write zero)***¶
¶
□ I do not know ¶
□ I prefer not to answer ¶
¶
¶
SN1_02. Thinking about your relatives, how many relatives do you feel at ease with, and feel that you can talk to about private matters? ¶
¶
□ None → ***skip to Y3***¶
¶
____ Number of relatives ¶
¶
□ I do not know ¶
□ I prefer not to answer ¶
¶
¶
SN1_02a. How many of these relatives do you see at least once a month? ¶
¶
____ Number of relatives ***(if none, write zero)***¶
¶
□ I do not know ¶
□ I prefer not to answer ¶
¶
¶
¶
¶
¶
SN1_03. About how often do you participate in groups or clubs, such as religious connected groups, self-help groups, charities, or a public service or community group.¶
¶
□ Never or almost never¶
□ A few times a year¶
□ Once or twice a month¶
□ Once a week¶
□ More than once a week¶
□ I do not know ¶
□ I prefer not to answer ¶
¶
¶
SN1_04. About how often do you go to religious meetings or services? ¶
¶
□ Never or almost never¶
□ A few times a year¶
□ Once or twice a month¶
□ Once a week¶
□ More than once a week¶
...

Comment [SF5]: New Section

Blood Pressure Medication Use

BM1_01. Do you currently take medication for your blood pressure?
□ Yes
□ No (if responding "no", please skip to the next page).

	Yes	No
BM1_02. Do you sometimes forget to take your blood pressure pills?	□	□
BM1_03. People sometimes miss taking their medications for reasons other than forgetting. Thinking over the past two weeks, were there any days when you did not take your blood pressure medicine?	□	□
BM1_04. Have you ever cut back or stopped taking your blood pressure medicine without telling your doctor because you felt worse when you took it?	□	□
BM1_05. When you travel or leave home, do you sometimes forget to bring along your blood pressure medicine?	□	□
BM1_06. Did you take all your blood pressure medicine yesterday?	□	□
BM1_07. When you feel like your symptoms are under control, do you sometimes stop taking your blood pressure medicine?	□	□
BM1_08. Taking blood pressure medicine every day is a real inconvenience for some people. Do you ever feel hassled about sticking to your treatment plan?	□	□

BM1_09. How often do you have difficulty remembering to take all your blood pressure medicine?
□ Never/rarely
□ Once in a while
□ Sometimes
□ Usually
□ All the time

More About You

Comment [SF6]: New Section – readiness to change

Below are some modifiable factors that likely influence blood pressure. These may not all apply to you, as you may already have excellent levels of these factors.

Physical activity:

The United States Office of Disease Prevention and Health Promotion 2008 Physical Activity Guidelines for Americans states that "Most health benefits occur with at least 150 minutes (2 hours and 30 minutes) a week of moderate intensity physical activity, such as brisk walking. Additional benefits occur with more physical activity. Both aerobic (endurance) and muscle-strengthening (resistance) physical activity are beneficial."

RC1_01. How motivated are you to make changes to your physical activity, using a scale of one to ten, where one = definitely not ready to change, and 10 = definitely ready to change?

Little intention of changing			Mixed feelings towards taking action				Motivated to take action		
1	2	3	4	5	6	7	8	9	10
□	□	□	□	□	□	□	□	□	□

RC1_02. On a scale of 1-10, with 10 being 100% confident, how confident are you that you can make changes in your physical activity?

Not confident			Moderately confident				Confident		
1	2	3	4	5	6	7	8	9	10
□	□	□	□	□	□	□	□	□	□

Diet:

The Dietary Approaches to Stop Hypertension (DASH) diet eating plan is a diet rich in fruits, vegetables, low fat or nonfat dairy. It also includes mostly whole grains; lean meats, fish and poultry; nuts and beans. It is high fiber and low to moderate in fat. It is a plan that follows US guidelines for sodium content, along with vitamins and minerals. It can be considered to be an Americanized version of the Mediterranean diet.

RC1_03. How motivated are you to make changes to your diet to be consistent with the DASH diet, using a scale of one to ten, where one = definitely not ready to change, and 10 = definitely ready to change?

Little intention of changing			Mixed feelings towards taking action				Motivated to take action		
1	2	3	4	5	6	7	8	9	10
□	□	□	□	□	□	□	□	□	□

RC1_04. On a scale of 1-10, with 10 being 100% confident, how confident are you that you can make changes in your diet to be more consistent with the DASH diet?

Not confident			Moderately confident				Confident		
1	2	3	4	5	6	7	8	9	10
□	□	□	□	□	□	□	□	□	□

Salt Intake:

The 2010 Dietary Guidelines for Americans recommend that everyone age 2 years and up should consume less than 2,300 milligrams (mg) of sodium each day. Some groups of people should further limit sodium intake to 1,500 mg per day, including:

- Adults age 51 years or older.
- All African Americans.
- Anyone who has high blood pressure, diabetes, or chronic kidney disease.

RC1_05. How motivated are you to make changes to your salt intake, using a scale of one to ten, where one = definitely not ready to change, and 10 = definitely ready to change?

Little intention of changing			Mixed feelings towards taking action				Motivated to take action		
1	2	3	4	5	6	7	8	9	10
□	□	□	□	□	□	□	□	□	□

RC1_06. On a scale of 1-10, with 10 being 100% confident, how confident are you that you can make changes in your salt intake?

Not confident			Moderately confident				Confident		
1	2	3	4	5	6	7	8	9	10
□	□	□	□	□	□	□	□	□	□

Overweight/Obesity:

Extensive scientific evidence shows that being overweight or obese increases risk of having high blood pressure.

RC1_07. How motivated are you to make changes to your body weight, using a scale of one to ten, where one = definitely not ready to change, and 10 = definitely ready to change?

Little intention of changing			Mixed feelings towards taking action				Motivated to take action		
1	2	3	4	5	6	7	8	9	10
□	□	□	□	□	□	□	□	□	□

RC1_08. On a scale of 1-10, with 10 being 100% confident, how confident are you that you can make changes in your body weight?

Not confident			Moderately confident				Confident		
1	2	3	4	5	6	7	8	9	10
□	□	□	□	□	□	□	□	□	□

Stress, and Stress Response:

Several studies showed that stress, and being slower at emotionally recovering from stressful events, increase risk of hypertension.

RC1_09. How motivated are you to make changes to the amount of stress in your life, or your response to that stress, using a scale of one to ten, where one = definitely not ready to change, and 10 = definitely ready to change?

Little intention of changing			Mixed feelings towards taking action				Motivated to take action		
1	2	3	4	5	6	7	8	9	10
□	□	□	□	□	□	□	□	□	□

RC1_10. On a scale of 1-10, with 10 being 100% confident, how confident are you that you can make changes in the amount of stress in your life, or your response to that stress?

Not confident			Moderately confident				Confident		
1	2	3	4	5	6	7	8	9	10
□	□	□	□	□	□	□	□	□	□

Alcohol Consumption:

Heavy and regular use of alcohol can increase blood pressure substantially. The American Heart Association recommends limiting alcohol consumption to no more than two drinks per day for men and one drink per day for women.

RC1_11. How motivated are you to make changes to the amount of alcohol you consume, using a scale of one to ten, where one = definitely not ready to change, and 10 = definitely ready to change?

Little intention of changing			Mixed feelings towards taking action				Motivated to take action		
1	2	3	4	5	6	7	8	9	10
□	□	□	□	□	□	□	□	□	□

RC1_12. On a scale of 1-10, with 10 being 100% confident, how confident are you that you can make changes in the amount of alcohol you consume?

Not confident			Moderately confident				Confident		
1	2	3	4	5	6	7	8	9	10
□	□	□	□	□	□	□	□	□	□

Blood Pressure (Antihypertensive) Medication Use:

Blood pressure medication has been shown in many studies to be very effective at lowering blood pressure.

RC1_13. How motivated are you to make changes to your blood pressure medication use, using a scale of one to ten, where one = definitely not ready to change, and 10 = definitely ready to change?

Little intention of changing			Mixed feelings towards taking action				Motivated to take action		
1	2	3	4	5	6	7	8	9	10
□	□	□	□	□	□	□	□	□	□

RC1_14. On a scale of 1-10, with 10 being 100% confident, how confident are you that you can make changes your blood pressure medication use?

Not confident			Moderately confident				Confident		
1	2	3	4	5	6	7	8	9	10
□	□	□	□	□	□	□	□	□	□

Family History of Hypertension

Comment [SF7]: New section

FH1_01. Did your biological mother ever have hypertension?

□ No
□ Yes
□ I do not know
□ I prefer not to answer

FH1_02. Did your biological father ever have hypertension?

□ No
□ Yes
□ I do not know
□ I prefer not to answer

FH1_03. How many full brothers and sisters do you have ***(Please include any brothers or sisters who may have died, but do not include half or step brothers and sisters).***

□ I do not have any brothers or sisters → S***kip to the page***
_____ brothers, ________ sisters
□ I do not know
□ I prefer not to answer

FH1_04. Of these brothers and sisters, how many have ever had hypertension?

___________ ***(if none, write 0)***
□ I do not know
□ I prefer not to answer

Your Sleep

Comment [D8]: This section (Sleep) was moved to the end of the survey. Several of the items were dropped to reduce participant burden.

The following questions relate to your usual sleep habits during the past month only. Your answers should indicate the most accurate reply for the majority of days and nights in the past month. ***Please answer all questions.***

SL1_01. **During the past month,** what time have you usually gone to bed at night?

BED TIME ___________ AM / PM

☐ I do not know
☐ I prefer not to answer

SL1_02. **During the past month**, how long (in minutes) has it usually taken you to fall asleep each night?

NUMBER OF MINUTES ___________

☐ I do not know
☐ I prefer not to answer

SL1_03. **During the past month**, at what time have you usually gotten up in the morning?

GETTING UP TIME ___________ AM / PM

☐ I do not know
☐ I prefer not to answer

SL1_04. **During the past month**, how many hours of actual sleep did you get on average at night? (This may be different than the number of hours you spent in bed.)

AVERAGE HOURS OF SL1_EEP PER NIGHT ___________

☐ I do not know
☐ I prefer not to answer

SL1_15. **During the past month**, how would you rate your sleep quality overall?

☐ Very good
☐ Fairly good
☐ Fairly bad
☐ Very bad
☐ I do not know
☐ I prefer not to answer

Deleted: ***For each of the remaining questions, check the one best response. Please answer all questions.***¶
¶
During the past month, how often have you had trouble sleeping because you………….¶
¶
SL1_05. …..Could not get to sleep within 30 minutes?¶
☐ Not during the past month¶
☐ Less than once a week ¶
☐ Once or twice a week¶
☐ Three or more times a week ¶
☐ I do not know¶
☐ I prefer not to answer¶
¶
¶
SL1_06. …..Woke up in the middle of the night or early morning?¶
☐ Not during the past month¶
☐ Less than once a week ¶
☐ Once or twice a week¶
☐ Three or more times a week ¶
☐ I do not know¶
☐ I prefer not to answer ¶
¶
¶
SL1_07. …..Had to get up to use the bathroom?¶
☐ Not during the past month¶
☐ Less than once a week ¶
☐ Once or twice a week¶
☐ Three or more times a week ¶
☐ I do not know¶
☐ I prefer not to answer ¶
¶
¶
...

Deleted: SL1_16. **During the past month**, how often have you taken medicine to help you sleep (prescribed or "over the counter")?¶
¶
☐ Not during the past month¶
☐ Less than once a week ¶
☐ Once or twice a week¶
☐ Three or more times a week ¶
☐ I do not know¶
☐ I prefer not to answer ¶
¶
SL1_17. **During the past month**, how often have you had trouble staying awake while driving, eating meals, or engaging in social activity?¶
¶
☐ Not during the past month¶
☐ Less than once a week ¶
☐ Once or twice a week¶
☐ Three or more times a week ¶
☐ I do not know¶
☐ I prefer not to answer ¶
¶
¶
SL1_18. **During the past month**, how much of a problem has it been for you to keep up enough enthusiasm to get things done?¶
¶
☐ No problem at all¶
☐ Somewhat of a problem¶
☐ A very big problem¶
☐ I do not know¶
☐ I prefer not to answer ¶
¶
¶
...

Thank you for completing this survey!

Please note that these responses will not be seen immediately. Resources are shown below if you feel that you would like to talk with someone immediately for assistance.

National Suicide Prevention Lifeline: 1-800-273-8255
National Sexual Assault Hotline: 1-800-656-4673

Other options are to:

- Call your doctor’s office
- Call 911 for emergency services
- Go to the nearest hospital emergency room.

Appendix 7:
Revised In-Person Baseline Assessment with Track Changes

(v.1.4 – November 6, 2015)

SECOND IN-PERSON VISIT SCREENING BASELINE

QUESTIONNAIRE AND ASSESSMENT FORMS

PID. Participant ID # ___________
BA01. Staff ID # ___________
BA02. Today's date (MMDDYY): ___________

Blood Pressure:
BA03a. Blood pressure 1st reading, systolic blood pressure: __________ mmHg
BA03b. Blood pressure 1st reading, diastolic blood pressure: ___________ mmHg
BA03c. Blood pressure 2nd reading, systolic blood pressure: __________ mmHg
BA03d. Blood pressure 2nd reading, diastolic blood pressure: ___________ mmHg
BA03e. Blood pressure 3rd reading, systolic blood pressure: __________ mmHg
BA03f. Blood pressure 3rd reading, diastolic blood pressure: ___________ mmHg
BA03g. Blood pressure 4th reading, systolic blood pressure: __________ mmHg
BA03h. Blood pressure 4th reading, diastolic blood pressure: ___________ mmHg
BA03i. Blood pressure 5th reading, systolic blood pressure: __________ mmHg
BA03j. Blood pressure 5th reading, diastolic blood pressure: ___________ mmHg

BA04. Were the 4th and 5th systolic blood pressure readings within 20 mmHg of each other?
- □ Yes
- □ No ***(repeat measurements***

BA05. Were the 4th and 5th diastolic blood pressure readings within 10 mmHg of each other?
- □ Yes
- □ No ***(repeat measurements)***

BA06a. Repeated blood pressure 1st reading, systolic blood pressure: __________ mmHg
BA06b. Repeated blood pressure 1st reading, diastolic blood pressure: ___________ mmHg
BA06c. Repeated blood pressure 2nd reading, systolic blood pressure: __________ mmHg
BA06d. Repeated blood pressure 2nd reading, diastolic blood pressure: ___________ mmHg
BA06e. Repeated blood pressure 3rd reading, systolic blood pressure: __________ mmHg
BA06f. Repeated blood pressure 3rd reading, diastolic blood pressure: ___________ mmHg
BA06g. Repeated blood pressure 4th reading, systolic blood pressure: __________ mmHg
BA06h. Repeated blood pressure 4th reading, diastolic blood pressure: ___________ mmHg
BA06i. Repeated blood pressure 5th reading, systolic blood pressure: __________ mmHg
BA06j. Repeated blood pressure 5th reading, diastolic blood pressure: ___________ mmHg

BA07. Blood pressure cuff size used: □ S □ Reg □ L □ XL

BA08. Arm that cuff was placed on: □ L □ R

BA09. Height: _______ . __ cm (one decimal place)

BA10. Weight: _______ . __ kg (one decimal place)

Waist Circumference:
BA11a. Measurement 1 _______ . __ cm (one decimal place)
BA11b. Measurement 2 _______ . __ cm (one decimal place)

BA12. Is the difference between Measurement 1 and Measurement 2 greater than 1.0cm?
□ Yes ***(repeat measurements 3&4)***
□ No

BA13a. Measurement 3 _______ . __ cm
BA13b. Measurement 4 _______ . __ cm

Medications (ME)

Comment [SF1]: Moved here from In-Person Screener

ME01. Do you take any prescription medications or over-the-counter drugs?

- ☐ No → ***skip to end of medications questions***
- ☐ Yes
- ☐ Don't know
- ☐ Prefer not to answer

ME02a. What is the name of the first prescription medication or over-the-counter drug that you take?

☐ Label product name: ______________________________

☐ Label generic name: ______________________________

☐ Don't know ☐ Prefer not to answer

ME02b. What is the dosage form?

Oral
- ☐ Pill, tablet, or capsule
- ☐ Sublingual or orally-disintegrating tablet
- ☐ Liquid solution or suspension (drink, syrup)
- ☐ Powder

Topical
- ☐ Liquid, cream, gel, or ointment
- ☐ Ear drops (otic)
- ☐ Eye drops (ophthalmic)
- ☐ Skin patch (transdermal)

Inhaled
- ☐ Inhaler or nebulizer

Injected
- ☐ Injection

Suppository
- ☐ Rectal (e.g., enema)
- ☐ Vaginal (e.g., douche, pessary)

Other:
- ☐ Don't know
- ☐ Prefer not to answer

ME02c. How frequently do you take it?

- ☐ ________ times per day
- ☐ ________ times per week
- ☐ ________ times per month
- ☐ Don't know
- ☐ Prefer not to answer

ME02d. What is the strength? ***(Record strength of how it is actually taken, not how it is prescribed.)***

- ☐ ________ %
- ☐ ________ mg
- ☐ ________ mcg
- ☐ ________ grams
- ☐ ________ I.U.
- ☐ ________ Other unit: ________
- ☐ Don't know
- ☐ Prefer not to answer

ME02e. Total dosage **per day. *(Record strength of how it is actually taken, not how it is prescribed.)***

- ☐ ________ %
- ☐ ________ mg
- ☐ ________ mcg
- ☐ ________ grams
- ☐ ________ I.U.
- ☐ ________ Other unit: ________
- ☐ Don't know
- ☐ Prefer not to answer

ME02f. Do you take it regularly or only as needed?

- ☐ Regularly
- ☐ Only as needed
- ☐ Don't know
- ☐ Prefer not to answer

ME02g. For how long have you been taking it?

- ☐ For ________ days
- ☐ For ________ weeks
- ☐ For ________ months
- ☐ For ________ years
- ☐ Don't know
- ☐ Prefer not to answer

ME02h. What is the medication used for?

____

ME02i. ***Interviewer comments*:**

____

ME02j. Do you take any other prescription medications or over-the-counter drugs?

- ☐ No → ***skip to end of medications questions***
- ☐ Yes
- ☐ Don't know
- ☐ Prefer not to answer

ME03a. What is the name of the next prescription medication or over-the-counter drug that you take?

☐ Label product name: ____________________

☐ Label generic name: ____________________

☐ Don't know ☐ Prefer not to answer

ME03b. What is the dosage form?

Oral
☐ Pill, tablet, or capsule
☐ Sublingual or orally-disintegrating tablet
☐ Liquid solution or suspension (drink, syrup)
☐ Powder

Topical
☐ Liquid, cream, gel, or ointment
☐ Ear drops (otic)
☐ Eye drops (ophthalmic)
☐ Skin patch (transdermal)

Inhaled
☐ Inhaler or nebulizer

Injected
☐ Injection

Suppository
☐ Rectal (e.g., enema)
☐ Vaginal (e.g., douche, pessary)

Other:
☐ Don't know
☐ Prefer not to answer

ME03c. How frequently do you take it?

☐ ________ times per day
☐ ________ times per week
☐ ________ times per month

☐ Don't know
☐ Prefer not to answer

ME03d. What is the strength? ***(Record strength of how it is actually taken, not how it is prescribed.)***

☐ ________ %
☐ ________ mg
☐ ________ mcg
☐ ________ grams
☐ ________ I.U.

☐ ________ Other unit: ________
☐ Don't know
☐ Prefer not to answer

ME03e. Total dosage **per day.** ***(Record strength of how it is actually taken, not how it is prescribed.)***

- ☐ __________ %
- ☐ __________ mg
- ☐ __________ mcg
- ☐ __________ grams
- ☐ __________ I.U.
- ☐ __________ Other unit: __________
- ☐ Don't know
- ☐ Prefer not to answer

ME3f. Do you take it regularly or only as needed?

- ☐ Regularly
- ☐ Only as needed
- ☐ Don't know
- ☐ Prefer not to answer

ME03g. For how long have you been taking it?

- ☐ For __________ days
- ☐ For __________ weeks
- ☐ For __________ months
- ☐ For __________ years
- ☐ Don't know
- ☐ Prefer not to answer

ME03h. What is the medication used for?

____

ME03i. ***Interviewer comments*:**

____

ME03j. Do you take any other prescription medications or over-the-counter drugs?

- ☐ No → ***skip to end of medications questions***
- ☐ Yes
- ☐ Don't know
- ☐ Prefer not to answer

ME04a. What is the name of the next prescription medication or over-the-counter drug that you take?

☐ Label product name: ____

☐ Label generic name: ____

☐ Don't know ☐ Prefer not to answer

ME04b. What is the dosage form?

Oral
- ☐ Pill, tablet, or capsule
- ☐ Sublingual or orally-disintegrating tablet
- ☐ Liquid solution or suspension (drink, syrup)
- ☐ Powder

Topical
- ☐ Liquid, cream, gel, or ointment
- ☐ Ear drops (otic)
- ☐ Eye drops (ophthalmic)
- ☐ Skin patch (transdermal)

Inhaled
- ☐ Inhaler or nebulizer

Injected
- ☐ Injection

Suppository
- ☐ Rectal (e.g., enema)
- ☐ Vaginal (e.g., douche, pessary)

Other:
- ☐ Don't know
- ☐ Prefer not to answer

ME04c. How frequently do you take it?
- ☐ ___________ times per day
- ☐ ___________ times per week
- ☐ ___________ times per month
- ☐ Don't know
- ☐ Prefer not to answer

ME04d. What is the strength? ***(Record strength of how it is actually taken, not how it is prescribed.)***
- ☐ ___________ %
- ☐ ___________ mg
- ☐ ___________ mcg
- ☐ ___________ grams
- ☐ ___________ I.U.
- ☐ ___________ Other unit: ___________
- ☐ Don't know
- ☐ Prefer not to answer

ME04e. Total dosage **per day**. ***(Record strength of how it is actually taken, not how it is prescribed.)***
- ☐ ___________ %
- ☐ ___________ mg
- ☐ ___________ mcg
- ☐ ___________ grams
- ☐ ___________ I.U.
- ☐ ___________ Other unit: ___________
- ☐ Don't know
- ☐ Prefer not to answer

ME04f. Do you take it regularly or only as needed?
- ☐ Regularly
- ☐ Only as needed
- ☐ Don't know
- ☐ Prefer not to answer

ME04g. For how long have you been taking it?

- ☐ For __________ days
- ☐ For __________ weeks
- ☐ For __________ months
- ☐ For __________ years
- ☐ Don't know
- ☐ Prefer not to answer

ME04h. What is the medication used for?

____

ME04i. ***Interviewer comments***:

____

ME04j. Do you take any other prescription medications or over-the-counter drugs?

- ☐ No → ***skip to end of medications section***
- ☐ Yes
- ☐ Don't know
- ☐ Prefer not to answer

ME05a. What is the name of the next prescription medication or over-the-counter drug that you take?

☐ Label product name: ____

☐ Label generic name: ____

☐ Don't know ☐ Prefer not to answer

ME05b. What is the dosage form?

Oral

- ☐ Pill, tablet, or capsule
- ☐ Sublingual or orally-disintegrating tablet
- ☐ Liquid solution or suspension (drink, syrup)
- ☐ Powder

Topical

- ☐ Liquid, cream, gel, or ointment
- ☐ Ear drops (otic)
- ☐ Eye drops (ophthalmic)
- ☐ Skin patch (transdermal)

Inhaled

- ☐ Inhaler or nebulizer

Injected

- ☐ Injection

Suppository

- ☐ Rectal (e.g., enema)
- ☐ Vaginal (e.g., douche, pessary)

Other:

- ☐ Don't know
- ☐ Prefer not to answer

ME05c. How frequently do you take it?

- ☐ ________ times per day
- ☐ ________ times per week
- ☐ ________ times per month
- ☐ Don't know
- ☐ Prefer not to answer

ME05d. What is the strength? ***(Record strength of how it is actually taken, not how it is prescribed.)***

- ☐ ________ %
- ☐ ________ mg
- ☐ ________ mcg
- ☐ ________ grams
- ☐ ________ I.U.
- ☐ ________ Other unit: ________
- ☐ Don't know
- ☐ Prefer not to answer

ME05e. Total dosage **per day. *(Record strength of how it is actually taken, not how it is prescribed.)***

- ☐ ________ %
- ☐ ________ mg
- ☐ ________ mcg
- ☐ ________ grams
- ☐ ________ I.U.
- ☐ ________ Other unit: ________
- ☐ Don't know
- ☐ Prefer not to answer

ME05f. Do you take it regularly or only as needed?

- ☐ Regularly
- ☐ Only as needed
- ☐ Don't know
- ☐ Prefer not to answer

ME05g. For how long have you been taking it?

- ☐ For ________ days
- ☐ For ________ weeks
- ☐ For ________ months
- ☐ For ________ years
- ☐ Don't know
- ☐ Prefer not to answer

ME05h. What is the medication used for?

____

ME05i. ***Interviewer comments*:**

____

ME05j. Do you take any other prescription medications or over-the-counter drugs?

- ☐ No → ***skip to end of end of medications questions***
- ☐ Yes
- ☐ Don't know
- ☐ Prefer not to answer

ME06a. What is the name of the next prescription medication or over-the-counter drug that you take?

☐ Label product name: ____________________

☐ Label generic name: ____________________

☐ Don't know

☐ Prefer not to answer

ME06b. What is the dosage form?

Oral
- ☐ Pill, tablet, or capsule
- ☐ Sublingual or orally-disintegrating tablet
- ☐ Liquid solution or suspension (drink, syrup)
- ☐ Powder

Topical
- ☐ Liquid, cream, gel, or ointment
- ☐ Ear drops (otic)
- ☐ Eye drops (ophthalmic)
- ☐ Skin patch (transdermal)

Inhaled
- ☐ Inhaler or nebulizer

Injected
- ☐ Injection

Suppository
- ☐ Rectal (e.g., enema)
- ☐ Vaginal (e.g., douche, pessary)

Other:
- ☐ Don't know
- ☐ Prefer not to answer

ME06c. How frequently do you take it?
- ☐ ________ times per day
- ☐ ________ times per week
- ☐ ________ times per month
- ☐ Don't know
- ☐ Prefer not to answer

ME06d. What is the strength? ***(Record strength of how it is actually taken, not how it is prescribed.)***
- ☐ ________ %
- ☐ ________ mg
- ☐ ________ mcg
- ☐ ________ grams
- ☐ ________ I.U.
- ☐ ________ Other unit: ________
- ☐ Don't know
- ☐ Prefer not to answer

ME06e. Total dosage **per day. *(Record strength of how it is actually taken, not how it is prescribed.)***

- ☐ __________ %
- ☐ __________ mg
- ☐ __________ mcg
- ☐ __________ grams
- ☐ __________ I.U.
- ☐ __________ Other unit: ____________
- ☐ Don't know
- ☐ Prefer not to answer

ME06f. Do you take it regularly or only as needed?

- ☐ Regularly
- ☐ Only as needed
- ☐ Don't know
- ☐ Prefer not to answer

ME06g. For how long have you been taking it?

- ☐ For __________ days
- ☐ For __________ weeks
- ☐ For __________ months
- ☐ For __________ years
- ☐ Don't know
- ☐ Prefer not to answer

ME06h. What is the medication used for?

____

ME06i. ***Interviewer comments*:**

____

ME06i. Do you take any other prescription medications or over-the-counter drugs?

- ☐ No → ***skip to end of medications questions***
- ☐ Yes
- ☐ Don't know
- ☐ Prefer not to answer

ME07a. What is the name of the next prescription medication or over-the-counter drug that you take?

☐ Label product name: ____

☐ Label generic name: ____

- ☐ Don't know
- ☐ Prefer not to answer

ME07b. What is the dosage form?

Oral
- ☐ Pill, tablet, or capsule
- ☐ Sublingual or orally-disintegrating tablet
- ☐ Liquid solution or suspension (drink, syrup)
- ☐ Powder

Topical
- ☐ Liquid, cream, gel, or ointment
- ☐ Ear drops (otic)
- ☐ Eye drops (ophthalmic)
- ☐ Skin patch (transdermal)

Inhaled
- ☐ Inhaler or nebulizer

Injected
- ☐ Injection

Suppository
- ☐ Rectal (e.g., enema)
- ☐ Vaginal (e.g., douche, pessary)

Other:
- ☐ Don't know
- ☐ Prefer not to answer

ME07c. How frequently do you take it?
- ☐ __________ times per day
- ☐ __________ times per week
- ☐ __________ times per month
- ☐ Don't know
- ☐ Prefer not to answer

ME07d. What is the strength? ***(Record strength of how it is actually taken, not how it is prescribed.)***
- ☐ __________ %
- ☐ __________ mg
- ☐ __________ mcg
- ☐ __________ grams
- ☐ __________ I.U.
- ☐ __________ Other unit: __________
- ☐ Don't know
- ☐ Prefer not to answer

ME07e. Total dosage **per day. *(Record strength of how it is actually taken, not how it is prescribed.)***
- ☐ __________ %
- ☐ __________ mg
- ☐ __________ mcg
- ☐ __________ grams
- ☐ __________ I.U.
- ☐ __________ Other unit: __________
- ☐ Don't know
- ☐ Prefer not to answer

ME07f. Do you take it regularly or only as needed?
- ☐ Regularly
- ☐ Only as needed
- ☐ Don't know
- ☐ Prefer not to answer

ME07g For how long have you been taking it?

- ☐ For ________ days
- ☐ For ________ weeks
- ☐ For ________ months
- ☐ For ________ years
- ☐ Don't know
- ☐ Prefer not to answer

ME07h. What is the medication used for?

____

ME07i. ***Interviewer comments***:

____

ME07j. Do you take any other prescription medications or over-the-counter drugs?

- ☐ No → ***skip to end of medications questions***
- ☐ Yes
- ☐ Don't know
- ☐ Prefer not to answer

ME08a. What is the name of the next prescription medication or over-the-counter drug that you take?

- ☐ Label product name: ____
- ☐ Label generic name: ____
- ☐ Don't know
- ☐ Prefer not to answer

ME08b. What is the dosage form?

Oral
- ☐ Pill, tablet, or capsule
- ☐ Sublingual or orally-disintegrating tablet
- ☐ Liquid solution or suspension (drink, syrup)
- ☐ Powder

Topical
- ☐ Liquid, cream, gel, or ointment
- ☐ Ear drops (otic)
- ☐ Eye drops (ophthalmic)
- ☐ Skin patch (transdermal)

Inhaled
- ☐ Inhaler or nebulizer

Injected
- ☐ Injection

Suppository
- ☐ Rectal (e.g., enema)
- ☐ Vaginal (e.g., douche, pessary)

Other:
- ☐ Don't know
- ☐ Prefer not to answer

ME08c. How frequently do you take it?

- ☐ ________ times per day
- ☐ ________ times per week
- ☐ ________ times per month
- ☐ Don't know
- ☐ Prefer not to answer

ME08d. What is the strength? *(Record strength of how it is actually taken, not how it is prescribed.)*

- ☐ ________ %
- ☐ ________ mg
- ☐ ________ mcg
- ☐ ________ grams
- ☐ ________ I.U.
- ☐ ________ Other unit: ________
- ☐ Don't know
- ☐ Prefer not to answer

ME08e. Total dosage **per day. *(Record strength of how it is actually taken, not how it is prescribed.)***

- ☐ ________ %
- ☐ ________ mg
- ☐ ________ mcg
- ☐ ________ grams
- ☐ ________ I.U.
- ☐ ________ Other unit: ________
- ☐ Don't know
- ☐ Prefer not to answer

ME08f. Do you take it regularly or only as needed?

- ☐ Regularly
- ☐ Only as needed
- ☐ Don't know
- ☐ Prefer not to answer

ME08g. For how long have you been taking it?

- ☐ For ________ days
- ☐ For ________ weeks
- ☐ For ________ months
- ☐ For ________ years
- ☐ Don't know
- ☐ Prefer not to answer

ME08h. What is the medication used for?

____

ME08i. ***Interviewer comments***:

____

ME08j. Do you take any other prescription medications or over-the-counter drugs?

- ☐ No → ***skip to end of medications questions***
- ☐ Yes
- ☐ Don't know
- ☐ Prefer not to answer

ME09a. What is the name of the next prescription medication or over-the-counter drug that you take?

☐ Label product name: ____________________

☐ Label generic name: ____________________

☐ Don't know ☐ Prefer not to answer

ME09b. What is the dosage form?

Oral
☐ Pill, tablet, or capsule
☐ Sublingual or orally-disintegrating tablet
☐ Liquid solution or suspension (drink, syrup)
☐ Powder

Topical
☐ Liquid, cream, gel, or ointment
☐ Ear drops (otic)
☐ Eye drops (ophthalmic)
☐ Skin patch (transdermal)

Inhaled
☐ Inhaler or nebulizer

Injected
☐ Injection

Suppository
☐ Rectal (e.g., enema)
☐ Vaginal (e.g., douche, pessary)

Other:
☐ Don't know
☐ Prefer not to answer

ME09c. How frequently do you take it?

☐ ________ times per day
☐ ________ times per week
☐ ________ times per month

☐ Don't know
☐ Prefer not to answer

ME09d. What is the strength? *(Record strength of how it is actually taken, not how it is prescribed.)*

☐ ________ %
☐ ________ mg
☐ ________ mcg
☐ ________ grams
☐ ________ I.U.

☐ ________ Other unit: ________
☐ Don't know
☐ Prefer not to answer

ME09e. Total dosage **per day. *(Record strength of how it is actually taken, not how it is prescribed.)***

☐ ________ %
☐ ________ mg
☐ ________ mcg
☐ ________ grams
☐ ________ I.U.
☐ ________ Other unit: ________
☐ Don't know
☐ Prefer not to answer

ME09f. Do you take it regularly or only as needed?

☐ Regularly
☐ Only as needed
☐ Don't know
☐ Prefer not to answer

ME09g. For how long have you been taking it?

☐ For ________ days
☐ For ________ weeks
☐ For ________ months
☐ For ________ years
☐ Don't know
☐ Prefer not to answer

ME09h. What is the medication used for?

____

ME09i. ***Interviewer comments*:**

____

ME09j. Do you take any other prescription medications or over-the-counter drugs?

☐ No → ***skip to end of medications questions***
☐ Yes
☐ Don't know
☐ Prefer not to answer

ME10a. What is the name of the next prescription medication or over-the-counter drug that you take?

☐ Label product name: ____

☐ Label generic name: ____

☐ Don't know ☐ Prefer not to answer

ME10b. What is the dosage form?

Oral
- ☐ Pill, tablet, or capsule
- ☐ Sublingual or orally-disintegrating tablet
- ☐ Liquid solution or suspension (drink, syrup)
- ☐ Powder

Topical
- ☐ Liquid, cream, gel, or ointment
- ☐ Ear drops (otic)
- ☐ Eye drops (ophthalmic)
- ☐ Skin patch (transdermal)

Inhaled
- ☐ Inhaler or nebulizer

Injected
- ☐ Injection

Suppository
- ☐ Rectal (e.g., enema)
- ☐ Vaginal (e.g., douche, pessary)

Other:
- ☐ Don't know
- ☐ Prefer not to answer

ME10c. How frequently do you take it?
- ☐ __________ times per day
- ☐ __________ times per week
- ☐ __________ times per month
- ☐ Don't know
- ☐ Prefer not to answer

ME10d. What is the strength? ***(Record strength of how it is actually taken, not how it is prescribed.)***
- ☐ __________ %
- ☐ __________ mg
- ☐ __________ mcg
- ☐ __________ grams
- ☐ __________ I.U.
- ☐ __________ Other unit: __________
- ☐ Don't know
- ☐ Prefer not to answer

ME10e. Total dosage **per day.** ***(Record strength of how it is actually taken, not how it is prescribed.)***
- ☐ __________ %
- ☐ __________ mg
- ☐ __________ mcg
- ☐ __________ grams
- ☐ __________ I.U.
- ☐ __________ Other unit: __________
- ☐ Don't know
- ☐ Prefer not to answer

ME10f. Do you take it regularly or only as needed?
- ☐ Regularly
- ☐ Only as needed
- ☐ Don't know
- ☐ Prefer not to answer

ME10g. For how long have you been taking it?

- ☐ For ____________ days
- ☐ For ____________ weeks
- ☐ For ____________ months
- ☐ For ____________ years
- ☐ Don't know
- ☐ Prefer not to answer

ME10h. What is the medication used for?

____

ME10i. ***Interviewer comments***:
____

FOOD FREQUENCY QUESTIONNAIRE:

RA Script to be read to participants: At this time, we are going to have you complete a few forms on your own. The first is a Food Frequency Questionnaire that will ask you about the types of foods and drinks that you consume. It should take around 20 minutes to complete. Please let me know if you have any questions.

Comment [SF2]: At this time, participants will be directed to complete the three page Harvard University Food Frequency Questionnaire (see proof included), known as the "80-out". It is designed to take around 20 minutes to complete and is self-administered with paper and pencil. Once completed, they will proceed to the next section of this assessment (DS_1).

DA80 Printed in U.S.A.

Dietary Assessment

ID: ☐☐☐☐☐☐☐ — ☐☐

0 1 2 3 4 5 6 7 8 9 (grid of ID bubbles)

1. Do you currently take multi-vitamins? (Please report **other individual** vitamins in question 2.)

○ No ○ Yes → **If YES, a)** How many do you take per week?

○ 2 or less ○ 3-5 ○ 6-9 ○ 10 or more

b) For how many years have you taken them?

○ 0-1 ○ 2-4 ○ 5-9 ○ 10 or more

c) What specific brand do you usually use? → Specify exact brand and type

2. Not counting multi-vitamins, do you take any of the following preparations:

a) Vitamin A? ○ No ○ Yes, seasonal only ○ Yes, most months → **If YES,**

How many years?	○ 0-1 yr.	○ 2-4 years	○ 5-9 years	○ 10+ years	○ Don't know
What dose per day?	○ Less than 8,000 IU	○ 8,000 to 12,000 IU	○ 13,000 to 22,000 IU	○ 23,000 IU or more	○ Don't know

b) Vitamin C? ○ No ○ Yes, seasonal only ○ Yes, most months → **If YES,**

How many years?	○ 0-1 yr.	○ 2-4 years	○ 5-9 years	○ 10+ years	○ Don't know
What dose per day?	○ Less than 400 mg.	○ 400 to 700 mg.	○ 750 to 1250 mg.	○ 1300 mg. or more	○ Don't know

c) Vitamin B_6? ○ No ○ Yes → **If YES,**

How many years?	○ 0-1 yr.	○ 2-4 years	○ 5-9 years	○ 10+ years	○ Don't know
What dose per day?	○ Less than 10 mg.	○ 10 to 39 mg.	○ 40 to 79 mg.	○ 80 mg. or more	○ Don't know

d) Vitamin E? ○ No ○ Yes → **If YES,**

How many years?	○ 0-1 yr.	○ 2-4 years	○ 5-9 years	○ 10+ years	○ Don't know
What dose per day?	○ Less than 100 IU	○ 100 to 250 IU	○ 300 to 500 IU	○ 600 IU or more	○ Don't know

e) Selenium ○ No ○ Yes → **If YES,**

How many years?	○ 0-1 yr.	○ 2-4 years	○ 5-9 years	○ 10+ years	○ Don't know
What dose per day?	○ Less than 80 mcg.	○ 80 to 130 mcg.	○ 140 to 250 mcg.	○ 260 mcg. or more	○ Don't know

f) Iron? ○ No ○ Yes → **If YES,**

How many years?	○ 0-1 yr.	○ 2-4 years	○ 5-9 years	○ 10+ years	○ Don't know
What dose per day?	○ Less than 51 mg.	○ 51 to 200 mg.	○ 201 to 400 mg.	○ 401 mg. or more	○ Don't know

g) Zinc? ○ No ○ Yes → **If YES,**

How many years?	○ 0-1 yr.	○ 2-4 years	○ 5-9 years	○ 10+ years	○ Don't know
What dose per day?	○ Less than 25 mg.	○ 25 to 74 mg.	○ 75 to 100 mg.	○ 101 mg. or more	○ Don't know

h) Calcium? (Include Calcium in Dolomite and Tums, etc.) ○ No ○ Yes → **If YES,**

How many years?	○ 0-1 yr.	○ 2-4 years	○ 5-9 years	○ 10+ years	○ Don't know
What dose per day?	○ Less than 400 mg.	○ 400 to 900 mg.	○ 901 to 1300 mg.	○ 1301 mg. or more	○ Don't know

i) Are there other supplements that you take on a regular basis? Please mark if yes:

○ Metamucil ○ Cod liver oil ○ Iodine ○ Beta-carotene ○ Other (please specify)
○ Vitamin D ○ Folic acid ○ Copper ○ Niacin
○ B-Complex Vitamins ○ Omega-3 fatty acids ○ Brewer's yeast ○ Magnesium

3. For each food listed, fill in the circle indicating how often on average you have used the amount specified **during the past year.**

DAIRY FOODS	NEVER OR LESS THAN ONCE PER MONTH	1-3 PER MONTH	1 PER WEEK	2-4 PER WEEK	5-6 PER WEEK	1 PER DAY	2-3 PER DAY	4-5 PER DAY	6+ PER DAY
Skim or low fat milk (8 oz. glass)	○	○	Ⓦ	○	○	Ⓓ	○	○	○
Whole milk (8 oz. glass)	○	○	Ⓦ	○	○	Ⓓ	○	○	○
Yogurt (1 cup)	○	○	Ⓦ	○	○	Ⓓ	○	○	○
Ice Cream (1/2 cup)	○	○	Ⓦ	○	○	Ⓓ	○	○	○
Cottage or ricotta cheese (1/2 cup)	○	○	Ⓦ	○	○	Ⓓ	○	○	○
Other cheese, e.g. American, cheddar, etc., plain or as part of a dish (1 slice or 1 oz. serving)	○	○	Ⓦ	○	○	Ⓓ	○	○	○
Margarine (pat), added to food or bread; exclude use in cooking	○	○	Ⓦ	○	○	Ⓓ	○	○	○
Butter (pat), added to food or bread; exclude use in cooking	○	○	Ⓦ	○	○	Ⓓ	○	○	○

(AVERAGE USE LAST YEAR)

4. What form of margarine do you usually use? → What specific brand and type (e.g., Parkay Corn Oil Spread)

○ None ○ Stick ○ Tub ○ Extra light
○ 'Lite' stick ○ 'Lite' tub ○ Squeeze

PLEASE TURN TO PAGE 2

Mark Reflex® by NCS MP42968:14131211109 A2402

3. (Continued) Please fill in your <u>average</u> use, <u>during the past year</u>, of each specified food.

Please try to average your seasonal use of foods over the entire year. For example, if a food such as peaches are eaten 4 times a week during the approximate 3 months that it is in season, then the <u>average</u> use would be once per week.

FRUITS	NEVER OR LESS THAN ONCE PER MONTH	1-3 PER MONTH	1 PER WEEK	2-4 PER WEEK	5-6 PER WEEK	1 PER DAY	2-3 PER DAY	4-5 PER DAY	6+ PER DAY
Fresh apples or pears (1)									
Oranges (1)									
Orange juice or grapefruit juice (small glass)									
Peaches, apricots or plums (1 fresh, or 1/2 cup canned)									
Bananas (1)									
Other fruits, fresh, frozen, or canned (1/2 cup)									

VEGETABLES	NEVER OR LESS THAN ONCE PER MONTH	1-3 PER MONTH	1 PER WEEK	2-4 PER WEEK	5-6 PER WEEK	1 PER DAY	2-3 PER DAY	4-5 PER DAY	6+ PER DAY
Tomatoes (1) or Tomato juice (small glass)									
String beans (1/2 cup)									
Broccoli (1/2 cup)									
Cabbage, cauliflower, or Brussels sprouts (1/2 cup)									
Carrots, raw (1/2 carrot or 2-4 sticks)									
Carrots, cooked (1/2 cup)									
Corn (1 ear or 1/2 cup frozen or canned)									
Peas or lima beans (1/2 cup fresh, frozen, canned)									
Yams or sweet potatoes (1/2 cup)									
Spinach or collard greens, cooked (1/2 cup)									
Beans or lentils, baked or dried (1/2 cup)									
Yellow (winter) squash (1/2 cup)									

MEAT, SWEETS, BAKED GOODS, CEREAL, MISC.	NEVER OR LESS THAN ONCE PER MONTH	1-3 PER MONTH	1 PER WEEK	2-4 PER WEEK	5-6 PER WEEK	1 PER DAY	2-3 PER DAY	4-5 PER DAY	6+ PER DAY
Eggs (1)									
Chicken or turkey, with skin (4-6 oz.)									
Chicken or turkey, without skin (4-6 oz.)									
Bacon (2 slices)									
Hot dogs (1)									
Processed meats, e.g. sausage, salami, bologna, etc. (piece or slice)									
Liver (3-4 oz.)									
Hamburger (1 patty)									
Beef, pork, or lamb as a sandwich or mixed dish, e.g. stew, casserole, lasagna, etc.									
Beef, pork, or lamb as a main dish, e.g., steak, roast, ham, etc. (4-6 oz.)									
Fish (3-5 oz.)									
Chocolate (1 oz.)									
Candy without chocolate (1 oz.)									
Pie, homemade (slice)									
Pie, ready made (slice)									
Cake (slice)									
Cookies (1)									
Cold breakfast cereal (1 cup)									
White bread (slice), including pita bread									
Dark bread (slice), including wheat pita bread									
French fried potatoes (4 oz.)									
Potatoes, baked, boiled (1) or mashed (1 cup)									
Rice or Pasta, e.g., spaghetti, noodles, etc. (1 cup)									
Potato chips or corn chips (small bag or 1 oz.)									
Nuts (small packet or 1 oz.)									
Peanut butter (1 Tbs)									
Oil and vinegar dressing, e.g., Italian, (1 Tbs)									

PLEASE GO TO PAGE 3

4. (Continued) Please fill in your **average** use, **during the past year**, of each specified food.

BEVERAGES	NEVER OR LESS THAN ONCE PER MONTH	1-3 PER MONTH	1 PER WEEK	2-4 PER WEEK	5-6 PER WEEK	1 PER DAY	2-3 PER DAY	4-5 PER DAY	6+ PER DAY
Coffee, not decaffeinated (1 cup)	◯	◯	Ⓦ	◯	◯	Ⓓ	◯	◯	◯
Tea (1 cup), not herbal tea	◯	◯	Ⓦ	◯	◯	Ⓓ	◯	◯	◯
Beer (1 glass, bottle, can)	◯	◯	Ⓦ	◯	◯	Ⓓ	◯	◯	◯
Wine (4 oz. glass)	◯	◯	Ⓦ	◯	◯	Ⓓ	◯	◯	◯
Liquor, e.g. whiskey, gin, etc. (1 drink or shot)	◯	◯	Ⓦ	◯	◯	Ⓓ	◯	◯	◯
Low calorie carbonated beverage, e.g., Diet Coke	◯	◯	Ⓦ	◯	◯	Ⓓ	◯	◯	◯
Carbonated beverage with sugar, e.g., Coke, Pepsi	◯	◯	Ⓦ	◯	◯	Ⓓ	◯	◯	◯
Hawaiian Punch, lemonade, or other fruit drinks	◯	◯	Ⓦ	◯	◯	Ⓓ	◯	◯	◯

Consider the serving size as 1 glass, bottle or can for these fruit and carbonated beverages.

5. **How many teaspoons of sugar do you add to your beverages or food each day?** → tsp. ⓪①②③④⑤⑥⑦⑧⑨

6. **Which cold breakfast cereal do you usually eat?** → Specify brand and type

◯ Don't eat cold breakfast cereal

7. **How much of the visible fat on your beef, pork or lamb do you remove before eating?**

◯ Remove all visible fat ◯ Remove most ◯ Remove small part of fat ◯ Remove none ◯ Don't eat meat

8. **What kind of fat do you usually use for frying and sautéing at home? (Exclude "Pam"-type spray)**

◯ Real butter ◯ Margarine ◯ Vegetable oil ◯ Vegetable shortening ◯ Lard

9. **What kind of fat do you usually use for baking at home?**

◯ Real butter ◯ Margarine ◯ Vegetable oil ◯ Vegetable shortening ◯ Lard

10. **How often do you eat food that is fried at home? (Exclude "Pam"-type spray)**

◯ Less than once a week ◯ 1-3 times per week ◯ 4-6 times per week ◯ Daily

11. **How often do you eat fried food away from home? (e.g. french fries, fried chicken, fried fish)**

◯ Less than once a week ◯ 1-3 times per week ◯ 4-6 times per week ◯ Daily

12. **What type of cooking oil do you usually use at home (e.g. Mazola Corn Oil)?** → Specify brand and type

13. **Do you use a microwave oven?**

◯ Yes → **If yes, for how many years?** → Number of years using a microwave

◯ No

14. **Do you currently follow a physician-prescribed special diet?**

◯ Yes → **a) If yes, for how many years?** → Number of years on diet

◯ No **b) If yes, what kind of diet do you follow? (Select more than one if necessary.)**

◯ Weight reduction (low calorie) ◯ Low cholesterol ◯ Low sodium
◯ Diabetic ◯ Low fat ◯ Low triglyceride ◯ Ulcer ◯ High Potassium
◯ Other → Specify type of diet

15. How has your use of the following foods and beverages changed over the **PAST FIVE YEARS?**

FOOD	USE HAS DECREASED	USE ABOUT THE SAME	USE HAS INCREASED
a) Whole milk	◯	◯	◯
b) Butter	◯	◯	◯
c) Margarine	◯	◯	◯
d) Eggs	◯	◯	◯
e) Fish	◯	◯	◯
f) Red meat	◯	◯	◯
g) Fruits	◯	◯	◯
h) Vegetables	◯	◯	◯
i) Whole wheat bread	◯	◯	◯
j) Whole grains	◯	◯	◯
k) Sugar	◯	◯	◯
l) Alcohol	◯	◯	◯

Copyright © 1991. Brigham and Women's Hospital

Questions for Participants to Answer on Their Own In-Person on Paper:
For each statement, please place a mark in the column that best describes how you have been feeling.

	Not at all or less than 1 day last week	1 or 2 days last week	3 to 4 days last week	5 to 7 days last week	Nearly every day for two weeks	I do not know	I prefer not to answer
DS_1. My appetite was poor.	☐	☐	☐	☐	☐	☐	☐
DS_2. I could not shake off the blues.	☐	☐	☐	☐	☐	☐	☐
DS_3. I had trouble keeping my mind on what I was doing.	☐	☐	☐	☐	☐	☐	☐
DS_4. I felt depressed.	☐	☐	☐	☐	☐	☐	☐
DS_5. My sleep was restless.	☐	☐	☐	☐	☐	☐	☐
DS_6. I felt sad.	☐	☐	☐	☐	☐	☐	☐
DS_7. I could not get going.	☐	☐	☐	☐	☐	☐	☐
DS_8. Nothing made me happy.	☐	☐	☐	☐	☐	☐	☐
C_9. I felt like a bad person.	☐	☐	☐	☐	☐	☐	☐
DS_10. I lost interest in my usual activities.	☐	☐	☐	☐	☐	☐	☐
DS_11. I slept much more than usual.	☐	☐	☐	☐	☐	☐	☐
DS_12. I felt like I was moving too slowly.	☐	☐	☐	☐	☐	☐	☐
DS_13. I felt fidgety.	☐	☐	☐	☐	☐	☐	☐
DS_14. I wished I were dead.	☐	☐	☐	☐	☐	☐	☐
DS_15. I wanted to hurt myself.	☐	☐	☐	☐	☐	☐	☐
DS_16. I was tired all the time.	☐	☐	☐	☐	☐	☐	☐
DS_17. I did not like myself.	☐	☐	☐	☐	☐	☐	☐
DS_18. I lost a lot of weight without trying to.	☐	☐	☐	☐	☐	☐	☐
DS_19. I had a lot of trouble getting to sleep.	☐	☐	☐	☐	☐	☐	☐
DS_20. I could not focus on the important things.	☐	☐	☐	☐	☐	☐	☐

Below is a list of common symptoms of anxiety. Please carefully read each item in the list. Indicate how much you have been bothered by that symptom during the past month, including today, by marking the box in the corresponding space in the column next to each symptom.

	Not At All	Mildly – it didn't bother me much	Moderately – it wasn't pleasant at all times	Severely – it bothered me a lot	I do not know	I prefer not to answer
BE_1. Numbness or tingling	☐	☐	☐	☐	☐	☐
BE_2. Feeling hot	☐	☐	☐	☐	☐	☐
BE _3. Wobbliness in legs	☐	☐	☐	☐	☐	☐
BE _4. Unable to relax	☐	☐	☐	☐	☐	☐
BE _5. Fear of worst happening	☐	☐	☐	☐	☐	☐
BE _6. Dizzy or lightheaded	☐	☐	☐	☐	☐	☐
BE _7. Heart pounding/racing	☐	☐	☐	☐	☐	☐
BE _8. Unsteady	☐	☐	☐	☐	☐	☐
BE _9. Terrified or afraid	☐	☐	☐	☐	☐	☐
BE _10. Nervous	☐	☐	☐	☐	☐	☐
BE _11. Feeling of choking	☐	☐	☐	☐	☐	☐
BE _12. Hands trembling	☐	☐	☐	☐	☐	☐
BE _13. Shaky/unsteady	☐	☐	☐	☐	☐	☐
BE _14. Fear of losing control	☐	☐	☐	☐	☐	☐
BE _15. Difficulty in breathing	☐	☐	☐	☐	☐	☐
BE _16. Fear of dying	☐	☐	☐	☐	☐	☐
BE _17. Scared	☐	☐	☐	☐	☐	☐
BE _18. Indigestion	☐	☐	☐	☐	☐	☐
BE _19. Faint/lightheaded	☐	☐	☐	☐	☐	☐
BE _20. Face flushed	☐	☐	☐	☐	☐	☐
BE _21. Hot/cold sweats	☐	☐	☐	☐	☐	☐

After questionnaires are complete, participants will be asked to perform the following two computer-based tests:

Attention Control: Attention Network Test (20 minutes)

Attention Network Test (ANT) is a brief computerized battery measuring three independent behavioral components of attention: Conflict resolution (ability to overcome distracting stimuli), spatial Orienting (the benefit of valid spatial pre-cues), and Alerting (the benefit of temporal pre-cues). Efficiency of orienting is examined by changes in RT that accompany cues indicating where the target will occur. The efficiency of the executive conflict resolution network is examined by requiring the subject to respond by pressing two keys indicating the direction (left or right) of a central arrow surrounded by congruent, incongruent or neutral flankers. Moderate to high reliabilities are found for all networks.

Sustained Attention to Response Task (15 minutes)

The Sustained Attention to Response Task (SART) is a computerized test of sustained attention, response inhibition (executive function) and self-regulation. Subjects are instructed to press a key in response to rapidly displayed integers (1-9) and withhold response to a designated "no-go" integer. SART errors consist of summed commission errors (button press on no-go trial) and omission errors (button not pressed on "go" integers). SART performance is associated with prefrontal cortex functioning, has been found to increase with mindfulness training and is correlated with scores on mindfulness questionnaires (specifically, the Mindful Attention Awareness Scale).

SAFETY PLAN

After the participant has completed the Beck Anxiety Inventory questionnaire and the Centers for Epidemiologic Studies Depressive Symptomatology questionnaire, ask the participant to wait for a few minutes while you check that all forms and assessments are completed. While the participant is waiting in a different room, check the scores according to the criteria below. If any scores trigger the safety plan, move forward with steps below as written.

Beck Anxiety Inventory (BA)

If participant scores ≥26 on the Beck Anxiety Inventory, while having the participant wait, immediately notify the collaborating psychiatrist, Dr. Ellen Flynn (cell phone # 401-258-9829), to determine need for a psychiatric consultation. She has provided her cell phone #, and your phone # will be entered into her phone recognition software to indicate it is a call from the MBHT study. It is likely she will answer the phone. If Dr. Flynn cannot be reached, please leave a phone message for her with your contact information and provide her with the Beck Anxiety Inventory results on the message. If Dr. Flynn is not immediately reachable, or following participant release after consulting with Dr. Flynn, immediately contact the PI, Dr. Eric Loucks (cell phone #401-369-0443).

Depressive Symptomatology (DS)

The CESD-R will be administered during the in-person assessment visits, and scores will be reviewed immediately upon completion of the in-person assessments.

1. Sadness (dysphoria): Question numbers 2,4, 6
2. Loss of Interest (anhedonia): Question numbers 8, 10
3. Appetite: Question numbers 1, 18
4. Sleep: Question numbers 5, 11, 19
5. Thinking / concentration: Question numbers 3, 20
6. Guilt (worthlessness): Question numbers 9, 17
7. Tired (fatigue): Question numbers 7, 16
8. Movement (agitation): Question numbers 12, 13
9. Suicidal ideation: Question numbers 14, 15

Participants are considered to meet criteria for major depressive episode if they have anhedonia or dysphoria nearly every day for the past two weeks, plus symptoms in an additional 4 DSM symptom groups noted as occurring nearly every day for the past two weeks. If participants meet criteria for major depressive episode, while having the participant wait, immediately notify the collaborating psychiatrist, Dr. Ellen Flynn (cell phone # 401-258-9829), to determine need for a psychiatric consultation. She has provided her cell phone #, and your phone # will be entered into her phone recognition software to indicate it is a call from the MBHT study. It is likely she will answer the phone. If Dr. Flynn cannot be reached, please leave a phone message for her with your contact information and provide her with the Depressive Symptomatology results on the message. If Dr. Flynn is not immediately reachable, or following participant release after consulting with Dr. Flynn, immediately contact the PI, Dr. Eric Loucks (cell phone #401-369-0443).

If participants respond having any suicidal ideation (DS questions 14 or 15), perform the following 2 steps:

1. **Immediately call 911 and Dr. Ellen Flynn.**

Specifically, while the participant is in the waiting room, ***call 911 immediately, and tell them:***

"My name is _____. I am working on a research study at Brown University. I have a study participant in the waiting room who has shared that he/she is currently suicidal." Please provide the participants' name to the 911 operator, as requested.

Finally, after calling 911, please call Dr. Ellen Flynn, who is the psychiatrist supporting this study. Her cell phone # is 401-258-9829. Please provide her with the same information as was done during the 911 call.

2. **While speaking calmly with the participant, let them know that you have called 911 and Dr. Flynn, and why (i.e. because we are concerned about you). You can speak with participant to keep him/her in the waiting room if 911 has sent assistance, but the discussion should not be clinical in nature.**

Examples of questions that could be asked in order to keep them in the waiting room:
- "Tell me what is going on."
- "What's happening right now?"
- Tell me more about why you are interesting in being part of this study.
- What are you hoping to get out of this study?

The following information can be provided to study participants.

National Suicide Prevention Lifeline: 1-800-273-8255

Other options are to:
- Call your doctor's office
- Call 911 for emergency services
- Go to the nearest hospital emergency room.

Local Non-Urgent Free or Inexpensive Mental Health Services:
Gateway Healthcare: 401-729-8701
Anchor Counseling Center: 401-475-9979
The Providence Center: 401-276-4020

E. Loucks responses to 12/4/15 email from Ita Irizarry – RE: "MINDFULNESS-BASED HYPERTENSION THERAPY PILOT STUDY," Amendment #4 submission.

Questions/Requests:

1. Regarding the request to add a control group, please confirm if this population will be identified, recruited, and consented with the same currently-approved procedures as the intervention group.

   Based on additional feedback from NCCIH on the study protocol, we have decided to hold off at this time on implementing the RCT. We have removed all language discussing the RCT and have attached an updated consent form (v.1.5) and Phone Screener. We will submit a new amendment to the IRB once the RCT is incorporated into the study protocol.

2. Will the new $100 compensation be provided to all participants who complete the study, both intervention and control groups? Will the compensation be given at the 1-year follow-up?

   Yes, all study participants will receive the $100. It will be given out at the time of the 1-year follow-up. We will not be including a control group at this time.

3. What is the rationale for adding the 1-year follow-up visit?

   There is early evidence in other mindfulness studies that have shown increases in effect by 1 year follow-up that were not present at 6 months. This longer follow-up time will allow us to determine longer term effectiveness of the intervention.

4. What is the rationale for revising the screening and baseline assessments?

   The screening assessments were revised to reduce participant burden. We removed all assessments in the first in-person screening that do not influence if the person qualifies for the study or not. For the in-person baseline assessment, we included items moved from the first in-person screening, as well as added some additional measures that are central to the objectives of the recent NIH grant to evaluation impacts of the intervention on measures of self regulation.

5. For the *In-Person Screening Assessment Form*, question BA2_08 (pg. 2) is marked as track-changed, but this was approved with Amendment #2. Was the question revised?

   No, it was not revised. It was marked in error.

6. If you haven't already, please register your study on the Clinical Trials website, per the terms of your NIH grant. Section 6 (pg. 254) states that the study must be registered within 21 days of the first participant enrollment.

   We will register the study on the Clinical Trials website within 21 days of the first participant enrollment for the NIH-funded study.

Consent Document Revisions:

1. The *Nature and Purpose of the Study* section (pg. 1, 3rd paragraph) only briefly mentions that participants may be randomized into the control group. To the *Explanation of Procedures* section, please add how that randomized will occur, and provide a further explanation of what participants in the control group will be asked to do at the baseline and 10-week visits.

   No longer applicable. We have decided to not implement the RCT at this time.

2. Portions of the consent are marked as track-changed, although they were approved with Amendment #3. For example, the *Explanation of Procedures* section (pgs. 2-3, #3) and the procedure table (pg. 3, focus group) are marked. Were changes made to these sections?

   No, these two sections were not revised. They were marked in error. We have removed the track changes.

3. To the *Discomforts and Risks* section (pg. 4, 3rd paragraph), please add an explanation of what will happen if a participant has a research-related injury.

   We have added the following language to the consent form (see v.1.5 attached):

   "While physical and mental injury is always a possibility the potential for harm is limited. Note that a research injury is any physical or mental injury or illness caused by your participation in the study. If you are injured by a medical treatment or procedure that you would have received even if you were not in the study, that is not a research injury. To help avoid research injury and potential added medical expenses, it is important to follow all study directions carefully. If you are covered by insurance and suffer a research injury, it is possible that some or all of the costs of treating your condition could appropriately be billed to your insurance company. If such costs are not covered your health insurance company, it is possible you would have to pay for these costs out of pocket. Brown University's policies do not cover payment for such things as lost wages, medical care expenses, or pain and suffering.

   Precautions should be taken to avoid injuries. If you do become injured during the study, you should call your doctor immediately. You should also alert the study staff that you have been injured. Heart attack and sudden death related to heart problems have been known to occur in people while they are exercising. This is very rare, however. Estimates of sudden cardiac death range from 0 to 2 per 100,000 hours. However, the researchers cannot guarantee that no complications will happen to you."

4. In the *Benefits* section (pg. 4), please remove the added compensation description. Compensation is not considered a benefit, and this information was already added to the *Nature and Purpose of the Study* section (pg. 1).

   This has been removed.

5. As this is clinical trial, please add the required language regarding the Clinical Trials website: "A description of this clinical trial will be available on http://www.Clinical Trials.gov, as required by U.S. Law. This Web site will not include information that can identify you. At most, the Web site will include a summary of the results. You can search this Web site at any time." This language must be added verbatim and cannot be changed, per NIH regulations.

   We have added this language to the last section of the consent.

See below for a summary of changes made to the MBHT study documents since Amendment #4 was originally submitted. These changes were made per discussion with the Brown University IRB; some additional changes have been made in order to reduce participant burden. All modified documents have been included in this email.

INFORMED CONSENT FORM

- Removed language regarding the RCT
- Removed compensation description from the "Benefit" section
- Added language regarding research-related injuries
- Updated the IRB contact information
- Added the required language regarding clinical trials

PHONE SCREENER

- Removed language regarding the RCT

IN-PERSON SCREENER

- Removed the 4th and 5th Blood Pressure readings, such that BP will be taken three times in-person rather than five times.

IN-PERSON BASELINE

- Removed the 4th and 5th Blood Pressure readings, such that BP will be taken three times in-person rather than five times.
- Added the data entry variables for the Attention Network Test (see p.23). This data will be entered by the RA at the end of the test.

HOME BASELINE

- Removed all sleep questions *except for* SL1_04
- Removed the sense of control scale (SE1)
- Removed the Mindfulness Skill Acquisition Scale (MS1)

Agreement to Participate in a Research Study

Investigation of the Effects of Mindfulness on Blood Pressure and Well-Being

You are being asked to take part in a Brown University research study about the effects of mindfulness and hypertension education on blood pressure and risk factors for hypertension. This form will explain the purpose of the study, how the study will be carried out and what you will be expected to do. It will also explain the possible risks and possible benefits of being in the study. If any part of the following description is not clear to you, you are encouraged to contact the researcher to answer any questions before you decide whether to take part in the study. If you decide to participate, please fill out and sign the last page of this form.

1a. Nature and Purpose of the Study

The purpose of the study is to investigate the impact of mindfulness practices and health education on blood pressure. You have been selected for this study because you expressed interest in the project and because you met entrance criteria for having prehypertension or hypertension, or another cardiovascular risk factor that could be influenced by this program.

In order to assess the effects of these practices, you will be asked to complete some questionnaires and ~~a~~ laboratory assessments before and after learning the mindfulness practices. Assessments will be completed at: baseline, 10 weeks, 6 months and 1 year. If you complete the study, you will be given $100 USD at the time of completion for your participation, to express our gratitude.

Participation in this study involves receiving training in mindfulness practices as well as health education on blood pressure.

1b. Explanation of Procedures

If you agree to participate, you will be asked to consent to the following:

1) Participate in an interview in which you will be asked questions about past and present mental health, including depression and suicide.

2) Complete questionnaires about a wide range of topics, including your diet, physical activity, smoking, medication use, personality, emotions, attention and past experiences, including stressful or traumatic experiences. These questions will probe sensitive psychological areas, including physical, emotion and sexual abuse. These questionnaires may take up to ~~2~~ 3 hours to complete. By completing the interviews and questionnaires, you are giving the researchers permission to use the information you have provided. You have the right not to answer any of the questions.

3) Directly assessed blood pressure, height, weight, waist circumference, hip circumference, physical activity, and antihypertensive (blood pressure) medication use at baseline and after the mindfulness course. Physical activity will be assessed for a week at a time using small actigraphy monitors that attach to your wrist and hip. If you take antihypertensive

medication, we will provide you with an electronic bottle cap that will automatically record when the pill bottle is opened during the study. This will help us measure how often the medication is used.

4) You will be asked to perform some cognitive tasks. One Some of these tasks may involve computer-based tests of attention or decision-making. Together these tests may take as long as 45 minutes.

5) You will participate in the mindfulness program, which consists of 9 weekly sessions of 2.5 hours each and will include one 8 hour weekend retreat. Daily homework assignments may take as long as one hour and consist of practicing mindfulness exercises with the aid of a guided audio CD and completing worksheets related to stress, thoughts, and common reactions to various types of events.

6) Class sessions will be audio taped so we can analyze the quality of the treatment you receive. The recordings will be transcribed so that we may analyze the text. The recordings will be identified by study number, will only be heard by study staff and will be destroyed after transcription.

7) You will be asked to complete a few short questionnaires each week during the 9 week condition.

8) After 9 10 weeks, you will be asked to complete questionnaires and return to the laboratory to repeat the same procedures for a second day of testing. You will also be invited to participate in a focus group to share any advice you may have on how to improve the intervention.

9) Three Six months and one year after the end of the 9 week conditionthe beginning of the study, you will be asked to return to the laboratory to repeat the same testing procedures.

Table Summarizing Activities and Time Commitment for this Study.

Activity	Time Commitment
First blood pressure screen	30 minutes
Second blood pressure screen	30 minutes
Baseline health assessments, such as questionnaires, height, weight and waist circumference.	~~2 hours~~3 hours
Mindfulness course<br>*In-person*	9 sessions that are 2.5 hours each.<br>1 retreat day on a Saturday that will be 8 hours *Total course time: 30.5 hours*
Home ~~mindfulness~~ practices assigned during mindfulness course.	1 hour per day, 6 days per week, for 8 weeks. *Total practice time: 48 hours*
Health assessments, such as questionnaires, blood pressure, height, weight and waist circumference, that take place *immediately after course completion*.	~~2 hours~~3 hours
Health assessments, such as questionnaires, blood pressure, height, weight and waist circumference, that begin ~~*take place that take place 3*~~ *6 months after baseline*~~course completion~~.	~~2 hours~~3 hours
Health assessments, such as questionnaires, blood pressure, height, weight and waist circumference, that begin *1 year after baseline*.	3 hours
Participate in focus group after intervention completion to share any advice you may have on how to improve the intervention.	1.5 hours
**TOTAL TIME COMMITMENT FOR STUDY**	**~~87.0 hours~~93.0 hours**

Feedback:

At the end of the study, you will receive individual feedback about the changes that occurred since the first assessment. Specifically, you will receive an individualized handout listing % change (increase or decrease) on scales of attention, stress, mood, health behaviours, weight, and blood pressure across the study.

2. Discomforts and Risks

The risks to you in this study are small. The questionnaires used in the study are routine, standardized forms for epidemiologic research. Certain questions may be upsetting as they may probe sensitive psychological areas and inquire about upsetting or traumatic events, including physical, sexual or emotional abuse and/or current psychiatric symptoms. Since your participation is voluntary, you have the right to skip any questions that make you uncomfortable.

Meditation-based interventions may results in discomfort with attention to unpleasant thoughts, feelings or body sensations. Some individuals may experience an initial increase in undesirable feelings with increased attention to them.

It is possible that injuries could be sustained during the study either from the gentle mindful movements (i.e., yoga), or from physical activities that participants engage in as a way to reduce blood pressure. To help limit this, you will receive a handout showing the yoga poses that will be offered during the course that you can show your health care provider so that they can advise on which poses to do, and which to avoid. Modifications of poses will be available as needed. None of the poses (or the yoga as a whole) are mandatory to be done. You will also be encouraged to explore physical activities that promote strength and conditioning as a way to reduce blood pressure. You will be encouraged to not go beyond any physical limits of your body, and will be encouraged to ask your healthcare provider about advised physical activities and mindful movements if you have any physical limitations.

While physical and mental injury is always a possibility the potential for harm is limited. Note that a research injury is any physical or mental injury or illness caused by your participation in the study. If you are injured by a medical treatment or procedure that you would have received even if you were not in the study, that is not a research injury. To help avoid research injury and potential added medical expenses, it is important to follow all study directions carefully. If you are covered by insurance and suffer a research injury, it is possible that some or all of the costs of treating your condition could appropriately be billed to your insurance company. If such costs are not covered your health insurance company, it is possible you would have to pay for these costs out of pocket. Brown University's policies do not cover payment for such things as lost wages, medical care expenses, or pain and suffering.

Precautions should be taken to avoid injuries. If you do become injured during the study, you should call your doctor immediately. You should also alert the study staff that you have been injured. Heart attack and sudden death related to heart problems have been known to occur in people while they are exercising. This is very rare, however. Estimates of sudden cardiac death range from 0 to 2 per 100,000 hours. However, the researchers cannot guarantee that no complications will happen to you.

3. Benefits

We cannot and do not guarantee or promise that you will receive any direct benefits from this study. However, participation in the study creates the potential benefit of a) identifying effective treatments for elevated blood pressure, b) gaining knowledge of the effects of mindfulness practices, c) receiving information about your psychological and physical functioning.

4. Alternative Therapies

A number of different therapies, including antihypertensive medication, diet changes, physical activity, and reducing excessive alcohol consumption may also be beneficial for reducing blood pressure. Education about these therapies are integrated into this course, but other forms of these alternative therapies are also available in the community.

5. Confidentiality

Your responses for this study will be kept confidential. All data that we collect will be linked to a study ID# instead of your name. All questionnaires in this study will be filled out through an

online survey or a paper version of the survey if you prefer. All of these questionnaires will be linked solely to your study ID#, so that your identity is protected and your answers are confidential. Although these measures have been taken to protect your personal information, complete confidentiality cannot be guaranteed when transmitting information over the internet.

While your confidentiality is protected to the extent of the law, there are limitations to confidentiality. If your questionnaire responses indicate that you pose a serious danger to yourself or to another person, then a collaborator (Dr. Ellen Flynn) who is a licensed psychiatrist, may contact you to discuss your responses and possible referral to a treatment provider. Questionnaire items that may warrant follow-up include endorsements of statements about hurting yourself, any high scores in depression, anxiety, or other clinically significant problems. You should also know that there are times when the law might require the release of your responses without your permission. For example, State law requires researchers to report abuse or neglect of children to the Department of Children, Youth and Families (DCYF). State law also requires researchers to report abuse or neglect of people age 60 and older to the Division of Elderly Affairs.

The findings of the study may be used for medical publication. Your name will not be used in any published reports about this study. Results will be reported in a summarized manner in such a way that you cannot be identified. All personally identifiable information will be "de-identified" and only a unique code number will be used. Study records will be identified with a unique code number and initials. All study records and specimens will be stored in a secure storage area.

Keeping study records: The Principal Investigator for this study will keep your research records indefinitely for research purposes.

6. Refusal/Withdrawal

Participation is voluntary, and you may decide to opt out or refuse any part of participation, including not answering certain questions. If you decide now to participate, you can change your mind later and quit the study. The decision to not participate or to withdraw from the study will not adversely affect current or future interactions with Brown University. The decision to not participate or to withdraw from the study will also not adversely affect your relationship with your physician.

If you decide not to participate, or if you quit the study, we will provide you with referrals for alternative treatments, if desired.

7. Contact Information

If you have questions about study procedures at any time you may contact the researchers: Dr. Eric B. Loucks, email: eric.loucks@brown.edu, telephone (401) 863-6283. If you would like more information about the rules for research studies, or the rights of people who take part in those studies, you may contact the Brown University Human Research Protection ~~Office~~Program, telephone number 1-866-309-2095 or 401-863-3050.

A description of this clinical trial will be available on http://www.Clinical Trials.gov, as required by U.S. Law. This Web site will not include information that can identify you. At most, the Web site will include a summary of the results. You can search this Web site at any time.

CONSENT FORM:
Please sign and return.

Please read the following agreement and sign to consent to participating.

I HAVE READ THE ABOVE DESCRIPTION OF THIS STUDY. ALL OF MY QUESTIONS HAVE BEEN SATISFACTORILY ANSWERED, AND, I AGREE TO PARTICIPATE IN THIS RESEARCH STUDY.

____

PRINT NAME

____________________________________ __________________

Signature of participant **Date**

CONTACT INFORMATION

Name (print):____

Permanent Address:____

Email(s):____

Telephone:__________________________**(cell)** __________________________**(other)**

PHONE-DELIVERED SCREENING QUESTIONNAIRE

PHONE SCREENING QUESTIONNAIRE PART 1

The script is shown below in ***bold italics.***

PID. Participant ID: ___________

SQ01. Staff ID: ______________

SQ02. Today's Date (MMDDYY): ________________

SQ03. Current Time (24 hour time, e.g. 14:45): ________

Please now call the participant.

SQ04. Was the participant reached? YES NO

Hello, my name is ____________. I am calling from the Brown University School of Public Health because (name of participant) expressed interest in participating in our mindfulness blood pressure study. Is he/she available to talk at this time?

I would like to do a 10-15 minute phone interview with you to determine if you are a good match for this particular study.

SQ05. ***Is now a good time to speak?***

[If yes, proceed to SQ05 script below]

If no… ***When would be a good time to talk?***

SQ06. Day to call back (DDMMYY) ____________

SQ07. Time to call back ______ AM/PM

OK, great. How about I give a quick overview of the study, and can then answer any questions you may have to see if it is a good fit for you. We will then go through a screening questionnaire to see if you qualify for the study. Does this sound OK?

In this study, we are looking to see if mindfulness practices improve blood pressure, and if education about hypertension risk factors may also improve blood pressure. If eligible, we will provide you with training in meditation, mindful movements, and the roles of things like diet, physical activity and medication in reducing blood pressure. You will be taught by a very experienced teacher who is an expert in these fields. The course is free and will take place over a 9-week period, where you come to a class once each week for 2.5 hours each time. There is also a one-day retreat on a Saturday that will be 8 hours long. As part of the project, we will also ask you to participate in health assessments before and after the study. Health assessments include measures such as blood pressure, height and weight, and questionnaires about your health and experiences. At the end of the study, you will be given $100 USD to express our gratitude.

Deleted: *A basic overview of the study is that it is*

Deleted: *Specifically*

Deleted: *Y*

Deleted: *s*

Deleted: *Also, w*

Do you have any questions about the study? *[answer questions]*

SQ08. ***Does this study sound like something you would be interested in doing? Yes No***

[If yes, proceed to next statement below. If no, politely thank the participant for considering being in this study, and end the call].

Great. There are a few things that I would like to make clear before we start the interview. First of all, some of the questions that I will ask now to figure out if you are eligible to be in this study will be of personal nature, including asking about your mental health and life's experiences.

SQ09. ***Are you in a private place to talk?***

[If yes, proceed to text below. If no, reschedule meeting using variables SQ06 and SQ07 above].

Because this interview is of a personal nature, it is important that you understand that everything you say will be kept strictly confidential. No one outside of our project will ever be able to see your answers, and we will not keep your name in the same place as any of your answers. If you are not eligible after the phone screen, we will destroy your information. If you like, though, we can keep your information on file for future studies.

SQ10. Participant's First Name: ____

SQ11. Participant's Last Name:____

Participant's Address (in case we need to send any study materials to you):
SQ12a. Street address: ______________
SQ12b. City: ______________________
SQ12c: State: _________
SQ12d: Zip Code: _________________

SQ13a. Participant's Phone number #1 (in case we need to contact you by phone)
____

SQ13b. Participant's Phone number #2____

SQ14. Participant's email address (or mailing address if no email):________________________

SQ15. Notes from interviewer related to participants' contact information (if any):
____
____
____

Deleted: ***Can I answer any questions about the study that you may have***

Deleted: SQ04

Deleted: SQ05

PHONE SCREENING QUESTIONNAIRE PART 2

PID. Participant ID #: ______________

INCLUSION CRITERIA: All answers in 3rd column must be YES. If an answer is NO, immediately proceed to question SQ40.			
			Comments
SQ26.	***What is your age?*** [Is age at least 18 years?]	YES NO	
SQ27.	***Can you read and write in English?***	YES NO	

EXCLUSION CRITERIA: All answers in 3rd column must be NO with the exception of SQ32a and SQ33a. If an answer (other than SQ32a and SQ33a) is YES, then immediately proceed to question SQ40.			
			Comments
I will now start to ask some questions about your mental health.			
SQ28.	***Has anyone ever told you that you have bipolar disorder or manic depression?***	**YES NO**	
SQ29.	***Has anyone ever used the word "Borderline" to describe you?***	**YES NO**	
SQ30a.<br><br>SQ30b.	***Have you ever had a hallucination or seen things that other people can't see, or hear things other people can't hear?***<br><br>***Have you ever been diagnosed with schizophrenia or psychosis?***	**YES NO**<br><br>**YES NO**	
 <br>SQ31_1<br>SQ31_2<br>SQ31_3<br>SQ31_4<br>SQ31_5<br>SQ31_6<br>SQ31_7<br>SQ31_8	***Have you ever taken any of the following medications that I am about to read to you?***<br>***Lithium***<br>***Seroquel (quetiapine)***<br>***Abilify (aripiprazole)***<br>***Zyprexa (olanzapine)***<br>***Clozaril (clozapine)***<br>***Haldol/Haloperidol***<br>***Geodon (ziprasidone)***<br>***Risperdal (risperidone)***	 <br>**YES NO DK**<br>**YES NO DK**<br>**YES NO DK**<br>**YES NO DK**<br>**YES NO DK**<br>**YES NO DK**<br>**YES NO DK**<br>**YES NO DK**	
SQ32a<br><br>SQ32b<br><br>SQ32c<br><br>SQ32d	***Have you ever had a suicide attempt?***<br><br>***[If yes, ask…] Have you considered killing yourself during the past month?***<br><br>***[If yes, ask…] Are you currently suicidal?***<br><br>***[If yes, keep participant on the phone, and follow suicide safety plan below]***<br><br>***[If no, ask…] Are you getting any help for that? If not, then provide list of resources from Safety Plan including Gateway, Anchor and The Providence Center] Not urgent, but inform Dr. Flynn about what was discussed.***	**YES NO**<br><br>**YES NO**<br><br>**YES NO**<br><br>**YES NO**	

EXCLUSION CRITERIA: All answers in 3rd column must be NO with the exception of SQ32a and SQ33a. If an answer (other than SQ32a and SQ33a) is YES, then immediately proceed to question SQ40.			
SQ33a<br><br>SQ33b<br><br>SQ33c	***Would you say you have a trauma history?***<br>***[If yes…]***<br>***In the past month, have you had any problems with dissociation (memory loss)?***<br>***In the past month, have you had any flashbacks (i.e. sudden and disturbing vivid memory) about the trauma?***	**YES NO**<br><br>**YES NO**<br><br>**YES NO**	
SQ34a<br><br><br><br><br>SQ34b	***In the past month, have you had any problems with obsessions or compulsions, such as washing your hands or checking the oven over and over again?***<br>***[If yes…] Has anyone diagnosed you with obsessive compulsive disorder?***	**YES NO**<br><br><br><br><br>**YES NO**	
SQ35	***In the past month, have you had a panic attack (i.e. sweating, heart palpitations, nausea, trouble breathing, fear of dying/choking/going crazy)?***	**YES NO**	
SQ36	***Have you had any problems with alcoholism or drug use in the past year?***	**YES NO**	
SQ37	***In the past year, have you had an eating disorder, such as starving, binge eating, or vomiting?***	**YES NO**	
SQ38a<br><br><br>SQ38b<br><br><br><br><br><br>SQ38c	***Do you currently have a mindfulness practice, such as meditation or yoga?***<br><br>***Please tell me more about your mindfulness practice, including how often you practice per week.***<br><br><br><br><br>***Do you currently practice meditation more than once per week? (yoga does not count as meditation in this context)***	**YES NO**<br><br><br>**Fill in response in comments section to the right**<br><br>**YES NO**	
SQ39	***This class will take place at Brown University in-person. Do you have any medical or mobility issues that would affect you being able to attend class?***	**YES NO**	
SQ40	Participant qualifies for next step of study (next step is 1st blood pressure screening)	**YES NO**<br><br>**If YES go to SQ41.**<br><br>**If NO, go to SQ42.**	

SQ41a. ***Thank you for taking the time to answer these questions. You qualify for the next stage of screening, which is to take your blood pressure at our office. If you're still interested, we'd like to schedule a time to have you come in to complete the in-person screener. It will only take 30 minutes or less and will involve taking your blood pressure 3-5 times. Is there a day or time that works best for you?*** *[Schedule the In-Person Screening visit now]*

SQ41b. ***As I mentioned earlier, there is a mindfulness intervention that is part of this study. If you are eligible based on the In-Person screening, you will then be invited to take part in a 9 week Mindfulness Course. We will be scheduling the mindfulness intervention at a time that works best with most of the participants. What days and times of the week typically work best with your schedule? Please keep in mind that the sessions are 2.5 hours long, and take place once per week for 9 weeks.***

[Record participant's availability for Intervention and then proceed with the script]

Thank you for your time and interest in this study. We look forward to meeting you in person on [DATE / TIME]. Do you have any questions before we end this call?

IF INELIGIBLE:

SQ42a***. Thank you for taking the time to answer these questions. According to the survey, you do not qualify for the study at this time. There may be other studies you quality for.***

SQ42b. ***Would you like me to keep your information to pass on to these studies?*** YES / NO

SQ42c. [If yes…] ***OK, thank you. We will keep this information for future studies you may qualify for. Thank you for taking the time to talk with me today. Can I answer any questions before hanging up?***

SQ42d. [If no…] ***OK, our copy of this information will be destroyed. Thank you for taking the time to talk with me today. Can I answer any questions before hanging up?***

Deleted: ***at two different times at least a week apart from each other. Let's book the in-person screening sessions*** [Book both sessions. We can cancel the second session if they do not qualify].

Deleted:

SAFETY PLAN

Enter details below on paper during screening (These variables should are also be entered in the survey via questions SQ10-SQ13). Destroy this paper after screening is complete.

Participant's First Name: ___________________________________

Participant's Last Name:_____________________________________

Participant's Address:

Street address: _____________

City: ____________________

State: ________

Zip Code: ________________

Participant's Phone number #1: ___________________________________

Participant's Phone number #2___________________________________

During the phone-based screening, if participants respond yes to "Are you currently suicidal?", the interviewer should perform the following 2 steps:

1. **Immediately have 911 and Dr. Ellen Flynn called by a colleague who has been informed beforehand that this is a possibility.**

<u>Specifically, while keeping the participant on the phone, show the text below in the box to a colleague.</u>

I have a study participant on the phone who is currently suicidal. Please call 911 immediately, and tell them:<br><br>***"I am calling on behalf of [my name] who is performing a research study at Brown University. He has a participant on the phone who says they are currently suicidal." Please provide the participants' contact information to the 911 operator (i.e. name, address, phone #, email address) as requested. This information is shown above.***<br><br>***Finally, after calling 911, please call Dr. Ellen Flynn, who is the psychiatrist supporting this study. Her cell phone # is 401-258-9829. Please provide her with the same information as was done during the 911 call.***

2. **While speaking calmly with the participant, let them know what you are doing. Specifically, let them know we are calling our study's psychiatrist Dr. Flynn and 911, and why you are doing that (i.e. because we are concerned about you). You can speak with participant to keep him/her on the phone, but the discussion should not be clinical in nature.**

Examples of questions that could be asked in order to keep them on the phone:

- "Tell me what is going on."
- "What's happening right now?"
- Tell me more about why you are interesting in being part of this study.
- What are you hoping to get out of this study?

The following information can be provided to study participants if they state they have had considered killing themselves in the past month. If they are currently suicidal, the main priority is to keep them on the phone while 911 and Dr. Flynn are being contacted.

National Suicide Prevention Lifeline: 1-800-273-8255

Other options are to:

- Call your doctor's office
- Call 911 for emergency services
- Go to the nearest hospital emergency room.

Local Non-Urgent Free or Inexpensive Mental Health Services:
Gateway Healthcare: 401-729-8701
Anchor Counseling Center: 401-475-9979
The Providence Center: 401-276-4020

IN-PERSON SCREENING

ASSESSMENT FORM

Deleted: FIRST

Deleted: VISIT

PID. Participant ID # ___________
BA2_01. Staff ID # ___________
BA2_02. Today's date (MMDDYY): ___________

Blood Pressure:

BA2_03a. Blood pressure 1st reading, systolic blood pressure: _________ mmHg
BA2_03b. Blood pressure 1st reading, diastolic blood pressure: __________ mmHg
BA2_03c. Blood pressure 2nd reading, systolic blood pressure: _________ mmHg
BA2_03d. Blood pressure 2nd reading, diastolic blood pressure: __________ mmHg
BA2_03e. Blood pressure 3rd reading, systolic blood pressure: _________ mmHg
BA2_03f. Blood pressure 3rd reading, diastolic blood pressure: __________ mmHg

BA2_04. Were the 2nd and 3rd systolic blood pressure readings within 20 mmHg of each other?
- ◻ Yes
- ◻ No ***(repeat measurements***

BA2_05. Were the 2nd and 3rd diastolic blood pressure readings within 10 mmHg of each other?
- ◻ Yes
- ◻ No ***(repeat measurements)***

BA2_06a. Repeated blood pressure 1st reading, systolic blood pressure: _________ mmHg
BA2_06b. Repeated blood pressure 1st reading, diastolic blood pressure: __________ mmHg
BA2_06c. Repeated blood pressure 2nd reading, systolic blood pressure: _________ mmHg
BA2_06d. Repeated blood pressure 2nd reading, diastolic blood pressure: _________ mmHg
BA2_06e. Repeated blood pressure 3rd reading, systolic blood pressure: _________ mmHg
BA2_06f. Repeated blood pressure 3rd reading, diastolic blood pressure: __________ mmHg

BA2_07. Blood pressure cuff size used: ◻ S ◻ Reg ◻ L ◻ XL

BA2_08. Arm that cuff was placed on: ◻ L ◻ R

Deleted: BA2_03g. Blood pressure 4th reading, systolic blood pressure: _________ mmHg¶
BA2_03h. Blood pressure 4th reading, diastolic blood pressure: __________ mmHg¶
BA2_03i. Blood pressure 5th reading, systolic blood pressure: _________ mmHg¶
BA2_03j. Blood pressure 5th reading, diastolic blood pressure: __________ mmHg¶

Deleted: 4th and 5th

Deleted: 4th and 5th

Deleted: BA2_06g. Repeated blood pressure 4th reading, systolic blood pressure: _________ mmHg¶
BA2_06h. Repeated blood pressure 4th reading, diastolic blood pressure: __________ mmHg¶
BA2_06i. Repeated blood pressure 5th reading, systolic blood pressure: _________ mmHg¶
BA2_06j. Repeated blood pressure 5th reading, diastolic blood pressure: __________ mmHg¶

Comment [SF1]: In order to reduce participant burden, Medication Use has been moved to the In-Person baseline assessment since it is not part of the eligibility criteria.

Deleted: Medications (ME)

Deleted: ¶
¶
¶
ME01. Do you take any prescription medications or over-the-counter drugs?¶
<#>Section Break (Continuous)
<#>No → ***skip to end of medications questions***¶
<#>Yes¶
<#>Don't know¶
<#>Prefer not to answer
Section Break (Continuous)
Section Break (Continuous)
¶
ME02a. What is the name of the first prescription medication or over-the-counter drug that you take?¶
¶
☐ Label product name: ____¶
¶
☐ Label generic name: ____¶
¶
☐ Don't know ☐ Prefer not to answer¶
¶
ME02b. What is the dosage form?¶ ... [1]

IN-PERSON BASELINE

QUESTIONNAIRE AND ASSESSMENT FORMS

Deleted: SECOND

Deleted: VISIT SCREENING

PID. Participant ID # __________
BA01. Staff ID # __________
BA02. Today's date (MMDDYY): ___________

Blood Pressure:
BA03a. Blood pressure 1st reading, systolic blood pressure: _________ mmHg
BA03b. Blood pressure 1st reading, diastolic blood pressure: __________ mmHg
BA03c. Blood pressure 2nd reading, systolic blood pressure: _________ mmHg
BA03d. Blood pressure 2nd reading, diastolic blood pressure: __________ mmHg
BA03e. Blood pressure 3rd reading, systolic blood pressure: _________ mmHg
BA03f. Blood pressure 3rd reading, diastolic blood pressure: __________ mmHg

Deleted: BA03g. Blood pressure 4th reading, systolic blood pressure: _________ mmHg¶
BA03h. Blood pressure 4th reading, diastolic blood pressure: __________ mmHg¶
BA03i. Blood pressure 5th reading, systolic blood pressure: _________ mmHg¶
BA03j. Blood pressure 5th reading, diastolic blood pressure: __________ mmHg

BA04. Were the 2nd and 3rd systolic blood pressure readings within 20 mmHg of each other?
□ Yes
□ No ***(repeat measurements***

Deleted: 4th and 5th

BA05. Were the 2nd and 3rd diastolic blood pressure readings within 10 mmHg of each other?
□ Yes
□ No ***(repeat measurements)***

Deleted: 4th and 5th

BA06a. Repeated blood pressure 1st reading, systolic blood pressure: _________ mmHg
BA06b. Repeated blood pressure 1st reading, diastolic blood pressure: __________ mmHg
BA06c. Repeated blood pressure 2nd reading, systolic blood pressure: _________ mmHg
BA06d. Repeated blood pressure 2nd reading, diastolic blood pressure: __________ mmHg
BA06e. Repeated blood pressure 3rd reading, systolic blood pressure: _________ mmHg
BA06f. Repeated blood pressure 3rd reading, diastolic blood pressure: __________ mmHg

Deleted: BA06g. Repeated blood pressure 4th reading, systolic blood pressure: _________ mmHg¶
BA06h. Repeated blood pressure 4th reading, diastolic blood pressure: __________ mmHg¶
BA06i. Repeated blood pressure 5th reading, systolic blood pressure: _________ mmHg¶
BA06j. Repeated blood pressure 5th reading, diastolic blood pressure: __________ mmHg¶

BA07. Blood pressure cuff size used: □ S □ Reg □ L □ XL

BA08. Arm that cuff was placed on: □ L □ R

BA09. Height: _______ . __ cm (one decimal place)

BA10. Weight: _______ . __ kg (one decimal place)

Waist Circumference:

BA11a. Measurement 1 _______ . __ cm (one decimal place)
BA11b. Measurement 2 _______ . __ cm (one decimal place)

BA12. Is the difference between Measurement 1 and Measurement 2 greater than 1.0cm?

□ Yes ***(repeat measurements 3&4)***
□ No

BA13a. Measurement 3 _______ . __ cm
BA13b. Measurement 4 _______ . __ cm

Medications (ME)

Comment [SF1]: Moved here from In-Person Screener

ME01. Do you take any prescription medications or over-the-counter drugs?

- ☐ No → ***skip to end of medications questions***
- ☐ Yes
- ☐ Don't know
- ☐ Prefer not to answer

ME02a. What is the name of the first prescription medication or over-the-counter drug that you take?

☐ Label product name: ______________________________

☐ Label generic name: ______________________________

☐ Don't know ☐ Prefer not to answer

ME02b. What is the dosage form?

Oral
- ☐ Pill, tablet, or capsule
- ☐ Sublingual or orally-disintegrating tablet
- ☐ Liquid solution or suspension (drink, syrup)
- ☐ Powder

Topical
- ☐ Liquid, cream, gel, or ointment
- ☐ Ear drops (otic)
- ☐ Eye drops (ophthalmic)
- ☐ Skin patch (transdermal)

Inhaled
- ☐ Inhaler or nebulizer

Injected
- ☐ Injection

Suppository
- ☐ Rectal (e.g., enema)
- ☐ Vaginal (e.g., douche, pessary)

Other:
- ☐ Don't know
- ☐ Prefer not to answer

ME02c. How frequently do you take it?

- ☐ ________ times per day
- ☐ ________ times per week
- ☐ ________ times per month
- ☐ Don't know
- ☐ Prefer not to answer

ME02d. What is the strength? ***(Record strength of how it is actually taken, not how it is prescribed.)***

- ☐ ________ %
- ☐ ________ mg
- ☐ ________ mcg
- ☐ ________ grams
- ☐ ________ I.U.
- ☐ ________ Other unit: ____________
- ☐ Don't know
- ☐ Prefer not to answer

ME02e. Total dosage **per day. *(Record strength of how it is actually taken, not how it is prescribed.)***

- ☐ ________ %
- ☐ ________ mg
- ☐ ________ mcg
- ☐ ________ grams
- ☐ ________ I.U.
- ☐ ________ Other unit: ____________
- ☐ Don't know
- ☐ Prefer not to answer

ME02f. Do you take it regularly or only as needed?

- ☐ Regularly
- ☐ Only as needed
- ☐ Don't know
- ☐ Prefer not to answer

ME02g. For how long have you been taking it?

- ☐ For ________ days
- ☐ For ________ weeks
- ☐ For ________ months
- ☐ For ________ years
- ☐ Don't know
- ☐ Prefer not to answer

ME02h. What is the medication used for?

____

ME02i. ***Interviewer comments:***

____

ME02j. Do you take any other prescription medications or over-the-counter drugs?

- ☐ No → ***skip to end of medications questions***
- ☐ Yes
- ☐ Don't know
- ☐ Prefer not to answer

ME03a. What is the name of the next prescription medication or over-the-counter drug that you take?

☐ Label product name: ____________________

☐ Label generic name: ____________________

☐ Don't know ☐ Prefer not to answer

ME03b. What is the dosage form?

Oral
☐ Pill, tablet, or capsule
☐ Sublingual or orally-disintegrating tablet
☐ Liquid solution or suspension (drink, syrup)
☐ Powder

Topical
☐ Liquid, cream, gel, or ointment
☐ Ear drops (otic)
☐ Eye drops (ophthalmic)
☐ Skin patch (transdermal)

Inhaled
☐ Inhaler or nebulizer

Injected
☐ Injection

Suppository
☐ Rectal (e.g., enema)
☐ Vaginal (e.g., douche, pessary)

Other:
☐ Don't know
☐ Prefer not to answer

ME03c. How frequently do you take it?

☐ ________ times per day
☐ ________ times per week
☐ ________ times per month

☐ Don't know
☐ Prefer not to answer

ME03d. What is the strength? ***(Record strength of how it is actually taken, not how it is prescribed.)***

☐ ________ %
☐ ________ mg
☐ ________ mcg
☐ ________ grams
☐ ________ I.U.

☐ ________ Other unit: ____________
☐ Don't know
☐ Prefer not to answer

ME03e. Total dosage **per day. *(Record strength of how it is actually taken, not how it is prescribed.)***

- ☐ ________ %
- ☐ ________ mg
- ☐ ________ mcg
- ☐ ________ grams
- ☐ ________ I.U.
- ☐ ________ Other unit: ____________
- ☐ Don't know
- ☐ Prefer not to answer

ME3f. Do you take it regularly or only as needed?

- ☐ Regularly
- ☐ Only as needed
- ☐ Don't know
- ☐ Prefer not to answer

ME03g. For how long have you been taking it?

- ☐ For ________ days
- ☐ For ________ weeks
- ☐ For ________ months
- ☐ For ________ years
- ☐ Don't know
- ☐ Prefer not to answer

ME03h. What is the medication used for?

____

ME03i. ***Interviewer comments*:**

____

ME03j. Do you take any other prescription medications or over-the-counter drugs?

- ☐ No → ***skip to end of medications questions***
- ☐ Yes
- ☐ Don't know
- ☐ Prefer not to answer

ME04a. What is the name of the next prescription medication or over-the-counter drug that you take?

☐ Label product name: ____

☐ Label generic name: ____

☐ Don't know ☐ Prefer not to answer

ME04b. What is the dosage form?

Oral
- ☐ Pill, tablet, or capsule
- ☐ Sublingual or orally-disintegrating tablet
- ☐ Liquid solution or suspension (drink, syrup)
- ☐ Powder

Topical
- ☐ Liquid, cream, gel, or ointment
- ☐ Ear drops (otic)
- ☐ Eye drops (ophthalmic)
- ☐ Skin patch (transdermal)

Inhaled
- ☐ Inhaler or nebulizer

Injected
- ☐ Injection

Suppository
- ☐ Rectal (e.g., enema)
- ☐ Vaginal (e.g., douche, pessary)

Other:
- ☐ Don't know
- ☐ Prefer not to answer

ME04c. How frequently do you take it?
- ☐ ________ times per day
- ☐ ________ times per week
- ☐ ________ times per month
- ☐ Don't know
- ☐ Prefer not to answer

ME04d. What is the strength? ***(Record strength of how it is actually taken, not how it is prescribed.)***
- ☐ ________ %
- ☐ ________ mg
- ☐ ________ mcg
- ☐ ________ grams
- ☐ ________ I.U.
- ☐ ________ Other unit: ________
- ☐ Don't know
- ☐ Prefer not to answer

ME04e. Total dosage **per day. *(Record strength of how it is actually taken, not how it is prescribed.)***
- ☐ ________ %
- ☐ ________ mg
- ☐ ________ mcg
- ☐ ________ grams
- ☐ ________ I.U.
- ☐ ________ Other unit: ________
- ☐ Don't know
- ☐ Prefer not to answer

ME04f. Do you take it regularly or only as needed?
- ☐ Regularly
- ☐ Only as needed
- ☐ Don't know
- ☐ Prefer not to answer

ME04g. For how long have you been taking it?

- ☐ For ___________ days
- ☐ For ___________ weeks
- ☐ For ___________ months
- ☐ For ___________ years
- ☐ Don't know
- ☐ Prefer not to answer

ME04h. What is the medication used for?

____

ME04i. ***Interviewer comments*:**

____

ME04j. Do you take any other prescription medications or over-the-counter drugs?

- ☐ No → ***skip to end of medications section***
- ☐ Yes
- ☐ Don't know
- ☐ Prefer not to answer

ME05a. What is the name of the next prescription medication or over-the-counter drug that you take?

☐ Label product name: ____

☐ Label generic name: ____

☐ Don't know ☐ Prefer not to answer

ME05b. What is the dosage form?

Oral
- ☐ Pill, tablet, or capsule
- ☐ Sublingual or orally-disintegrating tablet
- ☐ Liquid solution or suspension (drink, syrup)
- ☐ Powder

Topical
- ☐ Liquid, cream, gel, or ointment
- ☐ Ear drops (otic)
- ☐ Eye drops (ophthalmic)
- ☐ Skin patch (transdermal)

Inhaled
- ☐ Inhaler or nebulizer

Injected
- ☐ Injection

Suppository
- ☐ Rectal (e.g., enema)
- ☐ Vaginal (e.g., douche, pessary)

Other:
- ☐ Don't know
- ☐ Prefer not to answer

ME05c. How frequently do you take it?

- ☐ ________ times per day
- ☐ ________ times per week
- ☐ ________ times per month
- ☐ Don't know
- ☐ Prefer not to answer

ME05d. What is the strength? ***(Record strength of how it is actually taken, not how it is prescribed.)***

- ☐ ________ %
- ☐ ________ mg
- ☐ ________ mcg
- ☐ ________ grams
- ☐ ________ I.U.
- ☐ ________ Other unit: ________
- ☐ Don't know
- ☐ Prefer not to answer

ME05e. Total dosage **per day. *(Record strength of how it is actually taken, not how it is prescribed.)***

- ☐ ________ %
- ☐ ________ mg
- ☐ ________ mcg
- ☐ ________ grams
- ☐ ________ I.U.
- ☐ ________ Other unit: ________
- ☐ Don't know
- ☐ Prefer not to answer

ME05f. Do you take it regularly or only as needed?

- ☐ Regularly
- ☐ Only as needed
- ☐ Don't know
- ☐ Prefer not to answer

ME05g. For how long have you been taking it?

- ☐ For ________ days
- ☐ For ________ weeks
- ☐ For ________ months
- ☐ For ________ years
- ☐ Don't know
- ☐ Prefer not to answer

ME05h. What is the medication used for?

____

ME05i. ***Interviewer comments*:**

____

ME05j. Do you take any other prescription medications or over-the-counter drugs?

- ☐ No → ***skip to end of end of medications questions***
- ☐ Yes
- ☐ Don't know
- ☐ Prefer not to answer

ME06a. What is the name of the next prescription medication or over-the-counter drug that you take?

☐ Label product name: ____________________

☐ Label generic name: ____________________

☐ Don't know

☐ Prefer not to answer

ME06b. What is the dosage form?

Oral
- ☐ Pill, tablet, or capsule
- ☐ Sublingual or orally-disintegrating tablet
- ☐ Liquid solution or suspension (drink, syrup)
- ☐ Powder

Topical
- ☐ Liquid, cream, gel, or ointment
- ☐ Ear drops (otic)
- ☐ Eye drops (ophthalmic)
- ☐ Skin patch (transdermal)

Inhaled
- ☐ Inhaler or nebulizer

Injected
- ☐ Injection

Suppository
- ☐ Rectal (e.g., enema)
- ☐ Vaginal (e.g., douche, pessary)

Other:
- ☐ Don't know
- ☐ Prefer not to answer

ME06c. How frequently do you take it?
- ☐ ________ times per day
- ☐ ________ times per week
- ☐ ________ times per month
- ☐ Don't know
- ☐ Prefer not to answer

ME06d. What is the strength? ***(Record strength of how it is actually taken, not how it is prescribed.)***
- ☐ ________ %
- ☐ ________ mg
- ☐ ________ mcg
- ☐ ________ grams
- ☐ ________ I.U.
- ☐ ________ Other unit: ________
- ☐ Don't know
- ☐ Prefer not to answer

ME06e. Total dosage **per day.** ***(Record strength of how it is actually taken, not how it is prescribed.)***

- ☐ ________ %
- ☐ ________ mg
- ☐ ________ mcg
- ☐ ________ grams
- ☐ ________ I.U.
- ☐ ________ Other unit: ________
- ☐ Don't know
- ☐ Prefer not to answer

ME06f. Do you take it regularly or only as needed?

- ☐ Regularly
- ☐ Only as needed
- ☐ Don't know
- ☐ Prefer not to answer

ME06g. For how long have you been taking it?

- ☐ For ________ days
- ☐ For ________ weeks
- ☐ For ________ months
- ☐ For ________ years
- ☐ Don't know
- ☐ Prefer not to answer

ME06h. What is the medication used for?

____

ME06i. ***Interviewer comments:***

____

ME06i. Do you take any other prescription medications or over-the-counter drugs?

- ☐ No → ***skip to end of medications questions***
- ☐ Yes
- ☐ Don't know
- ☐ Prefer not to answer

ME07a. What is the name of the next prescription medication or over-the-counter drug that you take?

☐ Label product name: ____

☐ Label generic name: ____

- ☐ Don't know
- ☐ Prefer not to answer

ME07b. What is the dosage form?

Oral
- ☐ Pill, tablet, or capsule
- ☐ Sublingual or orally-disintegrating tablet
- ☐ Liquid solution or suspension (drink, syrup)
- ☐ Powder

Topical
- ☐ Liquid, cream, gel, or ointment
- ☐ Ear drops (otic)
- ☐ Eye drops (ophthalmic)
- ☐ Skin patch (transdermal)

Inhaled
- ☐ Inhaler or nebulizer

Injected
- ☐ Injection

Suppository
- ☐ Rectal (e.g., enema)
- ☐ Vaginal (e.g., douche, pessary)

Other:
- ☐ Don't know
- ☐ Prefer not to answer

ME07c. How frequently do you take it?
- ☐ ________ times per day
- ☐ ________ times per week
- ☐ ________ times per month
- ☐ Don't know
- ☐ Prefer not to answer

ME07d. What is the strength? ***(Record strength of how it is actually taken, not how it is prescribed.)***
- ☐ ________ %
- ☐ ________ mg
- ☐ ________ mcg
- ☐ ________ grams
- ☐ ________ I.U.
- ☐ ________ Other unit: ________
- ☐ Don't know
- ☐ Prefer not to answer

ME07e. Total dosage **per day. *(Record strength of how it is actually taken, not how it is prescribed.)***
- ☐ ________ %
- ☐ ________ mg
- ☐ ________ mcg
- ☐ ________ grams
- ☐ ________ I.U.
- ☐ ________ Other unit: ________
- ☐ Don't know
- ☐ Prefer not to answer

ME07f. Do you take it regularly or only as needed?
- ☐ Regularly
- ☐ Only as needed
- ☐ Don't know
- ☐ Prefer not to answer

ME07g For how long have you been taking it?

- ☐ For ________ days
- ☐ For ________ weeks
- ☐ For ________ months
- ☐ For ________ years
- ☐ Don't know
- ☐ Prefer not to answer

ME07h. What is the medication used for?

____

ME07i. ***Interviewer comments***:

____

ME07j. Do you take any other prescription medications or over-the-counter drugs?

- ☐ No → ***skip to end of medications questions***
- ☐ Yes
- ☐ Don't know
- ☐ Prefer not to answer

ME08a. What is the name of the next prescription medication or over-the-counter drug that you take?

- ☐ Label product name: ____
- ☐ Label generic name: ____
- ☐ Don't know
- ☐ Prefer not to answer

ME08b. What is the dosage form?

Oral
- ☐ Pill, tablet, or capsule
- ☐ Sublingual or orally-disintegrating tablet
- ☐ Liquid solution or suspension (drink, syrup)
- ☐ Powder

Topical
- ☐ Liquid, cream, gel, or ointment
- ☐ Ear drops (otic)
- ☐ Eye drops (ophthalmic)
- ☐ Skin patch (transdermal)

Inhaled
- ☐ Inhaler or nebulizer

Injected
- ☐ Injection

Suppository
- ☐ Rectal (e.g., enema)
- ☐ Vaginal (e.g., douche, pessary)

Other:
- ☐ Don't know
- ☐ Prefer not to answer

ME08c. How frequently do you take it?

- ☐ __________ times per day
- ☐ __________ times per week
- ☐ __________ times per month
- ☐ Don't know
- ☐ Prefer not to answer

ME08d. What is the strength? *(Record strength of how it is actually taken, not how it is prescribed.)*

- ☐ __________ %
- ☐ __________ mg
- ☐ __________ mcg
- ☐ __________ grams
- ☐ __________ I.U.
- ☐ __________ Other unit: __________
- ☐ Don't know
- ☐ Prefer not to answer

ME08e. Total dosage **per day. *(Record strength of how it is actually taken, not how it is prescribed.)***

- ☐ __________ %
- ☐ __________ mg
- ☐ __________ mcg
- ☐ __________ grams
- ☐ __________ I.U.
- ☐ __________ Other unit: __________
- ☐ Don't know
- ☐ Prefer not to answer

ME08f. Do you take it regularly or only as needed?

- ☐ Regularly
- ☐ Only as needed
- ☐ Don't know
- ☐ Prefer not to answer

ME08g. For how long have you been taking it?

- ☐ For __________ days
- ☐ For __________ weeks
- ☐ For __________ months
- ☐ For __________ years
- ☐ Don't know
- ☐ Prefer not to answer

ME08h. What is the medication used for?

____

ME08i. ***Interviewer comments***:

____

ME08j. Do you take any other prescription medications or over-the-counter drugs?

- ☐ No → ***skip to end of medications questions***
- ☐ Yes
- ☐ Don't know
- ☐ Prefer not to answer

ME09a. What is the name of the next prescription medication or over-the-counter drug that you take?

☐ Label product name: ______________________________

☐ Label generic name: ______________________________

☐ Don't know ☐ Prefer not to answer

ME09b. What is the dosage form?

Oral
- ☐ Pill, tablet, or capsule
- ☐ Sublingual or orally-disintegrating tablet
- ☐ Liquid solution or suspension (drink, syrup)
- ☐ Powder

Topical
- ☐ Liquid, cream, gel, or ointment
- ☐ Ear drops (otic)
- ☐ Eye drops (ophthalmic)
- ☐ Skin patch (transdermal)

Inhaled
- ☐ Inhaler or nebulizer

Injected
- ☐ Injection

Suppository
- ☐ Rectal (e.g., enema)
- ☐ Vaginal (e.g., douche, pessary)

Other:
- ☐ Don't know
- ☐ Prefer not to answer

ME09c. How frequently do you take it?
- ☐ ________ times per day
- ☐ ________ times per week
- ☐ ________ times per month
- ☐ Don't know
- ☐ Prefer not to answer

ME09d. What is the strength? *(Record strength of how it is actually taken, not how it is prescribed.)*
- ☐ ________ %
- ☐ ________ mg
- ☐ ________ mcg
- ☐ ________ grams
- ☐ ________ I.U.
- ☐ ________ Other unit: ________
- ☐ Don't know
- ☐ Prefer not to answer

ME09e. Total dosage **per day.** ***(Record strength of how it is actually taken, not how it is prescribed.)***

- ☐ ________ %
- ☐ ________ mg
- ☐ ________ mcg
- ☐ ________ grams
- ☐ ________ I.U.
- ☐ ________ Other unit: ____________
- ☐ Don't know
- ☐ Prefer not to answer

ME09f. Do you take it regularly or only as needed?

- ☐ Regularly
- ☐ Only as needed
- ☐ Don't know
- ☐ Prefer not to answer

ME09g. For how long have you been taking it?

- ☐ For ________ days
- ☐ For ________ weeks
- ☐ For ________ months
- ☐ For ________ years
- ☐ Don't know
- ☐ Prefer not to answer

ME09h. What is the medication used for?

____

ME09i. ***Interviewer comments:***

____

ME09j. Do you take any other prescription medications or over-the-counter drugs?

- ☐ No → ***skip to end of medications questions***
- ☐ Yes
- ☐ Don't know
- ☐ Prefer not to answer

ME10a. What is the name of the next prescription medication or over-the-counter drug that you take?

☐ Label product name: ____

☐ Label generic name: ____

☐ Don't know ☐ Prefer not to answer

ME10b. What is the dosage form?

Oral
- ☐ Pill, tablet, or capsule
- ☐ Sublingual or orally-disintegrating tablet
- ☐ Liquid solution or suspension (drink, syrup)
- ☐ Powder

Topical
- ☐ Liquid, cream, gel, or ointment
- ☐ Ear drops (otic)
- ☐ Eye drops (ophthalmic)
- ☐ Skin patch (transdermal)

Inhaled
- ☐ Inhaler or nebulizer

Injected
- ☐ Injection

Suppository
- ☐ Rectal (e.g., enema)
- ☐ Vaginal (e.g., douche, pessary)

Other:
- ☐ Don't know
- ☐ Prefer not to answer

ME10c. How frequently do you take it?
- ☐ ________ times per day
- ☐ ________ times per week
- ☐ ________ times per month
- ☐ Don't know
- ☐ Prefer not to answer

ME10d. What is the strength? ***(Record strength of how it is actually taken, not how it is prescribed.)***
- ☐ ________ %
- ☐ ________ mg
- ☐ ________ mcg
- ☐ ________ grams
- ☐ ________ I.U.
- ☐ ________ Other unit: ________
- ☐ Don't know
- ☐ Prefer not to answer

ME10e. Total dosage **per day. *(Record strength of how it is actually taken, not how it is prescribed.)***
- ☐ ________ %
- ☐ ________ mg
- ☐ ________ mcg
- ☐ ________ grams
- ☐ ________ I.U.
- ☐ ________ Other unit: ________
- ☐ Don't know
- ☐ Prefer not to answer

ME10f. Do you take it regularly or only as needed?
- ☐ Regularly
- ☐ Only as needed
- ☐ Don't know
- ☐ Prefer not to answer

ME10g. For how long have you been taking it?

- ☐ For __________ days
- ☐ For __________ weeks
- ☐ For __________ months
- ☐ For __________ years
- ☐ Don't know
- ☐ Prefer not to answer

ME10h. What is the medication used for?

____

ME10i. ***Interviewer comments***:
____

FOOD FREQUENCY QUESTIONNAIRE:

RA Script to be read to participants: At this time, we are going to have you complete a few forms on your own. The first is a Food Frequency Questionnaire that will ask you about the types of foods and drinks that you consume. It should take around 20 minutes to complete. Please let me know if you have any questions.

Comment [SF2]: At this time, participants will be directed to complete the three page Harvard University Food Frequency Questionnaire (see proof included), known as the "80-out". It is designed to take around 20 minutes to complete and is self-administered with paper and pencil. Once completed, they will proceed to the next section of this assessment (DS_1).

Questions for Participants to Answer on Their Own In-Person on Paper:
For each statement, please place a mark in the column that best describes how you have been feeling.

	Not at all or less than 1 day last week	1 or 2 days last week	3 to 4 days last week	5 to 7 days last week	Nearly every day for two weeks	I do not know	I prefer not to answer
DS_1. My appetite was poor.	☐	☐	☐	☐	☐	☐	☐
DS_2. I could not shake off the blues.	☐	☐	☐	☐	☐	☐	☐
DS_3. I had trouble keeping my mind on what I was doing.	☐	☐	☐	☐	☐	☐	☐
DS_4. I felt depressed.	☐	☐	☐	☐	☐	☐	☐
DS_5. My sleep was restless.	☐	☐	☐	☐	☐	☐	☐
DS_6. I felt sad.	☐	☐	☐	☐	☐	☐	☐
DS_7. I could not get going.	☐	☐	☐	☐	☐	☐	☐
DS_8. Nothing made me happy.	☐	☐	☐	☐	☐	☐	☐
C_9. I felt like a bad person.	☐	☐	☐	☐	☐	☐	☐
DS_10. I lost interest in my usual activities.	☐	☐	☐	☐	☐	☐	☐
DS_11. I slept much more than usual.	☐	☐	☐	☐	☐	☐	☐
DS_12. I felt like I was moving too slowly.	☐	☐	☐	☐	☐	☐	☐
DS_13. I felt fidgety.	☐	☐	☐	☐	☐	☐	☐
DS_14. I wished I were dead.	☐	☐	☐	☐	☐	☐	☐
DS_15. I wanted to hurt myself.	☐	☐	☐	☐	☐	☐	☐
DS_16. I was tired all the time.	☐	☐	☐	☐	☐	☐	☐
DS_17. I did not like myself.	☐	☐	☐	☐	☐	☐	☐
DS_18. I lost a lot of weight without trying to.	☐	☐	☐	☐	☐	☐	☐
DS_19. I had a lot of trouble getting to sleep.	☐	☐	☐	☐	☐	☐	☐
DS_20. I could not focus on the important things.	☐	☐	☐	☐	☐	☐	☐

Below is a list of common symptoms of anxiety. Please carefully read each item in the list. Indicate how much you have been bothered by that symptom during the past month, including today, by marking the box in the corresponding space in the column next to each symptom.

	Not At All	Mildly – it didn't bother me much	Moderately – it wasn't pleasant at all times	Severely – it bothered me a lot	I do not know	I prefer not to answer
BE_1. Numbness or tingling	☐	☐	☐	☐	☐	☐
BE_2. Feeling hot	☐	☐	☐	☐	☐	☐
BE _3. Wobbliness in legs	☐	☐	☐	☐	☐	☐
BE _4. Unable to relax	☐	☐	☐	☐	☐	☐
BE _5. Fear of worst happening	☐	☐	☐	☐	☐	☐
BE _6. Dizzy or lightheaded	☐	☐	☐	☐	☐	☐
BE _7. Heart pounding/racing	☐	☐	☐	☐	☐	☐
BE _8. Unsteady	☐	☐	☐	☐	☐	☐
BE _9. Terrified or afraid	☐	☐	☐	☐	☐	☐
BE _10. Nervous	☐	☐	☐	☐	☐	☐
BE _11. Feeling of choking	☐	☐	☐	☐	☐	☐
BE _12. Hands trembling	☐	☐	☐	☐	☐	☐
BE _13. Shaky/unsteady	☐	☐	☐	☐	☐	☐
BE _14. Fear of losing control	☐	☐	☐	☐	☐	☐
BE _15. Difficulty in breathing	☐	☐	☐	☐	☐	☐
BE _16. Fear of dying	☐	☐	☐	☐	☐	☐
BE _17. Scared	☐	☐	☐	☐	☐	☐
BE _18. Indigestion	☐	☐	☐	☐	☐	☐
BE _19. Faint/lightheaded	☐	☐	☐	☐	☐	☐
BE _20. Face flushed	☐	☐	☐	☐	☐	☐
BE _21. Hot/cold sweats	☐	☐	☐	☐	☐	☐

After questionnaires are complete, participants will be asked to perform the following two computer-based tests:

Attention Control: Attention Network Test (20 minutes)

Attention Network Test (ANT) is a brief computerized battery measuring three independent behavioral components of attention: Conflict resolution (ability to overcome distracting stimuli), spatial Orienting (the benefit of valid spatial pre-cues), and Alerting (the benefit of temporal pre-cues). Efficiency of orienting is examined by changes in RT that accompany cues indicating where the target will occur. The efficiency of the executive conflict resolution network is examined by requiring the subject to respond by pressing two keys indicating the direction (left or right) of a central arrow surrounded by congruent, incongruent or neutral flankers. Moderate to high reliabilities are found for all networks.

ANT data to be entered by RA *prior to* ANT:

PPT Age: _______

PPT Sex: Male / Female

Category: Normal

Diagonal of "subject info" window: 10"

Press "distance between eyes and screen" button to calculate. Should be 23.2.

ANT data to be entered by RA *after* ANT:

PPT Age: _______

Alerting effect (ms): _______

Orienting effect (ms): _______

Conflict effect (ms): _______

Mean RT for correct trials (ms): _______

Mean accuracy (%): _______

Sustained Attention to Response Task (15 minutes)

Deleted: Page Break

The Sustained Attention to Response Task (SART) is a computerized test of sustained attention, response inhibition (executive function) and self-regulation. Subjects are instructed to press a key in response to rapidly displayed integers (1-9) and withhold response to a designated "no-go" integer. SART errors consist of summed commission errors (button press on no-go trial) and omission errors (button not pressed on "go" integers). SART performance is associated with prefrontal cortex functioning, has been found to increase with mindfulness training and is correlated with scores on mindfulness questionnaires (specifically, the Mindful Attention Awareness Scale).

SAFETY PLAN

After the participant has completed the Beck Anxiety Inventory questionnaire and the Centers for Epidemiologic Studies Depressive Symptomatology questionnaire, ask the participant to wait for a few minutes while you check that all forms and assessments are completed. While the participant is waiting in a different room, check the scores according to the criteria below. If any scores trigger the safety plan, move forward with steps below as written.

Beck Anxiety Inventory (BA)

If participant scores ≥26 on the Beck Anxiety Inventory, while having the participant wait, immediately notify the collaborating psychiatrist, Dr. Ellen Flynn (cell phone # 401-258-9829), to determine need for a psychiatric consultation. She has provided her cell phone #, and your phone # will be entered into her phone recognition software to indicate it is a call from the MBHT study. It is likely she will answer the phone. If Dr. Flynn cannot be reached, please leave a phone message for her with your contact information and provide her with the Beck Anxiety Inventory results on the message. If Dr. Flynn is not immediately reachable, or following participant release after consulting with Dr. Flynn, immediately contact the PI, Dr. Eric Loucks (cell phone #401-369-0443).

Depressive Symptomatology (DS)

The CESD-R will be administered during the in-person assessment visits, and scores will be reviewed immediately upon completion of the in-person assessments.

1. Sadness (dysphoria): Question numbers 2,4, 6
2. Loss of Interest (anhedonia): Question numbers 8, 10
3. Appetite: Question numbers 1, 18
4. Sleep: Question numbers 5, 11, 19
5. Thinking / concentration: Question numbers 3, 20
6. Guilt (worthlessness): Question numbers 9, 17
7. Tired (fatigue): Question numbers 7, 16
8. Movement (agitation): Question numbers 12, 13
9. Suicidal ideation: Question numbers 14, 15

Participants are considered to meet criteria for major depressive episode if they have anhedonia or dysphoria nearly every day for the past two weeks, plus symptoms in an additional 4 DSM symptom groups noted as occurring nearly every day for the past two weeks. If participants meet criteria for major depressive episode, while having the participant wait, immediately notify the collaborating psychiatrist, Dr. Ellen Flynn (cell phone # 401-258-9829), to determine need for a psychiatric consultation. She has provided her cell phone #, and your phone # will be entered into her phone recognition software to indicate it is a call from the MBHT study. It is likely she will answer the phone. If Dr. Flynn cannot be reached, please leave a phone message for her with your contact information and provide her with the Depressive Symptomatology results on the message. If Dr. Flynn is not immediately reachable, or following participant release after consulting with Dr. Flynn, immediately contact the PI, Dr. Eric Loucks (cell phone #401-369-0443).

If participants respond having any suicidal ideation (DS questions 14 or 15), perform the following 2 steps:

1. **Immediately call 911 and Dr. Ellen Flynn.**

Specifically, while the participant is in the waiting room, ***call 911 immediately, and tell them:***

"My name is _____. I am working on a research study at Brown University. I have a study participant in the waiting room who has shared that he/she is currently suicidal." Please provide the participants' name to the 911 operator, as requested.

Finally, after calling 911, please call Dr. Ellen Flynn, who is the psychiatrist supporting this study. Her cell phone # is 401-258-9829. Please provide her with the same information as was done during the 911 call.

2. **While speaking calmly with the participant, let them know that you have called 911 and Dr. Flynn, and why (i.e. because we are concerned about you). You can speak with participant to keep him/her in the waiting room if 911 has sent assistance, but the discussion should not be clinical in nature.**

Examples of questions that could be asked in order to keep them in the waiting room:

- "Tell me what is going on."
- "What's happening right now?"
- Tell me more about why you are interesting in being part of this study.
- What are you hoping to get out of this study?

The following information can be provided to study participants.

National Suicide Prevention Lifeline: 1-800-273-8255

Other options are to:

- Call your doctor's office
- Call 911 for emergency services
- Go to the nearest hospital emergency room.

Local Non-Urgent Free or Inexpensive Mental Health Services:
Gateway Healthcare: 401-729-8701
Anchor Counseling Center: 401-475-9979
The Providence Center: 401-276-4020

HOME BASELINE ASSESSMENT

QUESTIONNAIRES ANSWERED BY PARTICIPANTS AT BASELINE (VIA ONLINE OR PAPER FORM)

Questionnaire Table of Contents

Introductory Questions....3
Background Questions....5
Physical Activity....8
Eating Practices....10
Alcohol Consumption....13
Smoking....14
About You....15
Parent's Education....37
Your Childhood Experiences....39
Blood Pressure Medication Use....42
More About You....43
Family History of Hypertension....47
Your Sleep....48
END SCRIPT....48

Introduction

We appreciate you taking the time to participate in this research study. These questionnaires will ask a series of questions on various aspects of your health, health behaviours, family, and other life circumstances. It should take approximately one hour. Please keep in mind that you can refuse to answer any questions that you are not comfortable with.

Introductory Questions

IQ1_01. Please enter the 4-digit ID number you were given. ___ ___ ___ ___.

IQ1_02. What is your main reason for participating in this study?

IQ1_03. What do you care about most?

IQ1_04. What gives you the most pleasure in your life?

IQ1_05. What are your greatest worries?

Please list three personal goals you have for taking this mindfulness program:

PG1_01.____
____
____
____
___

PG1_02. ____
____
____
____

PG1_03. ____
____
____
____
___

Background Questions

BQ1_01. How many years old are you?

BQ1_02. Are you Latino or Hispanic?

☐ No → ***skip to B3***
☐ Yes
☐ I do not know
☐ I prefer not to answer

B1_02a. Which of the following represents your family's country of origin? ***(check all that apply)***

☐ Cuba
☐ Mexico
☐ Puerto Rico
☐ Spain
☐ South America
☐ Columbia
☐ Dominican Republic
☐ Other Central American
☐ Other: ______________________
☐ I do not know
☐ I prefer not to answer

BQ1_03. If you were asked to put yourself into only one of these groups, in which one would you place yourself? ***(select one only):***

☐ Asian
☐ Pacific Islander
☐ African American/Black
☐ Caucasian/White
☐ Native American
☐ Other : ________________________
☐ I do not know
☐ I prefer not to answer

BQ1_04. Which of the following best describes your current work situation? ***(select one only)***

☐ Working full-time
☐ Working part-time
☐ Retired
☐ Unemployed: Looking for work
☐ Unemployed: Not currently looking for work
☐ Unemployed due to disability
☐ Keeping house or raising children full-time
☐ Military
☐ Full-time student
☐ Other:_______________________
☐ I do not know
☐ I prefer not to answer

BQ1_05. What is the highest grade or level of regular school you have completed?

☐ Elementary School
☐ Junior High
☐ High School
☐ College
☐ Graduate School
☐ I do not know
☐ I prefer not to answer

BQ1_06. What is the highest degree you earned? ***(select* <u>one</u> *only)***

☐ Elementary school
☐ Some high school, but no GED
☐ GED
☐ High school
☐ Associate degree (Junior College)
☐ Bachelor's degree
☐ Master's degree
☐ Doctorate (PhD, EdD, etc)
☐ Professional (MD, JD, DDS, DVM, etc.)
☐ Other: ______________________________
☐ I do not know
☐ I prefer not to answer

BQ1_07. Did you ever attend any other school like a technical, vocational, or trade school?

☐ No
☐ Yes
☐ I do not know
☐ I prefer not to answer

BQ1_08. In total, about how many full-time years of education have you had, including 1st grade and all years of school after 1st grade?

________ years

BQ1_09a. Do you currently live alone?

- ☐ No
- ☐ Yes → ***skip to next section***
- ☐ I do not know
- ☐ I prefer not to answer

Deleted: *BQ_10*

BQ1_09b. How many people currently live in your household, including yourself?

____

BQ1_09c. Of these people, how many are under 18?

____

BQ1_09d. Of the adults in your household (including yourself), how many bring income into the household?

____

Physical Activity

Physical activities are activities where you move and increase your heart rate above its resting rate, whether you do them for pleasure, work, or transportation. The following questions ask about the amount and intensity of physical activity you usually do. The intensity of the activity is related to *the amount of energy* you use doing these activities.

Examples of physical activity intensity levels:

Light activities
Your heart beats slightly faster than normal
You can talk and sing

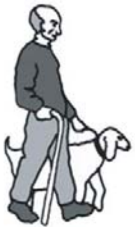

Light exercise

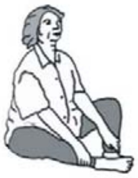

Light stretching

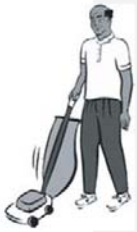

Light vacuuming or yard work

Moderate activities
Your heart beats faster than normal
You can talk but not sing

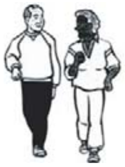

Brisk walking

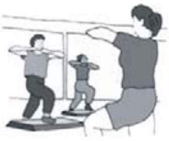

Aerobics class

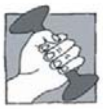

Strength training

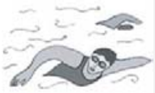

Swim gently

Vigorous activities
Your heart rate increases a lot
You can't talk, or your talking is broken up by large breaths

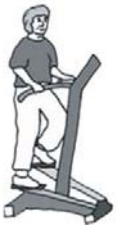

Aerobics classes

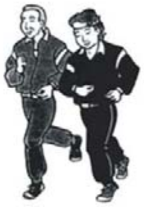

Jogging, Running, or Power Walking

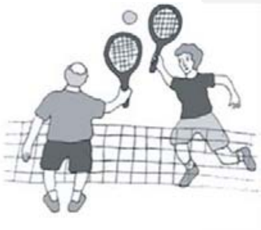

Singles tennis, Racquetball, Pickle ball

How physically active are you?

	YES	NO	I do not know	I prefer not to answer
PA1_01. I rarely or never do any physical activities.	☐	☐	☐	☐
PA1_02. I do some light and/or moderate physical activities, but not every week.	☐	☐	☐	☐
PA1_03. I do some light physical activity every week.	☐	☐	☐	☐
PA1_04. I do moderate physical activity every week but less than 5 days per week or less than 30 minutes on those days.	☐	☐	☐	☐
PA1_05. I do vigorous physical activities every week, but less than 3 days per week or less than 20 minutes on those days.	☐	☐	☐	☐
PA1_06. I do 30 minutes or more per day of moderate physical activities 5 or more days per week.	☐	☐	☐	☐
PA1_07. I do 20 minutes or more per day of vigorous physical activities 3 or more days per week.	☐	☐	☐	☐
PA1_08. I do activities to increase muscle strength, such as lifting weights or calisthenics, once a week or more.	☐	☐	☐	☐
PA1_09. I do activities to improve flexibility, such as stretching or yoga, once a week or more.	☐	☐	☐	☐

Deleted: Diet ¶

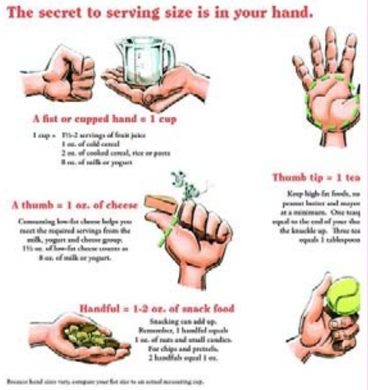

The first set of questions ask about foods you usually eat or drink. For each food, please place an X in the box (e.g. ☒) for the answer that best matches how often you eat or drink it. Remember to count fruit or vegetables that were part of another dish, like a banana on cereal or vegetables in a stew. Also, please pay attention to the serving sizes. For example, one serving size of bread is one slice, so a sandwich would contain 2 servings of bread. Please include all foods you eat, both at home and away from home. Enclosed are pictures of common serving sizes to assist you in filling out your questionnaire. ¶

Deleted: How Often You Eat or Drink This Item ... [1]

Eating Practices

	Definitely True	Mostly True	Mostly False	Definitely False	I Do Not Know	I Prefer Not to Answer
EE1_01. I deliberately take small helpings to control my weight.	☐	☐	☐	☐	☐	☐
EE1_02. I start to eat when I feel anxious.	☐	☐	☐	☐	☐	☐
EE1_03. Sometimes when I start eating, I just can't seem to stop.	☐	☐	☐	☐	☐	☐
EE1_04. When I feel sad, I often eat too much.	☐	☐	☐	☐	☐	☐
EE1_05. I don't eat some foods because they make me fat.	☐	☐	☐	☐	☐	☐
EE1_06. Being with someone who is eating, often makes me want to also eat.	☐	☐	☐	☐	☐	☐
EE1_07. When I feel tense or ''wound up'', I often feel I need to eat.	☐	☐	☐	☐	☐	☐
EE1_08. I often get so hungry that my stomach feels like a bottomless pit.	☐	☐	☐	☐	☐	☐
EE1_09. I'm always so hungry that it's hard for me to stop eating before finishing all of the food on my plate.	☐	☐	☐	☐	☐	☐
EE1_10. When I feel lonely, I console myself by eating.	☐	☐	☐	☐	☐	☐
EE1_11. I consciously hold back on how much I eat at meals to keep from gaining weight.	☐	☐	☐	☐	☐	☐
EE1_12. When I smell a sizzling steak or see a juicy piece of meat, I find it very difficult to keep from eating even if I've just finished a meal.	☐	☐	☐	☐	☐	☐
EE1_13. I'm always hungry enough to eat at any time.	☐	☐	☐	☐	☐	☐
EE1_14. If I feel nervous, I try to calm down by eating.	☐	☐	☐	☐	☐	☐
EE1_15. When I see something that looks very delicious, I often get so hungry that I have to eat right away.	☐	☐	☐	☐	☐	☐
EE1_16. When I feel depressed, I want to eat.	☐	☐	☐	☐	☐	☐

	Almost Never	Seldom	Usually	Almost Always	I Do Not Know	I Prefer Not to Answer
EE1_17. How often do you avoid ‘stocking up’’ on tempting foods?	☐	☐	☐	☐	☐	☐

	Unlikely	A little likely	Somewhat likely	Very likely	I Do Not Know	I Prefer Not to Answer
EE1_18. How likely are you to make an effort to eat less than you want?	☐	☐	☐	☐	☐	☐

	Never	Rarely	Sometimes	At least once a week	I Do Not Know	I Prefer Not to Answer
EE1_19. Do you go on eating binges even though you’re not hungry?	☐	☐	☐	☐	☐	☐

	Only at mealtimes	Sometimes between meals	Often between meals	Almost always	I Do Not Know	I Prefer Not to Answer
EE1_20. How often do you feel hungry?	☐	☐	☐	☐	☐	☐

	1	2	3	4	5	6	7	8	I Do Not Know	I Prefer Not to Answer
EE1_21. On a scale from 1 to 8, where 1 means no restraint in eating and 8 means total restraint, what number would you give yourself? Mark the number that best applies to you:	☐	☐	☐	☐	☐	☐	☐	☐	☐	☐

Table Salt Use:

TS1_01 Please report your average total use, during the past year, of "salt added at the table". Would you say…

- Never .... □
- Less than once per month .... □
- 1-3 shakes per month .... □
- 1 shake per week .... □
- 2-4 shakes per week .... □
- 5-6 shakes per week .... □
- 1 shake per day .... □
- 2-3 shakes per day .... □
- 4-5 shakes per day .... □
- 6+ shakes per day .... □

Alcohol Consumption

A drink of alcohol is defined as 1 can or bottle of beer, 1 glass of wine, 1 can or bottle of wine cooler, 1 cocktail, or 1 shot of liquor.

AC1_01. During the past 30 days, how many days per week or per month did you have at least 1 drink of any alcoholic beverage? [if none, ***skip to next section***]

Deleted: ***STOP***

____

AC1_02. On the days when you drank, about how many drinks did you drink on average?

____

AC1_03. ***Men:*** Considering all types of alcoholic beverages, how many times during the past 30 days did you have **5** or more drinks on an occasion?

Women: Considering all types of alcoholic beverages, how many times during the past 30 days did you have ***4*** or more drinks on an occasion?

____

Smoking

SM1_01. Have you smoked at least 100 cigarettes in your entire life?

- ☐ Yes
- ☐ No
- ☐ I Do Not Know
- ☐ Prefer not to answer

SM1_02. Did you ever become a daily smoker (that is, smoke every day or nearly every day for two months or longer)?

- ☐ Yes
- ☐ No → ***skip to next section***
- ☐ I Do Not Know
- ☐ Prefer not to answer

Deleted: *SC1_01*

SM1_03. How old were you when you last smoked daily?

Age ______ (in years)

☐ I Do Not Know ☐ Prefer not to answer ☐ Still smoking daily

SM1_04. Do you smoke cigarettes now?

- ☐ Yes
- ☐ No → ***skip to next section***
- ☐ I Do Not Know
- ☐ Prefer not to answer

SM1_04a. How many cigarettes per day do you smoke? (One pack equals 20 cigarettes)

Number of cigarettes ________

- ☐ I Do Not Know
- ☐ Prefer not to answer

About You

Please bring to mind a type of very tasty food that may contribute to hypertension through high salt intake or through eating too many calories (e.g., sweet sugary dessert, salty snack foods, etc.).

Think about the LAST WEEK you MOST WANTED this type of food. For each item, select a number (0 to 10) to indicate your rating.

At that time…	Not at All 0	1	2	3	4	5	6	7	8	9	Extremely 10
1. …how much did you want it?	□	□	□	□	□	□	□	□	□	□	□
2. …how much did you need it?	□	□	□	□	□	□	□	□	□	□	□
3. …how strong was the urge to have it?	□	□	□	□	□	□	□	□	□	□	□
At that time, how vividly did you…											
4. …picture it?	□	□	□	□	□	□	□	□	□	□	□
5. …imagine its taste?	□	□	□	□	□	□	□	□	□	□	□
6. …imagine its smell?	□	□	□	□	□	□	□	□	□	□	□
7. …imagine what it would feel like in your mouth or throat?	□	□	□	□	□	□	□	□	□	□	□
8. …imagine how your body would feel?	□	□	□	□	□	□	□	□	□	□	□
At that time…											
9. …how hard were you trying not to think about it?	□	□	□	□	□	□	□	□	□	□	□
10. …how intrusive were the thoughts?	□	□	□	□	□	□	□	□	□	□	□
11. …how hard was it to think about anything else?	□	□	□	□	□	□	□	□	□	□	□

Please bring to mind any times in the LAST WEEK when you had a desire to do sedentary activities (e.g., read a book, watch a movie, be on the computer, etc.) instead of physical activities (e.g., walking, gardening, exercise).

Think about the LAST WEEK you MOST WANTED to do a sedentary activity. For each item, select a number (0 to 10) to indicate your rating.

At that time…	Not at All 0	1	2	3	4	5	6	7	8	9	Extremely 10
1. …how much did you want it?	☐	☐	☐	☐	☐	☐	☐	☐	☐	☐	☐
2. …how much did you need it?	☐	☐	☐	☐	☐	☐	☐	☐	☐	☐	☐
3. …how strong was the urge to have it?	☐	☐	☐	☐	☐	☐	☐	☐	☐	☐	☐
At that time, how vividly did you…											
4. …picture it?	☐	☐	☐	☐	☐	☐	☐	☐	☐	☐	☐
5. …imagine how your body would feel?	☐	☐	☐	☐	☐	☐	☐	☐	☐	☐	☐
At that time…											
6. …how hard were you trying not to think about it?	☐	☐	☐	☐	☐	☐	☐	☐	☐	☐	☐
7. …how intrusive were the thoughts?	☐	☐	☐	☐	☐	☐	☐	☐	☐	☐	☐
8. …how hard was it to think about anything else?	☐	☐	☐	☐	☐	☐	☐	☐	☐	☐	☐

Please bring to mind any times in the LAST WEEK when you had a desire to drink alcohol, such as wine, beer or spirits.

Think about the LAST WEEK you MOST WANTED alcohol. For each item, select a number (0 to 10) to indicate your rating.

At that time…	Not at All 0	1	2	3	4	5	6	7	8	9	Extremely 10
1. …how much did you want it?	☐	☐	☐	☐	☐	☐	☐	☐	☐	☐	☐
2. …how much did you need it?	☐	☐	☐	☐	☐	☐	☐	☐	☐	☐	☐
3. …how strong was the urge to have it?	☐	☐	☐	☐	☐	☐	☐	☐	☐	☐	☐
At that time, how vividly did you…											
4. …picture it?	☐	☐	☐	☐	☐	☐	☐	☐	☐	☐	☐
5. …imagine its taste?	☐	☐	☐	☐	☐	☐	☐	☐	☐	☐	☐
6. …imagine its smell?	☐	☐	☐	☐	☐	☐	☐	☐	☐	☐	☐
7. …imagine what it would feel like in your mouth or throat?	☐	☐	☐	☐	☐	☐	☐	☐	☐	☐	☐
8. …imagine how your body would feel?	☐	☐	☐	☐	☐	☐	☐	☐	☐	☐	☐
At that time…											
9. …how hard were you trying not to think about it?	☐	☐	☐	☐	☐	☐	☐	☐	☐	☐	☐
10. …how intrusive were the thoughts?	☐	☐	☐	☐	☐	☐	☐	☐	☐	☐	☐
11. …how hard was it to think about anything else?	☐	☐	☐	☐	☐	☐	☐	☐	☐	☐	☐

Using the scale provided, please indicate how much each of the following statements reflects how you typically are.

	Not at all	A little	Somewhat	Fair Amount	Very much	I do not know	I prefer not to answer
SC1_01. I am good at resisting temptation.	☐	☐	☐	☐	☐	☐	☐
SC1_02. I have a hard time breaking bad habits.	☐	☐	☐	☐	☐	☐	☐
SC1_03. I am lazy.	☐	☐	☐	☐	☐	☐	☐
SC1_04. I say inappropriate things.	☐	☐	☐	☐	☐	☐	☐
SC1_05. I do certain things that are bad for me, if they are fun.	☐	☐	☐	☐	☐	☐	☐
SC1_06. I refuse things that are bad for me.	☐	☐	☐	☐	☐	☐	☐
SC1_07. I wish I had more self-discipline.	☐	☐	☐	☐	☐	☐	☐
SC1_08. People would say that I have iron self-discipline.	☐	☐	☐	☐	☐	☐	☐
SC1_09. Pleasure and fun sometimes keep me from getting work done.	☐	☐	☐	☐	☐	☐	☐
SC1_10. I have trouble concentrating.	☐	☐	☐	☐	☐	☐	☐
SC1_11. I am able to work effectively toward long-term goals.	☐	☐	☐	☐	☐	☐	☐
SC1_12. Sometimes I can't stop myself from doing something, even if I know it is wrong.	☐	☐	☐	☐	☐	☐	☐
SC1_13. I often act without thinking through all the alternatives.	☐	☐	☐	☐	☐	☐	☐

Please read each statement carefully before answering. To the left of each item, indicate how often you behave in the stated manner, using the following scale:

	Almost never	Not very often	Sometimes	Frequently	Almost always	I Do Not Know	Prefer not to answer
CO1_O1. When I fail at something important to me, I become consumed by feelings of inadequacy.							
CO1_02. I try to be understanding and patient towards those aspects of my personality I don't like.							
CO1_O3. When something painful happens I try to take a balanced view of the situation.							
CO1_O4. When I'm feeling down, I tend to feel like most other people are probably happier than I am.							
CO1_O5. I try to see my failings as part of the human condition.							
CO1_O6. When I'm going through a very hard time, I give myself the caring and tenderness I need.							
CO1_O7. When something upsets me I try to keep my emotions in balance.							
CO1_O8. When I fail at something that's important to me, I tend to feel alone in my failure							
CO1_O9. When I'm feeling down I tend to obsess and fixate on everything that's wrong.							

	Almost never	Not very often	Sometimes	Frequently	Almost always	I Do Not Know	Prefer not to answer
CO1_10. When I feel inadequate in some way, I try to remind myself that feelings of inadequacy are shared by most people.							
CO1_11. I'm disapproving and judgmental about my own flaws and inadequacies.							
CO1_12. I'm intolerant and impatient towards those aspects of my personality I don't like.							

The questions in this scale ask you about your feelings and thoughts during the last month. In each case, you will be asked to indicate by circling how often you felt or thought a certain way.

	Never	Almost Never	Sometimes	Fairly Often	Often	I do not know	I prefer not to answer
PS1_01. In the last month, how often have you been upset because of something that happened unexpectedly?	☐	☐	☐	☐	☐	☐	☐
PS1_02. In the last month, how often have you felt that you were unable to control the important things in your life?	☐	☐	☐	☐	☐	☐	☐
PS1_03. In the last month, how often have you felt nervous and "stressed"?	☐	☐	☐	☐	☐	☐	☐
PS1_04. In the last month, how often have you felt confident about your ability to handle your personal problems?	☐	☐	☐	☐	☐	☐	☐
PS1_05. In the last month, how often have you felt that things were going your way?	☐	☐	☐	☐	☐	☐	☐
PS1_06. In the last month, how often have you found that you could not cope with all the things that you had to do?	☐	☐	☐	☐	☐	☐	☐
PS1_07. In the last month, how often have you been able to control irritations in your life?	☐	☐	☐	☐	☐	☐	☐
PS1_08. In the last month, how often have you felt that you were on top of things?	☐	☐	☐	☐	☐	☐	☐
PS1_09. In the last month, how often have you been angered because of things that were outside of your control?	☐	☐	☐	☐	☐	☐	☐
PS1_10. In the last month, how often have you felt difficulties were piling up so high that you could not overcome them?	☐	☐	☐	☐	☐	☐	☐

Please indicate how often the following statements apply to you by checking the box that best describes your experience.

Comment [D1]: This section (ER1) was moved up in the questionnaire. None of the individual items have changed.

	Almost Never (0-10%)	Sometimes (11-35%)	About Half The Time (36-65%)	Most of the Time (66-90%)	Almost Always (91-100%)	I Do Not Know	I Prefer Not to Answer
ER1_01. I am clear about my feelings.	☐	☐	☐	☐	☐	☐	☐
ER1_02. I pay attention to how I feel.	☐	☐	☐	☐	☐	☐	☐
ER1_03. I experience my emotions as overwhelming and out of control.	☐	☐	☐	☐	☐	☐	☐
ER1_04. I have no idea how I am feeling.	☐	☐	☐	☐	☐	☐	☐
ER1_05. I have difficulty making sense out of my feelings.	☐	☐	☐	☐	☐	☐	☐
ER1_06. I am attentive to my feelings.	☐	☐	☐	☐	☐	☐	☐
ER1_07. I know exactly how I am feeling.	☐	☐	☐	☐	☐	☐	☐
ER1_08. I care about what I am feeling.	☐	☐	☐	☐	☐	☐	☐
ER1_09. I am confused about how I feel.	☐	☐	☐	☐	☐	☐	☐
ER1_10. When I'm upset, I acknowledge my emotions.	☐	☐	☐	☐	☐	☐	☐
ER1_11. When I'm upset, I become angry with myself for feeling that way.	☐	☐	☐	☐	☐	☐	☐
ER1_12. When I'm upset, I become embarrassed for feeling that way.	☐	☐	☐	☐	☐	☐	☐

	Almost Never (0-10%)	Sometimes (11-35%)	About Half The Time (36-65%)	Most of the Time (66-90%)	Almost Always (91-100%)	I Do Not Know	I Prefer Not to Answer
ER1_13. When I'm upset, I have difficulty getting work done.	☐	☐	☐	☐	☐	☐	☐
ER1_14. When I'm upset, I become out of control.	☐	☐	☐	☐	☐	☐	☐
ER1_15. When I'm upset, I believe that I will remain that way for a long time.	☐	☐	☐	☐	☐	☐	☐
ER1_16. When I'm upset, I believe that I will end up feeling very depressed.	☐	☐	☐	☐	☐	☐	☐
ER1_17. When I'm upset, I believe that my feelings are valid and important.	☐	☐	☐	☐	☐	☐	☐
ER1_18. When I'm upset, I have difficulty focusing on other things.	☐	☐	☐	☐	☐	☐	☐
ER1_19. When I'm upset, I feel out of control.	☐	☐	☐	☐	☐	☐	☐
ER1_20. When I'm upset, I can still get things done.	☐	☐	☐	☐	☐	☐	☐
ER1_21. When I'm upset, I feel ashamed at myself for feeling that way.	☐	☐	☐	☐	☐	☐	☐
ER1_22. When I'm upset, I know that I can find a way to eventually feel better.	☐	☐	☐	☐	☐	☐	☐

	Almost Never (0-10%)	Sometimes (11-35%)	About Half The Time (36-65%)	Most of the Time (66-90%)	Almost Always (91-100%)	I Do Not Know	I Prefer Not to Answer
ER1_23. When I'm upset, I feel like I am weak.	☐	☐	☐	☐	☐	☐	☐
ER1_24. When I'm upset, I feel like I can remain in control of my behaviours.	☐	☐	☐	☐	☐	☐	☐
ER1_25. When I'm upset, I feel guilty for feeling that way.	☐	☐	☐	☐	☐	☐	☐
ER1_26. When I'm upset, I have difficulty concentrating.	☐	☐	☐	☐	☐	☐	☐
ER1_27. When I'm upset, I have difficulty controlling my behaviours.	☐	☐	☐	☐	☐	☐	☐
ER1_28. When I'm upset, I believe there is nothing I can do to make myself feel better.	☐	☐	☐	☐	☐	☐	☐
ER1_29. When I'm upset, I become irritated at myself for feeling that way.	☐	☐	☐	☐	☐	☐	☐
ER1_30. When I'm upset, I start to feel very bad about myself.	☐	☐	☐	☐	☐	☐	☐
ER1_31. When I'm upset, I believe that wallowing in it is all I can do.	☐	☐	☐	☐	☐	☐	☐
ER1_32. When I'm upset, I lose control over my behaviour.	☐	☐	☐	☐	☐	☐	☐

	Almost Never (0-10%)	Sometimes (11-35%)	About Half The Time (36-65%)	Most of the Time (66-90%)	Almost Always (91-100%)	I Do Not Know	I Prefer Not to Answer
ER1_33. When I'm upset, I have difficulty thinking about anything else.	☐	☐	☐	☐	☐	☐	☐
ER1_34. When I'm upset I take time to figure out what I'm really feeling.	☐	☐	☐	☐	☐	☐	☐
ER1_35. When I'm upset, it takes me a long time to feel better.	☐	☐	☐	☐	☐	☐	☐
ER1_36. When I'm upset, my emotions feel overwhelming.	☐	☐	☐	☐	☐	☐	☐

This scale is made up of a list of statements each of which may or may not be true about you. For each statement, select “definitely true” if you are sure it is true about you and “probably true” if you think it is true but are not absolutely certain. Similarly, you should select “definitely false” if you are sure the statement is false and “probably false” if you think it is false but are not absolutely certain.

Comment [SF2]: NEW section

	Definitely False	Probably False	Probably True	Definitely True	I do not know	I prefer not to answer
**IS1_01** If I wanted to go on a trip for a day (for example, to the country or mountains), I would have a hard time finding someone to go with me.	☐	☐	☐	☐	☐	☐
**IS1_02** I feel that there is no one I can share my most private worries and fears with.	☐	☐	☐	☐	☐	☐
**IS1_03** If I were sick, I could easily find someone to help me with my daily chores.	☐	☐	☐	☐	☐	☐
**IS1_04** There is someone I can turn to for advice about handling problems with my family.	☐	☐	☐	☐	☐	☐
**IS1_05** If I decide one afternoon that I would like to go to a movie that evening, I could easily find someone to go with me.	☐	☐	☐	☐	☐	☐
**IS1_06** When I need suggestions on how to deal with a personal problem, I know someone I can turn to.	☐	☐	☐	☐	☐	☐
**IS1_07** I don’t often get invited to do things with others.	☐	☐	☐	☐	☐	☐
**IS1_08** If I had to go out of town for a few weeks, it would be difficult to find someone who would look after my house or apartment (the plants, pets, garden, etc.).	☐	☐	☐	☐	☐	☐
**IS1_09** If I wanted to have lunch with someone, I could easily find someone to join me.	☐	☐	☐	☐	☐	☐

	Definitely False	Probably False	Probably True	Definitely True	I do not know	I prefer not to answer
**IS1_10** If I was stranded 10 miles from home, there is someone I could call who could come and get me.	☐	☐	☐	☐	☐	☐
**IS1_11** If a family crisis arose, it would be difficult to find someone who could give me good advice about how to handle it.	☐	☐	☐	☐	☐	☐
**IS1_12** If I needed some help in moving to a new house or apartment, I would have a hard time finding someone to help me.	☐	☐	☐	☐	☐	☐

Please indicate how often each of the statements below is descriptive of you.

Comment [D3]: This section (LS1) was moved here. None of the individual items have changed.

Deleted: INSTRUCTIONS: I

**Statement**	Never	Rarely	Sometimes	Often	I Do Not Know	Prefer not to answer
LS1_01. I feel in tune with the people around me	☐	☐	☐	☐	☐	s
LS1_02. I lack companionship	☐	☐	☐	☐	☐	☐
LS1_03. There is no one I can turn to	☐	☐	☐	☐	☐	☐
LS1_04. I do not feel alone	☐	☐	☐	☐	☐	☐
LS1_05. I feel part of a group of friends	☐	☐	☐	☐	☐	☐
LS1_06. I have a lot in common with the people around me	☐	☐	☐	☐	☐	☐
LS1_07. I am no longer close to anyone	☐	☐	☐	☐	☐	☐
LS1_08. My interests and ideas are not shared by those around me	☐	☐	☐	☐	☐	☐
LS1_09. I am an outgoing person	☐	☐	☐	☐	☐	☐
LS1_10. There are people I feel close to	☐	☐	☐	☐	☐	☐
LS1_11. I feel left out	☐	☐	☐	☐	☐	☐
LS1_12. My social relationships are superficial	☐	☐	☐	☐	☐	☐
LS1_13. No one really knows me well	☐	☐	☐	☐	☐	☐
LS1_14. I feel isolated from others	☐	☐	☐	☐	☐	☐
LS1_15. I can find companionship when I want it	☐	☐	☐	☐	☐	☐
LS1_16. There are people who really understand me	☐	☐	☐	☐	☐	☐
LS1_17. I am unhappy being so withdrawn	☐	☐	☐	☐	☐	☐
LS1_18. People are around me but not with me	☐	☐	☐	☐	☐	☐
LS1_19. There are people I can talk to	☐	☐	☐	☐	☐	☐
LS1_20. There are people I can turn to	☐	☐	☐	☐	☐	☐

Below you will find a list of statements. Please indicate how often each statement applies to you generally in daily life.

Comment [D4]: The following three sections (IA1, RD1, and CR1) were moved here from later in the survey. None of the individual items have changed.

	0- Never	1	2	3	4	5 - Alway
IA1_01. When I am tense I notice where the tension is located in my body.	☐	☐	☐	☐	☐	☐
IA1_02. I notice when I am uncomfortable in my body.	☐	☐	☐	☐	☐	☐
IA1_03. I notice where in my body I am comfortable.	☐	☐	☐	☐	☐	☐
IA1_04. I notice changes in my breathing, such as whether it slows down or speeds up.	☐	☐	☐	☐	☐	☐
IA1_05. I do not notice (I ignore) physical tension or discomfort until they become more severe.	☐	☐	☐	☐	☐	☐
IA1_06. I distract myself from sensations of discomfort.	☐	☐	☐	☐	☐	☐
IA1_07. When I feel pain or discomfort, I try to power through it.	☐	☐	☐	☐	☐	☐
IA1_08. When I feel physical pain, I become upset.	☐	☐	☐	☐	☐	☐
IA1_09. I start to worry that something is wrong if I feel any discomfort.	☐	☐	☐	☐	☐	☐
IA1_10. I can notice an unpleasant body sensation without worrying about it.	☐	☐	☐	☐	☐	☐
IA1_11. I can pay attention to my breath without being distracted by things happening around me.	☐	☐	☐	☐	☐	☐
IA1_12. I can maintain awareness of my inner bodily sensations even when there is a lot going on around me.	☐	☐	☐	☐	☐	☐
IA1_13. When I am in conversation with someone, I can pay attention to my posture.	☐	☐	☐	☐	☐	☐
IA1_14. I can return awareness to my body if I am distracted.	☐	☐	☐	☐	☐	☐
IA1_15. I can refocus my attention from thinking to sensing my body.	☐	☐	☐	☐	☐	☐
IA1_16. I can maintain awareness of my whole body even when a part of me is in pain or discomfort.	☐	☐	☐	☐	☐	☐
IA1_17. I am able to consciously focus on my body as a whole.	☐	☐	☐	☐	☐	☐
IA1_18. I notice how my body changes when I am angry.	☐	☐	☐	☐	☐	☐
IA1_19. When something is wrong in my life I can feel it in my body.	☐	☐	☐	☐	☐	☐
IA1_20. I notice that my body feels different after a peaceful experience.	☐	☐	☐	☐	☐	☐
IA1_21. I notice that my breathing becomes free and easy when I feel comfortable.	☐	☐	☐	☐	☐	☐
IA1_22. I notice how my body changes when I feel happy / joyful.	☐	☐	☐	☐	☐	☐

	0- Never	1	2	3	4	5 - Always
IA1_23. When I feel overwhelmed I can find a calm place inside.	☐	☐	☐	☐	☐	☐
IA1_24. When I bring awareness to my body I feel a sense of calm.	☐	☐	☐	☐	☐	☐
IA1_25. I can use my breath to reduce tension.	☐	☐	☐	☐	☐	☐
IA1_26. When I am caught up in thoughts, I can calm my mind by focusing on my body/breathing.	☐	☐	☐	☐	☐	☐
IA1_27. I listen for information from my body about my emotional state.	☐	☐	☐	☐	☐	☐
IA1_28. When I am upset, I take time to explore how my body feels.	☐	☐	☐	☐	☐	☐
IA1_29. I listen to my body to inform me about what to do.	☐	☐	☐	☐	☐	☐
IA1_30. I am at home in my body.	☐	☐	☐	☐	☐	☐
IA1_31. I feel my body is a safe place.	☐	☐	☐	☐	☐	☐
IA1_32. I trust my body sensations.	☐	☐	☐	☐	☐	☐

We are interested in your recent experiences. Below is a list of things that people sometimes experience. Next to each item are five choices: “never”, “rarely”, “sometimes”, “often”, and “all the time”. Please choose one of these to indicate how much you currently have experiences similar to those described.

Deleted: darken

Please do not spend too long on each item–it is your first response that we are interested in. Please be sure to answer every item.

	Never	Rarely	Sometimes	Often	All the time
RD1_01. I think about what will happen in the future.	☐	☐	☐	☐	☐
RD1_02. I remind myself that thoughts aren’t facts.	☐	☐	☐	☐	☐
RD1_03. I am better able to accept myself as I am.	☐	☐	☐	☐	☐
RD1_04. I notice all sorts of little things and details in the world around me.	☐	☐	☐	☐	☐
RD1_05. I am kinder to myself when things go wrong.	☐	☐	☐	☐	☐
RD1_06. I can slow my thinking at times of stress.	☐	☐	☐	☐	☐
RD1_07. I wonder what kind of person I really am.	☐	☐	☐	☐	☐
RD1_08. I am not so easily carried away by my thoughts and feelings.	☐	☐	☐	☐	☐
RD1_09. I notice that I don’t take difficulties so personally.	☐	☐	☐	☐	☐
RD1_10. I can separate myself from my thoughts and feelings.	☐	☐	☐	☐	☐
RD1_11. I analyze why things turn out the way they do.	☐	☐	☐	☐	☐
RD1_12. I can take time to respond to difficulties.	☐	☐	☐	☐	☐
RD1_13. I think over and over again about what others have said to me.	☐	☐	☐	☐	☐
RD1_14. I can treat myself kindly.	☐	☐	☐	☐	☐
RD1_15. I can observe unpleasant feelings without being drawn into them.	☐	☐	☐	☐	☐
RD1_16. I have the sense that I am fully aware of what is going on around me and inside me.	☐	☐	☐	☐	☐
RD1_17. I can actually see that I am not my thoughts.	☐	☐	☐	☐	☐
RD1_18. I am consciously aware of a sense of my body as a whole.	☐	☐	☐	☐	☐
RD1_19. I think about the ways in which I am different from other people.	☐	☐	☐	☐	☐
RD1_20. I view things from a wider perspective.	☐	☐	☐	☐	☐

How do you cope with events? Everyone gets confronted with negative or unpleasant events now and then and everyone responds to them in his or her own way. By the following questions you are asked to indicate what you generally think, when you experience negative or unpleasant events.

	(Almost) Never	Some-times	Regularly	Often	(Almost) Always
CR1_01. I think that I have to accept that this has happened.	◻	◻	◻	◻	◻
CR1_02. I often think about how I feel about what I have experienced.	◻	◻	◻	◻	◻
CR1_03. I think I can learn something from the situation.	◻	◻	◻	◻	◻
CR1_04. I feel that I am the one who is responsible for what has happened.	◻	◻	◻	◻	◻
CR1_05. I think that I have to accept the situation.	◻	◻	◻	◻	◻
CR1_06. I am preoccupied with what I think and feel about what I have experienced.	◻	◻	◻	◻	◻
CR1_07. I think of pleasant things that have nothing to do with it.	◻	◻	◻	◻	◻
CR1_08. I think that I can become a stronger person as a result of what has happened.	◻	◻	◻	◻	◻
CR1_09. I keep thinking about how terrible it is what I have experienced.	◻	◻	◻	◻	◻
CR1_10. I feel that others are responsible for what has happened.	◻	◻	◻	◻	◻
CR1_11. I think of something nice instead of what has happened.	◻	◻	◻	◻	◻
CR1_12. I think about how to change the situation.	◻	◻	◻	◻	◻
CR1_13. I think that it hasn't been too bad compared to other things.	◻	◻	◻	◻	◻
CR1_14. I think that basically the cause must lie within myself.	◻	◻	◻	◻	◻
CR1_15. I think about a plan of what I can do best.	◻	◻	◻	◻	◻
CR1_16. I tell myself that there are worse things in life.	◻	◻	◻	◻	◻
CR1_17. I continually think how horrible the situation has been.	◻	◻	◻	◻	◻
CR1_18. I feel that basically the cause lies with others.	◻	◻	◻	◻	◻

Instructions: Below is a collection of statements about your everyday experience. Using the scale below, please indicate how frequently or infrequently you currently have each experience. Please answer according to what really reflects your experience rather than what you think your experience should be. Please treat each item separately from every other item.

Please indicate the degree to which you agree with each of the following items using the scale below. Simply check your response to each item

	**Almost always**	**Very frequently**	**Somewhat frequently**	**Somewhat infrequently**	**Very infrequently**	**Almost never**	**I do not know**	**I prefer not to answer**
MA1_01. I could be experiencing some emotion and not be conscious of it until some time later.	☐	☐	☐	☐	☐	☐	☐	☐
MA1_02. I break or spill things because of carelessness, not paying attention, or thinking of something else.	☐	☐	☐	☐	☐	☐	☐	☐
MA1_03. I find it difficult to stay focused on what's happening in the present.	☐	☐	☐	☐	☐	☐	☐	☐
MA1_04. I tend to walk quickly to get where I'm going without paying attention to what I experience along the way.	☐	☐	☐	☐	☐	☐	☐	☐
MA1_05. I tend not to notice feelings of physical tension or discomfort until they really grab my attention.	☐	☐	☐	☐	☐	☐	☐	☐
MA1_06. I forget a person's name almost as soon as I've been told it for the first time.	☐	☐	☐	☐	☐	☐	☐	☐

	Almost always	Very frequently	Somewhat frequently	Somewhat infrequently	Very infrequently	Almost never	I do not know	I prefer not to answer
MA1_07. It seems I am "running on automatic" without much awareness of what I'm doing.	☐	☐	☐	☐	☐	☐	☐	☐
MA1_08. I rush through activities without being really attentive to them.	☐	☐	☐	☐	☐	☐	☐	☐
MA1_09. I get so focused on the goal I want to achieve that I lose touch with what I am doing right now to get there.	☐	☐	☐	☐	☐	☐	☐	☐
MA1_10. I do jobs or tasks automatically, without being aware of what I'm doing.	☐	☐	☐	☐	☐	☐	☐	☐
MA1_11. I find myself listening to someone with one ear, doing something else at the same time.	☐	☐	☐	☐	☐	☐	☐	☐
MA1_12. I drive places on "automatic pilot" and then wonder why I went there.	☐	☐	☐	☐	☐	☐	☐	☐
MA1_13. I find myself preoccupied with the future or the past.	☐	☐	☐	☐	☐	☐	☐	☐
MA1_14. I find myself doing things without paying attention.	☐	☐	☐	☐	☐	☐	☐	☐
MA1_15. I snack without being aware that I'm eating.	☐	☐	☐	☐	☐	☐	☐	☐

Please rate each of the following statements using the scale provided. Write the number in the blank that best describes *your own opinion* of what is *generally true for you*.

	Never	Almost Never	Sometimes	Fairly Often	Often	I do not know	I prefer not to answer
FF1_01. When I'm walking, I deliberately notice the sensations of my body moving.	☐	☐	☐	☐	☐	☐	☐
FF1_02. I'm good at finding words to describe my feelings.	☐	☐	☐	☐	☐	☐	☐
FF1_03. I criticize myself for having irrational or inappropriate emotions.	☐	☐	☐	☐	☐	☐	☐
FF1_04. I perceive my feelings and emotions without having to react to them.	☐	☐	☐	☐	☐	☐	☐
FF1_05. When I do things, my mind wanders off and I'm easily distracted.	☐	☐	☐	☐	☐	☐	☐
FF1_06. When I take a shower or bath, I stay alert to the sensations of water on my body.	☐	☐	☐	☐	☐	☐	☐
FF1_07. I can easily put my beliefs, opinions, and expectations into words.	☐	☐	☐	☐	☐	☐	☐
FF1_08. I don't pay attention to what I'm doing because I'm daydreaming, worrying, or otherwise distracted.	☐	☐	☐	☐	☐	☐	☐
FF1_09. I watch my feelings without getting lost in them.	☐	☐	☐	☐	☐	☐	☐
FF1_10. I tell myself I shouldn't be feeling the way I'm feeling.	☐	☐	☐	☐	☐	☐	☐
FF1_11. I notice how foods and drinks affect my thoughts, bodily sensations, and emotions.	☐	☐	☐	☐	☐	☐	☐
FF1_12. It's hard for me to find the words to describe what I'm thinking.	☐	☐	☐	☐	☐	☐	☐
FF1_13. I am easily distracted.	☐	☐	☐	☐	☐	☐	☐
FF1_14. I believe some of my thoughts are abnormal or bad and I shouldn't think that way.	☐	☐	☐	☐	☐	☐	☐
FF1_15. I pay attention to sensations, such as the wind in my hair or sun on my face.	☐	☐	☐	☐	☐	☐	☐

	Never	Almost Never	Sometimes	Fairly Often	Often	I do not know	I prefer not to answer
FF1_16. I have trouble thinking of the right words to express how I feel about things.	☐	☐	☐	☐	☐	☐	☐
FF1_17. I make judgments about whether my thoughts are good or bad.	☐	☐	☐	☐	☐	☐	☐
FF1_18. I find it difficult to stay focused on what's happening in the present.	☐	☐	☐	☐	☐	☐	☐
FF1_19. When I have distressing thoughts or images, I "step back" and am aware of the thought or image without getting taken over by it.	☐	☐	☐	☐	☐	☐	☐
FF1_20. I pay attention to sounds, such as clocks ticking, birds chirping, or cars passing.	☐	☐	☐	☐	☐	☐	☐
FF1_21. In difficult situations, I can pause without immediately reacting.	☐	☐	☐	☐	☐	☐	☐
FF1_22. When I have a sensation in my body, it's difficult for me to describe it because I can't find the right words.	☐	☐	☐	☐	☐	☐	☐
FF1_23. It seems I am "running on automatic" without much aware- ness of what I'm doing.	☐	☐	☐	☐	☐	☐	☐
FF1_24. When I have distressing thoughts or images, I feel calm soon after.	☐	☐	☐	☐	☐	☐	☐
FF1_25. I tell myself that I shouldn't be thinking the way I'm thinking.	☐	☐	☐	☐	☐	☐	☐
FF1_26. I notice the smells and aromas of things.	☐	☐	☐	☐	☐	☐	☐
FF1_27. Even when I'm feeling terribly upset, I can find a way to put it into words.	☐	☐	☐	☐	☐	☐	☐
FF1_28. I rush through activities without being really attentive to them.	☐	☐	☐	☐	☐	☐	☐

	Never	Almost Never	Sometimes	Fairly Often	Often	I do not know	I prefer not to answer
FF1_29. When I have distressing thoughts or images, I am able just to notice them without reacting.	☐	☐	☐	☐	☐	☐	☐
FF1_30. I think some of my emotions are bad or inappropriate and I shouldn't feel them.	☐	☐	☐	☐	☐	☐	☐
FF1_31. I notice visual elements in art or nature, such as colors, shapes, textures, or patterns of light and shadow.	☐	☐	☐	☐	☐	☐	☐
FF1_32. My natural tendency is to put my experiences into words.	☐	☐	☐	☐	☐	☐	☐
FF1_33. When I have distressing thoughts or images, I just notice them and let them go.	☐	☐	☐	☐	☐	☐	☐
FF1_34. I do jobs or tasks automatically without being aware of what I'm doing.	☐	☐	☐	☐	☐	☐	☐
FF1_35. When I have distressing thoughts or images, I judge myself as good or bad depending what the thought or image is about.	☐	☐	☐	☐	☐	☐	☐
FF1_36. I pay attention to how my emotions affect my thoughts and behavior.	☐	☐	☐	☐	☐	☐	☐
FF1_37. I can usually describe how I feel at the moment in consider- able detail.	☐	☐	☐	☐	☐	☐	☐
FF1_38. I find myself doing things without paying attention.	☐	☐	☐	☐	☐	☐	☐
FF1_39. I disapprove of myself when I have irrational ideas.	☐	☐	☐	☐	☐	☐	☐

Parent's Education

CS1_01. Please check the box beside the highest grade or degree that your BIOLOGICAL MOTHER completed.

Never went to school	☐
Grades 1 to 3	☐
Grades 4 to 8	☐
Grades 9 to 11	☐
Grade 12	☐
GED	☐
One or more years of Vocational or Professional School after High School	☐
One or more years of College	☐
One or more years of Graduate or Professional School after College	☐
I Do Not Know	☐
I prefer not to answer	☐

CS1_02. Please check the box beside the highest grade or degree that your BIOLOGICAL FATHER completed.

Never went to school	☐
Grades 1 to 3	☐
Grades 4 to 8	☐
Grades 9 to 11	☐
Grade 12	☐
GED	☐
One or more years of Vocational or Professional School after High School	☐
One or more years of College	☐
One or more years of Graduate or Professional School after College	☐
I Do Not Know	☐
I prefer not to answer	☐

Now please think of the two most important adults in your home between the time you were born and age 18 years. Please check the category below that best described their level of education during this time period.

Deleted: lecel

CS1_03. First adult's highest level of education:

Never went to school	☐
Grades 1 to 3	☐
Grades 4 to 8	☐
Grades 9 to 11	☐
Grade 12	☐
GED	☐
One or more years of Vocational or Professional School after High School	☐
One or more years of College	☐
One or more years of Graduate or Professional School after College	☐
I Do Not Know	☐
I prefer not to answer	☐

CS1_04. Second adult's highest level of education

Never went to school	☐
Grades 1 to 3	☐
Grades 4 to 8	☐
Grades 9 to 11	☐
Grade 12	☐
GED	☐
One or more years of Vocational or Professional School after High School	☐
One or more years of College	☐
One or more years of Graduate or Professional School after College	☐
I Do Not Know	☐
I prefer not to answer	☐

Your Childhood Experiences

The following questions ask about some difficult experiences that you might have had as a child. These questions may be emotionally difficult to answer. Just as a reminder, you do not need answer any questions that you would prefer not to. Your answers to these questions, as with all questions, will remain confidential.

CE1_01. Before you were 18 years old, did a parent or other adult in the household often or very often…

Swear at you, insult you, put you down, or humiliate you?
or
Act in a way that made you afraid that you might be physically hurt?

□ No
□ Yes
□ I do not know
□ I prefer not to answer

CE1_02. Before you were 18 years old, did a parent or other adult in the household often or very often…

Push, grab, slap, or throw something at you?
or
Ever hit you so hard that you had marks or were injured?

□ No
□ Yes
□ I do not know
□ I prefer not to answer

CE1_03. Before you were 18 years old, did an adult or person at least 5 years older than you ever…

Touch or fondle you or have you touch their body in a sexual way?
or
Attempt or actually have oral, anal, or vaginal intercourse with you?

□ No
□ Yes
□ I do not know
□ I prefer not to answer

CE1_04. Before you were 18 years old, did you often or very often feel that …

No one in your family loved you or thought you were important or special?
or
Your family didn't look out for each other, feel close to each other, or support each other?

- □ No
- □ Yes
- □ I do not know
- □ I prefer not to answer

CE1_05. Before you were 18 years old, did you often or very often feel that …

You didn't have enough to eat, had to wear dirty clothes, and had no one to protect you?
or
Your parents were too drunk or high to take care of you or take you to the doctor if you needed it?

- □ No
- □ Yes
- □ I do not know
- □ I prefer not to answer

CE1_06. Before you were 18 years old, was a biological parent ever lost to you through divorce, abandonment, or other reason?

- □ No
- □ Yes
- □ I do not know
- □ I prefer not to answer

CE1_07. Before you were 18 years old, was your mother or stepmother:

Often or very often pushed, grabbed, slapped, or had something thrown at her?
or
Sometimes, often, or very often kicked, bitten, hit with a fist, or hit with something hard?
or
Ever repeatedly hit over at least a few minutes or threatened with a gun or knife?

- □ No
- □ Yes
- □ I do not know
- □ I prefer not to answer

CE1_08. Before you were 18 years old, did you live with anyone who was a problem drinker or alcoholic, or who used street drugs?

- □ No
- □ Yes
- □ I do not know
- □ I prefer not to answer

CE1_09. Before you were 18 years old, was a household member depressed or mentally ill, or did a household member attempt suicide?

- □ No
- □ Yes
- □ I do not know
- □ I prefer not to answer

CE1_10. Before you were 18 years old, did a household member go to prison?

- □ No
- □ Yes
- □ I do not know
- □ I prefer not to answer

Deleted: SN1_01. How many close friends do you have? By close friends, we mean people that you feel at ease with, and that you can talk to about private matters.¶

□ None → ***skip to Y2*** ¶

____ Number of close friends ¶

□ I do not know ¶

□ I prefer not to answer ¶

SN1_01a. How many of these close friends do you see at least once a month? ¶

____ Number of close friends ***(if none, write zero)***¶

□ I do not know ¶

□ I prefer not to answer ¶

SN1_02. Thinking about your relatives, how many relatives do you feel at ease with, and feel that you can talk to about private matters? ¶

□ None → ***skip to Y3***¶

____ Number of relatives ¶

□ I do not know ¶

□ I prefer not to answer ¶

SN1_02a. How many of these relatives do you see at least once a month? ¶

____ Number of relatives ***(if none, write zero)***¶

□ I do not know ¶

□ I prefer not to answer ¶

SN1_03. About how often do you participate in groups or clubs, such as religious connected groups, self-help groups, charities, or a public service or community group.¶

□ Never or almost never¶
□ A few times a year¶
□ Once or twice a month¶
□ Once a week¶
□ More than once a week¶
□ I do not know ¶
□ I prefer not to answer ¶

SN1_04. About how often do you go to religious meetings or services? ¶

□ Never or almost never¶
□ A few times a year¶
□ Once or twice a month¶
□ Once a week¶
□ More than once a week¶

... [2]

Blood Pressure Medication Use

Comment [SF5]: New Section

BM1_01. Do you currently take medication for your blood pressure?
□ Yes
□ No (if responding "no", please skip to the next page).

	Yes	No
BM1_02. Do you sometimes forget to take your blood pressure pills?	□	□
BM1_03. People sometimes miss taking their medications for reasons other than forgetting. Thinking over the past two weeks, were there any days when you did not take your blood pressure medicine?	□	□
BM1_04. Have you ever cut back or stopped taking your blood pressure medicine without telling your doctor because you felt worse when you took it?	□	□
BM1_05. When you travel or leave home, do you sometimes forget to bring along your blood pressure medicine?	□	□
BM1_06. Did you take all your blood pressure medicine yesterday?	□	□
BM1_07. When you feel like your symptoms are under control, do you sometimes stop taking your blood pressure medicine?	□	□
BM1_08. Taking blood pressure medicine every day is a real inconvenience for some people. Do you ever feel hassled about sticking to your treatment plan?	□	□

BM1_09. How often do you have difficulty remembering to take all your blood pressure medicine?
□ Never/rarely
□ Once in a while
□ Sometimes
□ Usually
□ All the time

More About You

Comment [SF6]: New Section – readiness to change

Below are some modifiable factors that likely influence blood pressure. These may not all apply to you, as you may already have excellent levels of these factors.

Physical activity:
The United States Office of Disease Prevention and Health Promotion 2008 Physical Activity Guidelines for Americans states that "Most health benefits occur with at least 150 minutes (2 hours and 30 minutes) a week of moderate intensity physical activity, such as brisk walking. Additional benefits occur with more physical activity. Both aerobic (endurance) and muscle-strengthening (resistance) physical activity are beneficial."

RC1_01. How motivated are you to make changes to your physical activity, using a scale of one to ten, where one = definitely not ready to change, and 10 = definitely ready to change?

Little intention of changing			Mixed feelings towards taking action				Motivated to take action		
1	2	3	4	5	6	7	8	9	10
☐	☐	☐	☐	☐	☐	☐	☐	☐	☐

RC1_02. On a scale of 1-10, with 10 being 100% confident, how confident are you that you can make changes in your physical activity?

Not confident			Moderately confident				Confident		
1	2	3	4	5	6	7	8	9	10
☐	☐	☐	☐	☐	☐	☐	☐	☐	☐

Diet:

The Dietary Approaches to Stop Hypertension (DASH) diet eating plan is a diet rich in fruits, vegetables, low fat or nonfat dairy. It also includes mostly whole grains; lean meats, fish and poultry; nuts and beans. It is high fiber and low to moderate in fat. It is a plan that follows US guidelines for sodium content, along with vitamins and minerals. It can be considered to be an Americanized version of the Mediterranean diet.

RC1_03. How motivated are you to make changes to your diet to be consistent with the DASH diet, using a scale of one to ten, where one = definitely not ready to change, and 10 = definitely ready to change?

Little intention of changing			Mixed feelings towards taking action				Motivated to take action		
1	2	3	4	5	6	7	8	9	10
☐	☐	☐	☐	☐	☐	☐	☐	☐	☐

RC1_04. On a scale of 1-10, with 10 being 100% confident, how confident are you that you can make changes in your diet to be more consistent with the DASH diet?

Not confident			Moderately confident				Confident		
1	2	3	4	5	6	7	8	9	10
☐	☐	☐	☐	☐	☐	☐	☐	☐	☐

Salt Intake:

The 2010 Dietary Guidelines for Americans recommend that everyone age 2 years and up should consume less than 2,300 milligrams (mg) of sodium each day. Some groups of people should further limit sodium intake to 1,500 mg per day, including:

- Adults age 51 years or older.
- All African Americans.
- Anyone who has high blood pressure, diabetes, or chronic kidney disease.

RC1_05. How motivated are you to make changes to your salt intake, using a scale of one to ten, where one = definitely not ready to change, and 10 = definitely ready to change?

Little intention of changing			Mixed feelings towards taking action				Motivated to take action		
1	2	3	4	5	6	7	8	9	10
□	□	□	□	□	□	□	□	□	□

RC1_06. On a scale of 1-10, with 10 being 100% confident, how confident are you that you can make changes in your salt intake?

Not confident			Moderately confident				Confident		
1	2	3	4	5	6	7	8	9	10
□	□	□	□	□	□	□	□	□	□

Overweight/Obesity:

Extensive scientific evidence shows that being overweight or obese increases risk of having high blood pressure.

RC1_07. How motivated are you to make changes to your body weight, using a scale of one to ten, where one = definitely not ready to change, and 10 = definitely ready to change?

Little intention of changing			Mixed feelings towards taking action				Motivated to take action		
1	2	3	4	5	6	7	8	9	10
□	□	□	□	□	□	□	□	□	□

RC1_08. On a scale of 1-10, with 10 being 100% confident, how confident are you that you can make changes in your body weight?

Not confident			Moderately confident				Confident		
1	2	3	4	5	6	7	8	9	10
□	□	□	□	□	□	□	□	□	□

Stress, and Stress Response:

Several studies showed that stress, and being slower at emotionally recovering from stressful events, increase risk of hypertension.

RC1_09. How motivated are you to make changes to the amount of stress in your life, or your response to that stress, using a scale of one to ten, where one = definitely not ready to change, and 10 = definitely ready to change?

Little intention of changing			Mixed feelings towards taking action				Motivated to take action		
1	2	3	4	5	6	7	8	9	10
□	□	□	□	□	□	□	□	□	□

RC1_10. On a scale of 1-10, with 10 being 100% confident, how confident are you that you can make changes in the amount of stress in your life, or your response to that stress?

Not confident			Moderately confident				Confident		
1	2	3	4	5	6	7	8	9	10
□	□	□	□	□	□	□	□	□	□

Alcohol Consumption:

Heavy and regular use of alcohol can increase blood pressure substantially. The American Heart Association recommends limiting alcohol consumption to no more than two drinks per day for men and one drink per day for women.

RC1_11. How motivated are you to make changes to the amount of alcohol you consume, using a scale of one to ten, where one = definitely not ready to change, and 10 = definitely ready to change?

Little intention of changing			Mixed feelings towards taking action				Motivated to take action		
1	2	3	4	5	6	7	8	9	10
□	□	□	□	□	□	□	□	□	□

RC1_12. On a scale of 1-10, with 10 being 100% confident, how confident are you that you can make changes in the amount of alcohol you consume?

Not confident			Moderately confident				Confident		
1	2	3	4	5	6	7	8	9	10
□	□	□	□	□	□	□	□	□	□

Blood Pressure (Antihypertensive) Medication Use:

Blood pressure medication has been shown in many studies to be very effective at lowering blood pressure.

RC1_13. How motivated are you to make changes to your blood pressure medication use, using a scale of one to ten, where one = definitely not ready to change, and 10 = definitely ready to change?

Little intention of changing			Mixed feelings towards taking action				Motivated to take action		
1	2	3	4	5	6	7	8	9	10
□	□	□	□	□	□	□	□	□	□

RC1_14. On a scale of 1-10, with 10 being 100% confident, how confident are you that you can make changes your blood pressure medication use?

Not confident			Moderately confident				Confident		
1	2	3	4	5	6	7	8	9	10
□	□	□	□	□	□	□	□	□	□

Family History of Hypertension

Comment [SF7]: New section

FH1_01. Did your biological mother ever have hypertension?

□ No
□ Yes
□ I do not know
□ I prefer not to answer

FH1_02. Did your biological father ever have hypertension?

□ No
□ Yes
□ I do not know
□ I prefer not to answer

FH1_03. How many full brothers and sisters do you have ***(Please include any brothers or sisters who may have died, but do not include half or step brothers and sisters).***

□ I do not have any brothers or sisters → S***kip to the page***
________ brothers, ________ sisters
□ I do not know
□ I prefer not to answer

FH1_04. Of these brothers and sisters, how many have ever had hypertension?

________ ***(if none, write 0)***
□ I do not know
□ I prefer not to answer

Your Sleep

The following question relates to your usual sleep habits during the past month only. Your answer should indicate the most accurate reply for the majority of days and nights in the past month.

SL1_04. **During the past month**, how many hours of actual sleep did you get on average at night? (This may be different than the number of hours you spent in bed.)

AVERAGE HOURS OF SL1_EEP PER NIGHT ___________

□ I do not know
□ I prefer not to answer

END SCRIPT

Thank you for completing this survey!

Please note that these responses will not be seen immediately. Resources are shown below if you feel that you would like to talk with someone immediately for assistance.

National Suicide Prevention Lifeline: 1-800-273-8255
National Sexual Assault Hotline: 1-800-656-4673

Other options are to:

- Call your doctor's office
- Call 911 for emergency services
- Go to the nearest hospital emergency room.

Comment [D8]: This section (Sleep) was moved to the end of the survey. Several of the items were dropped to reduce participant burden.

Deleted: ***For each of the remaining questions, check the one best response. Please answer all questions.***¶
¶
During the past month, how often have you had trouble sleeping because you…………..¶
¶
SL1_05. …..Could not get to sleep within 30 minutes?¶
□ Not during the past month¶
□ Less than once a week ¶
□ Once or twice a week¶
□ Three or more times a week ¶
□ I do not know¶
□ I prefer not to answer¶
¶
¶
SL1_06. …..Woke up in the middle of the night or early morning?¶
□ Not during the past month¶
□ Less than once a week ¶
□ Once or twice a week¶
□ Three or more times a week ¶
□ I do not know¶
□ I prefer not to answer ¶
¶
¶
SL1_07. …..Had to get up to use the bathroom?¶
□ Not during the past month¶
□ Less than once a week ¶
□ Once or twice a week¶
□ Three or more times a week ¶
□ I do not know¶
□ I prefer not to answer ¶
¶
¶
¶
SL1_08. …..Could not breathe comfortably?¶
□ Not during the past month¶
□ Less than once a week ¶
□ Once or twice a week¶
□ Three or more times a week ¶
□ I do not know¶
□ I prefer not to answer ¶
... [3]

Deleted: SL1_16. **During the past month**, how often have you taken medicine to help you sleep (prescribed or "over the counter")?¶
¶
□ Not during the past month¶
□ Less than once a week ¶
□ Once or twice a week¶
□ Three or more times a week ¶
□ I do not know¶
□ I prefer not to answer ¶
¶
SL1_17. **During the past month**, how often have you had trouble staying awake while driving, eating meals, or engaging in social activity?¶
¶
□ Not during the past month¶
□ Less than once a week ¶
□ Once or twice a week¶
□ Three or more times a week ¶
□ I do not know¶
□ I prefer not to answer ¶
¶
... [4]

Page 9: [1] Deleted	Saadeh, Frances	10/28/2015 10:57:00 AM

How Often You Eat or Drink This Item

	Never or less than 1 per month	1-3 per month	1 per week	2-4 per week	5-6 per week	1 per day	2-3 per day	4-5 per day	More than 5 per day	I do not know	I prefer not to answer
**DT1_01**. Skim/low fat milk (8 oz. glass)	☐	☐	☐	☐	☐	☐	☐	☐	☐	☐	☐
**DT1_02.** Whole milk (8 oz. glass)	☐	☐	☐	☐	☐	☐	☐	☐	☐	☐	☐
**DT1_03.** Ice cream (1 cup)	☐	☐	☐	☐	☐	☐	☐	☐	☐	☐	☐
**DT1_04.** Yogurt (1 cup)	☐	☐	☐	☐	☐	☐	☐	☐	☐	☐	☐
**DT1_05.** Cottage or ricotta cheese (1/2 cup)	☐	☐	☐	☐	☐	☐	☐	☐	☐	☐	☐
**DT1_06.** Butter, added to food or bread (1 pat), exclude use in cooking	☐	☐	☐	☐	☐	☐	☐	☐	☐	☐	☐
**DT1_07.** Fruit (not including fruit juice. A serving = 1 piece, or 1/2 grapefruit, or 1/2 cup berries)	☐	☐	☐	☐	☐	☐	☐	☐	☐	☐	☐
**DT1_08.** 100% fruit juice, not including fruit drinks (small glass)	☐	☐	☐	☐	☐	☐	☐	☐	☐	☐	☐

How Often You Eat or Drink This Item

	Never or less than 1 per month	1-3 per month	1 per week	2-4 per week	5-6 per week	1 per day	2-3 per day	4-5 per day	More than 5 per day	I do not know	I prefer not to answer
**DT1_09.** Vegetables - raw and cooked (1/2 cup), including mixed dishes such as soups, casseroles and lasagna. Do not include potatoes.	☐	☐	☐	☐	☐	☐	☐	☐	☐	☐	☐
**DT1_10.** Eggs (do not include egg beaters) (1)	☐	☐	☐	☐	☐	☐	☐	☐	☐	☐	☐
**DT1_11.** Chicken or turkey (4-6 oz.)	☐	☐	☐	☐	☐	☐	☐	☐	☐	☐	☐
**DT1_12.** Processed meat (1 piece sausage, 1 slice salami, 2 pieces bacon)	☐	☐	☐	☐	☐	☐	☐	☐	☐	☐	☐
**DT1_13.** Hot dog/ hamburger	☐	☐	☐	☐	☐	☐	☐	☐	☐	☐	☐

How Often You Eat or Drink This Item

	Never or less than 1 per month	1-3 per month	1 per week	2-4 per week	5-6 per week	1 per day	2-3 per day	4-5 per day	More than 5 per day	I do not know	I prefer not to answer
**DT1_14**. Beef, pork, or lamb as a sandwich or mixed dish, e.g. stew, casserole, lasagna, etc.	☐	☐	☐	☐	☐	☐	☐	☐	☐	☐	☐
**DT1_15**. Beef, pork, or lamb as a main dish, e.g. steak, roast, ham, etc. (4-6 oz.)	☐	☐	☐	☐	☐	☐	☐	☐	☐	☐	☐
**DT1_16**. Fish (including canned tuna) (3-5 oz.)	☐	☐	☐	☐	☐	☐	☐	☐	☐	☐	☐
**DT1_17**. Shellfish such as shrimp, lobster, scallops and clams as a main dish	☐	☐	☐	☐	☐	☐	☐	☐	☐	☐	☐
**DT1_18**. White bread (including pita bread) (slice)	☐	☐	☐	☐	☐	☐	☐	☐	☐	☐	☐
**DT1_19.** Dark bread (including whole wheat and rye) (slice)	☐	☐	☐	☐	☐	☐	☐	☐	☐	☐	☐
**DT1_20.** Bakery items (cookies, brownies, doughnuts, cake) (1 piece or slice)	☐	☐	☐	☐	☐	☐	☐	☐	☐	☐	☐
**DT1_21.** Nuts (small packet, 1 oz., 1 Tbs peanut butter)	☐	☐	☐	☐	☐	☐	☐	☐	☐	☐	☐
**DT1_22.** Pasta (e.g. spaghetti, and macaroni and cheese; 1 cup cooked)	☐	☐	☐	☐	☐	☐	☐	☐	☐	☐	☐
**DT1_23.** Potatoes, baked, boiled or mashed (do not include French fries/ chips) (1 potato or 1 cup)	☐	☐	☐	☐	☐	☐	☐	☐	☐	☐	☐

How Often You Eat or Drink This Item

	Never or less than 1 per month	1-3 per month	1 per week	2-4 per week	5-6 per week	1 per day	2-3 per day	4-5 per day	More than 5 per day	I do not know	I prefer not to answer
**DT1_24**. Regular soft drinks (including caffeine free, but NOT including diet colas) (consider the serving size as 1 glass, bottle or can)	☐	☐	☐	☐	☐	☐	☐	☐	☐	☐	☐
**DT1_25**. Coffee (regular or decaf) (1 cup)	☐	☐	☐	☐	☐	☐	☐	☐	☐	☐	☐

Page 41: [2] Deleted **Saadeh, Frances** **10/28/2015 11:57:00 AM**

SN1_01. How many close friends do you have? By close friends, we mean people that you feel at ease with, and that you can talk to about private matters.

☐ None → ***skip to Y2***

____ Number of close friends

☐ I do not know
☐ I prefer not to answer

SN1_01a. How many of these close friends do you see at least once a month?

____ Number of close friends ***(if none, write zero)***

☐ I do not know
☐ I prefer not to answer

SN1_02. Thinking about your relatives, how many relatives do you feel at ease with, and feel that you can talk to about private matters?

☐ None → ***skip to Y3***

____ Number of relatives

☐ I do not know
☐ I prefer not to answer

SN1_02a. How many of these relatives do you see at least once a month?

____ Number of relatives ***(if none, write zero)***

☐ I do not know
☐ I prefer not to answer

SN1_03. About how often do you participate in groups or clubs, such as religious connected groups, self-help groups, charities, or a public service or community group.

☐ Never or almost never
☐ A few times a year
☐ Once or twice a month
☐ Once a week
☐ More than once a week
☐ I do not know
☐ I prefer not to answer

SN1_04. About how often do you go to religious meetings or services?

☐ Never or almost never
☐ A few times a year
☐ Once or twice a month
☐ Once a week
☐ More than once a week
☐ I do not know
☐ I prefer not to answer

Page 48: [3] Deleted	Eric Loucks	11/2/2015 10:42:00 AM

For each of the remaining questions, check the one best response. Please answer all questions.

During the past month, how often have you had trouble sleeping because you…………..

SL1_05. …..Could not get to sleep within 30 minutes?
☐ Not during the past month

☐ Less than once a week
☐ Once or twice a week
☐ Three or more times a week
☐ I do not know
☐ I prefer not to answer

SL1_06. …..Woke up in the middle of the night or early morning?
☐ Not during the past month
☐ Less than once a week
☐ Once or twice a week
☐ Three or more times a week
☐ I do not know
☐ I prefer not to answer

SL1_07. …..Had to get up to use the bathroom?
☐ Not during the past month
☐ Less than once a week
☐ Once or twice a week
☐ Three or more times a week
☐ I do not know
☐ I prefer not to answer

SL1_08. …..Could not breathe comfortably?
☐ Not during the past month
☐ Less than once a week
☐ Once or twice a week
☐ Three or more times a week
☐ I do not know
☐ I prefer not to answer

SL1_09. …..Coughed or snored loudly?

☐ Not during the past month
☐ Less than once a week
☐ Once or twice a week
☐ Three or more times a week
☐ I do not know

☐ I prefer not to answer

SL1_10. …..Felt too cold?

☐ Not during the past month
☐ Less than once a week
☐ Once or twice a week
☐ Three or more times a week
☐ I do not know
☐ I prefer not to answer

SL1_11. …..Felt too hot?

☐ Not during the past month
☐ Less than once a week
☐ Once or twice a week
☐ Three or more times a week
☐ I do not know
☐ I prefer not to answer

SL1_12. …..Had bad dreams?

☐ Not during the past month
☐ Less than once a week
☐ Once or twice a week
☐ Three or more times a week
☐ I do not know
☐ I prefer not to answer

SL1_13. …..Had pain?

☐ Not during the past month
☐ Less than once a week
☐ Once or twice a week
☐ Three or more times a week
☐ I do not know
☐ I prefer not to answer

SL1_14a. Other reason(s), **please describe:** ____

SL1_14b. How often during the past month have you had trouble sleeping because of this?

☐ Not during the past month
☐ Less than once a week
☐ Once or twice a week
☐ Three or more times a week
☐ I do not know
☐ I prefer not to answer

**Page 48: [4] Deleted**	**Eric Loucks**	**11/2/2015 10:43:00 AM**

SL1_16. **During the past month**, how often have you taken medicine to help you sleep (prescribed or "over the counter")?

☐ Not during the past month
☐ Less than once a week
☐ Once or twice a week
☐ Three or more times a week
☐ I do not know
☐ I prefer not to answer

SL1_17. **During the past month**, how often have you had trouble staying awake while driving, eating meals, or engaging in social activity?

☐ Not during the past month
☐ Less than once a week
☐ Once or twice a week
☐ Three or more times a week
☐ I do not know
☐ I prefer not to answer

SL1_18. **During the past month**, how much of a problem has it been for you to keep up enough enthusiasm to get things done?

☐ No problem at all
☐ Somewhat of a problem
☐ A very big problem
☐ I do not know
☐ I prefer not to answer

SL1_19. Do you have a bed partner or a room-mate in the same room ?

- ☐ No bed partner or roommate in the same room → ***skip to next survey section:***
- ☐ Partner in same bed
- ☐ Room-mate in the same room
- ☐ I do not know
- ☐ I prefer not to answer

If you have a roommate or bed partner, <u>ask</u> him/her how often in the past month <u>you</u> have had . . . .

SL1_20. …..Loud snoring

- ☐ Not during the past month
- ☐ Less than once a week
- ☐ Once or twice a week
- ☐ Three or more times a week
- ☐ Unable to ask him/her
- ☐ he/she does not know
- ☐ he/she prefers not to answer

SL1_21. ……Long pauses between breaths while asleep

- ☐ Not during the past month
- ☐ Less than once a week
- ☐ Once or twice a week
- ☐ Three or more times a week
- ☐ Unable to ask him/her
- ☐ he/she does not know
- ☐ he/she prefers not to answer

SL1_22. …..Legs twitching or jerking while you sleep

- ☐ Not during the past month
- ☐ Less than once a week
- ☐ Once or twice a week
- ☐ Three or more times a week
- ☐ Unable to ask him/her
- ☐ he/she does not know
- ☐ he/she prefers not to answer

SL1_23. …..Episodes of disorientation or confusion during sleep

□ Not during the past month
□ Less than once a week
□ Once or twice a week
□ Three or more times a week
□ Unable to ask him/her
□ he/she does not know
□ he/she prefers not to answer

SL1_24a. ……Other restlessness while you sleep; ***please describe:***

____

SL1_24b. □ Not during the past month
□ Less than once a week
□ Once or twice a week
□ Three or more times a week
□ Unable to ask him/her
□ he/she does not know
□ he/she prefers not to answer

Brown University
Research Protections Office
Institutional Review Board
Modification Request

Date of Request: 2/26/16 **Investigator's Name and Title:** Eric Loucks, PhD, Assistant Professor

Study Title: Mindfulness-based Hyptertension Therapy Pilot Study (#1412001171)

Original Type of Review: ☐ Exempt ☐ Expedited ☒ Full Board

1) **Provide a brief lay summary of the overall project. Include enough detail to allow the IRB to evaluate the requested change(s) within the context of the overall project.** (Attach summary to this form)

See attached summary

2) **Provide a detailed description of the changes being requested** (Use additional pages, if necessary):

We are requesting permission to allow study participants the option to participate in the MBHT Intervention (i.e., 9-week MBHT course) via online video conferencing tool (e.g., Skype, Zoom, etc.) in situations when they are not able to attend class in-person.

3.) **State the reason (justification) for the requested modification.** (Use additional pages, if necessary):

We would like to be able to allow participants, who are not able to physically come to the study site on a given day (a maximum of 2 classes), the option to participate in the study intervention via online video conferencing tool (e.g., Skype, Zoom, etc.). None of the video footage would be recorded and participation via video conferencing would be optional. Video conferencing would only take place with video software that establishes unique connections, such as via Skype names, or Zoom conference call ID numbers. This disallows anyone to join the call other than those who are specifically invited by the study staff. Study participants who choose to decline this option would still be allowed to continue with the broader study.

4.) **What is your assessment of how the changes will affect the overall risk/benefit ratio of the study and the willingness of individuals to participate?**

We do not anticipate this amendendment will greatly affect the overall risk/benefti ratio of the study. If anything, it will allow participants additional flexibility in how they participate and will potentially reduce the number of classes missed, thus allowing participants, who otherwise might have missed classes, to get more out of the course.

5.) **Does the requested modification require new documents or changes to the approved consent form or other documents?**

☐ Consent/assent documents (attach revised version with changes highlighted)

☐ New/revised instruments (attach - if revised, highlight changes)

☐ New/revised advertising materials (attach - if revised, highlight changes)

Do you have a conflict of interest on this project according to Brown's policy? ☐**YES** ☒**NO**

If YES, has this conflict been previously disclosed to the IRB? ☐**YES** ☐**NO**

PI signature: [signature] Date: 2/26/16

Date of IRB Review: ___________ **IRB**

Determination: ______________________________

Signature of IRB Chair or Designate ____________________________

1. Provide a brief lay summary of the overall project. Include enough detail to allow the IRB to evaluate the requested change(s) within the context of the overall project.

The World Health Organization reported that suboptimal blood pressure (BP) is responsible for more than half of cardiovascular disease mortality world-wide. Furthermore, greater than half of those with hypertension have uncontrolled BP. *A 2009 Institute of Medicine report recommended prioritizing research to "Compare the effectiveness of mindfulness-based interventions (e.g. yoga, meditation, deep breathing training) and usual care in treating… cardiovascular risk factors."* Evidence-based mindfulness interventions, including Mindfulness-Based Stress Reduction, may have some effects on blood pressure, where a recent meta-analysis and systematic review of 4 randomized controlled trials demonstrated significant effects, but evidence of heterogeneity in effect sizes. The methodologically highest quality studies had the smallest effect sizes (range 0-5 mmHg). Mindfulness-Based Stress Reduction (MBSR) has been customized to a number of disease processes, such as Mindfulness-Based Cognitive Therapy for patients with recurrent depression, and Mindfulness-Based Relapse Prevention for patients with substance use addictions. Effect sizes have been increased by customizing mindfulness interventions to diseases of interest. The same may be true for hypertension, *however mindfulness interventions customized for prehypertensive/hypertensive patients have never been investigated. Until methodologically rigorous studies to evaluate customized interventions for hypertension are performed, we will not know if the observed preliminary effects of general mindfulness interventions on blood pressure reduction could be much more effective with a tailored approach.* Consequently, we propose to conduct a stage I behavioral therapy intervention study to evaluate whether MBSR customized to prehypertensive and hypertensive patients has the potential to provide clinically relevant reductions in BP. Consequently the specific aims are:

Stage 1a: Therapy Development/Manual Writing

1. To outline and evaluate key novel elements of mindfulness-based hypertension therapy (MBHT), customized from the evidence-based MBSR. *We hypothesize that the most important novel element will be generation of mindfulness skills specifically applied to hypertension risk factors such as diet, physical activity, obesity, alcohol consumption and antihypertensive medication adherence.* This aim will be achieved using (1) focus groups of participants undergoing the MBHT behavioral intervention, (2) discussion with experts (including cardiologists, epidemiologists, mindfulness experts, mindfulness intervention instructors) prior to, and following pilot testing of MBHT in participants, and (3) clinical judgment of the investigators performing the intervention.
2. To determine effectiveness of MBHT on primary outcomes (systolic blood pressure, retention rates, recruitment rates, and adverse effects) and secondary outcomes (hypertension risk factors such as diet, physical activity, obesity, and antihypertensive medication adherence) in hypertension subgroups, specifically participants with (1) prehypertension, (2) controlled hypertension, and (3) uncontrolled hypertension. Initial decisions about the targeted sample based on hypertension status will be made.
3. To develop an MBHT therapist manual and training program, including procedures for training, supervising, and evaluating therapists. Furthermore, acceptable therapist characteristics will be developed. The manual and training program will include themes such as specification of unique and common elements of MBHT *vs.* other interventions, description of interventions excluded from MBHT, and specification of key treatment parameters such as frequency and duration of treatment, session length, topics addressed, sequence of sessions, as well as therapist adherence and competency

measures. The MBHT training will consist of a therapist manual, a formal didactic training seminar, and at least one closely supervised training session.

Stage Ib: Pilot Trial

4. To determine whether a mindfulness-based hypertension therapy (MBHT) intervention, customized from the evidence-based MBSR, has promise to be an effective behavioral therapy for participants with hypertension and/or prehypertension. We will perform a randomized controlled pilot trial for MBHT *vs.* enhanced usual care control. *We hypothesize that MBHT will have adequate recruitment rates (≥10% of prehypertensive/hypertensive participants invited from physicians' offices), fairly low drop out rates (<15%), and medium effect sizes (e.g. 5-10 mmHg systolic BP) for reduction in blood pressure.*

These findings will provide publishable pilot data that will inform future randomized clinical trials that evaluate effects of MBHT on long-term changes in blood pressure *vs.* usual care and active control groups. *If proven effective, MBHT could be offered as a complementary program in the prehypertensive/hypertensive patient population that contributes to over half of the cardiovascular disease mortality world-wide.*

Brown University
Research Protections Office
Institutional Review Board
Modification Request

Date of Request: 4/25/16 **Investigator's Name and Title:** Eric Loucks, PhD, Assistant Professor

Study Title: Mindfulness-based Hypertension Therapy Pilot Study (#1412001171)

Original Type of Review: ☐ Exempt ☐ Expedited ☒ Full Board

1) **Provide a brief lay summary of the overall project. Include enough detail to allow the IRB to evaluate the requested change(s) within the context of the overall project.** (Attach summary to this form)

See attached summary

2) **Provide a detailed description of the changes being requested** (Use additional pages, if necessary):

See attached summary

3.) **State the reason (justification) for the requested modification.** (Use additional pages, if necessary):

See attached summary

4.) **What is your assessment of how the changes will affect the overall risk/benefit ratio of the study and the willingness of individuals to participate?**

See attached summary

5.) **Does the requested modification require new documents or changes to the approved consent form or other documents?**

☐ Consent/assent documents (attach revised version with changes highlighted)

☒ New/revised instruments (attach - if revised, highlight changes)

☒ New/revised advertising materials (attach - if revised, highlight changes)

Do you have a conflict of interest on this project according to Brown's policy? ☐**YES** ☒**NO**

If YES, has this conflict been previously disclosed to the IRB? ☐**YES** ☐**NO**

PI signature: [signature] Date: 4/25/16

1. **Provide a brief lay summary of the overall project. Include enough detail to allow the IRB to evaluate the requested change(s) within the context of the overall project.**

The World Health Organization reported that suboptimal blood pressure (BP) is responsible for more than half of cardiovascular disease mortality world-wide. Furthermore, greater than half of those with hypertension have uncontrolled BP. *A 2009 Institute of Medicine report recommended prioritizing research to "Compare the effectiveness of mindfulness-based interventions (e.g. yoga, meditation, deep breathing training) and usual care in treating… cardiovascular risk factors."* Evidence-based mindfulness interventions, including Mindfulness-Based Stress Reduction, may have some effects on blood pressure, where a recent meta-analysis and systematic review of 4 randomized controlled trials demonstrated significant effects, but evidence of heterogeneity in effect sizes. The methodologically highest quality studies had the smallest effect sizes (range 0-5 mmHg). Mindfulness-Based Stress Reduction (MBSR) has been customized to a number of disease processes, such as Mindfulness-Based Cognitive Therapy for patients with recurrent depression, and Mindfulness-Based Relapse Prevention for patients with substance use addictions. Effect sizes have been increased by customizing mindfulness interventions to diseases of interest. The same may be true for hypertension, *however mindfulness interventions customized for prehypertensive/hypertensive patients have never been investigated. Until methodologically rigorous studies to evaluate customized interventions for hypertension are performed, we will not know if the observed preliminary effects of general mindfulness interventions on blood pressure reduction could be much more effective with a tailored approach.* Consequently, we propose to conduct a stage I behavioral therapy intervention study to evaluate whether MBSR customized to prehypertensive and hypertensive patients has the potential to provide clinically relevant reductions in BP. Consequently the specific aims are:

Stage 1a: Therapy Development/Manual Writing

1. To outline and evaluate key novel elements of mindfulness-based hypertension therapy (MBHT), customized from the evidence-based MBSR. *We hypothesize that the most important novel element will be generation of mindfulness skills specifically applied to hypertension risk factors such as diet, physical activity, obesity, alcohol consumption and antihypertensive medication adherence.* This aim will be achieved using (1) focus groups of participants undergoing the MBHT behavioral intervention, (2) discussion with experts (including cardiologists, epidemiologists, mindfulness experts, mindfulness intervention instructors) prior to, and following pilot testing of MBHT in participants, and (3) clinical judgment of the investigators performing the intervention.
2. To determine effectiveness of MBHT on primary outcomes (systolic blood pressure, retention rates, recruitment rates, and adverse effects) and secondary outcomes (hypertension risk factors such as diet, physical activity, obesity, and antihypertensive medication adherence) in hypertension subgroups, specifically participants with (1) prehypertension, (2) controlled hypertension, and (3) uncontrolled hypertension. Initial decisions about the targeted sample based on hypertension status will be made.
3. To develop an MBHT therapist manual and training program, including procedures for training, supervising, and evaluating therapists. Furthermore, acceptable therapist characteristics will be developed. The manual and training program will include themes such as specification of unique and common elements of MBHT *vs.* other interventions, description of interventions excluded from MBHT, and specification of key treatment parameters such as frequency and duration of treatment, session length, topics addressed, sequence of sessions, as well as therapist adherence and competency

measures. The MBHT training will consist of a therapist manual, a formal didactic training seminar, and at least one closely supervised training session.

Stage Ib: Pilot Trial

4. To determine whether a mindfulness-based hypertension therapy (MBHT) intervention, customized from the evidence-based MBSR, has promise to be an effective behavioral therapy for participants with hypertension and/or prehypertension. We will perform a randomized controlled pilot trial for MBHT *vs.* enhanced usual care control. *We hypothesize that MBHT will have adequate recruitment rates (≥10% of prehypertensive/hypertensive participants invited from physicians' offices), fairly low drop out rates (<15%), and medium effect sizes (e.g. 5-10 mmHg systolic BP) for reduction in blood pressure.*

These findings will provide publishable pilot data that will inform future randomized clinical trials that evaluate effects of MBHT on long-term changes in blood pressure *vs.* usual care and active control groups. *<u>If proven effective, MBHT could be offered as a complementary program in the prehypertensive/hypertensive patient population that contributes to over half of the cardiovascular disease mortality world-wide.</u>*

2. Provide a detailed description of the changes being requested (Use additional pages, if necessary):

We would like to update the IRB on the following changes made to the overall study protocol, recruitment materials, and participant assessments…

- **Change in Study Name –** We have changed the study name <u>from</u>: "*The Mindfulness-based Hypertension Therapy Pilot Study (MBHT)*" <u>to</u> "*The Mindfulness-Based Blood Pressure Reduction Study (MB-BP)*". The rationale behind this change is that the original study title was not completely accurate since the program is not exactly "therapy", as much as it is a tailored intervention to see if participant's blood pressure can be reduced over time. The revision also reflects the fact that we are not only recruiting individuals with hypertension but those with prehypertension as well.

- **Revised NCCIH Safety Monitoring Protocol** – the formal Safety Monitoring Protocol submitted to the NCCIH (a division of the NIH) was revised back on November 26, 2015. We have attached the updated version 1.3 here to be included as part of the study protocol (see Appendix 1). Under the NIH Safety Monitoring Protocol, we are required to check in with study participants regularly (at four week intervals) to assess if they have sustained any physical injuries. Please see p.9-10 of Appendix 1 for complete details on this process. We have copied and pasted the most relevant text below.

  Monitoring of Physical Injuries: All patients will be monitored by study staff at weekly intervals in person or by telephone and assessed every four weeks with questionnaires about physical injuries, specifically the following questions:

  1. Have you sustained any physical injuries during the past four weeks?

     If yes,
     (a) Please describe the injury.
     (b) Please describe what happened to cause the injury.
     (c) Did this injury include:
     i. Broken bone(s)? yes/no
     ii. Lacerations requiring stiches? yes/no
     iii. Concussion? yes/no

     If injuries include any of the above (i – iii), it will be reported to Data Safety Monitoring Committee member Dr. Hank Wu, MD, who is a practicing physician and researcher. He will advise on any follow-up needed. It will also be reported to the chair of the Data Safety Monitoring Board.

- **Baseline and Follow up Assessments (split into two visits) –** Instead of having participants come into the office once for their baseline and follow up assessments, we would like to have them come in on two separate occasions at about one week apart. The reason behind this request is two-fold. First, participants will be asked to wear a Fitbit device, which will monitor their physical activity and sleep. The device will be worn for one week and will need to be returned to the office at the end of the week in order for the data to be synced and uploaded.

By having participants return to the office, it will ensure that the Fitbit tracker is returned and that the data are properly uploaded. The second reason for the return visit will be to allow the participant time to meet with the course instructor. This one-on-one meeting is already part of the mindfulness intervention. It is important to note that, apart from participant travel to and from the office, we are not adding any additional time to the length of the baseline and follow up assessments but rather the assessment content has been divided across two separate visits. If a participant indicates that he/she is unable to come into the office for the second visit, we can allow the participant the option of mailing his/her Fitbit tracker back to the office with a prepaid envelope.

- **Change in Data Collection Mode for Daily Practice Forms and other Class questionnaires** – we would like to change the mode of administration by which our mindfulness-course practice forms and questionnaires are able to be completed by participants. Currently, participants are asked to complete these forms on paper. Moving forward we would like to allow participants the option to complete the course assignments online in Qualtrics. We would still allow participants the option to complete on paper if preferred.

- **Revised Study Recruitment Materials and Protocol** – we have made revisions to our study advertisement material and protocol. Below are details regarding these changes:

  Revised Flyers and Advertisement Cards – we have revised our study advertisement card and flyer. See Appendix 2 for revisions noted with track changes. Note that the content of both the card and the flyer are the same, but that the print format is different (Appendix 3 contains a clean version of each). A summary of the changes as well as the justification for the revisions are below:

  Per feedback from a Graphic Designer at RISD, we have made the following modifications to our study advertisement materials: (1) Moved "Research Study" from title to explanatory paragraph to make more visually appealing. (2) Changed study phone number from PI's personal cell phone to a study cell phone paid for by the grant and indicated that texting as well as calling is permissible. (3) Modified the descriptive language used to describe the study so that it is easier to read and is more specific and accurate.

  Facebook Page and Advertisements – we have created a study Facebook page and would like to begin using Facebook to recruit for the study. See Appendix 4 for screenshots from the study page. Note that we will wait for IRB approval before making this page public.

  Twitter Page – we have created a study twitter page, on which we can tweet about upcoming events (e.g., mindfulness courses). See Appendix 5 for a screenshots of our twitter account page (that can be made public once IRB approval has been received).

  Craigslist Advertisement – see Appendix 6 for craigslist advertisement content. Note that the content is the same as that used for the advertisement cards.

- **NEW Study Webpage** – we have created two new MB-BP study webpages, which are found on the broader Mindfulness and Cardiovascular Health Lab websites. The Mindfulness and Cardiovascular Health Lab is located at the Brown University School of Public Health's Center for Population Health and Clinical Epidemiology and is directed by Dr. Eric Loucks. There are two websites for the Mindfulness and Cardiovascular Health Lab. One is hosted through Brown's domain and the other through an external website platform known as Weebly. The MB-BP study is featured on both websites. See Appendix 7 for screenshots and content from each of the websites. Note that these websites will not be publically launched until IRB approval has been received.

  Website URL's are below:

  Brown University Site: https://www.brown.edu/research/projects/blood-pressure-reduction/home
  Weebly webpage: http://mindfulnesscvlab.weebly.com/

Revisions to the in-class worksheets and assessments – the following revisions have been made to the mindfulness course worksheets / assignments:

> *Weeks 4 and 5: Goal Related to Diet, Alcohol Consumption, Physical Activity, or Stress Reaction/Response (v.1.1, Appendix 8)* – there were errors in the question variable names found in the tables on p.3-5. This was likely due to a cut and paste error. This has since been corrected. We have included a track changed version of Week 4 (Appendix 7). Week 5 is the same exact questions, so we did not include that version here. We can send upon request.

MB-BP Questionnaire Revisions - we have made changes to several of the individual assessments. See below for details as well as Appendices 9-12 for revised assessments with track changes)

Phone Screener: (v.1.5, See Appendix 9)

- Modified the language slightly to improve survey flow, ease of administration and to be more clear and concise about study involvement.
- For other revisions - see individual comments in attached screener document for detailed rationale on revisions

In-Person Screening: (v.1.5, See Appendix 10)

- Added in the Blood Pressure Safety Protocol, which is also reported to the NCCIH as part of the Data Safety Monitoring Plan.
- For other revisions - see individual comments in attached in-person screener document for detailed rationale on revisions

In-Person Baseline Assessment (v.1.5, See Appendix 11)

- Added in the Blood Pressure Safety Protocol, which is also reported to the NCCIH as part of the Data Safety Monitoring Plan.
- To reduce participant burden we have dropped several of the variables collected on the Food Frequency Questionnaire. See marked up FFQ found within Appendix 10 for details on variables dropped.

- We switched the order of administration for the ANT and SART. The SART is a better measure of attention, so it is important that it is completed first, in case the first measure has an effect on the outcome of the second measure completed.
- For other revisions - see individual comments in attached in-person baseline assessment for detailed rationale on revisions

Home Baseline Assessment: (v.1.5, See Appendix 12) - The only revision to this assessment is the addition of a scale known as the "self-efficacy for managing chronic disease questionnaire". See the track changes document for details.

Follow-Up Assessments – Follow-up assessments will take place at 10 weeks, 6 months, and 1 year post baseline. The follow-up assessments will be identical to those administered at baseline (Home and In-Person), with the exception that any variables that are not expected to change over time (e.g., race-ethnicity), will be removed in order to reduce participant burden. We have listed the question items below that will NOT be included in the follow up assessments. The only additional measure during follow-up assessments is recording the amount of formal and informal mindfulness practices that participants are doing (see Appendix 13).

In-Person Assessment – The in-person assessment used at baseline will be the same assessment used for all of the follow-ups.

Home Assessment – The variables found in the Home Baseline Questionnaire that will NOT be included in the follow up assessments are:

PG1_01-03	Personal goals
BQ1_01-03	Age, Race and Ethnicity
BQ1_05-08	Education, degree earned, technical/vocation schooling
CS1_01-04	Parents' education
CE1_01-10	Childhood experiences
FH1_01-04	Family history of hypertension

Measurement of Mindfulness Practices – see Appendix 13 for questions that will be asked during the follow up assessments in order to record the amount of formal and informal mindfulness practices that participants are doing:

3. State the reason (justification) for the requested amendment:

Please see justifications in Section 2 above.

4. What is your assessment of how the changes will affect the overall risk/benefit ratio of the study and the willingness of individuals to participate?

The changes we have made in this amendment affect the risk/benefit ratio in small ways, and if anything, we anticipate the revisions will decrease participant burden. We modified the study name to be more concise about the purpose of the study. We moved around some of the questions in an attempt to improve the overall survey flow and spread out the overall time/effort burden on participants. And although some new question items have been added to the assessments, we have also removed some items. We estimate, that the overall assessment time has not changed.

The revisions to the formalized Safety Monitoring protocol help reduce participant risk by predicting potential areas of concern and by outlining clear procedures with how to manage incidents that may come up.

5. Does the requested amendment require new documents or changes to the approved consent form or other documents?

Yes. Please see **Appendices 1-13.**

Appendix 1	NCCIH Safety Monitoring Protocol (v.1.3)
Appendix 2	Revised MB-BP Recruitment Card – *with track changes*
Appendix 3	Revised MB-BP Recruitment Card and Flyer – *clean copy*
Appendix 4	MB-BP Study Facebook page screenshots
Appendix 5	MB-BP Study Twitter account screenshots
Appendix 6	MB-BP Study Craigslist advertisement screenshots
Appendix 7	MB-BP Study webpage screenshots
Appendix 8	Week 4: Goal Related to Diet, Alcohol Consumption, Physical Activity, or Stress Reaction/Response (v.1.1) – *with track changes*
Appendix 9	Phone Screener (v.1.5) – *with track changes*
Appendix 10	In-person Screener (v.1.5) – *with track changes*
Appendix 11	In-Person Baseline Assessment (v.1.5) – *with track changes*
Appendix 12	Home Baseline Assessment (v.1.5) – *with track changes*
Appendix 13	Measurement of formal and informal mindfulness practice at time of follow ups - NEW

References

[1] Morisky DE, Green LW, Levine DM. Concurrent and predictive validity of a self-reported measure of medication adherence. Med Care. 1986;24 (1):67-74.

Appendix 1:
Safety Monitoring Protocol

(Formal protocol submitted to the NCCIH – revised on November 26, 2015)

PROTECTION OF HUMAN SUBJECTS

MINDFULNESS-BASED HYPERTENSION THERAPY

NIH Grant #: 1UH2AT009145-01

1. Risks to Human Subjects

1.1 Human Subjects Involvement, Characteristics, and Design

1.1.1 Involvement:

Participants will be recruited in part through cardiology and family practices via established relationships with physicians in Rhode Island and Massachusetts, such as Cathleen Hood MD, Sam Dudley MD, Charles Eaton MD and Hank Wu MD, amongst others. Furthermore, advertisements will be posted throughout Rhode Island, and distributed via social media, inviting participants interested in lowering their blood pressure to enroll

MBHT Intervention Description: This study proposes to customize MBSR to participants with prehypertension/hypertension creating an intervention called Mindfulness-Based Hypertension Therapy (MBHT). Specifically, MBHT is based on the standardized MBSR intervention described elsewhere,[153-156] and will consist of nine 2.5-hour weekly group sessions and an 8-hour one-day session, initially led by Dr. Loucks until further instructors are certified in MBHT if initial findings suggest an effect. Dr. Loucks is an expert in cardiovascular physiology, cardiovascular epidemiology, and social epidemiology. He completed MBSR instructor training at UMass Medical School, and has almost 20 years of mindfulness meditation experience, including teaching and mentoring community members, as well as a recent publication record investigating associations of mindfulness with cardiovascular health.[164,183,184] The unique areas of MBHT are expected to be education on hypertension risk factors, hypertension health effects, and specific mindfulness modules focused on awareness of diet, physical activity, medication adherence, alcohol consumption, stress, and social support for behavior change. A preliminary manual has been created based on the standardized MBSR manual developed at UMass Medical School (**Appendix A**), and will be further developed through the approaches described above, and sequentially revised based on participant feedback and preliminary findings.[155,156] MBHT sessions contain instruction and practice in mindfulness meditation, and conversations about stress and coping. Students learn a range of mindfulness skills including body scan exercises, meditation and yoga. Homework consists of practicing skills for ≥45 min/day, 6 days/week.

MBHT builds a foundation of mindfulness skills (e.g. meditation, self-awareness, etc.) through the MBSR curriculum. MBHT then directs attention towards hypertension risk factors. Early in the MBHT, the importance of hypertension for health and mortality is described, along with hypertension risk factors. Participants will have their blood pressure and hypertension risk factors assessed at baseline, and be provided with this information during the first in-person MBHT session. This phase aims to engage participants' interest in hypertension risk factors, and increase motivation for behavior change. MBHT encourages participants to explore personal readiness for change in the different hypertension risk factors, and explore utilizing mindfulness practices to engage with those risk factors that they choose to. Each week, focus is provided on different hypertension risk factors. However, common themes exist across all hypertension risk factors including (1) awareness of thoughts, emotions and physical sensations particularly surrounding hypertension risk factors such as overconsumption of palatable foods, sedentary activities, alcohol consumption, medication adherence; (2) craving, particularly for hypertension risk factors such as overconsumption of palatable foods, sedentary activities, and alcohol consumption; (3) the impact of bringing mindfulness to every moment, particularly in relation to hypertension risk factors. For example, when consuming highly palatable food, bringing awareness to the emotions, thoughts and physical sensations prior to eating it, during eating it, along with the many minutes, if not hours, afterwards. Participants are trained to bring non-judgmental attention to the often short-term pleasure of overconsumption of foods, sedentary activities, heavy alcohol consumption, or not taking medications, and bring non-judgmental attention to the longer term suffering associations with these activities. Through this process, participants are encouraged to reflect on if behavioral choices provide more benefit or harm to their well-being, and to choose the behaviors that bring benefit. (4) Self-care: as awareness of thoughts, emotions and physical sensations increases, and self-regulation will likely increases as a result of the meditation practices, the curriculum will emphasize to participants that it is common for people to start caring for themselves more. It is a way of better knowing ourselves, and through knowing ourselves in in each

moment, we often want to care for ourselves in each moment. This may mean taking medication that will help our health, or being more physical active, eating more healthily, or consuming more moderate amounts of alcohol. The intervention can be modified to change the dose of any of these 4 components, for example via (1) Increased use of specific meditations focused on health behaviors such as diet and physical activity. (2) Increased use of mediations focused on craving. Participants will be trained to notice craving arising, and fading away in relation to hypertension risk factors such as palatable high caloric foods, sedentary activities, and alcohol consumption. (3) Increased used of mindfulness practices related to long term experiences of health behaviors such as alcohol consumption, diet, physical activity and medication use on thoughts, emotions, physical sensations and well-being. (4) Self-care: Increased use of modules that show how mindfulness practice increased self-awareness and in doing so can increase people's desire to care for themselves. The potential use of loving kindness meditation will be considered.

MBSR Intervention Group: MBSR consists of eight 2.5-hour weekly group sessions, led by a certified instructor.[153,154] These sessions contain instruction and practice in mindfulness meditation, as well as conversations of stress, coping, and homework assignments. Students learn a range of mindfulness skills including body scan exercises, sitting meditation, and yoga. Homework consists of practicing these skills for at least 45 minutes per day, 6 days per week, in addition to practicing mindfulness skills during group meetings. The program concludes with an 8-hour intensive mindfulness retreat with the instructor. During and after the program, students are encouraged to pay mindful, nonjudgmental attention to daily activities like walking, eating and talking. One goal is for participants to see that most sensations, emotions, and thoughts are transient events that do not need to be acted upon. Towards the end of the program, participants are provided materials and encouragement to find existing groups in their communities to continue their practice with, such as meditation and yoga centers. The MBSR intervention will be performed by certified MBSR instructors with at least 500 hours of MBSR teaching experience, detailed further in Section 4.1.4.2 of the Research Strategy. There are over 80 instructors in New England. Participants will be enrolled in MBSR courses taking place close to their home or workplace. Having participants take part in standard MBSR courses will improve generalizability of these findings compared to if only a small number of instructors were selected to teach MBSR to participants. MBSR instructor competency and treatment fidelity are further described below.

MBHT and MBSR Competency and Treatment Fidelity: Treatment fidelity strategies will be performed in accordance with recommendations of the NIH Behavior Change consortium, specifically ensuring treatment fidelity in the following five areas: study design, training providers, delivery of treatment, receipt of treatment and enactment of treatment skills,[240] as follows. *Study design*: The study will provide the same treatment dose for each participant enrolled in the MBSR and MBHT interventions, including fixed length and number of frequency of contact sessions for all MBSR and MBHT sessions. All session durations will be recorded, and evaluated monthly by research staff to ensure consistency. Any deviations from the planned duration will be recorded. All class sessions will be audiotaped. Dr. Willoughby Britton and her team will review treatment audiotapes for one of out four sessions, using classes and sessions selected at random. Dr. Britton and her team will conduct competency ratings on these tapes using validated adherence scales (MBCT Adherence Scale, where items 1-11 in the scale are for MBSR, MBHT and MBCT[241]), and provide detailed feedback to treatment providers. We will ensure equivalent dose across conditions, including meditation, yoga and stress reduction training, through tracking the audio recordings. Possible setbacks in implementation of treatment will be addressed, including having a large pool of MBSR and MBHT instructors in the event that specific instructors no longer teach classes. Instructor attrition will be tracked.

Provider training: MBSR and MBHT will be performed by certified instructors. MBSR teacher certification is fairly extensive, and accreditation occurs through the University of Massachusetts Medical School Center for Mindfulness in Medicine, Health Care and Society, detailed elsewhere.[242] Examples of criteria for becoming a certified MBSR teaching include (i) completion of an eight-week MBSR course as a participant, (ii) completion of several multi-day residential training courses in mindfulness based stress reduction practice and teaching, (iii) substantial experience in teaching MBSR, (iv) strong references letters from colleagues and participants who have taken your MBSR courses, (v) completion of several multi-day mindfulness meditation retreats, (vi) have a graduate degree in a field connected to MBSR (e.g. education, psychology, medicine) or demonstration of equivalent understanding through work experience in a related field. There are 89 registered MBSR programs in Massachusetts (60), RI (9) and CT (20) that offer year-round program including the Center for Mindfulness where MBSR originated. Eligible programs must be 8 weeks and the instructors must have completed the MBSR Instructor certification training to participate in the study. *Delivery of treatment*: We will assess participants' perceptions of provider warmth and credibility using brief measures based on the validated

Working Alliance Inventory,[243] and Empathy Scale[244] at Weeks 4 and 8 of the intervention. Feedback will be provided to the interventionist, and measures of warmth and credibility will be adjusted for in sensitivity analyses, to evaluate if results differ when these measures are included vs. excluded. *Receipt of treatment and enactment of treatment skills:* These will be assessed using the Mindfulness Skill Acquisition Scale,[245] which assesses: (i) the frequency of how often participants apply strategies in their lives taught during MBSR and MBHT classes, and (ii) frequency by which participants refrain from using harmful coping strategies, such as mental/behavioral disengagement, substance use (e.g. smoking) and overeating.[245] Participants' meditation diaries, described in Section 5.4, will assess regularity of meditation practice.

Phone-Based Screening: For people who indicate interest in the study, this screening will take place by phone using trained research assistants to assess the exclusion criteria described above, with the exception of blood pressure which will be assessed in-person

In-Person Screening: If participants remain eligible after the phone-based screening, they will attend an in-person screening for blood pressure and medication assessment. If mean blood pressure shows hypertension/prehypertension (≥120 mmHg systolic, ≥80 mmHg diastolic pressure or taking antihypertensive medication), participants will be invited to return for a second blood pressure reading. At that time, if the mean blood pressure across both assessment times is ≥120 mmHg systolic, ≥80 mmHg diastolic pressure, they will be invited to participate in the study.

Baseline Assessments:

(1) *Demographics:* age, race/ethnicity, socioeconomic status (education).
(2) *Family History of Hypertension (FH):* Assesses biological parents' history of having hypertension, based on questions from New England Family Study LEAP Project.
(3) *Childhood socioecononomic status:* retrospective reporting of parents' education, based on standardized questionnaires used in the Atherosclerosis Risk in Communities (ARIC) study.
(4) *Adverse Childhood Experiences:* Measured using the standardized Adverse Childhood Experiences (ACE) questionnaire developed by Vincent Felitti et al.[185,186]
(5) *Depressive symptomatology:* Assessed using Center for Epidemiologic Studies Depression Scale Revised (CESD-R). The CESD survey has been used extensively in the epidemiologic literature to assess depressive symptomatology.[187] The scale was updated to the CESD-R by Van Dam *et al*., which allows diagnosable criteria similar to Diagnostic and Statistical Manual (DSM) of Mental Disorders.[188]
(6) *Anxiety:* Assessed using the validated Beck Anxiety Inventory.[189-195]
(7) *Medication use:* Assessed directly from participants' medication bottles and self-report using standardized forms, including medication name, dose, frequency of use, and reason of use.
(8) *Antihypertensive medication adherence:* Measured using (a) electronic medication bottle caps, and (b) the validated Morisky 8-item questionnaire.[196]
(9) *Systolic and diastolic blood pressure:* Blood pressure will be measured using a calibrated Omron HEM907XL Intellisense automated BP monitor with established validity,[197] following American Heart Association and Joint National Committee (JNC) guidelines.[178,198]
(10) *Anthropometry:* height, weight and waist circumference directly assessed using standard epidemiologic methods.[172]
(11) *Physical activity: (a)* MET minutes per week, assessed using actigraphy. We will use two accelerometers, one (the Actical) worn round the waist, and one (the Actiwatch-64) worn on the wrist (Philips Respironics, Andover, MA),[173,174] that have become the gold standard for such assessment.[175-177] (b) Rapid Assessment Physical Activity Scale. This scale was chosen because it is validated and responsive to behavioral changes including the types (e.g. yoga) introduced in MBHT and MBSR.[199,200]
(12) *Diet*: assessed utilizing the validated Food Frequency that allows for calculation of hypertension-related dietary factors, including salt intake, alcohol consumption, total caloric consumption, fruit and vegetable consumption, and Dietary Approaches to Stop Hypertension (DASH) eating pattern score.[180]
(13) *Cigarette smoking:* current smoking assessed using self-report standardized questions from the New England Family Study.
(14) *Sleep duration:* Sleep duration is assessed using actigraphy devices described above for assessing physical activity.
(15) *Mindfulness:* Assessed using the validated Mindfulness Attention Awareness Scale[204,205] and Five Facet Mindfulness Questionnaire.[205]
(16) *Emotional eating:* measured using the Three Factor Eating Questionnaire Revised 21-item (TFEQ-R21).[207,208]

(17) *Self-control:* assessed using the validated Self-Control Scale short form.[209,210]
(18) *Self-compassion:* Assessed using the validated Self-Compassion Scale (SCS).[113]
(19) *Perceived stress:* Assessed using the validated 10-item Perceived Stress Scale.[212,213]
(20) *Emotion regulation:* Measured using (a) the validated Difficulties in Emotion Regulation Scale,[214] and (b) positive reappraisal using the validated Cognitive Emotion Regulation Scale.[215-217]
(21) *Interoception:* Used the validated Multidimensional Assessment of Interoceptive Awareness (MAIA).[218-220]
(22) *Decentering:* Assessed using the validated Experiences Questionnaire.[221,222]
(23) *Attention control:* Assessed using (a) the Attention Network Test (ANT), which is a brief validated computerized battery measuring three independent behavioral components of attention: conflict resolution, spatial orienting, and alerting.[223], and (b) the Sustained Attention to Response Task (SART). The SART is a validated computerized test of sustained attention, response inhibition (executive function) and self-regulation.[224-226 227-229]
(24) *Craving:* craving for hypertension risk factors, including palatable foods, alcohol, and sedentary activities will be assessed using the validated Craving Experiences Questionnaire. [230]
(25) *Social integration:* Measured using the validated 12-item Interpersonal Support Evaluation List (ISEL-12) measure of social support.[231]
(26) *Loneliness:* Assessed using the validated R-UCLA Loneliness Scale.[232]
(27) *Readiness to change for hypertension risk factors:* Assessed using questionnaire on readiness to change for hypertension risk factors including physical activity, DASH diet, salt intake, overweight/obesity, stress and stress response, antihypertensive medication use, based on the Transtheoretical Model for stages of change.[233,234]
(28) *Functional Magnetic Resonance Imaging (fMRI):* In the UH3 phase only, if secondary data analyses during the UH2 phase indicate evidence of neuroimaging-based measures of self-regulation being important mechanisms, participants in the UH3 phase will undergo an MRI scanning session for approximately 30 minutes at baseline and 10 weeks follow-up. Scans will be acquired with a 3T scanner while the subject is in the resting state. Sample size will be 24 per group. Participants will undergo a separate consent form for fMRI imaging, so that they can be in the MBHT study without imaging if they prefer.

Follow-Up Assessments at 10 Weeks and 6 Months Follow-Up: Questionnaires and assessments administered at 10 weeks and 6 months follow-up are identical to those administered at the first in-person screening assessment and at baseline, with the exception that questionnaires for which the answers should not change or be informative (age, race/ethnicity, education, adverse childhood experiences, family history of hypertension) are not given at follow-ups. In addition adverse events are monitored at both the 10 week and 6 month follow-up period. We are also exploring performing a 1-year follow-up to enhance understanding of longer term target engagement. However due to the relatively brief period of time for the UH2 phase, the 6 month follow-up time is the primary endpoint for decisions on target engagement. In addition adverse events are monitored at all follow-up assessments.

Focus Groups: Focus group assessments of experts (including cardiologists, epidemiologists, mindfulness experts, mindfulness intervention instructors) will occur prior to, and following pilot testing of MBHT in participants. Focus groups of participants undergoing the MBHT behavioral intervention will take place following the final week of class. The goal of the focus groups will be to adapt MBHT to the unique needs of this population. Adaptions may include, for example, types of specific mindfulness exercises, sequence in which modules are introduced, relative emphasis on different types of modules (e.g. mindfulness practices customized to prehypertension/hypertension risk factors vs. general mindfulness practices), and class duration. Focus groups will be facilitated by Dr. Melissa Clark, who is an experienced focus group moderator, along with another team member present to assist and take notes. The groups will be held in comfortable and convenient meeting rooms at the Brown University School of Public Health.

We will follow study procedures similar to those performed during previous epidemiologic studies by our group. In-person screenings will take place at the Brown University Center for Population Health and Clinical Epidemiology, described in Sections 2.7.1 and 2.7.2 at baseline, completion of the 8-week MBHT course and at 6-months follow-up. Major assessment variables are shown in **Table 1.** Qualitative interviews (focus groups) will evaluate participants' experiences such as usefulness of specific modules, duration of sessions, time burden, and overall intervention effectiveness. Interviewer training includes high standards for selection of interviewers who are at ease with strangers, are culturally-sensitive and able to readily establish professional

rapport. Interviewers will be thoroughly trained in the administration of the structured interview, anthropometry, and blood pressure assessments. Routine quality control procedures will be in place regarding calibration of scales and blood pressure instruments. Clinical exams will be supervised by Dr. Loucks to at least monthly to evaluate accuracy of technique, particularly for anthropometry and blood pressure assessment. Re-training will be implemented if needed. Data will be entered and cleaned in an ongoing matter to facilitate quality control and preliminary analyses.

Table 1. Variables measured and assessment times in the Mindfulness-Based Hypertension Therapy Study.

Variables Measured	Asessment Times		
	Baseline	10 weeks	6 months
Demographics	x		
Family history of hypertension	x		
Childhood socioeconomic status	x		
Adverse childhood experiences	x		
Depressive symptomatology	x	x	x
Anxiety	x	x	x
Medication use	x	x	x
Antihypertensive medication adherence	x	x	x
Anthropometry	x	x	x
Physical activity	x	x	x
Diet	x	x	x
Cigarette smoking	x	x	x
Sleep duration	x	x	x
Mindfulness	x	x	x
Emotional eating	x	x	x
Self-control	x	x	x
Self-compassion	x	x	x
Perceived stress	x	x	x
Emotion regulation	x	x	x
Interoception	x	x	x
Decentering	x	x	x
Attention control	x	x	x
Craving for hypertension risk factors	x	x	x
Social integration	x	x	x
Loneliness	x	x	x
Readiness to change for hypertension risk factors	x	x	x

1.1.2 Inclusion/exclusion criteria:

Inclusion Criteria: Hypertension/prehypertension (≥120 mmHg systolic, ≥80 mmHg diastolic pressure or taking antihypertensive medication). Able to speak, read, and write in English. All adults (≥18 years of age), genders and racial/ethnic groups are eligible to be included.

Exclusion Criteria: Exclusion criteria follow standard guidelines and recommendations:[3] (a) current regular meditation practice (>once/week); (b) serious medical illness precluding regular class attendance; (c) current substance abuse, suicidal ideation or eating disorder, (d) history of bipolar or psychotic disorders or self-injurious behaviors. These participants are excluded because they may disrupt group participation, require additional or specialized treatment, or are already participating in practices similar to the intervention.

1.1.3 Vulnerable populations:

There are no participants in the study we are aware of who are considered part of a vulnerable population.

1.1.4 Collaborating sites:

There are no other collaborating sites that will assess participants during this study.

1.1.5 Data management:

The clinical data will be de-identified but linked. Private information such as name date of birth, next of kin, address for recontacting will be kept in a password protected, encrypted database on a different disk that the clinical data. Only the principal investigators for the purposes of patient safety or monitoring by the NIH, data safety monitoring boards or HIPPA compliance officer approved agents will be given access to identifiable personal information.

1.1.6 Equipoise

Clinical equipoise, defined as “no consensus within the expert clinical community about the comparative merits of the alternative [trial arms] to be tested”[138] applies to this study, and it will be designed in a way that the findings should disrupt clinical equipoise. The study will be described to potential participants as a

randomized controlled trial that will evaluate the impact of a stress reduction program on risk for cardiovascular disease. The statements in the study description and consent forms will show equipoise (i.e. "a state of genuine uncertainty on the part of the clinical investigator regarding the comparative therapeutic merits of each arm in a trial"[138]), where the state of the research does not know conclusively know whether the stress-reduction program will have an effect on subclinical cardiovascular disease (hence we are performing the trial). For example, the consent form will describe that is not currently known whether the stress reduction intervention we are testing reduces cardiovascular disease risk. Similarly, all study personnel including the PI will be instructed to demonstrate equipoise to the best of their ability, regardless of personal biases (in either direction – favoring treatment or control), so that any types of biases on treatment effects will not be communicated to study participants. Instruction will be provided for all study personnel on the importance of demonstrating equipoise. Further instruction will be provided on ways to distinguish appropriately among (1) validated knowledge accepted by the clinical community, (2) data on treatments that are promising but are not generally convincing, and (3) mere hunches, including how these principles apply to the current study.[138]

1.2 Sources of Materials

1.2.1 Research material obtained from living individuals

1.2.1.1 Outcomes

Primary Outcomes

Self-regulation will be assessed using targets identified in the UH2 phase. A list shown above in the Baseline Assessments section. Examples include emotional eating (measured using the Three Factor Eating Questionnaire),[207,208] self-control (assessed using the validated Self-Control Scale short form.[209,210]), self-compassion (Assessed using the validated Self-Compassion Scale (SCS).[113]), emotion regulation (measured using the validated Difficulties in Emotion Regulation Scale,[214] and positive reappraisal using the validated Cognitive Emotion Regulation Scale.[215-217]), interoception (using the validated Multidimensional Assessment of Interoceptive Awareness[218-220]), decentering (assessed using the validated Experiences Questionnaire.[221,222]), attention control (assessed using the validated Attention Network Test,[223], and the Sustained Attention to Response Task[224-226 227-229]), and craving for hypertension risk factors, including palatable foods, alcohol, and sedentary activities will be assessed using the validated Craving Experiences Questionnaire.[230]

Secondary Outcome

Prehypertension/Hypertension Medical Regimen Adherence Outcomes Assessment Methods: (1) Electronically-Measured Antihypertensive Medication Adherence: measured continuously using electronic medication bottle caps. (2) Body Mass Index: height and weight directly assessed using standard epidemiologic methods.[172] (3) Physical activity: MET minutes per week, assessed using actigraphy. We will use two accelerometers, one (the Actical) worn round the waist, and one (the Actiwatch-64) worn on the wrist (Philips Respironics, Andover, MA),[173,174] that have become the gold standard for such assessment.[175-177] Adherence to Joint National Commission-7 (JNC-7) guidelines is 30 min aerobic physical activity ≥4 days per week.[178] (4) Diet: assessed utilizing Dietary Approaches to Stop Hypertension (DASH) eating pattern score,[179] measured via diet history food frequency questionnaire.[180] Adherence to JNC-7 guidelines DASH eating pattern score (range 0-8).[178,179] (5) Alcohol consumption: Amount and frequency of alcohol consumption, will be assessed via self-report utilizing standard questions from the behavioral Risk Factor Surveillance Survey.[181] JNC-7-stated cut-point of healthy alcohol intake is ≤ 2 drinks (e.g. 24 oz. beer, 10 oz. wine, or 3 oz. 80-proof whiskey) per day in men and ≤1 drink per day in women.[178] Medical regimen adherence will be defined as adherence to JNC-7-recommended behavioral and medication treatment of hypertension.[178] Specifically, analyses will assess changes in mean Z-scores of health behaviors or medication adherence in 6 domains, being (1) Physical activity assessed via MET scores. (2) Electronically-measured antihypertensive medication adherence measured continuously using electronic medication bottle caps, where medication adherence is calculated as the percent of days during which medication was taken as prescribed. For multi-day dosings, participants will receive points for the proportion of actual over expected dosings. (3) Body Mass Index (kg/m^2), (4) Diet via Dietary Approaches to Stop Hypertension (DASH) eating pattern score[179] (5) Alcohol consumption (number of drinks per day).

1.2.2 Who will have access to individually identifiable private information about human subjects.

Only the principal investigators for the purposes of patient safety or monitoring by the NIH, data safety monitoring boards or HIPPA compliance officer approved agents will be given access to identifiable personal information.

1.2.3 How the specimens, records, and/or data are collected, managed, and protected as well as whether material or data that include individually identifiable private information will be collected specifically for the proposed research project.

Questionnaire data will typically be collected using Qualtrics, LLC (Provo, UT, USA) survey instruments, so that participants can complete questionnaires on their own time at home, using their computers or smart phones. Exceptions to this include the phone screening questionnaire, and the baseline and follow-up questions on depression and anxiety to allow for a safety protocol to be followed if there are high levels of depression, anxiety or suicidal ideation.

Assessments of blood pressure, height, weight, waist circumferences and medication use are assessed in-person by trained research assistants blinded to treatment allocation.

Confidentiality of Patient Records: The clinical data will be de-identified but linked. Private information such as name, date of birth, and address for recontacting will be kept in a password protected, encrypted database on a different disk than the clinical data. Only the principal investigator for the purposes of patient safety or monitoring by the NIH, data safety monitoring boards or HIPPA compliance officer approved agents will be given access to identifiable personal information.

Data management will be performed by downloading data at minimum every 2 weeks during data collection periods, and assessing data for missingness and errors. Data will be maintained in password-protected Microsoft Excel Spreadsheets, and then exported using .csv functions for analysis in SAS software.

1.3 Potential Risks and Minimization of Risk

1.3.1 Expected Risks

The following is a statement from NCCIH about the potential risks of meditation practice: “Meditation is considered to be safe for healthy people. There have been rare reports that meditation could cause or worsen symptoms in people who have certain psychiatric problems, but this question has not been fully researched. People with physical limitations may not be able to participate in certain meditative practices involving physical movement. Individuals with existing mental or physical health conditions should speak with their health care providers prior to starting a meditative practice and make their meditation instructor aware of their condition.”[235]

Discomfort during meditation: Meditation-based interventions may results in discomfort with attention to unpleasant thoughts, feelings or body sensations. Some individuals may experience an initial increase in undesirable emotions with increased attention to them. In order to minimize risk, instructors encourage participants to be aware of their discomfort and to set their own limitations. Participants are encouraged to alter their postures, including standing, sitting in chairs, lying down, as needed in order to be comfortable. In addition, the curriculum of the training program is specifically geared towards addressing such discomfort.

Psychological distress: Research subjects participating in this study may have feelings of loss of privacy from being contacted about participating in the study, and possible psychological distress caused by questions on the diagnostic interview that bring up painful memories or feelings. However, the resulting potential for injury to research subjects is judged to be minimal. We have already contacted and clinically evaluated thousands of participants from other studies such as the New England Family and Women’s Health Initiative using similar assessment procedures to this study, with good responses from the participants. With regard to psychological distress from taking part in the MBSR or MBHT intervention, given that screening questions will exclude participants with substantial mental illness, and given the NCCIH statement above that “Meditation is considered to be safe for healthy people.”[235] we expect that risk of psychological distress will be low. The risk of increased psychological distress from meditation will be clearly outlined in the consent form and participants will be encouraged to consult with both the course instructor and study staff in the case of any increased distress. Dr. Ellen Flynn, a licensed psychiatrist, will be available to advise on any psychological events that occur, and provide referrals for treatment if needed.

Loss of confidentiality: Likelihood: rare. Minimization: Confidentiality will be maintained by numerically coding all data, by disguising identifying information, and by keeping all data in locked file drawers. All information obtained from participants will be accessible only to research staff.

Injury due to physical activities: It is possible that injuries could be sustained from (1) the gentle mindful movements (yoga), or (2) physical activities that participants engage in as a result of the intervention

encouraging exploration of physical activity as a way to reduce blood pressure. *(1) Mindful movements:* Participants receive a handout during the orientation showing the yoga poses that will be offered during the course. They are encouraged to explore limits in their body related to movement, but not to go beyond those limits Participants are asked to listen to what their body is telling them more closely than what the mindful movement instructor is telling them. Modifications of poses are available, including for those limited to chairs or wheelchairs. Participants are encouraged to bring the handout of poses to their health care providers if they have any physical limitations, so that the providers can advise on which poses to do, and which to avoid. *(2) Physical activities:* Participants are encouraged to explore physical activities that promote strength and conditioning as a way to reduce blood pressure. As with the mindful movements, they are encouraged to explore limits in their body related to movement, but not to go beyond those limits. Participants are asked to listen to what their body is telling them more closely than what the mindful movement instructor is telling them. Furthermore, they are encouraged to ask their healthcare provider about advised physical activities if they have any physical limitations.

Risks associated with fMRI: This study will be conducted in a 3T MR scanner at UMass Medical School, which has been approved for research and clinical studies in children and adults by the FDA. Magnetic resonance (MR) technology does not use X-rays, it uses strong magnetic fields and radio waves. Subjects will be asked whether they have devices that can be affected by MRI, and if so, they will not be able to participate in this study. Significant risks also can arise if ferromagnetic materials are brought into the high magnetic field environment of the scanner and immediate vicinity, as they can become hazardous projectiles. These types of items are not permitted in the scanning area. The MR exams are painless, and except for the pulsating sounds, subjects will not be aware that MR scanning is taking place. With proper safety precautions in terms of the avoidance of metal objects, there are no known health risks associated with MRI. The safety of MRI is reflected in the fact that it is used in standard medical practice without the requirement for informed patient consent. Most people experience no ill effects from the magnetic field, but some report claustrophobia, dizziness, mild nausea, headaches, a metallic taste in their mouth, double vision, or a sensation of flashing lights. These symptoms are transient and resolve quickly after the subject exits the scanner. The technologist will be able to hear subjects at all times and subjects are free to end the procedure at any time. In rare cases, a very slight, uncomfortable tingling of the back due to the rapid switching of the magnetic field has been reported during certain types of scans. Subjects are asked to report this immediately so the scan can be changed to avoid this. Although these precautions will avoid all known risks associated with MR, this procedure may involve risks that are currently unknown. The scanner is noisy, but does not harm hearing. For comfort, subjects will be given earplugs to muffle the noise.

Risk of adverse events during the study: It is possible that some patients will have an adverse event during the study, including increased stress or anxiety. Participants with major mental health conditions, such as bipolar depression, suicidal ideation, borderline personality disorder, post-traumatic stress disorder, and obsessive compulsive disorder, are ineligible for the study. We expect risk of adverse events to be very low.

Impact statement: These risks are considered to be minimal and are addressed in the protocol and consent form.

Adverse Events

Mental Health Deterioration and Suicidality: Participants assigned to any treatment group may experience mental health or suicidal ideation. All patients will be monitored by study staff at weekly intervals in person or by telephone and assessed every four weeks with questionnaires about anxiety, depression and suicidal ideation, specifically the Beck Anxiety Inventory and the Center for Epidemiology Study Depression Scale Revised (CESD-R). Dr. Flynn is a licensed psychiatrist and has extensive experience evaluating research participants for clinical deterioration or suicidality.

Beck Anxiety Inventory (BA): If participant scores ≥26 on the Beck Anxiety Inventory, while having the participant wait, immediately notify the collaborating psychiatrist, Dr. Ellen Flynn (cell phone # 401-258-9829), to determine need for a psychiatric consultation. She has provided her cell phone #, and your phone # will be entered into her phone recognition software to indicate it is a call from the MBHT study. It is likely she will answer the phone. If Dr. Flynn cannot be reached, please leave a phone message for her with your contact information and provide her with the Beck Anxiety Inventory results on the message. If Dr. Flynn is not

immediately reachable, or following participant release after consulting with Dr. Flynn, immediately contact the PI, Dr. Eric Loucks (cell phone #401-369-0443).

Depressive Symptomatology: The CESD-R will be administered during the in-person assessment visits, and scores will be reviewed immediately upon completion of the in-person assessments.

1. Sadness (dysphoria): Question numbers 2,4, 6
2. Loss of Interest (anhedonia): Question numbers 8, 10
3. Appetite: Question numbers 1, 18
4. Sleep: Question numbers 5, 11, 19
5. Thinking / concentration: Question numbers 3, 20
6. Guilt (worthlessness): Question numbers 9, 17
7. Tired (fatigue): Question numbers 7, 16
8. Movement (agitation): Question numbers 12, 13
9. Suicidal ideation: Question numbers 14, 15

Participants are considered to meet criteria for major depressive episode if they have anhedonia or dysphoria nearly every day for the past two weeks, plus symptoms in an additional 4 DSM symptom groups noted as occurring nearly every day for the past two weeks. If participants meet criteria for major depressive episode, while having the participant wait, staff will immediately notify the collaborating psychiatrist, Dr. Ellen Flynn (cell phone # 401-258-9829), to determine need for a psychiatric consultation. She has provided her cell phone #, and the staff's phone #'s will be entered into her phone recognition software to indicate it is a call from the MBHT study. It is likely she will answer the phone. If Dr. Flynn cannot be reached, staff will leave a phone message for her with their contact information and provide her with the Depressive Symptomatology results on the message. If Dr. Flynn is not immediately reachable, or following participant release after consulting with Dr. Flynn, staff will immediately contact the PI, Dr. Eric Loucks (cell phone #401-369-0443).

If participants respond having any suicidal ideation (CES-D questions 14 or 15), staff will perform the following 2 steps:

1. Immediately call 911 and Dr. Ellen Flynn. Specifically, while the participant is in the waiting room, staff will call 911 immediately, and tell them about the situation. This will be followed by a call to Dr. Flynn.

2. While speaking calmly with the participant, staff will let them know that he/she called 911 and Dr. Flynn, and why (i.e. because we are concerned about them). Staff can speak with participant to keep him/her in the waiting room if 911 has sent assistance, but the discussion should not be clinical in nature.

The following information can be provided to study participants.

National Suicide Prevention Lifeline: 1-800-273-8255

Other options are to:
- Call your doctor's office
- Call 911 for emergency services
- Go to the nearest hospital emergency room.

Local Non-Urgent Free or Inexpensive Mental Health Services:
Gateway Healthcare: 401-729-8701
Anchor Counseling Center: 401-475-9979
The Providence Center: 401-276-4020

Injury due to physical activities: It is possible that injuries could be sustained from (1) the gentle mindful movements (yoga), or (2) physical activities that participants engage in as a result of the intervention encouraging exploration of physical activity as a way to reduce blood pressure.

(1) Mindful movements: Participants receive a handout during the orientation showing the yoga poses that will be offered during the course. They are encouraged to explore limits in their body related to movement, but not to go beyond those limits Participants are asked to listen to what their body is telling them more closely than what the mindful movement instructor is telling them. Modifications of poses are available, including for those limited to chairs or wheelchairs. Participants are encouraged to bring the handout of poses to their health care providers if they have any physical limitations, so that the providers can advise on which poses to do, and which to avoid.
(2) Physical activities: Participants are encouraged to explore physical activities that promote strength and conditioning as a way to reduce blood pressure. As with the mindful movements, they are encouraged to explore limits in their body related to movement, but not to go beyond those limits. Participants are asked to listen to what their body is telling them more closely than what the mindful movement instructor is telling them. Furthermore, they are encouraged to ask their healthcare provider about advised physical activities if they have any physical limitations.

All patients will be monitored by study staff at weekly intervals in person or by telephone and assessed every four weeks with questionnaires about physical injuries, specifically the following questions:

1. Have you sustained any physical injuries during the past four weeks?

   If yes,
   (a) please describe the injury
   (b) please describe what happened to cause the injury.
   (c) did this injury include
      i. Broken bone(s) yes/no
      ii. Lacerations requiring stiches yes/no
      iii. Concussion yes/no

If injuries include any of the following, it will be reported to Data Safety Monitoring Committee member Dr. Hank Wu, MD, who is a practicing physician and researcher. He will advise on any follow-up needed. It will also be reported to the chair of the Data Safety Monitoring Board.

a. Broken bone(s)
b. Lacerations requiring stiches
c. Concussion

Non-Response to Treatment: The possibility that the treatment will not yield benefit is another possible risk and will be explained during informed consent procedures. Non-responders (identified as minimal change in medical regimen adherence from baseline assessment) will be provided with referrals to other treatment, if desired.

1.4 Safety Monitoring

Oversight of internal monitoring of the participants' safety will be conducted by the PIs, Dr. Eric Loucks and Dr. Willoughby Britton. Oversight of the external Data and Safety Monitoring Committee will be conducted by the chair (Dr. Edmondson). The Data and Safety Monitoring Committee will include experts in cardiology, psychology/psychiatry, epidemiology, and biostatistics.

Entities Conducting Monitoring

The Institutional Review Board (IRBs) at Brown University will review all research procedures, and will provide oversight. Internal monitoring will be done by the Brown University principal investigators (Dr. Loucks and Dr. Britton) and the Brown University IRB. The Data Safety Monitoring Committee will provide external monitoring, and will meet every six months by phone or in-person. They will be provided data every six months to evaluate potential effects of the RCT on major outcomes (e.g. medical regimen adherence). Any serious adverse effects will be immediately reported to the principal investigators (Loucks, Britton) and the committee chair.

What is Monitored

Monitoring is done of all procedures to ensure that they conform to the approved protocol; of unforeseen circumstances that might arise and affect safety; of all reports of serious adverse events as defined in US Department of Health and Human Services regulations for the protection of human research subjects 45 CFR Part 46, and the FDA 312.32 (death, life-threatening experience, new or prolonged hospitalization, persistent or significant disability/incapacity); of other significant adverse events (adverse events that lead to drop out by participant or termination by the investigator); of unexpected adverse events resulting from the study; and of expected adverse events.

Monitoring is done of all study inclusion and exclusion criteria. During this clinical trial, we will notify officials, as mandated by law, if a participant reports intentions to harm him/herself or others, or reports child abuse or abuse of an elder. Dr. Ellen Flynn, a licensed psychiatrist, will be available to advise on any psychological events that occur, and provide referrals for treatment if needed.

Frequency of Monitoring

All adverse events will be continuously monitored by the PI. Participants will be given contact information so that they can inform us of events that occur in between study visits. Dr. Loucks will conduct daily oversight of participant safety. He will meet weekly with staff to review participant progress and their experiences with the experimental procedures, including adverse events. Any adverse events that are observed and/or reported will be immediately reported to Dr. Loucks, Dr. Britton, and the Data Safety Monitoring Committee chair. The Investigators and Data Safety Monitoring Committee will be available to meet outside of the regularly scheduled meetings (scheduled semiannually), if necessary, due to concerns regarding a particular participant or any problems that may arise for participants. If necessary, they will make appropriate recommendations for changes in protocol, or terminate the study. The Brown University IRB conducts the monitoring at the continuing reviews as scheduled, whenever modification requests are considered, and upon receiving reports of serious adverse events from the PI or anyone else.

Reporting Plan

Any serious adverse events that are observed and/or reported will be immediately reported to Dr. Loucks and the Data Safety Monitoring Committee Chair. Serious adverse events are then reported to the Brown University IRB and to NIH. Brown's IRB requires fatalities related to the study be reported within 24 hours. All serious adverse events related to this study will be reported to the Brown University IRB immediately by telephone and by written report within 48 hours of our receipt of information regarding the event. All other adverse events related to the study will be reported at the continuing review. Serious adverse events will also be reported in writing to the NIH Project Officer within 48 hours. All serious adverse events related to the study will be reported annually in the Progress Report sent to the NIH Project Officer.

Any actions taken by the IRB, other than acceptance of the adverse event report, will be reported to the NIH along with any changes or amendments to the protocol requested by the IRB in response to these reports. Proposed changes or amendments to the protocol in general must be requested first in writing to the Brown and IRB, which will then grant or deny permission to make the requested change or amendment in protocol. NIH will subsequently be informed of any substantive changes or amendments in approved protocol.

2 Adequacy of Protection Against Risks

2.1 Recruitment and Informed Consent

2.1.1 Recruitment:

Participants will be recruited in part through cardiology and family practices via established relationships with physicians in Rhode Island and Massachusetts, such as Cathleen Hood MD, Sam Dudley MD, Charles Eaton MD and Hank Wu MD, amongst others. Furthermore, advertisements will be posted throughout Rhode Island, and distributed via social media, inviting participants interested in lowering their blood pressure to enroll.

2.1.2 Informed Consent:

All informed consent forms and protocols have been reviewed and approved by the Brown University IRB. Each potential participant will be fully informed about the nature of the study, its risks and benefits, and

about his/her rights as a research subject. The participant will sign and receive a copy of the document stating that he/she has given his/her informed consent to participate before interviewing is begun. In every instance, project personnel will verbally review the content of the consent form before participants sign.

2.2 Protections Against Risk

Please refer to Section 1.3 above.

3 Potential Benefits of the Proposed Research to Human Subjects and Others

There may be no direct benefits from participating in this study. The risks to participants are minimal. The potential benefits of participating in the proposed study include improved knowledge on stress reduction techniques and risk factors for cardiovascular disease. Each participant will also be contributing to an important study which will answer questions on whether mindfulness-based stress reduction may influence health.

4 Importance of the Knowledge to be Gained

There is great interest in understanding the impact of mindfulness-based interventions on risk for cardiovascular disease, however the vast majority of studies to date have had substantial methodological limitations, leading at 2007 government report on "Meditation Practices for Health: State of the Research" to conclude that "as a whole, firm conclusions on the effects of meditation practices in healthcare cannot be drawn based on the available evidence."[36] This study proposes to fill a substantial gap in knowledge, and rigorously evaluate the effects of a mindfulness-based intervention on risk for hypertension and cardiovascular disease. Expected outcomes are to inform recommendations on whether mindfulness-based stress reduction has long-term impacts on subclinical cardiovascular disease and cardiovascular disease risk factors. Findings will further inform health care providers, communities, workplaces and individuals, who are currently grappling with whether to include mindfulness practices as part of approaches to reduce risk for cardiovascular disease.

5 Data and Safety Monitoring Plan

Please see Section 1.4 above for Safety Monitoring details.

Data Safety Monitoring Board:

Oversight of internal monitoring of the participants' safety will be conducted by the PIs, Dr. Eric Loucks and Dr. Willoughby Britton. Investigators on this application have extensive experience with clinical trials for mindfulness-based interventions and cardiovascular outcomes. Oversight of the external Data and Safety Monitoring Committee will be conducted by the chair, Dr. Donald Edmondson, PhD, is Assistant Professor of Behavioral Medicine at Columbia University Medical Center. He is a Psychologist, and has extensive research experience in evaluating effects of stress and psychsocial factors on cardiovascular disease outcomes.[246-264] The Data and Safety Monitoring committee will also include Dr. Wen-Chih (Hank) Wu and Dr. Tao Liu. Dr. Wu, MD, is Associate Professor of Medicine and Associate Professor of Epidemiology at Brown University. He is a practicing clinical cardiologist with research in preventive cardiology,[265-272] and will be able to advise on clinical outcomes and any cardiovascular complications arising from the study, in addition to methodological concerns. Dr. Liu, PhD, is an Assistant Professor of Biostatistics at Brown University, experienced in clinical trials.[273,274] He will receive all preliminary analyses from the primary statistician, and will have access to all data from the study, to evaluate any evidence of serious adverse effects or other concerns.

Entities Conducting Monitoring

The Institutional Review Board (IRBs) at Brown University will review all research procedures, and will provide oversight. Internal monitoring will be done by the principal investigators (Dr. Loucks and Dr. Britton) and the Brown University IRB. The Data Safety Monitoring Committee will provide external monitoring, and will meet every six months by phone or in-person. During the randomized-controlled trial phase (phase 4), they will be provided data every six months to evaluate potential effects of the RCT on the primary outcome (i.e. medical regimen adherence). Any serious adverse effects will be immediately reported to the principal investigator (Loucks) and the committee chair (Edmondson).

5.1 Entities Conducting Monitoring

The Institutional Review Board (IRBs) at Brown University will review all research procedures, and will provide oversight. Internal monitoring will be done by the Brown University principal investigators (Dr. Loucks

and Dr. Britton) and the Brown University IRB. Dr. The Data Safety Monitoring Committee will provide external monitoring, and will meet every six months by phone or in-person. They will be provided data every six months to evaluate potential effects of the RCT on major outcomes (e.g. medical regimen adherence). Any serious adverse effects will be immediately reported to the principal investigators (Loucks, Britton) and the committee chair.

5.2 What is Monitored

Monitoring is done of all procedures to ensure that they conform to the approved protocol; of unforeseen circumstances that might arise and affect safety; of all reports of serious adverse events as defined in US Department of Health and Human Services regulations for the protection of human research subjects 45 CFR Part 46, and the FDA 312.32 (death, life-threatening experience, new or prolonged hospitalization, persistent or significant disability/incapacity); of other significant adverse events (adverse events that lead to drop out by participant or termination by the investigator); of unexpected adverse events resulting from the study; and of expected adverse events.

Monitoring is done of all study inclusion and exclusion criteria. During this clinical trial, we will notify officials, as mandated by law, if a participant reports intentions to harm him/herself or others, or reports child abuse or abuse of an elder. Co-investigator Dr. Willoughby Britton is a clinical psychologist, who will be available on call in case of any psychological adverse events.

5.3 Frequency of Monitoring

All adverse events will be continuously monitored by the PI. Participants will be given contact information so that they can inform us of events that occur in between study visits. Dr. Loucks will conduct daily oversight of participant safety. He will meet weekly with staff to review participant progress and their experiences with the experimental procedures, including adverse events. Any adverse events that are observed and/or reported will be immediately reported to Dr. Loucks, Dr. Britton, and the Data Safety Monitoring Committee chair. The Investigators and Data Safety Monitoring Committee will be available to meet outside of the regularly scheduled meetings (scheduled semiannually), if necessary, due to concerns regarding a particular participant or any problems that may arise for participants. If necessary, they will make appropriate recommendations for changes in protocol, or terminate the study. The Brown University IRB conducts the monitoring at the continuing reviews as scheduled, whenever modification requests are considered, and upon receiving reports of serious adverse events from the PI or anyone else.

5.4 Reporting Plan

All adverse events will be continuously monitored by the PI. Participants will be given contact information so that they can inform us of events that occur in between study visits. Dr. Loucks will conduct daily oversight of participant safety. He will meet weekly with staff to review participant progress and their experiences with the experimental procedures, including adverse events. Any adverse events that are observed and/or reported will be immediately reported to Dr. Loucks, Dr. Britton, and the Data Safety Monitoring Committee chair. The Investigators and Data Safety Monitoring Committee will be available to meet outside of the regularly scheduled meetings (scheduled semiannually), if necessary, due to concerns regarding a particular participant or any problems that may arise for participants. If necessary, they will make appropriate recommendations for changes in protocol, or terminate the study. The Brown University IRB conducts the monitoring at the continuing reviews as scheduled, whenever modification requests are considered, and upon receiving reports of serious adverse events from the PI or anyone else.

Any actions taken by the IRB, other than acceptance of the adverse event report, will be reported to the NIH along with any changes or amendments to the protocol requested by the IRB in response to these reports. Proposed changes or amendments to the protocol in general must be requested first in writing to the Brown and IRB, which will then grant or deny permission to make the requested change or amendment in protocol. NIH will subsequently be informed of any substantive changes or amendments in approved protocol.

5.5 Data Quality Assurance and Confidentiality

Several procedures currently in practice in our laboratory will also be utilized for this study to guarantee the validity, integrity, accuracy, and completeness of the data. It will be made clear to all participants that all information obtained during assessments is confidential.

Procedures to guarantee the accuracy and completeness of the data during data collection, transmission, and analysis: The data manager (Saadeh) will review each file within 2 weeks of data collection for

completeness, accuracy, and validity of responses. Data files are accessible only to project personnel and are password protected. Ms. Saadeh will review the distributions of all raw data to ensure that data are within range and to check missing data with the hard copy of the data. The project database is password protected and accessible only to the project's data managers, the biostatistician, and the PI.

Procedures to maintain confidentiality: Study staff will maintain strict confidentiality regarding all aspects of a subject's participation in this study. Any individual information gathered in the course of the study will not be discussed with anyone who is not directly involved with this study. For each stage of the research, participant names and contact information will be maintained in a recruitment/enrollment database during the course of the study. Once individuals enroll in the study, names will be linked to participant ID number in this database, which will be kept in a restricted access folder on a secure server. This file will be assigned a code name unrelated to the name of the study. Signed consent forms will be kept in a locked file cabinet, separate from any other project data. Once data collection is completed, the corresponding recruitment/enrollment database will be deleted as it is unnecessary to maintain the link between participant identity and study data. All information collected as part of this study will be accessible only to research staff who have completed mandatory training in the protection of human subjects.

6 ClinicalTrials.gov Requirements

The RCT will be registered, and results reported, at Clinical Trials.gov in accordance with NIH recommendations. Registration in ClinicalTrials.gov will occur no later than 21 days after the first subject is enrolled. Reporting of results (including adverse events) will occur no later than 1 year after completion date.

APPENDICES

A. MBHT Curriculum Guide

B. CVs of Data Safety Monitoring Board Members

Curriculum Vitae
Prepared 10.6.2014

Personal Data

Name: Donald Edmondson
Birthdate: 12.12.1979
Birthplace: Wynne, AR
Citizenship: U.S.

Education

2013 M.P.H., Effectiveness and Outcomes Research; Mailman School of Public Health, Columbia University, New York, New York

2009 Ph.D., Personality Psychology; University of Connecticut, Storrs, CT;

Dissertation: **Edmondson, D**., et al. From shattered assumptions to weakened worldviews: Trauma symptoms signal anxiety buffer disruption. *Journal of Loss and Trauma*. 2011, 16, 358-85. (Crystal L. Park, Ph.D., Dissertation Chair).

2007 M.A., Clinical Psychology; University of Connecticut, Storrs, CT

2001 B.A., History; Union University, Jackson, TN.

Postdoctoral Training

2009-2010 Postdoctoral Research Scientist, Center for Behavioral Cardiovascular Health, Columbia University Medical Center, New York, NY

Hospital Appointment

2010-2011 Associate Research Scientist, Center for Behavioral Cardiovascular Health, Columbia University Medical Center, New York, NY

Academic Appointment

2011-present Assistant Professor of Medicine (Departments of Medicine and Psychiatry), Center for Behavioral Cardiovascular Health, Columbia University Medical Center, New York, NY

Professional Organizations and Societies

American Psychological Association
Association for Psychological Science
Society of Behavioral Medicine
American Psychosomatic Society
Society for Personality and Social Psychology

Honors

2014 Neal E. Miller Young Investigator award, Academy for Behavioral Medicine Research
2014 Grant reviewer, Israel Science Foundation
2013 Invited to join Social Psychology, Personality, and Interpersonal Processes (SPIP) study section as a permanent member
2011 NIH Early Career Reviewer, Social Psychology, Personality, and Interpersonal Processes (SPIP) study section
2011 Columbia Summer Research Institute selection
2010-present NIH Loan Repayment Grant recipient
2007 & 2008 Highest Distinction in Undergraduate Instruction, University of Connecticut
2006 Sydney Jourard Award for best paper, Division 32 of the Am Psych Assoc

2003	Psi Chi (psychology honors society), University of Memphis
1997-2001	Scholars of Excellence Provost's Scholar Award, Union University

Current Grants and Contracts:

Title of Project and Source	Dates
R01 HL117832 (**Edmondson**, PI)<br>**NIH/NHLBI**<br>Impact of Social-Interpersonal Factors in the ER on PTSD/Cardiac Outcomes	2013-2018
Loan Repayment Grant (**Edmondson**)<br>**NIH/NHLBI**<br>PTSD Due to Acute Coronary Syndromes<br>Role: Awardee	2011-2014
P01 HL088117 (Davidson, PI)<br>**NIH/NHLBI**<br>Depression, Biobehavioral Mechanisms & CHD/Mortality Outcomes<br>Role: Co-Investigator (in-kind support)	2008-2013
P01 HL47540 (J Schwartz, PI)<br>**NIH/NHLBI**<br>Psychosocial Factors and Cardiovascular Disease<br>Role: Co-Investigator (in-kind support)	1993-2014
ECRIP-HPR iSCRIPT Center (Davidson, PI)<br>**New York State Department of Health**<br>Innovative Strategies to deCrease Readmissions through Improving Patient & System sTress & behavior<br>Role: Training Director	2014-2016

Completed

KM1 CA156709 (Begg, PI)<br>**NIH/NCI**<br>Comparative Effectiveness Research Career Development Award<br>Role: Funded trainee	2011-2013
Seed Grant (Edmondson, PI)<br>**American Psychological Association**<br>Religious Struggle, PTSD, and Terror Management	2007-2008

Teaching Experience and Responsibilities

Instructor

2014	Course Instructor, P8112 Systematic Review and Metaanalysis, Columbia University Mailman School of Public Health
2013	Statistics Lectures, Epidemiology and Clinical Epidemiology Course, College of

Physicians and Surgeons, Columbia University (David Lederer and Steven Shea, Unit Directors)

2008	Structural Equation Modeling (with Tessa V. West), Yale University, graduate level
2006-2009	Theories of Personality, University of Connecticut, undergraduate; enrollment=100
	Abnormal Psychology, University of Connecticut, undergraduate; enrollment=100

Precepting and Invited Lectures

2012-2013	Epidemiology and Clinical Epidemiology Course, College of Physicians and Surgeons, Columbia University (David Lederer and Steven Shea, Unit Directors)
2012	Meta-analysis; N9653 Advanced Research Methods, Columbia University School of Nursing (Arlene Smaldone and Patricia Stone, Unit Directors) enrollment=30

Teaching Assistant

2008	Structural Equation Modeling (David Kenny, Instructor), University of Connecticut, graduate; enrollment= 45
2006-2009	Multivariate Statistics [Eric Lundquist (2 sections), Brian Connelly, Instructors], University of Connecticut, graduate; enrollment= 85
2004-2006	General Psychology I: Introductory, University of Connecticut; enrollment= 100
	Honors General Psychology II: Enhanced, University of Connecticut, undergraduate, enrollment= 100

Thesis/Dissertation Sponsorship

Student Name	Level of Learner	Date of Training	Nature of Responsibility
Laura Goorin	Ph.D.	2010	Dissertation Committee Member (TC)
Aurelie Athan	Ph.D.	2010	Dissertation Committee Member (TC)

Other Professional Activities

University Committees

Clinical/Epidemiological Research Task Force (Elizabeth Shane, leader)

Mailman School of Public Health student research practicum supervisor
2012- Safiya Richardson, M.D.: The influence of stress on cardiovascular events

BEST Diversity Program mentor; Biostatistics
2012- Introduce biostatistics to under-represented minority, economically disadvantaged, and disabled undergraduates, with special emphasis on cardiovascular and pulmonary research.

Co-editor (with Karina Davidson)

2012-*Progress in Cardiovascular Diseases* special issue: "Psychosocial factors and cardiovascular disease"

Ad hoc reviewer:

Ageing and Society	Annals of Behavioral Medicine
Anxiety, Stress, and Coping	Biological Psychiatry
Biological Psychology	Circulation
Cleveland Clinic Journal of Medicine	General Hospital Psychiatry
European Heart Journal	Health Psychology
International J for the Psych of Religion	Journal of Applied Social Psychology

Journal of Behavioral Medicine
Journal of Psychosomatic Research
J of Traumatic Stress
Personality and Individual Differences
Trials
J of Personality and Social Psychology
J of the American College of Cardiology
Metabolism
Psychosomatic Medicine
World Journal of Biological Psychiatry

Publications

A. Original, Peer Reviewed Articles

1. Wasson, L. T., Shaffer, J., Alcantara, C., Schwartz, J. E., & **Edmondson, D**. (2014). The association of posttraumatic stress disorder and quality of life during the first year after acute coronary syndrome. *International Journal of Cardiology.* Aug 13 [Epub ahead of print]
2. Alcántara, C., **Edmondson, D**., Moise, N., Oyola, D., Hiti, D., & Kronish, I. M. (2014). Anxiety Sensitivity and Medication Nonadherence in Patients with Uncontrolled Hypertension. *Journal of Psychosomatic Research, 77*(4), 283-286.
3. Davidson KW, Peacock J, Kronish IM, **Edmondson D**. (2014). Personalizing behavior change interventions through single-patient (N-of-1) randomized controlled trials. *Social and Personality Psychology Compass*, 8(8), 408-421.
4. **Edmondson D**, Green P, Ye S, Halazun H, Davidson KW (2014). Psychological stress and 30-day all-cause hospital readmission in acute coronary syndrome patients: An observational cohort study. *PLoS ONE*, 9(3): e91477. PMCID: PMC3951368
5. **Edmondson D,** Kronish IM, Wasson LT, Giglio J, Davidson KW, Whang W. (2014). A test of the diathesis-stress model in the emergency department: Who develops PTSD after an acute coronary syndrome? *Journal of Psychiatric Research,* 53:8-13. PMCID: PMC4023688 [Available on 2015/6/1].
6. Shaffer JA, Diaz K, Alcantara C, **Edmondson D,** Krupka DJ, Chaplin WF, Davidson KW. (2014). An inexpensive device for monitoring patients' weights via automated hovering. *International Journal of Cardiology,* 172(2): e263-4. PMCID: PMC3984000 [Available on 2015/3/15]
7. Shaffer JA, **Edmondson D,** Wasson LT, Falzon L, Homma K, Ezeokoli N, Li P, Davidson KW (2014). Vitamin D Supplementation for Depressive Symptoms: A Systematic Review and Meta-analysis of Randomized Controlled Trials. *Psychosomatic Medicine*, 2014, 76(3), 190-196. PMCID: PMC4008710 [Available on 2015/4/1]
8. Alcantara C, Peacock J, Hiti D, Davidson KW, **Edmondson D**. The association of short sleep after acute coronary syndrome with recurrent cardiac events and mortality. *International Journal of Cardiology*, 2014, 171(2):e11-2. PMCID: PMC3947905 [Available on 2015/2/1]
9. **Edmondson D.** (2014). An enduring somatic threat model of posttraumatic stress disorder due to acute life-threatening medical events. *Social and Personality Psychology Compass*, 8(3), 118-134. PMCID: PMC4048720 [Available on 2015/3/15]
10. Kronish IM, Lin JJ, Cohen BE, Voils CI, **Edmondson D.** (2014). Posttraumatic Stress Disorder increases risk for non-adherence to medications in patients with uncontrolled hypertension. *JAMA Internal Medicine*, 174(3), 468-470. PMCID: PMC3999520
11. **Edmondson, D.** & Cohen, B. (2013). Posttraumatic stress disorder and cardiovascular disease. *Progress in Cardiovascular Diseases*, 55, 548-56. PMCID: PMC3639489

12. Burg, MM, **Edmondson, D,** Whang, W, Shaffer, JA, Kronish, IM, Alacántara, C, Schwartz, JE, Muntner, P, Shimbo, D., Davidson, KW. (2013). The 'Perfect Storm' that leads to acute coronary syndrome: Do psychosocial factors play a role? *Progress in Cardiovascular Diseases*, 55, 601-610. PMCID:PMC3652628
13. **Edmondson, D.,** Gamboa, C., Cohen, A., Hyre Anderson, A., Kutner, N., Kronish, I. M., Mills, M.A. & Muntner, P. (2013). Association of posttraumatic stress disorder and depression with all-cause and cardiovascular disease mortality and hospitalization among Hurricane Katrina survivors with end-stage renal disease. *American Journal of Public Health,* 2013;103:e130-137. PMCID: PMC3673270
14. **Edmondson, D.**, Horowitz, C. R., Goldfinger, J. Z., Fei, K., Kronish, I M. (2013). Concerns about medications mediate the association of posttraumatic stress disorder with adherence to medication in stroke survivors. *British Journal of Health Psychology*, 18:799-813. PMCID: PMC3760991
15. **Edmondson D,** Kronish IM, Shaffer JA, Falzon L, Burg MM (2013). Posttraumatic stress disorder and risk for coronary heart disease: A meta-analytic review. *American Heart Journal*, *166*, 806-814. PMCID:PMC3815706 [Available on 2014/11/1]
16. **Edmondson, D**., Newman, J. D., Whang, W., & Davidson, K.W. (2013). Emotional triggers of acute myocardial infarction: Do they matter? *European Heart Journal, 34*, 300-6. PMCID:PMC3549526
17. **Edmondson D**, Richardson S, Fausett JK, Falzon L, Howard VJ, Kronish I. Prevalence of posttraumatic stress disorder in stroke survivors: A meta-analytic review. *PLoS ONE*. 2013;8:e66435. PMCID: PMC3686746
18. **Edmondson D,** Shaffer JA, Chaplin WF, Burg MM, Stone AA, Schwartz JE. Trait anxiety and trait anger measured by ecological momentary assessment and their correspondence with traditional trait questionnaires. *Journal of Research in Personality*, 2013;47:843-852. PMCID: PMC3815708
19. **Edmondson, D.,** Shimbo, D., Ye, S., Wyer, P., & Davidson, K.W. (2013). The association of emergency department crowding during treatment for acute coronary syndrome with subsequent posttraumatic stress disorder symptoms. *JAMA Internal Medicine,* 2013;173:472-475. PMCID: PMC3973030

    Editorial commentary: O'Malley PG. The environment of health care: Primum non nocere comment on "the association of emergency department crowding during treatment for acute coronary syndrome with subsequent posttraumatic stress disorder symptoms". *JAMA Internal Medicine,* 2013;173:474-474.

20. Goldfinger JZ, **Edmondson D,** Kronish IM, Fei K, Balakrishnan R, Tuhrim S, Horowitz CR (2013). Correlates of posttraumatic stress disorder in stroke survivors. *Journal of Stroke and Cerebrovascular Diseases,* Oct 19 [Epub ahead of print]. PMCID: PMC3992186 [Available on 2015/4/19]
21. Kronish IM, Diefenbach M, **Edmondson D**, Phillips LA, Fei K, Horowitz CR (2013). Key barriers to medication adherence in survivors of strokes and transient ischemic attacks. *Journal of General Internal Medicine*, *28*, 675-682. PMCID: PMC3631079
22. Shaffer JA, Kronish IM, Burg M, Clemow L, **Edmondson D** (2013). Association of acute coronary syndrome-induced posttraumatic stress disorder symptoms with self-reported sleep.

Annals of Behavioral Medicine, 46, 349-357. PMCID: PMC3800234 [Available on 2014/11/30].

23. **Edmondson, D**., Newman, J. D., Chang, M. J., Wyer, P., & Davidson, K.W. (2012). Depression is associated with longer emergency department length of stay in acute coronary syndrome patients. *BMC Emergency Medicine,* **12**:14 (5 November 2012). PMCID: PMC3546889.
24. **Edmondson, D**., Richardson, S., Falzon, L., Davidson, K.W., Mills, M.A., & Neria, Y. (2012). Posttraumatic stress disorder prevalence and risk of recurrence in acute coronary syndrome patients: A meta-analytic review. *PLoS ONE*. PMCID: PMCID: PMC3380054
25. **Edmondson D**, Shaffer JA, Denton EG, Shimbo D and Clemow L (2012). Posttraumatic stress and myocardial infarction risk perceptions in hospitalized acute coronary syndrome patients. *Frontiers in Psychology* 3:144. doi: 10.3389/fpsyg.2012.00144. PMCID: PMC3350942
26. Hale-Smith A, Park CL, **Edmondson D.** Measuring beliefs about suffering: Development of the views of suffering scale. *Psychol Assess*. 2012;24:855-866. PMCID: PMC3695743
27. Kronish, I. M., **Edmondson, D**., Li, Y., Cohen, B. (2012). Posttraumatic stress disorder and medication adherence: Results from the Mind Your Heart study. *Journal of Psychiatric Research*, *43*, 2192-7. PMCID: PMC3485414
28. Kronish, I., **Edmondson, D**., Goldfinger, J., & Horowitz, C. (2012). Posttraumatic stress disorder and adherence to medications in survivors of strokes and transient ischemic attacks. *Stroke*, *43*, 2192-7. PMC3404197
29. Kronish IM, Rieckmann N, Burg MM, **Edmondson D**, Schwartz JE, Davidson KW. (2012). The Effect of Enhanced Depression Care on Adherence to Risk Reducing Behaviors after Acute Coronary Syndromes: Findings from the COPES Trial. *American Heart Journal*, *164*, 524-9. PMCID:PMC3549526
30. Park, C.L., **Edmondson, D**., & Lee, J. (2012). Development of self-regulation abilities as predictors of psychological adjustment across the first year of college. *Journal of Adult Development*, *19*, 40-49.
31. Park, C.L., Mills, M. A., & **Edmondson, D**. (2012). PTSD as meaning violation: Testing a cognitive worldview perspective. *Psychological Trauma: Theory, Research, Practice, Policy, 4,* 66-73. PMCID: PMC4029350
32. Park, C. L., Sacco, S., & **Edmondson, D**. (2012). Expanding coping goodness of fit: Religious coping, health locus of control and depressed affect in heart failure patients. *Anxiety, Stress, and Coping, 25,* 137-53.
33. Richardson, S., Shaffer, J.A., Falzon, L., Davidson, K.W., Krupka, D., & **Edmondson, D**. (2012). Meta-analysis of perceived stress and its association with incident coronary heart disease. *American Journal of Cardiology*, 110, 1711-16. PMC3511594
34. Wortmann, J.H., Park, C.L., Swenson, E., & **Edmondson, D**. (2012). Spiritual struggle and adjustment to loss in college students: Moderation by denomination. *International Journal for the Psychology of Religion, 22*, 303-20.
35. **Edmondson, D.**, Chaudoir, S. C., Mills, M. A., Park, C. L., Holub, J., & Bartkowiak, J. M. (2011). From shattered assumptions to weakened worldviews: Trauma symptoms signal anxiety buffer disruption. *Journal of Loss and Trauma*, *16*, 358-85. PMCID: PMC3783359

36. **Edmondson, D.**, Rieckmann, N., Shaffer, J. A., Schwartz, J. E., Burg, M. M., Davidson, K. W., Clemow, L., Shimbo, D., & Kronish, I. M. (2011). Posttraumatic stress due to an acute coronary syndrome increases risk of 42-month major adverse cardiac events and all-cause mortality. *Journal of Psychiatric Research*, *45*, 1621-26. PMCID: PMC3210372
37. Ishikawa, J., Ishikawa, Y., **Edmondson, D**., Pickering, T. G., & Schwartz, J. E. (2011). Age and the difference in awake ambulatory blood pressure and clinic blood pressure: A meta-analysis. *Blood Pressure Monitoring*, *16*, 159-67. PMCID: PMC3839085
38. Newman JD, Muntner P, Shimbo D, Davidson KW, Shaffer JA, & **Edmondson D**. (2011). Post-traumatic stress disorder (PTSD) symptoms predict delay to hospital in patients with acute coronary syndrome. *PLoS One,* 2011, 6, e27640. PMCID: PMC3214073
39. Park, C. L., Wortmann, J.H., & **Edmondson, D**. (2011). Religious struggle as a predictor of subsequent mental and physical well-being in advanced heart failure patients. *Journal of Behavioral Medicine*, *34*, 426-36.
40. Shaffer, J. A., **Edmondson, D**., Chaplin, W. F., Schwartz, J. E., Iyer, P., Burg, M. M., Rieckmann, N., Shimbo, D., & Davidson, K. W. (2011). Directionality of the relationship between depression symptom dimensions and C-reactive protein among patients with acute coronary syndromes. *Psychosomatic Medicine, 73,* 370-77. PMCID: PMC3110525
41. Wortmann, J. H., Park, C. L., & **Edmondson, D.** (2011). Trauma and PTSD symptoms: Does spiritual struggle mediate the link? *Psychological Trauma: Theory, Research, Practice, Policy, 3,* 442-52. PMCID: PMC3269830
42. **Edmondson, D.,** Mills, M. A., & Park, C. L. (2010). Factor structure of the Acute Stress Disorder Scale in Hurricane Katrina evacuees. *Psychological Assessment, 22,* 269-278. PMCID: PMC2883796
43. **Edmondson, D**., & Park, C. L. (2009). Shifting foundations: Religious belief change and adjustment in college students. *Mental Health, Religion, and Culture, 12,* 289-302.
44. Park, C. L., **Edmondson, D.,** & Blank, T. O. (2009). Religious and non-religious pathways to stress-related growth in cancer survivors. *Applied Psychology: Health and Well-Being, 1,* 321-335.
45. Park, C. L., **Edmondson, D**., Blank, T. O., & Hale Smith, A. (2009). Religiousness/ spirituality and health behaviors in younger adult cancer survivors: Does faith promote a healthier lifestyle? *Journal of Behavioral Medicine*, *32*, 582-591
46. Penner, L. A., Dovidio, J. F., **Edmondson, D**., Albrecht, T. L., Dailey, R. K., & Markova, T. (2009). The experience of discrimination and Black-White health disparities in medical care. *Journal of Black Psychology, 35,* 180-203. PMCID: PMC3862356
47. Sullivan, T. P., Cavanaugh, C. E., Buckner, J. D., & **Edmondson, D**. (2009). Testing posttraumatic stress as a mediator of physical, sexual, and psychological intimate partner violence and substance problems among women. *Journal of Traumatic Stress, 22,* 575-584. PMCID: PMC3012603
48. Yanez, B., **Edmondson, D.**, Stanton, A. L., Park, C. L., Kwan, L., Ganz, P., & Blank, T. O. (2009). Facets of spirituality as predictors of adjustment to cancer: Relative contributions of having faith and finding meaning. *Journal of Consulting and Clinical Psychology, 77,* 730-741. PMCID: PMC2825181

49. **Edmondson, D**., Park, C. L., Chaudoir, S. R., & Wortmann, J. (2008). Death without God: Religious struggle, death concerns, and depression in the terminally ill. *Psychological Science, 19*, 628-632.
50. **Edmondson, D.,** Park, C. L., Blank, T. O., Fenster, J., & Mills, M. A. (2008). Deconstructing spiritual well-being: Existential well-being and HRQOL in cancer survivors. *Psycho-Oncology, 17*, 161-169.
51. Park, C. L., **Edmondson, D**., Blank, T. O., & Fenster, J. R. (2008). Meaning making and psychological adjustment following cancer: The mediating roles of growth, life meaning, and restored just world beliefs. *Journal of Consulting and Clinical Psychology, 76,* 863-875.
52. Park, C. L., **Edmondson, D.,** Fenster, J. R., & Blank, T. O. (2008). Positive and negative health behavior changes in cancer survivors: A stress and coping perspective. *Journal of Health Psychology, 13,* 1198-1206.
53. Mills, M. A., **Edmondson, D**., & Park, C. L. (2007). Trauma and stress response in Katrina's evacuees. *American Journal of Public Health*, *97,* S116-123. PMCID: PMC1854990

B. Case Reports

n/a

C. Reviews, Chapters and Editorials

1. **Edmondson D**, Davidson KW. Introduction to the special issue on psychosocial factors in cardiovascular disease. *Prog Cardiovasc Dis*. 2013; 55:509-510
2. Park, C. L., **Edmondson, D.,** & Hale Smith, A. Why religion? Meaning as the motivation. In K. I. Pargament (Ed.), APA Handbook of Psychology, Religion, and Spirituality. Washington, D.C., APA. 2013.
3. **Edmondson D**, Hiti D, Kronish IM. Posttraumatic stress disorder and cardiovascular disease. In *Cardiovascular Prevention: A Global Challenge*, Donna K. Arnett, ed. In press.
4. Park, C. L. & **Edmondson, D.** Religion as a source of meaning. In Mikulincer, M. & Shaver, P. (Eds.), Meaning, Mortality, and Choice: The Social Psychology of Existential Concerns. Washington, D.C., APA. 2012.
5. Park, C. L., **Edmondson, D.,** & Mills, M. A. Religious worldviews and stressful encounters: Reciprocal influence from a meaning making perspective. In T. W. Miller (Ed.), Handbook of stressful transitions across the lifespan. New York, Springer. 2009, 485-502.
6. **Edmondson, D**. & Mills, M. A. Research in New Orleans: Through the eyes of the victims of Hurricane Katrina. The Society for the Psychological Study of Social Issues: The Rookie. 2005, November.

D. Patents

n/a

E. Abstracts

1. Brondolo E, Wasson L, Alcantara C, **Edmondson D**. Posttraumatic stress disorder and smoking in year after an acute coronary syndrome event. Paper presented at American Psychosomatic Society, San Francisco, CA. March 2014.
2. Kronish I, Lin J, Cohen B, Voils C, Olamiju B, **Edmondson D**. PTSD and medication nonadherence in patients with uncontrolled hypertension. Paper presented at American Psychosomatic Society, San Francisco, CA. March 2014.
3. **Edmondson D**, Green P, Ye S, Halazun H, Davidson KW (2013). Psychological Stress And

30-day Readmission In Acute Coronary Syndrome Patients. Poster presented at the American Heart Association Scientific Sessions, Dallas.

4. **Edmondson D,** Richardson S, Fausett JK, Falzon L, Howard, VJ, Kronish, IK. (2013). Prevalence of PTSD in Stroke Survivors: A Meta-analytic Review. Poster presented at the American Heart Association Epidemiology and Prevention Scientific Sessions, New Orleans.
5. **Edmondson D,** Kronish, IK, Shaffer JA, Falzon L, Burg MM. (2013). Posttraumatic Stress Disorder and Risk for Coronary Heart Disease: A Meta-analytic Review of Prospective Studies Poster presented at the American Heart Association Epidemiology and Prevention Scientific Sessions, New Orleans.
6. Halazun H, Kronish I, **Edmondson D**, Davidson KW, Ye S (2013). The Impact of Patient Comorbidity on Risk of 30-day Readmission Following Hospitalization for Acute Coronary Syndromes. Poster presented at the American Heart Association Scientific Sessions, Dallas.
7. Happy, S., Morales, R., & **Edmondson, D.** (2013). Lifetime history of PTSD predicts nonadherence to medications after acute coronary syndrome. Poster presented at the 25th Annual Association for Psychological Science Convention, May 23-26, 2013.
8. Goldfinger JZ, Fei K, Balakrishnan R, Diefenbach MA, **Edmondson D**, Kronish IM, Tuhrim S, Horowitz CR. (2012, February). Prevalence and Correlates of PTSD After Stroke. Poster presented at the AHA/ASA International Stroke Conference, New Orleans, LA.
9. **Edmondson, D**., Newman, J., Chang, M., Wyer, P., & Davidson, K.W. (2012, April). Patients with suspected NSTEMI or unstable angina who are depressed wait longer in emergency departments than non-depressed patients. Poster presented at the 33rd Annual Meeting & Scientific Sessions of the Society of Behavioral Medicine, New Orleans.
10. **Edmondson, D**., Richardson, S., Falzon, L., Davidson, K. D., Mary Alice Mills, Ph.D., & Neria, Y. (2012, March). Acute coronary syndrome-induced posttraumatic stress disorder: A meta-analytic review of prevalence, association with clinical outcomes, and intervention effects. Poster presented at the Annual Meeting of the American Psychosomatic Society, Athens, Greece.
11. **Edmondson, D**., Ye, S., Schulman, J., & Davidson, K. D. (2012, March). Cost-effectiveness of enhanced depression treatment in acute coronary syndrome patients. Paper presented at the Annual Meeting of the American Psychosomatic Society, Athens, Greece.
12. **Edmondson, D**., Parsons, F., Shaffer, J. A., & Davidson, K. D. (2012, March). Wait, who are we missing? Demographic predictors of refusal to participate in a randomized controlled trial of depression treatment. Poster presented at the Annual Meeting of the American Psychosomatic Society, Athens, Greece.
13. **Edmondson, D**., Shaffer, J. A., Denton, E., Shimbo, D., & Clemow, L. P. (2011, March). PTSD and Recurrence Risk Perceptions in Acute Coronary Syndrome Patients. Paper presented at the Annual Meeting of the American Psychosomatic Society, San Antonio, TX.
14. Perez, S. A. (**student**), Duer-Hefel, J. T., Davidson, K. W., & **Edmondson, D**. (2011, November). Posttraumatic stress disorder symptoms and poor sleep quality in acute coronary syndrome patients. Paper presented at the American Heart Association Scientific Sessions, Orlando, FL.
15. Moran, M. D. (**student**), Duer-Hefel, J. T., Davidson, K. W., & **Edmondson, D**. (2011, November). Female patients with suspected NSTEMI or unstable angina wait longer in emergency department than male patients. Paper presented at the American Heart Association Scientific Sessions, Orlando, FL.
16. Shaffer, J. A., **Edmondson, D**., Chaplin, W., Schwartz, J. E., & Stone A. A. (2010, March).

Anxiety and anger in daily life: Contributions of the person and situation in momentary assessments of elicited states. Poster presented at the annual meeting of the American Psychosomatic Society, Portland.

17. **Edmondson, D**., Ishikawa, J., Yurgel, T., Burg, M. M., & Schwartz, J. E. (2010, March). Comparison of blood pressure assessment methods using CFA. Poster presented at the annual meeting of the American Psychosomatic Society, Portland.
18. Park, C. L. & **Edmondson, D**. (2010, May). Advances in the measurement of meaning making and meanings made. In C. L. Park (Chair), Innovations in meaning making research: Conceptual and methodological advances. Symposium conducted at the annual Association for Psychological Science Convention, Boston, MA.
19. **Edmondson, D**. & Schwartz, J. E. (2010, May). Potentially Traumatic Events Increase Resting Heart Rate in Civilians Assessed with ABPM. Poster presented at the annual meeting of the Association for Psychological Science, Boston.
20. Shaffer, J. A., **Edmondson, D**., Chaplin, W. F., Schwartz, J. E., Burg, M. M., Reickmann, N., & Davidson, K. W. (2010, August). Somatic and Cognitive Symptoms of Depression Predict Changes in C-Reactive Protein (CRP) in Patients with Acute Coronary Syndrome (ACS). Poster presented at the annual International Congress of Behavioral Medicine, Washington, D.C.
21. **Edmondson, D**., Kronish, I., Harlapur, M., Shaffer, J. A., Davidson, K. W., Schwartz, J. E., Burg, M. M., Iyer, P., Shimbo, D., Clemow, L., & Rieckmann, N. (2010, November). Posttraumatic Stress Disorder (PTSD) Symptoms Predict Worse Long-term Cardiovascular Prognosis in Post-Acute Coronary Syndrome (ACS) Patients. Paper presented at the American Heart Association Scientific Sessions, Chicago, IL.
22. **Edmondson, D**., Chaudoir, S. C., Bartkowiak, J., & Bestrom, P. (2009, February). Terror management and PTSD: Shattered assumptions or damaged worldview? Poster presented at the annual Society for Personality and Social Psychology Convention, Tampa, FL.
23. Park, C. L., **Edmondson, D**., & Blank, T. O. (2009, April). Religious and non-religious pathways to stress-related growth in younger adult cancer survivors. Paper presented at the 29th annual meeting of the Society of Behavioral Medicine, Montreal.
24. Mills, M. A., Park, C. L., & **Edmondson, D**. (2009, November). Shattered assumptions? A prospective study: The impact of trauma on global beliefs. Paper presented at the International Society for Traumatic Stress Studies, Atlanta.
25. Ishikawa, J., Ishikawa, Y., **Edmondson, D**., Schwartz, J. E., & Pickering, T. G. (2009, November). Age and the difference in awake ambulatory blood pressure and clinic blood pressure: A meta-analysis. Paper presented at the American Heart Association Scientific Sessions, Orlando.
26. **Edmondson, D**., Chaudoir, S. C., Holub, J., & Park, C. L. (2008, August). Weakened, but not shattered: Evidence of worldview dysfunction in PTSD. In D. Edmondson (Chair), The causes and psychological consequences of cultural worldview breach. Symposium conducted at the annual American Psychological Association Convention, Boston, MA.
27. **Edmondson, D**., Holub, J., Chaudoir, S. C., Gavitt, C., Bestrom, P., & Park, C. L. (2008, March). Religious struggle as adaptive meaning making in the service of terror management. In C. L. Park (Chair), Religious meaning systems in action: Stress appraisals, terror management, and restoration of global religious beliefs. Symposium conducted at the Annual Mid-Year Research Conference on Religion and Spirituality, Columbia, MD.

28. Sullivan, T. P., Cavanaugh, C. E., Buckner, J. D., & **Edmondson, D**. (2008, July). Intimate partner violence (IPV) and drug and alcohol use problems among community women: The roles of physical, sexual, and psychological IPV and PTSD. Paper presented at the International Family Violence and Child Victimization Research Conference, Portsmouth, New Hampshire.
29. Wortmann, J.H., Park, C.L., **Edmondson, D**., & Swenson, E. (2008, August). Denominational Differences in Religious Comfort and Struggle and Adjustment After Loss. Paper presented at the annual Convention of the American Psychological Association, Boston.
30. **Edmondson, D**., Park, C. L., Blank, T. O., & Smith, Hale Smith, A. (2008, August). Religion/Spirituality and Health Behaviors in Younger Adult Cancer Survivors. Poster presented at the annual American Psychological Association Convention, Boston, MA.
31. **Edmondson, D**., Park, C. L., & Blank, T. O. (2007, March). Change in existential well-being influences HRQOL in cancer survivors. Paper presented at the 28th annual meeting of the Society of Behavioral Medicine, Washington, DC.
32. **Edmondson, D**. (2007, March). Religious meaning influences the cognitive response to cancer, and vice versa. In M. Koltko-Rivera (Discussant), Trauma and spirituality: The recursive nature of spiritual meaning and meaning making. Symposium conducted at the annual Mid-Year Research Conference on Religion and Spirituality, Columbia, MD.
33. **Edmondson, D**., Park, C. L., Blank, T. O., & Fenster, J. R. (2007, August). The process and products of meaning making in cancer survivorship. Poster presented at the annual American Psychological Association Convention, San Francisco.
34. Mills, M. A., Park, C. L., & **Edmondson, D**. (2007, November). The immediate aftermath: Stress, coping, and distress in Hurricane Katrina's evacuees. Paper presented at the 23rd annual meeting of the International Society for Traumatic Stress Studies, Baltimore, MD.
35. Fenster, J., Park, C. L., **Edmondson, D**., & Blank, T. O. (2007, November). Relationship Between Previous Life Trauma, Resolution of Trauma, and Adjustment in Young Cancer Survivors. Poster presented at the International Society for Traumatic Stress Studies, Baltimore, MD.
36. **Edmondson, D**., Park, C. L., & Mills, M. A. (2006, March). A terror management model of religious engagement in older adults. In C. L. Park (Chair), Religion and death-related phenomena. Symposium conducted at the annual Mid-Year Research Conference on Religion and Spirituality, Columbia, MD.
37. **Edmondson, D**., & Mills, M. A. (2006, March). The role of religious coping in the cognitive processing of trauma. Paper presented at the annual Mid-Year Research Conference on Religion and Spirituality, Columbia, MD.
38. **Edmondson, D**., Park, C.L., Blank, T. O., Mills, M. A., & Fenster, J. (2006, March). Existential well-being and health related quality of life in cancer survivors. Paper presented at the 27th annual meeting of the Society of Behavioral Medicine, San Francisco.
39. Park, C. L., Blank, T. O., Fenster, J. R., & **Edmondson, D**. (2006, July). Spirituality, Meaning, and Well-being in Young to Middle Aged Cancer Survivors. Paper presented in Symposium, Meaning, Spirituality and Approaches to Decrease Distress (D. Pyritz, Chair) at the UICC World Cancer Congress. Washington, DC.
40. Park, C. L., Blank, T. O., Fenster, J. R., & **Edmondson, D**. (2006, July). Meaning Making Processes and Adjustment to Cancer Survivorship. Poster presented at the UICC World Cancer Congress, Washington, DC.

41. **Edmondson, D**., Wortmann, J., Ayotte, K., & Park, C. L. (2006, August). Model of religious struggle and depression in CHF (congestive heart failure) patients. Poster presented at the annual American Psychological Association Convention, New Orleans, LA.
42. **Edmondson, D**. (2006, August). Cancer survivorship as mortality salience: Terror management and wellbeing. Paper presented at the annual American Psychological Association Convention, New Orleans, LA.
43. **Edmondson, D**., & Park, C. L. (2005, April). Terror management perspectives on religion and adjustment to cancer. Paper presented in Symposium, Religion and Death-Related Adjustment (C. L. Park, Chair), at the Annual Midwinter Meeting of Division 36 (Psychology of Religion), American Psychological Association, Baltimore.
44. **Edmondson, D**., Parizeau, L., & Park, C. L. (2005, April). Religious and spiritual dimensions as determinants of health behaviors in college students. Paper presented at the annual Mid-Year Research Conference on Religion and Spirituality, Columbia, MD.
45. **Edmondson, D**., & Park, C. L. (2005, August). Religious belief change in college students. Paper presented at the American Psychological Association Convention, Washington, DC.

Invited Presentations

1. **Edmondson, D.** (2013, February). *PTSD and Cardiovascular Disease*. Invited presentation to New York University Department of Psychiatry.
2. **Edmondson, D.** (2011, April). *Religion and Meaning*. Presented at the Fourth Annual Herzliya Symposium on Social and Personality Psychology, Interdisciplinary Center, Herzliya, Israel.
3. **Edmondson, D**. (2010, April). *PTSD after acute coronary syndrome as an independent risk factor for MACE/ACM recurrence.* Presented at Division of General Medicine Spring Conference, Columbia University Medical Center, NY.
4. **Edmondson, D**. (2006, March). *Meaning and mortality in health research*. Presented at the University of Connecticut Center for Health Intervention and Prevention, "Sex, Drugs, and Health Behaviors II", Storrs, CT.
5. **Edmondson, D**., & Mills, M.A. (2006, March). *Acute Stress Disorder in Katrina's evacuees: Prevalence and predictors*. Presented at the University of Connecticut Economics Department Graduate Student Seminar series, Storrs, CT.

F. Media

2013

For: **Edmondson, D.,** Shimbo, D., Ye, S., Wyer, P., & Davidson, K.W. (2013). The association of emergency department crowding during treatment for acute coronary syndrome with subsequent posttraumatic stress disorder symptoms. *JAMA Intern Med*. 2013;173:472-475. NIHMS453931

1. **Chicago Tribune**

   Overcrowded ERs, PTSD signs tied in heart patients

   Being treated for a heart attack in a crowded emergency department may be linked to developing symptoms of a stress disorder, according to a new study.

For: **Edmondson D**, Richardson S, Fausett JK, Falzon L, Howard VJ, Kronish I. Prevalence of posttraumatic stress disorder in stroke survivors: A meta-analytic review. *PLoS ONE*. 2013;8:e66435

1. **CBS THIS MORNING**

PTSD can be triggered by serious medical events

June 29, 2013

Post-Traumatic Stress Disorder - PTSD - is often associated with military combat veterans, but a new study found that it can also be triggered by serious health events. **Donald Edmondson** and **Dr. Ian Kronish**, the co-authors of the study, talk to the "CBS This Morning: Saturday" co-hosts about their findings.

2. **WALL STREET JOURNAL LIVE**

PTSD: Not Just for Soldiers Anymore

July 24, 2013

A new study finds that one in four stroke patients are developing post traumatic stress disorder within a year of the stroke. And the odds of a psychological recovery are worse for younger patients. Sumathi Reddy and the study's lead author **Dr. Donald Edmondson** have details.

3. Also: **CNN; USA Today; LA Times; WCBS; Medscape; HHS Healthbeat**

2012

For **Edmondson, D**., Richardson, S., Falzon, L., Davidson, K.W., Mills, M.A., & Neria, Y. (2012). Posttraumatic stress disorder prevalence and risk of recurrence in acute coronary syndrome patients: A meta-analytic review. *PLoS ONE*. PMCID: PMC3380054

1. **JAMA**
Voelker R. Study: Acute coronary events linked with PTSD: JAMA. 2012 Jul 11;308(2):121.

2. **American Psychiatric Association**

Sinclair L. PTSD Often Overlooked in Treating Cardiac Patients. Psychiatric News. 2012;Sect. 22-5.

3. **NEW YORK TIMES**
Heart Attack Survivors May Develop P.T.S.D. By Tara Parker-Pope; June 20, 2012

4. **TIME**
Heart Attacks Can Trigger Post-Traumatic Stress By Amanda Macmillan**;** June 21, 2012

5. **ABC NEWS/ World News with Diane Sawyer**
   Heart Attacks and PTSD: A Vicious Cycle By Dr. Jenniffier Mahand; June 20, 2012

6. **FOX NEWS**
   PTSD prevalent among heart attack patients, study finds By Alex Crees; June 21, 2012

7. **CNN**
   PTSD strikes one in eight heart attack patients

8. **US News and World Report**
   Doheny, K. (2012), PTSD symptoms common after heart attack: Study. *U.S.News & World Report.*

9. **PBS NewsHour** for June 21, 2012

2011

For: **Edmondson, D.**, Rieckmann, N., Shaffer, J. A., Schwartz, J. E., Burg, M. M., Davidson, K. W., Clemow, L., Shimbo, D., & Kronish, I. M. (2011). Posttraumatic stress due to an acute coronary syndrome increases risk of 42-month major adverse cardiac events and all-cause mortality. *Journal of Psychiatric Research*, *45*, 1621-26. PMCID: PMC3210372

1. **Journal Watch Psychiatry**
   PTSD Affects Medical Outcomes After Acute Coronary Syndromes (2011, September 19). *Journal Watch Psychiatry*. http://psychiatry.jwatch.org.
   Internal Medicine News

2. **Internal Medicine News**
   PTSD Linked to Risk for Coronary Disease, Death (2010, December 10). *Internal Medicine News*. http://www.internalmedicinenews.com/news/mental-health/single-article/ptsd-linked-to-risk-for-coronary-disease-death/60996e5568.html.

2009

1. **UConn Magazine**
   Surviving the Ph.D. Process (2009, Fall/Winter). *UConn Magazine*
   http://uconnmagazine.uconn.edu/fwin2009/feature2.html.

Revised 5/1/2015

CURRICULUM VITAE
WEN-CHIH HANK WU, M.D., M.P.H.

**Business address**:	830 Chalkstone Ave, Providence, RI 02908
**Business Tel:**	(401) 273-7100 ext. 2426
**Business Fax:**	(401) 457-3311
**E-mail:**	**Wen-chih.wu@va.gov**<br>Wen-Chih_Wu@brown.edu

Education:

B.S., M.D.	University of Costa Rica School of Medicine<br>San Jose, Costa Rica, 1989-1995<br>Full-tuition Honor Scholarship 1990-1995<br>Honor Graduation 1995
M.P.H.	Brown University, June 2010
M.A.	Brown University, July 2010 (*ad eundem gradum*)

Postgraduate Training:

1996-1997	Internship, Primary Care Internal Medicine<br>University of Connecticut School of Medicine<br>Farmington, CT
1997-1999	Residency, Primary Care Internal Medicine<br>University of Connecticut School of Medicine<br>Farmington, CT
1999-2002	Fellowship, Cardiovascular Diseases<br>Brown University Medical School<br>Providence, RI
2008	Level 2 Training, Cardiac Computed Tomography Angiography; The Christ Hospital, Cincinnati, Ohio

Postgraduate Honors and Awards

1999	Medical Resident of the Year<br>University of Connecticut School of Medicine
2001-2003	Dean’s Teaching Excellence Awards<br>Brown Medical School
2002 – 2005	Teacher of the Year Award

	Cardiology Fellowship, Brown Medical School
2010	Beckwith Family Award for Outstanding Teaching<br>Dept. of Medicine, Alpert Medical School, Brown Univ.

Professional Licensure and Board Certification

1999-2011	Connecticut Medical License
1999-	ABIM- Internal Medicine
2001-	Rhode Island medical license
2002-	ABIM - Cardiology

Academic Appointments

2002 - 2010	Assistant Professor of Medicine (Cardiology)<br>Brown University Alpert Medical School
2010 –	Associate Professor of Medicine (Research),<br>Brown University Alpert Medical School
2012 -	Associate Professor of Epidemiology,<br>Brown University School of Public Health

Hospital Appointments

July, 2002-	Staff Cardiologist<br>Providence VA Medical Center
July 2004 - 2014	Medical Director, Cardiovascular Risk Reduction Clinic,<br>Providence VA Medical Center
July 2008 – July 2010	Medical Director, Heart Failure Clinic,<br>Providence VA Medical Center
July 2011 – September 2014	Medical Director, Intravenous Diuretic Clinic,<br>Providence VA Medical Center
July 2012 – current	Medical Director Shared-medical-appointment Clinics in Heart Failure, Providence VA Medical Center
March 2013 – current	Chief of Cardiology (Acting), Providence VA Medical Center
October 2014 -	Director of the Cardiovascular Wellness and Prevention Center at Miriam, Newport, and Rhode Island Hospitals

Other Appointments

1995-1996	General Practitioner<br>Orotina / San Mateo Division,<br>Ministry of Health of Costa Rica
2007-	Faculty, NHLBI R25HL088992-01, Short-Term Training Program to Increase Diversity in Health-Related Research
2008-2013	Faculty, NHLBI T32HL094300-01, Brown CardioPulmonary Research Training Program
2009-	Faculty, T35HL094308-01, Alpert Medical School Summer Research Program (AMSSRP) in Molecular Pathobiology and Health Services/Outcomes research in cardiopulmonary diseases
2012-	Core investigator, Providence VAMC Center of Innovation (COIN) for Long Term Support and Services
2012-2015	Core investigator, CHF QUERI, Palo Alto, CA
2012-2015	Core investigator, DM QUERI, Ann Arbor, MI

Editorial Positions

2008-2013	Section Editor, *Ferri's Clinical Advisor*, Ferri, FF [ed], Elsevier, St. Louis.
April 2014 - present	Academic Editor, PLoS One
April 2015 – present	Editorial Board: Alzheimer's & Dementia: Diagnosis, Assessment & Disease Monitoring (DADM) - a journal of the Alzheimer's Association

Ad Hoc Reviewer for *JAMA*

Ad Hoc Reviewer for *Chest*

Ad Hoc Reviewer for *American Journal of Medicine*

Ad Hoc Reviewer for *Cardiovascular Reviews and Reports*

Ad Hoc Reviewer for *Journal of the American Society of Echocardiography*

Ad Hoc Reviewer for *American Journal of Cardiology*

Ad Hoc Reviewer for *International Journal of Impotence Research*

Ad Hoc Reviewer for *Journal of Sexual Medicine*

Ad Hoc Reviewer for *Diabetes Care*

Study Section/Review Group

2008 Ad Hoc Reviewer for VA Cooperative Studies Program

2009 - 2010 NIH Special Emphasis Panel/Scientific Review Group 2009/10 ZRG1 HDM-P(58) R

2010 - present VA HSR&D Scientific Merit Review HSR1 Group

2010 – 2013 VA VISN 1 Career Development Award Program (V1CDA)

2011 – 2014 VA QUERI Rapid Response Projects review board

2012/08 Special Emphasis Panel/Scientific Review Group HQ2 R (CREATE review board)

2013 – 2015 VA Quality Enhancement Research Initiative (QUERI) Service Directed Project (SDP)

Proposal Review Section

Hospital Committees

2003-present	IRB Committee
2005-2010	Chair, Radiation Safety Committee
2006- 2010	Member, VA Advanced Clinic Access Committee
2006- 2010	Member, VA (VISN-1) Laboratory Utilization Council
2010-present	Member, Radiation Safety Committee
2012-present	Member, Education Leadership Committee

University Committees

2004 - 2007	Medical Faculty Executive Committee, Brown Univ.

Membership in Societies

1996-	Member, American College of Physicians
1999-	Member, American College of Cardiology
1999-	Member, American Heart Association
2003-	Interdisciplinary Working Group on Quality of Care & Outcomes Research, AHA
2005-	Fellow, American College of Cardiology

2007-	Member, Minority Members Committee, American Heart Association
2008-	Member, American Society of Preventive Cardiology
2008-	Member, Society of Cardiovascular Computed Tomography
2008-	Fellow, American Heart Association

Original Peer Reviewed Publications

1. **Wu WC**, Rathore SS, Wang Y, Radford MJ, Krumholz HM. Effectiveness of blood transfusion in elderly patients with acute myocardial infarction. *N Eng J Med* 2001; 345(17): 1230-6.

2. Shahi C, Rathore SS, Wang Y, Thakur R, **Wu WC**, Lewis JM, Petrillo MK, Radford MJ, Krumholz HM. Quality of care among elderly patients hospitalized with unstable Angina. *Am Heart J* 2001;142(2):263-70.

3. **Wu WC**, Tilkemeier PL. Significance of right ventricular visualization on stress sestamibi in a patient with dyspnea after atrial-septal-defect repair. *J Nucl Cardiol* 2002:9(2):236-7.

4. Sebastian CC, **Wu WC**, Shafer M, Choudhary G, Patel PM. Pneumopericardium and pneumothorax after permanent pacemaker implantation. *PACE* 2005; 28:1–3.

5. **Wu WC**, Sharma SC, Choudhary G, Coulter L, Coccio E, Eaton CB. Flow Mediated Vasodilation Predicts the Presence and Extent of Coronary Disease Assessed by Stress Thallium Imaging. *J Nucl Cardiol* 2005; 12(5):538-44.

6. Taveira TH, **Wu WC**, Martin OJ, Schleinitz MD, Friedmann P, Sharma SC. Pharmacist-led cardiac risk reduction model. *Prev Cardiol* 2006; 9(4):202-8.

7. Martin OJ, **Wu WC**, Taveira TH, Eaton CB, Sharma SC. Multidisciplinary Group Behavioral and Pharmacologic Intervention for Cardiac Risk Reduction in Diabetes-a Pilot Study. *Diabetes Educator* 2007; 33(1):118-27.

8. **Wu WC**, Schiffter TL, Henderson WG, Eaton CB, Poses RM, Uttley G, Sharma SC, Vezeridis M, Khuri SF, Friedmann PD. Levels of Preoperative Hematocrit Predict Postoperative Cardiovascular and Fatal Outcomes in Elderly Patients Undergoing Major Non-Cardiac Surgery. *J.A.M.A.* 2007; 297(22): 2481-8.*(selected publication submitted)

9. Pirraglia PA, Taveira TH, Cohen LB, **Wu WC**. The moderating effect of depression diagnosis on the effectiveness of a multi-factorial cardiovascular risk reduction clinic. *Preventing Chronic Disease* 2008; 5(4): A127 pp1-7

10. Khatana SAM, Taveira TH, Miner M, Eaton CB, **Wu WC.** Does Cardiovascular Risk Reduction Alleviate Erectile Dysfunction in Men with Type 2 Diabetes Mellitus? Int J Impot Res. 2008; 20(5): 501-6.

11. Taveira TH, Pirraglia PA, Cohen LB, **Wu WC.** Efficacy of a Pharmacist-led Cardiovascular Risk Reduction Clinic for Diabetic Patients with and without Mental Health Conditions. *Prev Cardiol* 2008; 11(4):195-200.

12. Khatana SA, Taveira TH, Choudhary G, Eaton CB, **Wu WC**. Change in Hemoglobin A1C and C-Reactive Protein Levels in Patients with Diabetes Mellitus. *J Cardiometab Synd* 2009;4:1-5.

13. Pirraglia PA, Taveira TH, Cohen LB, Dooley A, **Wu WC**. Maintenance of Cardiovascular Risk Goals in Veterans with Diabetes after Discharge from a Cardiovascular Risk Reduction Clinic. *Prev Cardiol* 2009;12(1):3-8.

14. Taveira TH, Friedmann PD, Cohen LB, Dooley AG, Khatana SAM, Pirraglia PA, **Wu WC**. Pharmacist-led Group Medical Appointment Model in Type 2 Diabetes. *Diabetes Educator* 2009; Dec 4 [Epub ahead of print], doi:10.1177/0145721709352383.

15. Choudhary G, Shin V, Punjani S, Ritter N, Sharma SC, **Wu WC.** The Role of Calcium Score and CT Angiography in the medical management of patients with normal myocardial perfusion imaging. J Nuc Cardiol 2009; Nov 21[Epub ahead of print]. doi:10.1007/s12350-009-9158-x.

16. Chen CK, Tseng VL, **Wu WC**, Greenberg PB. A Survey of the Current Role of Manual Extracapsular Cataract Extraction. Journal of Cataract and Refractive Surgery 2010; 36(4):692-3.

17. **Wu WC**, Smith TS, Henderson WG, Eaton CB, Poses RM, Uttley G, Mor V, Sharma SC, Vezeridis M, Khuri SF, Friedmann PD. Operative Blood Loss, Blood Transfusion and 30-day Mortality in Older Patients after Major Noncardiac Surgery. Annals of Surgery 2010 252(1):11-7.

18. Chen CK, Tseng VL, **Wu WC**, Scott IU, Greenberg PB. A Survey on the Use of General Anesthesia for Cataract Surgery. Graefes Arch Clin Exp Ophthalmol. 2010;248(7):1051-2.

19. Jankowich MD, Taveira TH, **Wu WC.** Decreased lung function is associated with increased arterial stiffness as measured by peripheral pulse pressure: data from NHANES III. Am J Hypertension 2010 23(6):614-9.

20. Cohen LB, Taveira TH, **Wu WC**, Pirraglia PA. Maintenance of risk factor control in patients with diabetes after discharge from a cardiovascular risk reduction clinic in patients with and without mental health conditions. Annals of Pharmacotherapy 2010; [Epub ahead of print], DOI 10.1345/aph.1P034.

21. Greenberg PB, Tseng VL, **Wu WC**, Liu J, Jiang L, Chen CK, Scott IU, Friedmann PD. Prevalence and Predictors of Ocular Complications Associated with Cataract Surgery in United States Veterans. Ophthalmology 2010 Oct 28. [Epub ahead of print].

22. Greenberg PB, Liu J, **Wu WC**, Jiang L, Tseng VL, Scott IU, Friedmann PD. Predictors of Mortality Within 90 Days of Cataract Surgery. Ophthalmology 2010 117(10):1894-9.

23. Khatana SAM, Taveira TH, Dooley AG, **Wu WC.** The Association between C-Reactive Protein Levels and Insulin Therapy in Obese vs. Non-Obese Veterans with Type 2 Diabetes Mellitus. *J Clin Hypertens* 2010;12(6):462-8.

24. Chen CK, Tseng VL, **Wu WC**, Scott IU, Greenberg PB. A Survey of the Management of Antithrombotic Therapy in Cataract Surgery Patients. J Cataract Refract Surg. 2010;36(7):1239-40.

25. Choudhary G, Atalay MK, Ritter N, Shin V, Grand D, Pearson C, Kirchner RM, **Wu WC.** Interobserver Reliability In The Assessment Of Coronary Stenoses By Multi-Detector Computed Tomography. J Comput Assist Tomogr 2011;35: 126-134.

26. Tseng VL, Greenberg PB, **Wu WC**, Jiang L, Li E, Kang JM, Scott IU, Friedmann PD. Cataract Surgery Complications in Nonagenarians. Ophthalmology. 2011 Mar 7. [Epub ahead of print]

27. Punjani S, **Wu WC**, Cohen S, Sharma SC, Choudhary G. Echocardiographic indices of diastolic function relate to functional capacity and quality of life in ambulatory men with atrial fibrillation. Journal of the American Society of Echocardiography 2011;24(5): 533-540.

28. Greenberg PB, Tseng VL, Jiang L, **Wu WC**. Cataract Surgery in the very ill. J Cataract Refract Surg 2011, 37(5):970-1.

29. **Wu WC**, Waring ME, Lessard D, Yarzebski J, Gore J, Goldberg RJ. 6-Month Mortality and Cardiac Catheterization in Non-ST Elevation Myocardial Infarction Patients with Anemia. Coronary Artery Disease 2011, 22:317–323.

30. Khatana SAM, Kane J, Taveira TH, Bauer MS, **Wu WC**. Monitoring and Prevalence Rates of Metabolic Syndrome in Veterans with Major Mental Illness. PLOS One 2011, 6(4): e19298.

31. Jankowich M**,** Choudhary G, Taveira TH, **Wu WC**. Age-, Race-, and Gender-Specific Prevalence of Diabetes among Smokers. Diabetes Research and Clinical Practice 2011, doi:10.1016/j.diabres.2011.05.029 (Epub ahead of print).

32. Taveira TH, Dooley AG, Cohen LB, Khatana SAM, **Wu WC**. Pharmacist-led Group Medical Appointments for the Management of Type 2 Diabetes with Co-Morbid Depression in Older Adults. Annals of Pharmacotherapy 2011; DOI: 10.1345/aph.1Q212 (Epub ahead of print).

33. Cohen LB, Taveira TH, Khatana SAM, Dooley AG, Pirraglia PA, **Wu WC**. Pharmacist-led Shared Medical Appointments for Multiple Cardiovascular Risk Reduction in Patients with Type 2 Diabetes. Diabetes Educator 2011; 37(6): 785-95. **DOI 10.1177/0145721711423980**

34. Malandrino N, **Wu WC**, Taveira TH, Whitlatch H, Smith RJ. Association between Red Blood Cell Distribution Width and Macrovascular and Microvascular Complications in Diabetes. Diabetologia 2011; 55(1):226-35.

35. **Wu WC**, Trivedi A, Friedmann PD, Henderson WG, Smith TS, Poses RM, Uttley G, Vezeridis M, Eaton CB, Mor V. Association between Hospital Intraoperative Blood Transfusion Practices for Surgical Blood Loss and Hospital Surgical Mortality Rates. Ann Surg. 2012 Feb 24. [Epub ahead of print]

36. Pirraglia PA, Rowland E, **Wu WC**, Taveira TH, Cohen LB, Friedmann PD, O'Toole TP. The benefits of a primary care clinic co-located and integrated in the mental health setting for veterans with serious mental illness. Preventing Chronic Disease 2012;9:E51. [Epub Feb 2]

37. Li E, Greenberg PB, Tseng V, Caffrey AR, **Wu WC**, Friedmann PD, LaPlante KL. In vitro coagulation effects of ophthalmic doses of bevacizumab. Journal of Ocular Pharmacology 2012, Feb 3. [Epub ahead of print].

38. Kapoor M, Schleinitz MD, Gemignani A, **Wu WC.** Outcomes of Patients with Chronic Heart Failure and Iron Deficiency Treated with Intravenous Iron: A Meta-analysis. Cardiovasc Hematol Disord Drug Targets. 2012 Sep 27. [Epub ahead of print]

39. Patel N, Taveira TH, Choudhary C, **Wu WC**. C-peptide Levels Predict Cardiovascular Mortality In Nondiabetic Adults. J Am Heart Assoc. 2012;1:e003152

40. Cohen S, Gaddam S, Gemignani A, **Wu WC**, Sharma S, Choudhary G. Right Ventricular Function Relates To Functional Capacity In Men With Atrial Fibrillation And Preserved Left Ventricular Ejection Fraction. Echocardiography 2013;0:1-9.

41. Kim J, Gaddam S, **Wu WC**, Behera V, Sharma S, Choudhary G. Stratified Reporting of High Sensitivity Troponin I Assay is Associated with Suboptimal Management of Patients with Acute Coronary Syndrome and Intermediate Troponin Elevation. Journal of Clinical Laboratory Analysis 2013 Sep;27(5):402-6. doi: 10.1002/jcla.21618. PubMed PMID: 24038227.

42. Sathya B, Davis R, Taveira T, Whitlatch H, **Wu WC**. Intensity of Peri-operative Glycemic Control and Postoperative Outcomes in Patients with Diabetes: A Meta-analysis. Diabetes Research and Clinical Practice 102 (2013), pp. 8-15. DOI information: 10.1016/j.diabres.2013.05.003

43. Chatterjee S, Kim J, Dahhan A, Choudhary G, Sharma S, **Wu WC**. Use of High-Sensitivity Troponin Assays Predicts Mortality in Patients with Normal Conventional Troponin Assays on Admission - Insights from a Meta-Analysis. Clinical Cardiology 2013. doi: 10.1002/clc.22196. [Epub ahead of print] PubMed PMID:24037966.

44. Khatana SAM, Jiang L, **Wu WC**. A comprehensive analysis of multidisciplinary management of dyslipidemia in a large health care system. Journal of Evaluation in Clinical Practice 2013. doi:10.1111/jep.12082. [Epub ahead of print] PubMed PMID: 24118549.

45. Sardar P; Chatterjee S; **Wu WC**; Lichstein E; Ghosh J; Aikat S; Mukherjee D. New Oral Anticoagulants are not Superior to Warfarin in Secondary Prevention of Stroke or Transient Ischemic Attacks, but Lower Risk of Intracranial Bleeding: Insights From a Meta- Analysis and Indirect Treatment Comparisons. PLoS ONE 2013; 8(10): e77694. doi:10.1371/journal.pone.0077694.

46. Taveira TH; **Wu WC;** Tschibelu E; Borsook D; Simonson D; Yamamoto R; Langleben DD; Swift R; Elman I. Naltrexone decreases body fat mass in olanzapine-treated schizophrenic or schizoaffective patients: a randomized double-blind placebo-controlled study. Journal of Psychopharmacology 2013. Nov 11. [Epub ahead of print] PMID: 24218048

47. Choudhary G, Jankowich M, **Wu WC.** Prevalence And Clinical Characteristics Associated With Pulmonary Hypertension In African-Americans. PLoS One. 2013;8(12):e84264. doi: 10.1371/journal.pone.0084264. PMID: 24358351.

48. Kirsh S, **Wu WC,** Edelman D, Aron DC. Research versus Quality Improvement: Distinct or a Distinction without a Difference? The Joint Commission Journal on Quality and Patient Safety 2014; 40(8): 365-75.

49. Taveira TH, **Wu WC**. Interventions to maintain cardiac risk control after discharge from a cardiovascular risk reduction clinic: A randomized controlled trial. Diabetes Res Clin Pract (2014), http://dx.doi.org/10.1016/j.diabres.2014.05.013 PMID: 24969964

51. Choudhary G, Jankowich M, **Wu WC**. Elevated Pulmonary Artery Systolic Pressure Predicts Heart Failure Admissions in African Americans: Jackson Heart Study. Circ Heart Fail. 2014 Jun 5. pii: CIRCHEARTFAILURE.114.001366. [Epub ahead of print]. PMID: 24902739

52. **Wu WC**, Jiang L, Friedmann PD, Trivedi A. Association Between Process Quality Measures for Heart Failure and Mortality Among U.S. Veterans. Am Heart J 2014; 168(5): 713–720.e3.

53. Greenberg PB, Chen AJ, **Wu WC**. Sudden vision loss and cardiovascular risk factors in African Americans: the Jackson Heart Study. Ophthalmic Epidemiol. 2015; 22(2):116-22. doi: 10.3109/09286586.2015.1012273. PubMed PMID: 25777311.

54. Jones LG, Sin M-K, Hage FG, Kheirbek RE, Morgan CJ, Zile MR, **Wu WC**, Deedwania P, Fonarow GC, Aronow WS, Prabhu SD, Fletcher RD, Ahmed A, Allman RM. Characteristics and Outcomes of Patients with Advanced Chronic Systolic Heart Failure Receiving Care at the Veterans Affairs vs. Other Hospitals: Insights from the BEST Trial. Circ Heart Fail. 2015; DOI: 10.1161/CIRCHEARTFAILURE.114.001300.

55. Chan KHK, Chacko SA, Song Y, Cho M, Eaton CB, **Wu WC**, Liu S. Genetic Variations in Magnesium-Related Ion Channels May Affect Diabetes Risk among African American and Hispanic American Women J. Nutr. 2015 jn.114.203489; published online January 7, 2015.

56. Steiner J, **Wu WC**, Jankowich M, Maron BA, Sharma S, Choudhary G. Echocardiographic Predictors of Mortality in Patients with Pulmonary Hypertension and Cardiopulmonary Comorbidities. PLoS ONE 03/2015; 10(3):e0119277. DOI:10.1371/journal.pone.0119277

57. Lin X, Zhang X, Guo J, Roberts CK, McKenzie S, **Wu WC,** Liu S, Song Y. Effects of Exercise Training on Cardiorespiratory Fitness and Biomarkers of Cardio-metabolic Health: A Systematic Review and Meta-analysis of Randomized Controlled Trials. J Am Heart Assoc. 2015 (4:e002014 doi: 10.1161/JAHA.115.002014)

58. Salmoirago-Blotcher E, Wayne P, Bock BC, Dunsiger S, **Wu WC**, Stabile L, Yeh G. Design and methods of the Gentle Cardiac Rehabilitation Study - A behavioral study of tai chi exercise for patients not attending cardiac rehabilitation. Contemporary clinical trials 06/2015; DOI:10.1016/j.cct.2015.06.020

59. Chen A, Trivedi AN, Jiang L, Vezeridis M, Henderson WG, **Wu WC**. Hospital Blood Transfusion Patterns During Major Noncardiac Surgery and Surgical Mortality. Medicine 2015; 94(32):e1342. doi: 10.1097/MD.0000000000001342.

60. Wellenius G, Kingsley S, Eliot M, Whitsel E, Wang Y, Coull B, Hou L, Margolis H, Margolis K, Mu L, **Wu WC,** Johnson K, Allison M, Manson J, Eaton C. Residential proximity to major roadways and incident hypertension in post-menopausal women. Environmental Research 2015; 142:522–528.

61. Santos CY, Lim YY, **Wu WC**, Polynice S, Maruff P, Snyder PJ. Resting-State Cardiac Workload is Related to Both Increased Neocortical Aggregation of β-Amyloid Protein and Relative Impairments in Spatial Working Memory in Pre-Clinical Alzheimer's Disease. Journal of Alzheimer's Disease 2015 (in press)

62. Lacy ME, Wellenius GA, Carnethon MR, Loucks EB, Carson AP, Luo X, Kiefe CI, Gjelsvik A, Gunderson EP, Eaton CB, **Wu WC**. Racial differences in the performance of existing risk prediction models for incident type 2 diabetes: The CARDIA study. Diabetes Care 2015 (in press).

63. Dev S, Lacy ME, Masoudi FA, **Wu WC.** Temporal Trends and Hospital Variation in Mineralocorticoid Receptor Antagonist Use in Veterans Discharged with Heart Failure. J Am Heart Assoc 2015 (in press)

<u>Other Peer Reviewed Publications</u>

1. Sadaniantz A, Wu WC. Survival of renal transplant patients after first myocardial infarction: a look to the past and promises for the future. *Am J Kidney Dis* 2000;36:211-3.

2. Rathore SS, Wu WC**,** Krumholz HM. Transfusion in elderly patients with myocardial infarction. Correspondence: authors reply to editor. *N Eng J Med 2002*;346(10):780-1.

3. Wu WC, **Ireland LA*,** Sadaniantz A. Evaluation of aortic valve disorders using stress echocardiography. *Echocardiography* 2004;21:451-8.

4. Sadaniantz A, Katz A, Wu WC. Miscellaneous use of stress echocardiography in patients with chronic pulmonary disease or congenital heart defect. *Echocardiography 2004;21:477-84.*

5. Wu WC, **Aziz GF*,** Sadaniantz A. Evaluation of aortic valve disorders using stress echocardiography. *Echocardiography* 2004;21:459-66.

6. Wu WC**, Bhavsar JH*,** Sadaniantz A. An overview of stress echocardiography in the study of patients with dilated and hypertropic cardiomyopathy. *Echocardiography* 2004;21:467-75.

7. Wu WC, Gordon PG. Invasive management of patients with ST elevation MI with >12 hour delay in presentation, the question remains unanswered. *Chest* 2004;126:2-4.

8. **Mehta SB*,** Wu WC. Management of coronary heart disease – stable angina, acute coronary syndrome, myocardial infarction. *Primary Care Clinics of North America* 2005; 32(4):1057-81.

9. **Patel PM*,** Wu WC. The electrocardiogram in the primary care office. *Primary Care Clinics of North America* 2005; 32(4):901-30.

10. Wu WC, Schiffter TL, Henderson WG. Preoperative Hematocrit Levels and Outcomes After Noncardiac Surgery J.A.M.A. 2007; 298(13): 1513-4.

11. **Karnik RS***, Wu WC. Self assessment in Cardiology. Common Tachyarrhythmias: Review Questions. *Hospital Physician* 2007; 43(11): 33-6.

12. **Regnante R***, Wu WC. Self-Assessment in Cardiology. Management of Chest Pain in the Emergency Department: Review Questions. *Hospital Physician* 2008; 44(11): 13-16.

13. Wu, WC. Does Blood Transfusion Increase Mortality Risk in Acute Myocardial Infarction? Am J Cardiol 2008; 102(8):1116-7 (Editorial letter).

14. **Leitner J***, Wu WC. Self-Assessment in Cardiology. Management of Aortic Valve Disease: Review Questions. *Hospital Physician*; November/December 2009: 33-7.

15. Greenberg PB, Tseng VL, **Wu WC**, Margo CE. Creating a Feedback Loop to Improve Cataract Surgery Outcomes. J Cataract Refract Surg 2011, Oct 22nd. [Epub ahead of print].

***Fellow/Resident/Trainee**

Books and Chapters

1. Salas I, Chan K, Wu WC. *Pain relief in cancer patients*. San Jose: Editorial Universidad de Costa Rica 1996. 79 pp. (Spanish).

2. **Aziz GA*,** Wu WC. Hypertension. In: *Primary Care for Women*. 2nd ed. Leppert PC, Peippert JF [ed], Lippincott, Williams, & Wilkins: Philadelphia, 2004.

3. **Mehta, SB*,** Wu, WC. Atrial myxoma. In: *Ferri's Clinical Advisor*, Ferri, FF [ed], Elsevier, St. Louis, 2007 (p.100).

4. **Mikaelian B*,** and Wu, WC. Carotid sinus syndrome. In: *Ferri's Clinical Advisor*, Ferri, FF [ed], Elsevier, St. Louis, 2007 (p. 158).

5. Wu, WC. Tricuspid Regurgitation. In: *Ferri's Clinical Advisor*, Ferri, FF [ed], Elsevier, St. Louis, 2006 (p. 860-861).

6. **Afshar M*,** and Wu, WC Tricuspid Stenosis. In: *Ferri's Clinical Advisor*, Ferri, FF [ed], Elsevier, St. Louis, 2007 (p. 913).

7. **Cho Y*,** and Wu, WC. Ventricular Septal Defect. In: *Ferri's Clinical Advisor*, Ferri, FF [ed], Elsevier, St. Louis, 2007 (pgs. 955-956).

8. **Patel PM*** and Wu, WC. Aneurysm, abdominal aorta. In: *Ferri's Clinical Advisor*, Ferri, FF [ed], Elsevier, St. Louis, 2006 (p. 59-60).

9. **Patel PM*** and Wu, WC. Cardiac tamponade. In: *Ferri's Clinical Advisor*, Ferri, FF [ed], Elsevier, St. Louis, 2006 (p. 155).

10. **Patel PM*** and Wu, WC. Cor pulmonale. In: *Ferri's Clinical Advisor*, Ferri, FF [ed], Elsevier, St. Louis, 2006 (p. 219-220).

11. **Mehta S*,** and Wu, WC. Tetralogy of Fallot. In: *Ferri's Clinical Advisor*, Ferri, FF [ed], Elsevier, St. Louis, 2007 (pgs. 880-881).

12. **Cho Y*,** Wu, WC. Peripheral Vascular Disease. In: *Ferri's Clinical Advisor*, Ferri, FF [ed], Elsevier, St. Louis, 2007 (pgs. 674-675).

13. Wu, WC. Temporomandibular Joint Syndrome. In: *Ferri's Clinical Advisor*, Ferri, FF [ed], Elsevier, St. Louis, 2009 (pg. 916).

14. **Gemignani, AS***; Ferri, FF; Wu, WC. Cardiomyopathy, hypertrophic. In: *Ferri's Clinical Advisor*, Ferri, FF [ed], Elsevier, St. Louis, 2011 (pg. 204-205).

15. **Gemignani, AS***; Ferri, FF; Wu, WC. Cardiomyopathy, dilated. In: *Ferri's Clinical Advisor*, Ferri, FF [ed], Elsevier, St. Louis, 2011 (pg. 202-203).

16. **Gemignani, AS***; Ferri, FF; Wu, WC. Cardiomyopathy, restrictive. In: *Ferri's Clinical Advisor*, Ferri, FF [ed], Elsevier, St. Louis, 2011 (pg. 206-207).

17. **Gemignani, AS***; Ferri, FF; Wu, WC. Mitral regurgitation. In: *Ferri's Clinical Advisor*, Ferri, FF [ed], Elsevier, St. Louis, 2011 (pg. 685-686).

18. **Pacheco, R***; Ferri, FF; Wu, WC. Mitral stenosis. In: *Ferri's Clinical Advisor*, Ferri, FF [ed], Elsevier, St. Louis, 2011 (pg. 687-688).

19. **Gemignani, AS***; Ferri, FF; Wu, WC. Mitral valve prolapse. In: *Ferri's Clinical Advisor*, Ferri, FF [ed], Elsevier, St. Louis, 2011 (pg. 689).

20. **Cohen S***; Ferri, FF; Wu, WC. Pericarditis. In: *Ferri's Clinical Advisor*, Ferri, FF [ed], Elsevier, St. Louis, 2011 (pg. 815-816).

21. **Punjani, S***; Ferri, FF; Wu, WC. Hyperosmolar hyperglycemic syndrome. In: *Ferri's Clinical Advisor*, Ferri, FF [ed], Elsevier, St. Louis, 2011 (pg. 542).

22. **Punjani, S***; Ferri, FF; Wu, WC. Diabetes mellitus. In: *Ferri's Clinical Advisor*, Ferri, FF [ed], Elsevier, St. Louis, 2011 (pg. 327-332).

23. **Punjani, S***; Ferri, FF; Wu, WC. Diabetic ketoacidosis. In: *Ferri's Clinical Advisor*, Ferri, FF [ed], Elsevier, St. Louis, 2011 (pg. 333-335).

24. **Punjani, S***; Wu, WC. Obesity. In: *Ferri's Clinical Advisor*, Ferri, FF [ed], Elsevier, St. Louis, 2011 (pg. 748-751).

25. **Punjani, S***; Ferri, FF; Wu, WC. Hypertension. In: *Ferri's Clinical Advisor*, Ferri, FF [ed], Elsevier, St. Louis, 2011 (pg. 548-551).

26. **Brancato, S***; Ferri, FF; Wu, WC. Heart block, second degree. In: *Ferri's Clinical Advisor*, Ferri, FF [ed], Elsevier, St. Louis, 2011 (pg. 472).

27. **Brancato, S***; Ferri, FF; Wu, WC. Multifocal atrial tachycardia. In: *Ferri's Clinical Advisor*, Ferri, FF [ed], Elsevier, St. Louis, 2011 (pg. 696).

28. Wu, WC; Ferri, FF. Aortic regurgitation. In: *Ferri's Clinical Advisor*, Ferri, FF [ed], Elsevier, St. Louis, 2011 (pg. 92).

29. **Earl, T***; Ferri, FF; Wu, WC. Heart block, complete. In: *Ferri's Clinical Advisor*, Ferri, FF [ed], Elsevier, St. Louis, 2011 (pg. 470-471).

30. **Brancato, S***; Ferri, FF; Wu, WC. Paroxysmal Supraventricular Tachycardia. In: *Ferri's Clinical Advisor*, Ferri, FF [ed], Elsevier, St. Louis, 2011 (pg. 800).

31. **Brancato, S***; Ferri, FF; Wu, WC. Sick sinus syndrome. In: *Ferri's Clinical Advisor*, Ferri, FF [ed], Elsevier, St. Louis, 2011 (pg. 1011).

32. **Cohen, S***; Ferri, FF; Wu, WC. Atrial septal defect. In: *Ferri's Clinical Advisor*, Ferri, FF [ed], Elsevier, St. Louis, 2011 (pg. 128-129).

33. **Earl, T***; Ferri, FF; Wu, WC. Atrial flutter. In: *Ferri's Clinical Advisor*, Ferri, FF [ed], Elsevier, St. Louis, 2011 (pg. 125-126).

34. **Earl, T***; Ferri, FF; Wu, WC. Atrial fibrillation. In: *Ferri's Clinical Advisor*, Ferri, FF [ed], Elsevier, St. Louis, 2011 (pg. 122-124).

35. **Cohen, S***; Ferri, FF; Wu, WC. Kawasaki disease. In: *Ferri's Clinical Advisor*, Ferri, FF [ed], Elsevier, St. Louis, 2011 (pg. 598-600).

36. **Pacheco, R***; Ferri, FF; Wu, WC. Radiation exposure. In: *Ferri's Clinical Advisor*, Ferri, FF [ed], Elsevier, St. Louis, 2011 (pg. 926-928).

37. **Earl, T***; Ferri, FF; Wu, WC. Long QT syndrome. In: *Ferri's Clinical Advisor*, Ferri, FF [ed], Elsevier, St. Louis, 2011 (pg. 628-629).

38. **Earl, T***; Ferri, FF; Wu, WC. Wolff-Parkinson-White. In: *Ferri's Clinical Advisor*, Ferri, FF [ed], Elsevier, St. Louis, 2011 (pg. 1192-1193).

39. **Sayeed, SM***; Ferri, FF; Wu, WC. Congestive heart failure. In: *Ferri's Clinical Advisor*, Ferri, FF [ed], Elsevier, St. Louis, 2011 (pg. 273-275).

40. **Silverstein, JR**; Ferri, FF; Wu, WC. Atrial Fibrillation. In *Ferri's Clinical Advisor* , FF [ed], Elsevier, St. Louis, 2012 (pg. 120-121).

41. **Silverstein, JR**; Ferri, FF; Wu, WC. Atrial Flutter. In *Ferri's Clinical Advisor* , FF [ed], Elsevier, St. Louis, 2012 (pg. 122-123).

42. **Pacheco, R**; Ferri, FF; Wu, WC. Atrial Myxoma. In *Ferri's Clinical Advisor* , FF [ed], Elsevier, St. Louis, 2012 (pg. 124).

43. **Cohen, S**; **Abdulbaki, A**; Ferri, FF; Wu, WC. Atrial Septal Defect, In *Ferri's Clinical Advisor* , FF [ed], Elsevier, St. Louis, 2012 (pg. 125-126).

44. **Lee, P**; Wu, WC. Brugada Syndrome, In *Ferri's Clinical Advisor* , FF [ed], Elsevier, St. Louis, 2012 (pg. 176-177).

45. **Lee, P**; Wu, WC. Cardiomyopathy, Stress-Induced, In *Ferri's Clinical Advisor* , FF [ed], Elsevier, St. Louis, 2012 (pg. 197)

46. **Brancato, S**; Wu, WC. Carotid Symdrome. In *Ferri's Clinical Advisor* , FF [ed], Elsevier, St. Louis, 2012 (pg. 198).

47. **Gemignani, A**; Ferri, FF; Wu, WC. Diabetes Mellitus, In *Ferri's Clinical Advisor* , FF [ed], Elsevier, St. Louis, 2012 (pg. 301-306).

48**. Gemignani, A**; Ferri, FF; Wu, WC. Diabetic Ketoacidosis, In *Ferri's Clinical Advisor* , FF [ed], Elsevier, St. Louis, 2012 (pg. 307-308).

49. **Earl, TJ**; Ferri, FF; Wu, WC. Heart Block, Complete, In *Ferri's Clinical Advisor* , FF [ed], Elsevier, St. Louis, 2012 (pg. 436).

50. **Silverstein, JR**; Ferri, FF; Wu, WC. Heart-Block, Second Degree, In *Ferri's Clinical Advisor* , FF [ed], Elsevier, St. Louis, 2012 (pg. 437).

51. **Kapoor, M;** Ferri, FF; Wu, WC. Heart Failure, In *Ferri's Clinical Advisor*, FF [ed], Elsevier, St. Louis, 2012 (pg. 438-441).

52. **Gemignani, A;** Ferri, FF; Wu, WC. Hyperosmolar, In *Ferri's Clinical Advisor* , FF [ed], Elsevier, St. Louis, 2012 (pg. 510).

53. **Gemignani, A**; Ferri, FF; Wu, WC. Hypertension, In *Ferri's Clinical Advisor* , FF [ed], Elsevier, St. Louis, 2012 (pg. 516-518)

54. **Abdulbaki, A**; Wu, WC. Kawasaki Disease, In *Ferri's Clinical Advisor* , FF [ed], Elsevier, St. Louis, 2012 (pg. 564-566).

55. **Silverstein, JR**; Ferri, FF; Wu, WC. Long QT Syndrome, In *Ferri's Clinical Advisor* , FF [ed], Elsevier, St. Louis, 2012 (pg. 596-597).

56. Wu, WC; Ferri, FF. Myocardial Infarction, In *Ferri's Clinical Advisor*, FF [ed], Elsevier, St. Louis, 2012 (pg. 675-678).

57. **Gemignani, A;** Ferri, FF; Wu, WC. Obesity, In *Ferri's Clinical Advisor* , FF [ed], Elsevier, St. Louis, 2012 (pg. 705-707)

58. **Truesdell, A;** Ferri, FF; Wu, WC. Sick Sinus Symdrome, In *Ferri's Clinical Advisor* , FF [ed], Elsevier, St. Louis, 2012 (pg. 936).

59. **Cohen, S**; Wu, WC.. Tetralogy of Fallot, In *Ferri's Clinical Advisor* , FF [ed], Elsevier, St. Louis, 2012 (pg. 1003-1004).

60. **Cohen, S; Abdulbaki, A**; Wu, WC. Ventricular Septal Defect, In *Ferri's Clinical Advisor* , FF [ed], Elsevier, St. Louis, 2012 (pg. 1077-1079).

61. **Silverstein, JR**; Ferri, FF; Wu, WC. Wolff-Parkinson-White Syndrome, In *Ferri's Clinical Advisor* , FF [ed], Elsevier, St. Louis, 2012 (pg. 1104-1005).

***Fellow/Resident/Trainee**

Abstracts

1. Lai T, Ma L, **Wu WC**, Knibbs D, Waters D, Chen C. Recovery of myocyte hypertrophy and slippage associated with the left ventricular remodeling of myocardial hybernation. J Am Coll Cardiol 1998;31:15A.

2. Lai T, Ma L, Linfert D, **Wu WC**, Olatidoye A, Gillam L, Waters D, Tsongalis G, Chen C. Reperfusion prevents apoptosis in hybernating myocardium. J Am Coll Cardiol 1998;31:394A.

3. Shahi C, Wang Y, **Wu WC**, Wang Y, Chen YT, Lewis JM, Krumholz HM, Radford MJ. Variability in quality of care for elderly patients admitted with unstable angina in Connecticut. J Am Coll Cardiol 1999; 33:337A.

4. Shahi C, Wang Y, **Wu WC**, Wang Y, Chen YT, Radford MJ, Krumholz HM. Prognostic significance of abnormal creatine kinase MB fraction with normal total creatine kinase in patients with acute coronary ischemic syndrome. J Am Coll Cardiol 1999; 33:394A.

5. Wright RM, **Wu WC**, Hamdan N, Fan A, Erickson A. Case report of carotid sinus hypersensitivity in a patient with head and neck cancer. 2000 Rhode Island Chapter of American College of Physicians Annual Meeting.

6. **Wu WC**, Cannistra AJ, Leavitt JI. Transient Brugada pattern in EKG mimicking anteroseptal myocardial infarction. 2002 Rhode Island Chapter of American College of Physicians Annual Meeting.

7. Taveira TH, Martin OJ, Petropoulos PN, Patel P, Sharma SC, **Wu WC**. Effectiveness of interdisciplinary cardiovascular risk reduction clinic for patients with coronary disease or diabetes. Circulation 2003; 107(19):e131,P4.

8. Martin OJ, **Wu WC**, Schleinitz MD, Taveira TH, Anderson ML, Patel PM, Donofrio G, Sharma S. Effectiveness of pharmacist-coordinated care to reduce cardiovascular risk factors in patients with diabetes mellitus. Circulation 2004; 109(7):e71,P203.

9. Uttley G., **Wu WC**, Freeman N, Sharma SC, Vezeridis M. The relationship of preoperative hematocrit levels with post-operative 30-day mortality in older veterans undergoing surgery. National Surgical Quality Improvement Program Meeting 2004.

10. **Wu WC**, Martin OJ, Taveira TH, Schleinitz MD, Mor V, Sharma SC. Durability of guideline adherence in coronary disease and diabetic patients after discharge from a cardiovascular risk reduction clinic. Circulation 2004; 109(20):e285, P41.

11. Patel PM, **Wu WC**, Cheng J, Martin OJ, Taveira TH, Sharma SC. Pattern of care of outpatients with coronary artery disease in a VA hospital. Circulation 2004;109(20):e231, P184.

12. Misra B, Gopalakrishnan G, Martin O, **Wu WC**, Taveira T, Anderson M, Sharma SC. Cardiovascular risk reduction programs for patients with diabetes. Circulation 2005;111(14): e209, P80.

13. **Wu WC**, Schifftner TL, Henderson WG, Eaton CB, Poses RM, Uttley G, Sharma SC, Vezeridis M, Khuri SF, Friedmann PD. Levels of Preoperative Hematocrit Predict Postoperative Cardiovascular and Fatal Outcomes in Elderly Patients Undergoing Major Non-Cardiac Surgery. VA HSR&D 2007 National Meeting Abstract. Washington, D.C., Feb 2007.

14. Pirraglia PA, Taveira TH, Cohen LB, **Wu W**. Depression diagnosis impact on the effectiveness of a multidisciplinary diabetes and cardiovascular risk reduction clinic. Journal of General Internal Medicine, 22(Supplement 1):17, 2007.

15. Taveira TH, Pirraglia PA, Cohen LB, **Wu WC**. Pharmacist-led Cardiac Risk Reduction Clinic Reduces Framingham Risk Score in Patients with Diabetes and Mental Illness. American Psychosomatic Society 65th Annual Scientific Meeting. Budapest, Hungary March 10, 2007. Psychosomatic Medicine 2007;69 (Supplement 1:1585) A-81.

16. Taveira TH, Cohen LB, Pirraglia PA, Friedmann PD, **Wu WC**. Pharmacist-led Group Medical Visits for Cardiovascular Risk Reduction in Diabetes. VA National HSRD 2008. National Meeting Abstract, Baltimore, MD, Feb 2008.

17. Khatana S, Taveira TH, Choudhary G, Eaton CB, **Wu WC**. Change In Hemoglobin A1c And C-reactive Protein Levels In Patients With Diabetes Mellitus American Heart Association 48th Cardiovascular Disease Epidemiology and Prevention and Nutrition, Physical Activity and Metabolism Conference. Colorado Springs, Colorado, March 11 –15, 2008.

18. Taveira TH, Cohen LB, Pirraglia PA, Friedmann PD, **Wu WC**. Pharmacist-led Group Medical Visits for Cardiovascular Risk Reduction in Diabetes. American Heart Association 48th Cardiovascular Disease Epidemiology and Prevention and Nutrition, Physical Activity and Metabolism Conference. Colorado Springs, Colorado, March 11 –15, 2008.

19. Cohen LB, Taveira TH, **Wu WC**, Pirraglia P. The Impact of Mental Health Conditions on Maintenance of Risk Factor Control in Diabetic Patients After Discharge from a Cardiovascular Risk Reduction Clinic. Academy Health 25th Annual Research Meeting. Marriott Wardman Park, Washington, DC. June 8-10, 2008.

20. **Wu WC**, Smith TS, Henderson WG, Eaton CB, Poses RM, Uttley G, Sharma SC, Vezeridis M, Khuri SF, Friedmann PD. Preoperative Hematocrit Is Not Predictive of CNS Events in Elderly Patients After Major Non-Cardiac Surgery. VA National QUERI Meeting 2008, Phoenix, AZ.

21. **Wu WC**, Lessard D, Goldberg RJ. Underuse of, and Mortality Associated With, Cardiac Catheterization in Patients with Acute Myocardial Infarction According to Anemia Status. VA National HSR&D meeting. Baltimore Marriott Waterfront, Baltimore, MD. Feb 10-12, 2009.

22. Prachanronarong K, Khatana SAM, Taveira TH, Cohen LB, Dooley AG, Pirraglia PA, Friedmann PD, **Wu WC.** Pharmacist-led Group Medical Visits for Multiple Cardiovascular Risk Reduction in Patients with Type 2 Diabetes. VA National HSR&D meeting. Baltimore Marriott Waterfront, Baltimore, MD. Feb 10-12, 2009.

23. **Wu WC,** Jiang L, Pirraglia P, O'Toole T, Friedmann P. Multidisciplinary Service Utilization and Cholesterol Guideline Implementation for Patients with Coronary Disease or Diabetes. AHA American Heart Association 49th Cardiovascular Disease Epidemiology and

Prevention and Nutrition, Physical Activity and Metabolism Conference. Circulation 2009; 119: e353, P322.

24. Taveira TH, Cohen LB, Dooley AG, Pirraglia PA, Friedmann PD, **Wu WC**. Pharmacist-Led Group Medical Visits for Diabetes in Patients with Comorbid Depression. AHA Joint Conference - Cardiovascular Disease Epidemiology and Prevention and Nutrition, Physical Activity, and Metabolism. Circulation 2009; 119: e359.

25. **Wu WC**, Parker D, Taveira TH, Choudhary G, Eaton C. Prevalence of Diabetes Among Adult Smokers: Need for Early Screening? AHA National Meeting 2009; Orlando, FLA.

26. **Wu WC**, Lessard D, Yarzebski J, Gore J, Goldberg RJ. Anemia and Invasive Management Utilization in Acute Non-ST Elevation Myocardial Infarction. AHA National Meeting 2009; Orlando, FLA.

27. Chen CK; Tseng VL; **Wu WC**; Greenberg PB. Current Role of Manual Extracapsular Cataract Extraction in Veterans Health Administration. American Society of Cataract and Refractive Surgery (ASCRS) 2010; Boston, MA.

28. Tseng VL; Greenberg PB; **Wu WC**; Jiang L; Scott IU; Friedmann PD. Cataract Surgery Complications in Nonagenarians. Joint Meeting of the American Academy of Ophthalmology and the Middle East Africa Council of Ophthalmology, October 16-19 2010; Chicago, IL.

29. Trivedi AN, Jiang L, Friedmann PD, **Wu WC.** Adherence to Quality Measures and Mortality among Veterans with Congestive Heart Failure. 2011 VA National HSRD meeting, Washington DC, February 2011.

30. Taveira TH, Dooley AG, Cohen LB, Khatana SAM, **Wu WC**. Effectiveness of Different Strategies in Maintaining Target Goals of Cardiovascular Risk Factors in Patients Discharged from a Cardiovascular Risk Reduction Clinic. AHA CVD Epidemiology and Prevention Scientific sessions 2011, March 14-16, San Diego, CA.

31. Khatana SAM, Kane, J, Taveira TH, Bauer MS, **Wu WC**. Monitoring and prevalence rates of metabolic syndrome in veterans with major mental illness. AHA CVD Epidemiology and Prevention Scientific sessions 2011, March 14-16, San Diego, CA.

32. Patel N, Taveira TH, Choudhary C, **Wu WC**. C-peptide Levels Predict Cardiovascular And Diabetes Related Mortality In Nondiabetic Adults, American Heart Association Scientific Sessions 2011, Orlando, FLA; November 15th, 2011.

33. Gemignani A, Kapoor M, Cohen S, Sharma SC, Choudhary G, **Wu WC**. Preoperative Renin-Angiotensin System Inhibitor Use is Associated with Worse Clinical Outcomes Following Cardiovascular Surgery: Results of a Meta-Analysis. American Heart Association Scientific Sessions 2011, Orlando, FLA; November 15th, 2011.

34. Jankowich M, Choudhary G, Klinger J, **Wu WC**. Relationship Between Lung Function And Albuminuria: Data From The Third National Health And Nutrition Examination Survey (NHANES III). 2012 ATS International Conference in San Francisco, California.

35. Grebla RC; Wellenius GA; Eaton CB; Gatsonis C, **Wu WC**. Beta-Blocker Monotherapy is Inferior to Treatment with Angiotensin-Converting-Enzyme Inhibitor/Angiotensin-Receptor

Blocker or Combined Therapy after Hospital Discharge among Patients with Acute Decompensated Heart Failure with Reduced Ejection Fraction. Circ Cardiovasc Qual Outcomes. 2012; 5: A99.

36. Lacy ME, Wellenius G, Eaton CB, Loucks EB, Correa A, Gjelsvik A, Wu WC. Examining the Gender-specific Effect of Adding Hemoglobin A1c to Existing Diabetes Risk Prediction Models: the Jackson Heart Study. American Diabetes Association 74th Scientific Sessions. San Francisco, CA: June 13-17, 2014.

37. Dev S, Lacy ME, Masoudi F, Wu WC. Patient Characteristics Associated with Appropriate Use of Aldosterone Antagonists in a National Cohort of Veterans Hospitalized with Heart Failure. Circ Cardiovasc Qual Outcomes. 2014;7:A158

Invited Presentations

Local

1. Outpatient Management of Chronic Stable Angina. RI Chapter of American College of Cardiology's Symposium: "Second Annual Cardiology for the Primary Care Provider", September 24, 2003; Warwick, RI

2. "BNP and the management of CHF." Medical Grand Rounds, Roger Williams Hospital, Boston University School of Medicine. March, 2005; Providence, RI.

3. "NOVEL RISK FACTORS OF CARDIOVASCULAR DISEASE", *Cardiovascular Disease and Cancer Behavioral Medicine Seminar Series*. The Miriam Hospital and Brown University. October 9, 2007; Providence, RI.

4. "Pathophysiology of Cardiovascular Diseases", RI Dept. of Health Cardiovascular disease educator certification course; October 7th, 2009, Warwick Sheraton Hotel, RI.

5. "Anemia and Transfusions in the ICU", Pulmonary Research Seminar, October 19th, 2009, Rhode Island Hospital.

6. "Outpatient management of CHF exacerbation", Primary Care Retreat, September 29th, 2010, White's of Westport, Westport, MA.

7. *"Group Medical Visits in Diabetes",* Center for Primary Care and Prevention Grand Rounds, March 15th, 2011; Memorial Hospital of Rhode Island.

8. *"Group Medical Visits in Diabetes",* Medical Grand Rounds -General Internal Medicine Update, March 22, 2011; Rhode Island Hospital.

9. *"Obesity, Insulin Resistance, Diabetes and Cardiovascular Disease",* Alpert Medical School of Brown University. Adult and Pediatric Endocrine Grand Rounds, October 17, 2012; Rhode Island Hospital.

10. Obesity, Insulin Resistance, Diabetes and Cardiovascular Disease, Alpert Medical School of Brown University. Cardiology Grand Rounds, May 9th, 2014; Rhode Island Hospital.

11. Magnesium Intake or Supplementation and Heart failure, Center for Primary Care and Prevention, Brown University. Grand Rounds, September 10th, 2014; Memorial Hospital of Rhode Island.

12. Cardiac Rehabilitation, Weight Control and Diabetes Research Center, Miriam hospital, September 29th, 2015, CARDIOVASCULAR BEHAVIORAL MEDICINE lecture series.

Regional

"Preoperative Risk Stratification for non-cardiac surgery", Cardiology Grand Rounds. Rhode Island Hospital and Lifespan. November 2nd, 2007.

"Radiation Exposure in Cardiac Imaging", at INVESTIGATION, INSTITUTIONAL COMMITTEES & INNOVATION Conference of the Massachusetts Medical Society, Waltham, MA. September 25, 2008

"Dyslipidemia in the Elderly, is it too late to help?", at 2009 Senior Symposium by the Connecticut Chapter of the American Society of Consultant Pharmacists, Ledyard, CT.

"Anemia and Transfusions in the ICU", at 2009 Update in Critical Care "Where are we now, and where are we going?" by Rhode Island Hospital, Providence, RI.

"Anemia and Acute Myocardial Infarction", Quality Scholars Conference. Louis Stokes Cleveland Dept. of Veterans Affairs Medical Center. May 21st, 2009.

"Pharmacist-Led Group Medical Appointments for Cardiac Risk Reduction in Diabetes", Medicine / Primary Care Noon Conference. Louis Stokes Cleveland Dept. of Veterans Affairs Medical Center. May 21st, 2009.

"Pharmacist-Led Group Medical Appointments for Cardiac Risk Reduction in Diabetes", Medicine / Primary Care Noon Conference. Louis Stokes Cleveland Dept. of Veterans Affairs Medical Center. May 21st, 2009.

"NOVEL RISK FACTORS OF CARDIOVASCULAR DISEASE", *Medicine Grand Rounds*, Honolulu VA Medical Center, February 24, 2010; Honolulu, HI.

"Interobserver Reliability in the Assessment of Coronary Stenoses by Multi-Detector Computed Tomography" West Roxbury VA Medical Center, October 22, 2010.

"Practical Approach to Group Visits or SMAs", VISN 7 (VHA) Shared Medical Appointments (SMA) Mini-Collaborative, October 25-26, 2011 in Duluth, GA.

"Status of SMA Implementation", VISN 7 (VHA) Shared Medical Appointments (SMA) Mini-Collaborative, April 24-25, 2012 in Duluth, GA.

Obesity, Insulin Resistance, Diabetes and Cardiovascular Disease. 44th Annual Samuel D. Plotkin Cardiovascular Symposium - April 28th, 2015, The Heart & Vascular Program at Baystate Medical Center, Springfield, MA.

National

1. Wu WC. Blood Transfusion Use in AMI. Society of Critical Care Medicine 33rd Critical Care Congress: "Transfusion Medicine Today – Blood Banking", February 23, 2004; Orlando, Florida.

2. **Wu WC,** Kirsh S, Edelman D. Workshop in "Inter-professional Diabetes Care: Research and Operational Issues of Group Appointments", 2008 VA HSR&D QUERI National Meeting, December 10-12, 2008; Phoenix, AZ.

3. Wu WC, Pascuzzi K, Taveira TH. "The Art & Science of Shared Medical Appointments: Lessons Learned", VA Employee Education System, May 31, 2011; National webcast.

4. **Wu WC**. "C-peptide Levels Predict Cardiovascular And Diabetes Related Mortality In Nondiabetic Adults", American Heart Association Scientific Sessions 2011, Orlando, FLA; November 15th, 2011.

5. **Wu WC**. "Adherence to Quality Measures and Mortality among Veterans with Congestive Heart Failure" CHF QUERI; March 14th, 2012; National webcast.

6. **Wu, WC**. "Group Medical Visits: System Redesign Practice to Enhance Patient Centered-Care" Specialty and Surgical Collaborative learning session; VA Employee Education System, September 21, 2012, National webcast

7. **Wu WC**. "Implementation of Transition of Care Model in CHF to Reduce Rehospitalization Rates" CHF QUERI; November 28th, 2012; National webcast.

8. **Wu WC**. "Group Visits" Specialty and Surgical Collaborative Practice Day (Learning Session #3), VA Employee Education System, January 9, 2014; VA National webcast

International

1. **Wu WC**. "Reducing hospitalization and improving outcomes in heart failure" Dept. of Emergency Medicine; National Taiwan University Hospital, August 18th, 2014.

Grants

2003-2005	Scios, Inc.	"The use of nesiritide for treatment of acute decompensated congestive heart failure complicated by acute renal insufficiency"<br>Wu, PI $20,000 Direct costs (no grant number)
2003- 2005	Merck, Inc.	"Effectiveness of different strategies in maintaining target of cardiovascular risk factors in patients discharged from cardiovascular risk reduction clinic"<br>Wu, PI $35,000 Direct costs (no grant number)
2004-2006	CVT-5131	"A Phase III, Randomized, Double-Blind Study of Intravenous CVT-3146 vs. Adenoscan® in Patients Undergoing Stress Myocardial

		Perfusion Imaging": Wu, site PI $20,000 Direct (no grant number)
2005	RI Foundation	Multidisciplinary Behavioral and Pharmacological Intervention for Cardiac Risk Reduction in Diabetes. Wu, PI Initiator $9,952 Direct costs (no grant number)
2005-2007	VA HSR&D	IIR- 04-313 "Variability in Surgical Blood transfusion Practices and Cardiovascular Outcome"; W. Wu, PI $ 171,850 Direct costs
2006-2007	ACCP	"Pharmacist-led Group Intervention for Diabetes and Psychiatric Illness": Role: Mentor, Co-I Taveira: trainee PI, $20,000 (no grant number)
2006-2008	ASHP	"Pharmacist-led Telehealth Disease Management in Patients with Diabetes and Depression": Role: Mentor, Co-I; Cohen, PI trainee), $25,000 (no grant number)
2006-2008	Auxilium, Inc.	"Effect of Testosterone on flow-mediated vasodilation, small artery elasticity index, insulin resistance, and erectile dysfunction in men with hypogonadism": Wu, Co-investigator (PI = Miner), $20,000 Direct. (no grant number)
2006-2008	Daugherty Foundation	"Multi-Targeted Cardiac Risk Intervention in Type 2 Diabetes", Wu, PI: $50,000 Direct costs (no grant number)
2007-2009	VA HSRD	VISN-1 Patient Safety Center of Inquiry, "Clinical Trial of a Home Safety Intervention for Alzheimer's Disease" (NRH 05-056 PI: Horvath), "PROVIDENCE VAMC CHF Clinic" substudy, Wu, co-I: $45,000 Direct costs
2008-2012	VA HSR&D	IAB – 06-269 "Group Intervention for DM Guideline Implementation", Wu, PI: $ 900,000 Direct costs
2009-2011	VA HSR&D	Supplement to IAB-06-269: Implementation of Honolulu VAMC in group intervention for DM and feasibility of video-conferencing diabetes group intervention in American Samoa, Wu, PI: $169,990.
2010-2011	VA QUERI - HSR&D	RRP 09-172 "Variations in Quality of Care and Outcomes for Veterans with Heart Failure", Wu, PI: $100,000 direct
2010-2013	Dept. Med, Brown Univ.	Chairman's developmental research grant program

		"Role of systemic vascular dysfunction in exercise tolerance rehabilitation in COPD" (Jankowich, PI, mentee): $50,000. Role: Co-I, Mentor.
2011	VA HSR&D	Supplement to IAB-06-269: Assessment of Patient Satisfaction in Group Intervention for DM, Wu, PI: $41,500
2011-2012	VA QUERI - HSR&D	RRP 10-229 "Medical Center Implementation of PCMH in Acute CHF to Reduce Rehospitalization Rates", Wu, PI: $100,000 direct
2012-2014	AHA Founder Affiliate	12CRP9840018 "Behavioral activation therapy for both depression and diabetes vs. diabetes alone delivered via group visits. Role: co-PI, mentor (Taveira, PI mentee); $75,000
2012-2013	RI Foundation	#20113648 "Effect of cardiovascular risk reduction on systemic vascular function in diabetics with COPD." Role: co-I, mentor (Jankowich, PI mentee); $13,790.
2012-2013	VA CHF QUERI	Heart Failure Shared-Medical-Appointment to provide early (<7 days) post-heart failure discharge follow-up. Role: PI; $24,800.
2013-2014	VA DM QUERI	RRP 12-452 "Video-Conference Shared Medical Appointments to Improve Rural Diabetes Care." Role: PI, $100,000
2013-2014	VA CHF QUERI	Pre-Implementation Study of Spironolactone Appropriateness and Safety Monitoring. Role: co-PI (Dev, PI); $100,000.
2013 – 2015	VA HSRD DM QUERI SDP	1 I01 HX000869-01A1 "Implementation Study of the Peer-to-Peer Program for Patients with Diabetes". Role: co-I & site PI (PI: Heisler) $335,000.
2013 – 2016	VA HSR&D	IIR 12-346 "Patient-experienced Integrated Care for Veterans with Multiple Chronic Conditions" Role: Co-I (PI: Meterko, M) $1,048,073
2014 – 2016	R34 AT 007569	Development of A Tai-Chi Program To Overcome Barriers To Cardiac Rehabilitation Role: Co-I (PI: Salmoirago-Blotcher)
4/2013 – 4/2015	VA V1CDA2012-19	Process Quality and Outcomes in Cataract Surgery Role: Mentor (Mentee: Paul Greenberg, MD – study PI) $245,000 (salary support).

6/2015 – 5/2017 1 F31 DK105791-01A1	The effect of sickle cell trait status on diabetes prediction in African Americans<br>Role: Sponsor (Mentee: Mary E. Lacy, PhD Candidate)
2015-2016 5R01CA155381-04	Quit for Health study: Role: Co-I<br>(PI: D Williams – Brown University, Subcontract 710-9852 TMH: S Dunsiger)
09/01/11-05/31/16 5 R01HL109116-04	"Efficacy of Exercise Videogames for Physical Activity Adoption and Maintenance"<br>(B. Bock, PI) $367,976<br>Role: Co-Investigator 0.6 Calendar
08/01/15 – 07/31/19 VA HSR&D IIR 14-293-2	"Group Medical Visits in Heart Failure for Post-Hospitalization Follow-Up" $275,000 per yr<br>Role: PI 3 CM

University Teaching Roles

July, 2002 - 2009	Section co-leader: BI/0281 - Pathophysiology course Brown Medical School
July, 2003 - 2009	Lecturer Bio 281 – "Pharmacologic management of CHF" The Warren Alpert Medical School of Brown University
July 2003 -	VA Site director, Fellowship Program in Cardiovascular Diseases at Brown University
July 2003 - 2008	Lecturer "EKG course" for Medical Students, Providence VA Medical Center, Alpert Medical School of Brown University
April 2008	Leader, Epidemiology for Medical students, small group sessions, Dept. of Community Health, Brown University
August 2008-	Program Director, Fellowship Program in Preventive Cardiology at Brown University
2010-2012	Member, Doctoral Thesis Committee, Dept. of Epidemiology, Brown University
2012-	Research Mentor: Doctoral Student, Dept. of Epidemiology, School of Public Health, Brown University

Hospital Teaching Roles

July, 2002-	Consult Service Attending in Cardiology and clinic preceptor, Brown Fellowship in Cardiovascular diseases

July, 2002-	Medical student inpatient preceptor,<br>Alpert Medical School, Brown University
July 2003 – present	Lecturer, EKG course Internal Medicine Residency<br>Program – Brown University
July 2005 – present	Lecturer, Preoperative cardiac evaluation,<br>Brown Fellowship in Cardiovascular diseases
July 2008 – present	Lecturer, Syncope and CV pharmacology for Internal<br>Medicine Residency Program – Brown University

Curriculum Vitae

Tao Liu, Ph.D.

Assistant Professor of Biostatistics (Research)
Brown University School of Public Health

Department of Biostatistics
Center for Statistical Sciences (CSS)
Center for AIDS Research (CFAR)
Alcohol Research Center on HIV (ARCH)
American College of Radiology Imaging Network (ACRIN)
The AMPATH Biostatistics and Data Program (AMPATH)

Tel: (401) 863-6480
Fax: (401) 863-9182
Email: tliu@stat.brown.edu
Campus Addr: Box GS121-7
Brown University
Providence, RI 201912, US

PROFESSIONAL APPOINTMENTS

A. Current Academic Positions

Assistant Professor of Biostatistics (Research)
Department of Biostatistics, Brown University School of Public Health.

Associate Director of the ARCH Data and Biostatistics Core
Brown Alcohol Research Center on HIV (ARCH).

Faculty Member
Brown Center for Statistical Sciences (CSS).
The Lifespan/Tuft/Brown Center for AIDS Research (CFAR).
The American College of Radiology Imaging Network (ACRIN).
Brown Alcohol Research Center on HIV (ARCH).

Affiliated Faculty Member
The AMPATH Biostatistics and Data Program (AMPATH), Kenya-USA.

B. Former Academic Appointments

Assistant Professor of Community Health (Research) (Sep. 2008 – Sep. 2012)
Biostatistics Division, Department of Community Health, the Alpert Medical School of Brown University.

Acting Co-Director of the CFAR Outcomes & Biostatistics Core (Jan. – Jun. 2010)
The Lifespan/Tuft University/Brown Center for AIDS Research (CFAR).

Postdoctoral Research Associate (Oct. 2006 – Aug. 2008)
Biostatistics Division, Department of Community Health, the Alpert Medical School of Brown University.

Graduate Teaching Assistant (Sep. 2003 – Oct. 2006)
Department of Biostatistics, Center for Clinical Epidemiology and Biostatistics (CCEB), the University of Pennsylvania.

Graduate Research Assistant / Consultant (May 2004 – May 2005)
Department of Biostatistics, Center for Clinical Epidemiology and Biostatistics (CCEB), the University of Pennsylvania.

Research Assistant / Graduate Instructor (Aug. 2001 – Jun. 2003)
Department of Statistics, Iowa State University.

Graduate Research Assistant (Aug. 1999 – Aug. 2001)
Department of Civil Engineering, Iowa State University.

Undergraduate Research Assistant (Aug. 1998 – May 1999)
Department of Environmental Science and Engineering, State Key Center of Environmental Stimulation and Pollution Control, Tsinghua University, Beijing, China.

C. Other Appointments

Research Fellow & Consultant (Jan. 2005 – Sep. 2006)
Research Statistics Unit, GlaxoSmithKline (GSK) Corp., Collegeville, PA

Summer Intern (Summer, 2003)
Department of Biostatistics, Amgen Incorporation, Thousand Oaks, CA

EDUCATION

2006 **Ph.D. Biostatistics** The University of Pennsylvania, PA, US

PhD Dissertation: Measuring sensitivity to nonignorable censoring in nonparametric and semiparametric survival modeling.

2002 **M.S. Statistics** Iowa State University, IA, US

Thesis: Improved Confidence Interval Estimation for Parameters in Nonlinear Models

2002 **M.S. Civil Engineering** Iowa State University, IA, US

Thesis: Ammonia Inhibition in Thermophilic Anaerobic Process

1999 **B.E. Environmental Engineering** Tsinghua University, Beijing, China

PUBLICATIONS

A. Book Chapter

1. Fedorov, V.V. and **Liu, T.*** (2007). "Enrichment Design." *Encyclopedia of Clinical Trials*. John Wiley & Son Press. (*Authorship in alphabetical order)

B. Journals Articles (Peer-reviewed)

1. **Liu, T.** and Hogan, J. (2015). "Inference about ATE from observational studies with continuous outcome and unmeasured confounding." Under revision for *Statistics in Medicine*.
2. Kahler, C., **Liu, T.**, Cioe, P., Bryant, V., Pinkston, M., Kojic, E., Onen, N., Baker, J., Hammer, J., Brook, J. and Patel, P. (2015). "Direct and Indirect Effects of Heavy Alcohol Use on HIV and non-HIV Outcomes in a Longitudinal Study of HIV Patients Receiving ART." Under review in *Journal of AIDS*.

3. **Liu, T.**, Bazerman, L., Pinkston, M., Nunn, A., Rana, A. and Beckwith, C. (2015). "Co-occurring HIV risk behaviors among males entering jail." Invited revision for *Journal of AIDS & Clinical Research*.
4. Merchant RC, Baird JR, **Liu T**, and Nirenberg TD (2015). "Short-term efficacy of a brief intervention to reduce drug misuse and increase drug treatment utilization among adult emergency department patients." *Academic Emergency Medicine* (Accepted).
5. Stillman, A.E., Gatsonis, C., Lima, J.A.C., Black, W.C., Cormack, J., Gareen, I., Hoffmann, U., **Liu, T.**, Mavromatic, K., Schnall, M.D., Udelson, J.E., Wood, P. (2015). "Rationale and Design of the RESCUE (Randomized Evaluation of Patients with Stable Angina Comparing Utilization of Noninvasive Examinations) Trial." Under review in *the American Heart Journal*.
6. Merchant RC, DeLong AK, **Liu T**, Baird JB (2015). "The impact of time elapsed participating in a study on a brief intervention aimed to increase uptake of rapid HIV and Hepatitis C screening among drug misusing adult Emergency Department patients." *AIDS and Behavior*. (in press).
7. Bernardino VL, Baird JR, **Liu T**, and Merchant RC (2015). "Comparison of substance use prevalence among Rhode Island and The Miriam Hospital Emergency Department patients to state and national general population prevalence estimates." *Rhode Island Medical Journal.* (In press).
8. Beaudoin F; Baird JR; **Liu T**; Merchant RC (2015). "Gender differences in substance use among adult emergency department patients: prevalence, severity, and need for intervention." *Academic Emergency Medicine*. (In press).
9. Shao W, Guan W*, Clark MA, **Liu T**, Santelices, C, Cortes, DE, and Merchant, RC (2015) "Does a video improve knowledge of HIV/AIDS and HIV testing among a global internet audience?" *International Journal of STD & AIDS*. (*student advised; Accepted).
10. Shao W, Guan W*, Clark MA, **Liu T**, Santelices C, Cortes DE, and Merchant RC (2015). "Variations in recruitment yield, costs, and speed and participant diversity across internet platforms in a global study of English- or Spanish-speaking Internet users.". Digital Culture & Education, 7(1), 40-86. (*student advised)
11. Merchant R.C., Clark, M.A., Santelices, C.A., **Liu, T.**, and Cortes, D. (2015). "Effectiveness of an HIV/AIDS and HIV testing informational video for Spanish-speaking Latinos in healthcare and non-healthcare settings". *AIDS and Behavior*. 19(3): 523—535. [PMID: 25179540].
12. Rana, A.I., **Liu, T.**, Gillani, F.S., Reece, R., Kojic, E.M., Zlotnick, C., and Wilson, I.B. (2014) "Multiple gaps in care common among newly diagnosed HIV patients." *AIDS Care*. 27(6): 679—687. [PMCID: PMC25634492] [PMCID: PMC4366312].
13. Tai, M.*, **Liu, T.**, and Merchant, R.C. (2014). "HIV/AIDS-related hospitalizations in the United States short-stay hospitals, 1982-2010." *Journal of the International Association of Providers of AIDS Care.* (*Advised student). (in press).
14. Merchant, R.C., Baird, J.R., **Liu, T.**, and Taylor, L.E. (2014). "HCV among the Miriam Hospital and Rhode Island Hospital adult emergency department patients." *Rhode Island Medical Journal,* 97(4): 35—39. [PMID: 24983020].
15. Merchant, R., Baird, J., **Liu, T.**, Taylor, L, Montague, B., and Nirenberg, T. (2014) "Brief intervention to increase emergency department uptake of combined rapid HIV and hepati-

tis C screening among a drug misusing population." *Academic Emergency Medicine*. 21:752—767. [PMID: 25125271] [PMCID: PMC4135533]

16. Federov, V. and **Liu, T.*** (2014). "Randomized discontinuation trials with binary outcomes." *The Journal of Statistical Theory and Practice,* 8:30—45. (*Authorship in alphabetical order)
17. **Liu, T.**, Hogan J.W., Wang, L., Zhang, S. and Kantor, R. (2013). "Optimal Allocation of Gold Standard Testing under Constrained Availability: Application to Assessment of HIV Treatment Failure." *Journal of the American Statistical Association (JASA)*, 108 (504): 1173—1188. [PMCID: PMC3963362].
18. Trillo, A., Merchant, R., Baird, J., Ladd, G., **Liu, T.**, and Nirenberg, T. (2013). "Interrelationship of alcohol misuse, HIV sexual risk and HIV screening uptake among emergency department patients." *BMC Emergency Medicine*, 13:9-22. [PMID: 23721108] [PMCID: PMC3686630]
19. Xu, H.*, Short, S.E., and **Liu, T.** (2013). "Dynamic relations between fast-food restaurant and body weight status: A longitudinal and multilevel analysis of Chinese adults." *Journal of Epidemiology & Community Health*, 67:271-279. [PMID: 22923769]. (*Advised student; Recipient of the 2009 Brown Global Health Scholarship).
20. **Liu, T.** and Heitjan, D.F. (2012). "Sensitivity of the discrete-time Kaplan-Meier estimate to non-ignorable censoring: Application in a clinical trial". *Statistics in Medicine*, 31: 2998-3010. [PMID: 22806989].
21. Trillo, A., Merchant, R.C., Baird, J.R., **Liu, T.**, and Nirenberg, T.D. (2012). "Sex differences in alcohol misuse and estimated blood alcohol concentrations among emergency department patients: Implications for brief interventions." *Academic Emergency Medicine*, 19(8): 924-33. [PMID: 22849748] [PMCID: PMC3424395].
22. Wei, F., Zhou, J., Wei, X., Zhang, J., Fleming, B.C., Terek, R., Pei, M., Chen, Q., **Liu, T.**, and Wei, L. (2012). "Activation of Indian Hedgehog promotes chondrocyte hypertrophy and upregulation of MMP-13 in human osteoarthritic cartilage." *Osteoarthritis and Cartilage*, 20(7):755-63. [PMID: 22469853] [PMCID: PMC3374008].
23. Anderson, A., Cindy, F., **Liu, T.**, Swarts, A., Siminya, M. and Cu-Uvin, S. (2012). "Effect of trichomoniasis therapy on genital HIV viral burden among African women." *Sexually Transmitted Diseases*. 39(8):638-642. [PMID: 22797689] [PMCID: PMC3398383].
24. Loucks, E.B., Gilman, S.E., Rogers, M., **Liu, T.**, Kawachi, I., Martins, L., Kubzansky, L., Buka, S.L. (2012). "Education and coronary heart disease risk associations may be affected by early life common prior causes: A propensity matching analysis." *Annals of Epidemiology,* 22(4):221-232. [PMID: 22463841] [PMCID: PMC3327475].
25. Merchant, R.C., Waxman, M.J., Maher, J.G., Clark, M.A., Celada, M.T., **Liu, T.**, Simmons, E.M., Beckwith, C.G. and Mayer, K.H. (2012). "Patient and clinician ethical perspectives on the 2006 Centers for Disease Control and Prevention HIV testing methods." *Public Health Reports,* 127(3):318- 329. [PMID: 22547863] [PMCID: PMC3314076].
26. Rosen (Asnis)*, L.M., **Liu, T**. and Merchant, R.C. (2012). "Efficiency of ICD-9 billing code searches to identify blood or body fluid exposure emergency department visits through a state-wide multi-center database." *Infection Control and Hospital Epidemiology*. 33(6): 581-588. [PMID: 22561713] (*Advised student)
27. Saffarini, C.M., Heger, N.E., Yamasaki, H., **Liu, T.**, Hall, S.J., and Boekelheide, K. (2012). "Induction and persistence of abnormal testicular cells following gestational exposure to di-

(n-butyl) phthalate in p53-null mice." *The Journal of Andrology*, 33(3):505-13. [PMID: 21868749].

28. Mabeya, H., Khozaim, K., **Liu, T.**, Orango, O., Chumba, D., Pisharodi, L., and Cu-Uvin, S. (2011). "Comparison of conventional cervical cytology versus visual inspection with acetic acid (VIA) among HIV-infected women in western Kenya." *Journal of Lower Genital Tract Disease*. 16(2): 92. [PMID: 22126834] [PMCID: PMC3289722].
29. Merchant, R.C., DePalo D.M., **Liu, T.**, Rich J.D., and Stein M.D. (2010). "Developing a system to predict laboratory-confirmed chlamydial and/or gonococcal urethritis in adult male emergency department patients." *Postgraduate Medicine*. 22(1): 52–60. [PMID: 20107289] [PMCID: PMC3021915].
30. Beckwith, C.G., **Liu, T.**, Bazerman, L.B., DeLong, A.K., Desjardins, S.F., Poshkus, M., Flanigan, T.P. (2010). "HIV risk behavior pre- and post-HIV testing in jail: A pilot study." J*ournal of Acquired Immune Deficiency Syndromes*, 53(4): 485–90. [PMID: 20035232] [PMCID: PMC2837107].
31. Merchant R.C., Lau T.S., **Liu, T.**, Mayer K.H., and Becker B.M. (2009). "Adult sexual assault evaluations at Rhode Island emergency departments, 1995-2001." *Journal of Urban Health*, 86(1): 43–53. [PMID: 18797994] [PMCID: PMC2629515].
32. Phelan, S., **Liu, T.**, Gorin, A., Lowe, M., Hogan, J., Fava, J., Wing, R. (2009). "What distinguishes long-term weight loss maintainers from the treatment-seeking obese? Analysis of environmental, behavioral, and psychosocial variables in diverse patient populations." *Annals of Behavioral Medicine*, 38: 94–104. [PMID: 19847584]
33. Moreira C., Venkatesh, K.K., DeLong A.K., **Liu, T.**, Kurpewski J., Ingersoll J., Caliendo A.M., Cu-Uvin S. (2009). "Effect of treatment of asymptomatic bacterial vaginosis on HIV-1 shedding in the genital tract among women on antiretroviral therapy: Pilot study." *Clinical Infectious Diseases*, 49: 991–992. [PMID: 19694541] [PMCID: PMC2730467].
34. Hogan, J.W. and **Liu, T.** (2008). "Mediation analysis for intervention trial: Objectives, models and inference." *Health Services and Outcomes Research Method*, 8: 77-79.
35. Anderson B.L., Wang C.C., **Liu, T.**, DeLong A.K., Kojic E.M., Kurpewski J., Ingersoll J., Mayer K., Caliendo A., and Cu-Uvin S. (2008). "Genital tract leukocytes and shedding of genital HIV-1 RNA." *Clinical Infectious Diseases*, 47(9): 1216–1221. [PMID: 18808359] [PMCID: PMC2714169].
36. Merchant, R.C., Chee, K.J., **Liu, T.**, Mayer, K.H., and Becker, B.M. (2008). "Incidence of visits for health care worker blood and body fluid exposures and HIV post-exposure prophylaxis provision at Rhode Island Emergency Departments." *Journal of AIDS*, 47: 358–368. [PMID: 18176321] [PMCID: PMC3180865].
37. Sung, S. and **Liu, T.** (2003). "Ammonia inhibition on thermophilic methanogens." *Chemosphere*, 53(1):43-52. [PMID: 12892665].
38. **Liu, T.** and Sung, S. (2002). "Ammonia inhibition on thermophilic aceticlastic methanogens." *Water Science and Technology*, 45(10):113–120. [PMID: 12188530].
39. **Liu, T.** and Sung, S. (2001). "Effect of ammonia inhibition in thermophilic anaerobic process." *Proceedings of the Water Environment Federation*, 2001(1): 1292-1306.

C. Non-Refereed Articles

38. **Liu, T.**, DeLong, A., and Blossom, B. (2009) "Challenge to IDSA recommendations for late neurologic Lyme disease treatment and post Lyme syndrome: A statistical review of NIH-funded treatment studies." The International Lyme And Associated Diseases Society (IDSA) Lyme Panel Review Hearing (http://www.ilads.org/lyme_disease/written_testimony/12%20Liu%20DeLong%20Blossom%20-%20Statistical%20Review.pdf)
39. **Liu, T.** (2006). "Measuring sensitivity to nonignorable censoring in nonparametric and semiparametric survival modeling." *Doctoral Dissertation*. The University of Pennsylvania. (http://repository.upenn.edu/dissertations/AAI3246193/)
40. Fedorov, V. V. and **Liu, T.*** (2005). "Randomized discontinuation trials: design and efficiency." GlaxoSmithKline Biomedical Data Science Technical Report, 3. (*Authorship in Alphabetical order).
41. **Liu, T.** (2001). "Ammonia inhibition in thermophilic anaerobic process." Master thesis, Iowa State Univ. (http://books.google.com/books?id=_XZfNwAACAAJ)

D. Manuscript in Progress

42. Merchant, R.C., **Liu, T.**, and Baird, J. (2015) "Comparison of Priorities for Addressing Adult Emergency Department Patient Substance Misuse based on Simplified Screening Strategies versus Formal Substance Misuse Evaluations." Under revision.
43. **Liu T**, Huang R, Hogan J, and Kantor R. (2015) "Improved Viral Load Monitoring Capacity with Rank-Based Algorithms for Pooled Assays". Under revision.
44. Merchant R.C., **Liu, T.**, and Baird, J.R. (2015) "Priorities for substance misuse interventions among adult emergency department patients based on prevalence, recency of use, frequency and severity of use misuse screening strategies." Under preparation.
45. **Liu T**, Hogan J, Xu Y, DeLong Am and Kantor R. (2015) "Covariate-Assisted Pooled Nucleic Acid Testing to Identify Antiretroviral Treatment Failure During HIV Infection". Under revision.
46. Omenge, O., **Liu, T.**, Christoffersen-Deb A., Itsura, P., Mabeya, H., Washington, S., and Cu-Uvin, S. (2015) "A comparison of Visual Inspection with Acetic Acid (VIA), Pap smear and HPV typing for follow-up after VIA/Cryotherapy in HIV-infected women." [working title] Under preparation.
47. Jones, M.M, **Liu, T.** and Shenassa, E. (2015). "Differential secular trends in BMI in relation to region of childhood residence: A 25 year follow-up of a nationally representative sample". Under preparation.

E. Abstracts / Conference Presentations

48. **Liu T.** and Hogan J. (2015). "Coherence, Relevance, Transparency: The three requirements for sensitivity analysis in clinical trials." The Fourth ISBS/DIA Joint Symposium on Biopharmaceutical Statistics, June 29-July 1, Beijing, China.
49. Merchant RC, **Liu T**, Clark M, Rosenberger J, Bauermeister J and Mayer K (2015). "How do young adult social media-using black, Hispanic and white MSM differ in their interest in using fingerstick rapid HIV self-testing? Implications for encouraging testing among hard to reach populations." The 2015 International AIDS Society Meeting (late breaker). Vancouver, Canada. July 20-22. (Submitted)

50. Kuo I, Kurth A, **Liu T**, Trezza C, Bazerman L, Castonguay B, Olsen H, Patrick R, Abbott S, Cates A, Peterson J, Beckwith C. (2015) "Gender Disparities Among HIV-Positive Individuals Involved in the Criminal Justice System: The CARE+ Corrections Study in Washington, DC". The 2015 National HIV Prevention Conference. Atlanta, GA, December 6-9, 2015. (Submitted).
51. Merchant RC, **Liu T**, Melissa C, Bauermeister, J, Mayer K. (2015). "Incongruence between self-perceived HIV risk and reported sexual risk among social media-using Black, Hispanic and white MSM." The 2015 National HIV Prevention Conference. Atlanta, GA, December 6-9, 2015. (Submitted).
52. Merchant RC, **Liu T**, Melissa C, Bauermeister, J, Mayer K. (2015). "Low PrEP utilization among among social media-using young adult Black, Hispanic and white MSM at higher risk for HIV acquisition." The 2015 National HIV Prevention Conference. Atlanta, GA, December 6-9, 2015. (Submitted).
53. (*Invited presentation*) **Liu T** (2015) "Coherence, Relevance, Transparency: The three requirements for sensitivity analysis in clinical trials" The 4th International Symposium on Biopharmaceutical Statistics, Beijing, China, June 29 - July 1, 2015.
54. Merchant RC, DeLong AK, **Liu T** and Baird JR (2015) "Does a brief intervention among drug misusing adult emergency department patients increase uptake of rapid HIV/HCV screening?" The 2015 National Summit on HCV and HIV Diagnosis, Prevention and Access to Care, June 4-6, Arlington VA. (Submitted).
55. Kuo I, Kurth A, Cates A, Trezza C, Bazerman L, Castonguay B, Olsen H, Patrick R, **Liu T**, Peterson J, Abbott S, Beckwith C (2015). "HIV Risk and Clinical Characteristics Among HIV-Positive Individuals Involved in the Criminal Justice System: The CARE+ Corrections Trial in Washington, DC" The 2015 International AIDS Society Meeting. Vancouver, Canada. July 20-22. (Accepted).
56. Merchant RC, Baird JR, **Liu T**, and Nirenberg TD. "Does A Brief Intervention For Adult Emergency Department Patients Decrease Drug Misuse And Increase Drug Treatment Services Utilization?" [abstract] 2015 Society for Academic Emergency Medicine Annual Meeting, San Diego, CA, May 12-15, 2015. (Accepted).
57. Merchant RC, Shao W, Guan W*, Clark MA, **Liu T**, Santelices C and Cortes DE (2015). "Variations in recruitment yield, costs, and speed and participant diversity across internet or social media platforms in a global study of HIV/AIDS and HIV testing knowledge". The 2015 International AIDS Society Meeting. Vancouver, Canada. July 20-22. (Accepted).
58. Merchant, R.C., Baird, J.R., **Liu T**, and Nirenberge, T.D. (2015). "Short-term efficacy of a brief intervention to reduce drug misuse and increase drug treatment utilization among adult emergency department patients". The 2015 Society for Academic Emergency Medicine (SAEM) Meeting. San Diego, CA. May 12-16, 2015. (Accepted).
59. Guan W*, **Liu T**, Baird JR, and Merchant RC (2015) "Evaluation of a Brief Intervention to Reduce Negative Consequences of Drug Misuse among Adult Emergency Department Patients." Brown Public Health Research Day (Poster Presentation). April 16 2015, Brown University, Providence, RI. (*Advised student)
60. Stillman, AE, Gatsonis, CG, Lima, J, Cormack, J, Gareen, I., Hoffmann, U, **Liu T.**, Mavromatis, K., Schnall, M., Udelson, J., Woodward, P., and RESCUE Investigators ACRIN. (2015). "Randomized Evaluation of Patients with Stable Angina Comparing Utilization of

Noninvasive Examinations (RESCUE) Trial." The 64th Annual Scientific Session & Expo of American College of Cardiology (ACC). San Diego, CA. March 14-16, 2015.

61. (*Invited lecture*) **Liu T** (2015) "Design of non-inferiority and equivalence clinical trials with time-to-event outcomes: The RESCUE study." North Sichuan Medical College School of Public Health, March 3-4, Sichuan, China.
62. **Liu, T.**, Hogan, J.W., Huang, R.*, Kantor, R. (2015) "Improved viral load monitoring capacity with rank-based algorithms for pooled assays". The 2015 Conference on Retroviruses and Opportunistic Infections (CROI). Seattle, WA. Feb 23-26, 2015. (*advised student).
63. (*Invited Poster Presentation*) **Liu T**, Hogan JW, and Kantor R (2014). "Optimal Allocation of Viral Load Testing Under Resource Constraints". The 2014 CFAR National Meeting. Providence, RI. November 5-6, 2014.
64. Merchant, R.C., Baird, J.R., **Liu, T.**, Beaudoin, F., and Nirenberg, T. (2014). "Severity of substance misuse and need for substance misuse interventions among adult emergency department patients." [abstract 189] American College of Emergency Physicians Scientific Assembly. Chicago, IL, October 27. (Peer reviewed).
65. Shao, W., Merchant, R., Clark, M., **Liu, T.**, Guan, W., Santelices, C, Cortes, D. (2014). "Does a Video Improve HIV/AIDS and HIV Testing Knowledge Among a Global Sample of Internet and Social Media Users?" [abstract 35819] National Conference on Health Communication, Marketing, and Media. Atlanta, GA, August 20. (Peer reviewed).
66. (*Invited Presentation*) **Liu, T.**, Hogan, J.W., and Kantor, R. (2014). "Optimizing Diagnosis of HIV Treatment Failure with Selective Use of Gold Standard Test." The 2014 New England Statistics Symposium. Harvard University School of Public Health, Boston, MA. April 25-26.
67. Shao, W., Merchant, R., Clark, M., **Liu, T.**, Guan, W., Santelices, C, Cortes, D. (2014). "How Do Internet and Social Media Sites Compare in Achieving Optimal Completion Rates and Costs for an Internet-Based Research Study?" The 2014 CDC National Conference on Health Communication, Marketing, and Media. (August 19-21). (Peer reviewed).
68. Omenge, E., **Liu, T.**, Itsura, P., Mabeya, H., Christofferson-deb, A., Washington, S., and Cu-Uvin, S. (2014) "Visual inspection with acetic acid is good for follow-up after cryotherapy in HIV-infected women." The 2014 Conference on Retroviruses and Opportunistic Infections. March 3-6, 2014, Boston, MA. (Peer reviewed).
69. Merchant RC, Baird JR, **Liu T.**, Taylor L, Montague B, and Nirenberg T. (2014). "Does a Brief Intervention Increase HIV/HCV Screening among Drug-using Emergency Department Patients?" [abstract #743] 2014 Society for Academic Emergency Medicine Annual Meeting, Dallas, TX, May 14, 2014. (Peer reviewed).
70. **Liu, T.**, Hogan, J.W., Wang, L., Zhang, S. and Kantor, R. (2014) "Optimizing diagnostic accuracy of HIV treatment failure in resource-limited settings with selective use of of viral load testing." The 17th International Workshop on HIV Observational Databases. Cavtat, Croatia. March 2014. (Reference #17-185). (Peer reviewed).
71. Jones, M.M, **Liu, T.,** and Shenassa, E.D. (2013) "Differential secular trends in BMI in relation to region of childhood residence: A 25 years follow-up of a nationally representative sample." (Poster, Session 4362). The American Public Health Association (APHA) 141st Annual Meeting. Nov 2-6, 2013, Boston, MA. (Peer reviewed).

72. Thakore, R.*, Pinkson, M., and **Liu, T.** (2013). "The effect of cocaine Use on the diet and metabolic status of HIV-infected individuals." Brown Public Health Research Day (Poster Presentation). April 18, Brown University, Providence, RI. (*Advised student).
73. Tai, M.*, **Liu, T.**, and Merchant, R.C. (2013). "HIV/AIDS-related hospitalization rates in US short-stay hospitals, 1982-2010." Brown Public Health Research Day (Poster Presentation). April 18, Brown University, Providence, RI. (*Advised student)
74. Omenge, E., Itsura, P., **Liu, T.,** Mabeya, H., Christoffersen-Deb, A., Washington, S., and Cu-Uvin, S. (2013). "Six Months Follow-up of HIV-infected Women After Visual Inspection with Acetic Acid (VIA) and Immediate Cryotherapy in Western, Kenya." The 2013 Conference on Retroviruses and Opportunistic Infections (CROI). March 3-6, 2013; Poster. (Peer-reviewed).
75. Merchant RC, Clark MA, **Liu, T.**, Santelices C, and Cortes D. (2012). "Can a video substitute for an in-person discussion in delivering HIV pre-test information to Spanish-speaking Latinos and better serve those with lower health literacy?" [Paper #31945] 2012 National Conference on Health Communication, Marketing, and Media. Atlanta, GA, August 7-9, 2012. (Peer-reviewed).
76. Merchant R.C., Clark M.A., **Liu, T.**, Santelices C., and Cortes D. (2012) "Can a video substitute for an in-person discussion in delivering HIV pre-test information to Spanish-speaking Latinos?" The 2012 National Summit on HIV and Viral Hepatitis Diagnosis, Prevention and Access To Care. Poster #116. Washington DC. November 26-28, 2012. (Peer-reviewed).
77. Tai, M.*, **Liu, T.**, and Merchant, R.C. (2013). "HIV/AIDS-related hospitalization rates in US short-stay hospitals, 1982-2010." The 2012 National Summit on HIV and Viral Hepatitis Diagnosis, Prevention and Access To Care. Abstract #80. Washington DC. November 26-28, 2012. (Peer-reviewed).
78. Kuo I., Kurth A., Bazerman L., Peterson J., Cates A., Azikiwe N., Wong J., **Liu, T.**, and Beckwith C. (2012). "CARE+ Corrections—Innovative use of technology in jails to support linkage and adherence to HIV care." The 2nd International Treatment as Prevention Workshop 2012, April, Vancouver, BC, Canada.
79. (*Invited Presentation*) **Liu, T.**, Hogan, J.W., Zhang, S., Kantor, R. (2012). "Using auxiliary biomarkers to improve pooling strategies for HIV viral load testing." The 2012 International Biometrics Society (IBS) ENAR Meeting, May, Washington DC.
80. (*Invited Presentation*) **Liu, T.** (2011). "Optimizing diagnosis of HIV viral failure with selective use of gold standard testing in resource limited settings." CFAR Research-in-Progress Lectureship Series, November 18, 2011. The Mariam Hospital, Providence, RI.
81. (*Invited Presentation*) **Liu, T.** (2011). "Instrumental variable method and principal stratification: An application to a CFAR study." ACRIN Working Group Meeting, Brown University, Oct 18, 2011, Providence, RI.
82. Baird J.R., Merchant R.C., Nirenberg T.D., **Liu, T.**, and Ladd G.T. (2011). "Increasing the uptake of testing for chlamydia and gonorrhea among young female Emergency Department patients." [Abstract: 244972] 139th American Public Health Association Annual Meeting, Nov 1, Washington, DC. (Peer-reviewed).
83. Trillo A., Merchant R.C., Baird J.R., **Liu, T.**, and Nirenberg, T.D. (2011). "Gender differences in alcohol misuse and estimated blood alcohol levels among emergency department patients: implications for brief interventions." [Abstract: 16] 8th Annual International Net-

work on Brief Interventions for Alcohol & Other Drugs (INEBRIA) Conference, Sept 22, Boston, MA. (Peer-reviewed).

84. Merchant R.C., Baird J.R., Nirenberg T.D., **Liu, T.**, and Ladd G.T. (2011). "Does self-report of alcohol misuse and sexual risk-taking behavior motivate patients to be tested for HIV? "[Abstract: 1399] CDC 2011 National HIV Prevention Conference, Aug 14-17, Atlanta, GA. (Peer-reviewed).

85. Merchant R.C., Baird J.R., Nirenberg T.D., **Liu, T.**, and Ladd G.T. (2011). "Increasing HIV sexual risk-taking as a function of alcohol misuse among emergency department patients." [Abstract: 1408] CDC 2011 National HIV Prevention Conference, Aug 14-17, Atlanta, GA. (Peer-reviewed).

86. (*Invited Presentation*) **Liu, T.** (2011). "Optimizing diagnosis of HIV treatment failure with selective use of gold standard test." ACRIN Working Group Meeting, Brown University, July 27, 2011, Providence, RI.

87. (*Invited Presentation*) **Liu, T.** (2011). "Comparison of positive predictive values in matched pairs clinical trials." Invited Lectureship Series of the 60th Anniversary of North Sichuan Medical College. June 7, 2011. Nanchong, Sichuan, China.

88. Hogan, J., **Liu, T.**, Kantor, R., Wang, L., and Zhang, S. (2011). "Optimizing diagnosis of HIV viral failure with selective use of gold standard testing in resource limited settings." Workshop on Statistical Methods in HIV research. April 14-15, 2011, Montreal Canada. (By Invitation).

89. **Liu, T.**, Hogan, J. and Kantor, R. (2011). "Optimal use of selective viral load testing for HIV treatment monitoring in resource limited settings." The 2011 International Biometrics Society (IBS) ENAR Meeting. March, Miami, FL.

90. Anderson, A., Firnhaber C., **Liu, T.**, Swarts, A., Siminya, M. and Cu-Uvin, S. (2011). " " The International Conference on Retroviruses and Opportunistic Infections. Feb 27-Mar 2, Boston, MA. (Peer-reviewed).

91. Anderson, B., Firnhaber, C., Swarts, A., Siminya, M., **Liu, T.**, and Cu-Uvin, S (2010). "Self-Collected Vaginal Swabs for Trichomoniasis Testing in HIV-infected Women in South Africa." The 34th Annual Scientific Meeting of Infectious Diseases Society for Obstetrics and Gynecology (IDSOG). Aug. 5-7. Santa Fe, NM. (Peer-reviewed).

92. Guzman H., Merchant, R.C., Baird, J.R., Nirenberg, T.D., **Liu, T.**, Ladd, G.T. (2010) "How does hazardous and heavy episodic alcohol drinking relate to HIV sexual risk among emergency department patients?" *Annals of Emergency Medicine*, 56 (3), S22-S22. [PII: S0196-0644(10)00690-6; doi:10.1016].

93. Guzman, H., Merchant, R.C., Baird, J.R., Nirenberg, T.D., **Liu, T.**, and Ladd, G.T. (2010). "How does hazardous and heavy episodic alcohol drinking relate to HIV sexual risk among emergency department patients?" 2010 American College of Emergency Physicians Scientific Assembly Research Forum, Las Vegas, NV. (Peer-reviewed).

94. Xu, H*, and **Liu, T.** (2010). "Community Context and Obesity among Chinese Adults: A Longitudinal and Multilevel Analysis." Population Association of America 2010 Annual Meeting, Dallas, TX. (Peer-reviewed) (*Advised student).

95. Mabeya, H., Khozaim, K., **Liu, T.,** Orango, O., Chumba, D., Pisharodi, L., and Cu-Uvin, S. (2010). "Comparison of Conventional Pap Smear and Visual Inspection with Acetic Acid (VIA) in HIV Infected Women in Western Kenya." The International Conference on Retroviruses and Opportunistic Infections (CROI) 2010, San Francisco, CA. (Peer-reviewed).

96. **Liu, T.**, DeLong, A., and Blossom, B. (2009). “Challenge to IDSA recommendations for late neurologic Lyme disease treatment and post Lyme syndrome.” The 2009 International Lyme and Associated Diseases Society (ILADS) Conference, Washington DC. 2010. (By invitation).

97. Merchant R.C., Waxman M.J., Clark M.A., Maher J.C., Celada M.T., Beckwith C.G., **Liu, T.**, Simmons E.A., and Mayer K.H. (2009). “Do patients and clinicians have ethical concerns about the 2006 CDC HIV testing recommendations?” The 2009 CDC National HIV Prevention Conference, Atlanta, GA. (Peer-reviewed).

98. Waxman M.J., Merchant R.C., Clark M.A., Maher J.G., Celada M.T., Beckwith C.G., **Liu, T.**, Simmons E.A., and Mayer K.H. (2009). “Will the 2006 CDC HIV testing recommendations reduce stigma and increase HIV testing?” The 2009 CDC National HIV Prevention Conference, Atlanta, GA. (Peer-reviewed).

99. Nijhawan A., McManus J., **Liu T.**, DeLong A.K., Chapman S., Rapoza K., Kurpewski J., Caliendo A. and Cu-Uvin S. (2009). “The effect of Acyclovir on CD4 count and CD4 percent slope in HIV-1/HSV-2 co-infected women taking highly active antiretroviral therapy (HAART).” The 47th IDSA Annual Meeting; Oct. 29-Nov. 1, 2009, Philadelphia, PA.

100. McManus J., Nijhawan A., **Liu T.**, DeLong A., Chapman S., Rapoza K., Kurpewski J., Caliendo A. and Cu-Uvin S. (2009). “The effect of Acyclovir on the prevalence of bacterial vaginosis on HIV-1/HSV-2 co-infected women taking highly active antiretroviral therapy (HAART).” 47th IDSA Annual Meeting; Oct. 29-Nov. 1, 2009, Philadelphia, PA.

101. Waxman, M.J., Merchant, R., Maher, J., Clark, M., Simmons, E., Celada, T., Beckwith, C., **Liu, T.** (2009). “Who prefers CDC-recommended streamlined methods for HIV testing?” The 2009 Society for Academic Emergency Medicine (SAEM) Annual Meeting, New Orleans, LA. (Peer-reviewed).

102. Beckwith, C.G., **Liu, T.**, Bazerman, L.B., DeLong, A.K., Pinkston, M., Nunn, A., and Flanigan, T.P. (2009). “Identification of multiple HIV risk factors among jail detainees.” The 2009 CDC National HIV Prevention Conference, Atlanta, GA. (Peer-reviewed).

103. Waxman, M.J., Merchant, R.C., Maher, J.G., Clark, M.A., Simmons, E.A., Celada, M.T., Beck- with, C.G., **Liu, T.**, Mayer, K.H. (2008). “Do clinicians prefer the 2006 or 2001 CDC HIV testing recommendations?” The 2008 Forum for Collaborative HIV Research. Poster # 151. (Peer-reviewed).

104. Waxman, M.J., Clark, M.A., Maher, J.C., Simmons, E.A., Celada, M.T., Beckwith, C.G., **Liu, T.**, and Mayer, K.H. (2008). “Do patients and clinicians have ethical concerns about the 2006 CDC HIV testing recommendations?” The CDC National HIV Prevention Conference. (Peer-reviewed).

105. Beckwith, C., **Liu, T.**, Bazerman, L., DeLong, A.K., Desjardins, S., and Flanigan, T. (2008). “HIV risk behavior pre- and post-HIV testing and counseling in jail.” The 2008 Infectious Diseases Society of America (IDSA) Annual Meeting, Washington DC. (Peer-reviewed).

106. Phelan, S., Gorin, A., **Liu, T.**, Hogan, J.W., Lowe, M., Fava, F., and Wing, R. (2008). “What distinguishes long-term weight loss maintainers from the treatment-seeking obese? Analysis of environmental, behavioral, and psychosocial variables.” The 2008 Annual Scientific Meeting of the North American Association for the Study of Obesity (NAASO), Phoenix, AZ. (Peer-reviewed).

107. Moreira C., Delong A.K., **Liu, T.**, Kurpewski J., Ingersoll J., Caliendo A., and Cu-Uvin S.C. (2008). “HIV-infected women on highly active antiretroviral therapy with asymptomatic bacterial vaginosis have low rates of genital tract HIV-1 RNA shedding.” The 17th International AIDS Conference (IAS), Mexico. (Peer-reviewed).

108. Merchant R.C., DePalo D.M., **Liu, T.**, Rich J.D., and Stein M.D. (2008). “Predictors of a chlamydia and/or gonorrhea infection among adult male emergency department patients.” The 2008 National Sexually Transmitted Diseases (STD) Prevention Conference, Chicago, IL. (Peer-reviewed).

109. Anderson B.L., **Liu, T.**, DeLong A.K., Ingersoll J., Wang C.C., Kurpewski J., Caliendo A., and Cu-Uvin S.C. (2007). “Genital tract shedding of HIV-1 RNA and lower genital tract infections may be linked by inflammation.” The 45th Annual Meeting of the Infectious Diseases Society of America (IDSA), San Diego, CA. 2008 (Peer-reviewed).

110. Wang C.C., **Liu T.**, Anderson B.L., DeLong A.K., Chapman S., Kurpewski J., Ingersoll J., Caliendo A.M., Cu-Uvin S.C. (2008) “Association between genital tract HIV-1 RNA shedding and mucosal inflammation among women on effective ART with undetectable plasma viral load.” Poster presentation. The 15th Annual Conference on Retroviruses and Opportunistic Infections (CROI), February, 2008, Boston, MA.

111. Anderson B.L., DeLong A.K., **Liu, T.**, Wang C.C., Kurpewski J., and Cu-Uvin S.C. (2007). “Association between genital tract white blood cells and viral shedding among HIV-infected women in the absence of genital infections.” The 34th Annual Scientific Meeting of Infectious Diseases Society for Obstetrics and Gynecology (IDSOG), Boston, MA. (Peer-reviewed).

112. **Liu, T**., Hogan, J.W., and DeLong, A.K. (2008). “Bounds of ATE and unmeasured confounding bias in observational studies with continuous outcome.” The 2008 International Biometric Society (IBS) ENAR meeting, Arlington, VA.

113. **Liu, T.** and Heitjan, D.F. (2007). “Sensitivity of Cox regression to nonignorable censoring.” The 2007 American Statistical Association (ASA) Joint Statistical meeting, Salt Lake City, UT.

114. (*Invited Presentation*) **Liu, T.** and Heitjan, D.F. (2006). “Sensitivity and Kaplan-Meier estimate to nonignorable censoring.” Dana-Farber Cancer Institute (Harvard University), Department of Biostatistics and Computation Biology, Cambridge, MA.

115. (*Invited Presentation*) **Liu, T.** and Heitjan, D.F. (2006). “Sensitivity and Kaplan-Meier estimate to nonignorable censoring.” Brown University, Center for Statistical Sciences, Department of Community Health, Providence, RI.

116. **Liu, T.** and Heitjan, D.F. (2006). “Sensitivity and Kaplan-Meier estimate to nonignorable censoring.” The 2006 American Statistical Association (ASA) Joint Statistical Meeting, Seattle, WA.

117. **Liu, T.** and Gao, L. (2006). “Relationship assessment between PK and PD.” The 2006 International Biometric Society (IBS) ENAR Meeting, Tampa, FL.

118. (*Invited Presentation; Distinguished Student Paper Award*) **Liu, T.** and Heitjan, D.F. (2006). “Sensitivity and Kaplan-Meier estimate to nonignorable censoring.” The 2006 International Biometric Society (IBS) ENAR Meeting, Tampa, FL.

119. **Liu, T.** and Fedorov, V.V. (2006). “Randomized discontinuation trials.” The 2006 International Biometric Society (IBS) ENAR Meeting, Tampa, FL.

RESEARCH GRANTS

A. Active Grants

1. Facilitating HIV Testing Among Young Adult MSM Through Social Networking
   My Role: Brown CSS sub-Award PI
   Funding Agency: NIH/Rhode Island Hospital subcontract
   PI: Merchant
   Type: R21
   Period: 4/15/2014-3-31/2016
   Cost: $91,042
   This project assesses acceptance of a new rapid HIV self-test, as compared to current HIV testing approaches, as well as usage of a MSM social-networking website to facilitate HIV testing and dissemination. The entire study will be internet-based, in regards to recruitment, enrollment, follow-up, and acquisition of HIV tests, which is appropriate given the high level of familiarity, use and comfort with the internet among young adult MSM.

2. Optimal HIV Treatment Monitoring Strategies under Resource Constraints
   My Role: Co-Investigator
   Funding Agency: NIH
   PI: Hogan, Kantor
   Type: R01
   Period: 12/1/2014-11/31/2019
   Cost: $2,882,699 (Project total)
   We propose to develop the statistical framework, theory and methods required to discover optimal diagnostic algorithms for monitoring treatment failure with limited or no VL availability; to use cohort data from both the US and Kenya to derive, calibrate and cross-validate the algorithms; to use extant blood samples from patients in a PEPFAR-funded HIV care program to design and cross-validate a new diagnostic algorithm that includes implementation of pooled assays; and to develop usable software that will enable programs to design their own protocols based on the characteristics of their patient population and local capacity for viral load testing.

3. Facilitating HIV/AIDS and HIV Testing Literacy for Emergency Department Patients
   My Role: Co-Investigator
   Funding Agency: NIH
   PI: Merchant
   Type: RO1
   Period: 7/23/2014-4/30/2019
   Cost: pending
   This project aims to address HIV/AIDS and HIV testing literacy deficits among emergency department patients. The video from the prior R21 study will be tested for efficacy as compared to a pictorial brochure in a multi-site, randomized, controlled, longitudinal study.

4. AMPATH-Oncology Institute: HPV and Cervical Cancer in Kenyan Women with HIV/AIDS
   My Role: Co-investigator and Lead Statistician
   Funding Agency: NCI

Brown PI: Lehrer, Darron, Omenge
Type: U54
Period: 9/19/2014-8/31/2019
Cost: $728,206 (Project total)
This project aims to assess the results of cryotherapy and LEEP among VIA positive HIV- and HIV+ women in western Kenya. Modifiable risk factors that lead to treatment failure and HPV types (persistence and alterations) also will be assessed.

5. “See and LEEP” Training for Kenyan Non-gynecologists
My Role: Brown sub-Award PI
Funding Agency: CFAR/PepFAR Supplement
PI: CuUvin
Type: P30
Period: 4/4/2014-3/31/2015
Cost: $364,969 (Project total)
This project proposes to evaluate the feasibility of training Kenyan nurses to perform cervical cancer screening and LEEP treatment for HIV+ women in Western Kenya and to assess its safety. Findings of the study will provide important information to improve health outcomes among HIV-infected women needing treatment for cervical neoplasia.

6. Alcohol and HIV: Biobehavioral Interactions and Intervention
My Role: Investigator
Funding Agency: NIAAA
PI: Monti
Type: P01 AA019072
Period: 09/30/10-08/31/15 (renewal pending)
Cost: $148,924
The goals of this alcohol research center are to study the effects of alcohol use on HIV disease progression and the effects of interventions to reduce alcohol use in HIV-infected populations. The center also fosters multidisciplinary collaborations and training in research on alcohol and HIV and dissemination of research findings to clinicians treating addictions and HIV.

7. CARE Corrections: A technology-based HIV and HCV tool
My Role: Brown sub-Award Principal Investigator
Funding Agency: NIDA/The Miriam Hospital subcontract
PI: Beckwith
Type: R01DA030747-01
Period: 07/01/10-06/30/15
Cost: $18,806
It involves providing biostatistical expertise and collaboration in a project related to a new intervention to reduce substance abuse and hence reduce risks of HIV and HCV infections.

8. A Stage 2 Cognitive-Behavioral Trial: Reduce Alcohol First in Kenya Intervention
My Role: Co-Investigator
Funding Agency: NIAAA/Brown
PI: Papas
Type: R01 AA020805-01

Period: 09/15/11 – 08/31/16
Cost: $429, 932
The major objectives of this stage 2 randomized clinical trial are to determine whether a para-professionally led group cognitive-behavioral therapy intervention is effective in reducing alcohol use among 336 HIV-infected Kenyan outpatients who report hazardous or binge drinking when compared against a group health education intervention

9. Evaluating the impact of patient-centric home health quality reports
My Role: Co-Investigator
Funding Agency: Healthcentric Advisors/AHRQ
Brown PI: Clark
Type: R21 HS021879-01
Period: 09/30/12-08/31/15
Cost: $69,111
The specific aims are to: (1) determine how home health consumers (patients and caregivers) use the current reporting content and format, and what changes they recommend to increase its frequency of use and decision-making utility; (2) determine how hospital discharge planners use the current reporting content and format, and what changes do they recommend to increase its frequency of use and decision-making utility; (3) evaluate whether or not home health consumers and hospital discharge planners who view a revised, consumer-centric, web-based public report understand the information and how to use it; and (4) evaluate the extent to which home health consumers offered a new consumer-centric report prior to hospital discharge use the information to inform their home health choices, and how their use, satisfaction and outcomes compare to consumers offered the old format.

10. Center for AIDS Research (CFAR) Biostatistics Core
My Role: Investigator
Funding Agency: NIH/The Miriam Hospital contract
Brown PI: Hogan
Type: P30 A1 42853
Period: 09/01/12 - 06/30/16
Cost: $100,966
The primary goal involves providing biostatistical expertise for the design and analysis of individual projects within CFAR; the core will also be responsible for implementation of data analyses and will participate in the preparation of reports and manuscripts.

B. Grant Submitted (Disposition Pending)

11. Mindfulness RCT for CVD Risk
My Role: Statistician
Funding Agency: NIH
PI: E. Loucks
Type: RO1 GRANT11597822
Cost: $535,497 (Project Total)
Period: 9/1/2014 - 8/31/2019
The research objective is to conduct a methodologically rigorous randomized controlled trial that will provide information on whether mindfulness-based stress reduction has sus-

tained one-year impacts on CVD risk factors, with systolic blood pressure and smoking as the primary outcomes.

12. Computerized Intervention to Improve HIV Medication Adherence
My Role: Brown sub-Award PI
Funding Agency: NIMH
PI: Ramsey
Type: RO1
This project aims to promote HIV medication adherence using an innovative computerized intervention. A revised computerized medication adherence intervention (MAI) will be tested for effectiveness as compared to a general health control intervention (GHC) in a randomized, controlled, longitudinal study.

13. The influence of fetal growth on infant growth and obesity among Samoans
My Role: Investigator
Funding Agency: NIMHD
PI: McGarvey
Type: RO1
The research objective is to document the relationships between maternal obesity and related pregnancy conditions to fetal growth and directly relate temporal patterns of fetal growth to childhood obesity.

14. Behavioral Intervention to Prevent Child Overweight and Obesity in American Samoa
My Role: Investigator
Funding Agency: NIMHD
PI: McGarvey
Type: R01
Cost: $1,250,000 (Project total)
The research objective is to conduct a randomized trial of family-based lifestyle intervention with conditional incentives on parents in order to prevent excessive weight gain among American Samoan children 7-12 years of age.

C. Completed Grants

15. Brief Intervention for Drug Misuse in the Emergency Department (BIDMED)
My Role: Brown Principal Investigator
Funding Agency: NIDA/Rhode Island Hospital subcontract
PI: Merchant
Type: R01 DA026066
Period: 03/15/09-02/28/14
Cost: $27,705
The primary goal involves providing biostatistical expertise and collaboration in a project related to a new intervention to reduce drug misuse in the Emergency Department.

16. Randomized Evaluation of Patients with Stable Angina Comparing Utilization of Noninvasive Examinations (RESCUE)
My Role: Protocol Statistician
Funding Agency: AHRQ
Brown PI: Gatsonis

Type: R01 HS019403
Period: 09/30/10-09/29/14
Cost: $401,721
This research project involves organizing and operating the Biostatistics Center and the Outcomes and Economics Core Laboratory components of the RESCUE trial - a randomized, multi-center study designed to compare CCTA and SPECT for patients with symptoms of stable angina.

17. Cervical Cancer See and Treat: How Best to Follow-Up
My Role: Co-Investigator
Funding Agency: NIAID/The Miriam Hospital subcontract
PI: Cu-UVin
Type: P30 A142853
Period: 07/01/11 – 06/30/13
Cost: $51,488
This proposal involves providing biostatistical expertise and collaboration in a project related to comparing three methods, visual inspection with acetic acid (VIA), pap smear and HPV typing, for diagnosing cervical neoplasia/cancer.

18. Methods to Optimize Use of Limited Resources for Monitoring Treatment of HIV/AIDS in the Developing World
My Role: Principal Investigator
Funding Agency: Center for AIDS Research
PI: Liu
Type: 5P30 AI042853-12
Period: 1/01/10-12/31/11
Cost: $40,000
This project involves developing a clinical algorithm that will make optimal use of viral load assays when they are available on a limited basis.

19. Increasing Viral Testing in the Emergency Department (InVITED)
My Role: Brown sub-Award Principal Investigator
Funding Agency: NIDA/Rhode Island Hospital subcontract
PI: Merchant
Type: R21 DA028645
Period: 3/04/10 – 2/28/2012
Cost: $15,000
This project involves providing biostatistical expertise and collaboration in a project related to a new intervention to reduce substance abuse.

20. Video-Based Delivery of HIV Test Information for Spanish-Speaking Latinos
My Role: Investigator
Funding Agency: DHHS/ Rhode Island Hospital subcontract
PI: Merchant
Type: R21 NR011997
Period: 03/04/11-02/29/13
Cost: $18,806
This research project involves comparing the effects of video delivery of HIV testing information for Spanish-Speaking Latinos.

21. New Approaches to Mediation Analysis using Causal Inference Methods
    My Role: Co-Investigator
    Funding Agency: NIAAA (stimulus)
    PI: Hogan
    Type: 1RC1AA019186-01
    Period: 10/01/09-09/30/11
    Cost: $327,201 (Project total)
    The proposed research will develop statistical approaches for discovering pathways and mechanisms of behavioral interventions targeted at alcohol abuse. Knowledge of mechanistic pathways allows deeper understanding of how and why certain interventions work, and opens the door to customizing interventions based on person-specific characteristics.

22. Shaping Long-Term Care in America
    My Role: Statistician
    Funding Agency: NIH/NIA
    PI: Mor
    Type: P01 AG027296
    Cost: $1,390,110 (Project total)
    Period: 09/01/08-06/30/10
    The overall objectives of the program project are to (1) Build a long term care data and policy repository and analysis infrastructure as the basis for currently proposed and future examining the nations long term care" system;" (2) Implement a survey of states policies and a national sample of nursing homes, integrating them with secondary data to serve the proposed research projects within an overall administrative structure; (3) integrate economic and organizational theory to develop measures and to propose testable hypothesis in the proposed projects focusing on provides responses to market and policy changes; and (4) systematically catalogue and broadly disseminate to numerous audiences project findings.

23. Analyzing Complex Longitudinal Data in Behavior Sciences
    My Role: Lead Statistician
    Funding Agency: NIH
    Brown PI: Hogan
    Type: RO1 HL079457
    Cost: $249,500 (Project total)
    Period: Aug., 1, 04 --- July, 31, 08
    This project will develop innovative approaches to the analysis of longitudinal data specifically for behavioral medicine trials. The first two components of the project will develop latent class and latent variable models for classifying patterns of behavior change in smoking cessation and weight change. The third aim of the study addresses the issue of informative missing data, presenting a unifying and coherent framework for pattern-mixture modeling and associated sensitivity analyses. All of the methods will be developed on and applied to data from recently completed or ongoing trials in behavioral medicine, particularly in smoking cessation and weight change.

24. Secondhand Tobacco Smoke Exposure, Maternal Stress, and Adverse Birth Outcomes
    My Role: Lead Statistician
    Funding Agency: Flight Attendants Medical Research Institute

PI: Shenassa
Type: FAMRI 032069
Cost: $623,239 (Project total)
Period: Sept. 1, 08 --- Dec. 31, 08

This is a study to elucidate mechanisms explaining the heightened susceptibility to adverse health effects of secondhand tobacco smoke exposure observed among people of low SES by examining the exacerbating role of stress as measured by Allostatic Load.

ACADEMIC SERVICES

A. Department and University Services

1. Search Committee for Assistant or Associate Professor, Teaching Scholar. 2015.
2. Biostatistics PhD Student Admission Committee. 2011— 2013, 2014 — 15.
3. The 2014 Atlantic Causal Inference Conference (ACIC) Organizing Committee. 2014.
4. Best Thesis Award Committee. 2013 — 2014
5. Biostatistics Master Student Admission Committee. 2014.
6. Biostatistics Curriculum Committee. 2012 — 2014.
7. The 2014 Scholarship for Overseas Chinese Students Evaluation Committee 2014.
8. Search Committee for Research Assistant Professor of Biostatistics. 2013.
9. Biostatistics PhD Qualifying Exam Committee. 2008 — 11, 2012 — 13
10. Biostatistics Graduate Program Committee. 2011 — 2013.
11. Epidemiology MPH Qualifying Exam Committee. 2010 — 2011.

B. Editorial Services

<u>Journal Reviewer</u>

Journal of the American Statistical Association (JASA); Annals of Applied Statistics; Annals of Epidemiology; Biometrics; Biostatistics; Statistics in Medicine; Statistical Science; Statistics and Probability Letters; Statistical Modelling; Journal of Computational and Graphical Statistics (JCGS); Health Services and Outcomes Research Methodology (HSOR); The American Journal of Epidemiology (AJE); BMC Infectious Diseases; Emerging Themes in Epidemiology; Epidemiology; Clinical Infectious Diseases (CID); International Journal of Epidemiology & Infection; Journal of AIDS & Clinical Research; Journal of Clinical Epidemiology (JCE).

<u>Book Reviewer</u>

1. Chapman & Hall/CRC Press.

<u>Journal Editorial Board</u>

1. 2010 – Present Journal of Clinical Research & Bioethics (JCRB).
2. 2012 – Present International Journal of Clinical Therapeutics and Diagnosis.
3. 2013 – Present Annals of Biometrics & Biostatistics (ABB).
4. 2010 – Present International Journal of Epidemiology & Infection

C. Grant Reviewer

1. 2015 Ad-Hoc reviewer for Beijing Natural Science Foundation & Shanyuan Joint Research Projects: *Infectious Diseases and Epidemiology.*
2. 2014 Ad-Hoc reviewer for the National Science Foundation (NSF): *Panel of Methodology, Measurement, and Statistics*.
2. 2013 Scientific expert reviewer for the French National Research Agency (ANR): *Basic Research on Networks of Trials*.

D. Other Academic Services

1. 2015 Judge of the Best Student Poster Awards, Brown Public Health Research Day, April 16th 2015.
2. 2011-2012 Chair of the 2011-12 Starr Lectureship Series on “Comparative Effective Research and Causal Inference”. Brown University.
3. 2011-2012 Chair of the Biostatistics Seminar Series. Department of Biostatistics, Center for Statistical Sciences, Brown University.
4. August 2009. Chair of “Biometrics Section” (No.570). The American Statistical Association Joint Statistical Meeting (JSM). Washington D.C.

HONORS & AWARDS

1. 2010 The Lifespan/Tuft/Brown CFAR Developmental Award.
2. 2006 The Biometric Society ENAR Distinguished Student Paper Award.
3. 2004 Jonathan Raz Award for the Best Doctoral Qualifying Exam.
4. 1999-2002 Iowa State University Graduate Scholarship.
5. 1994-1998 Tsinghua University Undergraduate Scholarships.
6. 1994 Outstanding Freshmen Award at Tsinghua University.
7. 1993 Second Prize, National Physics Olympiad (High School).

TEACHING

A. Brown University (Instructor):

1. Spring 2012 PHP 2511 Applied Regression Analysis. (student enrollment: 18).
2. Fall 2009 PHP 2510 Principles of Biostatistics and Data Analysis. (27).
3. Spring 2008 PHP 2603 Analysis of Longitudinal Data (with Prof. Hogan). (15).

4. Fall 2008 PHP 2601 Linear and Generalized Linear Models. (11).

B. University of Pennsylvania (Graduate Teaching Assistant/Instructor):

5. Spring 2006 BSTA 771 Applied Bayesian Analysis. (7).
6. Fall 2005 BSTA 630 Statistical Methods and Data Analysis. (26).
7. Spring 2004 EPI 520 Introductory Biostatistics. (32).

C. Iowa State University (Graduate Instructor):

8. Summer. 2002 STAT 402 Statistical Methods for Research Workers. (43).

G. Invited/Guess Lectures

1. Fall 2013 "Clinical Trials Methodology"
   Brown University, School of Public Health
2. Summer 2012 "Causality 101 Workshop: Recent Mediation Analysis Methods"
   Brown University, School of Public Health
3. Fall 2010 "Design of Clinical Trials with Survival Outcomes"
   Brown University, School of Public Health
4. Fall 2010 "Statistical Methods in Biology"
   Brown University, Department of Molecular Biology
4. Fall 2009 "Statistical Methods in Biology"
   Brown University, Department of Molecular Biology

ADVISING/CONSULTING

A. Thesis Advisor/Reader

1. Ran Wei, Sc.M. (Biostatistics) 2016 (Expected).
   Thesis: To be decided.
   My role: Thesis Advisor.
1. Wentao Guan, Sc.M. (Biostatistics) 2015.
   Thesis: Evaluation of a Brief Intervention to Reduce Negative Consequences of Drug Misuse among Adult Emergency Department Patients.
   My role: Thesis Advisor.
2. Jay Jia Xu, B.S. (Statistics) 2015.
   Thesis: A Comparison of INLA and JAGS When Applied To Multiple Imputation with the Penalized Spline of Propensity Prediction Method
   My role: Thesis reader.
3. Renxia Huang, Sc.M. (Biostatistics) 2014.
   Thesis: Combining Routine Clinical Markers and Pooled Viral Load Strategy to Monitor Antiretroviral Treatment Failure in Resource Poor Settings.

My role: Thesis Advisor.
Winner of the 2014 Best Thesis Award of Public Health

4. Andrea Austin, Ph.D. (Biostatistics) 2013.
Thesis: Methods for prediction and inference on social networks with extensions to bimolecular pathways.
My role: Committee member.

5. Miao Tai, Sc.M. (Biostatistics) 2013
Thesis: HIV/AIDS-related hospitalizations in United States short-stay hospitals, 1982-2010
My role: Thesis Advisor.

6. Rachel Thakore, B.S. (Community Health) 2013
Honors Thesis: The effect of cocaine use on the diet and metabolic status of HIV-infected individuals.
My role: Committee member

7. Xuan Deng, Sc.M. (Biostatistics) 2013
Thesis: Mediation effects of behavioral and psychological factors in maintaining weight loss.
My role: Committee member.

8. Edwin Sang, Sc.M. (Biostatistics) 2013
Thesis: Causal effect of HIV serostatus disclosure on mortality and loss to follow-up after HAART initiation using propensity score.
My role: Committee member
Winner of the 2013 Best Master Thesis Award of Public Health

9. Ann Mwangi, Ph.D. (Biostatistics) 2011
Thesis: Addressing selection bias in observational event history data, with application to HIV in Western Kenya.
My role: Committee member.

10. Jing Zhang, Ph.D. (Biostatistics) 2011
Thesis: Causal Inference for Mediation Effects.
My role: Committee member.

11. Raymond Ng, Sc.M. (Biostatistics) 2011
Thesis: Analysis of repeated FDG-PET scans in the National Oncology PET Registry (NOPR).
My role: Committee member.

12. Patricia S. Fox, Sc.M. (Biostatistics) 2009
Thesis: Transition within states of lung function: A multinomial logistic approach using two-year follow-up data from bacteriologically cured tuberculosis patients.
My role: Committee member.

13. Lisa tai (Asnis), Sc.M. (Biostatistics) 2009
Thesis: Evaluating the utilization and efficiency of ICD-9 codes to identify visits for blood or body fluid exposures in 11 Rhode Island emergency departments.
My role: Thesis advisor.

C. Graduate Academic Advisor

1.	Ran Wei,	Sc.M. (Biostatistics)	2016 (expected)
2.	Jin Shi,	Sc.M. (Biostatistics)	2016 (expected)
3.	Wuntao Guan	M.S. (Biostatistics)	2015
4.	Lisa Wang,	M.S. (Biostatistics)	2013
5.	Nuo Xu,	M.A. (Biostatistics)	2012
6.	Junchao Shangguan,	M.A. (Biostatistics)	2011
7.	Dong-Hyun Ahn,	M.A. (Biostatistics)	2010

D. Other Teaching and Advising Roles

1. C Yuan, Brown master student (Biostatistics). 2013 Summer Intern at Press Ganey. My role: Curriculum Practical Training Advisor.
2. J Shangguan, Brown master student (Biostatistics). 2010 Summer Intern at Fidelity Co. My role: Curriculum Practical Training Advisor.
3. Alina Kung, Brown undergraduate student (International Relationships). Awardee of International Scholars Program Scholarship 2010. “Evaluating the Effectiveness of American Healthcare NGOs in China.” My role: Research Advisor.
4. Hongwei Xu, Brown PhD student (Sociology). Awardee of Brown University Global Health Scholarship 2009. “A Longitudinal Study of the Coexistence of Underweight and Overweight Persons within Household in China: 1997-2006.” My role: Research Advisor.

E. Junior Faculty Mentoring and Medical Consulting

1. Ed Wing, M.D. and Rena Wing, Ph.D. (2014). CFAR Developmental Grant: "An Internet-based Behavioral Weight Programs for HIV+ patients". My role: Consultant.
2. Elizabeth M. Goldberg, M.D. (2014). UEMF Research Development Grant: “Is Emergency Department Hypertension Predictive of Future Hypertension?” My role: Mentor.
3. Gary Wessel, Ph.D. (MCBB) (2013). CFAR Developmental Grant: “Metagenomic Analysis of the Gut Microbiome in the HIV Infected Patients: Prospects for Therapy.” My role: Mentor.
4. Francesca Beaudoin, M.D. (2013). NIDA Emergency Medicine Foundation. “Prescription opioid use and misuse in older emergency department patients.” My role: Mentor.
5. Francesca Beaudoin, M.D. (2013). NIH RO3: “Chronic Opioid Use, Pain Sensitivity and Acute Pain in the Emergency Department.” My role: Consultant.
6. Elkanah Omenge Orango, M.D. (2013). CFAR Supplement: “Cryotherapy and HIV Genital Shedding.” My role: Mentor.
7. Nick Zaller, Ph.D. (2013). CFAR Supplement: “HIV treatment cascade among HIV+ person in community corrections (tentative title).” My role: Consultant.
8. Elkanah Orango, MD. (2013). International CFAR Developmental Award: “Outcome of excisional treatment for cervical neoplasia in HIV infected and uninfected women in Western Kenya.” My role: Consultant.

9. Kevin Dushay, M.D. (2012). CFAR Developmental Grant: “The prevalence of obstructive airway disease and the burden of chronic respiratory symptoms in HIV subjects enrolled in care in Kenya.” My role: Consultant.
10. Rana Aadia, M.D. (2012). K-23 Award: “Improving Retention in Care Among Postpartum HIV-infected Women in Mississippi.” My role: Mentor.
11. Willoughby Britton, Ph.D. (2012). K-Award (NCCAM): “Dismantling Mindfulness: Contributions of Attention vs. Acceptance.” My role: Consultant.
12. Becky Genberg, PhD. (2012). K-Award: “Linkage to care following home-based counseling and testing for HIV in western Kenya.” My role: Consultant.
13. Michelle Mellion, MD. (2011). CFAR Developmental Award: “The effect of alcohol on CSF cytokine concentrations in patients with HIV and its association with neuro-cognitive disturbances.” My role: Consultant.
14. Philips Chan, MD. (2011). K-Award: “Molecular Epidemiology of HIV Transmission Networks”. My role: Consultant.
15. Adrian Garder, MD. (2011). CFAR Developmental Award: “Implementation of GeneXpert® for evaluation of HIV positive TB suspects previously treated for TB in Western Kenya.” My role: Consultant.
16. Ammy Nunn, PhD. (2010). K-Award: “Exploring Patterns of Sexual Concurrency Among Urban African Americans.” My role: Consultant.
17. Rosemarie Martin, PhD (2010). Developmental Award: “Contingency Management for Drug Use in HIV+ Adults after Prison or Jail Release”. My role: Consultant.
18. Philips Chan, MD. (2010). Burroughs Wellness Grant: “Association of human leukocyte antigen and the development of antiretroviral drug resistance in HIV-1 infection”. My role: Consultant
19. Erna Kojic, MD (2009). K-Award: “HPV and related diseases in HIV infected women”. My role: Consultant.
20. Michael Waxman, MD (Assistant Professor of Emergency Medicine) (2007). The Brown Department of Emergency Medicine Junior Faculty Research Award. My role: Consultant.
21. Brenna Anderson, MD (Assistant Professor of Obstetrics & Gynecology) (2007). Developmental Award: “Effect of trichomoniasis therapy on genital HIV viral burden among African women”. My role: Consultant.

SOFTWARES

1. R-package: TVLT (Targeted testing under cost constraints); alpha-version.

PROFESSIONAL SOCIETIES

1. The American Association for the Advancement of Science (AAAS).
2. International Biometric Society
3. American Statistics Association (ASA).
4. The Tau Beta Pi Honor Society.

HOBBIES

Soccer; beekeeping; carpentry; organic gardening.

Appendix 2:
Revised MB-BP Recruitment Card
with track changes

~~RESEARCH STUDY:~~

Mindfulness-Based Intervention

for Blood Pressure Reduction

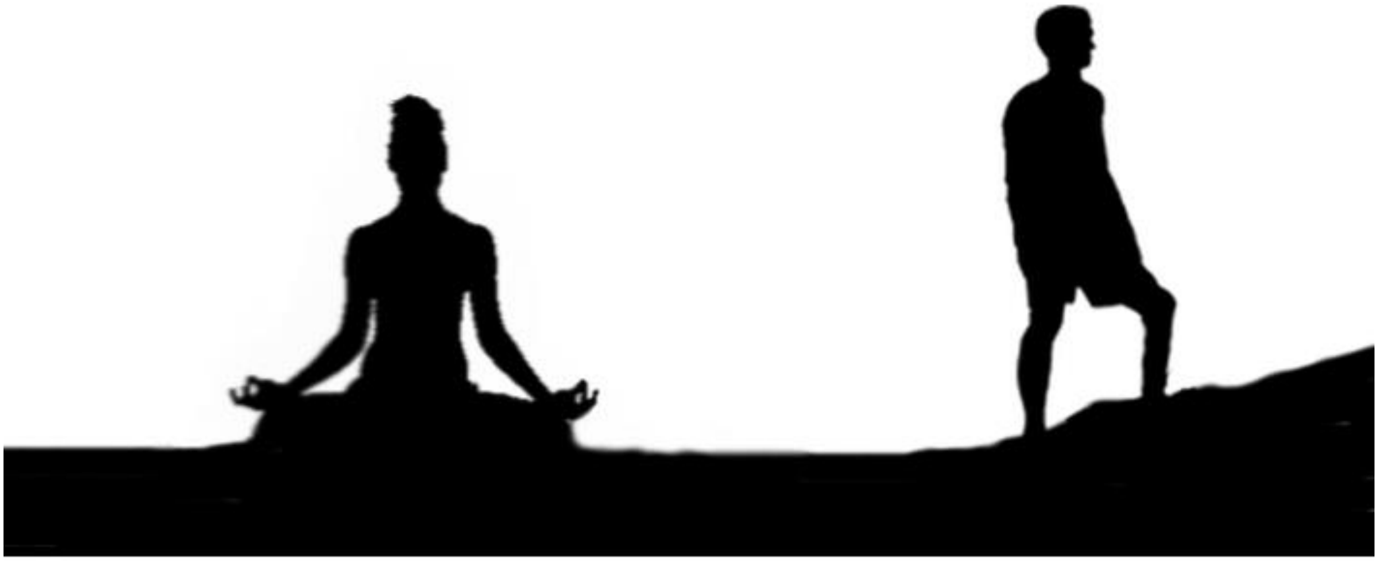

Free to qualified participants

For more information on our research study, or to see if you qualify, ~~call~~ contact the Brown University Mindfulness & Cardiovascular Health Lab at 401-~~369-0443~~400-4768 (call or text), or email: mindfulness@brown.edu.

Brown University
School of Public Health

This is a 9-week program that includes mindfulness meditation, mindful movements, education about hypertension, and support for reducing hypertension risk factors. The study is testing whether this intervention lowers blood pressure. The ~~program~~ course consists of nine weekly sessions that are 2.5 hours, ~~and~~ as well as an all-day weekend retreat. People who have a current ~~regulation~~ regular meditation practice (i.e. meditate more than once per week) are not eligible.

Research participation includes interviews, questionnaires and health measurements, such as ~~of~~ blood pressure, height, and weight~~,~~ ~~and waist circumference,~~, completed both before and after the meditation program.

Contact: For more information, or to see if you qualify, ~~call~~ contact the Brown University Mindfulness & Cardiovascular Health Lab at ~~401-369-0443~~401-400-4768 (call or text) or email at mindfulness@brown.edu.

Appendix 3:
Revised MB-BP Recruitment Card and Flyer - *clean copies*

MINDFULNESS-BASED INTERVENTION
FOR BLOOD PRESSURE REDUCTION

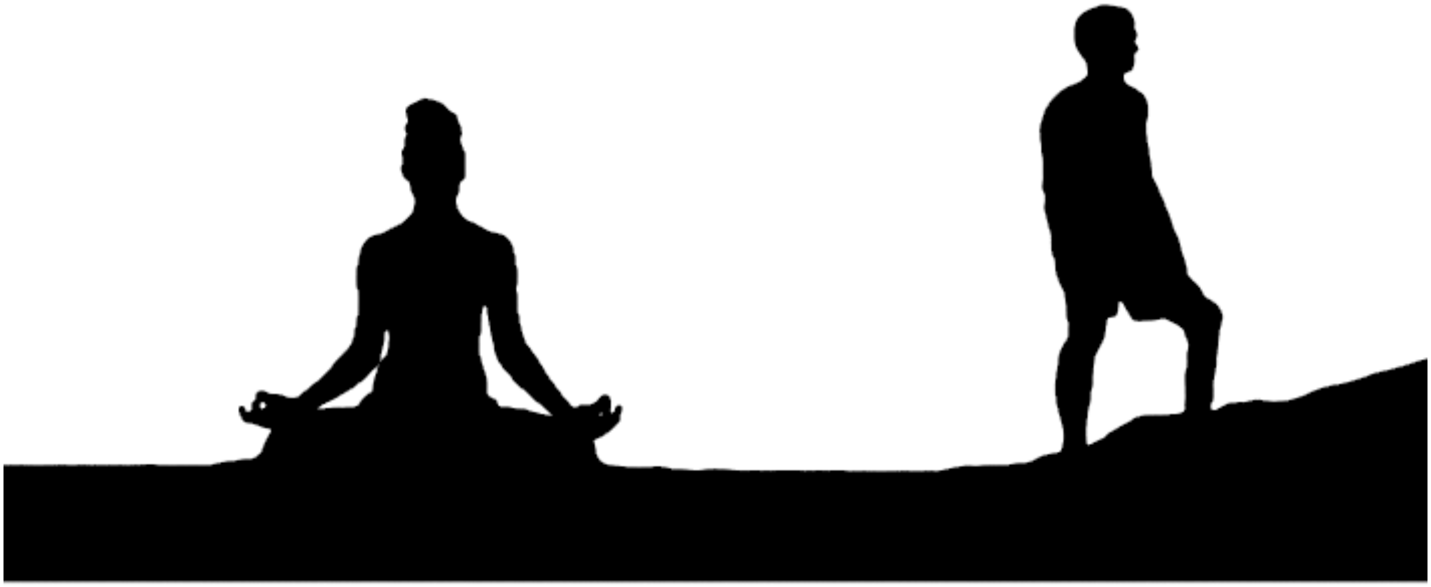

Free to qualified participants

For more information on our research study, or to see if you qualify, contact the Brown University Mindfulness & Cardiovascular Health Lab at 401-400-4768 (call or text), or email: mindfulness@brown.edu.

Brown University
School of Public Health

BROWN

This is a 9-week program that includes free training in mindfulness meditation, mindful movements, education about hypertension, and support for reducing hypertension risk factors. The study is testing whether this intervention lowers blood pressure. The course consists of nine weekly sessions that are 2.5 hours, as well as an all-day weekend retreat. People who have a current regular meditation practice (i.e. meditate more than once per week) are not eligible.

Research participation includes interviews, questionnaires and health measurements, such as blood pressure, height, and weight, completed both before and after the meditation program.

Contact: For more information, or to see if you qualify, contact the Brown University Mindfulness & Cardiovascular Health Lab at 401-400-4768 (call or text) or email at mindfulness@brown.edu.

MINDFULNESS-BASED INTERVENTION FOR BLOOD PRESSURE REDUCTION

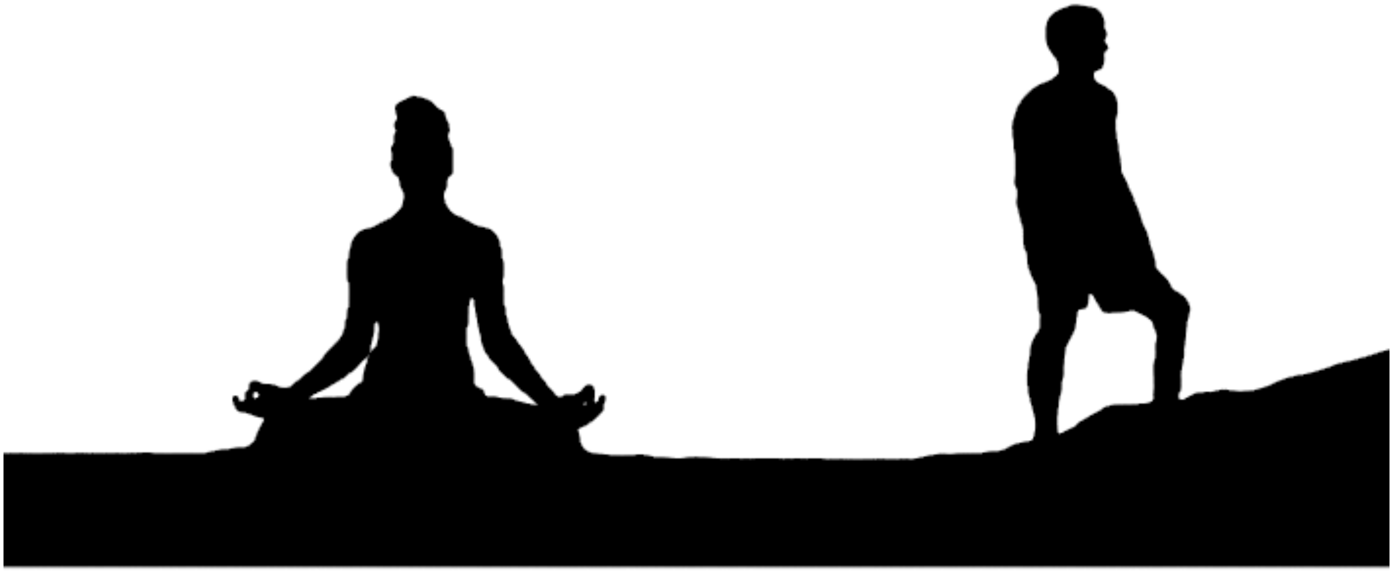

Free to qualified participants

This is a 9-week program that includes free training in mindfulness meditation, mindful movements, education about hypertension, and support for reducing hypertension risk factors. The research study is testing whether this intervention lowers blood pressure. The course consists of nine weekly sessions that are 2.5 hours, as well as an all-day weekend retreat. People who have a current regular meditation practice (i.e. meditate more than once per week) are not eligible.

Research participation includes interviews, questionnaires and health measurements, such as blood pressure, height, and weight, completed both before and after the meditation program.

Contact: For more information, or to see if you qualify, contact the Brown University Mindfulness & Cardiovascular Health Lab at: 401-400-4768 (call or text) or email: mindfulness@brown.edu.

Brown University
School of Public Health
BROWN

Mindfulness Blood Pressure Study
Call/Text: 401-400-4768
Email: mindfulness@brown.edu

Mindfulness Blood Pressure Study
Call/Text: 401-400-4768
Email: mindfulness@brown.edu

Mindfulness Blood Pressure Study
Call/Text: 401-400-4768
Email: mindfulness@brown.edu

Mindfulness Blood Pressure Study
Call/Text: 401-400-4768
Email: mindfulness@brown.edu

Mindfulness Blood Pressure Study
Call/Text: 401-400-4768
Email: mindfulness@brown.edu

Mindfulness Blood Pressure Study
Call/Text: 401-400-4768
Email: mindfulness@brown.edu

Mindfulness Blood Pressure Study
Call/Text: 401-400-4768
Email: mindfulness@brown.edu

Mindfulness Blood Pressure Study
Call/Text: 401-400-4768
Email: mindfulness@brown.edu

Mindfulness Blood Pressure Study
Call/Text: 401-400-4768
Email: mindfulness@brown.edu

Mindfulness Blood Pressure Study
Call/Text: 401-400-4768
Email: mindfulness@brown.edu

Mindfulness Blood Pressure Study
Call/Text: 401-400-4768
Email: mindfulness@brown.edu

Mindfulness Blood Pressure Study
Call/Text: 401-400-4768
Email: mindfulness@brown.edu

Appendix 4:
MB-BP Study Facebook
Page Screenshots

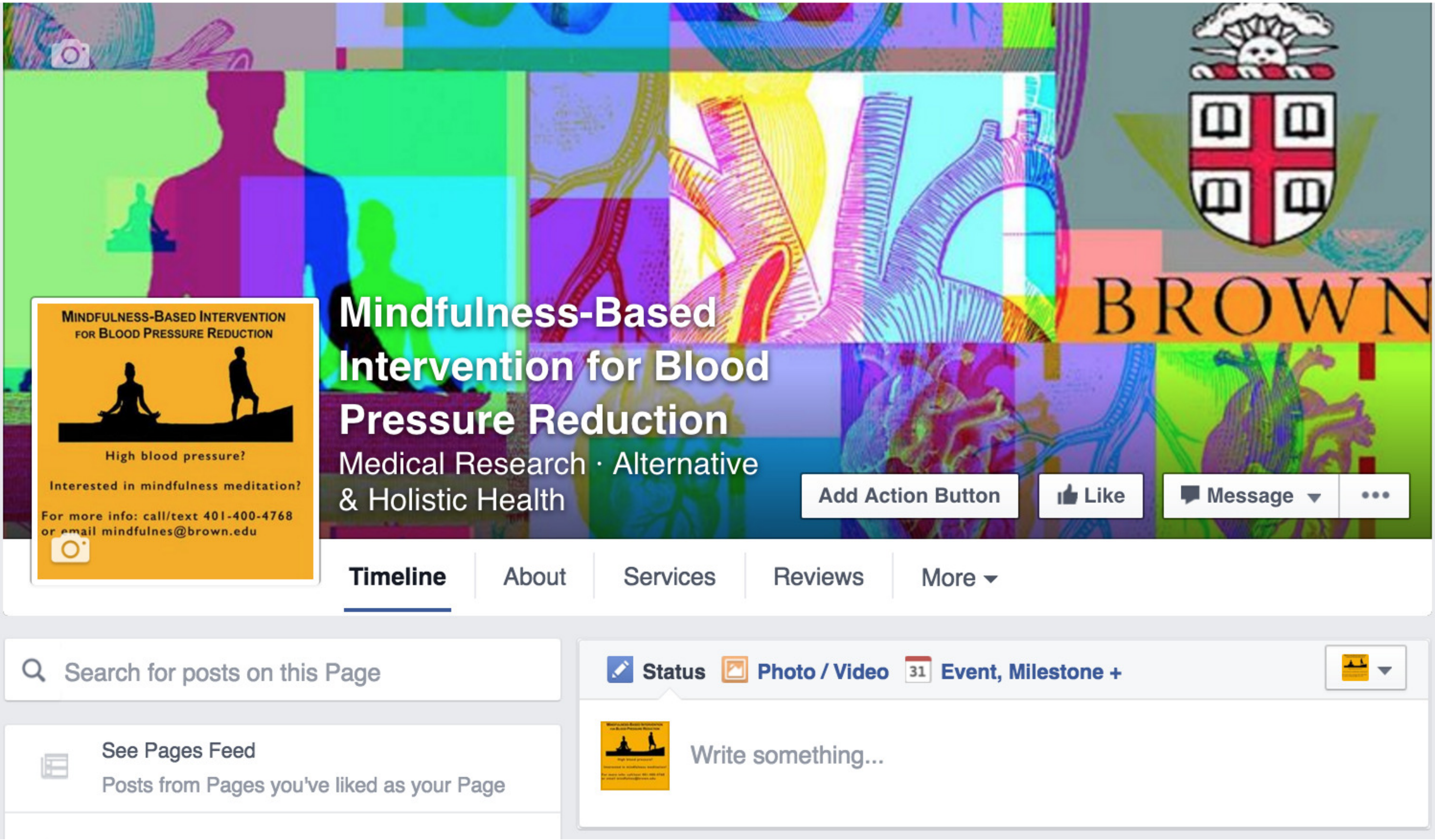
MINDFULNESS-BASED INTERVENTION FOR BLOOD PRESSURE REDUCTION
High blood pressure?
Interested in mindfulness meditation?
For more info: call/text 401-400-4768 or email mindfulnes@brown.edu
BROWN
Mindfulness-Based Intervention for Blood Pressure Reduction
Medical Research · Alternative & Holistic Health
Add Action Button
Like
Message
Timeline
About
Services
Reviews
More
Search for posts on this Page
See Pages Feed
Posts from Pages you've liked as your Page
Status
Photo / Video
31
Event, Milestone +
Write something...

See Pages Feed
Posts from Pages you've liked as your Page

Invite friends to like this Page

More Reach. Less Money.

Reach 96,000 people near Mindfulness-Based Intervention for Blood Pressure Reduction. Get started for $20.00.

Promote Local Business

ABOUT

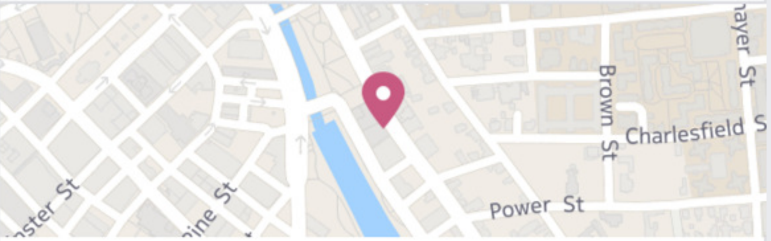

Research study looking at the effects of mindfulness meditation on blood pressure at the Brown University School of Public Health under PI Dr. Eric Loucks

121 S Main ST
Providence, RI

Save

Add phone number

http://mindfulnesscvlab.w...

Promote Website

Mindfulness-Based Intervention for Blood Pressure Reduction

Just now ·

We're enrolling participants now for our cycle beginning in June -- contact us if you're interested!

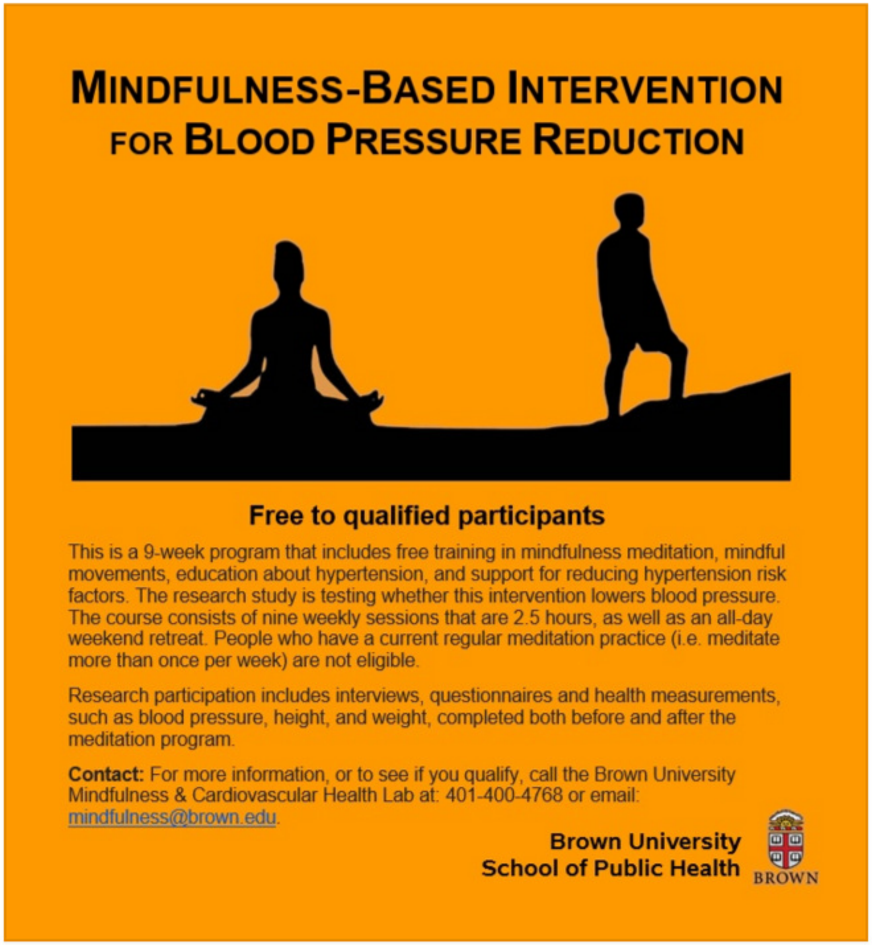

Boost Post

Long Description	The Mindfulness-Based Intervention for Blood Pressure Reduction (MBBP) is a research study currently being conducted in the Brown University School of Public Health in the Center for Population Health and Clinical Epidemiology.<br><br>Dr. Eric Loucks is the Principal Investigator of this study. As a cardiovascular physiologist and social epidemiologist, Dr. Loucks's research agenda focuses on elucidating biological mechanisms by which social and psychosocial factors may influence cardiovascular disease.	Edit

Pron

THIS

Webs

Overview

Page Info

PAGE INFO

Category	Companies & Organizations : Health/Medical/Pharmaceuticals
Name	Mindfulness-Based Intervention for Blood Pressure Reduction
Subcategories	Medical Research and Alternative & Holistic Health
Facebook Web Address	www.facebook.com/brown.mbbp
Address	121 S Main ST, Providence, Rhode Island 02912
Start Date	Enter your start date
Short Description	Studying the effects of mindfulness meditation on cardiovascular health at the Brown University School of Public Health under PI Dr. Eric Loucks
Impressum	Input Impressum for your Page
Long Description	We are in the Brown University School of Public Health, specifically in the Center for Population Health and Clinical Epidem... See More
Mission	To enable community members to partake in a Mindfulness-Based Intervention for blood pressure reduction ✎ Edit
Founded	+ Enter founding date
Awards	+ Enter awards
Safety Information	+ Enter safety information
Products	+ Enter products
Phone	Add a phone number
Email	mindfulness@brown.edu
Website	http://mindfulnesscvlab.weebly.com
Official Page	Enter the official brand, celebrity or organization your Page is about
Facebook Page ID	1121982851167360

Appendix 5:
MB-BP Study Twitter
Account Screenshots

Mindfulness-Based Blood Pressure Reduction Study – Twitter Account

Below is a screenshot from the MB-BP Twitter Page

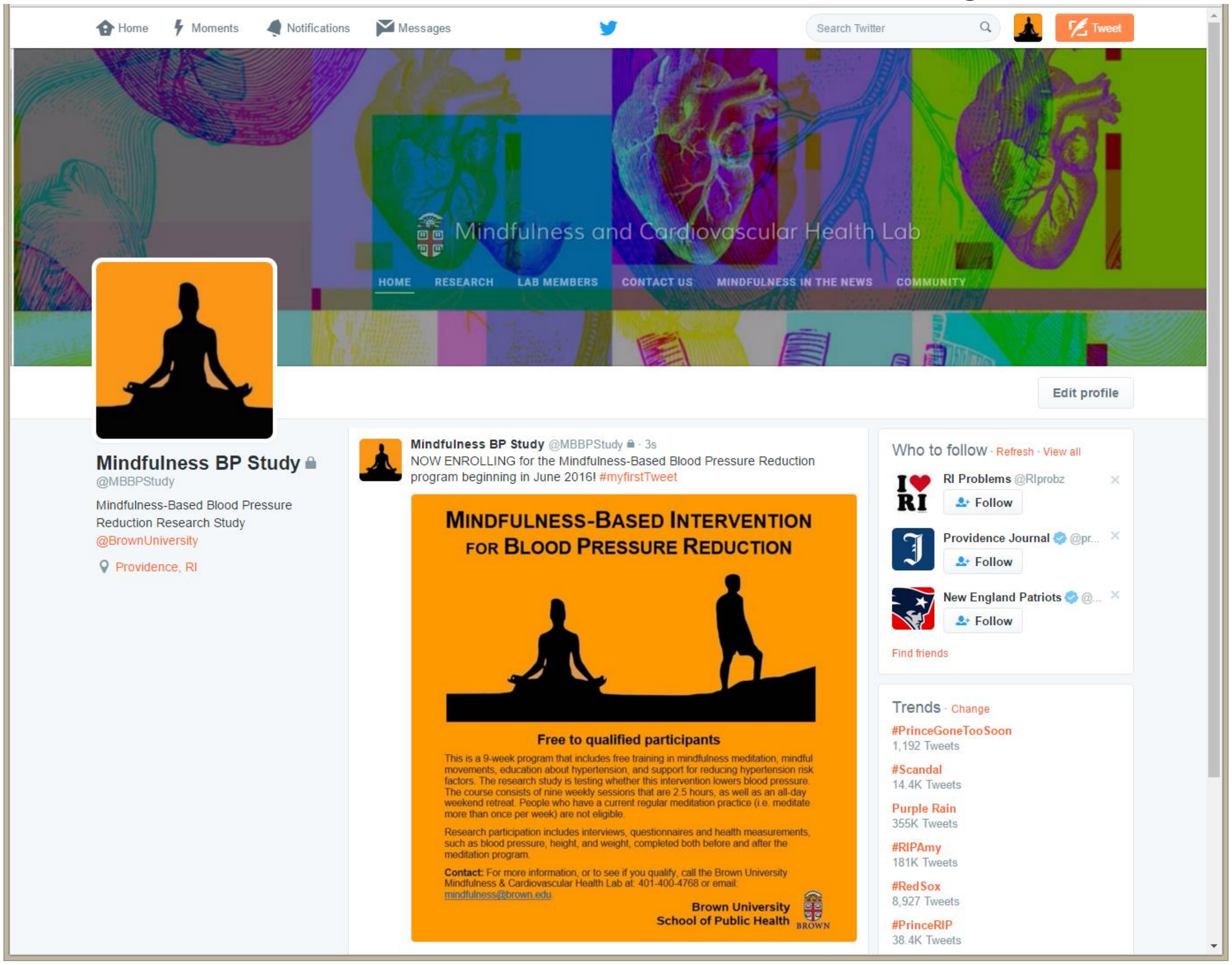

Note: that the current privacy setting is set so that no one can see the MB-BP tweets. This setting will remain in effect until IRB approval has been received.

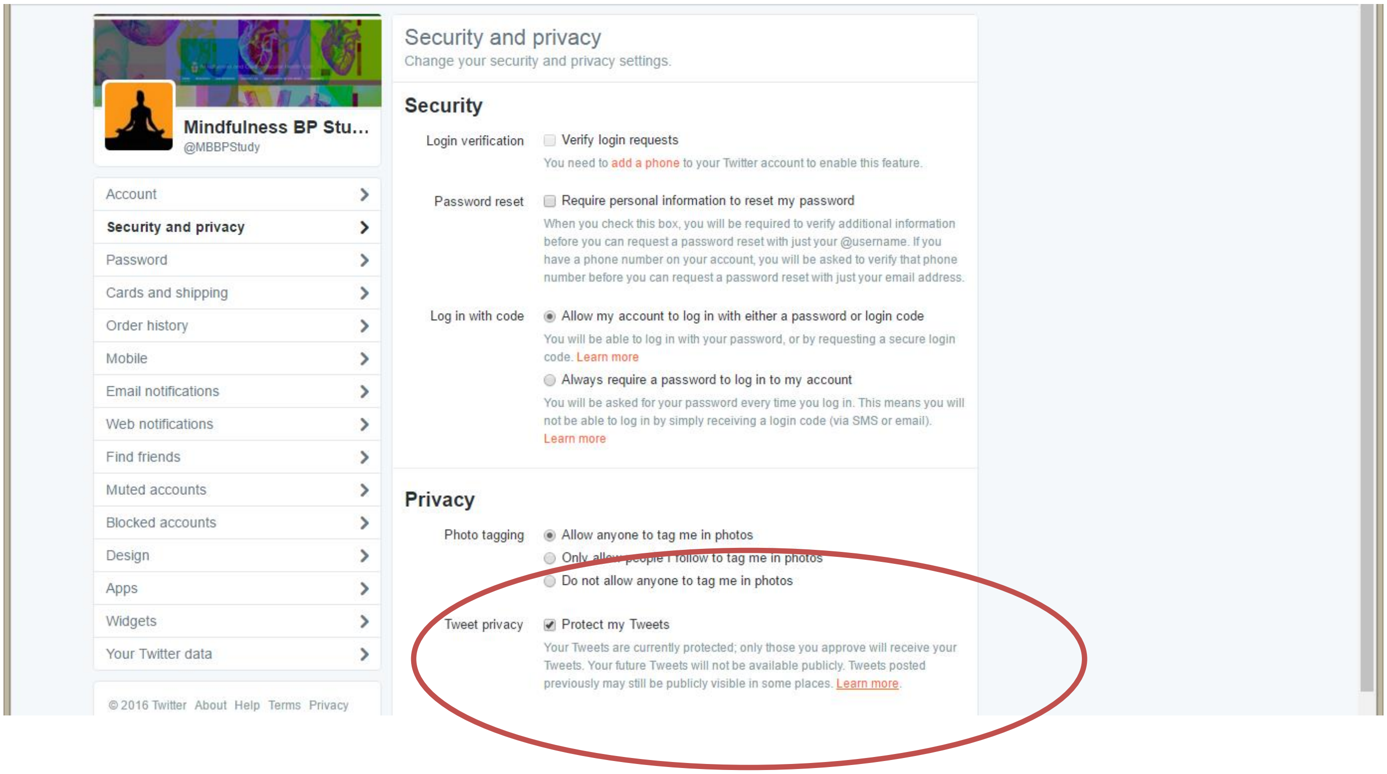

Appendix 6:
MB-BP Study Craigslist
Advertisement Screenshots

Mindfulness-Based Blood Pressure Reduction Study – Craigslist Advertisement

Screenshots of the MB-BP Craigslist Advertisement is shown below.

craigslist, Inc [US] https://post.craigslist.org/k/yC4Wqq8L5hGrjy37LOFI3A/FSTFg?s=preview

CL rhode island > jobs > et cetera > create posting

[logged in as mindfulness@brown.edu] [log out]

Description	Price
High Blood Pressure? Interested in Mindfulness meditation?	
rhode island > et cetera	$25.00 USD
**Total amount charged:**	**$25.00 USD**

Attention: Your posting will expire from the site in 30 days.

this is an unpublished draft. publish

edit post | edit location | edit images

CL rhode island > jobs > et cetera

reply Posted: less than a minute ago print

High Blood Pressure? Interested in Mindfulness meditation? (Providence)

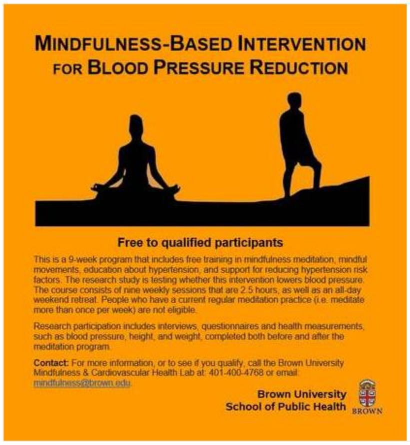

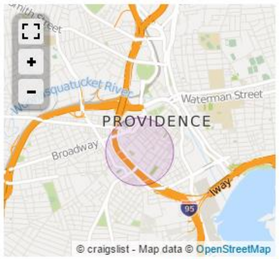

South Main Street
(google map)

compensation: **Contact us for details.**
employment type: **employee's choice**
non-profit organization

NOW ENROLLING: for the Brown University Mindfulness-Based Blood Pressure Reduction Study

This is a 9-week program that includes free training in mindfulness meditation, mindful movements, education about hypertension, and support for reducing hypertension risk factors. The study is testing whether this intervention lowers blood pressure. The course consists of nine weekly sessions that are 2.5 hours, as well as an all-day weekend retreat. People who have a current regular meditation practice (i.e. meditate more than once per week) are not eligible.

Research participation includes interviews, questionnaires and health measurements, such as blood pressure, height, and weight, completed both before and after the meditation program.

Contact: For more information, or to see if you qualify, contact the Brown University Mindfulness & Cardiovascular Health Lab at 401-400-4768 (call or text) or email at mindfulness@brown.edu.

- Principals only. Recruiters, please don't contact this job poster.
- do NOT contact us with unsolicited services or offers

posted: 2016-04-26 9:15am email to friend best of

edit post | edit location | edit images publish

Appendix 7:
MB-BP Study webpage
screenshots

Mindfulness-Based Blood Pressure Reduction Study Webpage – Brown University

Screenshots of the MB-BP study webpage are shown below.

HOME PAGE:

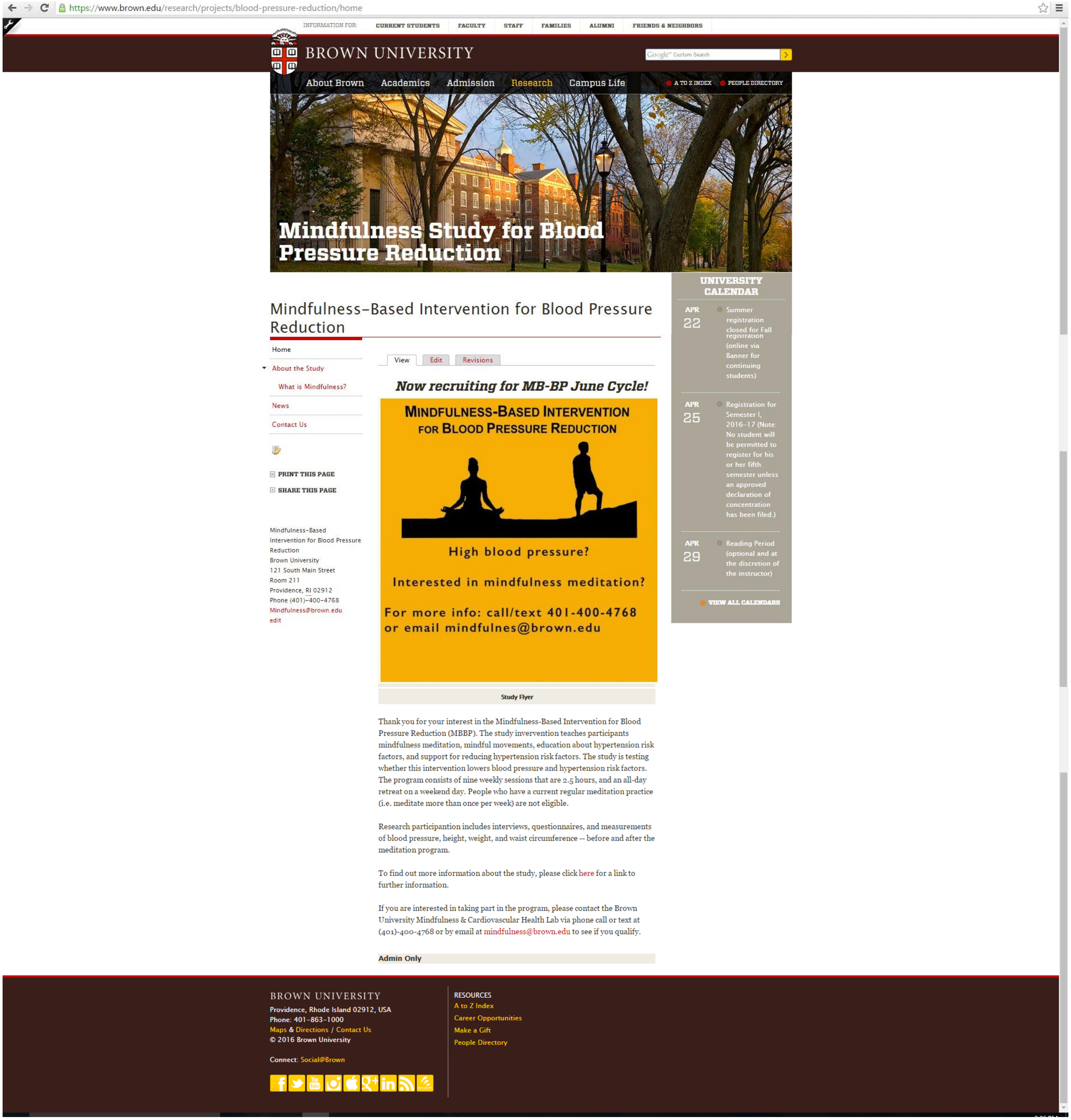
https://www.brown.edu/research/projects/blood-pressure-reduction/home

INFORMATION FOR: CURRENT STUDENTS | FACULTY | STAFF | FAMILIES | ALUMNI | FRIENDS & NEIGHBORS

BROWN UNIVERSITY

About Brown | Academics | Admission | Research | Campus Life | A TO Z INDEX | PEOPLE DIRECTORY

Mindfulness Study for Blood Pressure Reduction

Mindfulness-Based Intervention for Blood Pressure Reduction

- Home
- About the Study
  - What is Mindfulness?
- News
- Contact Us

PRINT THIS PAGE

SHARE THIS PAGE

Mindfulness-Based Intervention for Blood Pressure Reduction
Brown University
121 South Main Street
Room 211
Providence, RI 02912
Phone (401)-400-4768
Mindfulness@brown.edu
edit

View | Edit | Revisions

Now recruiting for MB-BP June Cycle!

Study Flyer

Thank you for your interest in the Mindfulness-Based Intervention for Blood Pressure Reduction (MBBP). The study invervention teaches participants mindfulness meditation, mindful movements, education about hypertension risk factors, and support for reducing hypertension risk factors. The study is testing whether this intervention lowers blood pressure and hypertension risk factors. The program consists of nine weekly sessions that are 2.5 hours, and an all-day retreat on a weekend day. People who have a current regular meditation practice (i.e. meditate more than once per week) are not eligible.

Research participantion includes interviews, questionnaires, and measurements of blood pressure, height, weight, and waist circumference -- before and after the meditation program.

To find out more information about the study, please click here for a link to further information.

If you are interested in taking part in the program, please contact the Brown University Mindfulness & Cardiovascular Health Lab via phone call or text at (401)-400-4768 or by email at mindfulness@brown.edu to see if you qualify.

Admin Only

UNIVERSITY CALENDAR

APR 22 — Summer registration closed for Fall registration (online via Banner for continuing students)

APR 25 — Registration for Semester I, 2016-17 (Note: No student will be permitted to register for his or her fifth semester unless an approved declaration of concentration has been filed.)

APR 29 — Reading Period (optional and at the discretion of the instructor)

VIEW ALL CALENDARS

BROWN UNIVERSITY
Providence, Rhode Island 02912, USA
Phone: 401-863-1000
Maps & Directions / Contact Us
© 2016 Brown University

Connect: Social@Brown

RESOURCES
A to Z Index
Career Opportunities
Make a Gift
People Directory

SUB-PAGES:

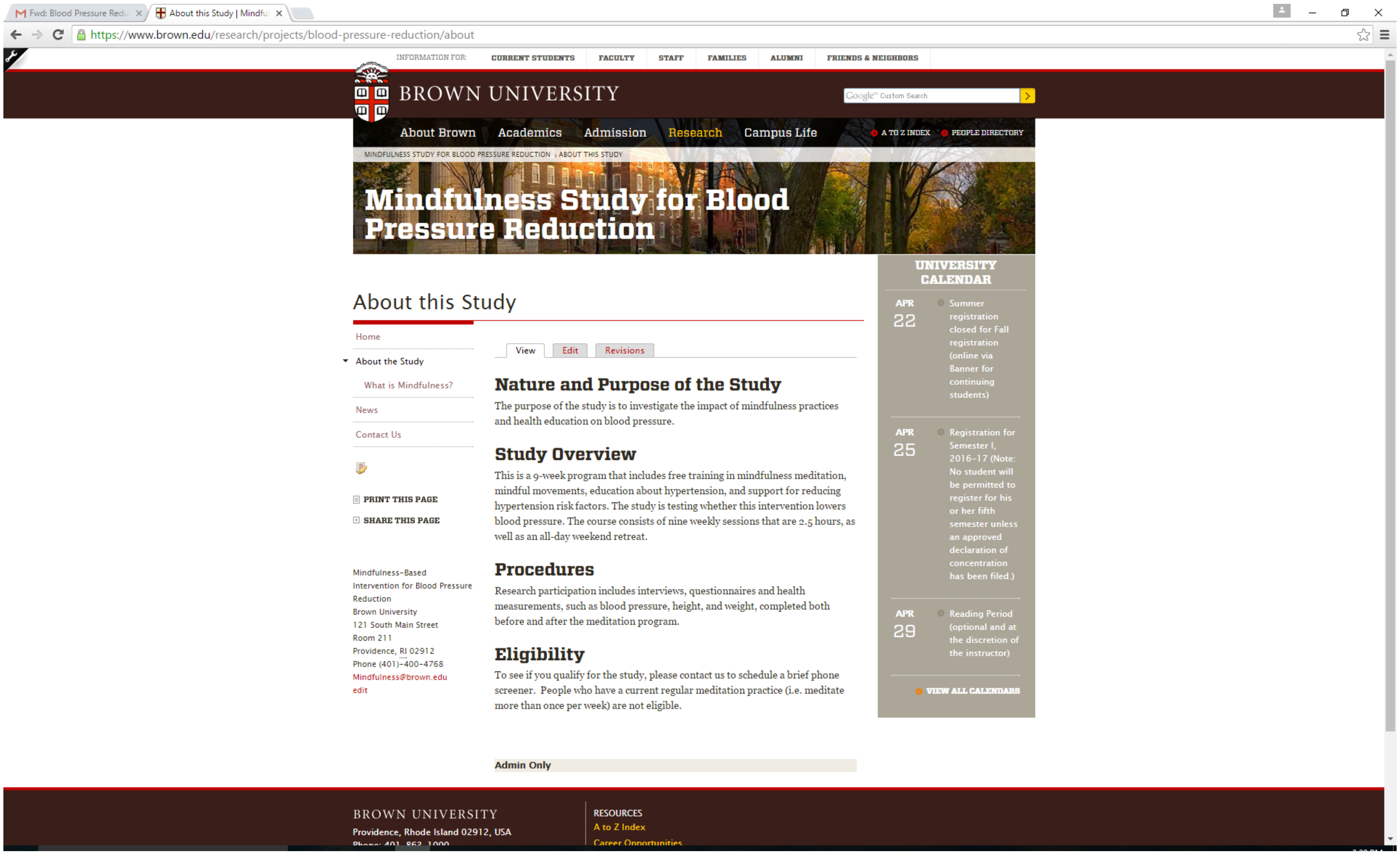

https://www.brown.edu/research/projects/blood-pressure-reduction/about

INFORMATION FOR: CURRENT STUDENTS | FACULTY | STAFF | FAMILIES | ALUMNI | FRIENDS & NEIGHBORS

BROWN UNIVERSITY

About Brown | Academics | Admission | Research | Campus Life | A TO Z INDEX | PEOPLE DIRECTORY

MINDFULNESS STUDY FOR BLOOD PRESSURE REDUCTION › ABOUT THIS STUDY

Mindfulness Study for Blood Pressure Reduction

About this Study

Home
About the Study
What is Mindfulness?
News
Contact Us

PRINT THIS PAGE
SHARE THIS PAGE

Mindfulness-Based Intervention for Blood Pressure Reduction
Brown University
121 South Main Street
Room 211
Providence, RI 02912
Phone (401)-400-4768
Mindfulness@brown.edu
edit

View | Edit | Revisions

Nature and Purpose of the Study

The purpose of the study is to investigate the impact of mindfulness practices and health education on blood pressure.

Study Overview

This is a 9-week program that includes free training in mindfulness meditation, mindful movements, education about hypertension, and support for reducing hypertension risk factors. The study is testing whether this intervention lowers blood pressure. The course consists of nine weekly sessions that are 2.5 hours, as well as an all-day weekend retreat.

Procedures

Research participation includes interviews, questionnaires and health measurements, such as blood pressure, height, and weight, completed both before and after the meditation program.

Eligibility

To see if you qualify for the study, please contact us to schedule a brief phone screener. People who have a current regular meditation practice (i.e. meditate more than once per week) are not eligible.

Admin Only

UNIVERSITY CALENDAR

APR 22 — Summer registration closed for Fall registration (online via Banner for continuing students)

APR 25 — Registration for Semester I, 2016-17 (Note: No student will be permitted to register for his or her fifth semester unless an approved declaration of concentration has been filed.)

APR 29 — Reading Period (optional and at the discretion of the instructor)

VIEW ALL CALENDARS

BROWN UNIVERSITY
Providence, Rhode Island 02912, USA

RESOURCES
A to Z Index
Career Opportunities

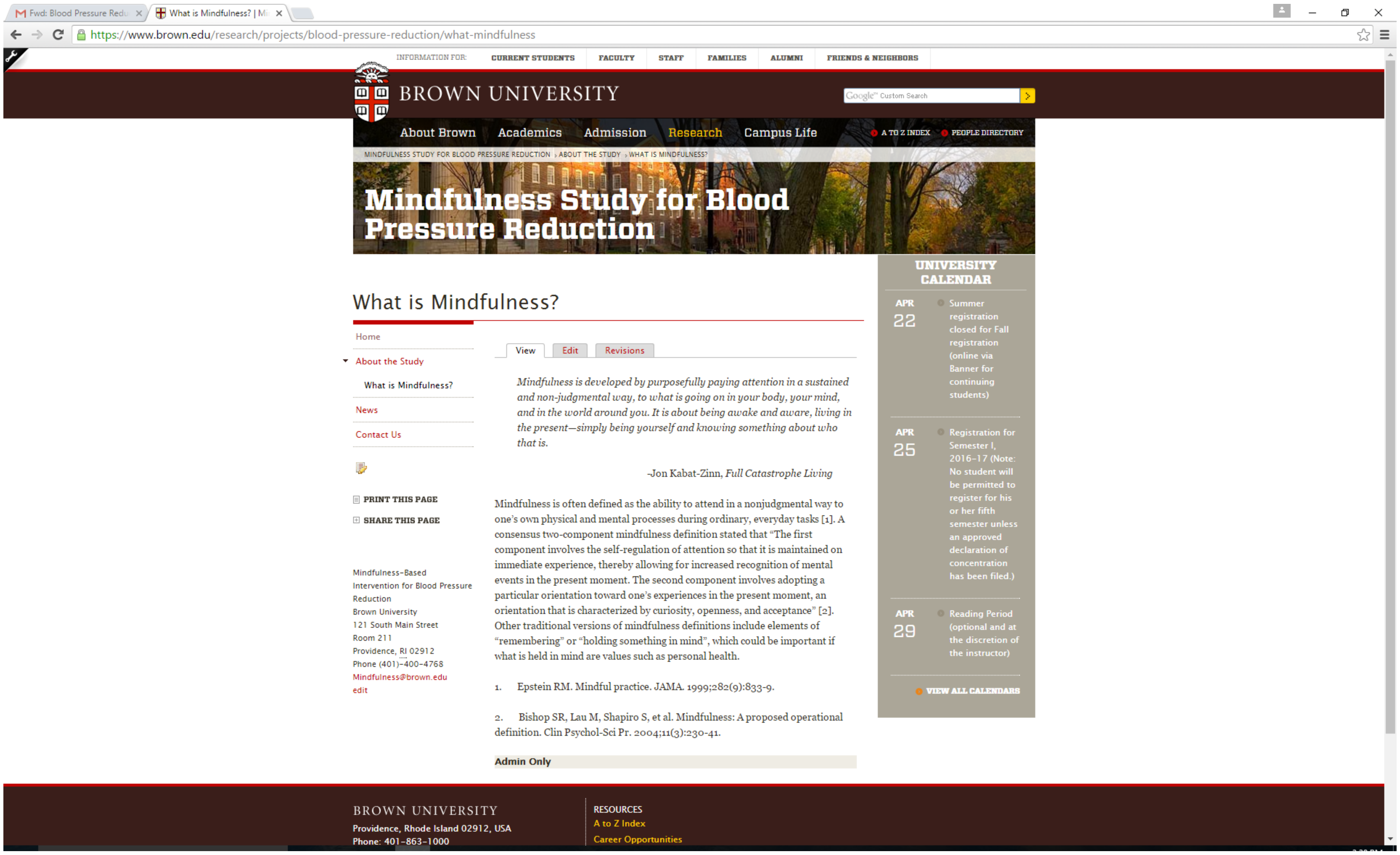

https://www.brown.edu/research/projects/blood-pressure-reduction/what-mindfulness

INFORMATION FOR: CURRENT STUDENTS | FACULTY | STAFF | FAMILIES | ALUMNI | FRIENDS & NEIGHBORS

BROWN UNIVERSITY

About Brown | Academics | Admission | Research | Campus Life | A TO Z INDEX | PEOPLE DIRECTORY

MINDFULNESS STUDY FOR BLOOD PRESSURE REDUCTION › ABOUT THE STUDY › WHAT IS MINDFULNESS?

Mindfulness Study for Blood Pressure Reduction

What is Mindfulness?

Home
About the Study
What is Mindfulness?
News
Contact Us

PRINT THIS PAGE
SHARE THIS PAGE

Mindfulness-Based Intervention for Blood Pressure Reduction
Brown University
121 South Main Street
Room 211
Providence, RI 02912
Phone (401)-400-4768
Mindfulness@brown.edu
edit

View | Edit | Revisions

Mindfulness is developed by purposefully paying attention in a sustained and non-judgmental way, to what is going on in your body, your mind, and in the world around you. It is about being awake and aware, living in the present—simply being yourself and knowing something about who that is.

-Jon Kabat-Zinn, *Full Catastrophe Living*

Mindfulness is often defined as the ability to attend in a nonjudgmental way to one's own physical and mental processes during ordinary, everyday tasks [1]. A consensus two-component mindfulness definition stated that "The first component involves the self-regulation of attention so that it is maintained on immediate experience, thereby allowing for increased recognition of mental events in the present moment. The second component involves adopting a particular orientation toward one's experiences in the present moment, an orientation that is characterized by curiosity, openness, and acceptance" [2]. Other traditional versions of mindfulness definitions include elements of "remembering" or "holding something in mind", which could be important if what is held in mind are values such as personal health.

1. Epstein RM. Mindful practice. JAMA. 1999;282(9):833-9.

2. Bishop SR, Lau M, Shapiro S, et al. Mindfulness: A proposed operational definition. Clin Psychol-Sci Pr. 2004;11(3):230-41.

Admin Only

UNIVERSITY CALENDAR

APR 22 — Summer registration closed for Fall registration (online via Banner for continuing students)

APR 25 — Registration for Semester I, 2016-17 (Note: No student will be permitted to register for his or her fifth semester unless an approved declaration of concentration has been filed.)

APR 29 — Reading Period (optional and at the discretion of the instructor)

VIEW ALL CALENDARS

BROWN UNIVERSITY
Providence, Rhode Island 02912, USA
Phone: 401-863-1000

RESOURCES
A to Z Index
Career Opportunities

CONTACT US PAGE:

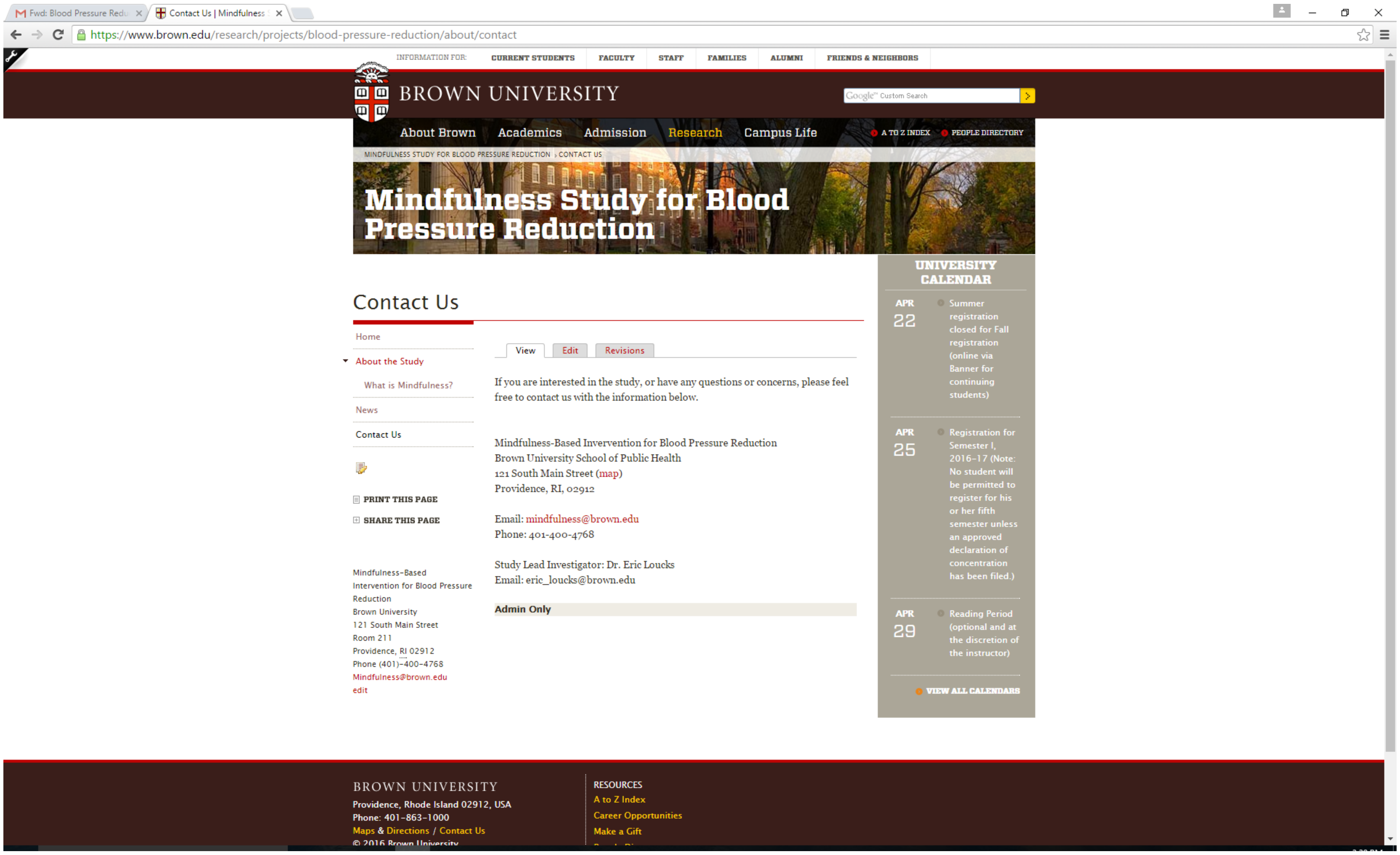

Fwd: Blood Pressure Red... | Contact Us | Mindfulness

https://www.brown.edu/research/projects/blood-pressure-reduction/about/contact

INFORMATION FOR: CURRENT STUDENTS | FACULTY | STAFF | FAMILIES | ALUMNI | FRIENDS & NEIGHBORS

BROWN UNIVERSITY

Google Custom Search

About Brown | Academics | Admission | Research | Campus Life | A TO Z INDEX | PEOPLE DIRECTORY

MINDFULNESS STUDY FOR BLOOD PRESSURE REDUCTION › CONTACT US

Mindfulness Study for Blood Pressure Reduction

Contact Us

- Home
- About the Study
  - What is Mindfulness?
- News
- Contact Us

PRINT THIS PAGE

SHARE THIS PAGE

View | Edit | Revisions

If you are interested in the study, or have any questions or concerns, please feel free to contact us with the information below.

Mindfulness-Based Inervention for Blood Pressure Reduction
Brown University School of Public Health
121 South Main Street (map)
Providence, RI, 02912

Email: mindfulness@brown.edu
Phone: 401-400-4768

Study Lead Investigator: Dr. Eric Loucks
Email: eric_loucks@brown.edu

Admin Only

Mindfulness-Based Intervention for Blood Pressure Reduction
Brown University
121 South Main Street
Room 211
Providence, RI 02912
Phone (401)-400-4768
Mindfulness@brown.edu
edit

UNIVERSITY CALENDAR

APR 22 — Summer registration closed for Fall registration (online via Banner for continuing students)

APR 25 — Registration for Semester I, 2016-17 (Note: No student will be permitted to register for his or her fifth semester unless an approved declaration of concentration has been filed.)

APR 29 — Reading Period (optional and at the discretion of the instructor)

VIEW ALL CALENDARS

BROWN UNIVERSITY
Providence, Rhode Island 02912, USA
Phone: 401-863-1000
Maps & Directions / Contact Us
© 2016 Brown University

RESOURCES
A to Z Index
Career Opportunities
Make a Gift

Mindfulness-Based Blood Pressure Reduction Study Webpage

The MB-BP study webpage is found within the Mindfulness and Cardiovascular Health Lab Website. The Mindfulness CV and Health Lab main page is shown below. Note that there is MB-BP content within the General Update section. This section can be used to draw attention to study enrollment phases.

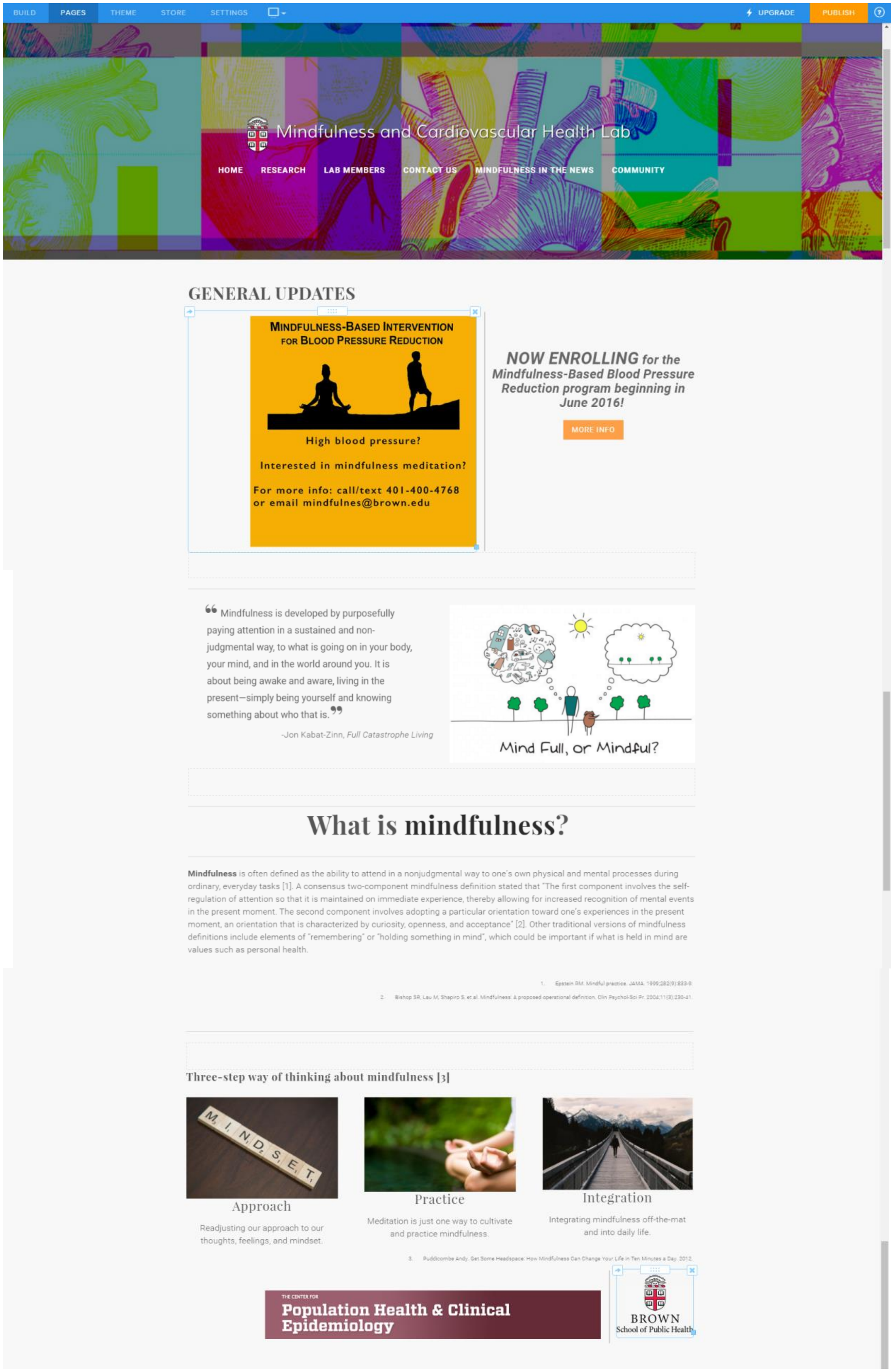

Mindfulness-Based Blood Pressure Reduction Study Webpage

The MB-BP study webpage is shown below.

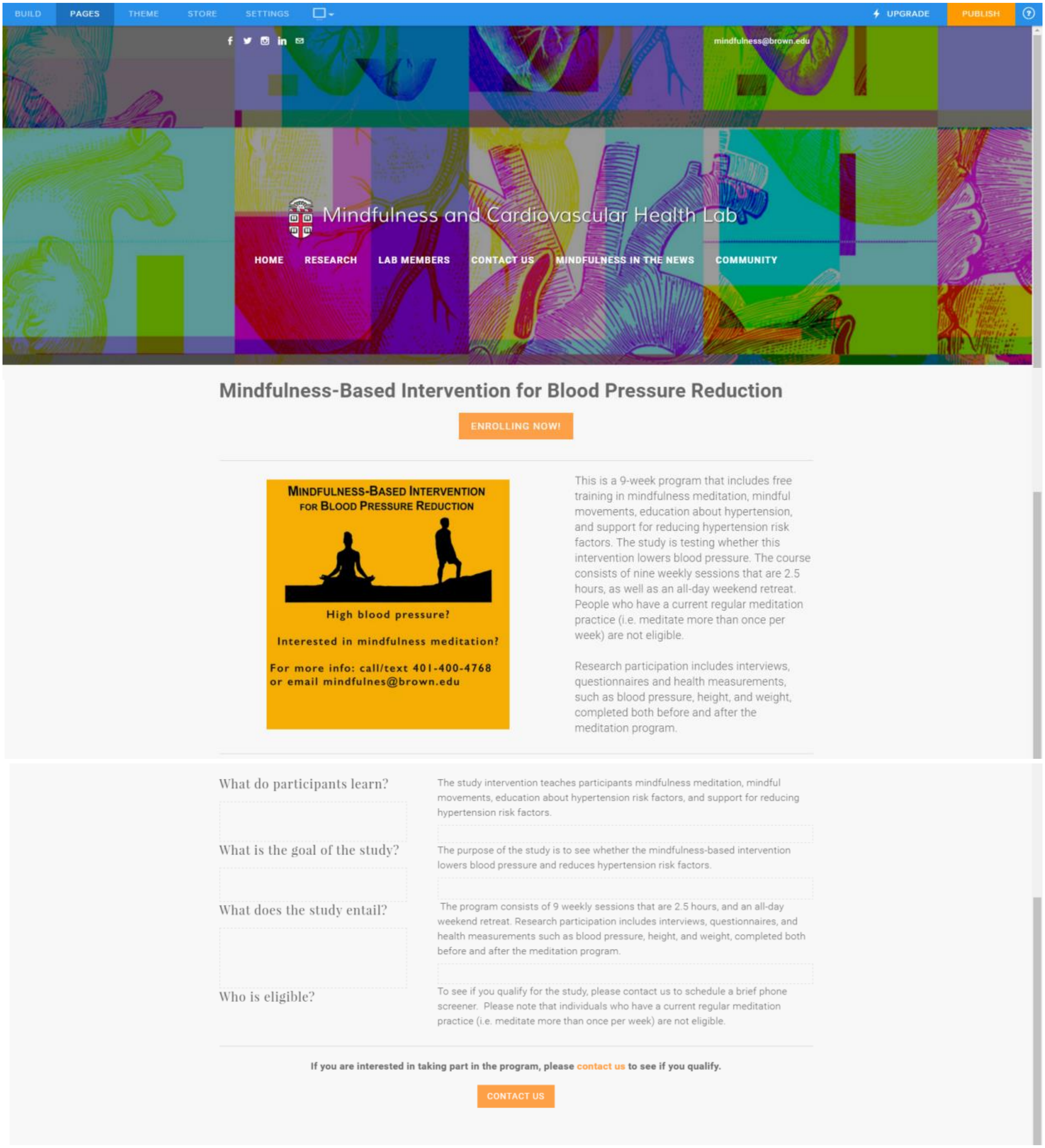

MB-BP Webpage settings are shown below:

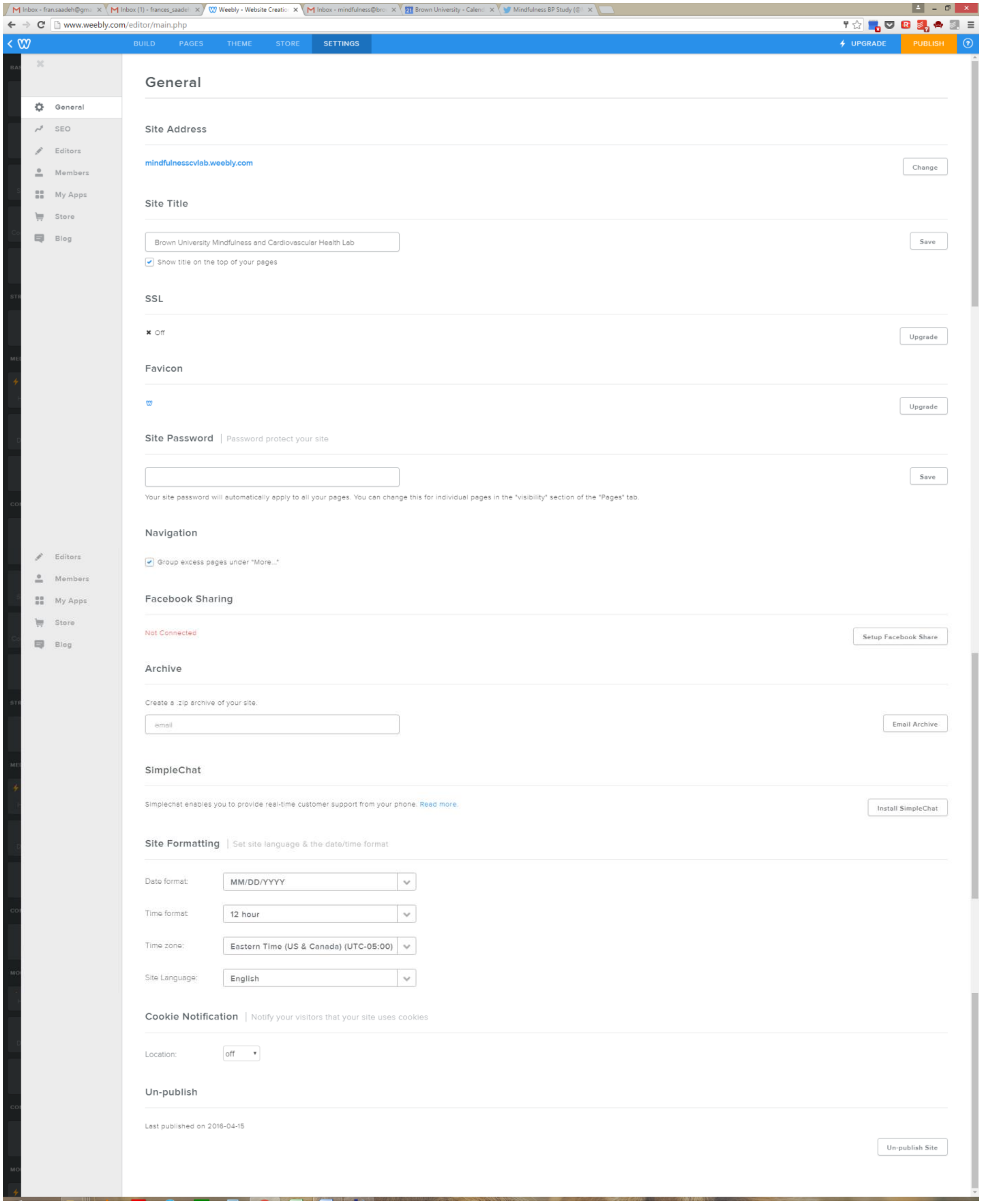

Appendix 8:

Week 4: Goal Related to Diet, Alcohol Consumption, Physical Activity, or Stress Reaction/Response - with Track Changes (v.1.1)

Participant ID#________

Week 4: Goal Related to Diet, Alcohol Consumption, Physical Activity, or Stress Reaction/Response

W4_GO_1. What is your goal related to diet, alcohol consumption, physical activity, or stress reaction/response for the week?

W4_GO_2. On a scale of 1-10, where 10 is high, how MOTIVATED are you to achieve this goal?

W4_GO_3. On a scale of 1-10, how CONFIDENT are you that you will achieve the goal?

W4_GO_4. What could you do that would bring your motivation or confidence a little higher?

W4_GO_5. What might make it difficult to achieve the goal this week, and if that happens, what will you do?

W4_GO_6. What is a way to measure this goal that resonates with you?

Deleted: *August 31, 2015*

Day/Date	Event related to goal	How did your body feel, in detail, before, during and after this experience?	What moods, feelings, and thoughts were there before, during and after this event?	How did it contribute, or not, to achieving the goal?	What thoughts are in your mind now as you write about this event?
**Mon**<br>**Date:**	W4_GO_D1_1.	W4_GO_D1_2a.<br>**Before:**<br><br>W4_GO_D1_2b.<br>**During:**<br><br>W4_GO_D1_2c.<br>**After:**	W4_GO_D1_3a.<br>**Before:**<br><br>W4_GO_D1_3b.<br>**During:**<br><br>W4_GO_D1_3c.<br>**After:**	W4_GO_D1_4.	W4_GO_D1_5.

Deleted: W4_GO_7. Goal for This Week Related to Diet, Alcohol Consumption, Physical Activity or Stress Reaction/Response:

____
____
_______________¶
____
____
_______________¶
____
____
_______________¶
¶
W4_GO_8. How Goal Will Be Measured:¶
____
____
_______________¶
____
____
_______________¶
____
____
_______________¶
¶
¶
¶

Comment [SF1]: W4_GO_7 and W4_GO_8 were removed b/c of redundancy

Deleted: *August 31, 2015*

Day/Date	Event related to goal	How did your body feel, in detail, before, during and after this experience?	What moods, feelings, and thoughts were there before, during and after this event?	How did it contribute, or not, to achieving the goal?	What thoughts are in your mind now as you write about this event?
**Tues Date:**	W4_GO_D2_1.	W4_GO_D2_2a.<br>**Before:**<br><br>W4_GO_D2_2b.<br>**During:**<br><br>W4_GO_D2_2c.<br>**After:**	W4_GO_D2_3a.<br>**Before:**<br><br>W4_GO_D2_3b.<br>**During:**<br><br>W4_GO_D2_3c.<br>**After:**	W4_GO_D2_4.	W4_GO_D2_5.
**Wed Date:**	W4_GO_D3_1.	W4_GO_D3_2a.<br>**Before:**<br><br>W4_GO_D3_2b.<br>**During:**<br><br>W4_GO_D3_2c.<br>**After:**	W4_GO_D3_3a.<br>**Before:**<br><br>W4_GO_D3_3b.<br>**During:**<br><br>W4_GO_D3_3c.<br>**After:**	W4_GO_D3_4.	W4_GO_D3_5.

Comment [SF2]: Corrected the cut and paste error on variables shown below

Deleted: 2a

Deleted: 3a

Deleted: 4

Deleted: Before:¶
¶
¶
¶
W4_GO_D2_3b.¶
During:¶
¶
¶
¶
W4_GO_D2_3c.¶
After:¶

Deleted: 2

Deleted: 2

Deleted: W4_GO_D2_1.¶

Deleted: W4_GO_D3_3a.¶
Before:¶
¶
¶
¶
W4_GO_D3_3b.¶
During:¶
¶
¶
¶
¶
W4_GO_D3_3c.¶
After:¶
...

Deleted: W4_GO_D3_4.¶

Deleted: W4_GO_D3_2a.¶
Before:¶
...

Deleted: W4_GO_D3_1.¶
¶
...

Deleted: *August 31, 2015*

Day/Date	Event related to goal	How did your body feel, in detail, before, during and after this experience?	What moods, feelings, and thoughts were there before, during and after this event?	How did it contribute, or not, to achieving the goal?	What thoughts are in your mind now as you write about this event?
**Thurs Date:**	W4_GO_D4_1.	W4_GO_D4_2a. **Before:** W4_GO_D4_2b. **During:** W4_GO_D4_2c. **After:**	W4_GO_D4_3a. **Before:** W4_GO_D4_3b. **During:** W4_GO_D4_3c. **After:**	W4_GO_D4_4.	W4_GO_D4_5.
**Fri Date:**	W4_GO_D5_1.	W4_GO_D5_2a. **Before:** W4_GO_D5_2b. **During:** W4_GO_D5_2c. **After:**	W4_GO_D5_3a. **Before:** W4_GO_D5_3b. **During:** W4_GO_D5_3c. **After:**	W4_GO_D5_4.	W4_GO_D5_5.

Deleted: W4_GO_D4_3a.¶
Before:¶
¶
¶
¶
W4_GO_D4_3b.¶
During:¶
¶
¶
¶
¶
W4_GO_D4_3c.¶
After:¶
¶
¶

Deleted: W4_GO_D4_4.¶

Deleted: W4_GO_D4_2a.¶
Before:¶
¶
¶
¶
W4_GO_D4_2b.¶
During:¶
¶

Deleted: W4_GO_D4_1.¶
¶
¶

Deleted: W4_GO_D5_3a.¶
Before:¶
¶
¶

Deleted: W4_GO_D5_4.¶

Deleted: W4_GO_D5_2a.¶
Before:¶
¶
¶

Deleted: W4_GO_D5_1.¶
¶
¶

Deleted: *August 31, 2015*

Day/Date	Event related to goal	How did your body feel, in detail, before, during and after this experience?	What moods, feelings, and thoughts were there before, during and after this event?	How did it contribute, or not, to achieving the goal?	What thoughts are in your mind now as you write about this event?
**Sat Date:**	W4_GO_D6_1.	W4_GO_D6_2a. **Before:** W4_GO_D6_2b. **During:** W4_GO_D6_2c. **After:**	W4_GO_D6_3a. **Before:** W4_GO_D6_3b. **During:** W4_GO_D6_3c. **After:**	W4_GO_D6_4.	W4_GO_D6_5.
**Sun Date:**	W4_GO_D7_1.	W4_GO_D7_2a. **Before:** W4_GO_D7_2b. **During:** W4_GO_D7_2c. **After:**	W4_GO_D7_3a. **Before:** W4_GO_D7_3b. **During:** W4_GO_D7_3c. **After:**	W4_GO_D7_4.	W4_GO_D7_5.

Deleted: W4_GO_D6_3a.¶
Before:¶
¶
¶
¶
W4_GO_D6_3b.¶
During:¶
¶
¶
¶
W4_GO_D6_3c.¶
After:¶
¶
¶

Deleted: W4_GO_D6_4.¶

Deleted: W4_GO_D6_2a.¶
Before:¶
¶
¶
¶
W4_GO_D6_2b.¶
During:¶
¶
¶ ...

Deleted: W4_GO_D6_1.¶
¶
¶

Deleted: W4_GO_D7_3a.¶
Before:¶
¶
¶ ...

Deleted: W4_GO_D7_4.¶

Deleted: W4_GO_D7_2a.¶
Before:¶
¶
¶ ...

Deleted: W4_GO_D7_1.¶
¶
¶

Deleted: *August 31, 2015*

Appendix 9:
Revised Phone Screener
with Track Changes

(v.1.5– April 14, 2016)

PHONE-DELIVERED SCREENING QUESTIONNAIRE

Deleted: MBHT

PHONE SCREENING QUESTIONNAIRE - PART 1 of 2

*The script is shown below in **bold italics.***

PID. Participant ID: ___________

SQ01. Staff ID: ______________

SQ02. Today's Date (MMDDYY): ________________

SQ03. Current Time (24 hour time, e.g. 14:45): ________

Please now call the participant.

SQ04. Was the participant reached?   YES   NO

Hello, my name is ____________. I am calling from the Brown University School of Public Health because (name of participant) expressed interest in participating in our mindfulness blood pressure study. Is he/she available to talk at this time?

I would like to do a 10-15 minute phone interview with you to determine if you are a good match for this particular study.

SQ05. ***Is now a good time to speak?***

[If yes, proceed to SQ05 script below]

If no… ***When would be a good time to talk?***

SQ06. Day to call back (DDMMYY) ____________

SQ07. Time to call back ______ AM/PM

OK, great. I'm going to give you a quick overview of the study first and can then move on to the screener to see if you may qualify. Feel free to interrupt me at any point if you have a question. Okay?

STUDY OVERVIEW: In this study, we are looking to see if mindfulness practices improve blood pressure, and if education about hypertension risk factors may also improve blood pressure. If eligible, we will provide you with training in meditation, mindful movements, and the roles of things like diet, physical activity and medication in reducing blood pressure.

You will be taught by a very experienced teacher who is an expert in these fields. The course is free and will take place over a 9-week period, where you come to a class once each week for 2.5 hours each time. There is also a one-day weekend retreat that will be 7.5 hours long. [Go over days / times of course and retreat]

As part of the project, we will also ask you to participate in health assessments before and after the study. Health assessments include measures such as blood pressure,

Deleted: *How about I*

Deleted: *answer any questions you may have to see if it is a good fit for you. We will then go through a screening questionnaire to see if you qualify for the study. Does this sound OK?*

Deleted: *on a Saturday*

Comment [SF1]: Retreat is 7.5hrs long and can be on a Saturday or a Sunday.

Deleted: *8*

Deleted: MBHT

height and weight, and questionnaires about your health and experiences. At the end of the study, you will be given $100 USD to express our gratitude. Do you have any questions about the study? [*Answer questions**]*

SQ08. ***Does this study sound like something you would be interested in doing? Yes No***

[If yes, proceed to next statement below. If no, politely thank the participant for considering being in this study, and end the call].

Great. There are a few things that I would like to go over before we start the interview. First, some of the questions that I will ask now to figure out if you are eligible to be in this study will be of personal nature, including asking about your mental health and life's experiences.

SQ09. ***Are you in a private place to talk?***

[If yes, proceed to text below. If no, reschedule meeting using variables SQ06 and SQ07 above].

Because this interview is of a personal nature, it is important that you understand that everything you say will be kept strictly confidential. No one outside of our project will ever be able to see your answers, and we will not keep your name in the same place as any of your answers. If you are not eligible after the phone screen, we will destroy your information. If you like, though, we can keep your information on file for future studies.

Ok, to begin, I am going to start by recording your name and contact information.

SQ10. Participant's First Name: ______________________________________

SQ11. Participant's Last Name:____

Participant's Address (in case we need to send any study materials to you):
SQ12a. Street address: _____________
SQ12b. City: _____________________
SQ12c: State: ________
SQ12d: Zip Code: ________________

SQ13a. Participant's Phone number #1 (in case we need to contact you by phone)
______________________________________ Type of Phone: work / home / cell / other

SQ13b. Participant's Phone number #2
______________________________________ Type of Phone: work / home / cell / other

SQ14. Participant's email address (or mailing address if no email):_______________________

SQ15. Notes from interviewer related to participants' contact information (if any):
____
____
____

Deleted: *make clear*

Deleted: *First of all, s*

Comment [SF2]: Type of phone was added so that communication with participants can be more informed.

Deleted: *MBHT*

PHONE SCREENING QUESTIONNAIRE - PART 2 of 2

PID. Participant ID #: ______________

SQ.recruit How did you find out about the study? *Select one.*

From a friend / family member / coworker .... [ ]
From a former or current participant .... [ ]
From PCP or other health care provider .... [ ]
Facebook .... [ ]
Twitter .... [ ]
Craigslist .... [ ]
Saw an orange flier / card (note location ____________) .... [ ]
Other (please describe ________________________________) .... [ ]

Comment [SF3]: For internal tracking to know how best to recruit

Comment [SF4]: Use of Facebook, Twitter, and Craigslist are subject to IRB approval

INCLUSION CRITERIA: All answers in 3rd column must be YES. If an answer is NO, immediately proceed to question SQ40.			
			Comments
SQ26.	***What is your age?*** [Is age at least 18 years?]	YES NO	
SQ27.	***Can you read and write in English?***	YES NO	

EXCLUSION CRITERIA: All answers in 3rd column must be NO with the exception of SQ32a and SQ33a. If an answer (other than SQ32a and SQ33a) is YES, then immediately proceed to question SQ40.			
			Comments
I will now start to ask some questions about your mental health.			
SQ28.	***Has anyone ever told you that you have bipolar disorder or manic depression?***	**YES NO**	
SQ29.	***Has anyone ever used the word "Borderline" to describe you?***	**YES NO**	
SQ30a.<br><br>SQ30b.	***Have you ever had a hallucination or seen things that other people can't see, or hear things other people can't hear?***<br><br>***Have you ever been diagnosed with schizophrenia or psychosis?***	**YES NO**<br><br>**YES NO**	
 <br>SQ31_1<br>SQ31_2<br>SQ31_3<br>SQ31_4<br>SQ31_5<br>SQ31_6<br>SQ31_7<br>SQ31_8	***Have you ever taken any of the following medications that I am about to read to you?***<br>***Lithium***<br>***Seroquel (quetiapine)***<br>***Abilify (aripiprazole)***<br>***Zyprexa (olanzapine)***<br>***Clozaril (clozapine)***<br>***Haldol/Haloperidol***<br>***Geodon (ziprasidone)***<br>***Risperdal (risperidone)***	 <br>**YES NO DK**<br>**YES NO DK**<br>**YES NO DK**<br>**YES NO DK**<br>**YES NO DK**<br>**YES NO DK**<br>**YES NO DK**<br>**YES NO DK**	

Deleted: *MBHT*

SQ32a	***Have you ever had a suicide attempt?***	**YES NO**	
SQ32b	***[If yes, ask…] Have you considered killing yourself during the past month?***	**YES NO**	
SQ32c	***[If yes, ask…] Are you currently suicidal?*** ***[If yes, keep participant on the phone, and follow suicide safety plan below]***	**YES NO**	
SQ32d	***[If no, ask…] Are you getting any help for that? If not, then provide list of resources from Safety Plan including Gateway, Anchor and The Providence Center] Not urgent, but inform Dr. Flynn about what was discussed.***	**YES NO**	

EXCLUSION CRITERIA: All answers in 3rd column must be NO with the exception of SQ32a and SQ33a. If an answer (other than SQ32a and SQ33a) is YES, then immediately proceed to question SQ40.

SQ33a	***Would you say you have a trauma history? [If yes…]***	**YES NO**	
SQ33b	***In the past month, have you had any problems with dissociation (memory loss)?***	**YES NO**	
SQ33c	***In the past month, have you had any flashbacks (i.e. sudden and disturbing vivid memory) about the trauma?***	**YES NO**	
SQ34a	***In the past month, have you had any problems with obsessions or compulsions, such as washing your hands or checking the oven over and over again?***	**YES NO**	
SQ34b	***[If yes…] Has anyone diagnosed you with obsessive compulsive disorder?***	**YES NO**	
SQ35	***In the past month, have you had a panic attack (i.e. sweating, heart palpitations, nausea, trouble breathing, fear of dying/choking/going crazy)?***	**YES NO**	
SQ36	***Have you had any problems with alcoholism or drug use in the past year?***	**YES NO**	
SQ37	***In the past year, have you had an eating disorder, such as starving, binge eating, or vomiting?***	**YES NO**	
SQ38a	***Do you currently have a mindfulness practice, such as meditation or yoga?***	**YES NO**	
SQ38b	***If Yes –*** ***Please tell me more about your mindfulness practice, including how often you practice per week.***	**Fill in response in comments section to**	

Deleted: MBHT

		the right	
SQ38c	***Do you currently practice meditation more than once per week? (yoga does not count as meditation in this context)***	**YES NO**	
SQ39	***This class will take place at Brown University in-person. Do you have any medical or mobility issues that would affect you being able to attend class?***	**YES NO**	
SQ40	Participant qualifies for next step of study (next step is 1st blood pressure screening)	**YES NO**<br><br>**If YES go to SQ41.**<br><br>**If NO, go to SQ42.**	

SQ41a. ***Thank you for taking the time to answer these questions. You qualify for the next stage of screening, which is to take your blood pressure at our office. If you're still interested, we'd like to schedule a time to have you come in to complete the in-person screener. It will only take 30 minutes or less and will involve taking your blood pressure 3-5 times, as well as measuring your height and weight. Is there a day or time that works best for you?*** *[Schedule the In-Person Screening visit now]*

Comment [SF5]: These measures were moved to the In-Person screener from the In-Person Baseline.

SQ41b. ***As I mentioned earlier, there is a mindfulness intervention that is part of this study. If you are eligible based on the In-Person screening, you will then be invited to take part in a 9 week Mindfulness Course. We will be scheduling the mindfulness intervention at a time that works best with most of the participants. What days and times of the week typically work best with your schedule? Please keep in mind that the sessions are 2.5 hours long, and take place once per week for 9 weeks.***

[Record participant's availability for Intervention and then proceed with the script]

Thank you for your time and interest in this study. We look forward to meeting you in person on [DATE / TIME]. Do you have any questions before we end this call?

IF INELIGIBLE:

SQ42a***. Thank you for taking the time to answer these questions. According to the survey, you do not qualify for the study at this time. There may be other studies you quality for.***

SQ42b. ***Would you like me to keep your information to pass on to these studies?*** YES / NO

SQ42c. [If yes…] ***OK, thank you. We will keep this information for future studies you may qualify for. Thank you for taking the time to talk with me today. Can I answer any questions before hanging up?***

Deleted: *MBHT*

SQ42d. [If no…] ***OK, our copy of this information will be destroyed. Thank you for taking the time to talk with me today. Can I answer any questions before hanging up?***

Deleted: *MBHT*

SAFETY PLAN

Enter details below on paper during screening (These variables should are also be entered in the survey via questions SQ10-SQ13). Destroy this paper after screening is complete.

Participant's First Name: ______________________________________

Participant's Last Name:____

Participant's Address:

Street address: _____________

City: _____________________

State: ________

Zip Code: ________________

Participant's Phone number #1: ______________________________________

Participant's Phone number #2______________________________________

During the phone-based screening, if participants respond yes to "Are you currently suicidal?", the interviewer should perform the following 2 steps:

1. **Immediately have 911 and Dr. Ellen Flynn called by a colleague who has been informed beforehand that this is a possibility.**

Specifically, while keeping the participant on the phone, show the text below in the box to a colleague.

I have a study participant on the phone who is currently suicidal. Please call 911 immediately, and tell them: <br>***"I am calling on behalf of [my name] who is performing a research study at Brown University. He has a participant on the phone who says they are currently suicidal." Please provide the participants' contact information to the 911 operator (i.e. name, address, phone #, email address) as requested. This information is shown above.*** <br>***Finally, after calling 911, please call Dr. Ellen Flynn, who is the psychiatrist supporting this study. Her cell phone # is 401-258-9829. Please provide her with the same information as was done during the 911 call.***

2. **While speaking calmly with the participant, let them know what you are doing. Specifically, let them know we are calling our study's psychiatrist Dr. Flynn and 911, and why you are doing that (i.e. because we are concerned about you). You can speak with participant to keep him/her on the phone, but the discussion should not be clinical in nature.**

Deleted: *MBHT*

Examples of questions that could be asked in order to keep them on the phone:

- "Tell me what is going on."
- "What's happening right now?"
- Tell me more about why you are interesting in being part of this study.
- What are you hoping to get out of this study?

The following information can be provided to study participants if they state they have had considered killing themselves in the past month. If they are currently suicidal, the main priority is to keep them on the phone while 911 and Dr. Flynn are being contacted.

National Suicide Prevention Lifeline: 1-800-273-8255

Other options are to:

- Call your doctor's office
- Call 911 for emergency services
- Go to the nearest hospital emergency room.

Local Non-Urgent Free or Inexpensive Mental Health Services:
Gateway Healthcare: 401-729-8701
Anchor Counseling Center: 401-475-9979
The Providence Center: 401-276-4020

Deleted: *MBHT*

Appendix 10:
Revised In-Person Screener
with Track Changes

(v.1.5– April 14, 2016)

IN-PERSON SCREENING

ASSESSMENT FORM

Deleted: *MBHT*

Deleted: *December 21, 2015*

PID. Participant ID # ___________
BA01. Staff ID # ___________
BA02. Today's date (MMDDYY): ___________

Blood Pressure:

BA03a. Blood pressure 1st reading, systolic blood pressure: _________ mmHg
BA03b. Blood pressure 1st reading, diastolic blood pressure: __________ mmHg
BA03c. Blood pressure 2nd reading, systolic blood pressure: _________ mmHg
BA03d. Blood pressure 2nd reading, diastolic blood pressure: __________ mmHg
BA03e. Blood pressure 3rd reading, systolic blood pressure: _________ mmHg
BA03f. Blood pressure 3rd reading, diastolic blood pressure: __________ mmHg

BA04. Were the 2nd and 3rd systolic blood pressure readings within 20 mmHg of each other?
□ Yes
□ No ***(repeat measurements)***

BA05. Were the 2nd and 3rd diastolic blood pressure readings within 10 mmHg of each other?
□ Yes
□ No ***(repeat measurements)***

BA06a. Repeated blood pressure 1st reading, systolic blood pressure: _________ mmHg
BA06b. Repeated blood pressure 1st reading, diastolic blood pressure: __________ mmHg
BA06c. Repeated blood pressure 2nd reading, systolic blood pressure: _________ mmHg
BA06d. Repeated blood pressure 2nd reading, diastolic blood pressure: _________ mmHg
BA06e. Repeated blood pressure 3rd reading, systolic blood pressure: _________ mmHg
BA06f. Repeated blood pressure 3rd reading, diastolic blood pressure: __________ mmHg

Blood Pressure Safety Protocol – The out-of-range blood pressure values are as follows: systolic blood pressure >200 mmHg or <90 mmHg; diastolic blood pressure >110 mmHg.

In absence of symptoms (chest pain, shortness of breath, dizziness, headache), for a SBP>200 or DBP>110 or both, we will strongly encourage participants to see their doctor right away or to go to urgent care. If there are symptoms, we will immediately call 911.

In absence of symptoms (chest pain, shortness of breath, dizziness, passing out), for a SBP<90, we will strongly encourage participants to see their doctor right away or to go to urgent care. If there are symptoms, we will immediately call 911.

BA07. Blood pressure cuff size used: □ S □ Reg □ L □ XL

BA08. Arm that cuff was placed on: □ L □ R

Comment [SF1]: Variable numbering was off. Should be "BA##" not "BA02_##". Changed for all.

Deleted: 2_

Comment [SF2]: This is part of our Safety Monitoring Plan, which is also reported to the NCCIH

Deleted: *MBHT*

Deleted: *December 21, 2015*

ELIGIBILITY OUTCOME - based on the average of the 2nd and 3rd BP (repeat) readings, note the participant's eligibility below and read the corresponding script:

Not Eligible: mean systolic BP < 120mmHg AND mean diastolic BP < 80mmHg

Unfortunately, at this time you are not eligible for the study. *If participant is interested, we can keep his/her contact information on file for future studies. END ASSESSMENT.*

Eligible: mean systolic BP ≥ 120mmHg OR mean diastolic BP ≥ 80mmHg

Congratulations, you are eligible for the study! At this time we would like to take your height and weight, ask a few follow up questions and go over the next steps of the study. *Note that if the participant does not wish to continue, thank the participant for his/her time and then END THE ASSESSMENT. Otherwise, continue below.*

BA09. Height: _______ . __ cm (one decimal place)

BA10. Weight: _______ . __ lbs (one decimal place)

BA11. What is your date of birth? ______/______/______ *[mm/dd/yyyy]*

BA12. How old are you?: _______ years

BA13. *Is participant male or female?* □ Male □ Female □ Unknown

BA14. Do you currently take any prescription medications to reduce blood pressure?

□ Yes – *go over eCAP protocol; equipment will be given to participant at baseline*

□ No

Go over the next phase of the study, including discussing the Fitbits and eCAPs (if applicable). Before participant leaves, schedule the in-person baseline assessment (must be within 1-3 weeks of the scheduled intervention) or have the Project Coordinator follow up to schedule.

END ASSESSMENT

Comment [SF3]: Scripts were added for ease of administration. The actual eligibility criteria has NOT been changed.

Comment [SF4]: Height and weight were moved here from the In-Person Baseline assessment. These variables are needed in order to set up the Fitbit equipment, which is given to participant at baseline.

Comment [SF5]: This information is needed to set up the Fitbit and eCAP equipment. This was moved here from the In-Person Baseline so that the equipment could be set up prior to the baseline assessment rather than during the assessment in order to save the participant time at baseline.

Deleted: *MBHT*

Deleted: *December 21, 2015*

Appendix 11:
Revised In-Person Baseline Assessment with Track Changes

(v.1.5– April 22, 2016)

IN-PERSON BASELINE

QUESTIONNAIRE AND ASSESSMENT FORMS

Deleted: MBHT

Deleted: December 21, 2015

PID. Participant ID # ___________
BA01. Staff ID # ___________
BA02. Today's date (MMDDYY): ___________

Blood Pressure:
BA03a. Blood pressure 1st reading, systolic blood pressure: __________ mmHg
BA03b. Blood pressure 1st reading, diastolic blood pressure: ___________ mmHg
BA03c. Blood pressure 2nd reading, systolic blood pressure: __________ mmHg
BA03d. Blood pressure 2nd reading, diastolic blood pressure: ___________ mmHg
BA03e. Blood pressure 3rd reading, systolic blood pressure: __________ mmHg
BA03f. Blood pressure 3rd reading, diastolic blood pressure: ___________ mmHg

BA04. Were the 2nd and 3rd systolic blood pressure readings within 20 mmHg of each other?
□ Yes
□ No ***(repeat measurements)***

BA05. Were the 2nd and 3rd diastolic blood pressure readings within 10 mmHg of each other?
□ Yes
□ No ***(repeat measurements)***

BA06a. Repeated blood pressure 1st reading, systolic blood pressure: __________ mmHg
BA06b. Repeated blood pressure 1st reading, diastolic blood pressure: ___________ mmHg
BA06c. Repeated blood pressure 2nd reading, systolic blood pressure: __________ mmHg
BA06d. Repeated blood pressure 2nd reading, diastolic blood pressure: ___________ mmHg
BA06e. Repeated blood pressure 3rd reading, systolic blood pressure: __________ mmHg
BA06f. Repeated blood pressure 3rd reading, diastolic blood pressure: ___________ mmHg

Blood Pressure Safety Protocol – The out-of-range blood pressure values are as follows: systolic blood pressure >200 mmHg or <90 mmHg; diastolic blood pressure >110 mmHg.

In absence of symptoms (chest pain, shortness of breath, dizziness, headache), for a SBP>200 or DBP>110 or both, we will strongly encourage participants to see their doctor right away or to go to urgent care. If there are symptoms, we will immediately call 911.

In absence of symptoms (chest pain, shortness of breath, dizziness, passing out), for a SBP<90, we will strongly encourage participants to see their doctor right away or to go to urgent care. If there are symptoms, we will immediately call 911.

BA07. Blood pressure cuff size used: □ S □ Reg □ L □ XL

BA08. Arm that cuff was placed on: □ L □ R

BA10. Weight: _______ . __ lb (one decimal place)

Comment [SF1]: This is part of our Safety Monitoring Plan, which is also reported to the NCCIH

Comment [SF2]: Height was moved to the In-Person Screener.

Deleted: BA09. Height: _______ . __ cm (one decimal place)

Comment [SF3]: Weight is measured in pounds not kilograms on our study scale.

Deleted: kg

Comment [SF4]: Waist Circumference was dropped as a measure due to too much variability in measurement and to reduce participant burden.

Deleted: Waist Circumference:

Deleted: ¶
BA11a. Measurement 1 _______ . __ cm (one decimal place)¶
BA11b. Measurement 2 _______ . __ cm (one decimal place)¶
¶
BA12. Is the difference between Measurement 1 and Measurement 2 greater than 1.0cm? ¶
□ Yes ***(repeat measurements 3&4)*** ¶
□ No¶
¶
BA13a. Measurement 3 _______ . __ cm ¶
BA13b. Measurement 4 _______ . __ cm¶

Deleted: *MBHT*

Deleted: *December 21, 2015*

Medications (ME)

ME01. Do you take any prescription medications or over-the-counter drugs?

- ☐ No → ***skip to end of medications questions***
- ☐ Yes
- ☐ Don't know
- ☐ Prefer not to answer

ME02a. What is the name of the first prescription medication or over-the-counter drug that you take?

☐ Label product name: ____

☐ Label generic name: ____

☐ Don't know ☐ Prefer not to answer

ME02b. What is the dosage form?

Oral
- ☐ Pill, tablet, or capsule
- ☐ Sublingual or orally-disintegrating tablet
- ☐ Liquid solution or suspension (drink, syrup)
- ☐ Powder

Topical
- ☐ Liquid, cream, gel, or ointment
- ☐ Ear drops (otic)
- ☐ Eye drops (ophthalmic)
- ☐ Skin patch (transdermal)

Inhaled
- ☐ Inhaler or nebulizer

Injected
- ☐ Injection

Suppository
- ☐ Rectal (e.g., enema)
- ☐ Vaginal (e.g., douche, pessary)

Other:
- ☐ Don't know
- ☐ Prefer not to answer

ME02c. How frequently do you take it?

- ☐ _________ times per day
- ☐ _________ times per week
- ☐ _________ times per month
- ☐ Don't know
- ☐ Prefer not to answer

Deleted: MBHT
Deleted: December 21, 2015

ME02d. What is the strength? ***(Record strength of how it is actually taken, not how it is prescribed.)***

- ☐ _________ %
- ☐ _________ mg
- ☐ _________ mcg
- ☐ _________ grams
- ☐ _________ I.U.
- ☐ _________ Other unit: _____________
- ☐ Don't know
- ☐ Prefer not to answer

ME02f. Do you take it regularly or only as needed?

- ☐ Regularly
- ☐ Only as needed
- ☐ Don't know
- ☐ Prefer not to answer

ME02g. For how long have you been taking it?

- ☐ For _________ days
- ☐ For _________ weeks
- ☐ For _________ months
- ☐ For _________ years
- ☐ Don't know
- ☐ Prefer not to answer

ME02h. What is the medication used for?

____

ME02i. ***Interviewer comments***:

____

ME02j. Do you take any other prescription medications or over-the-counter drugs?

- ☐ No → ***skip to end of medications questions***
- ☐ Yes
- ☐ Don't know
- ☐ Prefer not to answer

Comment [SF5]: Total dosage per day (ME.2-10.e) was dropped to reduce participant burden since this variable can be calculated.

Deleted: ME02e. Total dosage per day. ***(Record strength of how it is actually taken, not how it is prescribed.)***

Deleted: Section Break (Continuous)
<#>_________ %¶
<#>_________ mg¶
<#>_________ mcg¶
<#>_________ grams¶
<#>_________ I.U.¶
<#>_________ Other unit: _____________¶
<#>Don't know¶
<#>Prefer not to answer¶
Section Break (Continuous)
¶
Section Break (Continuous)

Deleted: *MBHT*

Deleted: *December 21, 2015*

ME03a. What is the name of the next prescription medication or over-the-counter drug that you take?

☐ Label product name: ____

☐ Label generic name: ____

☐ Don't know ☐ Prefer not to answer

ME03b. What is the dosage form?

<u>Oral</u>
☐ Pill, tablet, or capsule
☐ Sublingual or orally-disintegrating tablet
☐ Liquid solution or suspension (drink, syrup)
☐ Powder

<u>Topical</u>
☐ Liquid, cream, gel, or ointment
☐ Ear drops (otic)
☐ Eye drops (ophthalmic)
☐ Skin patch (transdermal)

<u>Inhaled</u>
☐ Inhaler or nebulizer

<u>Injected</u>
☐ Injection

<u>Suppository</u>
☐ Rectal (e.g., enema)
☐ Vaginal (e.g., douche, pessary)

<u>Other:</u>
☐ Don't know
☐ Prefer not to answer

ME03d. What is the strength? ***(Record strength of how it is actually taken, not how it is prescribed.)***

☐ _________ %
☐ _________ mg
☐ _________ mcg
☐ _________ grams
☐ _________ I.U.

☐ _________ Other unit: _____________
☐ Don't know
☐ Prefer not to answer

Deleted: ME03c. How frequently do you take it?¶
<#>Section Break (Continuous)
<#>_________ times per day ¶
<#>_________ times per week¶
<#>_________ times per month¶
<#>Don't know¶
<#>Prefer not to answer¶
Section Break (Continuous)
¶

Deleted: *MBHT*

Deleted: *December 21, 2015*

ME03e. Total dosage **per day.** ***(Record strength of how it is actually taken, not how it is prescribed.)***

- ☐ _________ %
- ☐ _________ mg
- ☐ _________ mcg
- ☐ _________ grams
- ☐ _________ I.U.
- ☐ _________ Other unit: _____________
- ☐ Don't know
- ☐ Prefer not to answer

ME3f. Do you take it regularly or only as needed?

- ☐ Regularly
- ☐ Only as needed
- ☐ Don't know
- ☐ Prefer not to answer

ME03g. For how long have you been taking it?

- ☐ For _________ days
- ☐ For _________ weeks
- ☐ For _________ months
- ☐ For _________ years
- ☐ Don't know
- ☐ Prefer not to answer

ME03h. What is the medication used for?

____

ME03i. ***Interviewer comments*:**

____

ME03j. Do you take any other prescription medications or over-the-counter drugs?

- ☐ No → ***skip to end of medications questions***
- ☐ Yes
- ☐ Don't know
- ☐ Prefer not to answer

ME04a. What is the name of the next prescription medication or over-the-counter drug that you take?

☐ Label product name:

____

☐ Label generic name:

____

☐ Don't know ☐ Prefer not to answer

Deleted: *MBHT*
Deleted: *December 21, 2015*

ME04b. What is the dosage form?

Oral
☐ Pill, tablet, or capsule
☐ Sublingual or orally-disintegrating tablet
☐ Liquid solution or suspension (drink, syrup)
☐ Powder

Topical
☐ Liquid, cream, gel, or ointment
☐ Ear drops (otic)
☐ Eye drops (ophthalmic)
☐ Skin patch (transdermal)

Inhaled
☐ Inhaler or nebulizer

Injected
☐ Injection

Suppository
☐ Rectal (e.g., enema)
☐ Vaginal (e.g., douche, pessary)

Other:
☐ Don't know
☐ Prefer not to answer

ME04c. How frequently do you take it?
☐ _________ times per day
☐ _________ times per week
☐ _________ times per month
☐ Don't know
☐ Prefer not to answer

ME04d. What is the strength? ***(Record strength of how it is actually taken, not how it is prescribed.)***
☐ _________ %
☐ _________ mg
☐ _________ mcg
☐ _________ grams
☐ _________ I.U.
☐ _________ Other unit: _____________
☐ Don't know
☐ Prefer not to answer

ME04f. Do you take it regularly or only as needed?
☐ Regularly
☐ Only as needed
☐ Don't know
☐ Prefer not to answer

Deleted: ME04e. Total dosage **per day.** ***(Record strength of how it is actually taken, not how it is prescribed.)***
Section Break (Continuous)
<#>_________ %¶
<#>_________ mg¶
<#>_________ mcg¶
<#>_________ grams¶
<#>_________ I.U.¶
<#>_________ Other unit: _____________¶
<#>Don't know¶
<#>Prefer not to answer¶
Section Break (Continuous)
¶

ME04g. For how long have you been taking it?

- ☐ For _________ days
- ☐ For _________ weeks
- ☐ For _________ months
- ☐ For _________ years
- ☐ Don't know
- ☐ Prefer not to answer

ME04h. What is the medication used for?

____

ME04i. ***Interviewer comments***:

____

ME04j. Do you take any other prescription medications or over-the-counter drugs?

- ☐ No → ***skip to end of medications section***
- ☐ Yes
- ☐ Don't know
- ☐ Prefer not to answer

ME05a. What is the name of the next prescription medication or over-the-counter drug that you take?

☐ Label product name:

____

☐ Label generic name:

____

☐ Don't know ☐ Prefer not to answer

ME05b. What is the dosage form?

Oral

- ☐ Pill, tablet, or capsule
- ☐ Sublingual or orally-disintegrating tablet
- ☐ Liquid solution or suspension (drink, syrup)
- ☐ Powder

Topical

- ☐ Liquid, cream, gel, or ointment
- ☐ Ear drops (otic)
- ☐ Eye drops (ophthalmic)
- ☐ Skin patch (transdermal)

Inhaled

- ☐ Inhaler or nebulizer

Injected

- ☐ Injection

Suppository

- ☐ Rectal (e.g., enema)
- ☐ Vaginal (e.g., douche, pessary)

Other:

- ☐ Don't know
- ☐ Prefer not to answer

Deleted: *MBHT*
Deleted: *December 21, 2015*

ME05c. How frequently do you take it?

☐ _________ times per day	☐ Don't know
☐ _________ times per week	☐ Prefer not to answer
☐ _________ times per month	

ME05d. What is the strength? ***(Record strength of how it is actually taken, not how it is prescribed.)***

☐ _________ %	☐ _________ Other unit: _____________
☐ _________ mg	☐ Don't know
☐ _________ mcg	☐ Prefer not to answer
☐ _________ grams	
☐ _________ I.U.	

ME05f. Do you take it regularly or only as needed?

☐ Regularly	☐ Don't know
☐ Only as needed	☐ Prefer not to answer

ME05g. For how long have you been taking it?

☐ For _________ days	☐ For _________ years
☐ For _________ weeks	☐ Don't know
☐ For _________ months	☐ Prefer not to answer

ME05h. What is the medication used for?

____

ME05i. ***Interviewer comments*:**

____

ME05j. Do you take any other prescription medications or over-the-counter drugs?

☐ No → ***skip to end of end of medications questions***	☐ Don't know
☐ Yes	☐ Prefer not to answer

Deleted: ¶
Section Break (Continuous)
ME05e. Total dosage **per day.** ***(Record strength of how it is actually taken, not how it is prescribed.)***
Section Break (Continuous)
<#>_________ %¶
<#>_________ mg¶
<#>_________ mcg¶
<#>_________ grams¶
<#>_________ I.U.¶
<#>_________ Other unit: _____________¶
<#>Don't know¶
<#>Prefer not to answer¶
Section Break (Continuous)

Deleted: *MBHT*
Deleted: *December 21, 2015*

ME06a. What is the name of the next prescription medication or over-the-counter drug that you take?

☐ Label product name: ____

☐ Label generic name: ____

☐ Don't know
☐ Prefer not to answer

ME06b. What is the dosage form?

Oral
☐ Pill, tablet, or capsule
☐ Sublingual or orally-disintegrating tablet
☐ Liquid solution or suspension (drink, syrup)
☐ Powder

Topical
☐ Liquid, cream, gel, or ointment
☐ Ear drops (otic)
☐ Eye drops (ophthalmic)
☐ Skin patch (transdermal)

Inhaled
☐ Inhaler or nebulizer

Injected
☐ Injection

Suppository
☐ Rectal (e.g., enema)
☐ Vaginal (e.g., douche, pessary)

Other:
☐ Don't know
☐ Prefer not to answer

ME06c. How frequently do you take it?
☐ _________ times per day
☐ _________ times per week
☐ _________ times per month
☐ Don't know
☐ Prefer not to answer

ME06d. What is the strength? ***(Record strength of how it is actually taken, not how it is prescribed.)***
☐ _________ %
☐ _________ mg
☐ _________ mcg
☐ _________ grams
☐ _________ I.U.
☐ _________ Other unit: _____________
☐ Don't know
☐ Prefer not to answer

Deleted: MBHT
Deleted: December 21, 2015

ME06f. Do you take it regularly or only as needed?

- ☐ Regularly
- ☐ Only as needed
- ☐ Don't know
- ☐ Prefer not to answer

ME06g. For how long have you been taking it?

- ☐ For _________ days
- ☐ For _________ weeks
- ☐ For _________ months
- ☐ For _________ years
- ☐ Don't know
- ☐ Prefer not to answer

ME06h. What is the medication used for?

____

ME06i. ***Interviewer comments*:**

____

ME06i. Do you take any other prescription medications or over-the-counter drugs?

- ☐ No → ***skip to end of medications questions***
- ☐ Yes
- ☐ Don't know
- ☐ Prefer not to answer

ME07a. What is the name of the next prescription medication or over-the-counter drug that you take?

☐ Label product name:

____

☐ Label generic name:

____

- ☐ Don't know
- ☐ Prefer not to answer

Deleted: ME06e. Total dosage **per day.** ***(Record strength of how it is actually taken, not how it is prescribed.)***

Section Break (Continuous)

<#>_________ %¶
<#>_________ mg¶
<#>_________ mcg¶
<#>_________ grams¶
<#>_________ I.U.¶
<#>_________ Other unit: _____________¶
<#>Don't know¶
<#>Prefer not to answer¶

Section Break (Continuous)

Deleted: *MBHT*

Deleted: *December 21, 2015*

ME07b. What is the dosage form?

Oral
☐ Pill, tablet, or capsule
☐ Sublingual or orally-disintegrating tablet
☐ Liquid solution or suspension (drink, syrup)
☐ Powder

Topical
☐ Liquid, cream, gel, or ointment
☐ Ear drops (otic)
☐ Eye drops (ophthalmic)
☐ Skin patch (transdermal)

Inhaled
☐ Inhaler or nebulizer

Injected
☐ Injection

Suppository
☐ Rectal (e.g., enema)
☐ Vaginal (e.g., douche, pessary)

Other:
☐ Don't know
☐ Prefer not to answer

ME07c. How frequently do you take it?
☐ _________ times per day
☐ _________ times per week
☐ _________ times per month
☐ Don't know
☐ Prefer not to answer

ME07d. What is the strength? ***(Record strength of how it is actually taken, not how it is prescribed.)***
☐ _________ %
☐ _________ mg
☐ _________ mcg
☐ _________ grams
☐ _________ I.U.
☐ _________ Other unit: _____________
☐ Don't know
☐ Prefer not to answer

Deleted: ME07e. Total dosage **per day.** ***(Record strength of how it is actually taken, not how it is prescribed.)***
Section Break (Continuous)
<#>_________ %¶
<#>_________ mg¶
<#>_________ mcg¶
<#>_________ grams¶
<#>_________ I.U.¶
<#>_________ Other unit: _____________¶
<#>Don't know¶
<#>Prefer not to answer¶
Section Break (Continuous)

ME07f. Do you take it regularly or only as needed?
☐ Regularly
☐ Only as needed
☐ Don't know
☐ Prefer not to answer

Deleted: *MBHT*
Deleted: *December 21, 2015*

ME07g For how long have you been taking it?

- ☐ For _________ days
- ☐ For _________ weeks
- ☐ For _________ months
- ☐ For _________ years
- ☐ Don't know
- ☐ Prefer not to answer

ME07h. What is the medication used for?

____

ME07i. ***Interviewer comments***:
____

ME07j. Do you take any other prescription medications or over-the-counter drugs?

- ☐ No → ***skip to end of medications questions***
- ☐ Yes
- ☐ Don't know
- ☐ Prefer not to answer

ME08a. What is the name of the next prescription medication or over-the-counter drug that you take?

- ☐ Label product name:
____
- ☐ Label generic name:
____
- ☐ Don't know
- ☐ Prefer not to answer

ME08b. What is the dosage form?

Oral
- ☐ Pill, tablet, or capsule
- ☐ Sublingual or orally-disintegrating tablet
- ☐ Liquid solution or suspension (drink, syrup)
- ☐ Powder

Topical
- ☐ Liquid, cream, gel, or ointment
- ☐ Ear drops (otic)
- ☐ Eye drops (ophthalmic)
- ☐ Skin patch (transdermal)

Inhaled
- ☐ Inhaler or nebulizer

Injected
- ☐ Injection

Suppository
- ☐ Rectal (e.g., enema)
- ☐ Vaginal (e.g., douche, pessary)

Other:
- ☐ Don't know
- ☐ Prefer not to answer

ME08c. How frequently do you take it?

- ☐ _________ times per day
- ☐ _________ times per week
- ☐ _________ times per month
- ☐ Don't know
- ☐ Prefer not to answer

ME08d. What is the strength? *(Record strength of how it is actually taken, not how it is prescribed.)*

- ☐ _________ %
- ☐ _________ mg
- ☐ _________ mcg
- ☐ _________ grams
- ☐ _________ I.U.
- ☐ _________ Other unit: _____________
- ☐ Don't know
- ☐ Prefer not to answer

Deleted: ¶
ME08e. Total dosage **per day.** ***(Record strength of how it is actually taken, not how it is prescribed.)***
Section Break (Continuous)
<#>_________ %¶
<#>_________ mg¶
<#>_________ mcg¶
<#>_________ grams¶
<#>_________ I.U.¶
<#>_________ Other unit: _____________¶
<#>Don't know¶
<#>Prefer not to answer¶
Section Break (Continuous)

ME08f. Do you take it regularly or only as needed?

- ☐ Regularly
- ☐ Only as needed
- ☐ Don't know
- ☐ Prefer not to answer

ME08g. For how long have you been taking it?

- ☐ For _________ days
- ☐ For _________ weeks
- ☐ For _________ months
- ☐ For _________ years
- ☐ Don't know
- ☐ Prefer not to answer

ME08h. What is the medication used for?

____

ME08i. ***Interviewer comments***:

____

ME08j. Do you take any other prescription medications or over-the-counter drugs?

- ☐ No → ***skip to end of medications questions***
- ☐ Yes
- ☐ Don't know
- ☐ Prefer not to answer

ME09a. What is the name of the next prescription medication or over-the-counter drug that you take?

☐ Label product name: ____

☐ Label generic name: ____

☐ Don't know ☐ Prefer not to answer

ME09b. What is the dosage form?

Oral
☐ Pill, tablet, or capsule
☐ Sublingual or orally-disintegrating tablet
☐ Liquid solution or suspension (drink, syrup)
☐ Powder

Topical
☐ Liquid, cream, gel, or ointment
☐ Ear drops (otic)
☐ Eye drops (ophthalmic)
☐ Skin patch (transdermal)

Inhaled
☐ Inhaler or nebulizer

Injected
☐ Injection

Suppository
☐ Rectal (e.g., enema)
☐ Vaginal (e.g., douche, pessary)

Other:
☐ Don't know
☐ Prefer not to answer

ME09c. How frequently do you take it?

☐ _________ times per day
☐ _________ times per week
☐ _________ times per month

☐ Don't know
☐ Prefer not to answer

ME09d. What is the strength? *(Record strength of how it is actually taken, not how it is prescribed.)*

☐ _________ %
☐ _________ mg
☐ _________ mcg
☐ _________ grams
☐ _________ I.U.

☐ _________ Other unit: _____________
☐ Don't know
☐ Prefer not to answer

Deleted: *MBHT*
Deleted: *December 21, 2015*

ME09f. Do you take it regularly or only as needed?

☐ Regularly
☐ Only as needed
☐ Don't know
☐ Prefer not to answer

ME09g. For how long have you been taking it?

☐ For _________ days
☐ For _________ weeks
☐ For _________ months
☐ For _________ years
☐ Don't know
☐ Prefer not to answer

ME09h. What is the medication used for?

____

ME09i. ***Interviewer comments*:**

____

ME09j. Do you take any other prescription medications or over-the-counter drugs?

☐ No → ***skip to end of medications questions***
☐ Yes
☐ Don't know
☐ Prefer not to answer

ME10a. What is the name of the next prescription medication or over-the-counter drug that you take?

☐ Label product name: ____

☐ Label generic name: ____

☐ Don't know ☐ Prefer not to answer

Deleted: ME09e. Total dosage **per day.** ***(Record strength of how it is actually taken, not how it is prescribed.)***
Section Break (Continuous)
<#>_________ %¶
<#>_________ mg¶
<#>_________ mcg¶
<#>_________ grams¶
<#>_________ I.U.¶
<#>_________ Other unit: _____________¶
<#>Don't know¶
<#>Prefer not to answer¶
Section Break (Continuous)

Deleted: *MBHT*

Deleted: *December 21, 2015*

ME10b. What is the dosage form?

Oral
- ☐ Pill, tablet, or capsule
- ☐ Sublingual or orally-disintegrating tablet
- ☐ Liquid solution or suspension (drink, syrup)
- ☐ Powder

Topical
- ☐ Liquid, cream, gel, or ointment
- ☐ Ear drops (otic)
- ☐ Eye drops (ophthalmic)
- ☐ Skin patch (transdermal)

Inhaled
- ☐ Inhaler or nebulizer

Injected
- ☐ Injection

Suppository
- ☐ Rectal (e.g., enema)
- ☐ Vaginal (e.g., douche, pessary)

Other:
- ☐ Don't know
- ☐ Prefer not to answer

ME10c. How frequently do you take it?
- ☐ _________ times per day
- ☐ _________ times per week
- ☐ _________ times per month
- ☐ Don't know
- ☐ Prefer not to answer

ME10d. What is the strength? ***(Record strength of how it is actually taken, not how it is prescribed.)***
- ☐ _________ %
- ☐ _________ mg
- ☐ _________ mcg
- ☐ _________ grams
- ☐ _________ I.U.
- ☐ _________ Other unit: _____________
- ☐ Don't know
- ☐ Prefer not to answer

Deleted: ME10e. Total dosage **per day.** ***(Record strength of how it is actually taken, not how it is prescribed.)***
Section Break (Continuous)
<#>_________ %¶
<#>_________ mg¶
<#>_________ mcg¶
<#>_________ grams¶
<#>_________ I.U.¶
<#>_________ Other unit: _____________¶
<#>Don't know¶
<#>Prefer not to answer¶
Section Break (Continuous)

ME10f. Do you take it regularly or only as needed?
- ☐ Regularly
- ☐ Only as needed
- ☐ Don't know
- ☐ Prefer not to answer

Deleted: *MBHT*

Deleted: *December 21, 2015*

ME10g. For how long have you been taking it?

- ☐ For _________ days
- ☐ For _________ weeks
- ☐ For _________ months
- ☐ For _________ years
- ☐ Don't know
- ☐ Prefer not to answer

ME10h. What is the medication used for?

____

ME10i. ***Interviewer comments***:
____

Deleted: *MBHT*

Deleted: *December 21, 2015*

FOOD FREQUENCY QUESTIONNAIRE:

RA Script to be read to participants: At this time, we are going to have you complete a few forms on your own. The first is a Food Frequency Questionnaire that will ask you about the types of foods and drinks that you consume. It should take around 20 minutes to complete. Please let me know if you have any questions.

Comment [SF6]: To reduce participant burden we have dropped several of the variables collected on the Food Frequency Questionnaire. See attached document with mark up for FFQ variables that were dropped.

Deleted: *MBHT*

Deleted: *December 21, 2015*

Dietary Assessment

ID: □□□□□□ – □□

1. Do you currently take multi-vitamins? (Please report other individual vitamins in question 2.)

○ No ○ Yes → **If YES, a) How many do you take per week?**
○ 2 or less ○ 3-5 ○ 6-9 ○ 10 or more

b) For how many years have you taken them?
○ 0-1 ○ 2-4 ○ 5-9 ○ 10 or more

c) What specific brand do you usually use? → Specify exact brand and type

2. Not counting multi-vitamins, do you take any of the following preparations:

a) Vitamin A? ○ No ○ Yes, seasonal only ○ Yes, most months	If YES,	How many years?	○ 0-1 yr.	○ 2-4 years	○ 5-9 years	○ 10+ years	○ Don't know
		What dose per day?	○ Less than 8,000 IU	○ 8,000 to 12,000 IU	○ 13,000 to 22,000 IU	○ 23,000 IU or more	○ Don't know
b) Vitamin C? ○ No ○ Yes, seasonal only ○ Yes, most months	If YES,	How many years?	○ 0-1 yr.	○ 2-4 years	○ 5-9 years	○ 10+ years	○ Don't know
		What dose per day?	○ Less than 400 mg.	○ 400 to 700 mg.	○ 750 to 1250 mg.	○ 1300 mg. or more	○ Don't know
c) Vitamin B_6? ○ No ○ Yes	If YES,	How many years?	○ 0-1 yr.	○ 2-4 years	○ 5-9 years	○ 10+ years	○ Don't know
		What dose per day?	○ Less than 10 mg.	○ 10 to 39 mg.	○ 40 to 79 mg.	○ 80 mg. or more	○ Don't know
d) Vitamin E? ○ No ○ Yes	If YES,	How many years?	○ 0-1 yr.	○ 2-4 years	○ 5-9 years	○ 10+ years	○ Don't know
		What dose per day?	○ Less than 100 IU	○ 100 to 250 IU	○ 300 to 500 IU	○ 600 IU or more	○ Don't know
e) Selenium? ○ No ○ Yes	If YES,	How many years?	○ 0-1 yr.	○ 2-4 years	○ 5-9 years	○ 10+ years	○ Don't know
		What dose per day?	○ Less than 80 mcg.	○ 80 to 130 mcg.	○ 140 to 250 mcg.	○ 260 mcg. or more	○ Don't know
f) Iron? ○ No ○ Yes	If YES,	How many years?	○ 0-1 yr.	○ 2-4 years	○ 5-9 years	○ 10+ years	○ Don't know
		What dose per day?	○ Less than 51 mg.	○ 51 to 200 mg.	○ 201 to 400 mg.	○ 401 mg. or more	○ Don't know
g) Zinc? ○ No ○ Yes	If YES,	How many years?	○ 0-1 yr.	○ 2-4 years	○ 5-9 years	○ 10+ years	○ Don't know
		What dose per day?	○ Less than 25 mg.	○ 25 to 74 mg.	○ 75 to 100 mg.	○ 101 mg. or more	○ Don't know
h) Calcium? (Include Calcium in Dolomite and Tums, etc.) ○ No ○ Yes	If YES,	How many years?	○ 0-1 yr.	○ 2-4 years	○ 5-9 years	○ 10+ years	○ Don't know
		What dose per day?	○ Less than 400 mg.	○ 400 to 900 mg.	○ 901 to 1300 mg.	○ 1301 mg. or more	○ Don't know

i) Are there other supplements that you take on a regular basis? Please mark if yes:

○ Metamucil	○ Cod liver oil	○ Iodine	○ Beta-carotene	○ Other (please specify)
○ Vitamin D	○ Folic acid	○ Copper	○ Niacin	
○ B-Complex Vitamins	○ Omega-3 fatty acids	○ Brewer's yeast	○ Magnesium	

3. For each food listed, fill in the circle indicating how often on average you have used the amount specified during the past year.

DAIRY FOODS	AVERAGE USE LAST YEAR: NEVER OR LESS THAN ONCE PER MONTH	1-3 PER MONTH	1 PER WEEK	2-4 PER WEEK	5-6 PER WEEK	1 PER DAY	2-3 PER DAY	4-5 PER DAY	6+ PER DAY
Skim or low fat milk (8 oz. glass)	○	○	Ⓦ	○	○	Ⓓ	○	○	○
Whole milk (8 oz. glass)	○	○	Ⓦ	○	○	Ⓓ	○	○	○
Yogurt (1 cup)	○	○	Ⓦ	○	○	Ⓓ	○	○	○
Ice Cream (1/2 cup)	○	○	Ⓦ	○	○	Ⓓ	○	○	○
Cottage or ricotta cheese (1/2 cup)	○	○	Ⓦ	○	○	Ⓓ	○	○	○
Other cheese, e.g., American, cheddar, etc., plain or as part of a dish (1 slice or 1 oz. serving)	○	○	Ⓦ	○	○	Ⓓ	○	○	○
Margarine (pat), added to food or bread; exclude use in cooking	○	○	Ⓦ	○	○	Ⓓ	○	○	○
Butter (pat), added to food or bread; exclude use in cooking	○	○	Ⓦ	○	○	Ⓓ	○	○	○

4. What form of margarine do you usually use? → What specific brand and type (e.g., Parkay Corn Oil Spread)

○ None ○ Stick ○ Tub ○ Extra light
○ 'Lite' stick ○ 'Lite' tub ○ Squeeze

PLEASE TURN TO PAGE 2

3. (Continued) Please fill in your __average__ use, __during the past year__, of each specified food.

Please try to average your seasonal use of foods over the entire year. For example, if a food such as peaches are eaten 4 times a week during the approximate 3 months that it is in season, then the __average__ use would be once per week.

FRUITS	NEVER OR LESS THAN ONCE PER MONTH	1-3 PER MONTH	1 PER WEEK	2-4 PER WEEK	5-6 PER WEEK	1 PER DAY	2-3 PER DAY	4-5 PER DAY	6+ PER DAY
Fresh apples or pears (1)	○	○	Ⓦ	○	○	Ⓓ	○	○	○
Oranges (1)	○	○	Ⓦ	○	○	Ⓓ	○	○	○
Orange juice or grapefruit juice (small glass)	○	○	Ⓦ	○	○	Ⓓ	○	○	○
Peaches, apricots or plums (1 fresh, or 1/2 cup canned)	○	○	Ⓦ	○	○	Ⓓ	○	○	○
Bananas (1)	○	○	Ⓦ	○	○	Ⓓ	○	○	○
Other fruits, fresh, frozen, or canned (1/2 cup)	○	○	Ⓦ	○	○	Ⓓ	○	○	○

VEGETABLES	NEVER OR LESS THAN ONCE PER MONTH	1-3 PER MONTH	1 PER WEEK	2-4 PER WEEK	5-6 PER WEEK	1 PER DAY	2-3 PER DAY	4-5 PER DAY	6+ PER DAY
Tomatoes (1) or Tomato juice (small glass)	○	○	Ⓦ	○	○	Ⓓ	○	○	○
String beans (1/2 cup)	○	○	Ⓦ	○	○	Ⓓ	○	○	○
Broccoli (1/2 cup)	○	○	Ⓦ	○	○	Ⓓ	○	○	○
Cabbage, cauliflower, or Brussels sprouts (1/2 cup)	○	○	Ⓦ	○	○	Ⓓ	○	○	○
Carrots, raw (1/2 carrot or 2-4 sticks)	○	○	Ⓦ	○	○	Ⓓ	○	○	○
Carrots, cooked (1/2 cup)	○	○	Ⓦ	○	○	Ⓓ	○	○	○
Corn (1 ear or 1/2 cup frozen or canned)	○	○	Ⓦ	○	○	Ⓓ	○	○	○
Peas or lima beans (1/2 cup fresh, frozen, canned)	○	○	Ⓦ	○	○	Ⓓ	○	○	○
Yams or sweet potatoes (1/2 cup)	○	○	Ⓦ	○	○	Ⓓ	○	○	○
Spinach or collard greens, cooked (1/2 cup)	○	○	Ⓦ	○	○	Ⓓ	○	○	○
Beans or lentils, baked or dried (1/2 cup)	○	○	Ⓦ	○	○	Ⓓ	○	○	○
Yellow (winter) squash (1/2 cup)	○	○	Ⓦ	○	○	Ⓓ	○	○	○

MEAT, SWEETS, BAKED GOODS, CEREAL, MISC.	NEVER OR LESS THAN ONCE PER MONTH	1-3 PER MONTH	1 PER WEEK	2-4 PER WEEK	5-6 PER WEEK	1 PER DAY	2-3 PER DAY	4-5 PER DAY	6+ PER DAY
Eggs (1)	○	○	Ⓦ	○	○	Ⓓ	○	○	○
Chicken or turkey, with skin (4-6 oz.)	○	○	Ⓦ	○	○	Ⓓ	○	○	○
Chicken or turkey, without skin (4-6 oz.)	○	○	Ⓦ	○	○	Ⓓ	○	○	○
Bacon (2 slices)	○	○	Ⓦ	○	○	Ⓓ	○	○	○
Hot dogs (1)	○	○	Ⓦ	○	○	Ⓓ	○	○	○
Processed meats, e.g., sausage, salami, bologna, etc. (piece or slice)	○	○	Ⓦ	○	○	Ⓓ	○	○	○
Liver (3-4 oz.)	○	○	Ⓦ	○	○	Ⓓ	○	○	○
Hamburger (1 patty)	○	○	Ⓦ	○	○	Ⓓ	○	○	○
Beef, pork, or lamb as a sandwich or mixed dish, e.g., stew, casserole, lasagna, etc.	○	○	Ⓦ	○	○	Ⓓ	○	○	○
Beef, pork, or lamb as a main dish, e.g. steak, roast, ham, etc. (4-6 oz.)	○	○	Ⓦ	○	○	Ⓓ	○	○	○
Fish (3-5 oz.)	○	○	Ⓦ	○	○	Ⓓ	○	○	○
Chocolate (1 oz.)	○	○	Ⓦ	○	○	Ⓓ	○	○	○
Candy without chocolate (1 oz.)	○	○	Ⓦ	○	○	Ⓓ	○	○	○
Pie, homemade (slice)	○	○	Ⓦ	○	○	Ⓓ	○	○	○
Pie, ready made (slice)	○	○	Ⓦ	○	○	Ⓓ	○	○	○
Cake (slice)	○	○	Ⓦ	○	○	Ⓓ	○	○	○
Cookies (1)	○	○	Ⓦ	○	○	Ⓓ	○	○	○
Cold breakfast cereal (1 cup)	○	○	Ⓦ	○	○	Ⓓ	○	○	○
White bread (slice), including pita bread	○	○	Ⓦ	○	○	Ⓓ	○	○	○
Dark bread (slice), including wheat pita bread	○	○	Ⓦ	○	○	Ⓓ	○	○	○
French fried potatoes (4 oz.)	○	○	Ⓦ	○	○	Ⓓ	○	○	○
Potatoes, baked, boiled (1) or mashed (1 cup)	○	○	Ⓦ	○	○	Ⓓ	○	○	○
Rice or Pasta, e.g., spaghetti, noodles, etc. (1 cup)	○	○	Ⓦ	○	○	Ⓓ	○	○	○
Potato chips or corn chips (small bag or 1 oz.)	○	○	Ⓦ	○	○	Ⓓ	○	○	○
Nuts (small packet or 1 oz.)	○	○	Ⓦ	○	○	Ⓓ	○	○	○
Peanut butter (1 Tbs)	○	○	Ⓦ	○	○	Ⓓ	○	○	○
Oil and vinegar dressing, e.g., Italian, (1 Tbs)	○	○	Ⓦ	○	○	Ⓓ	○	○	○

PLEASE GO TO PAGE 3

4. (Continued) Please fill in your <u>average</u> use, <u>during the past year</u>, of each specified food.

BEVERAGES	NEVER OR LESS THAN ONCE PER MONTH	1-3 PER MONTH	1 PER WEEK	2-4 PER WEEK	5-6 PER WEEK	1 PER DAY	2-3 PER DAY	4-5 PER DAY	6+ PER DAY
Coffee, not decaffeinated (1 cup)	○	○	Ⓦ	○	○	Ⓓ	○	○	○
Tea (1 cup), not herbal tea	○	○	Ⓦ	○	○	Ⓓ	○	○	○
Beer (1 glass, bottle, can)	○	○	Ⓦ	○	○	Ⓓ	○	○	○
Wine (4 oz. glass)	○	○	Ⓦ	○	○	Ⓓ	○	○	○
Liquor, e.g., whiskey, gin, etc. (1 drink or shot)	○	○	Ⓦ	○	○	Ⓓ	○	○	○
Low calorie carbonated beverage, e.g., Diet Coke	○	○	Ⓦ	○	○	Ⓓ	○	○	○
Carbonated beverage with sugar, e.g., Coke, Pepsi	○	○	Ⓦ	○	○	Ⓓ	○	○	○
Hawaiian Punch, lemonade, or other fruit drinks	○	○	Ⓦ	○	○	Ⓓ	○	○	○

Consider the serving size as 1 glass, bottle or can for these fruit and carbonated beverages.

5. How many teaspoons of sugar do you add to your beverages or food each day? tsp.

6. Which <u>cold</u> breakfast cereal do you usually eat? Specify exact brand and type

○ Don't eat cold breakfast cereal

7. How much of the visible fat on your beef, pork or lamb do you remove before eating?

○ Remove all visible fat ○ Remove most ○ Remove small part of fat ○ Remove none ○ Don't eat meat

8. What kind of fat do you usually use for frying and sautéing at home? (Exclude "Pam"-type spray)

○ Real butter ○ Margarine ○ Vegetable oil ○ Vegetable shortening ○ Lard

9. What kind of fat do you usually use for baking at home?

○ Real butter ○ Margarine ○ Vegetable oil ○ Vegetable shortening ○ Lard

10. How often do you eat food that is fried at home? (Exclude "Pam"-type spray)

○ Less than once a week ○ 1-3 times per week ○ 4-6 times per week ○ Daily

11. How often do you eat fried food away from home? (e.g., french fries, fried chicken, fried fish)

○ Less than once a week ○ 1-3 times per week ○ 4-6 times per week ○ Daily

12. What type of cooking oil do you usually use at home (e.g., Mazola Corn Oil)? Specify brand and type

13. Do you use a microwave oven?

○ Yes → **If yes, for how many years?** Number of years using a microwave

○ No

14. Do you currently follow a physician-prescribed special diet?

○ Yes → **a) If yes, for how many years?** Number of years on diet

○ No

b) If yes, what kind of diet do you follow? (Select more than one if necessary.)

○ Weight reduction (low calorie) ○ Low cholesterol ○ Low sodium

○ Diabetic ○ Low fat ○ Low triglyceride ○ Ulcer ○ High potassium

○ Other → Specify type of diet

15. How has your use of the following foods and beverages changed over the PAST FIVE YEARS?

FOOD	USE HAS DECREASED	USE ABOUT THE SAME	USE HAS INCREASED
a) Whole milk	○	○	○
b) Butter	○	○	○
c) Margarine	○	○	○
d) Eggs	○	○	○
e) Fish	○	○	○
f) Red meat	○	○	○
g) Fruits	○	○	○
h) Vegetables	○	○	○
i) Whole wheat bread	○	○	○
j) Whole grains	○	○	○
k) Sugar	○	○	○
l) Alcohol	○	○	○

Copyright © 1991, Brigham and Women's Hospital

Questions for Participants to Answer on Their Own In-Person on Paper:
For each statement, please place a mark in the column that best describes how you have been feeling.

	Not at all or less than 1 day last week	1 or 2 days last week	3 to 4 days last week	5 to 7 days last week	Nearly every day for two weeks	I do not know	I prefer not to answer
DS_1. My appetite was poor.	☐	☐	☐	☐	☐	☐	☐
DS_2. I could not shake off the blues.	☐	☐	☐	☐	☐	☐	☐
DS_3. I had trouble keeping my mind on what I was doing.	☐	☐	☐	☐	☐	☐	☐
DS_4. I felt depressed.	☐	☐	☐	☐	☐	☐	☐
DS_5. My sleep was restless.	☐	☐	☐	☐	☐	☐	☐
DS_6. I felt sad.	☐	☐	☐	☐	☐	☐	☐
DS_7. I could not get going.	☐	☐	☐	☐	☐	☐	☐
DS_8. Nothing made me happy.	☐	☐	☐	☐	☐	☐	☐
C_9. I felt like a bad person.	☐	☐	☐	☐	☐	☐	☐
DS_10. I lost interest in my usual activities.	☐	☐	☐	☐	☐	☐	☐
DS_11. I slept much more than usual.	☐	☐	☐	☐	☐	☐	☐
DS_12. I felt like I was moving too slowly.	☐	☐	☐	☐	☐	☐	☐
DS_13. I felt fidgety.	☐	☐	☐	☐	☐	☐	☐
DS_14. I wished I were dead.	☐	☐	☐	☐	☐	☐	☐
DS_15. I wanted to hurt myself.	☐	☐	☐	☐	☐	☐	☐
DS_16. I was tired all the time.	☐	☐	☐	☐	☐	☐	☐
DS_17. I did not like myself.	☐	☐	☐	☐	☐	☐	☐
DS_18. I lost a lot of weight without trying to.	☐	☐	☐	☐	☐	☐	☐
DS_19. I had a lot of trouble getting to sleep.	☐	☐	☐	☐	☐	☐	☐
DS_20. I could not focus on the important things.	☐	☐	☐	☐	☐	☐	☐

Deleted: *MBHT*
Deleted: *December 21, 2015*

Below is a list of common symptoms of anxiety. Please carefully read each item in the list. Indicate how much you have been bothered by that symptom during the past month, including today, by marking the box in the corresponding space in the column next to each symptom.

	Not At All	Mildly – it didn't bother me much	Moderately – it wasn't pleasant at all times	Severely – it bothered me a lot	I do not know	I prefer not to answer
BE_1. Numbness or tingling	☐	☐	☐	☐	☐	☐
BE_2. Feeling hot	☐	☐	☐	☐	☐	☐
BE _3. Wobbliness in legs	☐	☐	☐	☐	☐	☐
BE _4. Unable to relax	☐	☐	☐	☐	☐	☐
BE _5. Fear of worst happening	☐	☐	☐	☐	☐	☐
BE _6. Dizzy or lightheaded	☐	☐	☐	☐	☐	☐
BE _7. Heart pounding/racing	☐	☐	☐	☐	☐	☐
BE _8. Unsteady	☐	☐	☐	☐	☐	☐
BE _9. Terrified or afraid	☐	☐	☐	☐	☐	☐
BE _10. Nervous	☐	☐	☐	☐	☐	☐
BE _11. Feeling of choking	☐	☐	☐	☐	☐	☐
BE _12. Hands trembling	☐	☐	☐	☐	☐	☐
BE _13. Shaky/unsteady	☐	☐	☐	☐	☐	☐
BE _14. Fear of losing control	☐	☐	☐	☐	☐	☐
BE _15. Difficulty in breathing	☐	☐	☐	☐	☐	☐
BE _16. Fear of dying	☐	☐	☐	☐	☐	☐
BE _17. Scared	☐	☐	☐	☐	☐	☐
BE _18. Indigestion	☐	☐	☐	☐	☐	☐
BE _19. Faint/lightheaded	☐	☐	☐	☐	☐	☐
BE _20. Face flushed	☐	☐	☐	☐	☐	☐
BE _21. Hot/cold sweats	☐	☐	☐	☐	☐	☐

Deleted: *MBHT*
Deleted: *December 21, 2015*

After questionnaires are complete, participants will be asked to perform the following two computer-based tests:

Sustained Attention to Response Task (15 minutes)

The Sustained Attention to Response Task (SART) is a computerized test of sustained attention, response inhibition (executive function) and self-regulation. Subjects are instructed to press a key in response to rapidly displayed integers (1-9) and withhold response to a designated "no-go" integer. SART errors consist of summed commission errors (button press on no-go trial) and omission errors (button not pressed on "go" integers). SART performance is associated with prefrontal cortex functioning, has been found to increase with mindfulness training and is correlated with scores on mindfulness questionnaires (specifically, the Mindful Attention Awareness Scale).

Attention Control: Attention Network Test (20 minutes)

Attention Network Test (ANT) is a brief computerized battery measuring three independent behavioral components of attention: Conflict resolution (ability to overcome distracting stimuli), spatial Orienting (the benefit of valid spatial pre-cues), and Alerting (the benefit of temporal pre-cues). Efficiency of orienting is examined by changes in RT that accompany cues indicating where the target will occur. The efficiency of the executive conflict resolution network is examined by requiring the subject to respond by pressing two keys indicating the direction (left or right) of a central arrow surrounded by congruent, incongruent or neutral flankers. Moderate to high reliabilities are found for all networks.

ANT data to be entered by RA *prior to* ANT:

PPT Age: ______

PPT Sex: Male / Female

Category: Normal

Diagonal of "subject info" window: 10"

Press "distance between eyes and screen" button to calculate. Should be 23.2.

ANT data to be entered by RA *after* ANT:

PPT Age: ______

Alerting effect (ms): ______

Orienting effect (ms): ______

Conflict effect (ms): ______

Mean RT for correct trials (ms): ______

Mean accuracy (%):______

Comment [SF7]: We switched the order of administration for the ANT and SART. The SART is a better measure of attention, so it is important that it is completed first, in case the first measure has an effect on the outcome of the second measure completed.

Moved (insertion) [1]

Moved up [1]: Sustained Attention to Response Task (15 minutes)¶
¶
The Sustained Attention to Response Task (SART) is a computerized test of sustained attention, response inhibition (executive function) and self-regulation. Subjects are instructed to press a key in response to rapidly displayed integers (1-9) and withhold response to a designated "no-go" integer. SART errors consist of summed commission errors (button press on no-go trial) and omission errors (button not pressed on "go" integers). SART performance is associated with prefrontal cortex functioning, has been found to increase with mindfulness training and is correlated with scores on mindfulness questionnaires (specifically, the Mindful Attention Awareness Scale). ¶
¶

Deleted: MBHT

Deleted: December 21, 2015

SAFETY PLAN

After the participant has completed the Beck Anxiety Inventory questionnaire and the Centers for Epidemiologic Studies Depressive Symptomatology questionnaire, ask the participant to wait for a few minutes while you check that all forms and assessments are completed. While the participant is waiting in a different room, check the scores according to the criteria below. If any scores trigger the safety plan, move forward with steps below as written.

Beck Anxiety Inventory (BA)

If participant scores ≥26 on the Beck Anxiety Inventory, while having the participant wait, immediately notify the collaborating psychiatrist, Dr. Ellen Flynn (cell phone # 401-258-9829), to determine need for a psychiatric consultation. She has provided her cell phone #, and your phone # will be entered into her phone recognition software to indicate it is a call from the MBHT study. It is likely she will answer the phone. If Dr. Flynn cannot be reached, please leave a phone message for her with your contact information and provide her with the Beck Anxiety Inventory results on the message. If Dr. Flynn is not immediately reachable, or following participant release after consulting with Dr. Flynn, immediately contact the PI, Dr. Eric Loucks (cell phone #401-369-0443).

Depressive Symptomatology (DS)

The CESD-R will be administered during the in-person assessment visits, and scores will be reviewed immediately upon completion of the in-person assessments.

1. Sadness (dysphoria): Question numbers 2,4, 6
2. Loss of Interest (anhedonia): Question numbers 8, 10
3. Appetite: Question numbers 1, 18
4. Sleep: Question numbers 5, 11, 19
5. Thinking / concentration: Question numbers 3, 20
6. Guilt (worthlessness): Question numbers 9, 17
7. Tired (fatigue): Question numbers 7, 16
8. Movement (agitation): Question numbers 12, 13
9. Suicidal ideation: Question numbers 14, 15

Participants are considered to meet criteria for major depressive episode if they have anhedonia or dysphoria nearly every day for the past two weeks, plus symptoms in an additional 4 DSM symptom groups noted as occurring nearly every day for the past two weeks. If participants meet criteria for major depressive episode, while having the participant wait, immediately notify the collaborating psychiatrist, Dr. Ellen Flynn (cell phone # 401-258-9829), to determine need for a psychiatric consultation. She has provided her cell phone #, and your phone # will be entered into her phone recognition software to indicate it is a call from the MBHT study. It is likely she will answer the phone. If Dr. Flynn cannot be reached, please leave a phone message for her with your contact information and provide her with the Depressive Symptomatology results on the message. If Dr. Flynn is not immediately reachable, or following participant release after consulting with Dr. Flynn, immediately contact the PI, Dr. Eric Loucks (cell phone #401-369-0443).

Deleted: *MBHT*

Deleted: *December 21, 2015*

If participants respond having any suicidal ideation (DS questions 14 or 15), perform the following 2 steps:

1. **Immediately call 911 and Dr. Ellen Flynn.**

Specifically, while the participant is in the waiting room, ***call 911 immediately, and tell them:***

"My name is _____. I am working on a research study at Brown University. I have a study participant in the waiting room who has shared that he/she is currently suicidal." Please provide the participants' name to the 911 operator, as requested.

Finally, after calling 911, please call Dr. Ellen Flynn, who is the psychiatrist supporting this study. Her cell phone # is 401-258-9829. Please provide her with the same information as was done during the 911 call.

2. **While speaking calmly with the participant, let them know that you have called 911 and Dr. Flynn, and why (i.e. because we are concerned about you). You can speak with participant to keep him/her in the waiting room if 911 has sent assistance, but the discussion should not be clinical in nature.**

Examples of questions that could be asked in order to keep them in the waiting room:
- "Tell me what is going on."
- "What's happening right now?"
- Tell me more about why you are interesting in being part of this study.
- What are you hoping to get out of this study?

The following information can be provided to study participants.

National Suicide Prevention Lifeline: 1-800-273-8255

Other options are to:
- Call your doctor's office
- Call 911 for emergency services
- Go to the nearest hospital emergency room.

Local Non-Urgent Free or Inexpensive Mental Health Services:
Gateway Healthcare: 401-729-8701
Anchor Counseling Center: 401-475-9979
The Providence Center: 401-276-4020

Deleted: *MBHT*
Deleted: *December 21, 2015*

Appendix 12:
Revised Home Baseline Assessment with Track Changes

(v.1.5– April 22, 2016)

HOME BASELINE ASSESSMENT

QUESTIONNAIRES ANSWERED BY PARTICIPANTS AT BASELINE (VIA ONLINE OR PAPER FORM)

Deleted: MBHT

Deleted: December 21, 2015

Questionnaire Table of Contents

Introductory Questions....3
Background Questions....5
Physical Activity....8
Eating Practices....10
Alcohol Consumption....13
Smoking....14
About You....15
Parent's Education....37
Your Childhood Experiences....39
Chronic Illness....42
Blood Pressure Medication Use....43
More About You....44
Family History of Hypertension....48
Your Sleep....49
END SCRIPT....49

Deleted: Introductory Questions . 3¶
Background Questions . 5¶
Physical Activity . 8¶
Eating Practices . 10¶
Alcohol Consumption . 13¶
Smoking . 14¶
About You . 15¶
Parent's Education . 37¶
Your Childhood Experiences . 39¶
Blood Pressure Medication Use . 42¶
More About You . 43¶
Family History of Hypertension . 47¶
Your Sleep . 48¶
END SCRIPT . 48¶

Deleted: *MBHT*

Deleted: *December 21, 2015*

Introduction

We appreciate you taking the time to participate in this research study. These questionnaires will ask a series of questions on various aspects of your health, health behaviours, family, and other life circumstances. It should take approximately one hour. Please keep in mind that you can refuse to answer any questions that you are not comfortable with.

Introductory Questions

IQ1_01. Please enter the 4-digit ID number you were given. ___ ___ ___ ___.

IQ1_02. What is your main reason for participating in this study?

IQ1_03. What do you care about most?

IQ1_04. What gives you the most pleasure in your life?

IQ1_05. What are your greatest worries?

Deleted: MBHT

Deleted: December 21, 2015

Please list three personal goals you have for taking this mindfulness program:

PG1_01.____
____
____
____
___

PG1_02. ____
____
____
____

PG1_03. ____
____
____
____
___

Deleted: *MBHT*

Deleted: *December 21, 2015*

Background Questions

BQ1_01. How many years old are you?

BQ1_02. Are you Latino or Hispanic?

☐ No → ***skip to B3***
☐ Yes
☐ I do not know
☐ I prefer not to answer

B1_02a. Which of the following represents your family's country of origin? ***(check all that apply)***

☐ Cuba
☐ Mexico
☐ Puerto Rico
☐ Spain
☐ South America
☐ Columbia
☐ Dominican Republic
☐ Other Central American
☐ Other: ______________________
☐ I do not know
☐ I prefer not to answer

BQ1_03. If you were asked to put yourself into only one of these groups, in which one would you place yourself? ***(select one only):***

☐ Asian
☐ Pacific Islander
☐ African American/Black
☐ Caucasian/White
☐ Native American
☐ Other : ______________________
☐ I do not know
☐ I prefer not to answer

BQ1_04. Which of the following best describes your current work situation? ***(select one only)***

☐ Working full-time
☐ Working part-time
☐ Retired
☐ Unemployed: Looking for work
☐ Unemployed: Not currently looking for work
☐ Unemployed due to disability
☐ Keeping house or raising children full-time
☐ Military
☐ Full-time student
☐ Other: ______________________
☐ I do not know
☐ I prefer not to answer

Deleted: MBHT
Deleted: December 21, 2015

BQ1_05. What is the highest grade or level of regular school you have completed?

☐ Elementary School
☐ Junior High
☐ High School
☐ College
☐ Graduate School
☐ I do not know
☐ I prefer not to answer

BQ1_06. What is the highest degree you earned? ***(select <u>one</u> only)***

☐ Elementary school
☐ Some high school, but no GED
☐ GED
☐ High school
☐ Associate degree (Junior College)
☐ Bachelor's degree
☐ Master's degree
☐ Doctorate (PhD, EdD, etc)
☐ Professional (MD, JD, DDS, DVM, etc.)
☐ Other: ______________________________
☐ I do not know
☐ I prefer not to answer

BQ1_07. Did you ever attend any other school like a technical, vocational, or trade school?

☐ No
☐ Yes
☐ I do not know
☐ I prefer not to answer

BQ1_08. In total, about how many full-time years of education have you had, including 1st grade and all years of school after 1st grade?

________ years

Deleted: MBHT
Deleted: December 21, 2015

BQ1_09a. Do you currently live alone?

☐ No
☐ Yes → ***skip to next section***
☐ I do not know
☐ I prefer not to answer

BQ1_09b. How many people currently live in your household, including yourself?

____

BQ1_09c. Of these people, how many are under 18?

____

BQ1_09d. Of the adults in your household (including yourself), how many bring income into the household?

____

Deleted: *MBHT*
Deleted: *December 21, 2015*

Physical Activity

Physical activities are activities where you move and increase your heart rate above its resting rate, whether you do them for pleasure, work, or transportation. The following questions ask about the amount and intensity of physical activity you usually do. The intensity of the activity is related to *the amount of energy* you use doing these activities.

Examples of physical activity intensity levels:

Light activities
Your heart beats slightly faster than normal
You can talk and sing

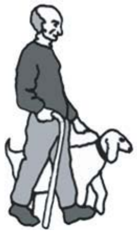

Light exercise

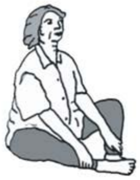

Light stretching

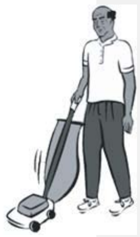

Light vacuuming or yard work

Moderate activities
Your heart beats faster than normal
You can talk but not sing

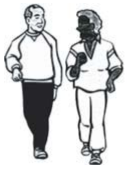

Brisk walking

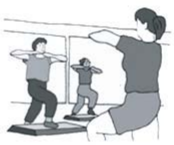

Aerobics class

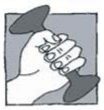

Strength training

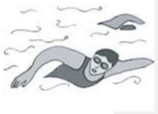

Swim gently

Vigorous activities
Your heart rate increases a lot
You can't talk, or your talking is broken up by large breaths

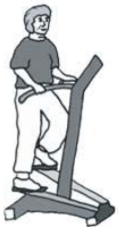

Aerobics classes

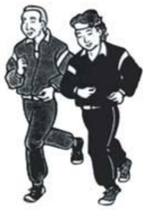

Jogging, Running, or Power Walking

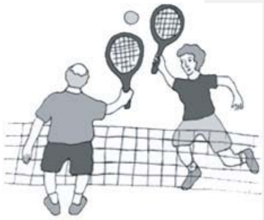

Singles tennis, Racquetball, Pickle ball

How physically active are you?

	YES	NO	I do not know	I prefer not to answer
PA1_01. I rarely or never do any physical activities.	☐	☐	☐	☐
PA1_02. I do some light and/or moderate physical activities, but not every week.	☐	☐	☐	☐
PA1_03. I do some light physical activity every week.	☐	☐	☐	☐
PA1_04. I do moderate physical activity every week but less than 5 days per week or less than 30 minutes on those days.	☐	☐	☐	☐
PA1_05. I do vigorous physical activities every week, but less than 3 days per week or less than 20 minutes on those days.	☐	☐	☐	☐
PA1_06. I do 30 minutes or more per day of moderate physical activities 5 or more days per week.	☐	☐	☐	☐
PA1_07. I do 20 minutes or more per day of vigorous physical activities 3 or more days per week.	☐	☐	☐	☐
PA1_08. I do activities to increase muscle strength, such as lifting weights or calisthenics, once a week or more.	☐	☐	☐	☐
PA1_09. I do activities to improve flexibility, such as stretching or yoga, once a week or more.	☐	☐	☐	☐

Deleted: *MBHT*

Deleted: *December 21, 2015*

Eating Practices

	Definitely True	Mostly True	Mostly False	Definitely False	I Do Not Know	I Prefer Not to Answer
EE1_01. I deliberately take small helpings to control my weight.	☐	☐	☐	☐	☐	☐
EE1_02. I start to eat when I feel anxious.	☐	☐	☐	☐	☐	☐
EE1_03. Sometimes when I start eating, I just can't seem to stop.	☐	☐	☐	☐	☐	☐
EE1_04. When I feel sad, I often eat too much.	☐	☐	☐	☐	☐	☐
EE1_05. I don't eat some foods because they make me fat.	☐	☐	☐	☐	☐	☐
EE1_06. Being with someone who is eating, often makes me want to also eat.	☐	☐	☐	☐	☐	☐
EE1_07. When I feel tense or ''wound up'', I often feel I need to eat.	☐	☐	☐	☐	☐	☐
EE1_08. I often get so hungry that my stomach feels like a bottomless pit.	☐	☐	☐	☐	☐	☐
EE1_09. I'm always so hungry that it's hard for me to stop eating before finishing all of the food on my plate.	☐	☐	☐	☐	☐	☐
EE1_10. When I feel lonely, I console myself by eating.	☐	☐	☐	☐	☐	☐
EE1_11. I consciously hold back on how much I eat at meals to keep from gaining weight.	☐	☐	☐	☐	☐	☐
EE1_12. When I smell a sizzling steak or see a juicy piece of meat, I find it very difficult to keep from eating even if I've just finished a meal.	☐	☐	☐	☐	☐	☐
EE1_13. I'm always hungry enough to eat at any time.	☐	☐	☐	☐	☐	☐
EE1_14. If I feel nervous, I try to calm down by eating.	☐	☐	☐	☐	☐	☐
EE1_15. When I see something that looks very delicious, I often get so hungry that I have to eat right away.	☐	☐	☐	☐	☐	☐
EE1_16. When I feel depressed, I want to eat.	☐	☐	☐	☐	☐	☐

Deleted: *MBHT*

Deleted: *December 21, 2015*

	Almost Never	Seldom	Usually	Almost Always	I Do Not Know	I Prefer Not to Answer
EE1_17. How often do you avoid 'stocking up'' on tempting foods?	☐	☐	☐	☐	☐	☐

	Unlikely	A little likely	Somewhat likely	Very likely	I Do Not Know	I Prefer Not to Answer
EE1_18. How likely are you to make an effort to eat less than you want?	☐	☐	☐	☐	☐	☐

	Never	Rarely	Sometimes	At least once a week	I Do Not Know	I Prefer Not to Answer
EE1_19. Do you go on eating binges even though you're not hungry?	☐	☐	☐	☐	☐	☐

	Only at mealtimes	Sometimes between meals	Often between meals	Almost always	I Do Not Know	I Prefer Not to Answer
EE1_20. How often do you feel hungry?	☐	☐	☐	☐	☐	☐

	1	2	3	4	5	6	7	8	I Do Not Know	I Prefer Not to Answer
EE1_21. On a scale from 1 to 8, where 1 means no restraint in eating and 8 means total restraint, what number would you give yourself? Mark the number that best applies to you:	☐	☐	☐	☐	☐	☐	☐	☐	☐	☐

Deleted: *MBHT*

Deleted: *December 21, 2015*

Table Salt Use:

TS1_01 Please report your average total use, during the past year, of "salt added at the table". Would you say…

Never	☐
Less than once per month	☐
1-3 shakes per month	☐
1 shake per week	☐
2-4 shakes per week	☐
5-6 shakes per week	☐
1 shake per day	☐
2-3 shakes per day	☐
4-5 shakes per day	☐
6+ shakes per day	☐

Deleted: MBHT

Deleted: December 21, 2015

Alcohol Consumption

A drink of alcohol is defined as 1 can or bottle of beer, 1 glass of wine, 1 can or bottle of wine cooler, 1 cocktail, or 1 shot of liquor.

AC1_01. During the past 30 days, how many days per week or per month did you have at least 1 drink of any alcoholic beverage? [if none, ***skip to next section***]

____

AC1_02. On the days when you drank, about how many drinks did you drink on average?

____

AC1_03. ***Men:*** Considering all types of alcoholic beverages, how many times during the past 30 days did you have **5** or more drinks on an occasion?

Women: Considering all types of alcoholic beverages, how many times during the past 30 days did you have ***4*** or more drinks on an occasion?

____

Deleted: MBHT

Deleted: December 21, 2015

Smoking

SM1_01. Have you smoked at least 100 cigarettes in your entire life?

- ☐ Yes
- ☐ No
- ☐ I Do Not Know
- ☐ Prefer not to answer

SM1_02. Did you ever become a daily smoker (that is, smoke every day or nearly every day for two months or longer)?

- ☐ Yes
- ☐ No → ***skip to next section***
- ☐ I Do Not Know
- ☐ Prefer not to answer

SM1_03. How old were you when you last smoked daily?

Age ______ (in years)

☐ I Do Not Know ☐ Prefer not to answer ☐ Still smoking daily

SM1_04. Do you smoke cigarettes now?

- ☐ Yes
- ☐ No → ***skip to next section***
- ☐ I Do Not Know
- ☐ Prefer not to answer

SM1_04a. How many cigarettes per day do you smoke? (One pack equals 20 cigarettes)

Number of cigarettes ________

- ☐ I Do Not Know
- ☐ Prefer not to answer

Deleted: *MBHT*

Deleted: *December 21, 2015*

About You

Please bring to mind a type of very tasty food that may contribute to hypertension through high salt intake or through eating too many calories (e.g., sweet sugary dessert, salty snack foods, etc.).

Think about the LAST WEEK you MOST WANTED this type of food. For each item, select a number (0 to 10) to indicate your rating.

At that time…	Not at All 0	1	2	3	4	5	6	7	8	9	Extremely 10
1. …how much did you want it?	☐	☐	☐	☐	☐	☐	☐	☐	☐	☐	☐
2. …how much did you need it?	☐	☐	☐	☐	☐	☐	☐	☐	☐	☐	☐
3. …how strong was the urge to have it?	☐	☐	☐	☐	☐	☐	☐	☐	☐	☐	☐
At that time, how vividly did you…											
4. …picture it?	☐	☐	☐	☐	☐	☐	☐	☐	☐	☐	☐
5. …imagine its taste?	☐	☐	☐	☐	☐	☐	☐	☐	☐	☐	☐
6. …imagine its smell?	☐	☐	☐	☐	☐	☐	☐	☐	☐	☐	☐
7. …imagine what it would feel like in your mouth or throat?	☐	☐	☐	☐	☐	☐	☐	☐	☐	☐	☐
8. …imagine how your body would feel?	☐	☐	☐	☐	☐	☐	☐	☐	☐	☐	☐
At that time…											
9. …how hard were you trying not to think about it?	☐	☐	☐	☐	☐	☐	☐	☐	☐	☐	☐
10. …how intrusive were the thoughts?	☐	☐	☐	☐	☐	☐	☐	☐	☐	☐	☐
11. …how hard was it to think about anything else?	☐	☐	☐	☐	☐	☐	☐	☐	☐	☐	☐

Please bring to mind any times in the LAST WEEK when you had a desire to do sedentary activities (e.g., read a book, watch a movie, be on the computer, etc.) instead of physical activities (e.g., walking, gardening, exercise).

Think about the LAST WEEK you MOST WANTED to do a sedentary activity. For each item, select a number (0 to 10) to indicate your rating.

At that time…	Not at All 0	1	2	3	4	5	6	7	8	9	Extremely 10
1. …how much did you want it?	□	□	□	□	□	□	□	□	□	□	□
2. …how much did you need it?	□	□	□	□	□	□	□	□	□	□	□
3. …how strong was the urge to have it?	□	□	□	□	□	□	□	□	□	□	□
At that time, how vividly did you…											
4. …picture it?	□	□	□	□	□	□	□	□	□	□	□
5. …imagine how your body would feel?	□	□	□	□	□	□	□	□	□	□	□
At that time…											
6. …how hard were you trying not to think about it?	□	□	□	□	□	□	□	□	□	□	□
7. …how intrusive were the thoughts?	□	□	□	□	□	□	□	□	□	□	□
8. …how hard was it to think about anything else?	□	□	□	□	□	□	□	□	□	□	□

Please bring to mind any times in the LAST WEEK when you had a desire to drink alcohol, such as wine, beer or spirits.

Think about the LAST WEEK you MOST WANTED alcohol. For each item, select a number (0 to 10) to indicate your rating.

At that time…	Not at All 0	1	2	3	4	5	6	7	8	9	Extremely 10
1. …how much did you want it?	□	□	□	□	□	□	□	□	□	□	□
2. …how much did you need it?	□	□	□	□	□	□	□	□	□	□	□
3. …how strong was the urge to have it?	□	□	□	□	□	□	□	□	□	□	□
At that time, how vividly did you…											
4. …picture it?	□	□	□	□	□	□	□	□	□	□	□
5. …imagine its taste?	□	□	□	□	□	□	□	□	□	□	□
6. …imagine its smell?	□	□	□	□	□	□	□	□	□	□	□
7. …imagine what it would feel like in your mouth or throat?	□	□	□	□	□	□	□	□	□	□	□
8. …imagine how your body would feel?	□	□	□	□	□	□	□	□	□	□	□
At that time…											
9. …how hard were you trying not to think about it?	□	□	□	□	□	□	□	□	□	□	□
10. …how intrusive were the thoughts?	□	□	□	□	□	□	□	□	□	□	□
11. …how hard was it to think about anything else?	□	□	□	□	□	□	□	□	□	□	□

Deleted: *MBHT*

Deleted: *December 21, 2015*

Using the scale provided, please indicate how much each of the following statements reflects how you typically are.

	Not at all	A little	Somewhat	Fair Amount	Very much	I do not know	I prefer not to answer
SC1_01. I am good at resisting temptation.	☐	☐	☐	☐	☐	☐	☐
SC1_02. I have a hard time breaking bad habits.	☐	☐	☐	☐	☐	☐	☐
SC1_03. I am lazy.	☐	☐	☐	☐	☐	☐	☐
SC1_04. I say inappropriate things.	☐	☐	☐	☐	☐	☐	☐
SC1_05. I do certain things that are bad for me, if they are fun.	☐	☐	☐	☐	☐	☐	☐
SC1_06. I refuse things that are bad for me.	☐	☐	☐	☐	☐	☐	☐
SC1_07. I wish I had more self-discipline.	☐	☐	☐	☐	☐	☐	☐
SC1_08. People would say that I have iron self-discipline.	☐	☐	☐	☐	☐	☐	☐
SC1_09. Pleasure and fun sometimes keep me from getting work done.	☐	☐	☐	☐	☐	☐	☐
SC1_10. I have trouble concentrating.	☐	☐	☐	☐	☐	☐	☐
SC1_11. I am able to work effectively toward long-term goals.	☐	☐	☐	☐	☐	☐	☐
SC1_12. Sometimes I can't stop myself from doing something, even if I know it is wrong.	☐	☐	☐	☐	☐	☐	☐
SC1_13. I often act without thinking through all the alternatives.	☐	☐	☐	☐	☐	☐	☐

Deleted: *MBHT*
Deleted: *December 21, 2015*

Please read each statement carefully before answering. To the left of each item, indicate how often you behave in the stated manner, using the following scale:

	Almost never	Not very often	Sometimes	Frequently	Almost always	I Do Not Know	Prefer not to answer
CO1_O1. When I fail at something important to me, I become consumed by feelings of inadequacy.							
CO1_02. I try to be understanding and patient towards those aspects of my personality I don't like.							
CO1_O3. When something painful happens I try to take a balanced view of the situation.							
CO1_O4. When I'm feeling down, I tend to feel like most other people are probably happier than I am.							
CO1_O5. I try to see my failings as part of the human condition.							
CO1_O6. When I'm going through a very hard time, I give myself the caring and tenderness I need.							
CO1_O7. When something upsets me I try to keep my emotions in balance.							
CO1_O8. When I fail at something that's important to me, I tend to feel alone in my failure							
CO1_O9. When I'm feeling down I tend to obsess and fixate on everything that's wrong.							

Deleted: *MBHT*

Deleted: *December 21, 2015*

	Almost never	Not very often	Sometimes	Frequently	Almost always	I Do Not Know	Prefer not to answer
CO1_10. When I feel inadequate in some way, I try to remind myself that feelings of inadequacy are shared by most people.							
CO1_11. I'm disapproving and judgmental about my own flaws and inadequacies.							
CO1_12. I'm intolerant and impatient towards those aspects of my personality I don't like.							

Deleted: MBHT

Deleted: December 21, 2015

The questions in this scale ask you about your feelings and thoughts during the last month. In each case, you will be asked to indicate by circling how often you felt or thought a certain way.

	Never	Almost Never	Sometimes	Fairly Often	Often	I do not know	I prefer not to answer
PS1_01. In the last month, how often have you been upset because of something that happened unexpectedly?	☐	☐	☐	☐	☐	☐	☐
PS1_02. In the last month, how often have you felt that you were unable to control the important things in your life?	☐	☐	☐	☐	☐	☐	☐
PS1_03. In the last month, how often have you felt nervous and "stressed"?	☐	☐	☐	☐	☐	☐	☐
PS1_04. In the last month, how often have you felt confident about your ability to handle your personal problems?	☐	☐	☐	☐	☐	☐	☐
PS1_05. In the last month, how often have you felt that things were going your way?	☐	☐	☐	☐	☐	☐	☐
PS1_06. In the last month, how often have you found that you could not cope with all the things that you had to do?	☐	☐	☐	☐	☐	☐	☐
PS1_07. In the last month, how often have you been able to control irritations in your life?	☐	☐	☐	☐	☐	☐	☐
PS1_08. In the last month, how often have you felt that you were on top of things?	☐	☐	☐	☐	☐	☐	☐
PS1_09. In the last month, how often have you been angered because of things that were outside of your control?	☐	☐	☐	☐	☐	☐	☐
PS1_10. In the last month, how often have you felt difficulties were piling up so high that you could not overcome them?	☐	☐	☐	☐	☐	☐	☐

Deleted: *MBHT*

Deleted: *December 21, 2015*

Please indicate how often the following statements apply to you by checking the box that best describes your experience.

	Almost Never (0-10%)	Sometimes (11-35%)	About Half The Time (36-65%)	Most of the Time (66-90%)	Almost Always (91-100%)	I Do Not Know	I Prefer Not to Answer
ER1_01. I am clear about my feelings.	☐	☐	☐	☐	☐	☐	☐
ER1_02. I pay attention to how I feel.	☐	☐	☐	☐	☐	☐	☐
ER1_03. I experience my emotions as overwhelming and out of control.	☐	☐	☐	☐	☐	☐	☐
ER1_04. I have no idea how I am feeling.	☐	☐	☐	☐	☐	☐	☐
ER1_05. I have difficulty making sense out of my feelings.	☐	☐	☐	☐	☐	☐	☐
ER1_06. I am attentive to my feelings.	☐	☐	☐	☐	☐	☐	☐
ER1_07. I know exactly how I am feeling.	☐	☐	☐	☐	☐	☐	☐
ER1_08. I care about what I am feeling.	☐	☐	☐	☐	☐	☐	☐
ER1_09. I am confused about how I feel.	☐	☐	☐	☐	☐	☐	☐
ER1_10. When I'm upset, I acknowledge my emotions.	☐	☐	☐	☐	☐	☐	☐
ER1_11. When I'm upset, I become angry with myself for feeling that way.	☐	☐	☐	☐	☐	☐	☐
ER1_12. When I'm upset, I become embarrassed for feeling that way.	☐	☐	☐	☐	☐	☐	☐

Deleted: *MBHT*

Deleted: *December 21, 2015*

	Almost Never (0-10%)	Sometimes (11-35%)	About Half The Time (36-65%)	Most of the Time (66-90%)	Almost Always (91-100%)	I Do Not Know	I Prefer Not to Answer
ER1_13. When I'm upset, I have difficulty getting work done.	☐	☐	☐	☐	☐	☐	☐
ER1_14. When I'm upset, I become out of control.	☐	☐	☐	☐	☐	☐	☐
ER1_15. When I'm upset, I believe that I will remain that way for a long time.	☐	☐	☐	☐	☐	☐	☐
ER1_16. When I'm upset, I believe that I will end up feeling very depressed.	☐	☐	☐	☐	☐	☐	☐
ER1_17. When I'm upset, I believe that my feelings are valid and important.	☐	☐	☐	☐	☐	☐	☐
ER1_18. When I'm upset, I have difficulty focusing on other things.	☐	☐	☐	☐	☐	☐	☐
ER1_19. When I'm upset, I feel out of control.	☐	☐	☐	☐	☐	☐	☐
ER1_20. When I'm upset, I can still get things done.	☐	☐	☐	☐	☐	☐	☐
ER1_21. When I'm upset, I feel ashamed at myself for feeling that way.	☐	☐	☐	☐	☐	☐	☐
ER1_22. When I'm upset, I know that I can find a way to eventually feel better.	☐	☐	☐	☐	☐	☐	☐

Deleted: *MBHT*

Deleted: *December 21, 2015*

	Almost Never (0-10%)	Sometimes (11-35%)	About Half The Time (36-65%)	Most of the Time (66-90%)	Almost Always (91-100%)	I Do Not Know	I Prefer Not to Answer
ER1_23. When I'm upset, I feel like I am weak.	☐	☐	☐	☐	☐	☐	☐
ER1_24. When I'm upset, I feel like I can remain in control of my behaviours.	☐	☐	☐	☐	☐	☐	☐
ER1_25. When I'm upset, I feel guilty for feeling that way.	☐	☐	☐	☐	☐	☐	☐
ER1_26. When I'm upset, I have difficulty concentrating.	☐	☐	☐	☐	☐	☐	☐
ER1_27. When I'm upset, I have difficulty controlling my behaviours.	☐	☐	☐	☐	☐	☐	☐
ER1_28. When I'm upset, I believe there is nothing I can do to make myself feel better.	☐	☐	☐	☐	☐	☐	☐
ER1_29. When I'm upset, I become irritated at myself for feeling that way.	☐	☐	☐	☐	☐	☐	☐
ER1_30. When I'm upset, I start to feel very bad about myself.	☐	☐	☐	☐	☐	☐	☐
ER1_31. When I'm upset, I believe that wallowing in it is all I can do.	☐	☐	☐	☐	☐	☐	☐
ER1_32. When I'm upset, I lose control over my behaviour.	☐	☐	☐	☐	☐	☐	☐

Deleted: MBHT
Deleted: December 21, 2015

	Almost Never (0-10%)	Sometimes (11-35%)	About Half The Time (36-65%)	Most of the Time (66-90%)	Almost Always (91-100%)	I Do Not Know	I Prefer Not to Answer
ER1_33. When I'm upset, I have difficulty thinking about anything else.	☐	☐	☐	☐	☐	☐	☐
ER1_34. When I'm upset I take time to figure out what I'm really feeling.	☐	☐	☐	☐	☐	☐	☐
ER1_35. When I'm upset, it takes me a long time to feel better.	☐	☐	☐	☐	☐	☐	☐
ER1_36. When I'm upset, my emotions feel overwhelming.	☐	☐	☐	☐	☐	☐	☐

Deleted: *MBHT*

Deleted: *December 21, 2015*

This scale is made up of a list of statements each of which may or may not be true about you. For each statement, select "definitely true" if you are sure it is true about you and "probably true" if you think it is true but are not absolutely certain. Similarly, you should select "definitely false" if you are sure the statement is false and "probably false" if you think it is false but are not absolutely certain.

	Definitely False	Probably False	Probably True	Definitely True	I do not know	I prefer not to answer
**IS1_01** If I wanted to go on a trip for a day (for example, to the country or mountains), I would have a hard time finding someone to go with me.	☐	☐	☐	☐	☐	☐
**IS1_02** I feel that there is no one I can share my most private worries and fears with.	☐	☐	☐	☐	☐	☐
**IS1_03** If I were sick, I could easily find someone to help me with my daily chores.	☐	☐	☐	☐	☐	☐
**IS1_04** There is someone I can turn to for advice about handling problems with my family.	☐	☐	☐	☐	☐	☐
**IS1_05** If I decide one afternoon that I would like to go to a movie that evening, I could easily find someone to go with me.	☐	☐	☐	☐	☐	☐
**IS1_06** When I need suggestions on how to deal with a personal problem, I know someone I can turn to.	☐	☐	☐	☐	☐	☐
**IS1_07** I don't often get invited to do things with others.	☐	☐	☐	☐	☐	☐
**IS1_08** If I had to go out of town for a few weeks, it would be difficult to find someone who would look after my house or apartment (the plants, pets, garden, etc.).	☐	☐	☐	☐	☐	☐
**IS1_09** If I wanted to have lunch with someone, I could easily find someone to join me.	☐	☐	☐	☐	☐	☐

Deleted: MBHT
Deleted: December 21, 2015

	Definitely False	Probably False	Probably True	Definitely True	I do not know	I prefer not to answer
**IS1_10** If I was stranded 10 miles from home, there is someone I could call who could come and get me.	☐	☐	☐	☐	☐	☐
**IS1_11** If a family crisis arose, it would be difficult to find someone who could give me good advice about how to handle it.	☐	☐	☐	☐	☐	☐
**IS1_12** If I needed some help in moving to a new house or apartment, I would have a hard time finding someone to help me.	☐	☐	☐	☐	☐	☐

Deleted: *MBHT*

Deleted: *December 21, 2015*

Please indicate how often each of the statements below is descriptive of you.

**Statement**	Never	Rarely	Sometimes	Often	I Do Not Know	Prefer not to answer
LS1_01. I feel in tune with the people around me	☐	☐	☐	☐	☐	s
LS1_02. I lack companionship	☐	☐	☐	☐	☐	☐
LS1_03. There is no one I can turn to	☐	☐	☐	☐	☐	☐
LS1_04. I do not feel alone	☐	☐	☐	☐	☐	☐
LS1_05. I feel part of a group of friends	☐	☐	☐	☐	☐	☐
LS1_06. I have a lot in common with the people around me	☐	☐	☐	☐	☐	☐
LS1_07. I am no longer close to anyone	☐	☐	☐	☐	☐	☐
LS1_08. My interests and ideas are not shared by those around me	☐	☐	☐	☐	☐	☐
LS1_09. I am an outgoing person	☐	☐	☐	☐	☐	☐
LS1_10. There are people I feel close to	☐	☐	☐	☐	☐	☐
LS1_11. I feel left out	☐	☐	☐	☐	☐	☐
LS1_12. My social relationships are superficial	☐	☐	☐	☐	☐	☐
LS1_13. No one really knows me well	☐	☐	☐	☐	☐	☐
LS1_14. I feel isolated from others	☐	☐	☐	☐	☐	☐
LS1_15. I can find companionship when I want it	☐	☐	☐	☐	☐	☐
LS1_16. There are people who really understand me	☐	☐	☐	☐	☐	☐
LS1_17. I am unhappy being so withdrawn	☐	☐	☐	☐	☐	☐
LS1_18. People are around me but not with me	☐	☐	☐	☐	☐	☐
LS1_19. There are people I can talk to	☐	☐	☐	☐	☐	☐
LS1_20. There are people I can turn to	☐	☐	☐	☐	☐	☐

Deleted: *MBHT*
Deleted: *December 21, 2015*

Below you will find a list of statements. Please indicate how often each statement applies to you generally in daily life.

	0- Never	1	2	3	4	5 - Always
IA1_01. When I am tense I notice where the tension is located in my body.	☐	☐	☐	☐	☐	☐
IA1_02. I notice when I am uncomfortable in my body.	☐	☐	☐	☐	☐	☐
IA1_03. I notice where in my body I am comfortable.	☐	☐	☐	☐	☐	☐
IA1_04. I notice changes in my breathing, such as whether it slows down or speeds up.	☐	☐	☐	☐	☐	☐
IA1_05. I do not notice (I ignore) physical tension or discomfort until they become more severe.	☐	☐	☐	☐	☐	☐
IA1_06. I distract myself from sensations of discomfort.	☐	☐	☐	☐	☐	☐
IA1_07. When I feel pain or discomfort, I try to power through it.	☐	☐	☐	☐	☐	☐
IA1_08. When I feel physical pain, I become upset.	☐	☐	☐	☐	☐	☐
IA1_09. I start to worry that something is wrong if I feel any discomfort.	☐	☐	☐	☐	☐	☐
IA1_10. I can notice an unpleasant body sensation without worrying about it.	☐	☐	☐	☐	☐	☐
IA1_11. I can pay attention to my breath without being distracted by things happening around me.	☐	☐	☐	☐	☐	☐
IA1_12. I can maintain awareness of my inner bodily sensations even when there is a lot going on around me.	☐	☐	☐	☐	☐	☐
IA1_13. When I am in conversation with someone, I can pay attention to my posture.	☐	☐	☐	☐	☐	☐
IA1_14. I can return awareness to my body if I am distracted.	☐	☐	☐	☐	☐	☐
IA1_15. I can refocus my attention from thinking to sensing my body.	☐	☐	☐	☐	☐	☐
IA1_16. I can maintain awareness of my whole body even when a part of me is in pain or discomfort.	☐	☐	☐	☐	☐	☐
IA1_17. I am able to consciously focus on my body as a whole.	☐	☐	☐	☐	☐	☐
IA1_18. I notice how my body changes when I am angry.	☐	☐	☐	☐	☐	☐
IA1_19. When something is wrong in my life I can feel it in my body.	☐	☐	☐	☐	☐	☐
IA1_20. I notice that my body feels different after a peaceful experience.	☐	☐	☐	☐	☐	☐
IA1_21. I notice that my breathing becomes free and easy when I feel comfortable.	☐	☐	☐	☐	☐	☐
IA1_22. I notice how my body changes when I feel happy / joyful.	☐	☐	☐	☐	☐	☐

Deleted: *MBHT*

Deleted: *December 21, 2015*

	0- Never	1	2	3	4	5 - Always
IA1_23. When I feel overwhelmed I can find a calm place inside.	☐	☐	☐	☐	☐	☐
IA1_24. When I bring awareness to my body I feel a sense of calm.	☐	☐	☐	☐	☐	☐
IA1_25. I can use my breath to reduce tension.	☐	☐	☐	☐	☐	☐
IA1_26. When I am caught up in thoughts, I can calm my mind by focusing on my body/breathing.	☐	☐	☐	☐	☐	☐
IA1_27. I listen for information from my body about my emotional state.	☐	☐	☐	☐	☐	☐
IA1_28. When I am upset, I take time to explore how my body feels.	☐	☐	☐	☐	☐	☐
IA1_29. I listen to my body to inform me about what to do.	☐	☐	☐	☐	☐	☐
IA1_30. I am at home in my body.	☐	☐	☐	☐	☐	☐
IA1_31. I feel my body is a safe place.	☐	☐	☐	☐	☐	☐
IA1_32. I trust my body sensations.	☐	☐	☐	☐	☐	☐

Deleted: *MBHT*

Deleted: *December 21, 2015*

We are interested in your recent experiences. Below is a list of things that people sometimes experience. Next to each item are five choices: “never”, “rarely”, “sometimes”, “often”, and “all the time”. Please choose one of these to indicate how much you currently have experiences similar to those described.

Please do not spend too long on each item–it is your first response that we are interested in. Please be sure to answer every item.

	Never	Rarely	Sometimes	Often	All the time
RD1_01. I think about what will happen in the future.	☐	☐	☐	☐	☐
RD1_02. I remind myself that thoughts aren’t facts.	☐	☐	☐	☐	☐
RD1_03. I am better able to accept myself as I am.	☐	☐	☐	☐	☐
RD1_04. I notice all sorts of little things and details in the world around me.	☐	☐	☐	☐	☐
RD1_05. I am kinder to myself when things go wrong.	☐	☐	☐	☐	☐
RD1_06. I can slow my thinking at times of stress.	☐	☐	☐	☐	☐
RD1_07. I wonder what kind of person I really am.	☐	☐	☐	☐	☐
RD1_08. I am not so easily carried away by my thoughts and feelings.	☐	☐	☐	☐	☐
RD1_09. I notice that I don’t take difficulties so personally.	☐	☐	☐	☐	☐
RD1_10. I can separate myself from my thoughts and feelings.	☐	☐	☐	☐	☐
RD1_11. I analyze why things turn out the way they do.	☐	☐	☐	☐	☐
RD1_12. I can take time to respond to difficulties.	☐	☐	☐	☐	☐
RD1_13. I think over and over again about what others have said to me.	☐	☐	☐	☐	☐
RD1_14. I can treat myself kindly.	☐	☐	☐	☐	☐
RD1_15. I can observe unpleasant feelings without being drawn into them.	☐	☐	☐	☐	☐
RD1_16. I have the sense that I am fully aware of what is going on around me and inside me.	☐	☐	☐	☐	☐
RD1_17. I can actually see that I am not my thoughts.	☐	☐	☐	☐	☐
RD1_18. I am consciously aware of a sense of my body as a whole.	☐	☐	☐	☐	☐
RD1_19. I think about the ways in which I am different from other people.	☐	☐	☐	☐	☐
RD1_20. I view things from a wider perspective.	☐	☐	☐	☐	☐

Deleted: *MBHT*

Deleted: *December 21, 2015*

How do you cope with events? Everyone gets confronted with negative or unpleasant events now and then and everyone responds to them in his or her own way. By the following questions you are asked to indicate what you generally think, when you experience negative or unpleasant events.

	(Almost) Never	Some-times	Regularly	Often	(Almost) Always
CR1_01. I think that I have to accept that this has happened.	□	□	□	□	□
CR1_02. I often think about how I feel about what I have experienced.	□	□	□	□	□
CR1_03. I think I can learn something from the situation.	□	□	□	□	□
CR1_04. I feel that I am the one who is responsible for what has happened.	□	□	□	□	□
CR1_05. I think that I have to accept the situation.	□	□	□	□	□
CR1_06. I am preoccupied with what I think and feel about what I have experienced.	□	□	□	□	□
CR1_07. I think of pleasant things that have nothing to do with it.	□	□	□	□	□
CR1_08. I think that I can become a stronger person as a result of what has happened.	□	□	□	□	□
CR1_09. I keep thinking about how terrible it is what I have experienced.	□	□	□	□	□
CR1_10. I feel that others are responsible for what has happened.	□	□	□	□	□
CR1_11. I think of something nice instead of what has happened.	□	□	□	□	□
CR1_12. I think about how to change the situation.	□	□	□	□	□
CR1_13. I think that it hasn't been too bad compared to other things.	□	□	□	□	□
CR1_14. I think that basically the cause must lie within myself.	□	□	□	□	□
CR1_15. I think about a plan of what I can do best.	□	□	□	□	□
CR1_16. I tell myself that there are worse things in life.	□	□	□	□	□
CR1_17. I continually think how horrible the situation has been.	□	□	□	□	□
CR1_18. I feel that basically the cause lies with others.	□	□	□	□	□

Deleted: *MBHT*
Deleted: *December 21, 2015*

Instructions: Below is a collection of statements about your everyday experience. Using the scale below, please indicate how frequently or infrequently you currently have each experience. Please answer according to what really reflects your experience rather than what you think your experience should be. Please treat each item separately from every other item.

Please indicate the degree to which you agree with each of the following items using the scale below. Simply check your response to each item

	Almost always	Very frequently	Somewhat frequently	Somewhat infrequently	Very infrequently	Almost never	I do not know	I prefer not to answer
MA1_01. I could be experiencing some emotion and not be conscious of it until some time later.	☐	☐	☐	☐	☐	☐	☐	☐
MA1_02. I break or spill things because of carelessness, not paying attention, or thinking of something else.	☐	☐	☐	☐	☐	☐	☐	☐
MA1_03. I find it difficult to stay focused on what's happening in the present.	☐	☐	☐	☐	☐	☐	☐	☐
MA1_04. I tend to walk quickly to get where I'm going without paying attention to what I experience along the way.	☐	☐	☐	☐	☐	☐	☐	☐
MA1_05. I tend not to notice feelings of physical tension or discomfort until they really grab my attention.	☐	☐	☐	☐	☐	☐	☐	☐
MA1_06. I forget a person's name almost as soon as I've been told it for the first time.	☐	☐	☐	☐	☐	☐	☐	☐

Deleted: MBHT

Deleted: December 21, 2015

	Almost always	Very frequently	Somewhat frequently	Somewhat infrequently	Very infrequently	Almost never	I do not know	I prefer not to answer
MA1_07. It seems I am “running on automatic” without much awareness of what I’m doing.	☐	☐	☐	☐	☐	☐	☐	☐
MA1_08. I rush through activities without being really attentive to them.	☐	☐	☐	☐	☐	☐	☐	☐
MA1_09. I get so focused on the goal I want to achieve that I lose touch with what I am doing right now to get there.	☐	☐	☐	☐	☐	☐	☐	☐
MA1_10. I do jobs or tasks automatically, without being aware of what I’m doing.	☐	☐	☐	☐	☐	☐	☐	☐
MA1_11. I find myself listening to someone with one ear, doing something else at the same time.	☐	☐	☐	☐	☐	☐	☐	☐
MA1_12. I drive places on “automatic pilot” and then wonder why I went there.	☐	☐	☐	☐	☐	☐	☐	☐
MA1_13. I find myself preoccupied with the future or the past.	☐	☐	☐	☐	☐	☐	☐	☐
MA1_14. I find myself doing things without paying attention.	☐	☐	☐	☐	☐	☐	☐	☐
MA1_15. I snack without being aware that I’m eating.	☐	☐	☐	☐	☐	☐	☐	☐

Deleted: MBHT

Deleted: December 21, 2015

Please rate each of the following statements using the scale provided. Write the number in the blank that best describes *your own opinion* of what is *generally true for you*.

	Never	Almost Never	Sometimes	Fairly Often	Often	I do not know	I prefer not to answer
FF1_01. When I'm walking, I deliberately notice the sensations of my body moving.	☐	☐	☐	☐	☐	☐	☐
FF1_02. I'm good at finding words to describe my feelings.	☐	☐	☐	☐	☐	☐	☐
FF1_03. I criticize myself for having irrational or inappropriate emotions.	☐	☐	☐	☐	☐	☐	☐
FF1_04. I perceive my feelings and emotions without having to react to them.	☐	☐	☐	☐	☐	☐	☐
FF1_05. When I do things, my mind wanders off and I'm easily distracted.	☐	☐	☐	☐	☐	☐	☐
FF1_06. When I take a shower or bath, I stay alert to the sensations of water on my body.	☐	☐	☐	☐	☐	☐	☐
FF1_07. I can easily put my beliefs, opinions, and expectations into words.	☐	☐	☐	☐	☐	☐	☐
FF1_08. I don't pay attention to what I'm doing because I'm daydreaming, worrying, or otherwise distracted.	☐	☐	☐	☐	☐	☐	☐
FF1_09. I watch my feelings without getting lost in them.	☐	☐	☐	☐	☐	☐	☐
FF1_10. I tell myself I shouldn't be feeling the way I'm feeling.	☐	☐	☐	☐	☐	☐	☐
FF1_11. I notice how foods and drinks affect my thoughts, bodily sensations, and emotions.	☐	☐	☐	☐	☐	☐	☐
FF1_12. It's hard for me to find the words to describe what I'm thinking.	☐	☐	☐	☐	☐	☐	☐
FF1_13. I am easily distracted.	☐	☐	☐	☐	☐	☐	☐
FF1_14. I believe some of my thoughts are abnormal or bad and I shouldn't think that way.	☐	☐	☐	☐	☐	☐	☐
FF1_15. I pay attention to sensations, such as the wind in my hair or sun on my face.	☐	☐	☐	☐	☐	☐	☐

Deleted: *MBHT*

Deleted: *December 21, 2015*

	Never	Almost Never	Sometimes	Fairly Often	Often	I do not know	I prefer not to answer
FF1_16. I have trouble thinking of the right words to express how I feel about things.	☐	☐	☐	☐	☐	☐	☐
FF1_17. I make judgments about whether my thoughts are good or bad.	☐	☐	☐	☐	☐	☐	☐
FF1_18. I find it difficult to stay focused on what's happening in the present.	☐	☐	☐	☐	☐	☐	☐
FF1_19. When I have distressing thoughts or images, I "step back" and am aware of the thought or image without getting taken over by it.	☐	☐	☐	☐	☐	☐	☐
FF1_20. I pay attention to sounds, such as clocks ticking, birds chirping, or cars passing.	☐	☐	☐	☐	☐	☐	☐
FF1_21. In difficult situations, I can pause without immediately reacting.	☐	☐	☐	☐	☐	☐	☐
FF1_22. When I have a sensation in my body, it's difficult for me to describe it because I can't find the right words.	☐	☐	☐	☐	☐	☐	☐
FF1_23. It seems I am "running on automatic" without much aware- ness of what I'm doing.	☐	☐	☐	☐	☐	☐	☐
FF1_24. When I have distressing thoughts or images, I feel calm soon after.	☐	☐	☐	☐	☐	☐	☐
FF1_25. I tell myself that I shouldn't be thinking the way I'm thinking.	☐	☐	☐	☐	☐	☐	☐
FF1_26. I notice the smells and aromas of things.	☐	☐	☐	☐	☐	☐	☐
FF1_27. Even when I'm feeling terribly upset, I can find a way to put it into words.	☐	☐	☐	☐	☐	☐	☐
FF1_28. I rush through activities without being really attentive to them.	☐	☐	☐	☐	☐	☐	☐

Deleted: *MBHT*

Deleted: *December 21, 2015*

	Never	Almost Never	Sometimes	Fairly Often	Often	I do not know	I prefer not to answer
FF1_29. When I have distressing thoughts or images, I am able just to notice them without reacting.	☐	☐	☐	☐	☐	☐	☐
FF1_30. I think some of my emotions are bad or inappropriate and I shouldn't feel them.	☐	☐	☐	☐	☐	☐	☐
FF1_31. I notice visual elements in art or nature, such as colors, shapes, textures, or patterns of light and shadow.	☐	☐	☐	☐	☐	☐	☐
FF1_32. My natural tendency is to put my experiences into words.	☐	☐	☐	☐	☐	☐	☐
FF1_33. When I have distressing thoughts or images, I just notice them and let them go.	☐	☐	☐	☐	☐	☐	☐
FF1_34. I do jobs or tasks automatically without being aware of what I'm doing.	☐	☐	☐	☐	☐	☐	☐
FF1_35. When I have distressing thoughts or images, I judge myself as good or bad depending what the thought or image is about.	☐	☐	☐	☐	☐	☐	☐
FF1_36. I pay attention to how my emotions affect my thoughts and behavior.	☐	☐	☐	☐	☐	☐	☐
FF1_37. I can usually describe how I feel at the moment in consider- able detail.	☐	☐	☐	☐	☐	☐	☐
FF1_38. I find myself doing things without paying attention.	☐	☐	☐	☐	☐	☐	☐
FF1_39. I disapprove of myself when I have irrational ideas.	☐	☐	☐	☐	☐	☐	☐

Deleted: *MBHT*

Deleted: *December 21, 2015*

Parent's Education

CS1_01. Please check the box beside the highest grade or degree that your BIOLOGICAL MOTHER completed.

Never went to school	☐
Grades 1 to 3	☐
Grades 4 to 8	☐
Grades 9 to 11	☐
Grade 12	☐
GED	☐
One or more years of Vocational or Professional School after High School	☐
One or more years of College	☐
One or more years of Graduate or Professional School after College	☐
I Do Not Know	☐
I prefer not to answer	☐

CS1_02. Please check the box beside the highest grade or degree that your BIOLOGICAL FATHER completed.

Never went to school	☐
Grades 1 to 3	☐
Grades 4 to 8	☐
Grades 9 to 11	☐
Grade 12	☐
GED	☐
One or more years of Vocational or Professional School after High School	☐
One or more years of College	☐
One or more years of Graduate or Professional School after College	☐
I Do Not Know	☐
I prefer not to answer	☐

Deleted: MBHT

Deleted: December 21, 2015

Now please think of the two most important adults in your home between the time you were born and age 18 years. Please check the category below that best described their level of education during this time period.

CS1_03. First adult's highest level of education:

Never went to school	☐
Grades 1 to 3	☐
Grades 4 to 8	☐
Grades 9 to 11	☐
Grade 12	☐
GED	☐
One or more years of Vocational or Professional School after High School	☐
One or more years of College	☐
One or more years of Graduate or Professional School after College	☐
I Do Not Know	☐
I prefer not to answer	☐

CS1_04. Second adult's highest level of education

Never went to school	☐
Grades 1 to 3	☐
Grades 4 to 8	☐
Grades 9 to 11	☐
Grade 12	☐
GED	☐
One or more years of Vocational or Professional School after High School	☐
One or more years of College	☐
One or more years of Graduate or Professional School after College	☐
I Do Not Know	☐
I prefer not to answer	☐

Deleted: MBHT

Deleted: December 21, 2015

Your Childhood Experiences

The following questions ask about some difficult experiences that you might have had as a child. These questions may be emotionally difficult to answer. Just as a reminder, you do not need answer any questions that you would prefer not to. Your answers to these questions, as with all questions, will remain confidential.

CE1_01. Before you were 18 years old, did a parent or other adult in the household often or very often…

Swear at you, insult you, put you down, or humiliate you?
or
Act in a way that made you afraid that you might be physically hurt?

- □ No
- □ Yes
- □ I do not know
- □ I prefer not to answer

CE1_02. Before you were 18 years old, did a parent or other adult in the household often or very often…

Push, grab, slap, or throw something at you?
or
Ever hit you so hard that you had marks or were injured?

- □ No
- □ Yes
- □ I do not know
- □ I prefer not to answer

CE1_03. Before you were 18 years old, did an adult or person at least 5 years older than you ever…

Touch or fondle you or have you touch their body in a sexual way?
or
Attempt or actually have oral, anal, or vaginal intercourse with you?

- □ No
- □ Yes
- □ I do not know
- □ I prefer not to answer

Deleted: *MBHT*
Deleted: *December 21, 2015*

CE1_04.Before you were 18 years old, did you often or very often feel that …

No one in your family loved you or thought you were important or special?
or
Your family didn't look out for each other, feel close to each other, or support each other?

- □ No
- □ Yes
- □ I do not know
- □ I prefer not to answer

CE1_05.Before you were 18 years old, did you often or very often feel that …

You didn't have enough to eat, had to wear dirty clothes, and had no one to protect you?
or
Your parents were too drunk or high to take care of you or take you to the doctor if you needed it?

- □ No
- □ Yes
- □ I do not know
- □ I prefer not to answer

CE1_06.Before you were 18 years old, was a biological parent ever lost to you through divorce, abandonment, or other reason?

- □ No
- □ Yes
- □ I do not know
- □ I prefer not to answer

CE1_07.Before you were 18 years old, was your mother or stepmother:

Often or very often pushed, grabbed, slapped, or had something thrown at her?
or
Sometimes, often, or very often kicked, bitten, hit with a fist, or hit with something hard?
or
Ever repeatedly hit over at least a few minutes or threatened with a gun or knife?

- □ No
- □ Yes
- □ I do not know
- □ I prefer not to answer

Deleted: *MBHT*
Deleted: *December 21, 2015*

CE1_08. Before you were 18 years old, did you live with anyone who was a problem drinker or alcoholic, or who used street drugs?

- □ No
- □ Yes
- □ I do not know
- □ I prefer not to answer

CE1_09. Before you were 18 years old, was a household member depressed or mentally ill, or did a household member attempt suicide?

- □ No
- □ Yes
- □ I do not know
- □ I prefer not to answer

CE1_10. Before you were 18 years old, did a household member go to prison?

- □ No
- □ Yes
- □ I do not know
- □ I prefer not to answer

Deleted: MBHT

Deleted: December 21, 2015

Chronic Illness

CD1_01. Do you have a chronic illness, health problem or disease?

□ Yes

□ No (if responding "no", please skip to the next section).

CD1_02. Please select the illness from the list below that has the greatest effect on your life or feels like the *most important* for you to be able to manage. You may have more than one, but the purpose of this question is to identify what you might view as your PRIMARY chronic illness/problem/disease.

- ☐ Diabetes
- ☐ Heart Disease
- ☐ Hypertension
- ☐ Obesity
- ☐ Metabolic Syndrome
- ☐ Arthritis
- ☐ Chronic Pain
- ☐ Asthma
- ☐ COPD
- ☐ Depression
- ☐ Anxiety
- ☐ Insomnia
- ☐ Substance use
- ☐ Tobacco use
- ☐ Alcohol overuse
- ☐ Prescription medication overuse
- ☐ Illicit drug use
- ☐ Other ______________________

We would like to know how confident you are in doing certain activities related to the chronic illness or disease you selected above. For each of the following questions, please choose the number that corresponds to your confidence that you can do the tasks regularly at the present time. Please keep the chronic illness or disease in mind as you answer the following questions.

How confident are you that….	Not at All Confident 1	2	3	4	5	6	7	8	9	Totally Confident 10
CD1_03a …you can keep the fatigue caused by your disease from interfering with the things you want to do?	□	□	□	□	□	□	□	□	□	□
CD1_03b …you can keep the physical discomfort or pain of your disease from interfering with the things you want to do?	□	□	□	□	□	□	□	□	□	□
CD1_03c …you can keep the emotional distress caused by your disease from interfering with the things you want to do?	□	□	□	□	□	□	□	□	□	□
CD1_03d …you can keep any other symptoms or health problems you have from interfering with the things you want to do?	□	□	□	□	□	□	□	□	□	□
CD1_03e …you can do the different tasks and activities needed to manage your health condition so as to reduce you need to see a doctor?	□	□	□	□	□	□	□	□	□	□
CD1_03f …you can do things other than just taking medication to reduce how much your illness affects your everyday life?	□	□	□	□	□	□	□	□	□	□

Comment [SF1]: This is a new scale. It was added so that our study findings can be analyzed and compared to the other concurrent studies.

Formatted: Font: 11 pt

Deleted: *MBHT*

Deleted: *December 21, 2015*

Blood Pressure Medication Use

BM1_01. Do you currently take medication for your blood pressure?
□ Yes
□ No (if responding "no", please skip to the next page).

	Yes	No
BM1_02. Do you sometimes forget to take your blood pressure pills?	□	□
BM1_03. People sometimes miss taking their medications for reasons other than forgetting. Thinking over the past two weeks, were there any days when you did not take your blood pressure medicine?	□	□
BM1_04. Have you ever cut back or stopped taking your blood pressure medicine without telling your doctor because you felt worse when you took it?	□	□
BM1_05. When you travel or leave home, do you sometimes forget to bring along your blood pressure medicine?	□	□
BM1_06. Did you take all your blood pressure medicine yesterday?	□	□
BM1_07. When you feel like your symptoms are under control, do you sometimes stop taking your blood pressure medicine?	□	□
BM1_08. Taking blood pressure medicine every day is a real inconvenience for some people. Do you ever feel hassled about sticking to your treatment plan?	□	□

BM1_09. How often do you have difficulty remembering to take all your blood pressure medicine?
□ Never/rarely
□ Once in a while
□ Sometimes
□ Usually
□ All the time

Deleted: *MBHT*
Deleted: *December 21, 2015*

More About You

Below are some modifiable factors that likely influence blood pressure. These may not all apply to you, as you may already have excellent levels of these factors.

Physical activity:

The United States Office of Disease Prevention and Health Promotion 2008 Physical Activity Guidelines for Americans states that "Most health benefits occur with at least 150 minutes (2 hours and 30 minutes) a week of moderate intensity physical activity, such as brisk walking. Additional benefits occur with more physical activity. Both aerobic (endurance) and muscle-strengthening (resistance) physical activity are beneficial."

RC1_01. How motivated are you to make changes to your physical activity, using a scale of one to ten, where one = definitely not ready to change, and 10 = definitely ready to change?

Little intention of changing			Mixed feelings towards taking action				Motivated to take action		
1	2	3	4	5	6	7	8	9	10
☐	☐	☐	☐	☐	☐	☐	☐	☐	☐

RC1_02. On a scale of 1-10, with 10 being 100% confident, how confident are you that you can make changes in your physical activity?

Not confident			Moderately confident				Confident		
1	2	3	4	5	6	7	8	9	10
☐	☐	☐	☐	☐	☐	☐	☐	☐	☐

Diet:

The Dietary Approaches to Stop Hypertension (DASH) diet eating plan is a diet rich in fruits, vegetables, low fat or nonfat dairy. It also includes mostly whole grains; lean meats, fish and poultry; nuts and beans. It is high fiber and low to moderate in fat. It is a plan that follows US guidelines for sodium content, along with vitamins and minerals. It can be considered to be an Americanized version of the Mediterranean diet.

RC1_03. How motivated are you to make changes to your diet to be consistent with the DASH diet, using a scale of one to ten, where one = definitely not ready to change, and 10 = definitely ready to change?

Little intention of changing			Mixed feelings towards taking action				Motivated to take action		
1	2	3	4	5	6	7	8	9	10
☐	☐	☐	☐	☐	☐	☐	☐	☐	☐

RC1_04. On a scale of 1-10, with 10 being 100% confident, how confident are you that you can make changes in your diet to be more consistent with the DASH diet?

Not confident			Moderately confident				Confident		
1	2	3	4	5	6	7	8	9	10
☐	☐	☐	☐	☐	☐	☐	☐	☐	☐

Deleted: *MBHT*

Deleted: *December 21, 2015*

Salt Intake:

The 2010 Dietary Guidelines for Americans recommend that everyone age 2 years and up should consume less than 2,300 milligrams (mg) of sodium each day. Some groups of people should further limit sodium intake to 1,500 mg per day, including:

- Adults age 51 years or older.
- All African Americans.
- Anyone who has high blood pressure, diabetes, or chronic kidney disease.

RC1_05. How motivated are you to make changes to your salt intake, using a scale of one to ten, where one = definitely not ready to change, and 10 = definitely ready to change?

Little intention of changing			Mixed feelings towards taking action				Motivated to take action		
1	2	3	4	5	6	7	8	9	10
☐	☐	☐	☐	☐	☐	☐	☐	☐	☐

RC1_06. On a scale of 1-10, with 10 being 100% confident, how confident are you that you can make changes in your salt intake?

Not confident			Moderately confident				Confident		
1	2	3	4	5	6	7	8	9	10
☐	☐	☐	☐	☐	☐	☐	☐	☐	☐

Overweight/Obesity:

Extensive scientific evidence shows that being overweight or obese increases risk of having high blood pressure.

RC1_07. How motivated are you to make changes to your body weight, using a scale of one to ten, where one = definitely not ready to change, and 10 = definitely ready to change?

Little intention of changing			Mixed feelings towards taking action				Motivated to take action		
1	2	3	4	5	6	7	8	9	10
☐	☐	☐	☐	☐	☐	☐	☐	☐	☐

RC1_08. On a scale of 1-10, with 10 being 100% confident, how confident are you that you can make changes in your body weight?

Not confident			Moderately confident				Confident		
1	2	3	4	5	6	7	8	9	10
☐	☐	☐	☐	☐	☐	☐	☐	☐	☐

Deleted: *MBHT*

Deleted: *December 21, 2015*

Stress, and Stress Response:

Several studies showed that stress, and being slower at emotionally recovering from stressful events, increase risk of hypertension.

RC1_09. How motivated are you to make changes to the amount of stress in your life, or your response to that stress, using a scale of one to ten, where one = definitely not ready to change, and 10 = definitely ready to change?

Little intention of changing			Mixed feelings towards taking action				Motivated to take action		
1	2	3	4	5	6	7	8	9	10
☐	☐	☐	☐	☐	☐	☐	☐	☐	☐

RC1_10. On a scale of 1-10, with 10 being 100% confident, how confident are you that you can make changes in the amount of stress in your life, or your response to that stress?

Not confident			Moderately confident				Confident		
1	2	3	4	5	6	7	8	9	10
☐	☐	☐	☐	☐	☐	☐	☐	☐	☐

Alcohol Consumption:

Heavy and regular use of alcohol can increase blood pressure substantially. The American Heart Association recommends limiting alcohol consumption to no more than two drinks per day for men and one drink per day for women.

RC1_11. How motivated are you to make changes to the amount of alcohol you consume, using a scale of one to ten, where one = definitely not ready to change, and 10 = definitely ready to change?

Little intention of changing			Mixed feelings towards taking action				Motivated to take action		
1	2	3	4	5	6	7	8	9	10
☐	☐	☐	☐	☐	☐	☐	☐	☐	☐

RC1_12. On a scale of 1-10, with 10 being 100% confident, how confident are you that you can make changes in the amount of alcohol you consume?

Not confident			Moderately confident				Confident		
1	2	3	4	5	6	7	8	9	10
☐	☐	☐	☐	☐	☐	☐	☐	☐	☐

Deleted: *MBHT*

Deleted: *December 21, 2015*

Blood Pressure (Antihypertensive) Medication Use:

Blood pressure medication has been shown in many studies to be very effective at lowering blood pressure.

RC1_13. How motivated are you to make changes to your blood pressure medication use, using a scale of one to ten, where one = definitely not ready to change, and 10 = definitely ready to change?

Little intention of changing			Mixed feelings towards taking action				Motivated to take action		
1	2	3	4	5	6	7	8	9	10
☐	☐	☐	☐	☐	☐	☐	☐	☐	☐

RC1_14. On a scale of 1-10, with 10 being 100% confident, how confident are you that you can make changes your blood pressure medication use?

Not confident			Moderately confident				Confident		
1	2	3	4	5	6	7	8	9	10
☐	☐	☐	☐	☐	☐	☐	☐	☐	☐

Deleted: MBHT

Deleted: December 21, 2015

Family History of Hypertension

FH1_01. Did your biological mother ever have hypertension?

□ No
□ Yes
□ I do not know
□ I prefer not to answer

FH1_02. Did your biological father ever have hypertension?

□ No
□ Yes
□ I do not know
□ I prefer not to answer

FH1_03. How many full brothers and sisters do you have ***(Please include any brothers or sisters who may have died, but do not include half or step brothers and sisters).***

□ I do not have any brothers or sisters → S***kip to the page***
_____ brothers, _______ sisters
□ I do not know
□ I prefer not to answer

FH1_04. Of these brothers and sisters, how many have ever had hypertension?

_________ ***(if none, write 0)***
□ I do not know
□ I prefer not to answer

Deleted: MBHT
Deleted: December 21, 2015

Your Sleep

The following question relates to your usual sleep habits during the past month only. Your answer should indicate the most accurate reply for the majority of days and nights in the past month.

SL1_04. **During the past month**, how many hours of actual sleep did you get on average at night? (This may be different than the number of hours you spent in bed.)

AVERAGE HOURS OF SL1_EEP PER NIGHT ___________

☐ I do not know
☐ I prefer not to answer

END SCRIPT

Thank you for completing this survey!

Please note that these responses will not be seen immediately. Resources are shown below if you feel that you would like to talk with someone immediately for assistance.

National Suicide Prevention Lifeline: 1-800-273-8255
National Sexual Assault Hotline: 1-800-656-4673

Other options are to:

- Call your doctor's office
- Call 911 for emergency services
- Go to the nearest hospital emergency room.

Deleted: *MBHT*
Deleted: *December 21, 2015*

Appendix 13:
Measurement of Formal and Informal Mindfulness Practice Post-intervention - *NEW*

MB-BP Study: Measurement of Mindfulness Practice post-Intervention

(To be administered at each Follow-up assessment)

MP1_01 **10wk follow up:** Since the date of the last mindfulness class, have you practiced mindfulness, either formally or informally, in any way?

6mo / 1yr follow ups: Think about the last six months. During that time, have you practiced mindfulness, either formally or informally, in any way?

No .... 0 **(*Skip to MP1_04*)**
Yes 1

On average, how minutes PER WEEK do you engage in the following types of mindfulness activities?	Average # Minutes per week	Less than weekly	*Don't Know*	*Prefer not to answer*
MP1_02a Body Scan	____	666	777	888
MP1_02b Yoga	____	666	777	888
MP1_02c Awareness of breath meditation	____	666	777	888
MP1_02d Sitting Meditation	____	666	777	888
MP1_02e Walking Meditation	____	666	777	888
MP1_02f Loving-kindness Meditation	____	666	777	888
MP1_02g Mountain Meditation	____	666	777	888
MP1_02h Visual Meditation	____	666	777	888
MP1_02i Eating Meditation	____	666	777	888
MP1_02j Meditation moving through regions, such as breath, physical sensations, sound, thoughts, and open awareness	____	666	777	888
MP1_02k Goal-related activity (e.g., physical activity, diet change, etc.). *Please describe*______________________________..................	____	666	777	888
MP1_02l Other mindful activities:<br>Other 1 *describe:* ________________ ....<br>Other 2 *describe:* ________________ ....<br>Other 3 *describe:* ________________	____	666	777	888

MP1_03.a-l **If > 0 min per day, skip. All others, ask:** Which of the following statements, BEST describes your current attitude towards [*insert mindfulness activity from table above*]?

I do not plan on practicing this activity..1
I see value in this activity but do not practice it regularly 2
Something else: *Describe*_____________________ 3
Don't Know 7
Prefer not to answer...8

MP1_04 **If NO Mindfulness Practice, Ask:** Which of the following statements, BEST describes your current attitude towards mindfulness?

I do not plan on practicing mindfulness 1
I see value in mindfulness but do not practice it regularly...........2
Other (*please describe* ____________________________)......3
Don't Know 7
Prefer not to answer...8

MP1_05 Which of the following, if any, have you participated in [since completing the course / the last six months]?

None, I have not practiced mindfulness 00

Check ALL that apply

MB-BP Study Booster Sessions............................... [ ]
MB-BP All day retreats.. [ ]
Meditation group *not* related the MB-BP Study .... [ ]
Mindful yoga group *not* related the MB-BP Study .... [ ]
Other 1 *describe:* ________________ .... [ ]
Other 2 *describe:* ________________ .... [ ]
Other 3 *describe:* ________________ .... [ ]
Don't Know.. 77
Prefer not to answer... 88

MP1_06 We are interested in the ways in which mindfulness practice may or may not impact your life. Please describe below your relationship to mindfulness since completing your course.

Don't Know 7
Prefer not to answer...8

Brown University
Research Protections Office
Institutional Review Board
Modification Request

Date of Request: 8/9/16 **Investigator's Name and Title:** Eric Loucks, PhD, Assistant Professor

Study Title: Mindfulness-based Hypertension Therapy Pilot Study (#1412001171)

Original Type of Review: ☐ Exempt ☐ Expedited ☒ Full Board

1) **Provide a brief lay summary of the overall project. Include enough detail to allow the IRB to evaluate the requested change(s) within the context of the overall project.** (Attach summary to this form)

See attached summary

2) **Provide a detailed description of the changes being requested** (Use additional pages, if necessary):

See attached summary

3.) **State the reason (justification) for the requested modification.** (Use additional pages, if necessary):

See attached summary

4.) **What is your assessment of how the changes will affect the overall risk/benefit ratio of the study and the willingness of individuals to participate?**

See attached summary

5.) **Does the requested modification require new documents or changes to the approved consent form or other documents?**

☐ Consent/assent documents (attach revised version with changes highlighted)

☒ New/revised instruments (attach - if revised, highlight changes)

☐ New/revised advertising materials (attach - if revised, highlight changes)

Do you have a conflict of interest on this project according to Brown's policy? ☐**YES** ☒**NO**

If YES, has this conflict been previously disclosed to the IRB? ☐**YES** ☐**NO**

PI signature: [signature] Date: 8/7/16

1. **Provide a brief lay summary of the overall project. Include enough detail to allow the IRB to evaluate the requested change(s) within the context of the overall project.**

The World Health Organization reported that suboptimal blood pressure (BP) is responsible for more than half of cardiovascular disease mortality world-wide. Furthermore, greater than half of those with hypertension have uncontrolled BP. *A 2009 Institute of Medicine report recommended prioritizing research to "Compare the effectiveness of mindfulness-based interventions (e.g. yoga, meditation, deep breathing training) and usual care in treating… cardiovascular risk factors."* Evidence-based mindfulness interventions, including Mindfulness-Based Stress Reduction, may have some effects on blood pressure, where a recent meta-analysis and systematic review of 4 randomized controlled trials demonstrated significant effects, but evidence of heterogeneity in effect sizes. The methodologically highest quality studies had the smallest effect sizes (range 0-5 mmHg). Mindfulness-Based Stress Reduction (MBSR) has been customized to a number of disease processes, such as Mindfulness-Based Cognitive Therapy for patients with recurrent depression, and Mindfulness-Based Relapse Prevention for patients with substance use addictions. Effect sizes have been increased by customizing mindfulness interventions to diseases of interest. The same may be true for hypertension, *however mindfulness interventions customized for prehypertensive/hypertensive patients have never been investigated. Until methodologically rigorous studies to evaluate customized interventions for hypertension are performed, we will not know if the observed preliminary effects of general mindfulness interventions on blood pressure reduction could be much more effective with a tailored approach.* Consequently, we propose to conduct a stage I behavioral therapy intervention study to evaluate whether MBSR customized to prehypertensive and hypertensive patients has the potential to provide clinically relevant reductions in BP. Consequently the specific aims are:

Stage 1a: Therapy Development/Manual Writing

1. To outline and evaluate key novel elements of mindfulness-based hypertension therapy (MBHT), customized from the evidence-based MBSR. *We hypothesize that the most important novel element will be generation of mindfulness skills specifically applied to hypertension risk factors such as diet, physical activity, obesity, alcohol consumption and antihypertensive medication adherence.* This aim will be achieved using (1) focus groups of participants undergoing the MBHT behavioral intervention, (2) discussion with experts (including cardiologists, epidemiologists, mindfulness experts, mindfulness intervention instructors) prior to, and following pilot testing of MBHT in participants, and (3) clinical judgment of the investigators performing the intervention.
2. To determine effectiveness of MBHT on primary outcomes (systolic blood pressure, retention rates, recruitment rates, and adverse effects) and secondary outcomes (hypertension risk factors such as diet, physical activity, obesity, and antihypertensive medication adherence) in hypertension subgroups, specifically participants with (1) prehypertension, (2) controlled hypertension, and (3) uncontrolled hypertension. Initial decisions about the targeted sample based on hypertension status will be made.
3. To develop an MBHT therapist manual and training program, including procedures for training, supervising, and evaluating therapists. Furthermore, acceptable therapist characteristics will be developed. The manual and training program will include themes such as specification of unique and common elements of MBHT *vs.* other interventions, description of interventions excluded from MBHT, and specification of key treatment parameters such as frequency and duration of treatment, session length, topics addressed, sequence of sessions, as well as therapist adherence and competency

measures. The MBHT training will consist of a therapist manual, a formal didactic training seminar, and at least one closely supervised training session.

Stage Ib: Pilot Trial

4. To determine whether a mindfulness-based hypertension therapy (MBHT) intervention, customized from the evidence-based MBSR, has promise to be an effective behavioral therapy for participants with hypertension and/or prehypertension. We will perform a randomized controlled pilot trial for MBHT *vs.* enhanced usual care control. *We hypothesize that MBHT will have adequate recruitment rates (≥10% of prehypertensive/hypertensive participants invited from physicians' offices), fairly low drop out rates (<15%), and medium effect sizes (e.g. 5-10 mmHg systolic BP) for reduction in blood pressure.*

These findings will provide publishable pilot data that will inform future randomized clinical trials that evaluate effects of MBHT on long-term changes in blood pressure *vs.* usual care and active control groups. *If proven effective, MBHT could be offered as a complementary program in the prehypertensive/hypertensive patient population that contributes to over half of the cardiovascular disease mortality world-wide.*

2. Provide a detailed description of the changes being requested (Use additional pages, if necessary):

We are requesting the following changes…

- **Addition of two new questions to the Home Baseline Assessment –** We would like to add two new questions about hypertension (BP1_1 and BP1_2) to the home baseline assessment (see attached pdf with track changes). Please note that these questions would be administered during the Home Baseline assessment only. However, for participants who have already completed the baseline questionnaire, we would capture this information during their next follow up assessment.

- **Permission to provide financial compensation to participants who withdrew from the study intervention but are still willing to complete follow up assessments** – since the start of our study, we have had two participants who have decided to withdraw from the study after the start of the intervention. We would be interested in re-contacting these and future individuals who withdraw to invite them to participate in the follow up assessments and would like to be able to provide them with $50 per follow up as an expression of gratitude for their time and effort.

3. State the reason (justification) for the requested amendment:

Please see justifications in Section 2 above.

4. What is your assessment of how the changes will affect the overall risk/benefit ratio of the study and the willingness of individuals to participate?

We do not anticipate any major changes to the risk/benefit ratio. Both of the new baseline questions are yes/no and take well under a minute to complete. Following up with participants who withdrew from the intervention about the possibility of completing the follow ups does not cause any significant additional risks. Study staff will be trained to remind participants that their participation is voluntary. Some individuals may have withdrawn from the study because of reasons related to their ability to complete the 9 week course, but may actually still be interested in participating in the research questionnaires. This would allow them that opportunity.

5. Does the requested amendment require new documents or changes to the approved consent form or other documents?

Yes. Changes to the Home Baseline Assessment (two new questions). See attached document with track changes – only p.43 was affected and is being attached.

References

[1] Morisky DE, Green LW, Levine DM. Concurrent and predictive validity of a self-reported measure of medication adherence. Med Care. 1986;24 (1):67-74.

HOME BASELINE ASSESSMENT

QUESTIONNAIRES ANSWERED BY PARTICIPANTS

AT BASELINE (VIA ONLINE OR PAPER FORM)

Blood Pressure and Blood Pressure Medication Use

The following questions are about blood pressure.

BP1_1. Does your blood pressure tend to be HIGHER when you have it measured in a clinical setting, such as a doctor's office? This is sometimes called "white coat hypertension."

□ No
□ Yes → **SKIP TO BM1_01**
□ I do not know
□ I prefer not to answer

BP1_2. Does your blood pressure tend to be LOWER when you have it measured in a clinical setting, such as a doctor's office? This is sometimes called "masked hypertension."

□ No
□ Yes
□ I do not know
□ I prefer not to answer

BM1_01. Do you currently take medication for your blood pressure?
□ Yes
□ No (if responding "no", please skip to the next page).

	Yes	No
BM1_02. Do you sometimes forget to take your blood pressure pills?	□	□
BM1_03. People sometimes miss taking their medications for reasons other than forgetting. Thinking over the past two weeks, were there any days when you did not take your blood pressure medicine?	□	□
BM1_04. Have you ever cut back or stopped taking your blood pressure medicine without telling your doctor because you felt worse when you took it?	□	□
BM1_05. When you travel or leave home, do you sometimes forget to bring along your blood pressure medicine?	□	□
BM1_06. Did you take all your blood pressure medicine yesterday?	□	□
BM1_07. When you feel like your symptoms are under control, do you sometimes stop taking your blood pressure medicine?	□	□
BM1_08. Taking blood pressure medicine every day is a real inconvenience for some people. Do you ever feel hassled about sticking to your treatment plan?	□	□

BM1_09. How often do you have difficulty remembering to take all your blood pressure medicine?
□ Never/rarely
□ Once in a while
□ Sometimes
□ Usually
□ All the time

Brown University
Research Protections Office
Institutional Review Board
Modification Request

Date of Request: 11/6/17 **Investigator's Name and Title:** Eric Loucks, PhD, Assistant Professor

Study Title: Mindfulness-based Blood Pressure Reduction (MB-BP) Study (#1412001171)

Original Type of Review: ☐ Exempt ☐ Expedited ☒ Full Board

1) **Provide a brief lay summary of the overall project. Include enough detail to allow the IRB to evaluate the requested change(s) within the context of the overall project.** (Attach summary to this form)

   See attached summary

2) **Provide a detailed description of the changes being requested** (Use additional pages, if necessary):

   See attached summary

3.) **State the reason (justification) for the requested modification.** (Use additional pages, if necessary):

   See attached summary

4.) **What is your assessment of how the changes will affect the overall risk/benefit ratio of the study and the willingness of individuals to participate?**

   See attached summary

5.) **Does the requested modification require new documents or changes to the approved consent form or other documents?**

   X Consent/assent documents (attach revised version with changes highlighted)

   ☒ New/revised instruments (attach - if revised, highlight changes)

   ☐ New/revised advertising materials (attach - if revised, highlight changes)

Do you have a conflict of interest on this project according to Brown's policy? ☐**YES** ☒**NO**

If YES, has this conflict been previously disclosed to the IRB? ☐**YES** ☐**NO**

PI signature: [signature] Date: 11/6/17

1. **Provide a brief lay summary of the overall project. Include enough detail to allow the IRB to evaluate the requested change(s) within the context of the overall project.**

Original lay summary providing broad overview of the project:

The World Health Organization reported that suboptimal blood pressure (BP) is responsible for more than half of cardiovascular disease mortality world-wide. Furthermore, greater than half of those with hypertension have uncontrolled BP. *A 2009 Institute of Medicine report recommended prioritizing research to "Compare the effectiveness of mindfulness-based interventions (e.g. yoga, meditation, deep breathing training) and usual care in treating… cardiovascular risk factors."* Evidence-based mindfulness interventions, including Mindfulness-Based Stress Reduction, may have some effects on blood pressure, where a recent meta-analysis and systematic review of 4 randomized controlled trials demonstrated significant effects, but evidence of heterogeneity in effect sizes. The methodologically highest quality studies had the smallest effect sizes (range 0-5 mmHg). Mindfulness-Based Stress Reduction (MBSR) has been customized to a number of disease processes, such as Mindfulness-Based Cognitive Therapy for patients with recurrent depression, and Mindfulness-Based Relapse Prevention for patients with substance use addictions. Effect sizes have been increased by customizing mindfulness interventions to diseases of interest. The same may be true for hypertension, *however mindfulness interventions customized for prehypertensive/hypertensive patients have never been investigated. Until methodologically rigorous studies to evaluate customized interventions for hypertension are performed, we will not know if the observed preliminary effects of general mindfulness interventions on blood pressure reduction could be much more effective with a tailored approach.* Consequently, we propose to conduct a stage I behavioral therapy intervention study to evaluate whether MBSR customized to prehypertensive and hypertensive patients has the potential to provide clinically relevant reductions in BP. Consequently the specific aims are:

Stage 1a: Therapy Development/Manual Writing

1. To outline and evaluate key novel elements of mindfulness-based hypertension therapy (MBHT), customized from the evidence-based MBSR. *We hypothesize that the most important novel element will be generation of mindfulness skills specifically applied to hypertension risk factors such as diet, physical activity, obesity, alcohol consumption and antihypertensive medication adherence.* This aim will be achieved using (1) focus groups of participants undergoing the MBHT behavioral intervention, (2) discussion with experts (including cardiologists, epidemiologists, mindfulness experts, mindfulness intervention instructors) prior to, and following pilot testing of MBHT in participants, and (3) clinical judgment of the investigators performing the intervention.
2. To determine effectiveness of MBHT on primary outcomes (systolic blood pressure, retention rates, recruitment rates, and adverse effects) and secondary outcomes (hypertension risk factors such as diet, physical activity, obesity, and antihypertensive medication adherence) in hypertension subgroups, specifically participants with (1) prehypertension, (2) controlled hypertension, and (3) uncontrolled hypertension. Initial decisions about the targeted sample based on hypertension status will be made.
3. To develop an MBHT therapist manual and training program, including procedures for training, supervising, and evaluating therapists. Furthermore, acceptable therapist characteristics will be developed. The manual and training program will include themes such as specification of unique and common elements of MBHT *vs.* other interventions, description of interventions excluded from MBHT, and specification of key treatment

parameters such as frequency and duration of treatment, session length, topics addressed, sequence of sessions, as well as therapist adherence and competency measures. The MBHT training will consist of a therapist manual, a formal didactic training seminar, and at least one closely supervised training session.

Stage Ib: Pilot Trial

4. To determine whether a mindfulness-based hypertension therapy (MBHT) intervention, customized from the evidence-based MBSR, has promise to be an effective behavioral therapy for participants with hypertension and/or prehypertension. We will perform a randomized controlled pilot trial for MBHT *vs.* enhanced usual care control. *We hypothesize that MBHT will have adequate recruitment rates (≥10% of prehypertensive/hypertensive participants invited from physicians' offices), fairly low drop out rates (<15%), and medium effect sizes (e.g. 5-10 mmHg systolic BP) for reduction in blood pressure.*

These findings will provide publishable pilot data that will inform future randomized clinical trials that evaluate effects of MBHT on long-term changes in blood pressure *vs.* usual care and active control groups. *If proven effective, MBHT could be offered as a complementary program in the prehypertensive/hypertensive patient population that contributes to over half of the cardiovascular disease mortality world-wide.*

Project update as of November 2017:

The Mindfulness-Based Blood Pressure Reduction (MB-BP) Study, formerly known as Mindfulness-Based Hypertension Therapy (MBHT), is currently in Year 2 of a five year NIH UH2 grant.

Data collection for Stage 1a of the project has ended and analysis is on-going. In total we ran three separate Mindfulness-Based Blood Pressure reduction (MB-BP) intervention courses, with a total sample size of 43 eligible participants who both enrolled and completed the 9-week intervention. We had eight eligible participants enroll in the study who later decided to not complete the course.

In June 2017 we began enrollment for the next phase (referenced as Stage 1b above, but now called 'Stage 2a') of the study, which is to conduct a Randomized Controlled Trial (RCT) with *enhanced usual care*. This phase will continue for the next 12-18 months, at which point we will transition to the third and final phase, an RCT with an *active* control (i.e., Mindfulness-Based Stress Reduction - MBSR) as well as ambulatory blood pressure monitoring.

2. Provide a detailed description of the changes being requested (Use additional pages, if necessary):

This amendment covers the following four requests:

(1) Qualitative Phone Interviews - First, we are requesting permission to modify the already approved focus group discussion questions into a one-on-one qualitative phone interview (see enclosed "Semi-structured qualitative phone interview"). Our intent is to then randomly select and re-contact Stage 1 participants, who did not participate in the focus group discussions, and invite them to take part in the one-on-one qualitative phone interviews instead. We would attempt to complete ten interviews. Participants who complete the phone interviews would be mailed $25 gift cards for their time and effort. ***Related enclosures: Appendices 1-3***

(2) 2-year Follow Ups – Second, we would like to re-contact participants who completed the course and invite them to come back into the office for a quick two year in-person assessment. This optional assessment is estimated to take 20-30 minutes to complete and would be a small sub-set of what is already approved by the IRB (i.e., blood pressure, weight, hypertension medication questions, and mindfulness practice questions). Participants would be given $50 USD for this additional component. For participants who have already been consented, we would have them sign the attached addendum to the consent form. For newly enrolled participants we would ask them to sign the attached revised consent form. It is already part of the approved study protocol and screening process to ask participants if we can keep their information on file to contact them about possible future studies or opportunities to participate in research. We would only re-contact participants who gave us permission to do so. ***Related enclosures: Appendices 3-4***

(3) Participant Compensation – Third, we are requesting permission to alter the order in which participants are compensated. As it is currently written, participants are given $25, $25, and $50 USD at their 10 week, 6 month and 12 month follow ups respectively. We are seeking to reverse that order and instead provide participants $50, $25, and $25 USD at their three follow ups. The total amount provided as compensation for participation would remain the same ($100 USD). Participants already enrolled into the study would be compensated in the manner already outlined and approved. However, we are proposing that newly enrolled participants would be compensated under the new guidelines outlined in this amendment (if approved). See p.1 of the revised consent form for this change. ***Related enclosure(s): Appendix 3***

(4) Recruitment assistance for UMass fMRI sub-study – the MB-BP Study is one of several concurrent studies being funded under the NCCIH Science of Behavior Change UH2 project "Mindfulness Influences on Self-Regulation: Mental and Physical Health Implications." Our collaborators at the UMass medical school have recently received IRB approval from the UMass Medical School IRB to conduct a related clinical trial that involves fMRI imaging of MB-BP participants to evaluate several mechanistic self-regulation targets of the Mindfulness-based blood pressure therapy (MB-BP), their neural substrates, and their potential impact on autonomic system measures. Their proposed project (titled, Mindful Self-Regulation for blood pressure MRI study) involves recruiting, screening, enrolling, and scanning a sub-set of MB-BP participants (i.e., goal is to enroll and image 60 eligible MB-BP participants over the 12-18 months). Eligible participants who agree to take part in the fMRI study at UMass Medical School

will be invited to travel up to the UMass facility twice – once at baseline and once at the 10 week follow up period – to undergo two MRI sessions (~1.5 hours each). Participants will be compensated $150 USD for each neuroimaging session. Note: see attached UMass Medical School IRB submission for the full version of their study protocol, informed consent form, phone screener, and IRB approval letter.

In this amendment we are requesting permission to approach newly enrolled MB-BP participants about the UMass Medical School fMRI study and to see if they would be interested in being contacted by the UMass medical school research staff to complete a brief 15-minute phone screener to determine their eligibility for the related neuroimaging study. To do this, Brown University research staff would use the attached "UMass Medical School fMRI Study Recruitment Talking Points" document. This document outlines the main features of the neuroimaging study and what participant involvement would entail. However, it is important to note that both the screening and the informed consent procedures will be conducted by UMass Medical School research staff and not by Brown University. Our role will be to introduce the fMRI study to our MB-BP participants and document their consent for UMass Medical School staff to contact them by phone for screening. We will only provide UMass Medical School research staff with the contact information of MB-BP participants who agree to be contacted regarding the neuroimaging study. Contact information of MB-BP participants who do not wish to be contacted by UMass will not be passed along. ***Related enclosures: Appendices 5-6***

3. State the reason (justification) for the requested amendment:

(1) The data collected in the proposed qualitative phone interviews will complement the qualitative data previously collected in the focus group discussions. It will also hopefully help to address any potential selection bias due to the more engaged and enthusiastic participants opting to take part in the focus group discussions.

(2) Analysis of the one year follow up data has been very promising, showing sustained reductions in blood pressure and other important outcomes. There are very few research studies that explore sustained effects at 12 months or even 24 months. Recontacting participants to have them complete a 2 year follow up would offer novel research findings to the field of mindfulness and cardiovascular health.

(3) We are proposing a change in the compensation protocol for two reasons. First, we believe that providing participants the larger sum for the first follow up rather than the last follow up will help with retention rates overall. We have often struggled to get participants to come in for the first follow up, particularly with members of the control group, who did not get the intervention. However, we have not had as much difficulty getting participants to complete their final follow up. We find that once individuals have already invested time to complete the first two follow ups, they are often intrinsically motivated to complete the final follow up and therefore don't require as much external motivation to participate. The second justification for altering the payment structure is that the 12 month data are not as important to the overall study and analyses as the 10 week data are.

(4) The UMass Medical School Mindful Self-Regulation for blood pressure MRI study is a related concurrent study on the same UH2 grant that is funding MB-BP. Since it involves recruiting a sub-set of MB-BP participants it makes the most sense for the research staff at Brown University to be the individuals to recruit.

4. What is your assessment of how the changes will affect the overall risk/benefit ratio of the study and the willingness of individuals to participate?

We do not anticipate any shifts in the risk / benefit ratio of the study. Participation in all aspects of the study is optional and will be explicitly stated at the time of recruitment and informed consent. This includes when approaching participants about potential involvement in the one on one qualitative phone interviews, 2 year follows ups, and possible participation in the fMRI study.

5. Does the requested amendment require new documents or changes to the approved consent form or other documents?

See enclosed attachments related to this amendment:

Appendix 1 - Protocol for semi-structured qualitative phone interview scripts and talking points.

Appendix 2 – Addendum to consent form to be signed by already enrolled participants

Appendix 3 – Revised Informed Consent (v.2.2) – with track changes

Appendix 4 – Two year in-person follow up assessment

Appendix 5 – UMass Medical School fMRI Study Recruitment Talking Points for research staff at Brown University to utilize

Appendix 6 – Umass Medical School IRB submission for fMRI sub-study

Appendix 1 -
Semi-structured qualitative phone interviews

Created from the already approved focus group discussion protocol and modified to be relevant for a one-on-one qualitative phone interview.

MB-BP qualitative phone interview protocol

Semi-Structured Script and Talking Points
"Hello, my name is __________ and I am calling from Brown University regarding the Mindfulness Based Blood Pressure Reduction Study that you took part in just over a year ago. As we begin to evaluate the intervention we would like to get a better understanding of what worked and didn't work by conducting personalized one-on-one phone interviews with yourself and other graduates of the program.<br><br>The interview would be done by phone, would take 20 minutes to a half an hour to complete, and could be scheduled at a time that is convenient for you. You would also receive a $25 gift card for your time. Would you be willing to offer feedback on your experiences with the mindfulness program?"<br><br>TALKING POINTS –<br><br>● **Confidentiality**: As a reminder, everything you say will remain confidential. No one outside of our project will be able to see your answers and your name and other identifying information will not be used in any reports or publications.<br><br>● Why and How? "We are hoping to get your feedback about the mindfulness course and use it to make the program more effective. Please feel free to share your point of view and know that we welcome both positive and negative feedback. There are no right or wrong answers just your perspective about the program."<br><br>**Important points:**<br>○ We are trying to improve the intervention<br>○ Discussing your opinions on the different activities<br>○ Please share your point of view<br>○ No wrong answers<br>○ We equally welcome positive and negative feedback<br><br>● **Suggestions:** "We would like to record this call with your permission so that it can be transcribed later by members of our team. If you would prefer not to be recorded, please let me know. Also, please understand that any report we write regarding your feedback and what we hear from you today will not be identified with you in any way and remains anonymous."<br><br>Suggestions to help us have a good discussion<br>■ Speak up<br>■ Audio recording if that is ok<br>■ Any report that we write about what we hear today will not be associated with your identity<br><br>● **What to expect:** "So before we begin I just want to let you know that we will be asking you a series of open ended questions. My role is listen and ask questions to better understand your experiences. So I might ask you for clarification or more detail about a comment but your thoughts are the important part of this discussion. However, in the interest of getting to all the questions I may need to move us along pleas know that this is only due to time considerations."<br><br>○ My role is to listen ask questions that help to understand your experience<br>○ In the interest of time, I may move to the next question to get your thoughts regarding the whole program<br><br>Any questions before we begin?
Discuss Class Overview: Each sessions central activity<br><br>"First I am going to read through a list of the major class activities you participated in. While I read through the activities I would like you to think about which was the most memorable for you and why."<br><br>"So, hearing the course activities, which is most memorable for you? Why?"
"Next I have a series of 4 questions for you about your experiences in the program."<br><br>1. What was most helpful about this course, and why?
2. After going through this mindfulness intervention, what is your understanding of how it works to improve your blood pressure?
3. We want to make this intervention better. You have been through it once. How do you think we can make it better?
4. Every instructor can improve. How can this instructor improve?
Read the survey: record responses.<br>• Each weekly session was 2.5 hours long. Do you think the session should be 2 hours, 2.5 or 3 and why?<br>• The retreat day was scheduled to be 7.5 hours long. How long do you think the day should be (5,6,7, or 8) and why?<br>• Please provide us with an additional feedback we may not have asked about at this time.

Appendix 2 -

Addendum to Informed Consent

To be signed by participants who have already enrolled in the study and who opt to complete the optional two year follow up assessment

Brown University
Consent to Participate in a Research Study
Addendum to provide additional information to subject after original consent and to seek permission to complete a two year follow up assessment

---

IRB Study #__1412001171__________
Consent Form Version Date: _12/6/2017__
Title of Study: Mindfulness-Based Blood Pressure Reduction (MB-BP) Study
Principal Investigator: Eric B. Loucks, PhD__

Study Contact telephone number: 401-400-4768
Study Contact email: mindfulness@brown.edu

The following information should be read as an addition to the original Consent form that you read and signed at the beginning of the study. Unless specifically stated otherwise in the following paragraphs, all information contained in that original Consent Form is still true and remains in effect. Your participation continues to be voluntary. You may refuse to participate, or may withdraw your consent to participate at any time, and for any reason, without jeopardizing your future care at this institution or your relationship with your health care provider.

New or additional information
You are being asked to participate in an optional two year follow up assessment that will take place in-person at the Brown University Mindfulness & Cardiovascular Health Lab located at 121 South Main Street, Providence, RI 02912. The two year follow up assessment is estimated to take around 20 to 30 minutes to complete and will consist of a subset of measures that you previously completed as a participant in the MB-BP Study. Specifically, we will be taking your blood pressure and weight as well as asking you a series of questions about hypertension medications and mindfulness practices.

Subject's Agreement:
I have read the information provided above. I have asked all the questions I have at this time. I voluntarily agree to continue to participate in this research study.

_______________________________________ ________________
Signature of Research Subject Date

____
Printed Name of Research Subject

_______________________________________ ________________
Signature of Research Team Member Obtaining Consent Date

____
Printed Name of Research Team Member Obtaining Consent

Appendix 3 -
Revised Informed Consent
(v.2.2 – with track changes)

For newly enrolled participants to complete at time of screening. The revisions in this document cover the request to follow up at two years as well as the change in payment structure.

The Mindfulness-Based Blood Pressure Reduction (MB-BP) Study

Agreement to Participate in a Research Study

Investigation of the Effects of Mindfulness on Blood Pressure and Well-Being

You are being asked to take part in a Brown University research study about the effects of mindfulness and hypertension education on blood pressure and risk factors for hypertension. This form will explain the purpose of the study, how the study will be carried out and what you will be expected to do. It will also explain the possible risks and possible benefits of being in the study. If any part of the following description is not clear to you, you are encouraged to contact the researcher to answer any questions before you decide whether to take part in the study. If you decide to participate, please fill out and sign the last page of this form.

1a. Nature and Purpose of the Study

The purpose of the study is to investigate the impact of mindfulness practices and health education on blood pressure. You have been selected for this study because you expressed interest in the project and because you met entrance criteria for having prehypertension or hypertension, or another cardiovascular risk factor that could be influenced by this program. Eligibility for the study is still being assessed. Therefore, it is possible you may not be eligible for the study even after signing this consent form. Your participation in this study is voluntary and can be withdrawn at any point in the project.

In order to assess the effects of the customized mindfulness intervention, you will be asked to complete some questionnaires and laboratory assessments before and after the intervention. Specifically, assessments will be completed at: baseline, 10 weeks, 6 months and 1 year. To express our gratitude for participation, you will be given $~~25~~50, $25, and $~~50~~ 25 (up to $100 USD total) at the 10 week, 6 month and 12 month follow ups respectively. As part of the study you will also be given a wireless blood pressure monitor (estimated value of $90) to use throughout the study. There is an optional 2 year follow up that you may be invited to complete, for which you will be given an additional $50 USD.

This is a Randomized Control Trial. Participants enrolled into the study will be randomly assigned to one of two groups: (1) the intervention group or (2) the wait-list control group. The wait-list control group will be given the opportunity to participate in the intervention after the six month follow up assessments are completed. Both the intervention and the control group will be asked to participate in the research assessments.

1b. Explanation of Procedures

If you agree to participate, you will be asked to consent to the following:

1) Participation in an interview in which you will be asked questions about past and present mental health, including depression and suicide (previously completed with your verbal consent).

2) Completion of an in-person screening assessment, during which your blood pressure, height, weight and other basic demographic and health data will be collected and assessed in order to determine eligibility for the study.

3) Completion of questionnaires administered in-person and online that ask about a wide range of topics, including your diet, physical activity, smoking, medication use, personality, emotions, attention and past experiences, including stressful or traumatic experiences. These questions will probe sensitive psychological areas, including physical, emotion and sexual abuse. These questionnaires may take up to 3 hours to complete. By completing the interviews and questionnaires, you are giving the researchers permission to use the information you have provided. You have the right not to answer any of the questions.

4) Directly assessed blood pressure, heart rate, height, weight, physical activity, and antihypertensive (blood pressure) medication use at baseline and after the mindfulness course. Physical activity will be assessed for a week at a time using small actigraphy monitors (i.e., Fitbits) that attach to your wrist. If you take antihypertensive medication, we will provide you with an electronic bottle cap that will automatically record when the pill bottle is opened during the study. This will help us measure how often the medication is used. We will also provide you with a wireless blood pressure monitor and will ask that you take your blood pressure at home systematically during each of the research assessment periods (i.e., baseline, 10 week, 6 month and 12 month).

5) You will be asked to perform some cognitive tasks. Some of these tasks may involve computer-based tests of attention or decision-making. Together these tests may take as long as 20 minutes.

6) During the in-person assessments you will also be given a battery of stress tests that are designed to induce a stress response so that we can monitor your cardiovascular response and recovery.

7) Attendance at an information session that is to be held within two weeks of the start of the intervention.

8) If randomized into the intervention group, you will participate in the mindfulness program, which consists of 9 weekly sessions of 2.5 hours each and will include one 7.5 hour weekend retreat. Daily at home practice assignments may take as long as one hour and consist of practicing mindfulness exercises with the aid of a guided meditations and completing worksheets related to stress, thoughts, and common reactions to various types of events. If you are randomized into the wait-list control group, you will be invited to take part in the mindfulness class after the completion of the 6 month follow up assessments.

9) Class sessions may be audio taped so we can analyze the quality of the treatment you receive. The recordings will be transcribed so that we may analyze the text. The recordings will be identified by study number, will only be heard by study staff and will be destroyed after transcription.

10) You may be asked to complete a few short questionnaires each week during the 9 week condition.

11) After 10 weeks from the start of the intervention, you will be asked to complete questionnaires and return to the laboratory to repeat the same procedures for a second day of testing. If in the intervention arm, you will also be invited to participate in a focus group to share any advice you may have on how to improve the intervention.

12) Six months and one year after the beginning of the study, you will be asked to return to the laboratory to repeat the same testing procedures.

~~12)~~13) In addition to the six month and one year follow up, you will also be re-contacted to participate in an optional, very brief two year follow up assessment. The two year follow up assessment is estimated to take around 20 to 30 minutes to complete and will consist of a subset of measures that you would have previously completed as a participant in the MB-BP Study. Specifically, we will be taking your blood pressure and weight as well as asking you a series of questions about hypertension medications and mindfulness practices. For this additional follow up you will be given $50 USD.

Table Summarizing Activities and Time Commitment for this Study.

**Activity**	**Estimated Time Commitment**
In-person screening assessment	0.5 hours
Baseline<br>In-person assessment<br>Online questionnaire<br>At home health monitoring (e.g., fitbit, BP, etc.)	 2.0 hours<br>1.0 hours<br>1.0 hours
Information session	1.0 hour
Intervention*<br>Mindfulness course<br>Home practice assigned during course<br><br>**Intervention group only; control group will be invited to take part in a class post 6 month follow up but it will not be required as part of the study.*	Nine 2.5 hour sessions<br>7.5 hour all day retreat<br>Up to 1hr daily home practice assignments<br>*Total course time: 30.0 hours*<br>*Max. practice time: 48 hours*
Focus group participation post intervention (*intervention group only*)	1.5 hours
Follow Ups – 10 week, 6 month, 12 month<br>In-person assessments<br>Online questionnaires<br>At home health monitoring (e.g., fitbit, BP, etc.)	Total follow up time:<br>6.0 hours<br>3.0 hours<br>3.0 hours
Follow Up – 2 year (in person only)	30 minutes
**TOTAL ESTIMATED TIME COMMITMENT**	~~17.5~~18.0 hours – CONTROL<br>97.~~0~~5 hours - INTERVENTION

Feedback:

At the end of the study, you will receive individual feedback about the changes that occurred since the first assessment. Specifically, you will receive an individualized handout listing % change (increase or decrease) on scales of attention, stress, mood, health behaviours, weight, and blood pressure across the study.

Uncontrolled Hypertension:
If during the in-person assessments it appears that you have uncontrolled hypertension (your average systolic blood pressure reading is 140 mmHg or greater and/or your average diastolic blood pressure is 90 mmHg or greater) AND you indicate to us that you are not currently being treated for hypertension, then we will be requesting your permission to contact your health care provider to notify him/her of the blood pressure results. If you do not have a health care provider and/or do not have health insurance, our staff will provide you with resources to help you search for one; although we cannot guarantee that we will be able to find you one nor that it will be free. It is your choice on whether or not you would like us to follow up with your health care provider. Your participation in the study is not contingent on this communication; however, it is our recommendation that all individuals with uncontrolled hypertension be under the care of a health care professional.

2. Discomforts and Risks

The risks to you in this study are small. The questionnaires used in the study are routine, standardized forms for epidemiologic research. Certain questions may be upsetting as they may probe sensitive psychological areas and inquire about upsetting or traumatic events, including physical, sexual or emotional abuse and/or current psychiatric symptoms. The cognitive tests and stress battery may also invoke a stress response that may be uncomfortable. All aspects of the study are voluntary; you have the right to skip anything during the study that makes you uncomfortable.

Meditation-based interventions may results in discomfort with attention to unpleasant thoughts, feelings or body sensations. Some individuals may experience an initial increase in undesirable feelings with increased attention to them.

It is possible that injuries could be sustained during the study either from the gentle mindful movements (i.e., yoga), or from physical activities that participants engage in as a way to reduce blood pressure. To help limit this, you will receive a handout showing the yoga poses that will be offered during the course that you can show your health care provider so that they can advise on which poses to do, and which to avoid. Modifications of poses will be available as needed. None of the poses (or the yoga as a whole) are mandatory to be done. You will also be encouraged to explore physical activities that promote strength and conditioning as a way to reduce blood pressure. You will be encouraged to not go beyond any physical limits of your body, and will be encouraged to ask your healthcare provider about advised physical activities and mindful movements if you have any physical limitations.

While physical and mental injury is always a possibility the potential for harm is limited. Note that a research injury is any physical or mental injury or illness caused by your participation in

the study. If you are injured by a medical treatment or procedure that you would have received even if you were not in the study, that is not a research injury. To help avoid research injury and potential added medical expenses, it is important to follow all study directions carefully. If you are covered by insurance and suffer a research injury, it is possible that some or all of the costs of treating your condition could appropriately be billed to your insurance company. If such costs are not covered your health insurance company, it is possible you would have to pay for these costs out of pocket. Brown University's policies do not cover payment for such things as lost wages, medical care expenses, or pain and suffering.

Precautions should be taken to avoid injuries. If you do become injured during the study, you should call your doctor immediately. You should also alert the study staff that you have been injured. Heart attack and sudden death related to heart problems have been known to occur in people while they are exercising. This is very rare, however. Estimates of sudden cardiac death range from 0 to 2 per 100,000 hours. However, the researchers cannot guarantee that no complications will happen to you.

3. Benefits

We cannot and do not guarantee or promise that you will receive any direct benefits from this study. However, participation in the study creates the potential benefit of a) identifying effective treatments for elevated blood pressure, b) gaining knowledge of the effects of mindfulness practices, c) receiving information about your psychological and physical functioning. As part of the study, you will receive a wireless blood pressure monitor that will be yours to keep. This monitor may provide additional opportunity to monitor your blood pressure at home, which may benefit your health by providing additional biofeedback.

4. Alternative Therapies

A number of different therapies, including antihypertensive medication, diet changes, physical activity, and reducing excessive alcohol consumption may also be beneficial for reducing blood pressure. Education about these therapies are integrated into this course, but other forms of these alternative therapies are also available in the community.

5. Confidentiality

Your responses for this study will be kept confidential. All data that we collect will be linked to a study ID# instead of your name. All questionnaires in this study will be filled out through an online survey or a paper version of the survey if you prefer. All of these questionnaires will be linked solely to your study ID#, so that your identity is protected and your answers are confidential. Although these measures have been taken to protect your personal information, complete confidentiality cannot be guaranteed when transmitting information over the internet.

While your confidentiality is protected to the extent of the law, there are limitations to confidentiality. If your questionnaire responses indicate that you pose a serious danger to yourself or to another person, then a collaborator (Dr. Ellen Flynn) who is a licensed psychiatrist, may contact you to discuss your responses and possible referral to a treatment provider. Questionnaire items that may warrant follow-up include endorsements of statements about

hurting yourself, any high scores in depression, anxiety, or other clinically significant problems. You should also know that there are times when the law might require the release of your responses without your permission. For example, State law requires researchers to report abuse or neglect of children to the Department of Children, Youth and Families (DCYF). State law also requires researchers to report abuse or neglect of people age 60 and older to the Division of Elderly Affairs.

The findings of the study may be used for medical publication. Your name will not be used in any published reports about this study. Results will be reported in a summarized manner in such a way that you cannot be identified. All personally identifiable information will be "de-identified" and only a unique code number will be used. Study records will be identified with a unique code number and initials. All study records and specimens will be stored in a secure storage area.

Keeping study records: The Principal Investigator for this study will keep your research records indefinitely for research purposes.

Certificate of Confidentiality: This research is covered by a Certificate of Confidentiality from the National Institutes of Health. The researchers with this Certificate may not disclose or use information, documents, or biospecimens that may identify you in any federal, state, or local civil, criminal, administrative, legislative, or other action, suit, or proceeding, or be used as evidence, for example, if there is a court subpoena, unless you have consented for this use. Information, documents, or biospecimens protected by this Certificate cannot be disclosed to anyone else who is not connected with the research except, if there is a federal, state, or local law that requires disclosure (such as to report child abuse or communicable diseases but not for federal, state, or local civil, criminal, administrative, legislative, or other proceedings, see below); if you have consented to the disclosure, including for your medical treatment; or if it is used for other scientific research, as allowed by federal regulations protecting research subjects.

The Certificate cannot be used to refuse a request for information from personnel of the United States federal or state government agency sponsoring the project that is needed for auditing or program evaluation by the National Center for Complementary and Integrative Health, which is funding this project. You should understand that a Certificate of Confidentiality does not prevent you from voluntarily releasing information about yourself or your involvement in this research. If you want your research information released to an insurer, medical care provider, or any other person not connected with the research, you must provide consent to allow the researchers to release it. The Certificate of Confidentiality will not be used to prevent disclosure as required by federal, state, or local law of situations of child abuse and neglect, or harm to self or others.

6. Refusal/Withdrawal

Participation is voluntary, and you may decide to opt out or refuse any part of participation, including not answering certain questions. If you decide now to participate, you can change your mind later and quit the study. The decision to not participate or to withdraw from the study will not adversely affect current or future interactions with Brown University. The decision to not participate or to withdraw from the study will also not adversely affect your relationship with your physician.

If you decide not to participate, or if you quit the study, we will provide you with referrals for alternative treatments, if desired.

7. Contact Information

If you have questions about study procedures at any time you may contact the researchers: Dr. Eric B. Loucks, email: eric.loucks@brown.edu, telephone (401) 863-6283. If you would like more information about the rules for research studies, or the rights of people who take part in those studies, you may contact the Brown University Human Research Protection Program, telephone number 1-866-309-2095 or 401-863-3050.

A description of this clinical trial will be available on http://www.Clinical Trials.gov, as required by U.S. Law. This Web site will not include information that can identify you. At most, the Web site will include a summary of the results. You can search this Web site at any time.

CONSENT FORM:
Please sign and return.

Please read the following agreement and sign to consent to participating.

I HAVE READ THE ABOVE DESCRIPTION OF THIS STUDY. ALL OF MY QUESTIONS HAVE BEEN SATISFACTORILY ANSWERED, AND, I AGREE TO PARTICIPATE IN THIS RESEARCH STUDY.

____

PRINT NAME

__________________________________ __________________

Signature of participant **Date**

STUDY PARTICIPANT CONTACT INFORMATION

Name (print):____

Permanent Address:____

Email(s):____

Telephone:_________________________**(cell)** _________________________**(other)**

Appendix 4 -
Two year in-person follow up assessment

Estimated to take 20-30 minutes to complete and consists of a subset of already approved measures

MB-BP STUDY: 2 YEAR IN PERSON FOLLOW UP ASSESSMENT

PID. Participant ID # __________
BA01. Staff ID # __________
BA02. Today's date (MMDDYY): ___________

Blood Pressure:
BA03a. Blood pressure 1st reading, systolic blood pressure: _________ mmHg
BA03b. Blood pressure 1st reading, diastolic blood pressure: __________ mmHg
BA03c. Blood pressure 2nd reading, systolic blood pressure: _________ mmHg
BA03d. Blood pressure 2nd reading, diastolic blood pressure: __________ mmHg
BA03e. Blood pressure 3rd reading, systolic blood pressure: _________ mmHg
BA03f. Blood pressure 3rd reading, diastolic blood pressure: __________ mmHg

If the difference of the 2nd and 3rd systolic BP reading is 20 mmHg or greater OR the difference of the 2nd and 3rd diastolic reading is 10 mmHg or greater, then repeat the BP readings. Otherwise, skip to BA07.

BA06a. Repeated blood pressure 1st reading, systolic blood pressure: _________ mmHg
BA06b. Repeated blood pressure 1st reading, diastolic blood pressure: __________ mmHg
BA06c. Repeated blood pressure 2nd reading, systolic blood pressure: _________ mmHg
BA06d. Repeated blood pressure 2nd reading, diastolic blood pressure: __________ mmHg
BA06e. Repeated blood pressure 3rd reading, systolic blood pressure: _________ mmHg
BA06f. Repeated blood pressure 3rd reading, diastolic blood pressure: __________ mmHg

Blood Pressure Safety Protocol – The out-of-range blood pressure values are as follows: systolic blood pressure >200 mmHg or <90 mmHg; diastolic blood pressure >110 mmHg.

In absence of symptoms (chest pain, shortness of breath, dizziness, headache), for a SBP>200 or DBP>110 or both, we will strongly encourage participants to see their doctor right away or to go to urgent care. If there are symptoms, we will immediately call 911.

In absence of symptoms (chest pain, shortness of breath, dizziness, passing out), for a SBP<90, we will strongly encourage participants to see their doctor right away or to go to urgent care. If there are symptoms, we will immediately call 911.

Follow Safety Protocol for Uncontrolled Hypertension (140/90 mmHg or greater)

BA07. Blood pressure cuff size used: □ S □ Reg □ L □ XL
BA08. Arm that cuff was placed on: □ L □ R

BP monitor BP monitor used:
❍ Unit #1 - HEM-705CP (1)
❍ Unit #2 - HEM-705CP (2)
❍ Other (specify: (3) ____________________

We are now going to take your weight. *Have ppt remove his/her shoes and empty his/her pockets. Remove bulky clothing as well.*
BA10. Weight: ______ . __ lb (one decimal place)

Medications (ME)

The next questions are about medications used to treat hypertension or high blood pressure.

ME01. Not including vitamins and supplements, do you currently take any prescription medications or over-the-counter drugs for high blood pressure?

- ☐ No
- ☐ Yes
- ☐ Don't know
- ☐ Prefer not to answer

ME01a IF NO, Have you ever taken medication for high blood pressure?

- ☐ No → ***skip to end of medications questions***
- ☐ Yes → ***skip to end of medications questions***

If you brought your medications with you, please take them out now as we will use them to complete the next section.

ME01b INTERVIEWER CHECKPOINT - Please select one:

- ☐ Rx info not known or available – ***skip to end of ME questions; will need to follow up***
- ☐ Ppt brought medications -- ***continue***
- ☐ Ppt did not bring medications but knows info – ***continue, may need follow up***

ME01c In total how many different medications and/or over the counter drugs do you currently take for high blood pressure? Again, do not count vitamins or supplements. _________ # of BP meds

Next we're going to ask you questions about each of the medications you currently take for high blood pressure.

ME02-10a. What is the name of the first prescription medication or over-the-counter drug that you take?

____

☐ Don't know ☐ Prefer not to answer

ME02-10b. What is the dosage form?

Oral
- ☐ Pill, tablet, or capsule
- ☐ Sublingual or orally-disintegrating tablet
- ☐ Liquid solution or suspension (drink, syrup)
- ☐ Powder

Topical
- ☐ Liquid, cream, gel, or ointment
- ☐ Ear drops (otic)
- ☐ Eye drops (ophthalmic)
- ☐ Skin patch (transdermal)

Inhaled
- ☐ Inhaler or nebulizer

Injected
- ☐ Injection

Suppository
- ☐ Rectal (e.g., enema)
- ☐ Vaginal (e.g., douche, pessary)

Other:
- ☐ Other _________
- ☐ Don't know
- ☐ Prefer not to answer

ME02-10c. How frequently do you take it?

- ☐ _________ times per day
- ☐ _________ times per week
- ☐ _________ times per month
- ☐ Don't know
- ☐ Prefer not to answer

ME02-10d. What is the strength? ***(Record strength of how it is actually taken, not how it is prescribed.)***_________ %

- ☐ _________ mg
- ☐ _________ mcg
- ☐ _________ grams
- ☐ _________ I.U.
- ☐ _________ Other unit: _____________
- ☐ Don't know
- ☐ Prefer not to answer

ME02-10f. Do you take it regularly or only as needed?

- ☐ Regularly
- ☐ Only as needed
- ☐ Don't know
- ☐ Prefer not to answer

ME02-10g. For how long have you been taking it?

- ☐ For _________ days
- ☐ For _________ weeks
- ☐ For _________ months
- ☐ For _________ years
- ☐ Don't know
- ☐ Prefer not to answer

ME02-10h. What is the medication used for?

- ☐ High blood pressure
- ☐ Prescribed for something other than high blood pressure but also acts as a hypertensive medication (*describe:* ____)

ME02-10i. ***Interviewer comments***:

____

Repeat for all blood pressure medications the participant is currently taking.

<u>Self-report scales for participant to complete</u>:

PROMIS Global Health Scale v.1.2 – *standardized scale (found on proceeding pages)*

SLEEP – *1 question only taken from the Pittsburgh Sleep Quality Index*

Mindful Attention Awareness Scale (MAAS) - *standardized scale (found on proceeding pages)*

Five Facet Mindfulness Questionnaire (FFMQ) - *standardized scale (found on proceeding pages)*

SART and Mindfulness Practice questions *found on proceeding pages*

Global Health

Please respond to each question or statement by marking one box per row.

		Excellent	Very good	Good	Fair	Poor
Global01	In general, would you say your health is:	☐ 5	☐ 4	☐ 3	☐ 2	☐ 1
Global02	In general, would you say your quality of life is:..	☐ 5	☐ 4	☐ 3	☐ 2	☐ 1
Global03	In general, how would you rate your physical health?	☐ 5	☐ 4	☐ 3	☐ 2	☐ 1
Global04	In general, how would you rate your mental health, including your mood and your ability to think?	☐ 5	☐ 4	☐ 3	☐ 2	☐ 1
Global05	In general, how would you rate your satisfaction with your social activities and relationships?	☐ 5	☐ 4	☐ 3	☐ 2	☐ 1
Global09r	In general, please rate how well you carry out your usual social activities and roles. (This includes activities at home, at work and in your community, and responsibilities as a parent, child, spouse, employee, friend, etc.)......	☐ 5	☐ 4	☐ 3	☐ 2	☐ 1
		**Completely**	**Mostly**	**Moderately**	**A little**	**Not at all**
Global06	To what extent are you able to carry out your everyday physical activities such as walking, climbing stairs, carrying groceries, or moving a chair?	☐ 5	☐ 4	☐ 3	☐ 2	☐ 1

© 2010-2016 PROMIS Health Organization and PROMIS Cooperative Group

In the past 7 days…

		Never	Rarely	Sometimes	Often	Always
Global10r	How often have you been bothered by emotional problems such as feeling anxious, depressed or irritable?	☐ 5	☐ 4	☐ 3	☐ 2	☐ 1

		None	Mild	Moderate	Severe	Very severe
Global08r	How would you rate your fatigue on average?	☐ 5	☐ 4	☐ 3	☐ 2	☐ 1

Global07r	How would you rate your pain on average?	☐ 0 No pain	☐ 1	☐ 2	☐ 3	☐ 4	☐ 5	☐ 6	☐ 7	☐ 8	☐ 9	☐ 10 Worst pain imaginable

SLEEP - The following question relates to your usual sleep habits during the past month only. Your answer should indicate the most accurate reply for the majority of days and nights in the past month.

SL1_04. **During the past month**, how many hours of actual sleep did you get on average at night? (This may be different than the number of hours you spent in bed.)

AVERAGE HOURS OF SL1_EEP PER NIGHT ___________

☐ I do not know

☐ I prefer not to answer

MAAS Instructions: Below is a collection of statements about your everyday experience. Using the scale below, please indicate how frequently or infrequently you currently have each experience. Please answer according to what really reflects your experience rather than what you think your experience should be. Please treat each item separately from every other item.

Please indicate the degree to which you agree with each of the following items using the scale below. Simply check your response to each item

	Almost always	Very frequently	Somewhat frequently	Somewhat infrequently	Very infrequently	Almost never
MA1_01. I could be experiencing some emotion and not be conscious of it until some time later.	☐	☐	☐	☐	☐	☐
MA1_02. I break or spill things because of carelessness, not paying attention, or thinking of something else.	☐	☐	☐	☐	☐	☐
MA1_03. I find it difficult to stay focused on what's happening in the present.	☐	☐	☐	☐	☐	☐
MA1_04. I tend to walk quickly to get where I'm going without paying attention to what I experience along the way.	☐	☐	☐	☐	☐	☐
MA1_05. I tend not to notice feelings of physical tension or discomfort until they really grab my attention.	☐	☐	☐	☐	☐	☐
MA1_06. I forget a person's name almost as soon as I've been told it for the first time.	☐	☐	☐	☐	☐	☐

	Almost always	Very frequently	Somewhat frequently	Somewhat infrequently	Very infrequently	Almost Never
MA1_07. It seems I am “running on automatic” without much awareness of what I’m doing.	☐	☐	☐	☐	☐	☐
MA1_08. I rush through activities without being really attentive to them.	☐	☐	☐	☐	☐	☐
MA1_09. I get so focused on the goal I want to achieve that I lose touch with what I am doing right now to get there.	☐	☐	☐	☐	☐	☐
MA1_10. I do jobs or tasks automatically, without being aware of what I’m doing.	☐	☐	☐	☐	☐	☐
MA1_11. I find myself listening to someone with one ear, doing something else at the same time.	☐	☐	☐	☐	☐	☐
MA1_12. I drive places on “automatic pilot” and then wonder why I went there.	☐	☐	☐	☐	☐	☐
MA1_13. I find myself preoccupied with the future or the past.	☐	☐	☐	☐	☐	☐
MA1_14. I find myself doing things without paying attention.	☐	☐	☐	☐	☐	☐
MA1_15. I snack without being aware that I’m eating.	☐	☐	☐	☐	☐	☐

FFMQ - Please rate each of the following statements using the scale provided. Write the number in the blank that best describes *your own opinion* of what is *generally true for you*.

	1 – Never or very rarely true	2 – Rarely true	3 – Sometimes true	4- Often true	5 – Very often or always true
FF1_01. When I'm walking, I deliberately notice the sensations of my body moving.	☐	☐	☐	☐	☐
FF1_02. I'm good at finding words to describe my feelings.	☐	☐	☐	☐	☐
FF1_03. I criticize myself for having irrational or inappropriate emotions.	☐	☐	☐	☐	☐
FF1_04. I perceive my feelings and emotions without having to react to them.	☐	☐	☐	☐	☐
FF1_05. When I do things, my mind wanders off and I'm easily distracted.	☐	☐	☐	☐	☐
FF1_06. When I take a shower or bath, I stay alert to the sensations of water on my body.	☐	☐	☐	☐	☐
FF1_07. I can easily put my beliefs, opinions, and expectations into words.	☐	☐	☐	☐	☐
FF1_08. I don't pay attention to what I'm doing because I'm daydreaming, worrying, or otherwise distracted.	☐	☐	☐	☐	☐
FF1_09. I watch my feelings without getting lost in them.	☐	☐	☐	☐	☐
FF1_10. I tell myself I shouldn't be feeling the way I'm feeling.	☐	☐	☐	☐	☐
FF1_11. I notice how foods and drinks affect my thoughts, bodily sensations, and emotions.	☐	☐	☐	☐	☐
FF1_12. It's hard for me to find the words to describe what I'm thinking.	☐	☐	☐	☐	☐
FF1_13. I am easily distracted.	☐	☐	☐	☐	☐
FF1_14. I believe some of my thoughts are abnormal or bad and I shouldn't think that way.	☐	☐	☐	☐	☐
FF1_15. I pay attention to sensations, such as the wind in my hair or sun on my face.	☐	☐	☐	☐	☐

	1 – Never or very rarely true	2 – Rarely true	3 – Sometimes true	4- Often true	5 – Very often or always true
FF1_16. I have trouble thinking of the right words to express how I feel about things.	☐	☐	☐	☐	☐
FF1_17. I make judgments about whether my thoughts are good or bad.	☐	☐	☐	☐	☐
FF1_18. I find it difficult to stay focused on what's happening in the present.	☐	☐	☐	☐	☐
FF1_19. When I have distressing thoughts or images, I "step back" and am aware of the thought or image without getting taken over by it.	☐	☐	☐	☐	☐
FF1_20. I pay attention to sounds, such as clocks ticking, birds chirping, or cars passing.	☐	☐	☐	☐	☐
FF1_21. In difficult situations, I can pause without immediately reacting.	☐	☐	☐	☐	☐
FF1_22. When I have a sensation in my body, it's difficult for me to describe it because I can't find the right words.	☐	☐	☐	☐	☐
FF1_23. It seems I am "running on automatic" without much aware- ness of what I'm doing.	☐	☐	☐	☐	☐
FF1_24. When I have distressing thoughts or images, I feel calm soon after.	☐	☐	☐	☐	☐
FF1_25. I tell myself that I shouldn't be thinking the way I'm thinking.	☐	☐	☐	☐	☐
FF1_26. I notice the smells and aromas of things.	☐	☐	☐	☐	☐
FF1_27. Even when I'm feeling terribly upset, I can find a way to put it into words.	☐	☐	☐	☐	☐
FF1_28. I rush through activities without being really attentive to them.	☐	☐	☐	☐	☐

	1 – Never or very rarely true	2 – Rarely true	3 – Sometimes true	4- Often true	5 – Very often or always true
FF1_29. When I have distressing thoughts or images, I am able just to notice them without reacting.	☐	☐	☐	☐	☐
FF1_30. I think some of my emotions are bad or inappropriate and I shouldn't feel them.	☐	☐	☐	☐	☐
FF1_31. I notice visual elements in art or nature, such as colors, shapes, textures, or patterns of light and shadow.	☐	☐	☐	☐	☐
FF1_32. My natural tendency is to put my experiences into words.	☐	☐	☐	☐	☐
FF1_33. When I have distressing thoughts or images, I just notice them and let them go.	☐	☐	☐	☐	☐
FF1_34. I do jobs or tasks automatically without being aware of what I'm doing.	☐	☐	☐	☐	☐
FF1_35. When I have distressing thoughts or images, I judge myself as good or bad depending what the thought or image is about.	☐	☐	☐	☐	☐
FF1_36. I pay attention to how my emotions affect my thoughts and behavior.	☐	☐	☐	☐	☐
FF1_37. I can usually describe how I feel at the moment in consider- able detail.	☐	☐	☐	☐	☐
FF1_38. I find myself doing things without paying attention.	☐	☐	☐	☐	☐
FF1_39. I disapprove of myself when I have irrational ideas.	☐	☐	☐	☐	☐

Sustained Attention to Response Task

The Sustained Attention to Response Task (SART) is a computerized test of sustained attention, response inhibition (executive function) and self-regulation. Subjects are instructed to press a key in response to rapidly displayed integers (1-9) and withhold response to a designated "no-go" integer. SART errors consist of summed commission errors (button press on no-go trial) and omission errors (button not pressed on "go" integers). SART performance is associated with prefrontal cortex functioning, has been found to increase with mindfulness training and is correlated with scores on mindfulness questionnaires (specifically, the Mindful Attention Awareness Scale).

MP1_01 Think about the last 12 months. During that time, have you practiced mindfulness, either formally or informally, in any way?

No .... 0 (***Skip to MP1_03***)
Yes 1

On average, how minutes PER WEEK do you engage in the following types of mindfulness activities?	Average # Minutes per week	Less than weekly	*Don't Know*	*Prefer not to answer*
MP1_02a Body Scan	____	666	777	888
MP1_02b Yoga	____	666	777	888
MP1_02c Awareness of breath meditation	____	666	777	888
MP1_02d Sitting Meditation	____	666	777	888
MP1_02e Walking Meditation	____	666	777	888
MP1_02f Loving-kindness Meditation	____	666	777	888
MP1_02g Mountain Meditation	____	666	777	888
MP1_02h Visual Meditation	____	666	777	888
MP1_02i Eating Meditation	____	666	777	888
MP1_02j Meditation moving through regions, such as breath, physical sensations, sound, thoughts, and open awareness	____	666	777	888
MP1_02k Goal-related activity (e.g., physical activity, diet change, etc.). *Please describe*______________________________	____	666	777	888

MP1_02l Other mindful activities:<br>Other 1 *describe:* _______________ ....<br>Other 2 *describe:* _______________ ....<br>Other 3 *describe:* _______________	____	666	777	888

MP1_03 Which of the following statements, BEST describes your current attitude towards mindfulness?

I do not plan on practicing mindfulness 1
I see value in mindfulness but do not practice it regularly...........2
I practice mindfulness regularly...3
Other (*please describe* ____________________________)......4
Don't Know 7
Prefer not to answer..8

MP1_04 Which of the following, if any, have you participated in [since completing the course / the last six months]?

None, I have not practiced mindfulness 00

Check ALL that apply

MB-BP Study Regular 1-1.5 hour Booster Sessions.......[ ]
MB-BP Study All Day Retreats.......................................[ ]
Meditation group *not* related the MB-BP Study ....[ ]
Mindful yoga group *not* related the MB-BP Study ....[ ]
Other 1 *describe:* ________________ ....[ ]
Other 2 *describe:* ________________ ....[ ]
Other 3 *describe:* ________________ ....[ ]
Don't Know...77
Prefer not to answer..88

MP1_05 We are interested in the ways in which mindfulness practice may or may not impact your life. Please describe below your relationship to mindfulness since completing your course.

Don't Know 7
Prefer not to answer..8

Appendix 5 -
UMass Medical School fMRI Study
Recruitment Talking Points

This form will be utilized by research staff at Brown University to approach MB-BP participants about possible involvement in a related sub-study

Recruitment Talking Points for UMass Medical School fMRI sub-study

As a participant in the Mindfulness-based Blood Pressure Reduction (MB-BP) you may be eligible to take part in a related clinical trial that involves receiving two separate brain scans or functional magnetic resonance imaging (fMRI) of your brain. Participation in this additional study in entirely optional and will not affect your ability to participate in the MB-BP study in any way. Would you be interested in hearing more about the study?

If Yes - Go over study details below with participant.

- This sub-study is being conducted by investigators at the UMass Medical School and is funded by the same NIH grant that is funding the MB-BP Study.
- Eligible participants who enroll in the fMRI study will be compensated $150 USD for each neuroimaging session ($300 USD total) completed.
- Neuroimaging will take place at the UMass Medical School at the Center for Comparative NeuroImaging (CCNI).
- Travel to and from UMass Medical School from Providence, Rhode Island is around 100 minutes (82 miles) round trip. Part of the compensation is intended to cover the cost of travel. There is free parking available for participants. If a participant does not have his/her own mode of transportation, study staff can arrange for transportation (e.g., Uber).
- Each participant will have two visits to UMass Med School Campus - before and after the MB-BP intervention (Visit 1 and 2, respectively). Each visit will include an MRI scan and is expected to last approximately 2 hours.
- Eligibility for the fMRI study will be determined by the research staff at UMass Medical school by completing a brief (15 minute) phone interview. However, some of the exclusion criteria include but are not limited to: contraindication(s) to entering the MR scanner such as the presence of metal in or on the body that cannot be removed or claustrophobia; pregnancy or trying to get pregnant; and a body weight of over 300 lbs.
- Informed consent will take place at UMass Medical School in person and all study procedures will be carried out by UMass research staff.
- The goal of this research is to determine the mental and neural mechanisms implicated in the modulation of blood pressure (associated with cardiovascular risk) by the adoption of a mindfulness-based blood pressure therapy.
- They are looking to enroll and scan up to 60 individuals from the MB-BP study.

Would you be interested in being contacted by the research staff at the UMass Medical School regarding this study? Note that by saying yes you'd only be agreeing to allow us to pass along your contact information so that someone at CCNI could call you for the 15-minute phone screener. We are not asking you to sign up at this time.

Appendix 6 -
UMass Medical School IRB Submission
for fMRI sub-study

This includes the study protocol, informed consent form, phone screener, questionnaire, and IRB Approval

Appendix 6A -
Study Protocol for UMass Medical School fMRI sub-study

1. TITLE

Mindfulness Influences on Self-Regulation: Mental and Physical Health Implications for blood pressure (Mindful Self-Regulation for blood pressure MRI study)

2. EXTERNAL IRB REVIEW HISTORY*

NA

3. PRIOR APPROVALS:

Participants recruited will be from those eligible participants already enrolled in a project for mindfulness intervention for blood pressure ongoing at Brown University (Dr. Eric Loucks), entitled Mindfulness-Based Blood Pressure Reduction (MB-BP) Study (formerly known as Mindfulness-Based Hypertension Therapy (MBHT).

There are no conflicts of interest, medical devices, biohazardous agents, or radiation.

4. OBJECTIVES*

The goal of this research is to determine the mental and neural mechanisms implicated in the modulation of blood pressure (associated with cardiovascular risk) by the adoption of a mindfulness-based blood pressure therapy. All Participants, pre-selected based on Brown University MB-BP study enrollment, will have met entrance criteria for having prehypertension or hypertension, or another cardiovascular risk factor that could be influenced by this program. We aim to assess the neural correlates of mindfulness training on self-regulation, specifically inhibitory control, self-compassion, interoceptive attention, and emotion regulation, in these chronic disease patients.

We hypothesize that the blood pressure changes induced by the tailored mindfulness-based intervention will be associated with the differential engagement of brain regions implicated in emotional self-regulation. Specifically, we hypothesize that alterations both in resting state brain function and evoked upon prompts such as interoceptive attention, self-compassion, and inhibitory control will notably be linked to physiological measures of the autonomic system. We hypothesize that the altered cardiovascular risk measures can be understood through using advanced neuroimaging techniques such as functional connectivity and diffusion tensor imaging.

5. BACKGROUND*

Self-regulation, or the ability to intentionally manage cognitive and emotional resources to accomplish goals, is crucial to addressing a wide range of health problems influenced by common behaviors, such as excessive eating, lack of physical activity, addiction, and poor adherence to medical regimens. Mindfulness interventions have initial evidence to influence self-regulation, however it is still poorly understood what specific elements of mindfulness interventions are most effective at influencing self-regulation, and if those changes in self-regulation translate into clinically

meaningful health behavior changes, such as improved medial regimen adherence. This study proposes to test the ability of customized mindfulness interventions to alter self-regulation in an ongoing study.

Mindfulness training is an evidence-based approach to patient-centered caring[1] and whole-person self-management.[2] Mindfulness is "the awareness that emerges through purposely, and non-judgmentally, paying attention to the unfolding of experience moment-by-moment".[3]

Developed at the University of Massachusetts Medical School by Jon Kabat-Zinn, Saki Santorelli, and colleagues, Mindfulness-based Stress Reduction (MBSR) is an 8-week program that serves as the basis for multiple different condition-specific mindfulness-based interventions (MBI) provided in medical settings.[4] In 2013, National Institutes of Health invested 44.3 million U.S. dollars into direct funding for MBI research (NIH Reporter 2014), and publications related to mindfulness-related research have exponentially increased over the past 30 years, with more than 500 publications in 2013 alone.[5]

Multiple clinical trials have demonstrated that MBIs improved self-management in major areas of community health and chronic illness management.[6, 7] One recent meta-analysis demonstrated that MBIs are broadly effective for anxiety, depression, and pain.[8] MBIs are associated with tobacco smoking reduction,[9, 10] reduction in alcohol consumption,[11, 12] reduction in prescription opioid misuse among patients with chronic pain[13] and reduced HIV-risk behaviors.[14] MBIs demonstrate increased asthma-related quality of life with reductions in stress and anxiety.[15] MBIs produce a reduction in systolic and diastolic blood pressure in patients with pre-hypertension,[16] and particularly large reductions in hypertension have been seen within studies of African-American patients.[17, 18] Among patients with non-insulin dependent diabetes, one small MBI study demonstrated improved glycemic control,[19] while large studies with active controls have demonstrated effective integration of diabetes self-management skills,[20] decreased stress and improved quality of life.[21] MBIs can reduce the stress associated with chronic illness,[6, 7] and they are particularly effective in reducing depressive relapse in Major Depressive Disorder.[22]

Despite all the evidence supporting MBIs, several gaps in the current research and models may be acting as barriers to rapid dissemination and integration of mindfulness into the healthcare delivery system. First, while a multitude of patient studies showing reductions in stress, pain, anxiety, and depression exist, little is known about any impact on the ability to self-manage and cope with chronic illness—although there is initial evidence for positive outcomes on these measures. Second, this study offers an opportunity to identify and test mechanistic self-regulation targets (physiologic, psychological, neuropsychological, etc.) that are influenced by mindfulness, in the context of a chronic medical condition.

The project has been funded as a core clinical trial for the NCCIH Science of Behavior Change UH2 project "Mindfulness Influences on Self-Regulation: Mental and Physical Health Implications." We will conduct an experimental MRI study to evaluate several mechanistic self-regulation targets of the Mindfulness-based blood pressure therapy (MB-BP), their neural substrates, and their potential impact on autonomic system measures.

The 8-week, 9-session MB-BP curriculum (approved for ongoing study at Brown University) is based on systematic and intensive training in mindfulness meditation and mindful hatha yoga.

The curriculum, which is based on the manualized and standardized MBSR curriculum, is designed to teach program participants how to integrate and apply mindfulness in their everyday lives to the range of challenges arising from medical and psychological conditions and life stresses. Furthermore, this program has been further customized to participants that have prehypertension or hypertension.

Embedded within the context of behavioral medicine, the MB-BP curriculum focuses on the experiential cultivation of both “formal” and “informal” mindfulness practices as a foundation for the cultivation of positive health behaviors and psychological and emotional resilience that can be effective utilized across the adult life span. The approach supports the learning, strengthening and integration of a range of mindfulness-based self-regulatory skills through the development and refinement of inherent internal resources. A primary aim is to cultivate ways of learning and being that can be utilized far beyond the completion of the program.

MB-BP builds a foundation of mindfulness skills (e.g. meditation, self-awareness, etc.) through the MBSR curriculum. MB-BP then directs attention towards hypertension risk factors. Early in the MB-BP, the importance of hypertension for health and mortality is described, along with hypertension risk factors. Participants will have their blood pressure and hypertension risk factors assessed at baseline, and be provided with this information during the first in-person MB-BP session. This phase aims to engage participants’ interest in hypertension risk factors, and increase motivation for behavior change. MB-BP encourages participants to explore personal readiness for change in the different hypertension risk factors, and explore utilizing mindfulness practices to engage with those risk factors that they choose to. Each week, focus is provided on different hypertension risk factors. However, common themes exist across all hypertension risk factors including (1) awareness of thoughts, emotions and physical sensations particularly surrounding hypertension risk factors such as overconsumption of palatable foods, sedentary activities, alcohol consumption, medication adherence; (2) craving, particularly for hypertension risk factors such as overconsumption of palatable foods, sedentary activities, and alcohol consumption; (3) the impact of bringing mindfulness to every moment, particularly in relation to hypertension risk factors. For example, when consuming highly palatable food, bringing awareness to the emotions, thoughts and physical sensations prior to eating it, during eating it, along with the many minutes, if not hours, afterwards. Participants are trained to bring non-judgmental attention to the often short-term pleasure of overconsumption of foods, sedentary activities, heavy alcohol consumption, or not taking medications, and bring non-judgmental attention to the longer term suffering associations with these activities. Through this process, participants are encouraged to reflect on if behavioral choices provide more benefit or harm to their well-being, and to choose the behaviors that bring benefit. (4) Self-care: as awareness of thoughts, emotions and physical sensations increases, and self-regulation will likely increases as a result of the meditation practices, the curriculum will emphasize to participants that it is common for people to start caring for themselves more. It is a way of better knowing ourselves, and through knowing ourselves in in each moment, we often want to care for ourselves in each moment. This may mean taking medication that will help our health, or being more physical active, eating more healthily, or consuming more moderate amounts of alcohol. The intervention can be modified to change the dose of any of these 4 components, for example via (1) Increased use of specific meditations focused on health behaviors such as diet and physical activity. (2) Increased use of meditations focused on craving. Participants will be trained to notice craving

arising, and fading away in relation to hypertension risk factors such as palatable high caloric foods, sedentary activities, and alcohol consumption. (3) Increased used of mindfulness practices related to long term experiences of health behaviors such as alcohol consumption, diet, physical activity and medication use on thoughts, emotions, physical sensations and well-being. (4) Self-care: Increased use of modules that show how mindfulness practice increased self-awareness and in doing so can increase people's desire to care for themselves. The potential use of loving kindness meditation will be considered.

REFERENCES

1. Krasner, M.S., R.M. Epstein, H. Beckman, A.L. Suchman, B. Chapman, C.J. Mooney, and T.E. Quill, *Association of an educational program in mindful communication with burnout, empathy, and attitudes among primary care physicians.* Jama, 2009. **302**(12): p. 1284-1293.
2. Benzo, R.P., *Mindfulness and motivational interviewing: two candidate methods for promoting self-management.* Chronic respiratory disease, 2013. **10**(3): p. 175-182.
3. Kabat-Zinn, J., *Wherever you go, there you are: Mindfulness meditation in everyday life*. 1994, Hyperion: New York. p. 277.
4. Cullen, M., *Mindfulness-based interventions: An emerging phenomenon.* Mindfulness, 2011. **2**(3): p. 186-193.
5. Black, D., *Research publications on mindfulness*. 2014.
6. Merkes, M., *Mindfulness-based stress reduction for people with chronic diseases.* Australian Journal of Primary Health, 2010. **16**(3): p. 200-210.
7. Niazi, A.K. and S.K. Niazi, *Mindfulness-based stress reduction: a non-pharmacological approach for chronic illnesses.* North American journal of medical sciences, 2011. **3**(1): p. 20.
8. Goyal, M., S. Singh, E.M. Sibinga, N.F. Gould, A. Rowland-Seymour, R. Sharma, Z. Berger, D. Sleicher, D.D. Maron, and H.M. Shihab, *Meditation programs for psychological stress and well-being: a systematic review and meta-analysis.* JAMA internal medicine, 2014. **174**(3): p. 357-368.
9. Brewer, J.A., S. Mallik, T.A. Babuscio, C. Nich, H.E. Johnson, C.M. Deleone, C.A. Minnix-Cotton, S.A. Byrne, H. Kober, and A.J. Weinstein, *Mindfulness training for smoking cessation: results from a randomized controlled trial.* Drug and alcohol dependence, 2011. **119**(1): p. 72-80.
10. Bowen, S., K. Witkiewitz, S.L. Clifasefi, J. Grow, N. Chawla, S.H. Hsu, H.A. Carroll, E. Harrop, S.E. Collins, and M.K. Lustyk, *Relative efficacy of mindfulness-based relapse prevention, standard relapse prevention, and treatment as usual for substance use disorders: a randomized clinical trial.* JAMA psychiatry, 2014. **71**(5): p. 547-556.
11. Garland, E.L., N.R. Schwarz, A. Kelly, A. Whitt, and M.O. Howard, *Mindfulness-oriented recovery enhancement for alcohol dependence: Therapeutic mechanisms and intervention acceptability.* Journal of social work practice in the addictions, 2012. **12**(3): p. 242-263.
12. Ostafin, B.D., K.T. Kassman, and I. Wessel, *Breaking the cycle of desire: Mindfulness and executive control weaken the relation between an implicit measure of alcohol*

valence and preoccupation with alcohol-related thoughts. Psychology of addictive behaviors, 2013. **27**(4): p. 1153.
13. Garland, E.L., E.G. Manusov, B. Froeliger, A. Kelly, J.M. Williams, and M.O. Howard, *Mindfulness-oriented recovery enhancement for chronic pain and prescription opioid misuse: results from an early-stage randomized controlled trial.* Journal of Consulting and Clinical Psychology, 2014. **82**(3): p. 448.
14. Margolin, A., Z. Schuman-Olivier, M. Beitel, R.M. Arnold, C.E. Fulwiler, and S.K. Avants, *A preliminary study of spiritual self-schema (3-S+) therapy for reducing impulsivity in HIV-positive drug users.* Journal of clinical psychology, 2007. **63**(10): p. 979-999.
15. Pbert, L., J.M. Madison, S. Druker, N. Olendzki, R. Magner, G. Reed, J. Allison, and J. Carmody, *Effect of mindfulness training on asthma quality of life and lung function: a randomised controlled trial.* Thorax, 2012: p. thoraxjnl-2011-200253.
16. Hughes, J.W., D.M. Fresco, R. Myerscough, M. van Dulmen, L.E. Carlson, and R. Josephson, *Randomized controlled trial of mindfulness-based stress reduction for prehypertension.* Psychosomatic medicine, 2013. **75**(8).
17. Schneider, R.H., F. Staggers, C.N. Alexander, W. Sheppard, M. Rainforth, K. Kondwani, S. Smith, and C.G. King, *A randomized controlled trial of stress reduction for hypertension in older African Americans.* Hypertension, 1995. **26**(5): p. 820-827.
18. Palta, P., G. Page, R. Piferi, J. Gill, M. Hayat, A. Connolly, and S. Szanton, *Evaluation of a mindfulness-based intervention program to decrease blood pressure in low-income African-American older adults.* Journal of Urban Health, 2012. **89**(2): p. 308-316.
19. Rosenzweig, S., D.K. Reibel, J.M. Greeson, and J.S. Edman, *Mindfulness-based stress reduction is associated with improved glycemic control in type 2 diabetes mellitus: a pilot study.* Alternative Therapies in Health and Medicine, 2007. **13**(5): p. 36.
20. Miller, C.K., J.L. Kristeller, A. Headings, and H. Nagaraja, *Comparison of a mindful eating intervention to a diabetes self-management intervention among adults with type 2 diabetes: a randomized controlled trial.* Health Education & Behavior, 2014. **41**(2): p. 145-154.
21. van Son, J., I. Nyklíček, V.J. Pop, M.C. Blonk, R.J. Erdtsieck, P.F. Spooren, A.W. Toorians, and F. Pouwer, *The effects of a mindfulness-based intervention on emotional distress, quality of life, and HbA1c in outpatients with diabetes (DiaMind).* Diabetes care, 2013. **36**(4): p. 823-830.
22. Piet, J. and E. Hougaard, *The effect of mindfulness-based cognitive therapy for prevention of relapse in recurrent major depressive disorder: a systematic review and meta-analysis.* Clinical psychology review, 2011. **31**(6): p. 1032-1040.

6. Inclusion and Exclusion Criteria*

Participants will have been enrolled in Brown University study with the following criteria

Inclusion Criteria:
-men or women
-between the ages of 18 and 65
-participants that have prehypertension or hypertension.

Exclusion Criteria:
-Current regular mindfulness practice (>1 week) or lifetime mindfulness meditation practice
-Diagnosis of an active medical illness
-Diagnosis of an active major psychiatric disorder
-Current medication use consistent with an active medical illness or psychiatric disorder
-A priori unable to commit time required for the study
-Inability to consent

Exclusion Criteria for the present MRI application

-Contraindication(s) to entering the MR scanner such as the presence of metal in or on the body that cannot be removed or claustrophobia
-Pregnancy
-Weight > 300 lbs

7. STUDY-WIDE NUMBER OF SUBJECTS*

60 participants in total, from those already recruited for ongoing, non-MRI study through Brown University study, all meeting study entrance criteria for having prehypertension or hypertension, or another cardiovascular risk factor that could be influenced by this program; half enrolled in MB-BP at Brown University, whereas the other half will be waitlist controls.

8. STUDY-WIDE RECRUITMENT METHODS*

All participants will have been previously recruited for a study at Brown University. Eligible and interested participants will be further recruited for this MRI add-on.

9. STUDY TIMELINES*

Each participant will have two visits to UMass Med School Campus - before and after the MB-BP intervention (Visit 1 and 2, respectively). Each visit will include an MRI scan and is expected to last approximately 2 hours.

The endpoint of the study is after the second study visit (Visit 2) has been completed. However, the PI and study personnel may make study termination decisions in coordination with participants. A participant's involvement in this study will also end if the subject withdraws their consent, is unable to adhere to the study protocol (e.g., finds

the MRI procedure excessively uncomfortable), or is determined to be ineligible during the study by the investigators.

We anticipate that it will take approximately 3 years to enroll all 60 study participants, and that it will take 1 year to complete the primary analyses.

10. STUDY ENDPOINTS*

The primary outcome will be differential over-time change in MRI resting-state functional connectivity (rsFC) patterns for the intervention versus comparison group (e.g. default-mode, salience, and central executive, networks comprising regions such as anterior cingulate cortex, medial orbital cortex, insula, amygdala, and hippocampus). A significant group x time interaction will represent differential over-time change in the outcome (rsFC) for the intervention versus comparison group. The model for Hypothesis 1 will be parameterized with indicator variables for study group, time, and a group-time interaction (for example using FSL general linear model (GLM), which can estimate multivariable models, where the response *Y* at each voxel is modeled as a linear combination of one or more predictors; applying "Randomise" permutation function). As such we will also apply such a technique of regression modeling to predict change in hypertensive functional status, specifically blood pressure [standard-of-care clinic-assessed systolic and diastolic BP for monitoring hypertension treatment: baseline clinic BP obtained at two in-person screening visits ≥1 week apart, followed by in-person assessments at all follow-up periods (8w, 3m, 6m), all as part of Brown University project], while considering important clinical and demographic covariates.

The secondary outcomes will be in-scanner self-regulation task functional connectivity outcomes (1. emotional Go/NoGo inhibitory control task; 2. self-compassion task, which contrasts self-reassurance conditions with a self-criticism condition; 3. interoceptive attention task, which contrasts an interoceptive attention condition with an exteroceptive attention condition). We will test for differential brain connectivity relation to each self-regulation manipulation between the intervention versus comparison group. Finally, associations between any differential intervention effect on self-regulation-task associated brain connectivity change will be further modeled for predictive ability of blood pressure change.

11. PROCEDURES INVOLVED*

Participation will entail a total time commitment of about 4.5 hours over the course of the study.

<u>Telephone Screen</u>:~15 minutes

Potential participants, already enrolled in Brown University study, will be prescreened by telephone. Respondents are informed that everything they say will be kept strictly confidential and they may stop or ask questions at any time.

Respondents will already have satisfied age and medical or psychiatric illness inclusion and exclusion criteria for participation enrollment in Brown University study.

For this MRI study, women will be asked if they are currently pregnant or planning to become pregnant. If the subject meets all inclusion criteria and no exclusion criteria (see #6 Inclusion and Exclusion Criteria, above), they will be asked for their contact information (phone number and email address) and about scheduling considerations for providing consent, and determine study visits. This information will be stored in a locked cabinet.

Participants who do not meet eligibility criteria will be asked to verbally provide consent to be re-contacted for future studies. If consent is obtained, this will be recorded on the phone screen which will be stored in a locked cabinet, accessible only by study staff.

Informed and Written Consent:~15 minutes

Consent will be obtained at Umass Medical School. The subject consent form will be reviewed in detail with the participant by trained study personnel. If needed, the participant will be given the consent form to take home for further consideration and discussion prior to signing. Sufficient time will be provided so that the subject can understand the study, ask questions, and express concerns.

Once the subject has read and understood the consent form and determined that they wish to participate in study, the IRB-approved written informed consent form will be signed and dated by the subject and the study personnel obtaining consent. The participant will be given a copy of the signed informed consent form; originals shall be kept on file by the investigator in a locked cabinet.

The study visit will proceed with the neuroimaging session to examine brain function and structure using diffusion tensor imaging (DTI), resting state and task-based functional magnetic resonance imaging (fMRI), as well as arterial spin labeling.

Neuroimaging sessions: ~1.5 hours each

Subjects will undergo two MRI sessions – one at each of 2 study visits. These will include anatomic scans, arterial spin labeling, Diffusion Tensor Imaging (DTI), as well as resting state and task-based fMRI. Imaging will be conducted on a 3 Tesla magnetic resonance imaging scanner (Phillips 3 Tesla Ingenia CX dStream), which has been approved for research and clinical studies in children and adults by the FDA. Magnetic resonance (MR) technology does not use ionizing radiation like an X-ray. Instead, it uses strong magnetic fields and radio waves to collect the images and data. There are no known hazards or risks associated with these techniques. To ensure subjects can safely undergo MRI scanning, all participants will be screened by the Research Coordinator prior to scanning for the presence of any MRI incompatible medical implant, external decide or metal (including jewelry, body piercings, tattoos with metallic pigments).

Acquisitions will include T1 and T2 images (in agreement with NINDS Common Data Elements, and suitable for incorporation in FITBIR), T1-weighted, ME-MPRAGE sequence; T2-weighted double echo fast spin echo sequence, (2) a multi-flip angle 3D spoiled gradient echo (FLASH type) compatible with the BIRN protocol; Resting state and functional MRI: a multiband echo-planar imaging sequence with T2*-weighted BOLD contrast; a fieldmap for distortion correction; Diffusion Tensor Imaging sequence that uses a standard single shot, spin echo, echo planar acquisition with diffusion weighting gradient pulses comprised of multiple diffusion-encoding directions with additional weighted images; Arterial spin labeling ASL): pseudo-continuous ASL (PCASL) pulse sequence complemented by a single PCASL image with long repetition time (TR; proton density) for scaling perfusion-weighted maps to absolute units of cerebral blood flow (CBF; ml/100g/min). During the imaging session, autonomic system measures will also be obtained using respiratory belt, finger plethysmograph for heart rate (HRV), and finger sensors for skin conductance.

A quick questionnaire about mental and physical state during the scanning session Amsterdam Resting-State Questionnaire (ARSQ 2.0) will be administered immediately after each MRI scanning session (< 5 minutes).

Post-processing of MRI data will be performed with standard software (Freesurfer, FSL, and DTIStudio) to quantify the regional gray and white matter structural properties, as well as functional, and arterial properties in the brain.

12. Data and Specimen Banking*

Neuroimaging data and accompanying physiological, behavioral, and questionnaire, as well as participant data including telephone screening informed consent forms will be kept. All personally identifiable information will be "de-identified", and coded with the subject's identification number. Thus, all study data will be stored without personal identifiers.

All electronic information will be stored on a password-protected server accessible by study personnel only or on personal employee computers, located in locked offices at UMass Medical School. All written information will be locked in a file cabinet, located in a locked office. The office referred to is in the Center for Comparative NeuroImaging (CCNI). The CCNI is a secure building with swipe card entry access. Subjects will be assigned a code for all documentation and data analysis sheets. The key linking the names and codes will be stored on this password-protected server. Documents that contain identifying information will be stored separately from any primary research data. Imaging data will be transferred from the MRI system by means of a portable drive. Once the transfer is completed, the portable drive will be formatted to clean it of the acquired data. The data will only be stored in a password-protected computer accessible to study investigator and study staff. We also push the neuroimaging data to a server within the UMASS system. This server is accessible only by the authorized personnel and it is backed-up regularly to password-protected drives. Aside from UMMS IRB representatives, only approved study personnel will have access to raw research data.

The master list of subjects' addresses and phone numbers will be destroyed at the end of the study. Data will be kept at least for a minimum of 5 years following publication of results, in accordance with American Psychological Association guidelines for data retention, and likely indefinitely. While there is no plan to destroy the raw data, if we later decide to destroy the raw data, paper raw data will be shredded and electronic raw data will be erased from hard drives and any electronic backup media. For the sake of transparency in our research practices, according to recommended practices the de-identified neuroimaging, physiological, task, questionnaire, and other participant data relevant for analysis may be made available to the public via a web repository (e.g. Open Science Framework). In that case, any de-identified data that have been posted publicly will remain publicly accessible in perpetuity.

13. Data Analysis and Management*

We will follow current recommended methods for MRI data analysis. The fMRI data will be analyzed using the standard software package FSL, together with RETROICOR for retrospective correction of physiological motion artifacts using our peripheral measures of cardiac and respiratory activity as independent assessments of physiological noise in the blood oxygen level-dependent (BOLD) signal. Following standard preprocessing steps (de-warping, slice timing correction, motion correction, removal of physiological noise signals), the statistical analysis of brain activation data will be performed.

In addition to time point and group assignment, the statistical models will include among regressors of interest blood pressure, gender, age, in-scanner task outcomes, and the following regressors of no interest: head motion parameters and cardiac and respiratory components of physiological noise. Hypotheses will be expressed as: Is there any significant difference between the 2 time points in functional connectivity during resting state and task-specific induction (a, NoGo trials versus Go trials; b) self-reassurance versus self-criticism; and c) Interoception-versus-Exteroception. The null hypothesis will be that any brain network functional connectivity changes between the 2 time points is not significantly different between the intervention and the comparison groups. Significance testing will be done using permutation methods.

We estimate that after outlier or neuroimaging artifact exclusion and any withdrawals from the study, there will be about 50 total participants. Data analysis will be performed using standard statistical software. Based on our current observations, we may obtain high effect sizes, i.e. 1 or 1.5. We will recruit up to 60 participants so that we can exclude outliers.

14. Provisions to Monitor the Data to Ensure the Safety of Subjects*

A trained neuroradiologist associated with the MRI imaging center at University of Massachusetts Medical School will read the diagnostic MRI scans. In the event that there is a suspicious reading on the MRI, the principal investigator will be informed of this and arrange for the neuroradiologist to share with the subject the findings and facilitate in helping them determine what follow-up care is needed and appropriate.

15. Withdrawal of Subjects Without Their Consent*

A subject's participation in this neuroimaging study will normally end at the completion of the visit. However, subjects may withdraw at any time and for any reason. If the subject decides to withdraw, no special procedures need to be followed. The subject can simply leave the study at any time. Also, the trained study personnel may make study termination decisions if the subject is not able to adhere to the study protocol.

16. Risks to Subjects*

This study will be conducted in a 3T MR scanner which has been approved for research and clinical studies in children and adults by the FDA. Magnetic resonance (MR) technology does not use ionizing radiation like an X-ray. Instead, it uses strong magnetic fields and radio waves to collect the images and data. There are no known hazards or risks associated with these techniques.

As it is the case for any MR scanner, significant risks can arise if ferromagnetic materials (this includes many types of common metal objects) are brought into the high magnetic field environment of the scanner and immediate vicinity, as they can become hazardous projectiles.

During the scan, some people do report claustrophobia (fear of being in enclosed small spaces), dizziness, mild nausea, headaches, a metallic taste in their mouth, double vision, or sensation of flashing lights. In rare cases during certain types of scans, a very slight, uncomfortable tingling of the back due to the rapid switching of the magnetic field has been reported. Although the intensity is not harmful to their hearing, the sounds that subjects hear inside the scanner may be annoying.

During the scan, the comfort of the participant will be checked through the intercom system. The MRI technician can talk through the intercom system and the participant can hear through the head phones he/she will be wearing during the experiment. Similarly, the participants can talk during the experiment (in between the acquisitions) and the MRI technician can hear through the intercom system. In the unlikely event of any discomfort to the participant, the scan session will immediately be stopped and the participant will be taken out of the magnet.

Identification of a real or false positive abnormality, i.e. lesion or a tumor, during the MRI examination is possible. In the existence of such a condition, further investigation and/or treatment may be necessary. Although possible abnormalities are not related to the study, there may be emotional and physical risks associated with getting the follow-up medical evaluations. The initial diagnosis revealed by our scan may be false positive or it may be a true positive finding that may lead only to earlier identification of a condition. However, this condition may not have a survival possibility and the subject would need to live longer with the knowledge of this condition.

Breach of Confidentiality:

As with all studies, there is a chance that data could be linked back to a subject's true identity, breaching confidentiality, and so there is a risk of psychological and social stigma that could adversely impact the subject. However, there will be many safeguards in place to prevent this occurrence.

17. POTENTIAL DIRECT BENEFITS TO SUBJECTS*
Not Applicable

18. VULNERABLE POPULATIONS*
Not Applicable

19. MULTI-SITE RESEARCH*

This study is a collaboration between UMass Medical School (UMMS), Brown University, and MGH. All imaging acquisition will be done at UMMS. All imaging analyses will be done at UMMS, in collaboration with MGH for analysis of autonomic system data [respiratory belt, finger plethysmograph for heart rate (HRV), and finger sensors for skin conductance]. All recruitment, MB-BP intervention and other measures are done at Brown University.

20. COMMUNITY-BASED PARTICIPATORY RESEARCH*
Not Applicable

21. SHARING OF RESEARCH RESULTS WITH SUBJECTS*

Results will not be routinely shared with subjects. However, if an MRI reading is indicative of pathology, such as hemorrhagic stroke, ischemic stroke, multiple sclerosis, tumors, and aneurysms, these results will be shared with subjects. In addition, during the clinical interview the study clinicians may determine that the study subject has psychiatric symptoms that are in need of treatment, or that the subject might be harmful to themselves or someone else. In this case, these results will be shared with the subject, and we will help the subject find appropriate care.

22. SETTING

All participants will be recruited from an ongoing MB-BP study at Brown University, for the neuroimaging visits at UMMS.

Facilities:

Center for Comparative NeuroImaging (CCNI):

- **Telephone Screens and Data Analysis will be performed at the CCNI.**

- **Informed Consent will be done at the CCNI in a private office.**

The CCNI is a research laboratory of ~3,000sq-ft building on the main campus. This center contains a conference room, faculty offices (~140 square feet each), secretary cubicle, shared student room, a wet lab, and a small room for mechanical and electronic work.

CCNI offices and laboratories are networked with switched 10/100 Mb/sec TCP/IP communication lines. CCNI currently has five SGI, two Sun stations, and ten PC's. Some of these SGI and PC are in a "common" area where collaborators will have access to them for data analysis and software

In addition to the shared computers in the CCNI, we have computers within the offices located in BNRI. There are two identical Mac Pro5.1 with 2 2.4 GHz Quad-Core Intel Xeon processors. Each of has 6GB of memory and total of 8 cores. There are also two iMac9.1 with 3.06 GHz Intel Core 2 Duo processors. One of these is equipped with 4GB of memory while the other one has 6GB of memory and both of them have 2 cores. All of our Macintosh computers have dual booth capacity so that we can run Windows7 and use Windows System compatible software if needed. In addition, we have a Dell OptiPlex 755 Desktop, which has the Linux operating system installed on it.

Matlab2016 (Natick, MA) is available to perform various analyses. LCModel version 6.2.2 is installed on the Dell OptiPlex Desktop to perform proton spectroscopy analysis and to determine the measured metabolite levels. SPSS (to perform statistical analysis), Microsoft Office Applications as well as Adobe Application (to write our reports and papers and to prepare illustrations), EndNoteX8 (to manage references) are available for our researchers. We also have free image analysis software such as FreeSurfer (Martinos Center for Biomedical Imaging, Charles Town, MA), FSL (FMRIB, Oxford, UK) and SPM (Welcome Trust Centre for NeuroImaging, London, UK) to perform the anatomical neuroimaging analysis.

Advanced MRI Center (AMRIC):

- **Neuroimaging data will be acquired at AMRIC.**

The AMRIC facilitates a new Philips Ingenia CX dStream 3.0T system with a higher order shim function which enables us to obtain improved image quality in field-sensitive applications and techniques such as single-voxel spectroscopy, chemical shift imaging, single-shot EPI and balanced FFE.

This Ingenia 3.0T system has a higher order shim function which offers advanced shimming capabilities to obtain improved image quality in field-sensitive applications and techniques such as single-voxel spectroscopy, chemical shift imaging, single-shot EPI and balanced FFE.

This Ingenia 3.0T system features high performance whole body, non-resonant, self-shielded gradient technology with new amplifiers that deliver high peak and slew rates for the demanding requirements of the latest and emerging clinical imaging techniques. The Quasar Dual gradient system provides industry leading performance specifications for peak strength and slew rate with a dual mode capability that optimizes advanced applications requiring very high peak mode capabilities. The maximum gradient amplitudes and slew rates corresponding to the dual mode are 80 mT/m, 100 mT/m/ms and 40 mT/m, 200 mT/m/ms respectively.

This Ingenia 3.0T system has a multiple RF sources, which adapts the RF signals to suit each individual patient. This results in faster scans, enhanced image uniformity/consistency, over a broader range of applications.

This Ingenia 3.0T system features MultiBand SENSE which allows to use state-of-the-art acceleration factors in the brain by simultaneously exciting multiple slices. Due to a shorter minimum TR for fMRI, larger anatomical coverage or higher temporal resolution can be used. In the DWI/DTI sequences larger anatomical coverage or higher number of diffusion directions can be acquired. With MultiBand SENSE, fMRI and DTI exams can be performed with high speed and high resolution, simultaneously.

This Ingenia 3.0T system is equipped with a Multi-nuclear spectroscopy (MNS) system, which provide the ability to perform 13C, 31P, 7Li, 23Na, 19F and other nuclei spectroscopy and imaging. The multiple RF amplifiers in this system includes two 18 kW solid-state 1H channel narrowband amplifier and one 4 kW broadband (10-130 MHz) Multi-nuclear amplifier.

This Ingenia 3.0T system has a bore diameter of 60 cm and provides a full-size 50 cm field-of-view.

fMRI Stimulus Delivery System

A fully integrated fMRI stimulus delivery system from MRA (Model: fMRI-0502-STD1; MRA; Washington, PA, USA; http://www.mra1.com/) and Presentation (Neurobehavioral Systems, Inc; Albany, CA, USA; http://www.neurobs.com/) is available for both clinical and research. The MRA complete system includes the fMRI stimulus delivery console, Windows computer system, patient response hand switches, video projection into the MRI bore, and MRI compatible patient headphones.

Presentation is the world's most popular experimental control software for neuroscience, is a stimulus delivery and experimental control program. It runs on PC, and delivers auditory, visual and multimodal stimuli with sub-millisecond temporal precision. Presentation is powerful enough to handle almost any behavioral, psychological or physiological experiment using fMRI, ERP, MEG, psychophysics, eye movements, single neuron recording, reaction time measures, other performance measures, and more.

MRI Compatible Goggle Set

MediGoggle Adult Research Set (Cambridge Research Systems Ltd, England; http://www.crsltd.com/) has interchangeable prescriptive goggles suitable for use in MRI and fMRI environments. It is fully MRI compatible with no metallic components and an easy 'click' lens system -6 to +6 dioptre lens sets in 0.5 dioptre increments.

In Vivo Physiological monitoring system

Medrad Veris 8600 MR Vital Signs Monitor (Model: 8600; S/N: 023426; Medrad, Inc; Warrendale, PA, USA; http://www.medrad.com/) is available to monitor patients while they are undergoing an MR exam. It interprets and displays physiologic data as waveforms and numeric information which include ECG, NIBP, SpO2, CO2, respiration, temperature, O2, anesthetic gases, and IBP.

The Advanced MRI Center also includes a nurses' station, two patient holding rooms, and two patient changing rooms with lockers.

- *Recruitment sites:*

The Subjects will be recruited through the Brown University study.

23. RESOURCES AVAILABLE

All staff are required initially and every 3 years to successfully complete a UMass - designated CITI program. All participating personnel will thoroughly review the study protocol and will review their roles and responsibilities with experienced study personnel and the Principal Investigator. Personnel are notified of protocol changes, and questions/discussion points are addressed at weekly lab meetings.

Below are the Roles, Responsibilities, and Training for the research staff involved in this study:

**Role**	**Responsibilities**	**Training**
Principal Investigator	Overseeing and managing all activities related to this protocol, including recruitment, data collection, analysis and interpretations. This also includes manuscript preparation and presenting data at conferences. The PI will oversee all IRB	Holds a graduate-level degree (PhD, MD, RN, PsyD, or equivalent) and has had 5 or more years of experience in the field of

	communications.	imaging in psychiatry.
Data Analyst	Collection, analysis and interpretation of data, including neuroimaging data and combining the neuroimaging data with clinical data. Participates in manuscript preparation and data presentation at conferences.	Formally trained in neuroimaging data collection, analysis.
Study Coordinator:	Assists with maintenance of regulatory documents, IRB communications, assists with subject recruitment. Administer telephone screen, consent subjects, coordinate visit dates/times with subjects, arrange subject compensation, measure height/weight, administer mood and psychological assessments, as well as other scales/questionnaires, administer neuropsychological/neurocognitive assessments, manage subject documentation. Participates in manuscript preparation.	Thoroughly review protocol, consenting process, and relevant study procedures with Principal Investigator and/or co-Investigators.

24. LOCAL RECRUITMENT METHODS

Dr. Eric Loucks at Brown University and his team have worked on several mindfulness projects. For the current MB-BP project, they have successfully recruited over 40 participants in a previous enrollment cycle for the MB-BP study, and they anticipate recruiting as many in each of the upcoming cycles, from which will be identified potential participants in the present MRI study. Recruitment will occur by Dr. Louck's team informing participants that we are actively looking for subjects who fit our research criteria, of the opportunity to participate in clinical research, and if the candidates are interested, ask them if they accept our study staff to approach them. Interested candidates will undergo a phone screen to determine their eligibility. If they appear eligible, they will be invited to participate in the study.

Describe the amount, method, and timing of any payments to subjects.

Participants will be compensated $100 per imaging visit, plus $50 in travel expenses (participants will receive free parking). Thus, a total of $300 per participant for baseline and follow-up visit.

25. LOCAL NUMBER OF SUBJECTS

60 participants enrolled in total. As the participants will be recruited from an ongoing study of persons already screened, typical screen failures will be reduced to those individuals failing to satisfy the MRI requirements, which a fraction of usual screen failures. Nevertheless, we anticipate that more than 100 participants may need screening from the Brown University project.

26. CONFIDENTIALITY

All data/documents will be stored in locked cabinets and/or on password protected computers, accessible only by study staff. Data/documents will be stored by subject ID number to avoid linkage of data to identity. Subject number and corresponding identifying information will be kept in separate locked file cabinets, accessible only by study staff. All data/documents will be stored indefinitely.

Approved study personnel are responsible for receipt or transmission of data/documents and specimens locally. Imaging data will be transferred from the AMRIC MRI system by means of a portable drive. All portable drives used to transfer data are encrypted in accordance with UMMS data security policies. Once the transfer is completed, the portable drive will be formatted to clean it of the acquired data. Data will be stored on a password-protected computer indefinitely, accessible only by study staff. Data will also be pushed to servers within the UMASS systems, this server is accessible only by the authorized personnel and it is backed up regularly to password-protected drives.

For the sake of transparency in our research practices, according to recommended practices the de-identified neuroimaging, physiological, task, questionnaire, and participant data will be made available to the public via a web repository (e.g. Open Science Framework). Only researchers who apply and get permission to use the information for a specific research project will be able to access the information. Qualified researchers who can access the national databases can be from government, academic, or commercial institutions.

27. PROVISIONS TO PROTECT THE PRIVACY INTERESTS OF SUBJECTS

Subjects interact with and provide information to approved study staff only. Subjects are informed that study information will be kept strictly confidential, and that study staff will do everything that they can to prevent others from learning about the subject's participation. As frequently as possible, study procedures take place in rooms with closable doors, with a minimum number of participating staff.

For all study procedures, subjects are informed that study related information will be kept strictly confidential, and that study staff will do everything that they can to prevent others from learning about the subject's participation. Subjects are informed that their participation is entirely voluntary, and that they may stop and ask questions, or choose to not take part in/quit the study at any time.

All information acquired through research procedures is stored in locked cabinets and/or on password protected computers.

28. Compensation for Research-Related Injury

No funds have been set aside for research-related injury compensation. In the unlikely event of injury, subjects or their insurance are responsible for coverage.

29. Economic Burden to Subjects

Not Applicable

30. Consent Process

Participants are expected to be competent to give consent for themselves; no vulnerable populations are to take part in this study.

The consenting process will take place in the CCNI or AMRIC facilities. Consent will be obtained in person by Dr. King or a research coordinator, or a trained member of personnel. In order to obtain consent, the study protocol, purpose, risks, and benefits will be explained to the subject. Subjects will be asked to indicate their understanding of the key elements of the protocol including the purpose, risks, and benefits in order to determine that the participant understands the elements required for informed consent. We will only obtain consent if these direct questions have been answered appropriately.

The subject will have the option of thinking about participation for as long as they would like. This includes the option to take the informed consent form home and re-schedule the consent visit for a later date, if that would be helpful to the subject. The subject will also be informed that they can contact Dr. King or the research coordinator at any time if they have a question about the consent form and/or study procedure.

We will be following SOP: Informed Consent Process for Research (HRP-802).

31. Process to Document Consent in Writing

We will be following SOP: Written Documentation of Consent (HRP-803)

32. Drugs or Devices

We are not testing any devices for safety or effectiveness

Appendix 6B - Phone Screener for UMass Medical School fMRI sub-study - to be administered by UMass research staff

Mindful Self-Regulation for blood pressure MRI study – PHONE SCREEN

Date: ________________ Time: _________ Interviewer: ____________________________

Screening # ___________________________ (month.date.year.hour.minute)

My name is __________________. I am calling from the Center for Comparative NeuroImaging at the UMass Medical School, because you expressed interest in participating in our brain imaging study through your participation in the Brown University Mindfulness-Based Blood Pressure Reduction Study, formerly known as Mindfulness-Based Hypertension Therapy. Is that correct?
[If yes, proceed, if no, note in Potential Subject Log].

In order to determine if you are a good match for this particular study, I will be asking you some questions about your health and history which will take from 10 to 15 minutes. Is now a good time to speak? [If yes, proceed, if no, ask for other times and note in Potential Subject Log].

There are a few things that I'd like to make clear before we start the interview. First of all, some of the questions will be very personal and sensitive. Are you in a private place to talk? Because this interview is of a personal nature, it is important that you understand that everything you say will be kept strictly confidential. No one outside of our project will ever be able to see your answers, and we will not keep your name in the same place as any of your answers. Your answers to our brief questions about marijuana and other "recreational" drug use will also be kept strictly confidential and will not be reported.

I am asking these questions because you are participating in the Mindfulness for Blood Pressure Intervention Study at Brown University, we would we would like you to participate in a study where we will image your brain 2 times, before and after the intervention. Everything you say in this interview will be kept strictly confidential, and you may stop or ask questions at any time. If you don't understand what I am asking, please stop me and ask me to explain. If you are eligible for the study we will ask for your name once we finish with this interview, otherwise we will not ask for your name. If you like, though, we can keep your information on file for future studies.

These phone screens are kept in a ring binder in a secure location. They will not be destroyed. Are you comfortable with starting the interview now?

**INCLUSION CRITERIA: Need YES to all Yes/No questions**		
1. How old are you?______ D.O.B. ________ Between 18 and 65?	Yes ___	No ___
2. Are you participating in Brown University Mindfulness for Blood Pressure Intervention Study?	Yes ___	No ___

Additional information		
3. Are you male or female or identify as male or female?	Male ___	Female: ___
4. Are you right or left handed?	Right: ___	Left: ___
5. Current occupation?		
6. Are you currently diagnosed with Attention Deficit Hyperactivity Disorder?<br>• When were you diagnosed? ________________<br>• If diagnosis was age 7+: How young can you recall symptoms and	Yes ___	No ___

what kind of issues did you experience?		
7. Are you currently taking medication(s) for ADHD?<br>• Drug:__________Dose?______ Freq:_________<br>• Since when:________________	Yes ___	No ___
8. Have you taken medications for ADHD before?<br>• Drug(s): ______________________________<br>• Dose(s): ____________Freq(s):_____________<br>• When/How long: ________________________	Yes ___	No ___
9. Do you currently smoke tobacco cigarettes?<br>• If yes, about how many cigarettes a day/week? __________<br>• How long have you smoked?__________	Yes: ___	No: ___
10. Do you currently smoke marijuana?<br>• If yes, how many times per day/week do you smoke? _________<br>• How long have you smoked marijuana?_________	Yes: ___	No: ___
11. Do you currently use any other recreational drugs?<br>• If yes, what? How often? For how long?	Yes: ___	No: ___

**Medical History**		
12. Have you ever had a neurological problem? (such as a seizure disorder, epilepsy, migraines or multiple sclerosis)<br>• Describe _____________________-<br>• Any medications/treaments?(name, dose, frequency, how long?)	Yes: ___	No: ___
13. Have you ever had a head trauma or lost consciousness?<br>Describe (eg. when, severity, diagnosed and by whom, how long the loss of consciousness):	Yes: ___	No: ___
14. Are you currently on any medications?<br>Drug: ___________ Dose:________ Reason:<br><br>Drug: ___________ Dose:________ Reason:	Yes: ___	No: ___
15. Have you ever brain surgery or had a stroke? (Condition associated with structural brain alteration on MRI)	Yes: ___	No: ___
16. Do you experience claustrophobia?	Yes: ___	No: ___
17. Have you ever had an operation or other surgical procedure?<br>• If yes, which types? When?	Yes: ___	No: ___
18. [If applicable] Are you pregnant?	Yes: ___	No: ___

**Psychiatric History**		
19. Do you have a serious psychiatric, cognitive or medical disorder which could interfere with completion of the study?	Yes: ___	No: ___
• Do you have a current or past diagnosis with any of these psychological disorders?<br>• Schizophrenia or schizoaffective disorder<br>• Autism or other Pervasive Developmental Disorder<br>• Obsessive-Compulsive Disorder<br>• Claustrophobia<br>• Alcohol or substance abuse or dependence (past six months). (>14/week or >4 drinks at any one time for a male, or >7 drinks/week or >3 drinks at any one time for a female) or substance abuse) ?<br>*(above are exclusionary conditions)*<br>• Anxiety/Depression<br>• Other ______________<br>▪ Details:<br>• Are you currently taking medications for any psychological problems?<br>Drug: ___________ Dose: ________ Freq: ___________<br>Duration: _________________________	_____<br>_____<br>_____<br>_____<br>_____<br>_____<br><br>_____<br>_____	_____<br>_____<br>_____<br>_____<br>_____<br>_____<br><br>_____<br>_____
20. Have you ever been hospitalized for psychosis or suicidal thoughts?<br>When? _____________ How long? ________	Yes ___	No ___

**MRI and Safety**		
21. Do you have any metal in/on your body that cannot be removed? E.g.<br>☐ surgical implants<br>☐ pins<br>☐ pacemakers<br>☐ prosthetic heart valve<br>☐ aneurism clips or other vascular stent, filters, or other devices<br>☐ surgical clips<br>☐ staples<br>☐ neuro-stimulator devices<br>☐ implanted infusion pumps<br>☐ cochlear (ear) implants<br>☐ Ocular (eye) implants or metal fragments in eyes<br>☐ other prostheses<br>☐ other: _________ Describe: ______________________________	Yes: ___	No: ___
22. Do you have braces or any other metal which may be potentially magnetic?<br>• This includes braces, false teeth, retainer, special hair dye, etc.	Yes: ___	No: ___
23. Do you have any tattoos?<br>• If yes, is there any chance that it/they contain metallic pigments?	Yes: ___	No: ___
24. Have you ever been exposed to shrapnel or metal filings (e.g. employed as a machinist, metal worker, welder, or other?	Yes: ___	No: ___
25. Is there any chance that you have ever been hit with any stray metal (BB's, metal shavings, etc)	Yes: ___	No: ___
26. Any condition known to be incompatible with MRI scan	Yes: ___	No: ___
27. Have you ever had an MRI or other scan?<br>• If yes, were there any difficulties?	Yes: ___	No: ___
28. Are you over 300 lbs? (weight______)	Yes: ___	No: ___

☐ At conclusion of the pre-screen, enter name and screening # in screen log

**If the candidate is NOT eligible for the current study:**<br>"I'm sorry, but you are not eligible for our study. if we have another study in the future that you might be eligible for, would you like to be contacted for that?"	Yes ___	No ___

If the candidate appears to be eligible for the current study, and is interested:

Phone #'s: ________________ _________________	Ok to leave a message? Yes____ No____
Email address: ______________________________	
Preferred method of contact? Phone/email (circle one)	
Scheduling considerations:	

☐ separate pages 1-4 from page 5, add the subject's study ID number to the top of pages 1-4 and store in locked file separate from page 5 and the screen log.

Investigator Signature: _______________________________ __ __ / __ __ / __ __ __ __
d d m m y y y y

Pre-Screen

Mindful Self-Regulation for blood pressure MRI study		
**Name:**		
**Email:**		**Screening Date:**
**Phone**	**Phone**	___ ___ / ___ ___ / ___ ___ ___ ___<br>d d m m y y y y

Appendix 6C - Informed Consent form for UMass Medical School fMRI sub-study to be administered by UMass research staff

UNIVERSITY OF MASSACHUSETTS MEDICAL SCHOOL

COMMITTEE FOR THE PROTECTION OF HUMAN SUBJECTS IN RESEARCH

CONSENT TO PARTICIPATE IN A RESEARCH STUDY

Title of research study:

Mindfulness Influences on Self-Regulation:

Mental and Physical Health Implications for blood pressure

(Mindfulness MB-BP Self-Regulation MRI)

Investigator: Jean King, Ph.D.

Sponsor: The National Institutes of Health

Why are you being invited to take part in a research study?

You are being asked to participate because you are between 18 and 65 years of age and participating in the Mindfulness-Based Blood Pressure intervention study, have thus met entrance criteria for having prehypertension or hypertension, or another cardiovascular risk factor that could be influenced by this program, and do not have any other active medical or psychiatric illness, and are able to have MRI scanning done.

What should you know about a research study?

Your participation is entirely voluntary.

You may decide not to take part or decide to quit the study at any time, without any changes in the quality of the health care you receive.

You will be told about any new information or changes in the study that might affect your willingness to participate.

You are free to ask all the questions you want before deciding if you want to be in this study.

Why are we doing this research?

The purpose of the study is to begin to find out if a mindfulness-based blood pressure intervention affects brain function and whether any alterations are associated with mechanisms of self-regulation that accompany participation in such a program. We hope that such relationships exist, so that we can better understand the effect of mindfulness on self-regulation mental and behavioral changes that could impact blood pressure regulation. This knowledge may help researchers hone in on effective mechanisms, and healthcare providers to improve the care of people with prehypertension, hypertension, or other related cardiovascular risk factors.

Formatted: Font: 10 pt
Formatted: Font: 10 pt
Formatted: Font: 10 pt
Formatted: Font: 10 pt
Formatted: Font: 10 pt
Formatted: Font: 10 pt

How long will the research last?

We expect that you will be in this research study for 2 Visits, which should take approximately 4.5 hours in total to complete.

How many people will be studied?

We expect about 60 people to be in this portion of the research study at UMass Medical School.

~~1~~ *What happens if I say yes, I want to be in this research?*

Formatted: Indent: Left: 0", Hanging: 0.25", No bullets or numbering

If you want to participate in this research, you will be asked to come in for a total of two visits – before and after the Mindfulness-Based Blood Pressure intervention (Visits 1 and 2). Each visit includes an MRI scan, and should take about 2 hours total to complete.

Magnetic Resonance Imaging (MRI) **~1.5 hours**:

This study includes two magnetic resonance imaging (MRI) scans. An MRI is a technique for taking images of the brain. It uses a magnetic field and radio waves - no ionizing radiation is used. We will take you to the MRI imaging suite at the University of Massachusetts Medical School's main campus and ask you a series of safety questions before we bring you into the MRI room. Some of these questions might be repetitive but is important that we make sure it is safe for you to have an MRI scan.

Because the scanner contains an extremely strong magnet you will be asked to remove all metal objects on you. This includes things like: watches, rings, necklaces, bracelets, earrings and other body piercings, belts, loose change, wallet (with credit cards), items of clothing containing magnetic materials (for example, under wire bras, certain types of zippers), and shoes. We will ask you change into a hospital gown and pants ("scrubs") before you go into the scanning room. Your clothing and personal items will be locked up in a safe place until the session is completed.

You will spend 90 minutes in an MRI scanner, which looks like a large cylinder with a tube running down the center. You will be asked to lie down on your back on a foam-padded table and to put your head into a special holder. The table slides inside the "hole" of the scanner. Soft foam rubber sponges may be placed on both sides of your head for comfort and to keep your head still. A headset with microphone allows you to hear and talk to the technician operating the scanner at all times.

During the scanning procedure, you will hear a number of different sounds. These sounds, which can be loud, are part of the normal operation of the scanner. These noises vary with the particular scan being performed, and include sounds like a hammer hitting a piece of wood, repetitive buzzing noises, and long series of loud beeps. Some scans are silent. These sounds, or combinations of them, will then be repeated several times, depending upon the specific scan sequence being used. The sounds you hear during the scanning session will not harm your hearing, but you will be given a pair of earplugs to wear for your comfort. Even with these earplugs in you will always be able hear the technician because they do not block all sound. You are free to talk during the preparation time and during the breaks, but you should not talk during the actual scanning process. During these sessions, you should try to remain as still as possible.

Formatted: Font: 10 pt
Formatted: Font: 10 pt
Formatted: Font: 10 pt
Formatted: Font: 10 pt
Formatted: Font: 10 pt
Formatted: Font: 10 pt

The entire time that you will be in the scanner will be no longer than 90 minutes. When the session is over the technician will move you out of the scanner and assist you from the table. At this time, you will be asked a series of questions from a questionnaire that asks your experiences while in the MRI scanner (Amsterdam Resting-State Questionnaire).– This survey will take approximately 5 minutes to complete.

The MRI scanner takes moment-to-moment images of your brain's blood-flow and tissue. These images are digitally stored. Thousands of patients across the country safely undergo such procedures in radiology departments every day. The MRI procedure itself is not experimental; rather, it is well known to be safe.

What are the risks of being in this study?

Magnetic resonance (MR) technology does not use ionizing radiation like an X-ray. Instead, it uses strong magnetic fields and radio-frequency waves to collect the images and data. There are no known serious health hazards or risks associated with these techniques for most people. However, significant risks may exist for people with:

- Cardiac pacemakers
- Aneurysm clips (in your brain) and other vascular stents (tubes in your veins), filters, clips or other devices that have been surgically put in you for any reason
- Prosthetic heart valves (a mechanical device that helps your heart pump blood)
- Other prostheses (a piece of equipment that replaces a missing part of the body)
- Neuro-stimulator devices (a piece of equipment that activates nerves)
- Implanted infusion pumps (a device that stays in your body and helps give you medicine)
- Cochlear (ear) implants (a device used to help you hear)
- Ocular (eye) implants (a device put in your eye to help you see)
- Known metal fragments or pieces in eyes
- Contact with shrapnel (pieces of metal from something that has exploded)
- Contact with metal filings (people who are sheet metal workers, welders, or others could have this contact)
- Any surgeries where something metal was put in you
- Certain tattoos (please tell the study doctor if you have a tattoo so that we can make sure it is safe)
- Certain intrauterine contraception devices (IUD's)

You will be asked whether you have such devices and if so, you will not be able to participate in this study. Significant risks also can arise if certain types of metal objects are brought into the scanning area, as they can become hazardous projectiles. These types of items are not permitted in the scanning area. The exams are painless, and except for the pulsating sounds, you will not be aware that MR scanning is taking place.

This study will be conducted in an MR scanner which has been approved by the FDA for clinical and research studies. Although there are no known significant health risks from these scans, there could be adverse effects that are delayed or very mild, such that they have not yet been recognized. Most people experience no ill effects from the large magnetic field, but some people

Formatted: Font: 10 pt
Formatted: Font: 10 pt
Formatted: Font: 10 pt
Formatted: Font: 10 pt
Formatted: Font: 10 pt
Formatted: Font: 10 pt

do report claustrophobia (fear of being in small spaces), dizziness, mild nausea, headaches, a metallic taste in their mouth, double vision or sensation of flashing lights. These symptoms, if present, disappear shortly after leaving the MR machine. If you experience discomfort from being in the scanner, you should notify the examiner immediately and you will be removed from the scanner. You should also note you will be asked to remain as still as possible during the scanning session. Remaining motionless can result in physical discomfort.

You may feel cramped inside the scanner. The technologist will be able to hear you at all times and you are free to end the procedure at any time.

In rare cases, a very slight, uncomfortable tingling of the back due to the rapid switching of the magnetic field has been reported during certain types of scans. In case you have such a sensation, you are asked to report this immediately, so the scan can be changed to avoid this. Although these precautions will avoid all known risks associated with MR, this procedure may involve risks to you that are currently unforeseeable.

A qualified neuroradiologist will interpret your brain scan, as you are having images taken of your brain tissue and brain blood flow. We will retain your contact information in the event that it is medically important to tell you of any aspect of your brain scan. Any medically important findings on the MRI will be identified and reported to you. This process could also produce some discomfort, and would only be done if believed in your medical best interests. Please note that you will not be billed for the procedure, and that we will also help you get any follow-up care you might need.

Another -risk of being in this study is a loss of your personal information. This is very unlikely to happen, and we will do everything to make sure that your information is protected.

What are my responsibilities if I take part in this research?

If you take part in the research, it is important for your safety that you:

- Follow the directions of the study doctor and research staff.
- Tell your health care providers that you are in a research study.
- Tell your study doctor and staff about all medications you are taking (prescription and over the counter) and all of your health issues.
- Call the study doctor or staff at 774-455-4272 if you have any questions.

Will being in this study help me in any way?

There may be no benefit to being in this study. However, knowledge gained from this study may help others with your condition in the future.

Will being in this study cost me any money?

Participating in this study will NOT require a payment, co-pay, or insurance charge. There will be no cost to you from being in this research study other than the cost of your transportation to UMass Medical School.

Formatted: Font: 10 pt
Formatted: Font: 10 pt
Formatted: Font: 10 pt
Formatted: Font: 10 pt
Formatted: Font: 10 pt
Formatted: Font: 10 pt

What happens to information about me?

Efforts will be made to limit access to your personal information, including research study and medical records, to people who have a need to review this information. We cannot promise complete secrecy. The UMMS Institutional Review Board (the committee that reviews, approves, and monitors research on human subjects) and other representatives of UMMS may need to review records of individual subjects. As a result, they may see your name, but they are bound by rules of confidentiality not to reveal your identity to others. Your identity will remain confidential in any study results that are made public.

We will communicate the ~~reults~~ results of this study to others by publishing the reults in scholarly books or journals, presenting them at professional conferences, or using them for teaching purposes. In addition, for transparency in our research practices and according to current recommendations, we will make available the de-identified data ~~-~~(including your and other participants'imaging, physiological, questionnaire, behavioral), ~~we collect from you nd and other participants accessible to~~ through a public bank~~the public~~ ~~vis~~ ~~via~~ or a web repository such as through the Open Science Framework. This means that only researchers who apply and get permission to use the information for a specific research project will be able to access the information. Qualified researchers who can access the national databases can be from government, academic or commercial institutions. This will enable interested parties, like other researchers, to reproduce our results and use the acquired data in other analyses. All of your information will be kept in strict confidence; you will never be personally identified in any communication or publicly-accessible data sets.”

What happens if I am injured because I took part in this research?

If you are injured while on study, seek treatment and contact the study doctor as soon as you are able.

The University of Massachusetts Medical School does not provide funds for the treatment of research-related injury. If you are injured as a result of your participation in this study, treatment will be provided. You or your insurance carrier will be expected to pay the costs of this treatment. No additional financial compensation for injury or lost wages is available.

You do not give up any of your legal rights by signing this form.

Will I be given any money or other compensation for being in this study?

For ~~both~~ your first visit~~s~~ ~~involving MRI's~~ you will receive $10~~5~~0 ~~each~~ for completing both the MRI and the questionnaires, and $50 in travel expenses (free parking), ~~-~~even if you choose to discontinue your participation due to any discomfort you might experience during the scanning session. If your first visit is successful, you will be asked to return for a second visit, with the same compensation scheme, for a possible total of $300.

What happens if I do not want to be in this research?

Formatted: Font: Times New Roman, 12 pt
Formatted: Font: Times New Roman, 12 pt
Formatted: Font: Times New Roman, 12 pt
Formatted: Font: Times New Roman, 12 pt
Formatted: Font: Times New Roman, 12 pt
Formatted: Font: Times New Roman, 12 pt
Formatted: Font: Times New Roman, 12 pt
Formatted: Font: 10 pt
Formatted: Font: 10 pt
Formatted: Font: 10 pt
Formatted: Font: 10 pt
Formatted: Font: 10 pt
Formatted: Font: 10 pt

If you decide not to take part in the research, it will not affect your usual care and it will not be held against you.

What happens if I say yes, but I change my mind later?

You are free to leave the study at any time. There are no penalties and you do not lose any benefits to which you are otherwise entitled. Data that has already been used will remain part of the study database and may not be removed in order to maintain the integrity of the research However, any identifiable information will be destroyed so that no one can tell the data belonged to you. If you decide to stop your participation, we may ask if you are willing to have us contact you for safety follow-up purposes or to provide further data collection from routine medical care.

There are no risks to dropping out of the study.

Can I be removed from the research without my OK?

The person in charge of the research study or the sponsor can remove you from the research study without your approval. Possible reasons for removal include inability to comply with study procedures.

Who can I talk to?

If you have questions, concerns, or complaints, or think the research has hurt you, you can talk to ~~the~~ Dr. Jean King, PhD's research team at 774-455-4272 or Dr. Eric Loucks' research team at 401-400-4768~~401-XXX-XXXX~~.

Formatted: Font: (Default) Times New Roman, (none)

This research has been reviewed and approved by an Institutional Review Board. You may talk to them at (508) 856-4261 or irb@umassmed.edu for any of the following:

- Your questions, concerns, or complaints are not being answered by the research team.
- You cannot reach the research team.
- You want to talk to someone besides the research team.
- You have questions about your rights as a research subject.
- You want to get information or provide input about this research.

Formatted: Font: 10 pt
Formatted: Font: 10 pt
Formatted: Font: 10 pt
Formatted: Font: 10 pt
Formatted: Font: 10 pt
Formatted: Font: 10 pt

Signature Block for Capable Adult

Your signature documents your permission to take part in this research.

Signature of subject	Date

Printed name of subject

Signature of person obtaining consent	Date

Printed name of person obtaining consent

Formatted: Font: 10 pt

Formatted: Font: 10 pt

Formatted: Font: 10 pt

Formatted: Font: 10 pt

Formatted: Font: 10 pt

Formatted: Font: 10 pt

Appendix 6D -
Questionnaire for UMass Medical School fMRI sub-study

Amsterdam Resting-State Questionnaire 2.0

Below is a collection of statements about your experience in the scanner. Using the responses below, please indicate how you felt. Please answer according to what really reflects your experience rather than what you think your experience should be.

Version 1.A

	**Completely disagree**	**Somewhat disagree**	**Neither agree or disagree**	**Somewhat agree**	**Completely agree**
I imagined talking to myself.					
I pictured events.					
I felt relaxed.					
I thought about people I like.					
I thought about my heartbeat.					
I felt pain.					
I had difficulty staying awake.					
I thought about myself.					
I had difficulty holding on to my thoughts.					
I felt comfortable.					
I thought about the future.					
I thought in images.					
I thought in words.					
I felt tired.					
I was conscious of my body.					
I thought about my health.					
I thought about solving problems.					
I had busy thoughts.					
I felt ill.					
I thought about others.					
I felt sleepy.					
I felt happy.					
I thought about my feelings.					
I pictured places.					
I thought about things I need to do.					
I placed myself in other people's shoes.					
I had rapidly switching thoughts.					
I thought about my breathing.					
I had silent conversations.					
I thought about my behavior.					
I had my eyes closed					
I was able to rate the statements					

Amsterdam Resting-State Questionnaire 2.0

Version 1.B

	**Completely disagree**	**Somewhat disagree**	**Neither agree or disagree**	**Somewhat agree**	**Completely agree**
I thought about my heartbeat.					
I thought about solving problems.					
I felt sleepy.					
I had difficulty holding on to my thoughts.					
I thought about people I like.					
I had rapidly switching thoughts.					
I pictured places.					
I thought about my behavior.					
I had difficulty staying awake.					
I was conscious of my body.					
I had silent conversations.					
I thought about others.					
I had busy thoughts.					
I thought about the future.					
I felt comfortable.					
I thought about things I need to do.					
I felt tired.					
I thought about my health.					
I thought about my feelings.					
I placed myself in other people's shoes.					
I felt pain.					
I thought in words.					
I thought in images.					
I pictured events.					
I felt relaxed.					
I thought about my breathing.					
I thought about myself.					
I imagined talking to myself.					
I felt happy.					
I felt ill.					
I had my eyes closed					
I was able to rate the statements					

Appendix 6E - UMass Medical School fMRI sub-study IRB Approval Letter

University of Massachusetts
UMASS Medical School

12/12/2017

Jean King, PhD
University of Massachusetts
Psychiatry

Dear Dr. King:

The IRB reviewed the following:

Type of Submission:	Study
Review Type:	Non-Committee
Project Title:	Mindfulness Influences on Self-Regulation:<br>Mental and Physical Health Implications for blood pressure<br>(Mindfulness MB-BP Self-Regulation MRI)
Investigator:	Jean King, PhD
IRB ID:	H00014090
Funding Agency:	NATIONAL INSTITUTES OF HEALTH
Grant Title:	MINDFULNESS INFLUENCES ON SELF-REGULATION: MENTAL AND PHYSICAL HEALTH IMPLICATIONS
Grant ID:	3UH2AT009145-03S1
IND or IDE:	None
IRB Review Date:	12/3/2017
Documents Reviewed:	umms-hrp-504---ISP MB-BP MRI-2017.12.12.docx<br>Amsterdam Resting-State Questionnaire 2017.11.06.docx<br>Phone Screen MB-BP MRI H00014090 2017.12.05.doc<br>Consent_MB-BP MRI 2017.12.05.doc

The IRB approved the research from 12/12/2017 to 12/11/2018 inclusive. Before 10/27/2018 or within 30 days of closing the study, whichever is earlier, you are required to submit a completed Continuing Review Progress Report and necessary attachments to request continuing approval or study closure.

If continuing review approval is not granted before the expiration date of 12/11/2018, approval of this research expires on that date.

Stamped consent documents are included with this approval. Use these to document consent.

In conducting this research, you are required to follow the requirements listed in the INVESTIGATOR MANUAL.

Sincerely,

Crystal Davis, MPH
IRB Protocol Specialist

cc: Payne, Laurellee

Research Protections Office
Box 1986
Providence, RI 02912
Tel: (401) 863-3050
Fax: (401) 863-7292

Memorandum

**To:**	Eric Loucks, Box G-S121-2
**From:**	Research Protections Office
**Date:**	March 12, 2015
**RE:**	Protocol Entitled: Mindfulness-Based Hypertension Therapy Pilot Study (#1412001171).

The above referenced protocol received a full board review and approval by the Brown University IRB on January 15, 2015. IRB approval is valid for 1 year from January 15, 2015 through January 15, 2016.

Should the research continue, the next progress report must be submitted two months prior to the expiration date for review and approval.

Any amendments or changes to the research protocol including changes to the consent documents and questionnaires/surveys must be submitted to the IRB for review and approval prior to implementation. Also, any change in the status of participants to any of the vulnerable populations (as identified in 45 CFR 46) including, pregnant women, prisoners, or those with diminished capacity, requires IRB review and approval prior to their continuing in the study.

The IRB anticipates that investigators will employ recruitment procedures that allow for the equitable recruitment of women and minorities into research studies.

Note: All research staff must successfully complete the Brown University Education Program in the Protection of Human Research Participants (CITI at http://www.citiprogram.org) prior to beginning work on the project.

*** For Your Information ***

The next deadline for submission of new protocols, amendments to protocols, and annual progress reports requiring full board review is March 31, 2015.

All pertinent federal and University policies and guidelines related to the involvement of participants in research can be obtained through the Research Protections Office (RPO) at

http://www.brown.edu/research/institutional-review-board-irb. This includes the *Belmont Report*, 45 CFR 46, and the Brown University Federal Wide Assurance #00004460.

Research Protections Office
Box 1986
Providence, RI 02912
Tel: (401) 863-3050
Fax: (401) 863-7292

Memorandum

To: Eric Loucks, Box G-S121-2

From: Research Protections Office

Date: July 01, 2015

RE: Protocol Entitled: Mindfulness-Based Hypertension Therapy Pilot Study (#1412001171).
Amendment #1

The above amendment to the protocol, received an expedited review and approval by the IRB on July 01, 2015.

This amendment dated 6/17/15, (originally submiited 4/8/15) includes changing the survey software to Qualtrex, updating the phone screening questionnaire, clarifying the study safety plan, modifying the consent form, adding questionnaires to the baseline survey, and modifying recrutiment procedures.

This amendment approval date does not change the continuing review date for the overall project. Should the research continue, the next progress report must be submitted for review and approval two months prior to the expiration date.

Any amendments or changes to the research protocol including changes to the consent documents and questionnaires/surveys must be submitted to the IRB for review and approval prior to implementation. Also, any change in the status of participants to any of the vulnerable populations (as identified in 45 CFR 46) including, pregnant women, prisoners, or those with diminished capacity, requires IRB review and approval prior to their continuing in the study.

The IRB anticipates that investigators will employ recruitment procedures that allow for the equitable recruitment of women and minorities into research studies.

Note: All research staff must successfully complete the Brown University Education Program in the Protection of Human Research Participants (CITI at http://www.citiprogram.org) prior to beginning work on the project.

*** For Your Information ***

The next deadline for submission of new protocols, amendments to protocols, and annual progress reports requiring full board review is July 31, 2015.

All pertinent federal and University policies and guidelines related to the involvement of participants in research can be obtained through the Research Protections Office (RPO) at http://www.brown.edu/research/institutional-review-board-irb. This includes the *Belmont Report*, 45 CFR 46, and the Brown University Federal Wide Assurance #00004460.

Research Protections Office
Box 1986
Providence, RI 02912
Tel: (401) 863-3050
Fax: (401) 863-7292

BROWN

Memorandum

To: Eric Loucks, Box G-S121-2
From: Research Protections Office
Date: August 07, 2015
RE: Protocol Entitled: Mindfulness-Based Hypertension Therapy Pilot Study (#1412001171).
Amendment #2

The above amendment to the protocol, received an expedited review and approval by the IRB on August 06, 2015.

This amendment dated 7/15/15 (revised and re-submitted on 8/5/15) included: The addition of a new participant population (NEFS sub-set) and new recruitment procedures for that population, revisions to the In-person Screening Assessments 1 and 2, revisions to wording of the phone screening questionniare, addition of a place for staff to note contact information on the safety plan document, adding a version footer to the consent document.

This amendment approval date does not change the continuing review date for the overall project. Should the research continue, the next progress report must be submitted for review and approval two months prior to the expiration date.

Any amendments or changes to the research protocol including changes to the consent documents and questionnaires/surveys must be submitted to the IRB for review and approval prior to implementation. Also, any change in the status of participants to any of the vulnerable populations (as identified in 45 CFR 46) including, pregnant women, prisoners, or those with diminished capacity, requires IRB review and approval prior to their continuing in the study.

The IRB anticipates that investigators will employ recruitment procedures that allow for the equitable recruitment of women and minorities into research studies.

Note: All research staff must successfully complete the Brown University Education Program in the Protection of Human Research Participants (CITI at http://www.citiprogram.org) prior to beginning work on the project.

*** For Your Information ***

The next deadline for submission of new protocols, amendments to protocols, and annual progress reports requiring full board review is August 31, 2015.

All pertinent federal and University policies and guidelines related to the involvement of participants in research can be obtained through the Research Protections Office (RPO) at http://www.brown.edu/research/institutional-review-board-irb. This includes the *Belmont Report*, 45 CFR 46, and the Brown University Federal Wide Assurance #00004460.

BROWN

Research Protections Office
Box 1986
Providence, RI 02912
Tel: (401) 863-3050
Fax: (401) 863-7292

Memorandum

To: Eric Loucks, Box G-S121-2

From: Research Protections Office

Date: September 03, 2015

RE: Protocol Entitled: Mindfulness-Based Hypertension Therapy Pilot Study (#1412001171).
Amendment #3

The above amendment to the protocol, received an expedited review and approval by the IRB on September 03, 2015.

This amendment dated 9/1/15 included the addition of a funding source (grant #1 UH2 AT009145-01, grant title: Mindfulness Influences on Self-Regulation: Mental and Physical Health Implications), a change in scope of work and related study activities secondary data analyses involving concurrent studies, additional measures and study procedures, and revised informed consent document.

This amendment approval date does not change the continuing review date for the overall project. Should the research continue, the next progress report must be submitted for review and approval two months prior to the expiration date.

Any amendments or changes to the research protocol including changes to the consent documents and questionnaires/surveys must be submitted to the IRB for review and approval prior to implementation. Also, any change in the status of participants to any of the vulnerable populations (as identified in 45 CFR 46) including, pregnant women, prisoners, or those with diminished capacity, requires IRB review and approval prior to their continuing in the study.

The IRB anticipates that investigators will employ recruitment procedures that allow for the equitable recruitment of women and minorities into research studies.

Note: All research staff must successfully complete the Brown University Education Program in the Protection of Human Research Participants (CITI at http://www.citiprogram.org) prior to beginning work on the project.

*** For Your Information ***

The next deadline for submission of new protocols, amendments to protocols, and annual progress reports requiring full board review is September 30, 2015.

All pertinent federal and University policies and guidelines related to the involvement of participants in research can be obtained through the Research Protections Office (RPO) at http://www.brown.edu/research/institutional-review-board-irb. This includes the *Belmont Report*, 45 CFR 46, and the Brown University Federal Wide Assurance #00004460.

Research Protections Office
Box 1986
Providence, RI 02912
Tel: (401) 863-3050
Fax: (401) 863-7292

Memorandum

To: Eric Loucks, Box G-S121-2
From: Research Protections Office
Date: January 08, 2016
RE: Protocol Entitled: Mindfulness-Based Hypertension Therapy Pilot Study (#1412001171).
Amendment #4

The above amendment to the protocol, received an expedited review and approval by the IRB on January 07, 2016.

This amendment (dated: 11/11/2015) requests approval to (a) add compensation, (b) add a NIH safety monitoring plan, (c) update the names and content of the phone screen and assessments, (d) add a 1-year follow-up visit, and (e) update the consent document to incorporate these changes.

This amendment approval date does not change the continuing review date for the overall project. Should the research continue, the next progress report must be submitted for review and approval two months prior to the expiration date.

Any amendments or changes to the research protocol including changes to the consent documents and questionnaires/surveys must be submitted to the IRB for review and approval prior to implementation. Also, any change in the status of participants to any of the vulnerable populations (as identified in 45 CFR 46) including, pregnant women, prisoners, or those with diminished capacity, requires IRB review and approval prior to their continuing in the study.

The IRB anticipates that investigators will employ recruitment procedures that allow for the equitable recruitment of women and minorities into research studies.

Note: All research staff must successfully complete the Brown University Education Program in the Protection of Human Research Participants (CITI at http://www.citiprogram.org) prior to beginning work on the project.

*** For Your Information ***

The next deadline for submission of new protocols, amendments to protocols, and annual progress reports requiring full board review is February 01, 2016.

All pertinent federal and University policies and guidelines related to the involvement of participants in research can be obtained through the Research Protections Office (RPO) at http://www.brown.edu/research/institutional-review-board-irb. This includes the *Belmont Report*, 45 CFR 46, and the Brown University Federal Wide Assurance #00004460.

BROWN

Research Protections Office
Box 1986
Providence, RI 02912
Tel: (401) 863-3050
Fax: (401) 863-7292

Memorandum

To: Eric Loucks, Box G-S121-2

From: Research Protections Office

Date: February 26, 2016

RE: Protocol Entitled: Mindfulness-Based Hypertension Therapy Pilot Study (#1412001171).
Amendment #5

The above amendment to the protocol, received an expedited review and approval by the IRB on February 26, 2016.

This amendment (dated: 2/26/16) requests approval to allow participants, who are not able to come to the study site in person, the option to participate by online video conferencing.

This amendment approval date does not change the continuing review date for the overall project. Should the research continue, the next progress report must be submitted for review and approval two months prior to the expiration date.

Any amendments or changes to the research protocol including changes to the consent documents and questionnaires/surveys must be submitted to the IRB for review and approval prior to implementation. Also, any change in the status of participants to any of the vulnerable populations (as identified in 45 CFR 46) including, pregnant women, prisoners, or those with diminished capacity, requires IRB review and approval prior to their continuing in the study.

The IRB anticipates that investigators will employ recruitment procedures that allow for the equitable recruitment of women and minorities into research studies.

Note: All research staff must successfully complete the Brown University Education Program in the Protection of Human Research Participants (CITI at http://www.citiprogram.org) prior to beginning work on the project.

*** For Your Information ***

The next deadline for submission of new protocols, amendments to protocols, and annual progress reports requiring full board review is February 29, 2016.

All pertinent federal and University policies and guidelines related to the involvement of participants in research can be obtained through the Research Protections Office (RPO) at http://www.brown.edu/research/institutional-review-board-irb. This includes the *Belmont Report*, 45 CFR 46, and the Brown University Federal Wide Assurance #00004460.

BROWN

Office of Research Integrity
Box 1986
Providence, RI 02912
Tel: (401) 863-3050
Fax: (401) 863-7292

Memorandum

**To:**	Eric Loucks, Box G-S121-2
**From:**	Human Research Protection Program
**Date:**	May 09, 2016
**RE:**	Protocol Entitled: The Mindfulness-Based Blood Pressure Reduction Study (MB-BP) (#1412001171). Amendment #6

The above amendment to the protocol, received an expedited review and approval by the IRB on May 09, 2016.

This amendment (dated: 4/11/2016) requests approval to (a) change the study title; (b) revise the NCCIH Safety Monitoring Protocol; (c) separate the Baseline and Follow-up Assessments into two visits; (d) change the data collection mode to Qualtrics for Daily Practice Forms and Class questionnaires; (e) revise study recruitment material and use online advertising; (f) revise the in-class worksheets and assessments; and (g) revise the MB-BP Questionnaire, In-Person Assessment, Home Assessment, and Measurement of Mindfulness Practices.

This amendment approval date does not change the continuing review date for the overall project. Should the research continue, the next progress report must be submitted for review and approval two months prior to the expiration date.

Any amendments or changes to the research protocol including changes to the consent documents and questionnaires/surveys must be submitted to the IRB for review and approval prior to implementation. Also, any change in the status of participants to any of the vulnerable populations (as identified in 45 CFR 46) including, pregnant women, prisoners, or those with diminished capacity, requires IRB review and approval prior to their continuing in the study.

The IRB anticipates that investigators will employ recruitment procedures that allow for the equitable recruitment of women and minorities into research studies.

Note: All research staff must successfully complete the Brown University Education Program in the Protection of Human Research Participants (CITI at http://www.citiprogram.org) prior to beginning work on the project.

*** For Your Information ***

The next deadline for submission of new protocols, amendments to protocols, and annual progress reports requiring full board review is May 31, 2016.

All pertinent Federal and University policies and guidelines related to the involvement of participants in research can be obtained through the Human Research Protection Program at http://www.brown.edu/research/institutional-review-board-irb. This includes the *Belmont Report*, 45 CFR 46, and the Brown University Federal Wide Assurance #00004460.

Office of Research Integrity
Box 1986
Providence, RI 02912
Tel: (401) 863-3050
Fax: (401) 863-7292

BROWN

Memorandum

To: Eric Loucks, Box G-S121-2

From: Human Research Protection Program

Date: September 13, 2016

RE: Protocol Entitled: The Mindfulness-Based Blood Pressure Reduction Study (MB-BP) (#1412001171).
Amendment #7

The above amendment to the protocol, received an expedited review and approval by the IRB on September 13, 2016.

Approval of the amendment (memo dated August 9, 2016) includes the addition of 2 new questions to the Home Baseline Assessment.

This amendment approval date does not change the continuing review date for the overall project. Should the research continue, the next progress report must be submitted for review and approval two months prior to the expiration date.

Any amendments or changes to the research protocol including changes to the consent documents and questionnaires/surveys must be submitted to the IRB for review and approval prior to implementation. Also, any change in the status of participants to any of the vulnerable populations (as identified in 45 CFR 46) including, pregnant women, prisoners, or those with diminished capacity, requires IRB review and approval prior to their continuing in the study.

The IRB anticipates that investigators will employ recruitment procedures that allow for the equitable recruitment of women and minorities into research studies.

Note: All research staff must successfully complete the Brown University Education Program in the Protection of Human Research Participants (CITI at http://www.citiprogram.org) prior to beginning work on the project.

*** For Your Information ***

The next deadline for submission of new protocols, amendments to protocols, and annual progress reports requiring full board review is September 30, 2016.

All pertinent Federal and University policies and guidelines related to the involvement of participants in research can be obtained through the Human Research Protection Program at http://www.brown.edu/research/institutional-review-board-irb. This includes the *Belmont Report*, 45 CFR 46, and the Brown University Federal Wide Assurance #00004460.

BROWN

Office of Research Integrity
Box 1986
Providence, RI 02912
Tel: (401) 863-3050
Fax: (401) 863-7292

Memorandum

**To:**	Eric Loucks, Box G-S121-2
**From:**	Human Research Protection Program
**Date:**	January 25, 2018
**RE:**	Protocol Entitled: The Mindfulness-Based Blood Pressure Reduction Study (MB-BP) (#1412001171).<br>Amendment #10

The above amendment to the protocol, received an expedited review and approval by the IRB on January 25, 2018.

This amendment (dated: 11/6/2017) requests approval to (a) modify the approved focus group discussion questions into a one-on-one qualitative phone interview, (b) re-contact Stage 1 participants for a 2-year in-person assessment, (c) rearrange compensation amounts, (d) add recruitment for and data sharing from the UMass Medical School fMRI study, (e) add the "UMass Medical School fMRI Study Recruitment Talking Points," (f) add a consent addendum for enrolled participants to complete the phone interview, and (g) revise the approved consent to reflect the appropriate changes.

This amendment approval date does not change the continuing review date for the overall project. Should the research continue, the next progress report must be submitted for review and approval two months prior to the expiration date.

Any amendments or changes to the research protocol including changes to the consent documents and questionnaires/surveys must be submitted to the IRB for review and approval prior to implementation. Also, any change in the status of participants to any of the vulnerable populations (as identified in 45 CFR 46) including, pregnant women, prisoners, or those with diminished capacity, requires IRB review and approval prior to their continuing in the study.

The IRB anticipates that investigators will employ recruitment procedures that allow for the equitable recruitment of women and minorities into research studies.

Note: All research staff must successfully complete the Brown University Education Program in the Protection of Human Research Participants (CITI at http://www.citiprogram.org) prior to beginning work on the project.

*** For Your Information ***

The next deadline for submission of new protocols, amendments to protocols, and annual progress reports requiring full board review is January 31, 2018.

All pertinent Federal and University policies and guidelines related to the involvement of participants in research can be obtained through the Human Research Protection Program at HRPP. This includes the *Belmont Report*, 45 CFR 46, and the Brown University Federal Wide Assurance #00004460.

Research Protections Office
Box 1986
Providence, RI 02912
Tel: (401) 863-3050
Fax: (401) 863-7292

Memorandum

To: Eric Loucks, Box G-S121-2

From: Research Protections Office

Date: December 17, 2015

RE: Protocol Entitled: Mindfulness-Based Hypertension Therapy Pilot Study (#1412001171).
Continuing Review #1

The above referenced progress report received a full board review and approval by the Brown University IRB on December 17, 2015. Continuation of this study is approved through January 15, 2017.

The Board determined that future progress reports of this full board protocol may be reviewed under expedited category #9.

Should the research continue, the next progress report must be submitted two months prior to the expiration date for review and approval.

Any amendments or changes to the research protocol including changes to the consent documents and questionnaires/surveys must be submitted to the IRB for review and approval prior to implementation. Also, any change in the status of participants to any of the vulnerable populations (as identified in 45 CFR 46) including, pregnant women, prisoners, or those with diminished capacity, requires IRB review and approval prior to their continuing in the study.

The IRB anticipates that investigators will employ recruitment procedures that allow for the equitable recruitment of women and minorities into research studies.

Note: All research staff must successfully complete the Brown University Education Program in the Protection of Human Research Participants (CITI at http://www.citiprogram.org) prior to beginning work on the project.

*** For Your Information ***

The next deadline for submission of new protocols, amendments to protocols, and annual progress reports requiring full board review is January 04, 2016.

All pertinent federal and University policies and guidelines related to the involvement of participants in research can be obtained through the Research Protections Office (RPO) at http://www.brown.edu/research/institutional-review-board-irb. This includes the *Belmont Report*, 45 CFR 46, and the Brown University Federal Wide Assurance #00004460.

BROWN

Office of Research Integrity
Box 1986
Providence, RI 02912
Tel: (401) 863-3050
Fax: (401) 863-7292

Memorandum

To: Eric Loucks, Box G-S121-2

From: Human Research Protection Program

Date: December 16, 2016

RE: Protocol Entitled: The Mindfulness-Based Blood Pressure Reduction Study (MB-BP) (#1412001171).
Continuing Review #2

The above referenced progress report received an expedited review and approval under Expedited Category 9 by the Brown University IRB on December 15, 2016. Continuation of this study is approved through January 15, 2018.

Should the research continue, the next progress report must be submitted two months prior to the expiration date for review and approval.

Any amendments or changes to the research protocol including changes to the consent documents and questionnaires/surveys must be submitted to the IRB for review and approval prior to implementation. Also, any change in the status of participants to any of the vulnerable populations (as identified in 45 CFR 46) including, pregnant women, prisoners, or those with diminished capacity, requires IRB review and approval prior to their continuing in the study.

The IRB anticipates that investigators will employ recruitment procedures that allow for the equitable recruitment of women and minorities into research studies.

Note: All research staff must successfully complete the Brown University Education Program in the Protection of Human Research Participants (CITI at http://www.citiprogram.org) prior to beginning work on the project.

*** For Your Information ***

The next deadline for submission of new protocols, amendments to protocols, and annual progress reports requiring full board review is January 03, 2017.

All pertinent Federal and University policies and guidelines related to the involvement of participants in research can be obtained through the Human Research Protection Program at

HRPP. This includes the *Belmont Report*, 45 CFR 46, and the Brown University Federal Wide Assurance #00004460.

Office of Research Integrity
Box 1986
Providence, RI 02912
Tel: (401) 863-3050
Fax: (401) 863-7292

BROWN

Memorandum

**To:**	Eric Loucks, Box G-S121-2
**From:**	Human Research Protection Program
**Date:**	January 02, 2018
**RE:**	Protocol Entitled: The Mindfulness-Based Blood Pressure Reduction Study (MB-BP) (#1412001171).<br>Continuing Review #3

The above referenced progress report received an expedited review and approval under Expedited Category 9 by the Brown University IRB on January 02, 2018. Continuation of this study is approved through January 15, 2019.

Should the research continue, the next progress report must be submitted two months prior to the expiration date for review and approval.

Any amendments or changes to the research protocol including changes to the consent documents and questionnaires/surveys must be submitted to the IRB for review and approval prior to implementation. Also, any change in the status of participants to any of the vulnerable populations (as identified in 45 CFR 46) including, pregnant women, prisoners, or those with diminished capacity, requires IRB review and approval prior to their continuing in the study.

The IRB anticipates that investigators will employ recruitment procedures that allow for the equitable recruitment of women and minorities into research studies.

Note: All research staff must successfully complete the Brown University Education Program in the Protection of Human Research Participants (CITI at http://www.citiprogram.org) prior to beginning work on the project.

*** For Your Information ***

The next deadline for submission of new protocols, amendments to protocols, and annual progress reports requiring full board review is January 31, 2018.

All pertinent Federal and University policies and guidelines related to the involvement of participants in research can be obtained through the Human Research Protection Program at

HRPP. This includes the *Belmont Report*, 45 CFR 46, and the Brown University Federal Wide Assurance #00004460.

Office of Research Integrity
Box 1986
Providence, RI 02912
Tel: (401) 863-3050
Fax: (401) 863-7292

Memorandum

To: Eric Loucks, Box G-S121-2

From: Human Research Protection Program

Date: January 09, 2019

RE: Protocol Entitled: The Mindfulness-Based Blood Pressure Reduction Study (MB-BP) (#1412001171).
Continuing Review #4

The above referenced progress report received an expedited review and approval under Expedited Category 9 by the Brown University IRB on January 09, 2019. Continuation of this study is approved through January 15, 2020.

Should the research continue, the next progress report must be submitted two months prior to the expiration date for review and approval.

Any amendments or changes to the research protocol including changes to the consent documents and questionnaires/surveys must be submitted to the IRB for review and approval prior to implementation. Also, any change in the status of participants to any of the vulnerable populations (as identified in 45 CFR 46) including, pregnant women, prisoners, or those with diminished capacity, requires IRB review and approval prior to their continuing in the study.

The IRB anticipates that investigators will employ recruitment procedures that allow for the equitable recruitment of women and minorities into research studies.

Note: All research staff must successfully complete the Brown University Education Program in the Protection of Human Research Participants (CITI at http://www.citiprogram.org) prior to beginning work on the project.

*** For Your Information ***

The next deadline for submission of new protocols, amendments to protocols, and annual progress reports requiring full board review is January 31, 2019.

All pertinent Federal and University policies and guidelines related to the involvement of participants in research can be obtained through the Human Research Protection Program at

HRPP. This includes the *Belmont Report*, 45 CFR 46, and the Brown University Federal Wide Assurance #00004460.

cc: Webb, Julie
